# Access to a new class of synthetic building blocks *via* trifluoromethoxylation of pyridines and pyrimidines†
†Electronic supplementary information (ESI) available: Experimental procedures and analysis data for new compounds. See DOI: 10.1039/c5sc02983j


**DOI:** 10.1039/c5sc02983j

**Published:** 2015-10-07

**Authors:** Pengju Feng, Katarzyna N. Lee, Johnny W. Lee, Chengbo Zhan, Ming-Yu Ngai

**Affiliations:** a Department of Chemistry , Stony Brook University , Stony Brook , New York 11794-3400 , USA; b Institute of Chemical Biology and Drug Discovery , Stony Brook University , Stony Brook , New York 11794-3400 , USA . Email: ming-yu.ngai@stonybrook.edu

## Abstract

Since the first synthesis of trifluoromethyl ethers in 1935, the trifluoromethoxy (OCF_3_) group has made a remarkable impact in medicinal, agrochemical, and materials science research. However, our inability to facilely incorporate the OCF_3_ group into molecules, especially heteroaromatics, has limited its potential across a broad spectrum of technological applications. Herein, we report a scalable and operationally simple protocol for regioselective trifluoromethoxylation of a wide range of functionalized pyridines and pyrimidines under mild reaction conditions. The trifluoromethoxylated products are useful scaffolds that can be further elaborated by amidation and palladium-catalysed cross coupling reactions. Mechanistic studies suggest that a radical *O*-trifluoromethylation followed by the OCF_3_-migration reaction pathway is operable. Given the unique properties of the OCF_3_ group and the ubiquity of pyridine and pyrimidine in biologically active molecules and functional materials, trifluoromethoxylated pyridines and pyrimidines could serve as valuable building blocks for the discovery and development of new drugs, agrochemicals, and materials.

## Abstract 2

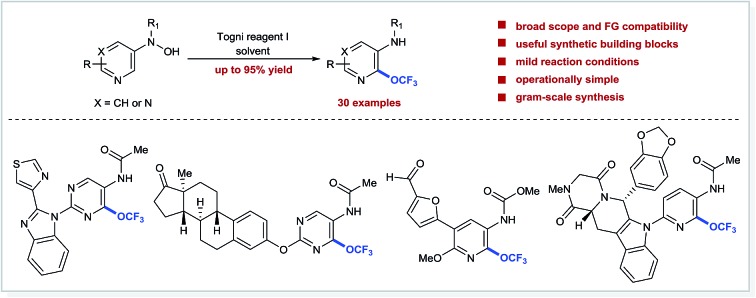
One-pot trifluoromethoxylation of functionalized pyridines and pyrimidines *via* OCF_3_-migraion.

## Introduction

**Fig. 1:**
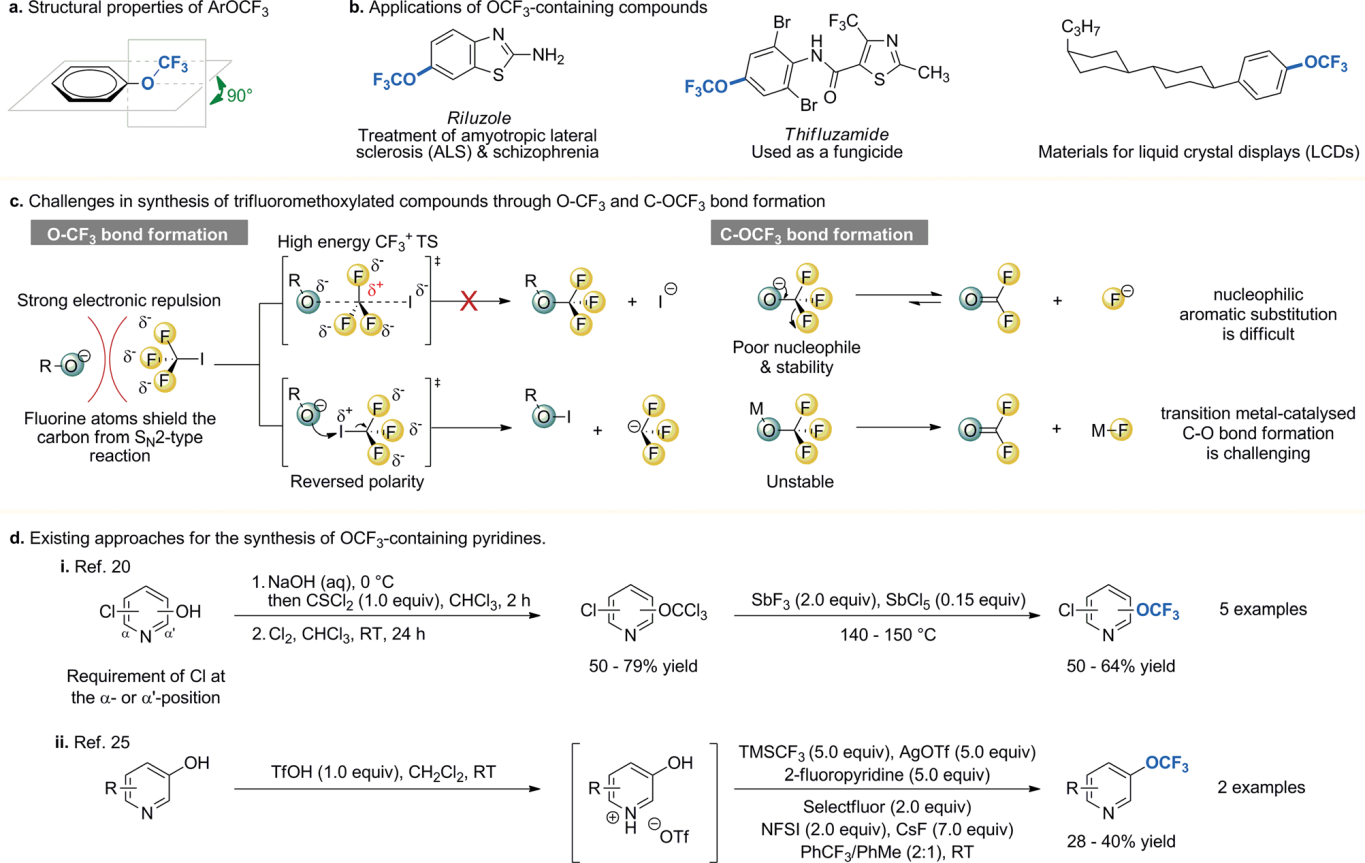
Properties, applications, synthetic challenges and methods of synthesis of OCF_3_-containing compounds. NFSI = *N*-fluorobenzenesulfonimide, TMSCF_3_ = trifluoromethyltrimethylsilane.

The trifluoromethoxy (OCF_3_) group has made a significant impact in medicinal, agrochemical, life- and materials science research^1^–^5^ since Booth and Burchfield reported the first synthesis of trifluoromethyl ethers in 1935.^6^ The increasing importance of the OCF_3_ group can be attributed to its unique structural and electronic properties. First of all, in aryl trifluoromethyl ethers the OCF_3_ moiety lies in the plane orthogonal to arene ring (Fig. 1a)^7^ and studies have shown that this unusual orientation may be beneficial for providing additional binding affinity in drug–target complexes.^8^ In addition, the OCF_3_ group is among the most electronegative groups (*χ*(*F*) = 4.0, *χ*(OCF_3_) = 3.7).^9^ Molecules bearing an electron-withdrawing group have better metabolic stability. Moreover, the OCF_3_ group has an excellent lipophilicity (π_*x*_(SCF_3_) = +1.44, π_*x*_(SF_5_) = +1.23, π_*x*_(OCF_3_) = +1.04, π_*x*_(CF_3_) = +0.88, π_*x*_(OCH_3_) = –0.02);^10^ compounds with higher lipophilicity show enhancement in their *in vivo* uptake and transport in biological systems. Therefore, the OCF_3_ group is introduced into biologically active molecules to improve their efficacy and minimize their side effects (Fig. 1b).^1^,^2^,^5^ Furthermore, incorporation of the OCF_3_ group into organic molecules can increase their melting point and boiling point difference under ambient pressure, and lower their surface tension, dielectric constant, and pour point.^1^,^11^,^12^ These properties are particularly useful in designing electronic devices and materials; as a result, the OCF_3_-containing molecules can be found in electro-optical materials used for the development of liquid crystal displays,^13^ soluble organic semi-conductor,^14^ and melt-processable fluoropolymers such as perfluoroalkoxy alkanes.^12^

Given the unique properties of the OCF_3_ group and the ubiquity of pyridines and pyrimidines in biologically active molecules and functional materials, trifluoromethoxylated pyridines and pyrimidines could serve as valuable synthetic building blocks for the discovery and development of new drugs, agrochemicals, and functional materials. However, synthesis of OCF_3_ containing heteroarenes through either O–CF_3_ or C–OCF_3_ bond formation remains a formidable challenge in organic synthesis (Fig. 1c).^1^–^5^,^15^ Unlike its analogous methoxy (OCH_3_) group, the OCF_3_ group cannot be formed *via* trifluoromethylation of hard nucleophiles such as phenoxides with CF_3_I through S_N_2 type mechanism.^11^,^16^,^17^ This is due to (i) strong electron repulsion between three fluorine atoms and an incoming nucleophile; (ii) formation of energetically disfavoured CF_3_ carbocation transition state structure (TS); and (iii) competing iodination of nucleophiles due to the reversed electron density. In addition, the thermal instability of transition metal–OCF_3_ complexes (they readily decompose to form fluorophosgene and metal fluoride)^18^ and the poor nucleophilicity of the OCF_3_ anion (a reactive electrophile is needed for the C–OCF_3_ bond formation)^19^ have hampered the development of the C–OCF_3_ bond formation through either transition metal-catalysed C–O bond formation or nucleophilic substitution. Strategies for the synthesis of trifluoromethoxylated heteroaromatic compounds are very rare.^20^–^25^ Leroux and co-workers reported a detailed examination of several different approaches and concluded that the presence of a chlorine atom at the α-, and/or α′- position of hydroxy-pyridines is critical (Fig. 1d).^20^ Without it, little or no desired product was isolated. This requirement greatly limited its application. Recently, Qing and co-workers reported a novel, direct synthesis of pyridyl trifluoromethyl ethers from unprotected hydroxypyridines.^25^ However, excess amounts of reagents and oxidants were required. In addition, only two examples with moderate yield were reported. Due to the lack of a general synthetic method for the synthesis of trifluoromethoxylated pyridines and pyrimidines, their full potential has not been fully exploited in pharmaceutical, agrochemical, and materials applications.

Herein, we report a scalable and operationally simple protocol for regioselective synthesis of trifluoromethoxylated functionalized pyridines and pyrimidines. Several unique features distinguish our strategy from the existing approaches: (i) many substrates with complex skeletons are trifluoromethoxylated at or below room temperature (17 out of the 30 examples); (ii) a wide range of functional groups and substitution patterns are tolerated; (iii) this transformation is amenable to gram-scale synthesis; (iv) halogen or amino group is used as synthetic handles for further elaborations, and (v) the operational simplicity of our protocol would render trifluoromethoxylation available to broader synthetic community. More importantly, this strategy allows access to a new class of synthetic building blocks to aid the discovery and development of new functional molecules.

## Results and discussion

It is known that the N–O bond is relatively weak (bond dissociation energy = ∼57 kcal mol^–1^) due to the lone-pair electron repulsion between the nitrogen and the oxygen atoms. In addition, electron withdrawing *O*-substituent and/or electron donating *N*-substituent could promote heterolytic cleavage of the N–O bond to form nitrenium ion and oxy-anion.^26^ Recently, we took advantage of these properties and successfully synthesized trifluoromethoxylated aromatic compounds through *O*-trifluoromethylation of *N*-aryl-*N*-hydroxylamine derivatives to form N-OCF_3_ compounds followed by thermally induced OCF_3_-migration.^27^ However, to apply this strategy to the synthesis of trifluoromethoxylated heteroaromatic compounds such as pyridine and pyrimidine, two challenges had to be addressed.

First of all, reaction conditions for the synthesis of *N-*acetyl/methoxycarbonyl-*N*-pyridinylhydroxylamine precursors are very limited,^28^–^30^ because the presence of heteroarenes complicates their synthesis. For instance, reduction of nitro-pyridines to the corresponding *N*-pyridinyl-*N*-hydroxylamines often accompanied by over reduction side-products (*i.e.* formation of aminopyridines). In addition, formation of undesired bis-protected side products and pyridinium salts during the protection of *N*-pyridinyl-*N*-hydroxylamines lowered the yields of precursors. After extensive optimization, we were able to obtain pure precursors in good to excellent yields. We have identified a robust catalytic hydrazine reduction protocol (using 5% rhodium on carbon as a catalyst) for converting nitropyridines and pyrimidines to *N*-heteroaryl-*N*-hydroxylamines. This method is general and high yielding; most of the reduction products can be used directly without further purification (only filtration through celite to remove Rh/C followed by the removal of the solvent is required). We have also observed that isolation of the intermediate hydroxylamines is not necessary; subsequent *N*-protection can be performed one-pot and the selectivity can be controlled by the rate of addition of acyl chloride or methylchloroformate at appropriate temperature (see ESI† for detail experimental procedures).

Another challenge was that the *O*-trifluoromethylation and OCF_3_-migration processes for the protected *N*-heteroaryl-*N*-hydroxylamine derivatives were inefficient under our previously developed reaction conditions. Exposure of methyl (5-bromo-6-methoxypyridin-3-yl)(hydroxyl)carbamate (**1a**) to 1-trifluoromethyl-1,2-benziodoxol-3(1*H*)-one (Togni reagent II, **B**)^31^,^32^ in the presence of cesium carbonate (Cs_2_CO_3_, 0.1 equiv.) in chloroform (CHCl_3_) at room temperature for 15 hours afforded the desired product **2a** in only 29% yield (Table 1, entry 1).^27^ Examination of different trifluoromethylation reagents such as 3,3-dimethyl-1-(trifluoromethyl)-1,2-benziodoxole (Togni reagent I, **A**),^31^ 5-(trifluoromethyl)dibenzothiophenium trifluoromethanesulfonate (Umemoto reagent, **C**),^33^ and [(oxido)phenyl(trifluoromethyl)-λ^4^-sulfanylidene] dimethylammonium tetrafluoroborate (Shibata–Johnson reagent, **D**)^34^ revealed that Togni reagent I was superior to other reagents and delivered the desired product **2a** in 42% yield (entries 2–4). Higher yield was obtained in the absence of Cs_2_CO_3_ (entry 5). Screening of solvents showed that CH_2_Cl_2_ gave the best result, providing the desired product **2a** in 87% NMR yield (entries 6–10).

**Table 1:** Optimization of trifluoromethoxylation of **1a**

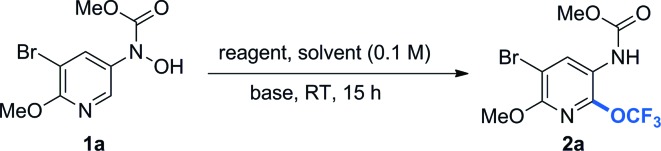				
Entry	Reagent	Base (0.1 equiv.)	Solvent	Yield^*a*^
1	**B**	Cs_2_CO_3_	CHCl_3_	29%
2	**A**	Cs_2_CO_3_	CHCl_3_	42%
3	**C**	Cs_2_CO_3_	CHCl_3_	10%
4	**D**	Cs_2_CO_3_	CHCl_3_	4%
5	**A**	—	CHCl_3_	72%
6	**A**	—	DMF	24%
7	**A**	—	THF	48%
8	**A**	—	CH_3_CN	69%
9	**A**	—	CH_3_NO_2_	74%
10	**A**	—	CH_2_Cl_2_	87%
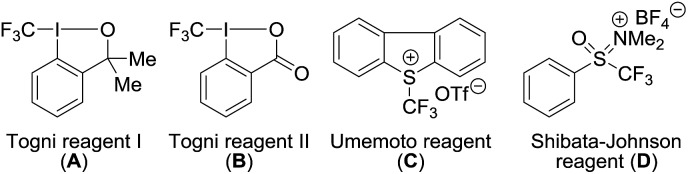				

^*a*^Yields were determined by ^1^H-NMR analysis using benzotrifluoride as the internal standard.

With the optimized reaction conditions in hand, we next directed our attention to exploring the scope and generality of the trifluoromethoxylation reaction. Gratifyingly, a wide range of functional groups and substitution patterns were tolerated (Table 2). Halogen functionalities remain intact after the reaction, providing useful synthetic handles for further functionalization (**2a–h**, **2k**, **2r**, **2s**, **2u**, **2v**). Other functional groups such as alkyl- and aryl-ethers (**2a**, **2b**, **2i**, **2k–m**, **2v–x**), aldehydes (**2m**), ketone (**2x**), alkene (**2v**), alkyne (**2w**), amide (**2y**), esters (**2v–w**), and benzo[1,3]dioxole (**2y**) proved compatible under the reaction conditions. In addition, this methodology was successfully applied to pyridines bearing a wide array of heteroaryl substituents such as furan (**2m**), pyrazole (**2n**), 1,2,4-triazole (**2o**), benzimidazole (**2p**), benzotriazole (**2q**), indole (**2r**, **2y**), 7-azaindole (**2s**), thiazole (**2t**), and 2,6-dichloropurine (**2u**). These products could be useful building blocks for drug and agrochemical discovery and development because it is estimated that over 70% of all pharmaceutical products bear heterocyclic moieties.^35^ More excitingly, our mild reaction conditions allow late-stage trifluoromethoxylation of complex organic molecules. For examples, estrone and Tadalafil (Cialis, sales in 2013: US$2.159 billion)^36^ conjugated pyridines (**1x**, **1y**) were trifluoromethoxylated to afford desired products **2x** and **2y** in 71% and 66% yield, respectively. Remarkably, no epimerization was observed under our reaction conditions. These results further demonstrated the synthetic utility of our strategy.

**Table 2:** Selected examples of trifluoromethoxylation of pyridines

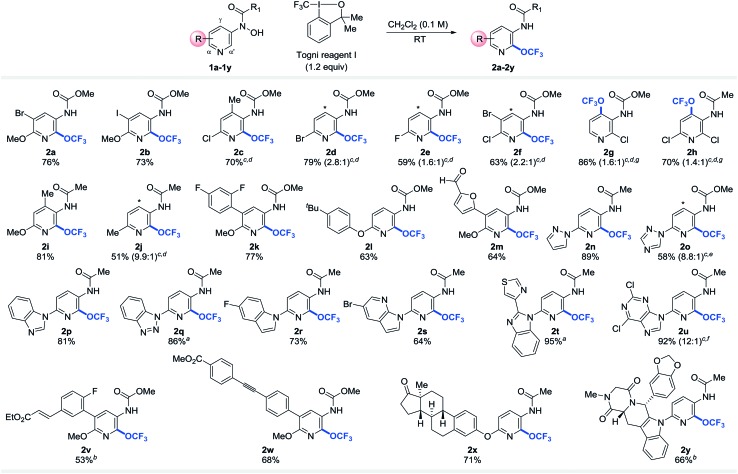

^*a*^Cited yields and isomeric ratios are of isolated material by column chromatography. RT then 50 °C in CH_2_Cl_2_.

^*b*^4 °C in CH_2_Cl_2_ (0.01 M).

^*c*^Following the *O*-trifluoromethylation reaction in CH_2_Cl_2_ at RT, the reaction mixture was concentrated, the residue was dissolved in MeNO_2_, and the resulting mixture was heated.

^*d*^120 °C.

^*e*^80 °C.

^*f*^60 °C.

^*g*^Atropisomeric ratio.

Several features of the reaction are noteworthy. First of all, the reaction is sensitive to the electronic properties of substituents on pyridine. Substrates with an electron donating substituent *para* to the protected *N*-hydroxylamine readily undergo rearrangement to yield the desired products of trifluoromethoxylation at or below room temperature (**2a–b**, **2i**, **2k–n**, **2p**, **2r–2s**, **2v–y**). In the absence of such substituents, higher reaction temperatures are required for the OCF_3_-migration step (**2c–h**, **2o**, **2q**, **2t–u**). These observations are consistent with the formation of nitrenium ion through heterolytic cleavage of N–O bond (*vide infra*).^26^ Secondly, for the reactions that take place at or below room temperature, the OCF_3_ group is introduced exclusively to the α′-position.^37^–^39^ Since α- and α′-carbon of pyridines are metabolically labile sites, incorporation of an electron withdrawing OCF_3_ group to the α′-position could improve their metabolic stability.^40^,^41^ If the α′-position is blocked, product of γ-OCF_3_ pyridine is formed instead (**2g** and **2h**). Interestingly, atropisomers are obtained in these cases. This is because the OCF_3_ group lies in the plane orthogonal to the pyridine ring (Fig. 1a), which prevents the free rotation of the adjacent amide or carbamate group (see ESI†).^7^,^42^–^45^ Finally, the regioselectivity erodes as the reaction temperature increases (**2d–f**, **2o**, **2u**).

To probe the applicability of the trifluoromethoxylation reaction to other heteroarenes, pyrimidines substituted with benzimidazolyl (**3a**), indolyl (**3b**), methoxy (**3c**), phenoxy (**3d**), or estronyl (**3e**) groups were examined (Table 3). To our delight, these substrates were trifluoromethoxylated to afford the corresponding desired products (**4a–4e**) in good yields. Notably, none of the trifluoromethoxylated pyridines and pyrimidines reported here has ever been prepared before.

**Table 3:** Selected examples of trifluoromethoxylation of pyrimidines

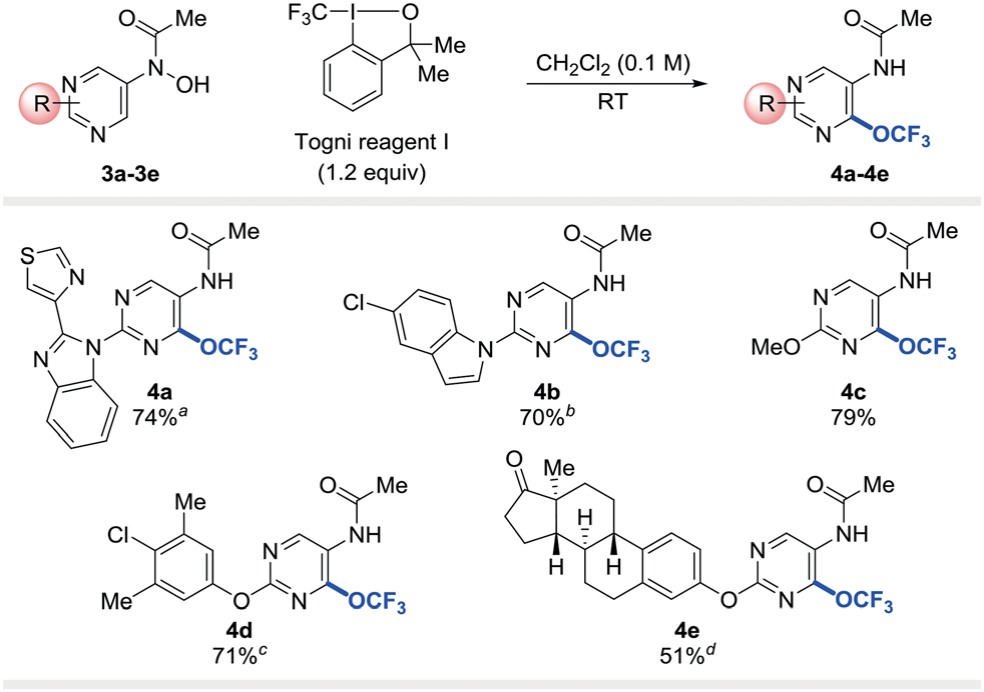

^*a*^Cited yields are of isolated material by column chromatography. Following the *O*-trifluoromethylation reaction in CH_2_Cl_2_ at RT, the reaction mixture was concentrated, the residue was dissolved in MeNO_2_, and the resulting mixture was heated at 80 °C.

^*b*^CH_2_Cl_2_ (0.01 M).

^*c*^RT then 50 °C in CH_2_Cl_2_ (0.03 M).

^*d*^RT then 50 °C in CH_2_Cl_2_.

**Scheme 1:**
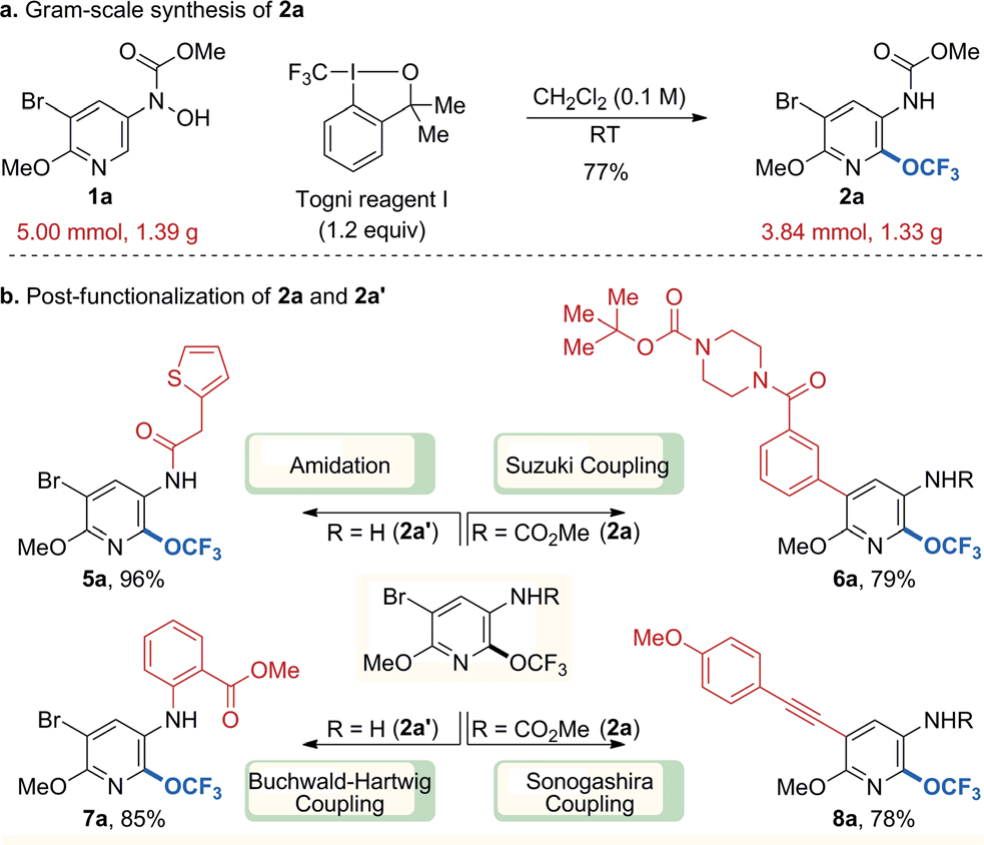
Gram-scale synthesis and post-functionalization. Cited yields are of isolated material by column chromatography.

To ensure that our products can serve as useful building blocks for molecular screening, our protocol must be scalable and further functionalization of the trifluoromethoxylated products must be possible. To evaluate the reaction efficacy on preparative scale, a gram-scale reaction of **1a** (1.39 g, 5.00 mmol) was performed (Scheme 1a) and the efficiency of the small-scale reaction was retained upon scale-up. Our trifluoromethoxylated products also proved to be versatile (Scheme 1b). For instance, **2a** could be further elaborated through palladium-catalysed Suzuki and Sonogashira couplings to afford the desired products (**6a**, **8a**) in good yields. In addition, deprotected amino-pyridine (**2a′**) could be efficiently incorporated into other molecules through amidation and palladium-catalysed Buchwald–Hartwig coupling (**5a**, **7a**).

Although our strategy is operationally simple and scalable, has broad substrate scope, and tolerates a wide range of functional groups, this procedure, much like other methods, is not without limitations. First of all, substrates with the protected *N*-hydroxylamino-group at α-, γ-, or α′-position do not give the product of trifluoromethoxylation. Presumably, the formation of nitrenium ion is energetically disfavoured in these cases, because it involves placing the positive charge on the endocyclic nitrogen atom.^46^ In addition, preparation of *N*-heteroaryl-*N*-hydroxylamine precursors is required for this transformation. Furthermore, Togni reagent I is relatively expensive, and thus large-scale synthesis would be costly. Therefore, more improvements are needed for the development of a truly general and industrially practical trifluoromethoxylation reaction. Nevertheless, with the method accessing unprecedented and versatile synthetic building blocks in hand, the discovery and development of new pharmaceuticals, agrochemicals, and functional materials can be expected.

**Scheme 2:**
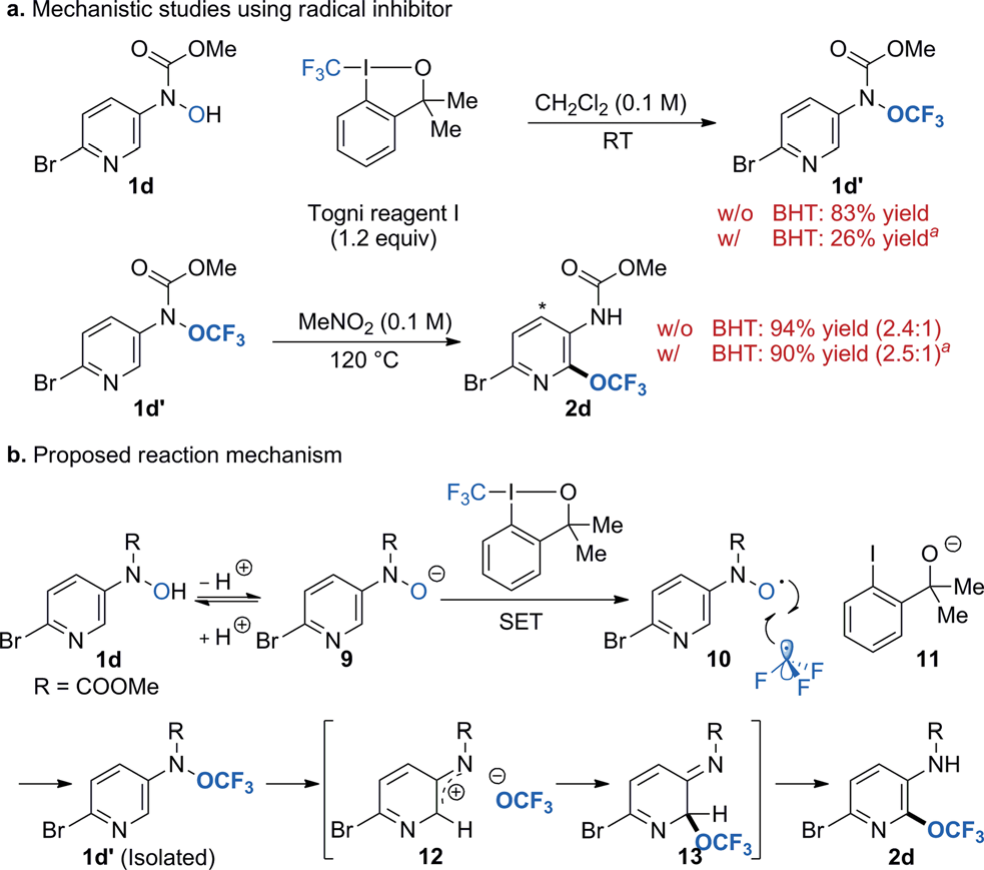
Mechanistic studies and proposed reaction mechanism. ^*a*^1 equiv. of BHT was used. Yields were determined by ^1^H-NMR analysis using benzotrifluoride as the internal standard. BHT = butylated hydroxytoluene. w/ = with. w/o = without.

To gain some insight into the reaction mechanism, we performed reactions in the presence of radical trap butylated hydroxytoluene (BHT) (Scheme 2a). We chose to use substrate **1d** because we could isolate the *O*-trifluoromethylated intermediate **1d′** and study each step (*i.e. O*-trifluoromethylation and OCF_3_-migration) separately. Addition of BHT (1 equiv.) to a reaction mixture of **1d** and Togni reagent I had detrimental effect to the formation of *O*-trifluoromethylated *N*-hydroxylamine intermediate **1d′**. This result strongly suggests the involvement of radical species in the reaction pathway, which is in agreement with literature precedents.^47^,^48^ On the other hand, BHT did not affect the reaction yield for the OCF_3_-migration process (step 2, Scheme 2a). These experiments argue against the presence of long-lived radical species in the OCF_3_-migration process and are consistent with our previous finding.^27^ Moreover, introduction of electron rich substituent para to the N-OCF_3_ group facilitates the OCF_3_-migration process. These observations support the formation of nitrenium ion and trifluoromethoxide.^26^,^27^ On the basis of these results, a plausible mechanism for the trifluoromethoxylation reaction is illustrated in Scheme 2b. Deprotonation of **1d** forms *N*-hydroxyl anion **9**, which undergoes single-electron transfer (SET) with Togni reagent I to generate *N*-hydroxyl radical **10**, trifluoromethyl radical, and alkoxide **11**.^48^ Reaction of *N*-hydroxyl radical and trifluoromethyl radical affords the *O*-trifluoromethylated hydroxylamine **1d′**, which could be isolated and characterized. This intermediate will then undergo thermally induced heterolytic cleavage of the N–O bond to form a tight ion pair of nitrenium ion **12** and trifluoromethoxide. Rapid recombination of this ion pair gives **13**, which upon tautomerization (*i.e.* migration of proton) yields the desired product **2d**.

## Conclusions

In summary, we reported an operationally simple protocol for the regioselective trifluoromethoxylation of functionalized pyridines and pyrimidines. The strategy uses commercially available and bench stable Togni reagent I, features a broad substrate scope, tolerates a wide range of functional groups, and is amenable to gram scale synthesis. With this procedure, a variety of highly functionalized pyridines and pyrimidines could be trifluoromethoxylated under mild reaction conditions. Since heteroarenes are ubiquitous in biologically active natural products, pharmaceuticals, and agrochemicals, we expect that our work will provide valuable OCF_3_-containing heteroaromatic building blocks for the discovery and development of new drugs, agrochemicals, and functional materials.

## Supplementary Material

Supplementary information

## References

[cit1] Leroux F., Jeschke P., Schlosser M. (2005). Chem. Rev..

[cit2] Jeschke P., Baston E., Leroux F. R. (2007). Mini-Rev. Med. Chem..

[cit3] Leroux F. R., Manteau B., Vors J. P., Pazenok S. (2008). Beilstein J. Org. Chem..

[cit4] Manteau B., Pazenok S., Vors J. P., Leroux F. R. (2010). J. Fluorine Chem..

[cit5] Landelle G., Panossian A., Leroux F. R. (2014). Curr. Top. Med. Chem..

[cit6] Booth H. S., Burchfield P. E. (1935). J. Am. Chem. Soc..

[cit7] Federsel D., Herrmann A., Christen D., Sander S., Willner H., Oberhammer H. (2001). J. Mol. Struct..

[cit8] Muller K., Faeh C., Diederich F. (2007). Science.

[cit9] Mcclinton M. A., Mcclinton D. A. (1992). Tetrahedron.

[cit10] Hansch C. and Leo A., Substituent Constants for Correlation Analysis in Chemistry and Biology, Wiley, New York, 1979. 10.1021/jm00212a024 836503

[cit11] Kirsch P., Modern Fluoroorganic Chemistry : Synthesis, Reactivity, Applications, Wiley-VCH, Weinheim, 2004.

[cit12] Siegemund G., Schwertfeger W., Feiring A., Smart B., Behr F., Vogel H. and McKusick B., in Ullmann's Encyclopedia of Industrial Chemistry, Wiley-VCH Verlag GmbH & Co. KGaA, 2000, 10.1002/14356007.a11_349.

[cit13] Kirsch P., Bremer M. (2000). Angew. Chem., Int. Ed..

[cit14] Mamada M., Shima H., Yoneda Y., Shimano T., Yamada N., Kakita K., Machida T., Tanaka Y., Aotsuka S., Kumaki D., Tokito S. (2015). Chem. Mater..

[cit15] Liang T., Neumann C. N., Ritter T. (2013). Angew. Chem., Int. Ed..

[cit16] Shimizu M., Hiyama T. (2005). Angew. Chem., Int. Ed..

[cit17] Umemoto T., Adachi K., Ishihara S. (2007). J. Org. Chem..

[cit18] Zhang C. P., Vicic D. A. (2012). Organometallics.

[cit19] Kolomeitsev A. A., Vorobyev M., Gillandt H. (2008). Tetrahedron Lett..

[cit20] Manteau B., Genix P., Brelot L., Vors J. P., Pazenok S., Giornal F., Leuenberger C., Leroux F. R. (2010). Eur. J. Org. Chem..

[cit21] Fuss A., Koch V. (1990). Synthesis.

[cit22] Morimoto K., Makino K., Sakata G. (1992). J. Fluorine Chem..

[cit23] Guiadeen D., Kothandaraman S., Yang L. H., Mills S. G., MacCoss M. (2008). Tetrahedron Lett..

[cit24] Sokolenko T. M., Davydova Y. A., Yagupolskii Y. L. (2012). J. Fluorine Chem..

[cit25] Liu J.-B., Chen C., Chu L., Chen Z.-H., Xu X.-H., Qing F.-L. (2015). Angew. Chem., Int. Ed..

[cit26] Tabolin A. A., Ioffe S. L. (2014). Chem. Rev..

[cit27] Hojczyk K. N., Feng P., Zhan C., Ngai M.-Y. (2014). Angew. Chem., Int. Ed..

[cit28] Novak M., Nguyen T. M. (2003). J. Org. Chem..

[cit29] Rajagopal S., Brooks M. E., Nguyen T. M., Novak M. (2003). Tetrahedron.

[cit30] Novak M., Toth K., Rajagopal S., Brooks M., Hott L. L., Moslener M. (2002). J. Am. Chem. Soc..

[cit31] Eisenberger P., Gischig S., Togni A. (2006). Chem.–Eur. J..

[cit32] Matousek V., Pietrasiak E., Schwenk R., Togni A. (2013). J. Org. Chem..

[cit33] Umemoto T. (1996). Chem. Rev..

[cit34] Noritake S., Shibata N., Nakamura S., Toru T., Shiro M. (2008). Eur. J. Org. Chem..

[cit35] Bartoli G., Dalpozzo R., Nardi M. (2014). Chem. Soc. Rev..

[cit36] Weber F., Sedelmeier G. (2014). Nachr. Chem..

[cit37] Novikov V. N., Pozharskii A. F., Doronkin V. N. (1976). Khim. Geterotsikl. Soedin..

[cit38] Hirota M., Masuda H., Hamada Y., Takeuchi I. (1979). Bull. Chem. Soc. Jpn..

[cit39] Mcgill C. K., Rappa A. (1988). Adv. Heterocycl. Chem..

[cit40] Ishida H., Isami S., Matsumura T., Umehara H., Yamashita Y., Kajita J., Fuse E., Kiyoi H., Naoe T., Akinaga S., Shiotsu Y., Arai H. (2008). Bioorg. Med. Chem. Lett..

[cit41] St Jean D. J., Fotsch C. (2012). J. Med. Chem..

[cit42] Shishkov I. F., Geise H. J., van Alsenoy C., Khristenko L. V., Vilkov L. V., Senyavian V. M., van der Veken B., Herrebout W., Lokshin B. V., Garkusha O. G. (2001). J. Mol. Struct..

[cit43] Kapustin E. G., Bzhezovsky V. M., Yagupolskii L. M. (2002). J. Fluorine Chem..

[cit44] Klocker J., Karpfen A., Wolschann P. (2003). Chem. Phys. Lett..

[cit45] Böhm H. J., Banner D., Bendels S., Kansy M., Kuhn B., Müller K., Obst-Sander U., Stahl M. (2004). ChemBioChem.

[cit46] Sullivan M. B., Cramer C. J. (2000). J. Am. Chem. Soc..

[cit47] Li Y., Studer A. (2012). Angew. Chem., Int. Ed..

[cit48] Matoušek V., Pietrasiak E., Sigrist L., Czarniecki B., Togni A. (2014). Eur. J. Org. Chem..

