# Supplementary material for: Access to a new class of synthetic building blocks via trifluoromethoxylation of pyridines and pyrimidines
Source: Chem Sci. 2015 Oct 7;7(1):424–9. doi: 10.1039/c5sc02983j (PMC5110255; doi:10.1039/c5sc02983j)

Electronic Supplementary Material (ESI) for Chemical Science.
This journal is © The Royal Society of Chemistry 2015

Supporting Information

Access to a New Class of Synthetic Building Blocks *via* Trifluoromethoxylation of Pyridines and Pyrimidines

Pengju Feng,‡ Katarzyna N. Lee,‡ Johnny W. Lee, Chengbo Zhan, and Ming-Yu Ngai*

Department of Chemistry, Stony Brook University, Stony Brook, NY 11794-3400

Institute of Chemical Biology and Drug Discovery, Stony Brook University, Stony Brook, New York 11794-3400

E-mail: ming-yu.ngai@stonybrook.edu

Table of Contents

Materials and Methods 5

Experimental Data 6

Optimization of the one-pot synthesis of protected *N*-heteroaryl-*N*-hydroxylamines 6

Methyl (5-bromo-6-methoxypyridin-3-yl)(hydroxy)carbamate (1a) 7

N-(5-Iodo-6-methoxypyridin-3-yl)hydroxylamine (S1) 7

Methyl hydroxy(5-iodo-6-methoxypyridin-3-yl)carbamate (1b) 8

N-(6-Chloro-4-methylpyridin-3-yl)hydroxylamine (S2) 8

Methyl (6-chloro-4-methylpyridin-3-yl)(hydroxy)carbamate (1c) 9

N-(6-Bromopyridin-3-yl)hydroxylamine (S3) 9

Methyl (6-bromopyridin-3-yl)(hydroxy)carbamate (1d) 10

Methyl (6-fluoropyridin-3-yl)(hydroxy)carbamate (1e) 10

N-(5-Bromo-6-chloropyridin-3-yl)hydroxylamine (S4) 11

Methyl (5-bromo-6-chloropyridin-3-yl)(hydroxy)carbamate (1f) 11

N-(2-Chloropyridin-3-yl)hydroxylamine (S5) 12

Methyl (2-chloropyridin-3-yl)(hydroxy)carbamate (1g) 12

N-(2,6-Dichloropyridin-3-yl)-*N*-hydroxyacetamide (1h) 13

N-Hydroxy-*N*-(6-methoxy-4-methylpyridin-3-yl)acetamide (1i) 13

N-(6-Methylpyridin-3-yl)hydroxylamine (S6) 14

N-Hydroxy-*N*-(6-methylpyridin-3-yl)acetamide (1j) 14

3-(2,4-Difluorophenyl)-2-methoxy-5-nitropyridine (S7) 15

N-(5-(2,4-Difluorophenyl)-6-methoxypyridin-3-yl)hydroxylamine (S8) 15

Methyl (5-(2,4-difluorophenyl)-6-methoxypyridin-3-yl)(hydroxy)carbamate (1k) 16

2-(4-(*tert*-Butyl)phenoxy)-5-nitropyridine (S9) 16

N-(6-(4-(*tert*-Butyl)phenoxy)pyridin-3-yl)hydroxylamine (S10) 17

Methyl (6-(4-(*tert*-butyl)phenoxy)pyridin-3-yl)(hydroxy)carbamate (1l) 18

Methyl (5-(5-formylfuran-2-yl)-6-methoxypyridin-3-yl)(hydroxy)carbamate (1m) 18

5-Nitro-2-(1*H*-pyrazol-1-yl)pyridine (S11) 19

N-(6-(1*H*-Pyrazol-1-yl)pyridin-3-yl)-*N*-hydroxyacetamide (1n) 19

5-Nitro-2-(1*H*-1,2,4-triazol-1-yl)pyridine (S12) 20

N-(6-(1*H*-1,2,4-Triazol-1-yl)pyridin-3-yl)hydroxylamine (S13) 20

Methyl (6-(1*H*-1,2,4-triazol-1-yl)pyridin-3-yl)(hydroxy)carbamate (1o) 21

N-(6-(1*H*-Benzo[*d*]imidazol-1-yl)pyridin-3-yl)-*N*-hydroxyacetamide (1p) 22

1-(5-Nitropyridin-2-yl)-1*H*-benzo[*d*][1,2,3]triazole (S15) 22
N-(6-(1*H*-Benzo[*d*][1,2,3]triazol-1-yl)pyridin-3-yl)-*N*-hydroxyacetamide (1q) 23
5-Fluoro-1-(5-nitropyridin-2-yl)-1*H*-indole (S16) 23
N-(6-(5-Fluoro-1*H*-indol-1-yl)pyridin-3-yl)-*N*-hydroxyacetamide (1r) 24
5-Bromo-1-(5-nitropyridin-2-yl)-1*H*-pyrrolo[2,3-*b*]pyridine (S17) 25
N-(6-(5-Bromo-1*H*-pyrrolo[2,3-b]pyridin-1-yl)pyridin-3-yl)-*N*-hydroxyacetamide (1s) 25
4-(1-(5-Nitropyridin-2-yl)-1*H*-benzo[*d*]imidazol-2-yl)thiazole (S18) 26
N-Hydroxy-*N*-(6-(2-(thiazol-4-yl)-1*H*-benzo[d]imidazol-1-yl)pyridin-3-yl)acetamide (1t). 26
2,6-Dichloro-9-(5-nitropyridin-2-yl)-9*H*-purine (S19) 27
N-(6-(2,6-Dichloro-9*H*-purin-9-yl)pyridin-3-yl)-*N*-hydroxyacetamide (1u)........................ 27
Methyl 4-((4-(5-(hydroxy(methoxycarbonyl)amino)-2-methoxypyridin-3-yl)phenyl)ethynyl)benzoate (1w) 28
(8*R*,9*S*,13*S*,14*S*)-13-Methyl-3-((5-nitropyridin-2-yl)oxy)-6,7,8,9,11,12,13,14,15,16-decahydro-17*H*-cyclopenta[*a*]phenanthren-17-one (S20) 29
N-Hydroxy-*N*-(6-(((8*R*,9*S*,13*S*,14*S*)-13-methyl-17-oxo-7,8,9,11,12,13,14,15,16,17-decahydro-6*H*-cyclopenta[*a*]phenanthren-3-yl)oxy)pyridin-3-yl)acetamide (1x) 30
(6*R*,12*aR*)-6-(Benzo[*d*][1,3]dioxol-5-yl)-2-methyl-7-(5-nitropyridin-2-yl)-2,3,6,7,12,12a-hexahydropyrazino[1',2':1,6]pyrido[3,4-b]indole-1,4-dione (S21) 31
N-(6-((6*R*,12a*R*)-6-(Benzo[*d*][1,3]dioxol-5-yl)-2-methyl-1,4-dioxo-1,3,4,6,12,12a-hexahydropyrazino[1',2':1,6]pyrido[3,4-b]indol-7(2*H*)-yl)pyridin-3-yl)-*N*-hydroxyacetamide (1y) 31
4-(1-(5-Nitropyrimidin-2-yl)-1*H*-benzo[*d*]imidazol-2-yl)thiazole (S22) 32
N-Hydroxy-*N*-(2-(2-(thiazol-4-yl)-1*H*-benzo[d]imidazol-1-yl)pyrimidin-5-yl)acetamide (3a) 33
5-Chloro-1-(5-nitropyrimidin-2-yl)-1*H*-indole (S23).. 33
N-(2-(5-Chloro-1*H*-indol-1-yl)pyrimidin-5-yl)-*N*-hydroxyacetamide (3b).......................... 34
N-Hydroxy-*N*-(2-methoxypyrimidin-5-yl)acetamide (3c).. 35
2-(4-Chloro-3,5-dimethylphenoxy)-5-nitropyridine (S24) 35
N-(2-(4-Chloro-3,5-dimethylphenoxy)pyrimidin-5-yl)-*N*-hydroxyacetamide (3d).............. 36
(8*R*,9*S*,13*S*,14*S*)-13-Methyl-3-((5-nitropyrimidin-2-yl)oxy)-6,7,8,9,11,12,13,14,15,16-decahydro-17*H*-Cyclopenta[a]phenanthren-17-one (S25) 36
N-Hydroxy-*N*-(2-(((8*R*,9*S*,13*S*,14*S*)-13-methyl-17-oxo-7,8,9,11,12,13,14,15,16,17-decahydro-6*H*-cyclopenta[*a*]phenanthren-3-yl)oxy)pyrimidin-5-yl)acetamide (3e) 37
Methyl (5-bromo-6-methoxy-2-(trifluoromethoxy)pyridin-3-yl)carbamate (2a).................. 37
Methyl (5-iodo-6-methoxy-2-(trifluoromethoxy)pyridin-3-yl)carbamate (2b)..................... 38

Methyl (6-chloro-4-methyl-2-(trifluoromethoxy)pyridin-3-yl)carbamate (2c) 38

Methyl (6-bromo-2-(trifluoromethoxy)pyridin-3-yl)carbamate (2d) and methyl (6-bromo-4-(trifluoromethoxy)pyridin-3-yl)carbamate (2d-II) 39

Methyl (6-fluoro-2-(trifluoromethoxy)pyridin-3-yl)carbamate (2e) and methyl (6-fluoro-4-(trifluoromethoxy)pyridin-3-yl)carbamate (2e-II)... 39

Methyl (5-bromo-6-chloro-2-(trifluoromethoxy)pyridin-3-yl)carbamate (2f) and methyl (5-Bromo-6-chloro-4-(trifluoromethoxy)pyridin-3-yl)carbamate (2f-II).................................. 40

Methyl (2-chloro-4-(trifluoromethoxy)pyridin-3-yl)carbamate (2g).................................... 41

N-(2,6-Dichloro-4-(trifluoromethoxy)pyridin-3-yl)acetamide (2h) 42

N-(6-Methoxy-4-methyl-2-(trifluoromethoxy)pyridin-3-yl)acetamide (2i) 42

N-(6-Methyl-2-(trifluoromethoxy)pyridin-3-yl)acetamide (2j).. 43

Methyl (5-(2,4-difluorophenyl)-6-methoxy-2-(trifluoromethoxy)pyridin-3-yl)carbamate (2k) 43

Methyl (6-(4-(*tert*-butyl)phenoxy)-2-(trifluoromethoxy)pyridin-3-yl)carbamate (2l) 44

Methyl (5-(5-formylfuran-2-yl)-6-methoxy-2-(trifluoromethoxy)pyridin-3-yl)carbamate (2m) 44

N-(6-(1*H*-Pyrazol-1-yl)-2-(trifluoromethoxy)pyridin-3-yl)acetamide (2n).......................... 45

Methyl (6-(1*H*-1,2,4-triazol-1-yl)-2-(trifluoromethoxy)pyridin-3-yl)carbamate (2o) and methyl (6-(1*H*-1,2,4-triazol-1-yl)-4-(trifluoromethoxy)pyridin-3-yl)carbamate (2o-II)....... 46

N-(6-(1*H*-Benzo[*d*]imidazol-1-yl)-2-(trifluoromethoxy)pyridin-3-yl)acetamide (2p) 47

N-(6-(1*H*-Benzo[*d*][1,2,3]triazol-1-yl)-2-(trifluoromethoxy)pyridin-3-yl)acetamide (2q) ... 47

N-(6-(5-Fluoro-1*H*-indol-1-yl)-2-(trifluoromethoxy)pyridin-3-yl)acetamide (2r) 48

N-(6-(5-Bromo-1*H*-pyrrolo[2,3-*b*]pyridin-1-yl)-2-(trifluoromethoxy)pyridin-3-yl)acetamide (2s).. 48

N-(6-(2-(Thiazol-4-yl)-1*H*-benzo[*d*]imidazol-1-yl)-2-(trifluoromethoxy)pyridin-3-yl)acetamide (2t).. 49

N-(6-(2,6-Dichloro-9*H*-purin-9-yl)-2-(trifluoromethoxy)pyridin-3-yl)acetamide (2u) and *N*-(6-(2,6-dichloro-9*H*-purin-9-yl)-4-(trifluoromethoxy)pyridin-3-yl)acetamide (2u-II).......... 49

Ethyl (*E*)-3-(4-fluoro-3-(2-methoxy-5-((methoxycarbonyl)amino)-6-(trifluoromethoxy)pyridin-3-yl)phenyl)acrylate (2v) 50

Methyl 4-((4-(2-methoxy-5-((methoxycarbonyl)amino)-6-(trifluoromethoxy)pyridin-3-yl)phenyl)ethynyl)benzoate (2w) 51

N-(6-(((8*R*,9*S*,13*S*,14*S*)-13-Methyl-17-oxo-7,8,9,11,12,13,14,15,16,17-decahydro-6*H*-cyclopenta[*a*]phenanthren-3-yl)oxy)-2-(trifluoromethoxy)pyridin-3-yl)acetamide (2x) 52

N-(6-((6*R*,12a*R*)-6-(Benzo[*d*][1,3]dioxol-5-yl)-2-methyl-1,4-dioxo-1,3,4,6,12,12a-hexahydropyrazino[1',2':1,6]pyrido[3,4-b]indol-7(2*H*)-yl)-2-(trifluoromethoxy)pyridin-3-

yl)acetamide (2y) 52
N-(2-(2-(Thiazol-4-yl)-1*H*-benzo[*d*]imidazol-1-yl)-4-(trifluoromethoxy)pyrimidin-5-yl)acetamide (4a) 53
N-(2-(5-Chloro-1*H*-indol-1-yl)-4-(trifluoromethoxy)pyrimidin-5-yl)acetamide (4b) 54
N-(2-Methoxy-4-(trifluoromethoxy)pyrimidin-5-yl)acetamide (4c) 54
N-(2-(4-Chloro-3,5-dimethylphenoxy)-4-(trifluoromethoxy)pyrimidin-5-yl)acetamide (4d)55
N-(2-(((8*S*,9*R*,13*R*,14*R*)-13-Methyl-17-oxo-7,8,9,11,12,13,14,15,16,17-decahydro-6*H*-cyclopenta[*a*]phenanthren-3-yl)oxy)-4-(trifluoromethoxy)pyrimidin-5-yl)acetamide (4e) .. 55
Methyl (6-bromopyridin-3-yl)(trifluoromethoxy)carbamate (1d’) 56
5-Bromo-6-methoxy-2-(trifluoromethoxy)pyridin-3-amine (2a’) 57
N-(5-Bromo-6-methoxy-2-(trifluoromethoxy)pyridin-3-yl)-2-(thiophen-2-yl)acetamide (5a) 57
tert-Butyl 4-(3-(2-methoxy-5-((methoxycarbonyl)amino)-6-(trifluoromethoxy)pyridin-3-yl)benzoyl)piperazine-1-carboxylate (6a) 58
Methyl 2-((5-bromo-6-methoxy-2-(trifluoromethoxy)pyridin-3-yl)amino)benzoate (5a) 59
Methyl (6-methoxy-5-((4-methoxyphenyl)ethynyl)-2-(trifluoromethoxy)pyridin-3-yl)carbamate (8a) 59
Procedure for the large scale trifluoromethoxylation 60
Methyl (5-bromo-6-methoxy-2-(trifluoromethoxy)pyridin-3-yl)carbamate (2a) 60
O-Trifluoromethylation in the presence of a radical trap 60
O-CF_3 Migration in the presence of a radical trap 61
Spectroscopic Data 62

Materials and Methods

All air- and moisture-insensitive reactions were carried out under an ambient atmosphere, magnetically stirred, and monitored by thin layer chromatography (TLC) using Agela Technologies TLC plates pre-coated with 250 μm thickness silica gel 60 F254 plates and visualized by fluorescence quenching under UV light. Flash chromatography was performed on SiliaFlash® Silica Gel 40-63μm 60Å particle size using a forced flow of eluent at 0.3–0.5 bar pressure.[1] All air- and moisture-sensitive manipulations were performed using oven-dried glassware, including standard Schlenk and glovebox techniques under an atmosphere of nitrogen. Diethyl ether and THF were distilled from deep purple sodium benzophenone ketyl. Methylene chloride, chloroform and acetonitrile were dried over CaH_2 and distilled. Nitromethane was dried over calcium sulphate and distilled. Methylene chloride and nitromethane were degassed *via* three freeze-pump-thaw cycles. All other chemicals were used as received. All deuterated solvents were purchased from Cambridge Isotope Laboratories. NMR spectra were recorded on either a Bruker Ascend 700 spectrometer operating at 700 MHz for ^1H acquisitions and 175 MHz for ^{13}C acquisitions, a Bruker 500 Advance spectrometer operating at 500 MHz, 125 MHz, and 470 MHz for ^1H, ^{13}C, and ^{19}F acquisitions, respectively, a Bruker 400 Nanobay spectrometer operating at 400 MHz, 100 MHz, and 376 MHz for ^1H, ^{13}C, and ^{19}F acquisitions, respectively. Chemical shifts were referenced to the residual proton solvent peaks (^1H: $CDCl_3$, δ 7.26; $(CD_3)_2SO$, δ 2.50; CD_3OD, δ 3.31; CD_3CN, δ 1.94), solvent ^{13}C signals ($CDCl_3$, δ 77.16; $(CD_3)_2SO$, δ 39.52; CD_3OD, δ 49.00),[2] dissolved or external neat $PhCF_3$ (^{19}F, δ –63.3 relative to $CFCl_3$).[3] Signals are listed in ppm, and multiplicity identified as s = singlet, br = broad, d = doublet, t = triplet, q = quartet, m = multiplet; coupling constants in Hz; integration. High-resolution mass spectra were performed at Mass Spectrometry Services at the Univ. of Illinois at Urbana-Champaign and were obtained using Waters Q-TOF Ultima ESI mass spectrometer. Concentration under reduced pressure was performed by rotary evaporation at 25–30 ºC at appropriate pressure. Purified compounds were further dried under high vacuum (0.01–0.05 Torr). Yields refer to purified and spectroscopically pure compounds.

---

[1] Still, W. C.; Kahn, M.; Mitra, A. *J. Org. Chem.* **1978**, *43*, 2925–2927.

[2] Fulmer, G. R.; Miller, A. J. M.; Sherden, N. H.; Gottlieb, H. E.; Nudelman, A.; Stoltz, B. M.; Bercaw, J. E.; Goldberg, K. I. *Organometallics*. **2010**, *29*, 2176–2179.

[3] Wang, X.; Xu, Y.; Mo, F.; Ji, G.; Qiu, D.; Feng, J.; Ye, Y.; Zhang, S.; Zhang, Y.; Wang, J. *J. Am. Chem. Soc*. **201**3, *135*, 10330–10333.

Experimental Data

Optimization of the one-pot synthesis of protected *N*-heteroaryl-*N*-hydroxylamines

Table S1. Optimization of the one-pot synthesis of *N*-(6-(5-bromo-1*H*-pyrrolo[2,3-b]pyridin-1-yl)pyridin-3-yl)-*N*-hydroxyacetamide (1s)

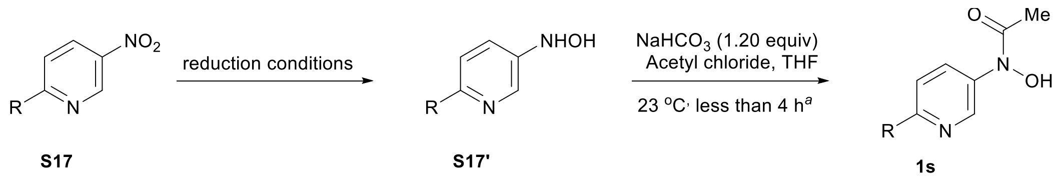

R = 5-bromo-1*H*-pyrrolo[2,3-*b*]pyridine

Entry	Reaction conditions	Reaction time	Observations	Formation of S_{17}'	Yield of **1s**
**1**	H_2NNH_2•H_2O (1.20 eq.) 5% Rh/C,THF, rt	2 h	A lot of yellow solid precipitated out after 20 min	/	0%
**2**	NaH_2PO_2(2.5 eq.), 5% Pd/C,THF/H_2O, 45°C	2 h	Colourless suspension	/	0%[b]
**3**	Zn(4.0 eq), NH_4Cl (1.0 eq) EtOH/H_2O, rt	2 h	Gray suspension	Yes	0%[c]
**4**	$HCOONH_4$ (2.0 eq.), 5% Pd/C, EtOH, 50°C	2 h	Colourless suspension	/	0%[c]
**5**	$HCOONH_4$ (2.0 eq.), 5% Pd/C, THF, 50°C	2 h	Colourless suspension	Yes	20%[d]
**6**	SmI_2 (4.0 eq) THF/MeOH, rt	2 h	Some yellow solid precipitated out immediately	/	0%
**7**	SmI_2 (4.0 eq) THF/MeOH, rt	10 min	Some yellow solid precipitated out immediately	Yes	10%[e]
**8**	H_2NNH_2•H_2O (1.20 eq.) 5% Rh/C,THF, rt	20 min	Very little yellow solid precipitated out	Yes	86%

[a]Reaction time for the second step has to be carefully controlled. [b]A large amount of *N*-protected aminopyridine was formed. [c]Mostly starting material and a small amount of *N*-protected aminopyridine was formed. [d]Starting material, *N*-protected aminopyridine, and desired product were formed. [e]Starting material, insoluble yellow solid, and desired product were formed.

The presence of heteroarenes complicated the synthesis of the protected *N*-heteroaryl-*N*-hydroxylamine precursors. As shown in Table S1, no desired product was obtained under the standard conditions used for the reduction of nitroarenes (entry 1). Upon examination of different reduction conditions, we found that compound **S17** was easily overreduced to aminopyridine (entry 2) or gave a mixture of starting material, overreduced aminopyridine with or without desired *N*-pyridinyl-*N*-hydroxylamine (entries 3-7). After closely monitoring the reaction, we obtained the desired product **1s** in 86% yield using hydrazine as a reductant and Rh/C as a catalyst (entry 8). We observed that protection of *N*-pyridinyl-*N*-hydroxylamine was also very time sensitive. Reactions usually go to completion in less than 15 min. Extending the reaction time longer than 4h leads to the formation of side-products (*O*-acetyl-*N*-pyridinylhydroxylamine and *N*-acetoxy-*N*-pyridinylacetamide). It is worth to mention that the optimized conditions are applicable to most of the substrates. However, more optimization experiments were needed for some of the substrates. For example, reduction of 2-fluoro-5-nitropyridine requires high reaction temperature (40 °C) and

hydrazine has to be added in one portion; otherwise a side product of 2-hydrazinyl-5-nitropyridine is formed. The key parameters for obtaining the desired products in high yields are: solvent, reaction temperature, reaction time and the rate of hydrazine addition (see reaction procedures for further details).

Methyl (5-bromo-6-methoxypyridin-3-yl)(hydroxy)carbamate (1a)

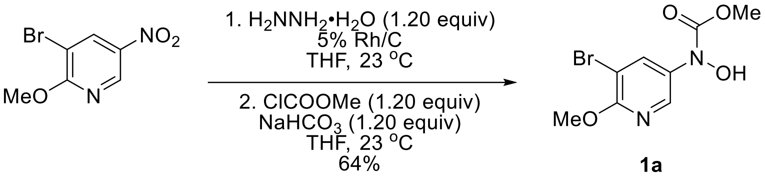

1a

Under N_2 atmosphere, to a suspension of 3-bromo-2-methoxy-5-nitropyridine (5.00 g, 21.5 mmol, 1.00 equiv) and 5% Rh/C (0.123 g, 0.30 mol% Rh) in THF (107 mL, 0.200 M) hydrazine monohydrate (1.29 g, 25.8 mmol, 1.20 equiv) was added dropwise. The reaction mixture was monitored *via* TLC using EtOAc:hexanes 1:1 (v/v) as an eluent until the disappearance of the starting 3-bromo-2-methoxy-5-nitropyridine (R_f = 0.90 (EtOAc:hexanes 1:1 (v/v)) and the appearance of the hydroxylamine intermediate (R_f = 0.61 (EtOAc:hexanes 1:1 (v/v)). Subsequently, sodium bicarbonate (2.14 g, 25.8 mmol, 1.20 equiv) was added to the reaction mixture followed by a solution of methyl chloroformate (2.42 g, 25.75 mmol, 1.20 equiv) in THF (6.58 mL, 0.200 M) via a syringe pump (at a rate of 10.0 mL/h). After the addition was complete, the reaction mixture was filtered through a short pad of celite and the celite was washed with EtOAc. The organic layers were combined and concentrated in vacuo. The residue was purified by chromatography on silica gel, eluting with EtOAc:hexanes (3:7 to 1:1 (v/v)), to afford the title compound as a slightly light yellow solid (3.82 g, 13.8 mmol, 64% yield).

R_f = 0.54 (EtOAc:hexanes 1:1 (v/v)). NMR Spectroscopy: ^{1}H NMR (500 MHz, $(CD_3)_2SO$, 25 ºC, δ): 10.60 (s, 1H) 8.29 (d, J = 2.44 Hz, 1H) 8.13 (d, J = 2.44 Hz, 1H) 3.93 (s, 3H) 3.74 (s, 3H). ^{13}C NMR (125 MHz, $(CD_3)_2SO$, 25 ºC, δ): 157.0, 155.4, 138.9, 135.7, 134.5, 105.4, 55.0, 53.7. Mass Spectrometry: HRMS (ESI-TOF) (m/z): calcd for $C_8H_{10}BrN_2O_4$ ([M + H]$^+$), 276.9818, found, 276.9821.

***N*-(5-Iodo-6-methoxypyridin-3-yl)hydroxylamine (S1)**

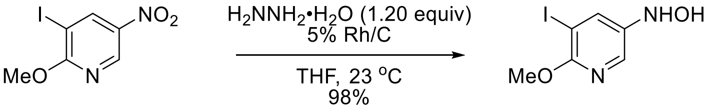

S1

Under N_2 atmosphere, to a suspension of 3-iodo-2-methoxy-5-nitropyridine (0.500 g, 1.79 mmol, 1.00 equiv) and 5% Rh/C (10.3 mg, 0.30 mol% Rh) in THF (8.93 mL, 0.200 M) hydrazine monohydrate (0.11 g, 2.14 mmol, 1.20 equiv) was added dropwise. After the reaction mixture was stirred at 23 °C for 1 h, it was filtered through a short pad of celite and concentrated *in vacuo* to

afford the title compound as a slightly brown solid (0.467 g, 1.76 mmol, 98% yield). The product was used directly without further purification.

R_f = 0.35 (EtOAc:hexanes 1:1 (v/v)). NMR Spectroscopy: ^{1}H NMR (500 MHz, $(CD_3)_2SO$, 25 ºC, δ): 8.51 (br. s, 1H) 8.21 (s, 1H) 7.75 (d, J = 2.44 Hz, 1H) 7.69 (d, *J* = 2.44 Hz, 1H) 3.81 (s, 3H). ^{13}C NMR (175 MHz, $(CD_3)_2SO$, 25 ºC, δ): 155.9, 143.7, 134.6, 131.1, 79.5, 54.3. Mass Spectrometry: HRMS (ESI-TOF) (m/z): calcd for $C_6H_8IN_2O_2$ ($[M + H]^+$), 266.9625, found, 266.9624.

Methyl hydroxy(5-iodo-6-methoxypyridin-3-yl)carbamate (1b)

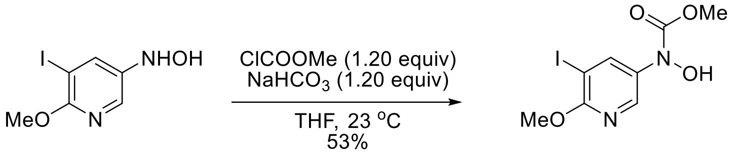

S1 **1b**

Under N_2 atmosphere, to a stirred suspension of *N*-(5-iodo-6-methoxypyridin-3-yl)hydroxylamine **(S1)** (0.350 g, 1.32 mmol, 1.00 equiv) and $NaHCO_3$ (0.131 g, 1.58 mmol, 1.20 equiv) in THF (6.58 mL, 0.200 M) at 23 °C was slowly added a solution of methyl chloroformate (0.148 g, 1.58 mmol, 1.20 equiv) in THF (6.58 mL, 0.200 M) via a syringe pump (at a rate of 10.0 mL/h). After the addition was complete, the reaction mixture was filtered through a short pad of celite and the celite was washed with EtOAc. The organic layers were combined and concentrated in vacuo. The residue was purified by chromatography on silica gel, eluting with EtOAc:hexanes (1:1 to 1:0 (v/v)), to afford the title compound as a slightly light brown solid (0.229 g, 0.710 mmol, 54% yield).

R_f = 0.63 (EtOAc:hexanes 1:0 (v/v)). NMR Spectroscopy: ^{1}H NMR (500 MHz, $(CD_3)_2SO$, 25 ºC, δ): 10.54 (br. s, 1H) 8.26 (br. s, 2H) 3.89 (br. s, 3H) 3.73 (br. s, 3H). ^{13}C NMR (125 MHz, $(CD_3)_2SO$, 25 ºC, δ): 158.8, 154.9, 141.5, 139.4, 134.0, 79.1, 54.8, 53.1. HRMS (ESI-TOF) (m/z): calcd for $C_8H_{10}IN_2O_4$ ($[M + H]^+$), 324.9680, found, 324.9682.

***N*-(6-Chloro-4-methylpyridin-3-yl)hydroxylamine (S2)**

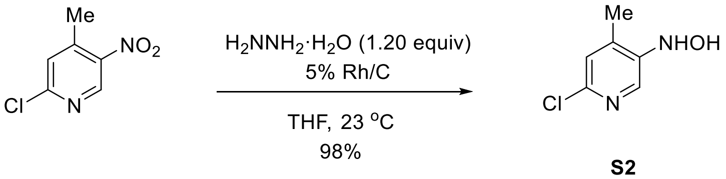

S2

Under N_2 atmosphere, hydrazine monohydrate (1.04 g, 20.9 mmol, 1.20 equiv) was added dropwise to a suspension of 2-chloro-4-methyl-5-nitropyridine (3.00 g, 17.4 mmol, 1.00 equiv) and 5% Rh/C (0.300 g, 0.838 mol% Rh) in THF (85.0 mL, 0.204 M) at 23 °C. The reaction mixture was stirred at 23 °C for 3 h and filtered through a short pad of celite. The celite was washed with EtOAc. The organic solutions were combined and concentrated *in vacuo* to afford the title compound as a white solid (2.70 g, 17.1 mmol, 98% yield). The product was used directly without further purification.

R$_f$ = 0.28 (hexanes/EtOAc 3:2 (v/v)). NMR Spectroscopy: ^{1}H NMR (500 MHz, $(CD_3)_2SO$, 25 ºC, δ): 8.52 (s, 1H), 8.34 (s, 1H), 8.01 (s, 1H), 7.13 (s, 1H), 2.08 (s, 3H). ^{13}C NMR (125 MHz, $(CD_3)_2SO$, 25 ºC, δ): 145.3, 140.2, 135.1, 133.0, 123.8, 16.1. Mass Spectrometry: HRMS (ESI-TOF) (m/z): calcd for $C_6H_8ClN_2O$ ([M + H]$^+$), 159.0320, found, 159.0319.

Methyl (6-chloro-4-methylpyridin-3-yl)(hydroxy)carbamate (1c)

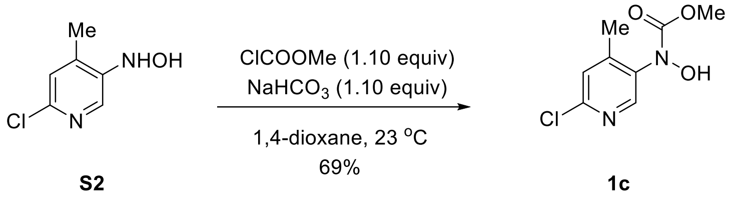

Under N_2 atmosphere, a solution of methyl chloroformate (0.623 g, 6.59 mmol, 1.10 equiv) in 1,4-dioxane (10.0 mL, 0.660 M) was slowly added to a stirred suspension of *N*-(6-chloro-4-methylpyridin-3-yl)hydroxylamine (**S2**) (0.950 g, 5.99 mmol, 1.00 equiv) and $NaHCO_3$ (0.554 g, 6.59 mmol, 1.10 equiv) in 1,4-dioxane (40.0 mL, 0.150 M) at 23 °C. After the reaction was complete (4 h), the reaction mixture was concentrated *in vacuo*. The residue was purified by chromatography on silica gel, eluting with hexanes:EtOAc (4:1 to 3:2 (v/v)), to afford the title compound as a red solid (0.890 g, 4.11 mmol, 69% yield).

R$_f$ = 0.27 (hexanes/EtOAc 3:2 (v/v)). NMR Spectroscopy: ^{1}H NMR (700 MHz, $(CD_3)_2SO$, 25 ºC, δ): 10.53 (s, 1H), 8.29 (s, 1H), 7.52 (s, 1H), 3.68 (s, 3H), 2.23 (s, 3H). ^{13}C NMR (175 MHz, $(CD_3)_2SO$, 25 ºC, δ): 155.6, 148.8, 148.4, 147.9, 137.7, 125.6, 53.2, 16.8. Mass Spectrometry: HRMS (ESI-TOF) (m/z): calcd for $C_8H_{10}ClN_2O_3$ ([M + H]$^+$), 217.0374, found, 217.0373.

***N*-(6-Bromopyridin-3-yl)hydroxylamine (S3)**

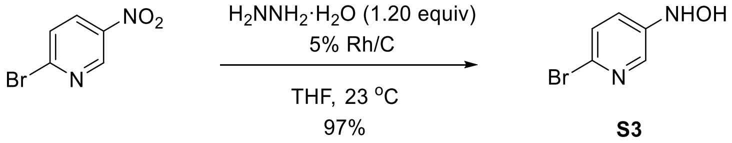

Under N_2 atmosphere, hydrazine monohydrate (0.888 g, 17.7 mmol, 1.20 equiv) was added dropwise to a suspension of 2-bromo-5-nitropyridine (3.00 g, 14.8 mmol, 1.00 equiv) and 5% Rh/C (0.290 g, 0.952 mol% Rh) in THF (75.0 mL, 0.197 M) at 23 °C. After the reaction was complete (2.5 h), the reaction mixture was filtered through a short pad of celite and the celite was washed with EtOAc. The organic solutions were combined and concentrated *in vacuo* to afford the title compound as a yellow solid (2.70 g, 14.3 mmol, 97% yield). The product was used directly without further purification.

R$_f$ = 0.31 (hexanes/EtOAc 3:2 (v/v)). NMR Spectroscopy: ^{1}H NMR (500 MHz, $(CD_3)_2SO$, 25 ºC, δ): 8.67 (s, 1H), 8.65 (s, 1H), 7.91 (d, J = 2.8 Hz, 1H), 7.38 (d, J = 8.6 Hz, 1H), 7.14 (dd, J = 8.6, 2.8 Hz, 1H). ^{13}C NMR (125 MHz, $(CD_3)_2SO$, 25 ºC, δ): 147.8, 135.1, 129.6, 127.3, 123.4. Mass Spectrometry: HRMS (ESI-TOF) (m/z): calcd for $C_5H_6BrN_2O$ ([M + H]$^+$), 188.9658, found,

188.9657.

Methyl (6-bromopyridin-3-yl)(hydroxy)carbamate (1d)

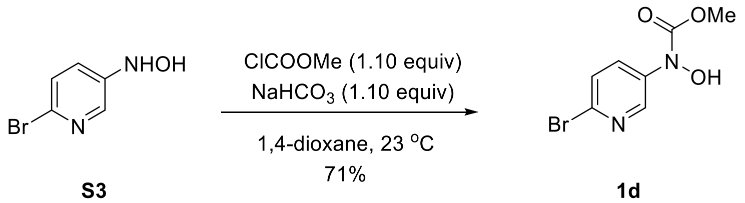

Under N_2 atmosphere, a solution of methyl chloroformate (0.520 g, 5.50 mmol, 1.10 equiv) in 1,4-dioxane (10.0 mL, 0.550 M) was added dropwise to a stirred suspension of *N*-(6-bromopyridin-3-yl)hydroxylamine (**S3**) (0.940 g, 5.00 mmol, 1.00 equiv) and $NaHCO_3$ (0.462 g, 5.50 mmol, 1.10 equiv) in 1,4-dioxane (40.0 mL, 0.125 M) at 23 °C. After the reaction was complete (3 h), the reaction mixture was filtered through a short pad of celite and the celite was washed with EtOAc. The combined organic layers were concentrated *in vacuo*. The residue was purified by chromatography on silica gel, eluting with hexanes:EtOAc (4:1 to 3:2 (v/v)), to afford the title compound as a yellow solid (0.870 g, 3.52 mmol, 71% yield).

R_f = 0.27 (hexanes/EtOAc 3:2 (v/v)). NMR Spectroscopy: ^{1}H NMR (500 MHz, $(CD_3)_2SO$, 25 °C, δ): 10.74 (s, 1H), 8.58 (d, J = 2.7 Hz, 1H), 7.89 (dd, J = 2.7, 3.8 Hz, 1H), 7.64 (d, J = 3.8 Hz, 1H), 3.78 (s, 3H). ^{13}C NMR (125 MHz, $(CD_3)_2SO$, 25 °C, δ): 154.4, 140.9, 138.8, 134.9, 129.6, 127.5, 53.4. Mass Spectrometry: HRMS (ESI-TOF) (m/z): calcd for $C_7H_8BrN_2O_3$ ($[M + H]^+$), 246.9713, found, 246.9712.

Methyl (6-fluoropyridin-3-yl)(hydroxy)carbamate (1e)

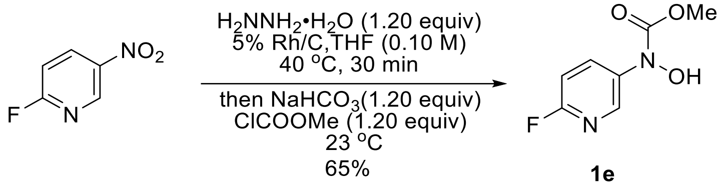

Under N_2 atmosphere, a suspension of 2-fluoro-5-nitropyridine (1.00 g, 7.04 mmol, 1.00 equiv) and 5% Rh/C (80.8 mg, 0.60 mol% Rh) in THF (35.2 mL, 0.200 M) was heated to 40 °C. Hydrazine monohydrate (0.422 g, 8.44 mmol, 1.20 equiv) was added all at once. The reaction mixture was stirred at 40 °C for 30 min and then cooled down to 23 °C. $NaHCO_3$ (0.708 g, 8.44 mmol, 1.2 equiv) was added, followed by dropwise addition of methyl chloroformate (0.800 g, 8.44 mmol, 1.20 equiv) in THF (35.2 mL, 0.240 M) at 23 °C. After the reaction was complete (1 h), the reaction mixture was filtered through a short pad of celite, washed with EtOAc, and concentrated *in vacuo*. The residue was purified by chromatography on silica gel, eluting with hexanes:EtOAc (4:1 to 2:1 (v/v)), to afford the title compound as a red solid (0.850 g, 4.57 mmol, 65% yield).

R_f = 0.22 (hexanes:EtOAc 2:1 (v/v)). NMR Spectroscopy: ^{1}H NMR (700 MHz, $(CD_3)_2SO$, 25 °C, δ): 10.67 (s, 1H) 8.36 (d, J = 1.29 Hz, 1H) 8.09–8.06 (m, 1H) 7.21 (dd, J = 8.82, 3.23 Hz,

1H) 3.76 (s, 3H). ^{13}C NMR (175 MHz, $(CD_3)_2SO$, 25 ºC, δ): 159.3 (d, *J* = 232.8 Hz), 154.8, 138.8 (d, *J* = 15.8 Hz), 137.3 (d, *J* = 5.25 Hz), 133.8 (d, *J* = 8.75 Hz), 109.2 (d, *J* = 40.3 Hz), 53.3. ^{19}F NMR (376 MHz, $CDCl_3$, 25 ºC, δ): –74.7 (s). Mass Spectrometry: HRMS (ESI-TOF) (m/z): calcd for $C_7H_8FN_2O_3$ ([M + H]$^+$), 187.0513, found, 187.0513.

_N_-(5-Bromo-6-chloropyridin-3-yl)hydroxylamine (S4)

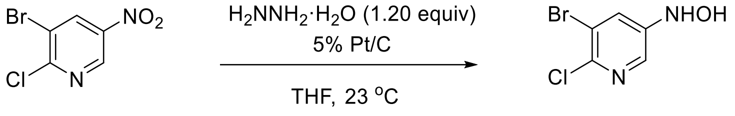

Under N_2 atmosphere, hydrazine monohydrate (0.506 g, 10.1 mmol, 1.20 equiv) was added dropwise to a suspension of 3-bromo-2-chloro-5-nitropyridine (2.00 g, 8.42 mmol, 1.00 equiv) and Pt/C (0.200 g, 0.609 mol% Pt) in THF (75.0 mL, 0.112 M) at 23 °C. The reaction mixture was stirred at 23 °C for 3 h, filtered through a short pad of celite, washed with EtOAc, and concentrated *in vacuo*. The residue was dissolved in ether and filtered off. The filtrate was kept at -20 ºC overnight and filtered again to remove the solid. The filtrate was concentrated *in vacuo* to afford the title compound as a yellow solid (1.22 g, 5.46 mmol, 65% yield). The product was used directly without further purification.

R*f* = 0.31 (hexanes/EtOAc 3:2 (v/v)). NMR Spectroscopy: ^{1}H NMR (500 MHz, $(CD_3)_2SO$, 25 ºC, δ): 8.90 (s, 1H), 8.86 (s, 1H), 7.92 (d, *J* = 2.5 Hz, 1H), 7.53 (d, *J* = 2.5 Hz, 1H). ^{13}C NMR (125 MHz, $(CD_3)_2SO$, 25 ºC, δ): 148.5, 137.9, 133.2, 125.2, 118.8. Mass Spectrometry: HRMS (ESI-TOF) (m/z): calcd for $C_5H_5BrClN_2O$ ([M + H]$^+$), 224.9246, found, 224.9245.

Methyl (5-bromo-6-chloropyridin-3-yl)(hydroxy)carbamate (1f)

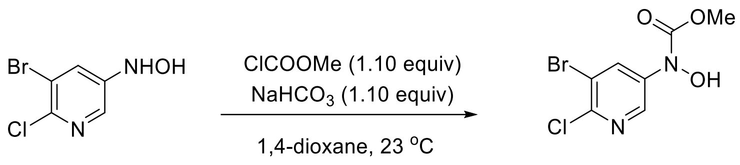

Under N_2 atmosphere, a solution of methyl chloroformate (0.256 g, 2.71 mmol, 1.10 equiv) in 1,4-dioxane (10.0 mL, 0.271 M) was added dropwise to a stirred suspension of *N*-(5-bromo-6-chloropyridin-3-yl)hydroxylamine (**S4**) (0.550 g, 2.46 mmol, 1.00 equiv) and $NaHCO_3$ (0.227 g, 2.71 mmol, 1.10 equiv) in 1,4-dioxane (15.0 mL, 0.164 M) at 23 °C. The reaction mixture was stirred for 6 h and concentrated *in vacuo*. The residue was purified by chromatography on silica gel, eluting with hexanes:EtOAc (4:1 to 3:2 (v/v)), to afford the title compound as a yellow solid (0.55 g, 1.95 mmol, 80% yield).

R*f* = 0.42 (hexanes/EtOAc 3:2 (v/v)). NMR Spectroscopy: ^{1}H NMR (500 MHz, $(CD_3)_2SO$, 25 ºC, δ): 10.89 (s, 1H), 8.63 (d, *J* = 2.3 Hz, 1H), 8.33 (d, *J* = 2.3 Hz, 1H), 3.80 (s, 3H). ^{13}C NMR (125 MHz, $(CD_3)_2SO$, 25 ºC, δ): 154.3, 143.0, 138.9, 138.4, 131.7, 118.5, 53.6. Mass Spectrometry:

HRMS (ESI-TOF) (m/z): calcd for $C_7H_7BrClN_2O_3$ ([M + H]$^+$), 282.9301, found, 282.9302.

_N_-(2-Chloropyridin-3-yl)hydroxylamine (S5)

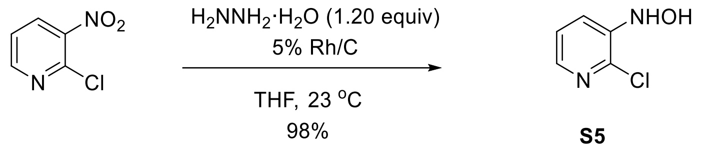

Under N_2 atmosphere, hydrazine monohydrate (1.89 g, 37.8 mmol, 1.20 equiv) was added dropwise to a suspension of 2-chloro-3-nitropyridine (5.00 g, 31.5 mmol, 1.00 equiv) and Rh/C (0.500 g, 0.771 mol% Rh) in THF (150 mL, 0.210 M) at 23 °C. The reaction mixture was stirred at 23 °C for 3 h, filtered through a short pad of celite, washed with EtOAc, and concentrated *in vacuo* to afford of the title compound as a red solid (4.50 g, 31.1 mmol, 98% yield). The product was used directly without further purification.

R_f = 0.28 (hexanes/EtOAc 3:2 (v/v)). NMR Spectroscopy: ^{1}H NMR (500 MHz, $(CD_3)_2SO$, 25 ºC, δ): 8.74 (s, 1H), 8.51 (s, 1H), 7.80–7.76 (m, 1H), 7.49–7.46 (m, 1H), 7.31–7.28 (m, 1H). ^{13}C NMR (125 MHz, $(CD_3)_2SO$, 25 ºC, δ): 144.5, 138.9, 134.5, 123.7, 121.2. Mass Spectrometry: HRMS (ESI-TOF) (m/z): calcd for $C_5H_6ClN_2O$ ([M + H]$^+$), 145.0163, found, 145.0161.

Methyl (2-chloropyridin-3-yl)(hydroxy)carbamate (1g)

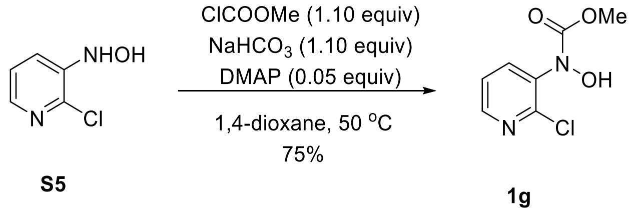

Under N_2 atmosphere, a solution of methyl chloroformate (0.140 g, 1.53 mmol, 1.10 equiv) in dioxane (7.00 mL, 0.220 M) was slowly added via a syringe pump (at a rate of 10.0 mL/h) to a stirred suspension of *N*-(2-chloropyridin-3-yl)hydroxylamine (**S5**) (0.200 g, 1.39 mmol, 1.00 equiv), $NaHCO_3$ (0.130 g, 1.53 mmol, 1.10 equiv) and DMAP (8.50 mg, 0.0700 mmol, 0.0500 equiv) in dioxane (7.00 mL, 0.200 M) at 50 °C. After the addition was complete, the reaction mixture was stirred at 50 °C for another 12 h and then filtered through a short pad of celite and the celite was washed with EtOAc. The organic layers were combined and concentrated *in vacuo*. The residue was purified by chromatography on silica gel, eluting with hexanes:EtOAc (5:1 to 1:1 (v/v)), to afford the title compound as a yellow gum (0.210 g, 1.04 mmol, 75% yield).

R_f = 0.19 (hexanes/EtOAc 5:1 (v/v)). NMR Spectroscopy: ^{1}H NMR (700 MHz, $(CD_3)_2SO$, 25 ºC, δ): 10.63 (s, 1H), 8.39 (dd, *J* = 4.73, 1.72 Hz, 1H), 7.96 (dd, *J* = 7.74, 1.72 Hz, 1H), 7.52 (dd, *J* = 7.74, 4.73 Hz, 1H), 3.69 (s, 3 H). ^{13}C NMR (175 MHz, $(CD_3)_2SO$, 25 ºC, δ): 155.3, 148.9, 148.1, 137.9, 136.6, 124.0, 53.2. Mass Spectrometry: HRMS (ESI-TOF) (m/z): calcd for $C_7H_7ClN_2O_3Na$ ([M + Na]$^+$), 227.0010, found, 227.0019.

N-(2,6-Dichloropyridin-3-yl)-*N*-hydroxyacetamide (1h)

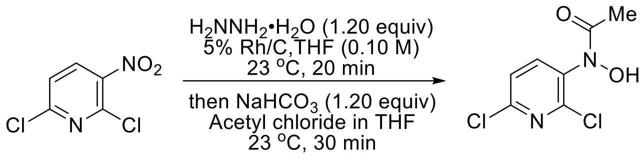

65%

1h

Under N_2 atmosphere, a suspension of 2-fluoro-5-nitropyridine (1.00 g, 7.04 mmol, 1.00 equiv) and 5% Rh/C (80.8 mg, 0.60 mol% Rh) in THF (35.2 mL, 0.200 M) was heated to 40 °C. Hydrazine monohydrate (0.422 g, 8.44 mmol, 1.20 equiv) was added all at once. The reaction mixture was stirred at 40 °C for 30 min and then cooled down to 23 °C. $NaHCO_3$ (0.708 g, 8.44 mmol, 1.2 equiv) was added, followed by dropwise addition of methyl chloroformate (0.800 g, 8.44 mmol, 1.20 equiv) in THF (35.2 mL, 0.240 M) at 23 ºC. The reaction mixture was stirred at 23 °C for 1 h and then filtered through a short pad of celite, washed with EtOAc, and concentrated *in vacuo*. The residue was purified by chromatography on silica gel, eluting with hexanes:EtOAc (4:1 to 2:1 (v/v)), to afford the title compound as a red solid (0.850 g, 4.57 mmol, 65% yield).

R_f = 0.22 (hexanes:EtOAc 2:1 (v/v)). NMR Spectroscopy: ^{1}H NMR (700 MHz, $(CD_3)_2SO$, 25 ºC, δ): 10.95 (br. s., 1H), 8.01 (d, *J* = 7.74 Hz, 1H), 7.67 (d, *J* = 8.17 Hz, 1H), 2.19 (br. s., 3H). ^{13}C NMR (175 MHz, $(CD_3)_2SO$, 25 ºC, δ): 171.0, 147.5, 146.9, 140.9, 135.9, 124.6, 20.8. Mass Spectrometry: HRMS (ESI-TOF) (m/z): calcd for $C_7H_7Cl_2N_2O_2$ ($[M + H]^+$), 220.9879, found, 220.9878.

N-Hydroxy-*N*-(6-methoxy-4-methylpyridin-3-yl)acetamide (1i)

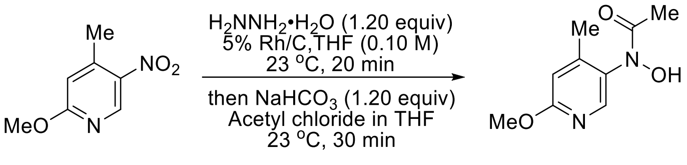

79%

1i

Under N_2 atmosphere, a suspension of 2-methoxy-4-methyl-5-nitropyridine (491 mg, 2.92 mmol, 1.00 equiv) and 5% Rh/C (33.6 mg, 0.60 mol% Rh) in dioxane (14.6 mL, 0.200 M) was stirred at 40 °C. Hydrazine monohydrate (146 mg, 3.50 mmol, 1.20 equiv) in dioxane (14.6 mL, 0.240 M) was added via a syringe pump (at a rate of 15.0 mL/h). The reaction mixture was stirred at 40 °C for 1 h and then cooled down to 23 °C. $NaHCO_3$ (290 mg, 3.50 mmol, 1.2 equiv) was added, followed by dropwise addition of acetyl chloride (270 mg, 3.50 mmol, 1.20 equiv) in THF (29.2 mL, 0.120 M) at 23 ºC. The reaction mixture was stirred at 23 °C for 1 h and then filtered through a short pad of celite, washed with EtOAc, and concentrated *in vacuo*. The residue was purified by chromatography on silica gel, eluting with hexanes:EtOAc (4:1 to 1:1 (v/v)), to afford the title compound as a brown solid (450 mg, 2.29 mmol, 79% yield).

R_f = 0.08 (hexanes:EtOAc 4:1 (v/v)). NMR Spectroscopy: ^{1}H NMR (700 MHz, $(CD_3)_2SO$, 90 ºC, δ): 10.21 (br. s., 1H), 8.01 (s, 1H), 6.73 (s, 1H), 3.86 (s, 3H), 2.18 (s, 3H), 2.08 (s, 3H). ^{13}C

NMR (175 MHz, $(CD_3)_2SO$, 25 °C, δ): 170.9, 162.9, 148.1, 145.7, 132.4, 110.9, 53.4, 20.8, 17.1. Mass Spectrometry: HRMS (ESI-TOF) (m/z): calcd for $C_9H_{12}N_2O_3K$ ($[M + K]^+$), 236.0509, found, 236.0499.

N-(6-Methylpyridin-3-yl)hydroxylamine (S6)

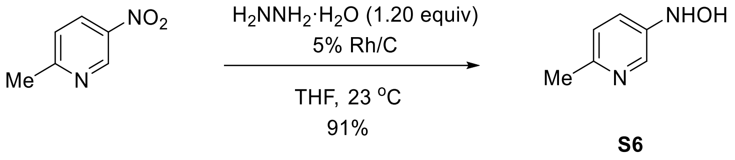

Under N_2 atmosphere, hydrazine monohydrate (0.870 g, 17.4 mmol, 1.20 equiv) was added dropwise to a suspension of 2-methyl-5-nitropyridine (2.00 g, 14.5 mmol, 1.00 equiv) and Rh/C (0.120 g, 0.402 mol% Rh) in THF (75.0 mL, 0.193 M) at 23 °C. The reaction mixture was stirred at 23 °C for 2 h, filtered through a short pad of celite, washed with EtOAc, and concentrated *in vacuo* to afford the title compound as a yellow solid (1.63 g, 13.1 mmol, 91% yield). The product was used directly without further purification.

R_f = 0.20 (EtOAc). NMR Spectroscopy: ^1H NMR (700 MHz, $(CD_3)_2SO$, 25 °C, δ): 8.40 (s, 1H), 8.31 (s, 1H), 8.02 (d, J = 2.4 Hz, 1H), 7.10 (dd, J = 8.3, 2.4 Hz, 1H), 7.03 (d, J = 8.3 Hz, 1H). ^{13}C NMR (175 MHz, $(CD_3)_2SO$, 25 °C, δ): 148.4, 145.5, 134.8, 122.4, 120.8, 23.0. Mass Spectrometry: HRMS (ESI-TOF) (m/z): calcd for $C_6H_9N_2O$ ($[M + H]^+$), 125.0709, found, 125.0708.

N-Hydroxy-*N*-(6-methylpyridin-3-yl)acetamide (1j)

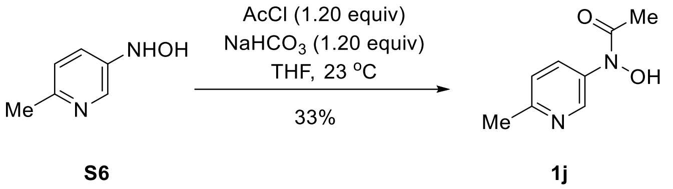

Under N_2 atmosphere, a solution of acetyl chloride (0.300 g, 3.87 mmol, 1.20 equiv) in THF (16.00 mL, 0.240 M) was added dropvise to a stirred suspension of *N*-(6-methylpyridin-3-yl)hydroxylamine (**S6**) (0.400 g, 3.22 mmol, 1.00 equiv) and $NaHCO_3$ (0.330 g, 3.87 mmol, 1.20 equiv) in THF (16.00 mL, 0.200 M) at 23 °C. The reaction mixture was stirred at 23 °C for another 30 min and then filtered through a short pad of celite and the celite was washed with EtOAc. The organic layers were combined and concentrated *in vacuo*. The residue was purified by chromatography on silica gel, eluting with EtOAc to afford the title compound as a light yellow solid (0.176 g, 1.06 mmol, 33% yield).

R_f = 0.17 (EtOAc). NMR Spectroscopy: ^1H NMR (700 MHz, $(CD_3)_2SO$, 25 °C, δ): 10.77 (br. s, 1H), 8.72 (br. s, 1H), 7.87 (dd, J = 8.17, 2.15 Hz, 1H), 7.25 (d, J = 8.60 Hz, 1H), 2.43 (s, 3H), 2.21 (br. s, 3H). ^{13}C NMR (175 MHz, $(CD_3)_2SO$, 25 °C, δ): 170.4, 153.6, 140.5, 136.0, 127.4, 122.6, 23.4, 22.1. Mass Spectrometry: HRMS (ESI-TOF) (m/z): calcd for $C_8H_{11}N_2O_2$ ($[M + H]^+$), 167.0815, found, 167.0816.

3-(2,4-Difluorophenyl)-2-methoxy-5-nitropyridine (S7)

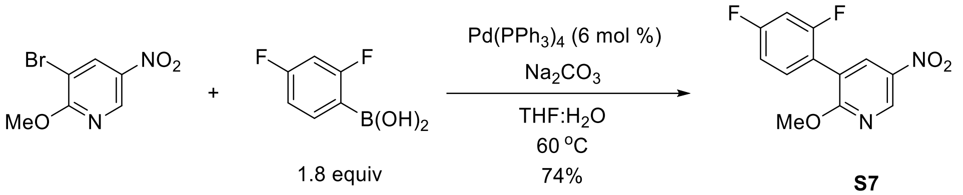

To a flask charged with 3-bromo-2-methoxy-5-nitropyridine (0.700 mg, 3.00 mmol, 1.00 equiv), (2,4-difluorophenyl)boronic acid (663 mg, 4.20 mmol, 1.40 equiv), $Pd(PPh_3)_4$ (104 mg, 0.0900 mmol, 3 mol%), and Na_2CO_3 (808 mg, 7.62 mmol, 2.54 equiv) were added THF (9.10 mL) and H_2O (7.62 mL). The mixture was degassed via three freeze-pump-thaw cycles and then heated at 60 °C under N_2 atmosphere for 3 h. After that time, $Pd(PPh_3)_4$ (104 mg, 0.0900 mmol, 3 mol%) and (2,4-difluorophenyl)boronic acid (189.5 mg, 1.20 mmol, 0.4 equiv) were added and the resulting mixture was stirred at 60 °C under N_2 atmosphere for further 15 h. The solution was cooled to rt. H_2O (30 mL) and CH_2Cl_2 (30 mL) were added and the layers were separated. Aqueous layer was further extracted with CH_2Cl_2 (2 × 30 mL). The combined organic layers were dried ($MgSO_4$), filtered and concentrated *in vacuo*. The crude residue was purified by flash chromatography eluting with hexanes and then with EtOAc:hexanes (1:9 (v/v)). The purification afforded the title compound as a white solid (589 mg, 2.21 mmol, 74% yield).

R_f = 0.69 (EtOAc:hexanes 1:4 (v/v)). NMR Spectroscopy: ^{1}H NMR (400 MHz, $CDCl_3$, 25 ºC, δ): 9.11 (d, J = 2.76 Hz, 1H), 8.36 (d, J = 2.51 Hz, 1H), 7.37 (td, J = 8.34, 6.40 Hz, 1H), 7.05–6.88 (m, 2H), 4.07 (s, 3H). ^{13}C NMR (175 MHz, $CDCl_3$, 25 ºC, δ): 164.6, 163.5 (dd, J = 249.9 Hz, J = 11.6 Hz), 160.3 (dd, J = 250.8 Hz, J = 12.0 Hz), 144.2, 139.5, 134.7, 132.4 (m), 118.9, 118.4 (dd, J = 15.1 Hz, J = 3.5 Hz), 111.8 (dd. J = 21.4 Hz, J = 3.4 Hz), 104.6 (t, J = 25.5 Hz), 55.4. ^{19}F NMR (376 MHz, $CDCl_3$, 25 ºC, δ): –108.8 (m), –109.9 (m). Mass Spectrometry: HRMS (ESI-TOF) (m/z): calcd for $C_{12}H_9F_2N_2O_3$ ([M + H]$^+$), 267.0576, found, 267.0577.

***N*-(5-(2,4-Difluorophenyl)-6-methoxypyridin-3-yl)hydroxylamine (S8)**

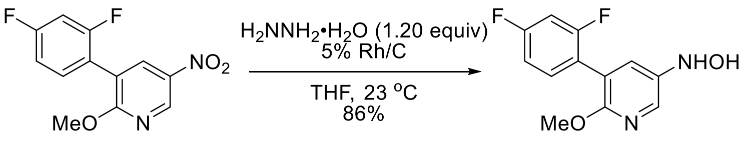

Under N_2 atmosphere, to a suspension of 3-(2,4-difluorophenyl)-2-methoxy-5-nitropyridine **(S7)** (0.300 g, 1.13 mmol, 1.00 equiv) and 5% Rh/C (6.5 mg, 0.30 mol% Rh) in THF (11.3 mL, 0.100 M) hydrazine monohydrate (0.068 g, 1.35 mmol, 1.20 equiv) was added dropwise. The reaction mixture was stirred at 23 °C for 1 h and filtered through a short pad of celite. The pad of celite was washed with EtOAc. The combined organic solution was concentrated *in vacuo* to afford the title

compound as a slightly light yellow solid (0.245 g, 0.973 mmol, 86% yield). The product was used directly without further purification.

R$_f$ = 0.41 (EtOAc:hexanes 1:1 (v/v)). NMR Spectroscopy: ^{1}H NMR (700 MHz, $(CD_3)_2SO$, 25 ºC, δ): 8.48 (br. s, 1H), 8.23 (br. s, 1H), 7.83 (br. s, 1H), 7.46 (q, *J* = 7.60 Hz, 1H), 7.33 (t, *J* = 9.68 Hz, 1H), 7.19 (br. s, 1H), 7.16 (t, *J* = 8.39 Hz, 1H), 3.78 (s, 3H). ^{13}C NMR (175 MHz, $(CD_3)_2SO$, 25 ºC, δ): 162.0 (dd, *J* = 245.3 Hz, *J* = 11.7 Hz), 159.4 (dd, *J* = 249.2 Hz, *J* = 12.7 Hz), 155.0, 142.7, 132.8 (q, *J* = 4.7 Hz), 131.4, 127.0, 120.8 (dd, *J* = 12.4 Hz, *J* = 3.5 Hz), 116.8, 111.5 (dd, *J* = 17.5 Hz, *J* = 3.5 Hz), 104.1 (t, *J* = 26.1 Hz), 53.3. ^{19}F NMR (376 MHz, $CDCl_3$, 25 ºC, δ): –112.0 (m), –112.7 (m). Mass Spectrometry: HRMS (ESI-TOF) (m/z): calcd for $C_{12}H_{11}F_2N_2O_2$ ([M + H]$^+$), 253.0783, found, 253.0784.

Methyl (5-(2,4-difluorophenyl)-6-methoxypyridin-3-yl)(hydroxy)carbamate (1k)

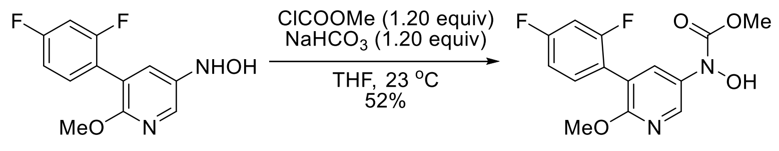

S8 **1k**

Under N_2 atmosphere, to a stirred suspension of *N*-(5-(2,4-difluorophenyl)-6-methoxypyridin-3-yl)hydroxylamine **(S8)** (0.250 g, 0.99 mmol, 1.00 equiv) and $NaHCO_3$ (0.099 g, 6.46 mmol, 1.20 equiv) in THF (4.96 mL, 0.200 M) at 23 °C was slowly added a solution of methyl chloroformate (0.112 g, 1.19 mmol, 1.20 equiv) in THF (4.96 mL, 0.200 M) via a syringe pump (at a rate of 10.0 mL/h). After the addition was complete, the reaction mixture was filtered through a short pad of celite and the celite was washed with EtOAc. The organic layers were combined and concentrated *in vacuo*. The residue was purified by chromatography on silica gel, eluting with EtOAc:hexanes (1:2 to 1:1 (v/v)), to afford the title compound as a slightly yellow solid (0.163 g, 0.52 mmol, 52% yield).

R$_f$ = 0.31 (EtOAc:hexanes 1:1 (v/v)). NMR Spectroscopy: ^{1}H NMR (700 MHz, $(CD_3)_2SO$, 25 ºC, δ): 10.54 (s, 1H), 8.32 (s, 1H), 7.77 (s, 1H), 7.49 (q, *J* = 8.03 Hz, 1H), 7.35 (t, *J* = 9.68 Hz, 1H), 7.18 (t, *J* = 8.17 Hz, 1H), 3.85 (s, 3H), 3.73 (s, 3H). ^{13}C NMR (175 MHz, $(CD_3)_2SO$, 25 ºC, δ): 162.2 (d, *J* = 246.9 Hz), 159.5 (d, *J* = 247.5 Hz), 157.7, 155.1, 139.9, 133.7, 133.4, 132.9 (m), 119.9 (d, *J* = 15.2 Hz), 116.9, 111.7 (dd, *J* = 22.8 Hz, *J* = 5.3 Hz), 104.2 (t, *J* = 26.3 Hz), 53.81, 53.1. ^{19}F NMR (376 MHz, $(CD_3)_2SO$, 25 ºC, δ): –111.2 (m), –112.1 (m). Mass Spectrometry: HRMS (ESI-TOF) (m/z): calcd for $C_{14}H_{13}F_2N_2O_4$ ([M + H]$^+$), 311.0838, found, 311.0841.

2-(4-(*tert*-Butyl)phenoxy)-5-nitropyridine (S9)

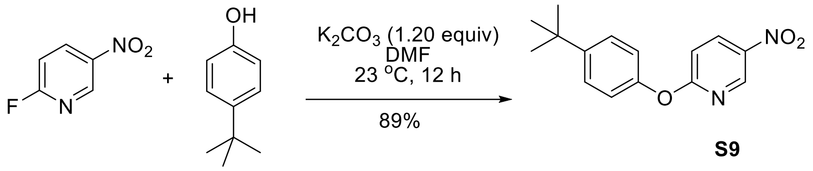

S9

Under N_2 atmosphere, to a mixture of 4-(*tert*-butyl)phenol (1.26 g, 8.40 mmol, 1.20 equiv) and K_2CO_3 (1.16 g, 8.40 mmol, 1.20 equiv) in DMF (25.0 mL, 0.336 M) was added 2-fluoro-5-nitropyridine (1.00 g, 7.04 mmol, 1.00 equiv) and the reaction mixture was stirred at 23 °C for 16 h. The reaction mixture was poured to LiCl solution (100 mL), extracted with EtOAc. The combined organic layers was dried (MgSO4), filtered and concentrated *in vacuo* and purified by chromatography on silica gel, eluting with hexanes:EtOAc (20:1 to 10:1 (v/v)), to afford the title compound as a light yellow solid (1.71 g, 6.27 mmol, 89% yield).

R_f = 0.70 (hexanes:EtOAc 4:1 (v/v)). ^{1}H NMR (700 MHz, $(CD_3)_2SO$, 25 ºC, δ): 9.02 (d, J = 3.01 Hz, 1H), 8.60 (dd, J = 9.04, 3.01 Hz, 1H), 7.47 (d, J = 8.60 Hz, 2H), 7.22 (d, J = 9.04 Hz, 1H), 7.13 (d, J = 8.60 Hz, 2H), 1.30 (s, 9H). ^{13}C NMR (175 MHz, $(CD_3)_2SO$, 25 ºC, δ): 166.5, 150.3, 148.0, 144.7, 140.3, 135.7, 126.6, 121.0, 111.5, 34.2, 31.2.

N-(6-(4-(*tert*-Butyl)phenoxy)pyridin-3-yl)hydroxylamine (S10)

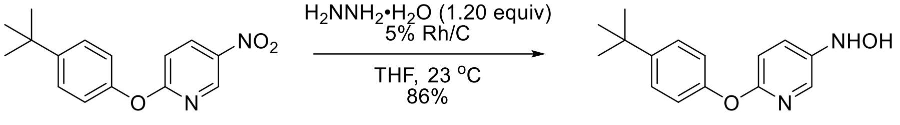

S9 **S10**

Under N_2 atmosphere, to a suspension of 2-(4-(*tert*-butyl)phenoxy)-5-nitropyridine **(S9)** (0.600 g, 2.20 mmol, 1.00 equiv) and 5% Rh/C (12.7 mg, 0.30 mol% Rh) in THF (11.0 mL, 0.200 M) hydrazine monohydrate (0.132 g, 2.64 mmol, 1.20 equiv) was added dropwise. The reaction mixture was stirred at 23 °C for 1 h and then filtered through a short pad of celite. The celite was washed with EtOAc. The combined organic solution was concentrated *in vacuo* to afford the title compound as an off–white solid (0.495 g, 1.92 mmol, 86% yield). The product was used directly without further purification.

R_f = 0.55 (EtOAc:hexanes 1:1 (v/v)). NMR Spectroscopy: ^{1}H NMR (700 MHz, $(CD_3)_2SO$, 25 ºC, δ): 8.50 (s, 1H), 8.35 (s, 1H), 7.76 (d, J = 2.58 Hz, 1H), 7.39 (d, J = 8.60 Hz, 2H), 7.34 (dd, J = 8.82, 2.80 Hz, 1H), 6.95 (d, J = 8.61 Hz, 2H), 6.89 (d, J = 8.60 Hz, 1H), 1.30 (s, 9H). ^{13}C NMR (175 MHz, $(CD_3)_2SO$, 25 ºC, δ): 156.8, 153.0, 145.8, 144.5, 132.0, 126.3, 125.8, 119.4, 111.8, 34.1, 31.3. Mass Spectrometry: HRMS (ESI-TOF) (m/z): calcd for $C_{15}H_{19}N_2O_2$ ($[M + H]^+$), 259.1441, found, 259.1442.

Methyl (6-(4-(*tert*-butyl)phenoxy)pyridin-3-yl)(hydroxy)carbamate (1l)

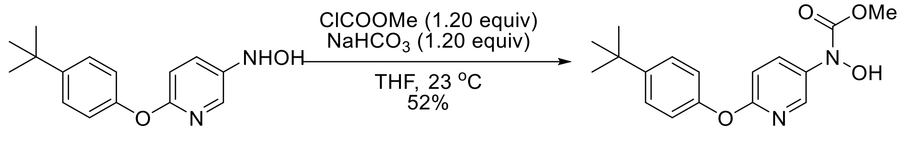

Under N_2 atmosphere, to a stirred suspension of *N*-(6-(4-(*tert*-butyl)phenoxy)pyridin-3-yl)hydroxylamine **(S10)** (0.491 g, 0.990 mmol, 1.00 equiv) and $NaHCO_3$ (0.099 g, 1.19 mmol, 1.20 equiv) in THF (4.96 mL, 0.100 M) at 23 °C was slowly added a solution of methyl chloroformate (0.112 g, 1.19 mmol, 1.20 equiv) in THF (4.96 mL, 0.100 M) via a syringe pump (at a rate of 10.0 mL/h). After the addition was complete, the reaction mixture was filtered through a short pad of celite and the celite was washed with EtOAc. The organic layers were combined and concentrated in vacuo. The residue was purified by chromatography on silica gel, eluting with EtOAc:hexanes (1:2 to 1:1 (v/v)), to afford the title compound as a off–white solid (0.163 g, 0.52 mmol, 52% yield).

R_f = 0.48 (EtOAc:hexanes 1:1 (v/v)). NMR Spectroscopy: ^1H NMR (700 MHz, $(CD_3)_2SO$, 25 ºC, δ): 10.53 (s, 1H), 8.23 (d, *J* = 2.58 Hz, 1H), 7.91 (dd, *J* = 9.03, 2.58 Hz, 1H), 7.42 (d, *J* = 8.60 Hz, 2H), 7.08–6.97 (m, 3H), 3.72 (s, 3H), 1.30 (s, 9H). ^{13}C NMR (175 MHz, $(CD_3)_2SO$, 25 ºC, δ): 160.0, 155.0, 151.7, 146.8, 140.2, 134.8, 133.4, 126.4, 120.5, 111.0, 53.1, 34.2, 31.3. Mass Spectrometry: HRMS (ESI-TOF) (m/z): calcd for $C_{17}H_{21}N_2O_4$ ($[M + H]^+$), 317.1496, found, 317.1497.

Methyl (5-(5-formylfuran-2-yl)-6-methoxypyridin-3-yl)(hydroxy)carbamate (1m)

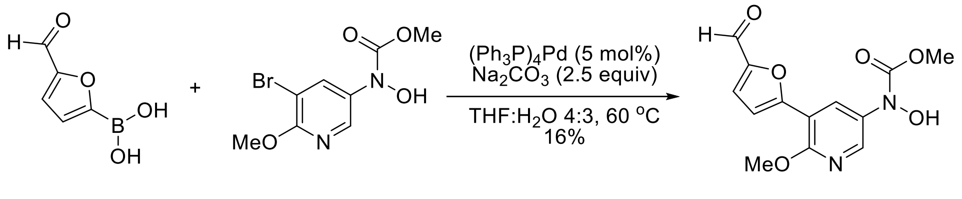

Methyl (5-bromo-6-methoxypyridin-3-yl)(hydroxy)carbamate (0.400 g, 1.44 mmol, 1.00 equiv), (5-formylfuran-2-yl)boronic acid (0.283 g, 2.02 mmol, 1.40 equiv), Na_2CO_3 (0.383 g, 3.61 mmol, 2.5 equiv), THF:H_2O 4:3 (8.42 mL, 0.200 M), and palladium-tetrakis(triphenylphosphine) (0.0830 g, 0.0700 mmol, 0.05 equiv) were degassed via three freeze-pump-thaw cycles. The resulting mixture was heated at 60 °C overnight and then allowed to cool to room temperature after which water was added. The mixture was then extracted with dichloromethane and the organic extracts was dried with $MgSO_4$, filtered and concentrated in vacuo. The residue was purified by chromatography on silica gel, eluting with EtOAc:hexanes (3:8 to 1:1 (v/v)), to afford the pure cross-coupled product as a slightly light orange solid (0.070 g, 0.24 mmol, 16% yield).

R_f = 0.21 (EtOAc:hexanes 4:6 (v/v)). NMR Spectroscopy: ^1H NMR (700 MHz, $(CD_3)_2SO$,

25 ºC, δ): 10.65 (s, 1H), 9.65 (s, 1H), 8.38 (d, *J* = 2.58 Hz, 1H), 8.32 (d, *J* = 2.58 Hz, 1H), 7.68 (d, *J* = 3.44 Hz, 1H), 7.28 (d, *J* = 3.87 Hz, 1H), 4.05 (s, 3H), 3.76 (s, 3H). ^{13}C NMR (175 MHz, $(CD_3)_2SO$, 25 ºC, δ): 178.2, 156.4, 155.0, 152.4, 151.6, 139.9, 133.8, 127.7, 125.2, 113.6, 111.4, 54.2, 53.2. Mass Spectrometry: HRMS (ESI-TOF) (m/z): calcd for $C_{13}H_{13}N_2O_6$ ([M + H]$^+$), 294.0799, found, 294.0801.

5-Nitro-2-(1*H*-pyrazol-1-yl)pyridine (S11)

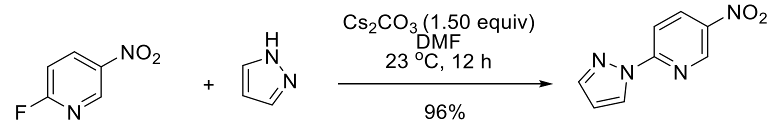

S11

Under N_2 atmosphere, a solution of 2-fluoro-5-nitropyridine (0.71 g, 5.00 mmol, 1.00 equiv) was added to a mixture of 1*H*-pyrazole (0.51 g, 7.50 mmol, 1.50 equiv) and Cs_2CO_3 (2.44 g, 7.50 mmol, 1.50 equiv) in DMF (25.0 mL, 0.300 M) and the reaction mixture was stirred at 23 °C for 20 h. The reaction mixture was poured to LiCl solution (100 mL), extracted with EtOAc. The combined organic layers was dried ($MgSO_4$), filtered and concentrated *in vacuo*. The residue was recrystalized from hexanes/EtOAc,, to afford the title compound as a yellow solid (0.910 g, 4.78 mmol, 96% yield).

R$_f$ = 0.77 (hexanes:EtOAc 5:1 (v/v)). NMR Spectroscopy: ^{1}H NMR (700 MHz, $(CD_3)_2SO$, 25 ºC, δ): 9.29 (br. s., 1H), 8.75 (d, *J* = 8.17 Hz, 1H), 8.73–8.71 (m, 1H), 8.12 (d, *J* = 9.04 Hz, 1H), 7.99–7.98 (m, 1H), 6.72–6.70 (m, 1H). ^{13}C NMR (175 MHz, $(CD_3)_2SO$, 25 ºC, δ): 153.7, 145.0, 144.4, 142.3, 135.3, 128.5, 112.2, 110.0. Mass Spectrometry: HRMS (ESI-TOF) (m/z): calcd for $C_8H_7N_4O_2$ ([M + H]$^+$), 191.0564, found, 191.0562.

***N*-(6-(1*H*-Pyrazol-1-yl)pyridin-3-yl)-*N*-hydroxyacetamide (1n)**

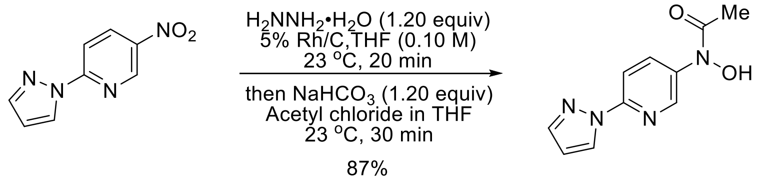

S11 **1n**

Under N_2 atmosphere, a suspension of 5-nitro-2-(1*H*-pyrazol-1-yl)pyridine (**S11**) (200 mg, 1.05 mmol, 1.00 equiv) and 5% Rh/C (12.6 mg, 0.60 mol% Rh) in THF (10.5 mL, 0.100 M) was stirred at 23 °C. Hydrazine monohydrate (63.1 mg, 1.26 mmol, 1.20 equiv) was added dropwise. The reaction mixture was stirred at 23 °C for 20 min. $NaHCO_3$ (106 mg, 1.26 mmol, 1.20 equiv) was added, followed by dropwise addition of acetyl chloride (98.9 mg, 1.26 mmol, 1.20 equiv) in THF (10.5 mL, 0.120 M) at 23 ºC. The reaction mixture was stirred at 23 °C for 30 min and then filtered through a short pad of celite. The celite was washed with EtOAc. The combined organic solution was concentrated in vacuo. The residue was recrystalized from hexanes/EtOAc, to afford

the title compound as a yellow solid (200 mg, 0.917 mmol, 87% yield).

R*f* = 0.26 (hexanes:EtOAc 1:1(v/v)). NMR Spectroscopy: ^{1}H NMR (700 MHz, $(CD_3)_2SO$, 25 ºC, δ): 10.95 (br. s., 1H), 8.77 (br. s., 1H), 8.59 (d, *J* = 2.15 Hz, 1H), 8.22 (dd, *J* = 9.03, 2.58 Hz, 1H), 7.95 (d, *J* = 9.03 Hz, 1H), 7.82 (s, 1H), 6.60–6.56 (m, 1H), 2.27 (s, 3H). ^{13}C NMR (175 MHz, $(CD_3)_2SO$, 25 ºC, δ): 170.6, 146.9, 142.1, 139.1, 136.7, 130.1, 126.9, 111.7, 108.2, 22.2. Mass Spectrometry: HRMS (ESI-TOF) (m/z): calcd for $C_{10}H_{11}N_4O_2$ ($[M + H]^+$), 219.0877, found, 219.0877.

5-Nitro-2-(1*H*-1,2,4-triazol-1-yl)pyridine (S12)

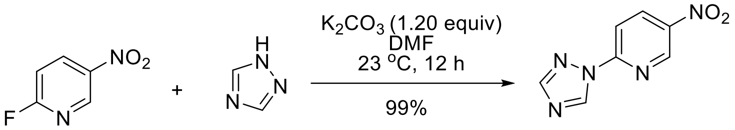

Under N_2 atmosphere, to a mixture of 1*H*-1,2,4-triazole (0.580 g, 8.40 mmol, 1.20 equiv) and K_2CO_3 (1.16 g, 8.40 mmol, 1.20 equiv) in DMF (25.0 mL, 0.336 M) was added 2-fluoro-5-nitropyridine (1.00 g, 7.04 mmol, 1.00 equiv) and the reaction mixture was stirred at 23 °C for 20 h. The reaction mixture was poured to LiCl solution (100 mL), extracted with EtOAc. The combined organic layers was dried ($MgSO_4$), filtered and concentrated *in vacuo* and purified by chromatography on silica gel, eluting with hexanes:EtOAc (5:1 to 2:1 (v/v)), to afford the title compound as a yellow solid (1.34 g, 7.01 mmol, 99% yield).

R*f* = 0.58 (hexanes:EtOAc 1:1(v/v)). ^{1}H NMR (700 MHz, $(CD_3)_2SO$, 25 ºC, δ): 9.52 (br. s., 1H), 9.35 (s, 1H), 8.84 (d, *J* = 8.17 Hz, 1H), 8.44 (s, 1H), 8.10 (d, *J* = 9.04 Hz, 1H). ^{13}C NMR (175 MHz, $(CD_3)_2SO$, 25 ºC, δ): 154.0, 151.7, 145.1, 143.5, 143.4, 136.0, 113.4.

***N*-(6-(1*H*-1,2,4-Triazol-1-yl)pyridin-3-yl)hydroxylamine (S13)**

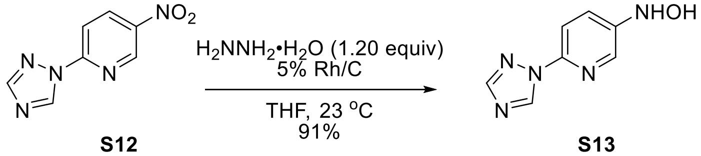

Under N_2 atmosphere, to a suspension of 5-nitro-2-(1*H*-1,2,4-triazol-1-yl)pyridine **(S12)** (0.500 g, 2.26 mmol, 1.00 equiv) and 5% Rh/C (15.0 mg, 0.30 mol% Rh) in THF (13.0 mL, 0.200 M) hydrazine monohydrate (0.190 g, 3.14 mmol, 1.20 equiv) was added dropwise. The reaction mixture was stirred at 23 °C for 1 h. The reaction mixture was filtered through a short pad of celite, washed with EtOAc, and concentrated in vacuo to afford the title compound as a slightly light yellow (0.691 g, 2.38 mmol, 91% yield). The product was used directly without further purification.

R*f* = 0.36 (EtOAc:hexanes 1:0 (v/v)). NMR Spectroscopy: ^{1}H NMR (700 MHz, $(CD_3)_2SO$, 25 ºC, δ): 9.20 (s, 1H), 8.80 (s, 1H), 8.73 (d, *J* = 2.15 Hz, 1H), 8.22 (s, 1H), 8.05 (d, *J* = 2.58 Hz, 1H), 7.71 (d, *J* = 9.03 Hz, 1H), 7.45 (dd, *J* = 8.82, 2.80 Hz, 1H). ^{13}C NMR (175 MHz, $(CD_3)_2SO$,

25 ºC, δ): 152.3, 147.9, 141.7, 141.0, 132.8, 123.0, 113.3. Mass Spectrometry: HRMS (ESI-TOF) (m/z): calcd for $C_7H_8N_5O$ ([M + H]$^+$), 178.0723, found, 178.0723.

Methyl (6-(1*H*-1,2,4-triazol-1-yl)pyridin-3-yl)(hydroxy)carbamate (1o)

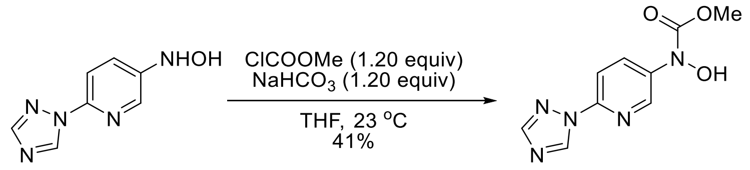

Under N_2 atmosphere, to a stirred suspension of *N*-(6-(1*H*-1,2,4-triazol-1-yl)pyridin-3-yl)hydroxylamine **(S13)** (0.150 g, 0.85 mmol, 1.00 equiv) and $NaHCO_3$ (0.084 g, 1.02 mmol, 1.20 equiv) in THF (4.23 mL, 0.100 M) at 23 °C was slowly added a solution of methyl chloroformate (0.095 g, 1.02 mmol, 1.20 equiv) in THF (4.23 mL, 0.100 M) via a syringe pump (at a rate of 10.0 mL/h). After the addition was complete, a white solid precipitated from the reaction mixture. The reaction mixture was then filtered through and the solid was washed with water to afford the title compound as a white solid (0.081 g, 0.34 mmol, 41% yield).

R_f = 0.08 (MeOH:EtOAc 1:9 (v/v)). NMR Spectroscopy: ^{1}H NMR (700 MHz, $(CD_3)_2SO$, 25 ºC, δ): 10.80 (s, 1H), 9.33 (s, 1H), 8.72 (d, *J* = 2.58 Hz, 1H), 8.29 (s, 1H), 8.19–8.17 (m, 1H), 7.89 (d, *J* = 9.03 Hz, 1H), 3.80 (s, 3H). ^{13}C NMR (175 MHz, $(CD_3)_2SO$, 25 ºC, δ): 154.6, 152.9, 144.6, 141.8, 139.2, 138.5, 130.2, 112.9, 53.4. Mass Spectrometry: HRMS (ESI-TOF) (m/z): calcd for $C_9H_9N_5O_3Na$ ([M + Na]$^+$), 258.0598, found, 258.0598.

1-(5-Nitropyridin-2-yl)-1*H*-benzo[*d*]imidazole (S14)

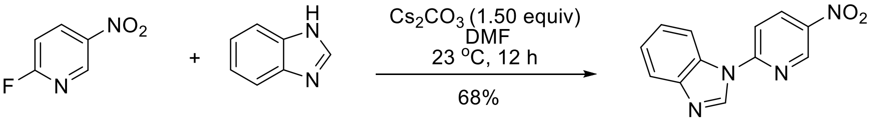

Under N_2 atmosphere, a solution of 2-fluoro-5-nitropyridine (1.32 g, 9.31 mmol, 1.10 equiv) in DMF (20.0 mL, 0.466 M) was added to a mixture of 1*H*-benzo[*d*]imidazole (1.00 g, 8.46 mmol, 1.00 equiv) and Cs_2CO_3 (2.76 g, 8.46 mmol, 1.50 equiv) in DMF (22.3 mL, 0.379 M) at 23 °C. The resulting mixture was stirred at 23 °C for 7 h. The reaction mixture was poured to LiCl solution (100 mL), extracted with EtOAc. The combined organic layers was dried ($MgSO_4$), filtered and concentrated *in vacuo*. The residue was recrystalized from hexanes/EtOAc, to afford the title compound as a yellow solid (1.38 g, 5.75 mmol, 68% yield).

R_f = 0.29 (hexanes:EtOAc 1:1 (v/v)). NMR Spectroscopy: ^{1}H NMR (700 MHz, $CDCl_3$, 25 ºC, δ): 9.46 (d, *J* = 2.58 Hz, 1H), 8.75–8.72 (m, 1H), 8.72–8.68 (m, 1H), 8.22 (d, *J* = 8.17 Hz, 1H), 7.90 (d, *J* = 7.74 Hz, 1H), 7.79 (dd, *J* = 8.82, 1.94 Hz, 1H), 7.49–7.46 (m, 1H), 7.45–7.42 (m, 1H). ^{13}C NMR (175 MHz, $CDCl_3$, 25 ºC, δ): 153.6, 145.9, 144.9, 141.9, 141.1, 134.7, 131.8, 125.5, 124.7,

121.3, 113.7, 112.8. Mass Spectrometry: HRMS (ESI-TOF) (m/z): calcd for $C_{12}H_9N_4O_2$ ([M + H]$^+$), 241.0720, found, 241.0724.

N-(6-(1H-Benzo[d]imidazol-1-yl)pyridin-3-yl)-N-hydroxyacetamide (1p)

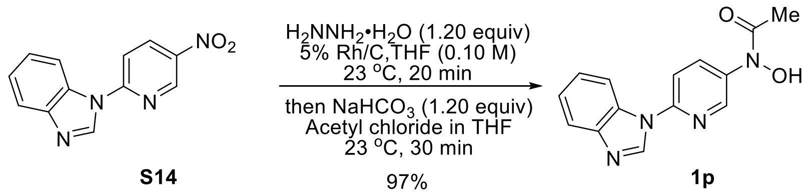

Under N_2 atmosphere, a suspension of 1-(5-nitropyridin-2-yl)-1*H*-benzo[*d*]imidazole (**S14**) (600 mg, 2.50 mmol, 1.00 equiv) and 5% Rh/C (28.7 mg, 0.60 mol% Rh) in THF (25.0 mL, 0.100 M) was stirred at 23 °C. Hydrazine monohydrate (150 mg, 3.00 mmol, 1.20 equiv) was added dropwise. The reaction mixture was stirred at 23 °C for 20 min. $NaHCO_3$ (252 mg, 3.00 mmol, 1.20 equiv) was added, followed by dropwise addition of a solution of acetyl chloride (236 mg, 3.00 mmol, 1.20 equiv) in THF (25.0 mL, 0.120 M). The reaction mixture was stirred at 23 °C for 30 min and then filtered through a short pad of celite. The celite was washed with EtOAc. The combined organic solution was concentrated in vacuo. The residue was recrystalized from hexanes/EtOAc, to afford the title compound as a yellow solid (650 mg, 2.42 mmol, 97% yield).

R_f = 0.15 (EtOAc). NMR Spectroscopy: ^{1}H NMR (700 MHz, $(CD_3)_2SO$, 25 ºC, δ): 11.01 (br. s., 1H), 8.92 (s, 2H), 8.28 (dd, *J* = 9.03, 2.58 Hz, 1H), 8.25 (d, *J* = 7.74 Hz, 1 H), 7.97 (d, *J* = 9.03 Hz, 1H), 7.77 (d, *J* = 7.74 Hz, 1H), 7.40–7.36 (m, 1H), 7.35–7.32 (m, 1H), 2.29 (s, 3H). ^{13}C NMR (175 MHz, $(CD_3)_2SO$, 25 ºC, δ): 170.8, 145.5, 144.1, 142.2, 139.7, 136.6, 131.9, 130.0, 123.9, 123.0, 119.9, 114.4, 113.7, 22.2. Mass Spectrometry: HRMS (ESI-TOF) (m/z): calcd for $C_{14}H_{13}N_4O_2$ ([M + H]$^+$), 269.1033, found, 269.1037.

1-(5-Nitropyridin-2-yl)-1H-benzo[d][1,2,3]triazole (S15)

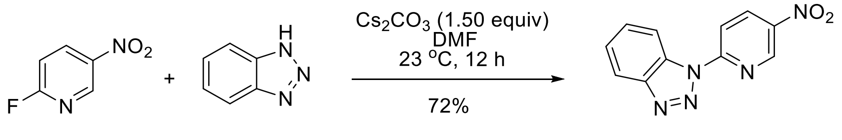

Under N_2 atmosphere, to a mixture of 1*H*-benzo[*d*][1,2,3]triazole (0.890 g, 7.50 mmol, 1.50 equiv) and Cs_2CO_3 (2.44 g, 7.50 mmol, 1.50 equiv) in DMF (25.0 mL, 0.300 M) was added 2-fluoro-5-nitropyridine (0.71 g, 5.00 mmol, 1.00 equiv) and the reaction mixture was stirred at 23 °C for 20 h. The reaction mixture was poured to LiCl solution (100 mL), extracted with EtOAc. The combined organic layers was dried ($MgSO_4$), filtered and concentrated *in vacuo*. The residue was recrystalized from hexanes/EtOAc, to afford the title compound as a yellow solid (0.870 g, 3.61 mmol, 72% yield).

R_f = 0.54 (hexanes:EtOAc 5:1 (v/v)). ^{1}H NMR (700 MHz, $(CD_3)_2SO$, 25 ºC, δ): 9.52 (br. s., 1H), 8.90 (d, *J* = 9.03 Hz, 1H), 8.65 (d, *J* = 8.17 Hz, 1H), 8.51 (d, *J* = 9.04 Hz, 1H), 8.28 (d, *J* = 8.17

Hz, 1H), 7.82 (t, *J* = 7.53 Hz, 1H), 7.63 (t, *J* = 7.53 Hz, 1H). ^{13}C NMR (175 MHz, $(CD_3)_2SO$, 25 ºC, δ): 153.8, 146.3, 145.1, 142.7, 135.4, 130.9, 130.2, 126.1, 120.1, 114.7, 114.3. Mass Spectrometry: HRMS (ESI-TOF) (m/z): calcd for $C_{11}H_8N_5O_2$ ($[M + H]^+$), 242.0673, found, 242.0674.

N-(6-(1*H*-Benzo[*d*][1,2,3]triazol-1-yl)pyridin-3-yl)-*N*-hydroxyacetamide (1q)

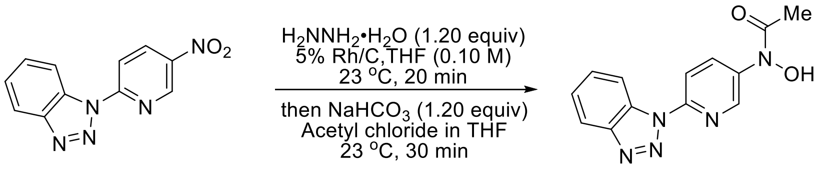

Under N_2 atmosphere, a suspension of 1-(5-nitropyridin-2-yl)-1*H*-benzo[*d*][1,2,3]triazole (**S15**) (253 mg, 1.05 mmol, 1.00 equiv) and 5% Rh/C (12.6 mg, 0.60 mol% Rh) in THF (21.0 mL, 0.0500 M) was stirred at 23 °C. Hydrazine monohydrate (63.1 mg, 1.26 mmol, 1.20 equiv) was added dropwise. The reaction mixture was stirred at 23 °C for 20 min. $NaHCO_3$ (106 mg, 1.26 mmol, 1.20 equiv) was added, followed by dropwise addition of a solution of acetyl chloride (98.9 mg, 1.26 mmol, 1.20 equiv) in THF (10.5 mL, 0.120 M). The reaction mixture was stirred at 23 °C for 30 min and then filtered through a short pad of celite. The celite was washed with EtOAc. The combined organic solution was concentrated in vacuo. The residue was recrystalized from hexanes/EtOAc, to afford the title compound as a yellow solid (281 mg, 1.04 mmol, 99% yield).

R_f = 0.34 (hexanes:EtOAc 1:1 (v/v)). NMR Spectroscopy: ^{1}H NMR (700 MHz, $(CD_3)_2SO$, 25 ºC, δ): 11.07 (s, 1H), 9.02 (d, *J* = 2.58 Hz, 1H), 8.56 (d, *J* = 8.17 Hz, 1H), 8.39 (dd, *J* = 9.03, 2.58 Hz, 1H), 8.28 (d, *J* = 9.03 Hz, 1H), 8.22–8.19 (m, 1H), 7.72 (td, *J* = 7.64, 1.08 Hz, 1H), 7.55 (ddd, *J* = 8.07, 6.99, 0.86 Hz, 1H), 2.31 (s, 3H). ^{13}C NMR (175 MHz, $(CD_3)_2SO$, 25 ºC, δ): 171.2, 146.4, 146.0, 139.0, 137.5, 130.8, 130.0, 129.2, 125.3, 119.6, 114.3, 114.2, 22.3. Mass Spectrometry: HRMS (ESI-TOF) (m/z): calcd for $C_{13}H_{12}N_5O_2$ ($[M + H]^+$), 270.0986, found, 270.0986.

5-Fluoro-1-(5-nitropyridin-2-yl)-1*H*-indole (S16)

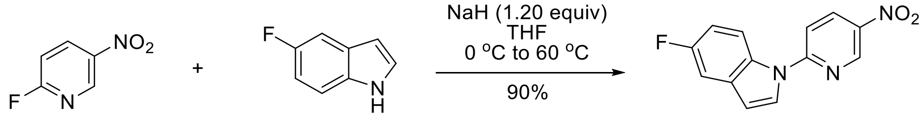

Under N_2 atmosphere, 5-fluoro-1*H*-indole (1.14 g, 8.45 mmol, 1.20 equiv) was dissolved in DMF (35.2 mL, 0.240 M) and stirred at 0 °C. NaH (0.338g, 8.45 mmol, 1.20 equiv, 60 % dispersion in mineral oil) was added in portionwise. After 30 min, 2-fluoro-5-nitropyridine (1.00 g, 7.04 mmol. 1.00 equiv) was added and then the reaction mixture was slowly warmed up to 60 °C and stirred at 60 ºC for 16 h. The reaction mixture was poured to a solution of LiCl (100 mL), extracted with EtOAc, and washed with brine. The combined organic layers was dried ($MgSO_4$), filtered and

concentrated *in vacuo*. The residue was purified by chromatography on silica gel, eluting with hexanes:EtOAc (10:1 to 5:1 (v/v)), to afford the title compound as a yellow solid (1.63 g, 6.34 mmol, 90% yield).

R_f = 0.20 (hexanes:EtOAc 5:1 (v/v)). NMR Spectroscopy: ^{1}H NMR (700 MHz, $CDCl_3$, 25 ºC, δ): 9.39 (d, *J* =2.15 Hz, 1H), 8.58 (dd, *J* = 9.03, 2.58 Hz, 1H), 8.51 (dd, *J* = 9.25, 4.52 Hz, 1H), 7.76 (d, *J* = 3.87 Hz, 1H), 7.54 (d, *J* = 9.04 Hz, 1H), 7.30 (dd, *J* = 9.03, 2.58 Hz, 1H), 7.11 (td, *J* = 9.03, 2.58 Hz, 1H), 6.78 (d, *J* = 3.01 Hz, 1H). ^{13}C NMR (175 MHz, $CDCl_3$, 25 ºC, δ): 159.5 (d, *J* = 238.8 Hz), 155.8, 145.6(d, *J* = 3.50 Hz), 140.4, 134.0, 132.1(*J* = 9.94 Hz), 126.5, 116.4 (d, *J* = 9.00 Hz), 112.5 (d, *J* = 24.7 Hz), 112.0, 108.9 (d, *J* = 3.76 Hz), 106.8 (d, *J* = 23.6 Hz). ^{19}F NMR (376 MHz, $CDCl_3$, 25 ºC, δ): –121.5.0 (m). Mass Spectrometry: HRMS (ESI-TOF) (m/z): calcd for $C_{13}H_9FN_3O_2$ ([M + H]$^+$), 258.0673, found, 258.0675.

***N*-(6-(5-Fluoro-1*H*-indol-1-yl)pyridin-3-yl)-*N*-hydroxyacetamide (1r)**

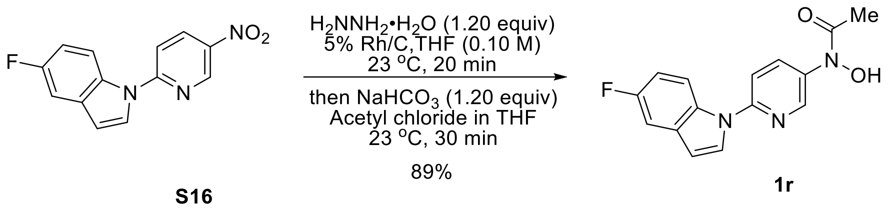

S16 **1r**

Under N_2 atmosphere, a suspension of 5-fluoro-1-(5-nitropyridin-2-yl)-1*H*-indole (**S16**) (0.500 g, 1.94 mmol, 1.00 equiv) and 5% Rh/C (21.8 mg, 0.60 mol% Rh) in THF (20.0 mL, 0.100 M) was stirred at 23 °C. Hydrazine monohydrate (117 mg, 2.33 mmol, 1.20 equiv) was added dropwise. The reaction mixture was stirred at 23 °C for 20 min. $NaHCO_3$ (196 mg, 2.33 mmol, 1.20 equiv) was added, followed by dropwise addition of a solution of acetyl chloride (183 mg, 2.33 mmol, 1.20 equiv) in THF (20 mL, 0.120 M). The reaction mixture was stirred at 23 °C for 30 min and then filtered through a short pad of celite. The celite was washed with EtOAc. The combined organic solution was concentrated in vacuo. The residue was purified by chromatography on silica gel, eluting with hexanes:EtOAc (2:1 to 1:1 (v/v)), to afford the title compound as a yellow solid (0.490 g, 1.72 mmol, 89% yield).

R_f = 0.14 (hexanes:EtOAc 1:1 (v/v)). NMR Spectroscopy: ^{1}H NMR (700 MHz, $(CD_3)_2SO$, 25 ºC, δ): 10.95 (br. s., 1H), 8.84 (br. s., 1H), 8.39 (dd, *J* = 8.82, 4.52 Hz, 1H), 8.23–8.17 (m, 1H), 8.09 (d, *J* = 3.44 Hz, 1H), 7.79 (d, *J* = 8.60 Hz, 1H), 7.43 (dd, *J* = 9.25, 2.37 Hz, 1H), 7.10 (td, *J* = 9.14, 2.37 Hz, 1H), 6.75 (d, *J* = 3.01 Hz, 1H), 2.27 (br. s., 3H), ^{13}C NMR (175 MHz, $(CD_3)_2SO$, 25 ºC, δ): 170.6, 157.8 (d, *J* = 233.2 Hz),148.0, 139.4, 135.4, 131.3, 130.6 (d, *J* = 10.1 Hz), 130.2, 128.2, 115.2 (d, *J* = 9.14 Hz), 113.6, 110.8 (d, *J* = 25.2 Hz), 105.6 (d, *J* = 23.3 Hz), 105.2 (d, *J* = 3.38 Hz), 22.1. ^{19}F NMR (376 MHz, $CDCl_3$, 25 ºC, δ): –123.6 (m). Mass Spectrometry: HRMS (ESI-TOF) (m/z): calcd for $C_{15}H_{13}FN_3O_2$ ([M + H]$^+$), 286.0986, found, 286.0989.

5-Bromo-1-(5-nitropyridin-2-yl)-1*H*-pyrrolo[2,3-*b*]pyridine (S17)

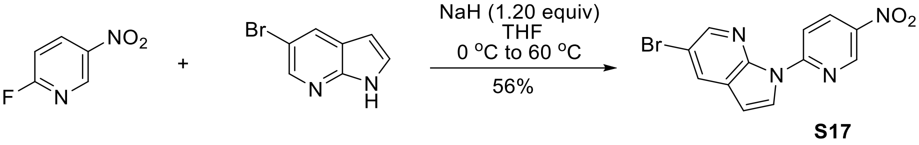

Under N_2 atmosphere, 5-bromo-1*H*-pyrrolo[2,3-*b*]pyridine (1.66 g, 8.45 mmol, 1.20 equiv) was dissolved in DMF (35.2 mL, 0.240 M) and stirred at 0 ºC. NaH (0.338g, 8.45 mmol, 1.20 equiv, 60 % dispersion in mineral oil) was added in portionwise. After 30 min, 2-fluoro-5-nitropyridine (1.00 g, 7.04 mmol. 1.00 equiv) was added and then the reaction mixture was slowly warmed up to 60 ˚C and stirred at 60 °C for 16 h. The reaction mixture was poured to a solution of LiCl (100 mL), extracted with EtOAc. The combined organic layers washed with brine, dried ($MgSO_4$), filtered and concentrated in vacuo. The residue was purified by chromatography on silica gel, eluting with hexanes:EtOAc (20:1 to 10:1 (v/v)), to afford the title compound as a yellow solid (1.26 g, 3.94 mmol, 56% yield).

R_f = 0.69 (hexanes:EtOAc 5:1 (v/v)). NMR Spectroscopy: ^{1}H NMR (500 MHz, $CDCl_3$, 25 ºC, δ): 9.31 (d, *J* = 2.58 Hz, 1H), 9.30 (d, *J* = 9.04 Hz, 1H), 8.64 (d, *J* = 2.58 Hz, 1H), 8.62 (d, *J* = 2.58 Hz, 1H), 8.49 (d, *J* = 4.30 Hz, 1H), 8.46 (d, *J* = 2.15 Hz, 1H), 8.10 (d, *J* = 2.15 Hz, 1H). ^{13}C NMR (175 MHz, $CDCl_3$, 25 ºC, δ): 153.8, 146.4, 144.9, 144.3, 141.1, 134.0, 131.9, 127.8, 125.8, 114.6, 114.4, 104.5. Mass Spectrometry: HRMS (ESI-TOF) (m/z): calcd for $C_{12}H_8BrN_4O_2$ ($[M + H]^+$), 318.9825, found, 318.9826.

***N*-(6-(5-Bromo-1*H*-pyrrolo[2,3-b]pyridin-1-yl)pyridin-3-yl)-*N*-hydroxyacetamide (1s)**

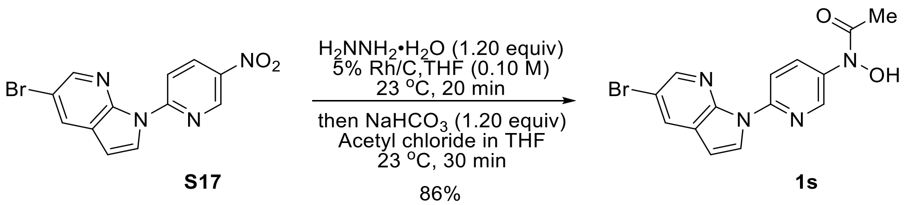

Under N_2 atmosphere, a suspension of 5-bromo-1-(5-nitropyridin-2-yl)-1*H*-pyrrolo[2,3-*b*]pyridine (**S17**) (350 mg, 1.10 mmol, 1.00 equiv) and 5% Rh/C (12.6 mg, 0.60 mol% Rh) in THF (11.0 mL, 0.100 M) was stirred at 23 °C. Hydrazine monohydrate (65.9 mg, 1.32 mmol, 1.20 equiv) was added dropwise. The reaction mixture was stirred at 23 °C for 20 min. $NaHCO_3$ (111 mg, 1.32 mmol, 1.2 equiv) was added, followed by dropwise addition of a solution of acetyl chloride (104 mg, 1.32 mmol, 1.20 equiv) in THF (11.0 mL, 0.120 M). The reaction mixture was stirred at 23 °C for 30 min and then filtered through a short pad of celite. The celite was washed with EtOAc. The combined organic solution was concentrated in vacuo. The residue was purified by chromatography on silica gel, eluting with hexanes:EtOAc (5:1 to 1:1 (v/v)), to afford the title compound as a yellow solid (330 mg, 0.950 mmol, 86% yield).

R_f = 0.17 (hexanes:EtOAc 1:1 (v/v)). NMR Spectroscopy: ^{1}H NMR (700 MHz, $(CD_3)_2SO$, 25 ºC, δ): 10.94 (br. s., 1H), 8.83 (br. s., 1H), 8.71 (d, *J* = 8.60 Hz, 1H), 8.46 (br. s., 1H), 8.39 (d, *J* = 3.44

Hz, 1H), 8.36 (br. s., 1H), 8.24 (d, *J* = 9.03 Hz, 1H), 6.74 (d, *J* = 3.44 Hz, 1H), 2.27 (br. s., 3H). ^{13}C NMR (175 MHz, $(CD_3)_2SO$, 25 ºC, δ): 170.6, 145.5, 145.0, 143.3, 139.6, 136.0, 131.6, 129.6, 128.1, 124.4, 114.7, 112.7, 102.4, 22.2. Mass Spectrometry: HRMS (ESI-TOF) (m/z): calcd for $C_{14}H_{12}BrN_4O_2$ ([M + H]$^+$), 347.0138, found, 347.0142.

4-(1-(5-Nitropyridin-2-yl)-1*H*-benzo[*d*]imidazol-2-yl)thiazole (S18)

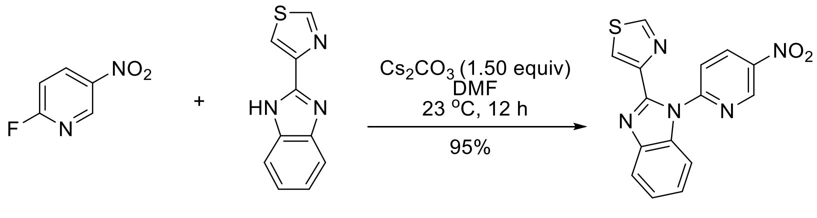

S18

Under N_2 atmosphere, a solution of 2-fluoro-5-nitropyridine (0.710 g, 5.00 mmol, 1.00 equiv) in DMF (5 mL, 1.00 M) was added to a mixture of 4-(1*H*-benzo[d]imidazol-2-yl)thiazole (1.51 g, 7.50 mmol, 1.50 equiv) and Cs_2CO_3 (2.44 g, 7.50 mmol, 1.50 equiv) in DMF (20.0 mL, 0.372 M) at 23 °C. The resulting mixture was stirred at 23 °C for 12 h. The reaction mixture was poured to LiCl solution (100 mL), extracted with EtOAc. The combined organic layers was dried ($MgSO_4$), filtered and concentrated *in vacuo*. The residue was purified by chromatography on silica gel, eluting with hexanes:EtOAc (2:1 to 1:1 (v/v)), to afford the title compound as a yellow solid (2.30 g, 7.11 mmol, 95% yield).

R_f = 0.34 (hexanes:EtOAc 1:1 (v/v)). NMR Spectroscopy: ^{1}H NMR (700 MHz, $CDCl_3$, 25 ºC, δ): 9.41 (d, *J* = 2.15 Hz, 1 H), 8.63 (d, *J* = 2.15 Hz, 1H), 8.60–8.56 (m, 1H), 8.28 (d, *J* = 2.15 Hz, 1H), 7.87 (d, *J* = 7.74 Hz, 1H), 7.55 (d, *J* = 7.74 Hz, 1H), 7.44–7.38 (m, 2H), 7.38–7.34 (m, 1H). ^{13}C NMR (175 MHz, $CDCl_3$, 25 ºC, δ): 154.8, 153.1, 146.6, 146.4, 145.2, 143.1, 143.0, 135.3, 133.4, 125.0, 124.4, 121.9, 121.5, 120.4, 111.3. Mass Spectrometry: HRMS (ESI-TOF) (m/z): calcd for $C_{15}H_{10}N_5O_2S$ ([M + H]$^+$), 324.0550, found, 324.0555.

***N*-Hydroxy-*N*-(6-(2-(thiazol-4-yl)-1*H*-benzo[d]imidazol-1-yl)pyridin-3-yl)acetamide (1t)**

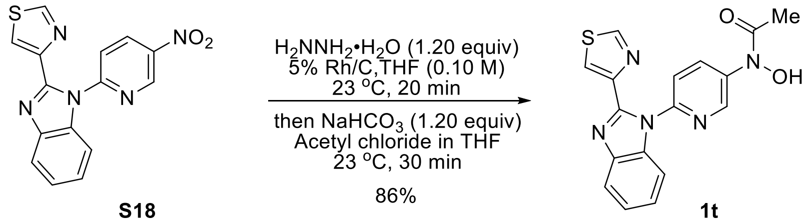

S18 **1t**

Under N_2 atmosphere, a suspension of 4-(1-(5-nitropyridin-2-yl)-1*H*-benzo[*d*]imidazol-2-yl)thiazole (323 mg, 1.00 mmol, 1.00 equiv) and 5% Rh/C (11.5 mg, 0.60 mol% Rh) in THF (10.0 mL, 0.100 M) was stirred at 23 °C. Hydrazine monohydrate (60.1 mg, 1.20 mmol, 1.20 equiv) was added dropwise. The reaction mixture was stirred at 23 °C for 20 min. $NaHCO_3$ (101 mg, 1.20 mmol, 1.20 equiv) was added, followed by dropwise addition of acetyl chloride (94.2 mg, 1.20

mmol, 1.20 equiv) in THF (10 mL, 0.120 M). The reaction mixture was stirred at 23 °C for 30 min and then filtered through a short pad of celite. The celite was washed with EtOAc. The combined organic solution was concentrated in vacuo. The residue was recrystalized from hexanes/EtOAc, to afford the title compound as a yellow solid (303 mg, 0.862 mmol, 86% yield).

R_f = 0.14 (hexanes:EtOAc 1:1 (v/v)). NMR Spectroscopy: ^{1}H NMR (700 MHz, $(CD_3)_2SO$, 25 ºC, δ): 11.04 (s, 1H), 9.01 (d, *J* = 1.72 Hz, 1H), 8.91 (br. s., 1H), 8.44 (d, *J* = 1.72 Hz, 1H), 8.26 (dd, *J* = 8.60, 2.58 Hz, 1H), 7.80 (d, *J* = 8.17 Hz, 1H), 7.51 (d, *J* = 8.60 Hz, 1H), 7.38–7.33 (m, 2H), 7.32–7.29 (m, 1H), 2.31 (s, 3H). ^{13}C NMR (175 MHz, $(CD_3)_2SO$, 25 ºC, δ): 171.0, 154.7, 146.6, 146.1, 145.1, 142.3, 139.4, 138.0, 135.8, 128.6, 123.8, 123.2, 122.6, 121.5, 119.4, 111.1, 22.4. Mass Spectrometry: HRMS (ESI-TOF) (m/z): calcd for $C_{17}H_{14}N_5O_2S$ ([M + H]$^+$), 352.0863, found, 352.0869.

2,6-Dichloro-9-(5-nitropyridin-2-yl)-9*H*-purine (S19)

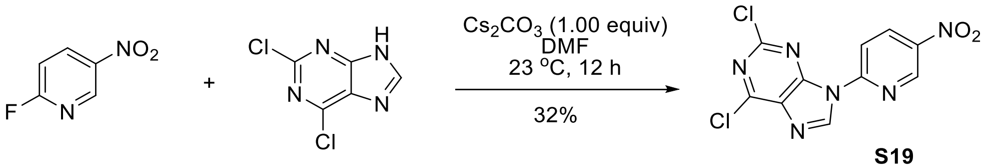

Under N_2 atmosphere, a solution of 2-fluoro-5-nitropyridine (0.78 g, 5.50 mmol, 1.10 equiv) in DMF (5 mL, 1.1 M) was added to a mixture of 2,6-dichloro-9*H*-purine (0.950 g, 5.00 mmol, 1.00 equiv) and Cs_2CO_3 (1.63 g, 5.00 mmol, 1.00 equiv) in DMF (20 mL, 0.25 M) at 23 °C. The reaction mixture was stirred at 23 °C for 12 h, poured to LiCl solution (100 mL), extracted with EtOAc. The combined organic layers was dried ($MgSO_4$), filtered and concentrated *in vacuo*. The residue was purified by chromatography on silica gel, eluting with hexanes:EtOAc (10:1 to 4:1 (v/v)), to afford the title compound as a yellow solid (0.500g, 1.61 mmol, 32% yield).

R_f = 0.50 (hexanes:EtOAc 4:1 (v/v)). NMR Spectroscopy: ^{1}H NMR (500 MHz, $CDCl_3$, 25 ºC, δ): 9.40 (d, *J* = 2.44 Hz, 1H), 9.30 (s, 1H), 8.95 (d, *J* = 8.85 Hz, 1H), 8.81 (dd, *J* = 9.00, 2.59 Hz, 1H). ^{13}C NMR (125 MHz, $CDCl_3$, 25 ºC, δ): 154.4, 153.3, 152.0, 150.7, 145.2, 143.8, 143.3, 135.4, 132.8, 115.0. Mass Spectrometry: HRMS (ESI-TOF) (m/z): calcd for $C_{10}H_5Cl_2N_6O_2$ ([M + H]$^+$), 310.9846, found, 310.9848.

***N*-(6-(2,6-Dichloro-9*H*-purin-9-yl)pyridin-3-yl)-*N*-hydroxyacetamide (1u)**

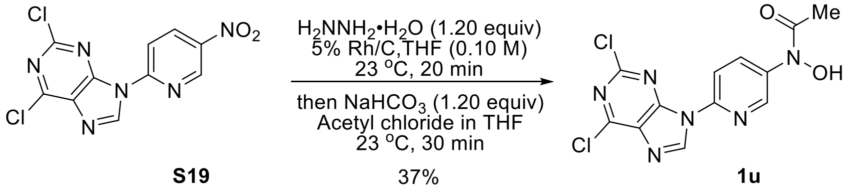

Under N_2 atmosphere, a suspension of 2,6-dichloro-9-(5-nitropyridin-2-yl)-9*H*-purine (270 mg, 0.870 mmol, 1.00 equiv) and 5% Rh/C (10.0 mg, 0.60 mol% Rh) in THF (8.7 mL, 0.100 M) was stirred at 23 °C. Hydrazine monohydrate (52.0 mg, 1.04 mmol, 1.20 equiv) was added dropwise.

The reaction mixture was stirred at 23 °C for 20 min. $NaHCO_3$ (87.4 g, 1.04 mmol, 1.2 equiv) was added, followed by dropwise addition of a solution of acetyl chloride (81.6 mg, 1.04 mmol, 1.20 equiv) in THF (8.7 mL, 0.120 M). The reaction mixture was stirred at 23 °C for 30 min and then filtered through a short pad of celite. The celite was washed with EtOAc. The combined organic solution was concentrated in vacuo. The residue was purified by chromatography on silica gel, eluting with hexanes:EtOAc (1:1 to EtOAc (v/v)), to afford the title compound as a yellow solid (110 mg, 0.324 mmol, 37% yield).

R_f = 0.13 (EtOAc). NMR Spectroscopy: ^{1}H NMR (500 MHz, $(CD_3)_2SO$, 25 ºC, δ): 11.07 (br. s., 1H), 9.34 (s, 1H), 8.95 (d, J = 2.44 Hz, 1H), 8.39 (dd, J = 9.00, 2.59 Hz, 1H), 8.28 (d, J = 8.85 Hz, 1H), 2.29 (s, 3H). ^{13}C NMR (125 MHz, $(CD_3)_2SO$, 25 ºC, δ): 171.0, 152.1, 151.7, 150.3, 145.7, 142.2, 139.5, 138.1, 131.8, 129.7, 115.7, 22.3. Mass Spectrometry: HRMS (ESI-TOF) (m/z): calcd for $C_{12}H_8C_{13}N_6O_2$ ([M + H]$^+$), 339.0159, found, 339.0172.

Ethyl (*E*)-3-(4-fluoro-3-(5-(hydroxy(methoxycarbonyl)amino)-2-methoxypyridin-3-yl)phenyl)acrylate (1v)

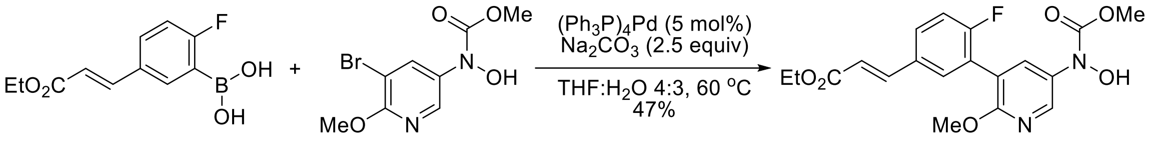

1a **1v**

Methyl (5-bromo-6-methoxypyridin-3-yl)(hydroxy)carbamate (**1a**) (0.300 g, 1.08 mmol, 1.00 equiv), (*E*)-(5-(3-ethoxy-3-oxoprop-1-en-1-yl)-2-fluorophenyl)boronic acid (0.361 g, 1.52 mmol, 1.40 equiv), Na_2CO_3 (0.287 g, 2.71 mmol, 2.5 equiv), and palladium-tetrakis(triphenylphosphine) (0.062 g, 0.05 mmol, 0.05 equiv) in THF:H_2O 4:3 (6.32 mL, 0.200 M), were degassed *via* three freeze-pump-thaw cycles. The resulting mixture was heated at 60 °C overnight and then allowed to cool to room temperature after which water was added (twice the volume of THF:H_2O 4:3 used). The mixture was then extracted with dichloromethane (twice the volume of THF:H_2O 4:3 used) and the organic extracts was dried with $MgSO_4$, filtered and concentrated in vacuo. The residue was purified by chromatography on silica gel, eluting with EtOAc:hexanes (3:8 to 1:1 (v/v)), to afford the pure cross-coupled product as a yellow solid (0.198 g, 0.51 mmol, 47% yield).

R_f = 0.36 (EtOAc:hexanes 1:1 (v/v)). NMR Spectroscopy: ^{1}H NMR (700 MHz, $(CD_3)_2SO$, 25 ºC, δ): 10.55 (s, 1H), 8.33 (d, J = 2.58 Hz, 1H), 7.90–7.79 (m, 3H), 7.68 (d, J = 15.92 Hz, 1H), 7.35 (t, J = 9.03 Hz, 1H), 6.67 (d, J = 16.35 Hz, 1H), 4.18 (q, J = 7.17 Hz, 2H), 3.86 (s, 3H), 3.73 (s, 3H), 1.25 (t, J = 7.10 Hz, 3H). ^{13}C NMR (175 MHz, $(CD_3)_2SO$, 25 ºC, δ): 166.2, 160.4 (d, J = 250.2 Hz), 157.7, 155.2, 143.0, 140.1, 133.9, 133.4, 131.9 (d, J = 3.5 Hz), 130.8 (d, J = 3.5 Hz), 130.3 (d, J = 8.6 Hz), 124.1 (d, J = 16.2 Hz), 118.6, 117.3, 116.3 (d, J = 22.7 Hz), 60.1, 53.8, 53.1, 14.2. ^{19}F NMR (376 MHz, $CDCl_3$, 25 ºC, δ): –111.3 (m). Mass Spectrometry: HRMS (ESI-TOF) (m/z): calcd for $C_{19}H_{20}FN_2O_6$ ([M + H]$^+$), 393.1356, found, 393.1351.

Methyl 4-((4-(5-(hydroxy(methoxycarbonyl)amino)-2-methoxypyridin-3-

yl)phenyl)ethynyl)benzoate (1w)

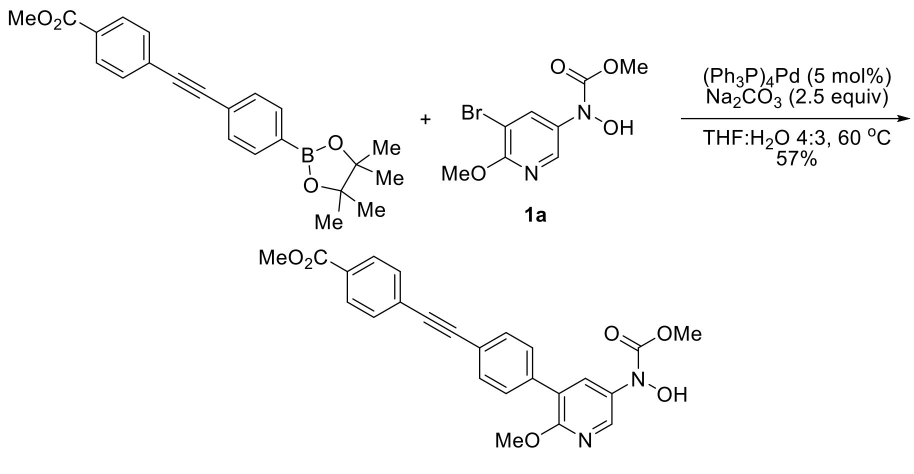

1w

Methyl (5-bromo-6-methoxypyridin-3-yl)(hydroxy)carbamate (**1a**) (0.300 g, 1.08 mmol, 1.00 equiv), methyl 4-((4-(4,4,5,5-tetramethyl-1,3,2-dioxaborolan-2-yl)phenyl)ethynyl)benzoate (0.152 g, 1.52 mmol, 1.40 equiv), Na_2CO_3 (0.287 g, 2.71 mmol, 2.5 equiv), THF:H_2O 4:3 (6.32 mL, 0.200 M), and palladium-tetrakis(triphenylphosphine) (0.062 g, 0.05 mmol, 0.05 equiv) were degassed via three freeze-pump-thaw cycles. The resulting mixture was heated at 60 °C overnight and then allowed to cool to room temperature after which water was added (twice the volume of THF:H_2O 4:3 used). The mixture was then extracted with dichloromethane (twice the volume of THF:H_2O 4:3 used) and the organic extracts was dried with $MgSO_4$, filtered and concentrated in vacuo. The residue was purified by chromatography on silica gel, eluting with EtOAc:hexanes (3:8 to 1:1 (v/v)), to afford the pure cross-coupled product as a white solid (0.199 g, 0.63 mmol, 57% yield).

R_f = 0.38 (EtOAc:hexanes 1:1 (v/v)). NMR Spectroscopy: ^{1}H NMR (700 MHz, $(CD_3)_2SO$, 25 ºC, δ): 10.40 (s, 1H), 8.29 (d, *J* = 2.58 Hz, 1H), 8.03–7.99 (m, 2H), 7.89 (d, *J* = 3.01 Hz, 1H), 7.73–7.70 (m, 2H), 7.68–7.63 (m, 4H), 3.93 (s, 3H), 3.89 (s, 3H), 3.75 (s, 3H). ^{13}C NMR (175 MHz, $(CD_3)_2SO$, 25 ºC, δ): 165.6, 157.3, 155.2, 139.3, 136.4, 133.9, 132.5, 131.7, 131.6, 129.5, 129.4, 127.0, 122.2, 121.1, 92.1, 89.2, 53.8, 53.1, 52.4. Mass Spectrometry: HRMS (ESI-TOF) (m/z): calcd for $C_{24}H_{20}N_2O_6$ ([M + H]$^+$), 435.1453, found, 435.1452.

(8*R*,9*S*,13*S*,14*S*)-13-Methyl-3-((5-nitropyridin-2-yl)oxy)-6,7,8,9,11,12,13,14,15,16-decahydro-17*H*-cyclopenta[*a*]phenanthren-17-one (S20)

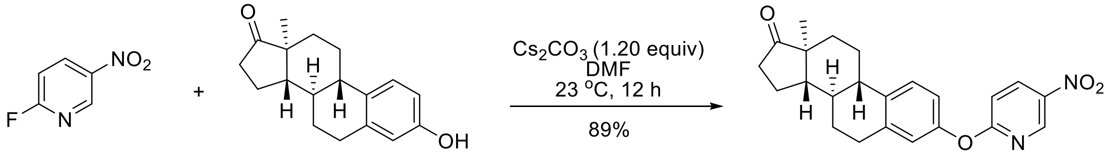

S20

Under N_2 atmosphere, a solution of 2-fluoro-5-nitropyridine (0.320 g, 2.22 mmol, 1.20 equiv) in

DMF (8.50 mL, 0.261 M) was added to a mixture of estrone (0.500 g, 1.85 mmol, 1.00 equiv) and Cs_2CO_3 (0.720 g, 2.22 mmol, 1.20 equiv) in DMF (10.0 mL, 0.185 M) at 23 °C. The resulting mixture was stirred at 23 °C for 12 h. The reaction mixture was poured to LiCl solution (100 mL), extracted with EtOAc. The combined organic layers was dried ($MgSO_4$), filtered and concentrated *in vacuo*. The residue was was recrystalized from hexanes/EtOAc, to afford the title compound as a light yellow solid (0.650g, 1.66 mmol, 89% yield).

R_f = 0.52 (hexanes:EtOAc 2:1 (v/v)). NMR Spectroscopy: ^{1}H NMR (700 MHz, $CDCl_3$, 25 ºC, δ): 9.06 (d, *J* = 2.58 Hz, 1H), 8.46 (dd, *J* = 9.03, 2.58 Hz, 1H), 7.36 (d, *J* = 8.60 Hz, 1H), 7.03 (d, *J* = 9.03 Hz, 1H), 6.93 (dd, *J* = 8.60, 2.58 Hz, 1H), 6.89 (d, *J* = 2.58 Hz, 1H), 2.97–2.92 (m, 2H), 2.52 (dd, *J* = 19.36, 8.60 Hz, 1H), 2.46–2.40 (m, 1H), 2.33 (td, *J* = 11.19, 4.30 Hz, 1H), 2.20–2.12 (m, 1H), 2.11–2.02 (m, 2H), 1.99 (dt, *J* = 12.91, 3.01 Hz, 1H), 1.68–1.45 (m, 6H), 0.93 (s, 3H). ^{13}C NMR (175 MHz, $CDCl_3$, 25 ºC, δ): 220.9, 167.2, 150.7, 145.3, 140.3, 138.8, 137.7, 135.0, 127.0, 121.5, 118.8, 111.5, 50.6, 48.1, 44.3, 38.1, 36.0, 31.7, 29.6, 26.5, 25.9, 21.7, 14.0. Mass Spectrometry: HRMS (ESI-TOF) (m/z): calcd for $C_{23}H_{25}N_2O_4$ ($[M + H]^+$), 393.1809, found, 393.1812.

_N_-Hydroxy-_N_-(6-(((8_R_,9_S_,13_S_,14_S_)-13-methyl-17-oxo-7,8,9,11,12,13,14,15,16,17-decahydro-6_H_-cyclopenta[_a_]phenanthren-3-yl)oxy)pyridin-3-yl)acetamide (1x)

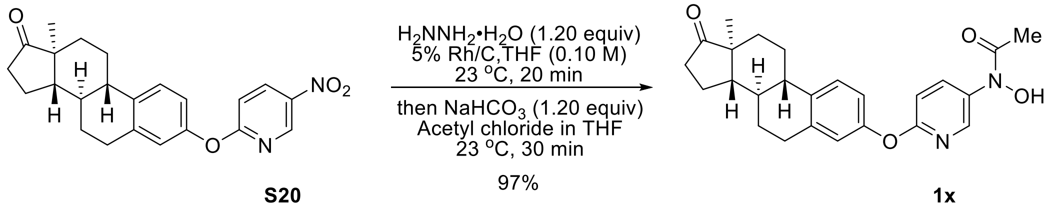

Under N_2 atmosphere, a suspension of (8*R*,9*S*,13*S*,14*S*)-13-methyl-3-((5-nitropyridin-2-yl)oxy)-6,7,8,9,11,12,13,14,15,16-decahydro-17*H*-cyclopenta[*a*]phenanthren-17-one (**S20**) (300 mg, 0.760 mmol, 1.00 equiv) and 5% Rh/C (8.70 mg, 0.60 mol% Rh) in THF (7.60 mL, 0.100 M) was stirred at 23 °C. Hydrazine monohydrate (45.9 mg, 0.910 mmol, 1.20 equiv) was added dropwise. The reaction mixture was stirred at 23 °C for 20 min. $NaHCO_3$ (76.4 mg, 0.91 mmol, 1.20 equiv) was added, followed by dropwise addition of acetyl chloride (71.4 mg, 0.91 mmol, 1.20 equiv) in THF (7.6 mL, 0.120 M). The reaction mixture was stirred at 23 °C for 30 min and then filtered through a short pad of celite. The celite was washed with EtOAc. The combined organic solution was concentrated in vacuo. The residue was recrystalized from hexanes/EtOAc, to afford the title compound as a yellow solid (310 mg, 0.737 mmol, 97% yield).

R_f = 0.13 (hexanes:EtOAc 1:1 (v/v)). NMR Spectroscopy: ^{1}H NMR (700 MHz, $(CD_3)_2SO$, 25 ºC, δ): 10.76 (s, 1H), 8.34 (d, *J* = 1.83 Hz, 1H), 8.00 (dd, *J* = 8.85, 2.44 Hz, 1H), 7.31 (d, *J* = 8.54 Hz, 1H), 7.01 (d, *J* = 8.85 Hz, 1H), 6.86 (dd, *J* = 8.24, 2.44 Hz, 1H), 6.81 (d, *J* = 2.44 Hz, 1H), 2.87–2.80 (m, 2H), 2.50 (dt, *J* = 3.66, 1.83 Hz, 3H), 2.36–2.48 (m, 2H), 2.26 (br. s., 1H), 2.20 (br. s., 3H), 2.07 (dd, *J* = 18.92, 8.85 Hz, 1H), 2.01–1.91 (m, 2H), 1.82–1.75 (m, 1H), 1.63–1.32 (m, 6H), 0.85 (s, 3H). ^{13}C NMR (175 MHz, $(CD_3)_2SO$, 25 ºC, δ): 219.7, 170.5, 160.0, 151.9, 139.7, 138.0,

135.9, 134.2, 133.0, 126.6, 120.8, 118.3, 110.9, 49.6, 47.3, 43.6, 37.6, 35.4, 31.4, 29.0, 25.9, 25.4, 21.9, 21.2, 13.5. Mass Spectrometry: HRMS (ESI-TOF) (m/z): calcd for $C_{25}H_{29}N_2O_4$ ([M + H]$^+$), 421.2122, found, 421.2125.

(6*R*,12*aR*)-6-(Benzo[*d*][1,3]dioxol-5-yl)-2-methyl-7-(5-nitropyridin-2-yl)-2,3,6,7,12,12a-hexahydropyrazino[1',2':1,6]pyrido[3,4-b]indole-1,4-dione (S21)

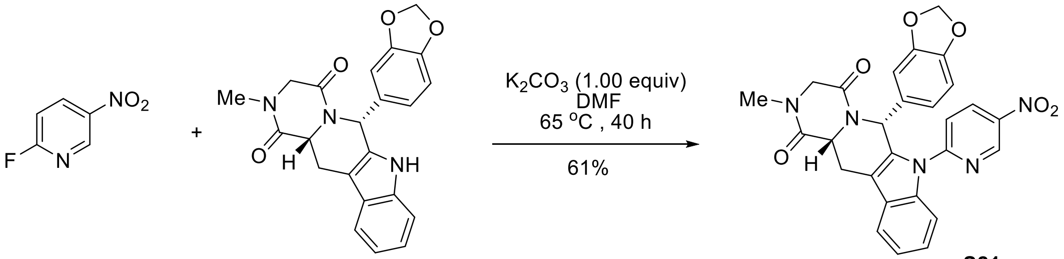

Under N_2 atmosphere, to a mixture of (6*R*,12*aR*)-6-(benzo[*d*][1,3]dioxol-5-yl)-2-methyl-2,3,6,7,12,12a-hexahydropyrazino[1',2':1,6]pyrido[3,4-b]indole-1,4-dione (1.00 g, 2.60 mmol, 1.00 equiv) and K_2CO_3 (0.36 g, 2.60 mmol, 1.00 equiv) in DMF (26.0 mL, 0.100 M) was added 2-fluoro-5-nitropyridine (0.550 g, 3.90 mmol, 1.50 equiv) and the reaction mixture was stirred at 23 °C for 40 h. The reaction mixture was poured to water (100 mL), extracted with EtOAc, washed with brine. The combined organic layers was dried ($MgSO_4$), filtered and concentrated *in vacuo*. The residue was purified by chromatography on silica gel, eluting with hexanes:EtOAc (1:1 to 1:3 (v/v)), to afford the title compound as a yellow solid (0.810 g, 1.58 mmol, 61% yield).

R_f = 0.51 (EtOAc). NMR Spectroscopy: ^{1}H NMR (700 MHz, DMSO, 25 °C, δ): 9.44 (d, *J* = 2.58 Hz, 1H), 8.53 (dd, *J* = 8.82, 2.80 Hz, 1H), 7.73–7.69 (m, 1H), 7.55–7.50 (m, 1H), 7.41 (d, *J* = 9.03 Hz, 1H), 7.35–7.28 (m, 2H), 7.00 (s, 1H), 6.52–6.44 (m, 3H), 5.80 (dd, *J* = 6.45, 1.29 Hz, 2H), 4.38 (dd, *J* = 11.62, 4.30 Hz, 1H), 4.16–4.10 (m, 1H), 3.93 (d, *J* = 17.21 Hz, 1H), 3.84 (dd, *J* = 16.13, 4.52 Hz, 1H), 3.27 (ddd, *J* = 16.35, 11.62, 1.29 Hz, 1H), 3.06–3.01 (m, 3H). ^{13}C NMR (175 MHz, $CDCl_3$, 25 °C, δ): 166.5, 166.4, 154.6, 147.6, 147.0, 145.6, 141.6, 136.6, 134.6, 134.0, 133.9, 127.6, 124.6, 122.8, 121.9, 119.6, 118.0, 113.3, 110.9, 108.2, 107.8, 101.2, 55.6, 55.4, 52.4, 33.8, 23.9. Mass Spectrometry: HRMS (ESI-TOF) (m/z): calcd for $C_{27}H_{22}N_5O_6$ ([M + H]$^+$), 512.1565, found, 512.1567.

***N*-(6-((6*R*,12a*R*)-6-(Benzo[*d*][1,3]dioxol-5-yl)-2-methyl-1,4-dioxo-1,3,4,6,12,12a-hexahydropyrazino[1',2':1,6]pyrido[3,4-b]indol-7(2*H*)-yl)pyridin-3-yl)-*N*-hydroxyacetamide**

(1y)

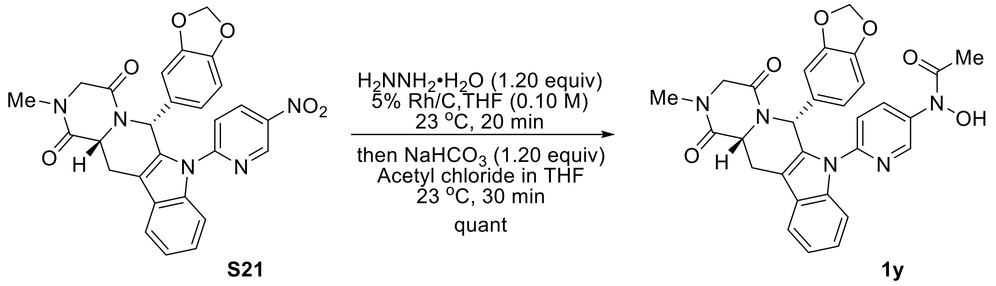

Under N_2 atmosphere, a suspension of (6*R*,12*aR*)-6-(benzo[*d*][1,3]dioxol-5-yl)-2-methyl-7-(5-nitropyridin-2-yl)-2,3,6,7,12,12a-hexahydropyrazino[1',2':1,6]pyrido[3,4-b]indole-1,4-dione (300 mg, 0.590 mmol, 1.00 equiv) and 5% Rh/C (6.80 mg, 0.60 mol% Rh) in THF (5.90 mL, 0.100 M) was stirred at 23 °C. Hydrazine monohydrate (35.4 mg, 0.710 mmol, 1.20 equiv) was added dropwise. The reaction mixture was stirred at 23 °C for 20 min. $NaHCO_3$ (59.6 mg, 0.710 mmol, 1.20 equiv) was added, followed by dropwise addition of a solution of acetyl chloride (55.7 mg, 0.710 mmol, 1.20 equiv) in THF (5.90 mL, 0.120 M). The reaction mixture was stirred at 23 °C for 30 min and then filtered through a short pad of celite. The celite was washed with EtOAc. The combined organic solution was concentrated in vacuo. The residue was recrystalized from hexanes/EtOAc, to afford the title compound as a white solid (320 mg, 0.59 mmol, quant yield).

R_f = 0.17 (EtOAc). NMR Spectroscopy: ^{1}H NMR (700 MHz, $(CD_3)_2SO$, 25 ºC, δ): 11.03 (br. s., 1H), 8.96 (br. s., 1H), 8.22 (d, *J* = 8.60 Hz, 1 H), 7.78–7.70 (m, 1H), 7.43–7.34 (m, 2H), 7.23–7.16 (m, 2H), 6.68 (br. s., 1H), 6.54–6.47 (m, 1H), 6.28 (d, *J* = 1.72 Hz, 1H), 6.19 (dd, *J* = 8.17, 1.72 Hz, 1H), 5.84 (dd, *J* = 12.91, 2.58 Hz, 2H), 4.53 (d, *J* = 11.62 Hz, 1H), 4.19 (d, *J* = 16.78 Hz, 1H), 3.92 (d, *J* = 17.21 Hz, 1H), 3.66 (d, *J* = 15.92 Hz, 1H), 2.97–2.87 (m, 3H), 2.44–2.43 (m, 1 H), 2.31 (br. s., 3H). ^{13}C NMR (175 MHz, $(CD_3)_2SO$, 25 ºC, δ): 166.9, 166.4, 146.8, 146.0, 137.0, 136.4, 135.4, 134.6, 126.0, 123.1, 120.9, 120.3, 119.5, 119.0, 110.3, 109.3, 107.5, 107.1, 100.9, 54.9, 54.2, 51.5, 32.8, 23.3, 22.3. Mass Spectrometry: HRMS (ESI-TOF) (m/z): calcd for $C_{29}H_{26}N_5O_6$ ([M + H]$^+$), 540.1878, found, 540.1881.

4-(1-(5-Nitropyrimidin-2-yl)-1*H*-benzo[*d*]imidazol-2-yl)thiazole (S22)

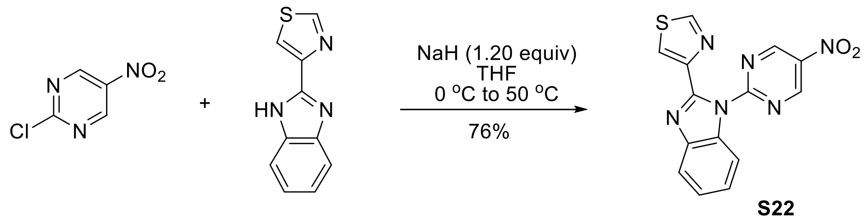

Under N_2 atmosphere, 4-(1*H*-benzo[*d*]imidazol-2-yl)thiazole (362 mg, 1.80 mmol, 1.20 equiv) was dissolved in THF (7.50 mL, 0.240 M) and stirred at 0 °C. NaH (72.0 mg, 1.80 mmol, 1.20 equiv, 60 % dispersion in mineral oil) was added in portionwise. After 30 min, a solution of 2-chloro-5-nitropyrimidine (239 mg, 1.50 mmol. 1.00 equiv) in THF (2.50 mL, 0.600 M) was added and then the reaction mixture was slowly warmed up to 50 °C for 12 h. The reaction mixture was poured to

water (100 mL), extracted with EtOAc, washed with brine. The combined organic layers was dried ($MgSO_4$), filtered and concentrated *in vacuo*. The residue was purified by chromatography on silica gel, eluting with hexanes:EtOAc (5:1 to 1:1 (v/v)), to afford the title compound as a yellow solid (369 mg, 1.14 mmol, 76% yield).

R_f = 0.33 (hexanes:EtOAc 2:1 (v/v)). NMR Spectroscopy: ^{1}H NMR (700 MHz, $CDCl_3$, 25 ºC, δ): 9.46 (s, 2H), 8.70 (d, *J* = 2.15 Hz, 1H), 8.20 (s, 1H), 8.18–8.13 (m, 1H), 7.89 (dd, *J* = 6.45, 2.58 Hz, 1H), 7.48–7.43 (m, 2H). ^{13}C NMR (175 MHz, $CDCl_3$, 25 ºC, δ): 159.0, 154.5, 154.4, 152.7, 147.6, 147.3, 143.0, 139.7, 134.1, 125.7, 125.2, 121.1, 120.6, 113.6. Mass Spectrometry: HRMS (ESI-TOF) (m/z): calcd for $C_{14}H_9N_6O_2S$ ([M + H]$^+$), 325.0502, found, 325.0506.

N-Hydroxy-N-(2-(2-(thiazol-4-yl)-1H-benzo[d]imidazol-1-yl)pyrimidin-5-yl)acetamide (3a)

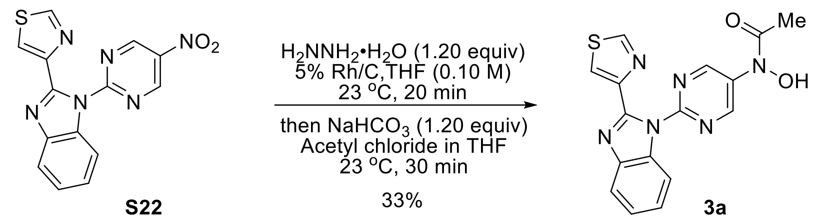

S22 3a

Under N_2 atmosphere, a suspension of 4-(1-(5-nitropyrimidin-2-yl)-1*H*-benzo[d]imidazol-2-yl)thiazole (200 mg, 0.620 mmol, 1.00 equiv) and 5% Rh/C (7.12 mg, 0.60 mol% Rh) in THF (6.20 mL, 0.100 M) was stirred at 23 °C. Hydrazine monohydrate (37.0 mg, 0.740 mmol, 1.20 equiv) was added dropwise. The reaction mixture was stirred at 23 °C for 20 min. $NaHCO_3$ (62.2 mg, 0.740 mmol, 1.20 equiv) was added, followed by dropwise addition of a solution of acetyl chloride (58.1 mg, 0.740 mmol, 1.20 equiv) in THF (6.20 mL, 0.120 M). The reaction mixture was stirred at 23 °C for 30 min and then poured to water (100 mL), extracted with EtOAc. The combined organic layers was dried ($MgSO_4$), filtered and concentrated *in vacuo*. The residue was purified by chromatography on silica gel, eluting with hexanes:EtOAc (1:1 (v/v)), to afford the title compound as a yellow gum (72 mg, 0.204 mmol, 33% yield).

R_f = 0.13 (EtOAc). NMR Spectroscopy: ^{1}H NMR (700 MHz, $(CD_3)_2SO$, 25 ºC, δ): 11.23 (s, 1H), 9.21 (s, 2H), 9.00 (br. s., 1H), 8.49 (d, *J* = 1.72 Hz, 1H), 7.81 (dd, *J* = 6.45, 1.72 Hz, 1H), 7.74–7.65 (m, 1H), 7.41–7.32 (m, 2H), 2.32 (s, 3H). ^{13}C NMR (175 MHz, $(CD_3)_2SO$, 25 ºC, δ): 171.5, 154.5, 151.2, 148.0, 146.8, 146.3, 142.3, 135.4, 134.8, 124.3, 123.6, 122.0, 119.6, 111.9, 22.1. Mass Spectrometry: HRMS (ESI-TOF) (m/z): calcd for $C_{16}H_{13}N_6O_2S$ ([M + H]$^+$), 353.0815, found, 353.0817.

5-Chloro-1-(5-nitropyrimidin-2-yl)-1H-indole (S23)

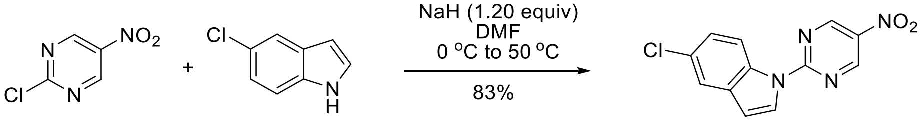

S23

Under N_2 atmosphere, 5-chloro-1*H*-indole (0.570 g, 3.76 mmol, 1.20 equiv) was dissolved in DMF

(10 mL, 0.376 M) and stirred at 0 °C. NaH (0.15g, 3.76 mmol, 1.20 equiv, 60 % dispersion in mineral oil) was added in portionwise. After 30 min, a solution of 2-chloro-5-nitropyrimidine (0.500 g, 3.13 mmol. 1.00 equiv) in DMF (5.6 mL, 0.559 M) was added and then the reaction mixture was slowly warmed up to 50 °C for 12 h. The reaction mixture was poured to a solution of LiCl (100 mL), extracted with EtOAc. The combined organic layers washed with brine, dried ($MgSO_4$), filtered and concentrated in vacuo. The residue was purified by chromatography on silica gel, eluting with hexanes:EtOAc (10:1 to 5:1 (v/v)), to afford the title compound as a yellow solid (0.710 g, 2.58 mmol, 83% yield).

R_f = 0.76 (hexanes:EtOAc 4:1 (v/v)). NMR Spectroscopy: ^{1}H NMR (700 MHz, $CDCl_3$, 25 ºC, δ): 9.43 (s, 2H), 8.69 (d, J = 8.60 Hz, 1H), 8.27 (d, J = 3.87 Hz, 1H), 7.58 (d, J = 1.72 Hz, 1H), 7.33 (dd, J = 8.82, 1.94 Hz, 1H), 6.73 (d, J = 3.01 Hz, 1H). ^{13}C NMR (175 MHz, $CDCl_3$, 25 ºC, δ): 158.7, 154.8, 138.2, 133.9, 133.3, 129.6, 127.5, 125.0, 121.0, 118.1, 109.7. Mass Spectrometry: HRMS (ESI-TOF) (m/z): calcd for $C_{12}H_{11}ClN_5O_2$ ($[M + NH_4]^+$), 292.0596, found, 292.0599.

_N_-(2-(5-Chloro-1_H_-indol-1-yl)pyrimidin-5-yl)-_N_-hydroxyacetamide (3b)

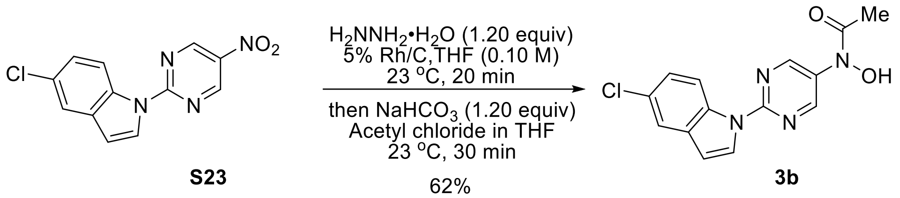

Under N_2 atmosphere, a suspension of 5-chloro-1-(5-nitropyrimidin-2-yl)-1*H*-indole (300 mg, 1.09 mmol, 1.00 equiv) and 5% Rh/C (12.6 mg, 0.60 mol% Rh) in THF (10.9 mL, 0.100 M) was stirred at 23 °C. Hydrazine monohydrate (65.6 mg, 1.31 mmol, 1.20 equiv) was added dropwise. The reaction mixture was stirred at 23 °C for 20 min. $NaHCO_3$ (110 mg, 1.31 mmol, 1.20 equiv) was added, followed by dropwise addition of a solution of acetyl chloride (103 mg, 1.31 mmol, 1.20 equiv) in THF (10.9 mL, 0.120 M). The reaction mixture was stirred at 23 °C for 30 min and then poured to water (100 mL), extracted with EtOAc. The combined organic layers was dried ($MgSO_4$), filtered and concentrated *in vacuo*. The residue was recrystalized from hexanes/EtOAc, to afford the title compound as a yellow solid (205 mg, 0.677 mmol, 62% yield).

R_f = 0.14 (hexanes:EtOAc 2:1 (v/v)). NMR Spectroscopy: ^{1}H NMR (700 MHz, $(CD_3)_2SO$, 25 ºC, δ): 11.10 (s, 1H), 9.13 (s, 2H), 8.69 (d, J = 9.03 Hz, 1H), 8.30 (d, J = 3.87 Hz, 1H), 7.72 (d, J = 2.15 Hz, 1H), 7.34 (dd, J = 8.60, 2.15Hz, 1H), 6.78 (d, J = 3.87 Hz, 1H), 2.28 (s, 3H). ^{13}C NMR (175 MHz, $(CD_3)_2SO$, 25 ºC, δ): 171.0, 152.5, 148.9, 133.1, 133.0, 132.0, 127.3, 126.4, 123.4, 120.2, 117.0, 106.1, 21.8. Mass Spectrometry: HRMS (ESI-TOF) (m/z): calcd for $C_{14}H_{12}ClN_4O_2$ ($[M + H]^+$), 303.0643, found, 303.0645.

_N_-Hydroxy-_N_-(2-methoxypyrimidin-5-yl)acetamide (3c)

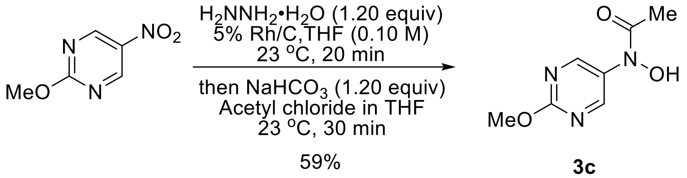

3c

Under N_2 atmosphere, a suspension of 2-methoxy-5-nitropyrimidine (180 mg, 1.16 mmol, 1.00 equiv) and 5% Rh/C (13.8 mg, 0.60 mol% Rh) in THF (11.6 mL, 0.100 M) was stirred at 23 °C. Hydrazine monohydrate (69.7 mg, 1.39 mmol, 1.20 equiv) was added dropwise. The reaction mixture was stirred at 23 °C for 20 min. $NaHCO_3$ (117 mg, 1.39 mmol, 1.20 equiv) was added, followed by dropwise addition of a solution of acetyl chloride (109 mg, 1.39 mmol, 1.20 equiv) in THF (11.6 mL, 0.120 M). The reaction mixture was stirred at 23 °C for 30 min and then filtered through a short pad of celite. The celite was washed with EtOAc. The combined organic solution was concentrated in vacuo. . The residue was purified by chromatography on silica gel, eluting with hexanes:EtOAc (2:1 to 0:1 (v/v)), to afford the title compound as a gray gum (125 mg, 0.682 mmol, 59% yield).

R_f = 0.33 (EtOAc). NMR Spectroscopy: ^1H NMR (700 MHz, $(CD_3)_2SO$, 25 °C, δ): 10.93 (br. s, 1H), 8.81 (s, 2H), 3.91 (s, 3H), 2.22 (br. s., 3H). ^{13}C NMR (175 MHz, $(CD_3)_2SO$, 25 °C, δ): 170.7, 161.8, 151.0, 131.8, 54.9, 21.6. Mass Spectrometry: HRMS (ESI-TOF) (m/z): calcd for $C_7H_{10}N_3O_3$ ($[M + H]^+$), 184.0717, found, 184.0718.

2-(4-Chloro-3,5-dimethylphenoxy)-5-nitropyridine (S24)

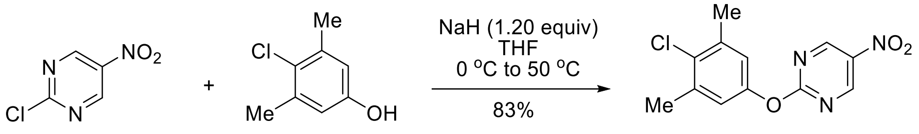

S24

Under N_2 atmosphere, 4-chloro-3,5-dimethylphenol (240 mg, 1.52 mmol, 1.20 equiv) was dissolved in THF (8.40 mL, 0.181 M) and stirred at 0 °C. NaH (60.8 mg, 1.52 mmol, 1.20 equiv, 60 % dispersion in mineral oil) was added in portionwise. After 30 min, a solution of 2-chloro-5-nitropyrimidine (200 mg, 1.24 mmol. 1.00 equiv) in THF (4.00 mL, 0.310 M) was added and then the reaction mixture was slowly warmed to 50 °C for 12h. The reaction mixture was poured to water (100 mL), extracted with EtOAc, washed with brine. The combined organic layers was dried ($MgSO_4$), filtered and concentrated *in vacuo*. The residue was purified by chromatography on silica gel, eluting with hexanes:EtOAc (20:1 to 1:1 (v/v)), to afford the title compound as a yellow solid (288 mg, 1.03 mmol, 83% yield).

R_f = 0.61 (hexanes:EtOAc 10:1 (v/v)). NMR Spectroscopy: ^1H NMR (500 MHz, $CDCl_3$, 25 °C, δ): 9.32 (s, 2H), 6.94 (s, 2H), 2.41 (s, 6H). ^{13}C NMR (125 MHz, $CDCl_3$, 25 °C, δ): 167.1, 156.5, 149.9, 139.1, 138.4, 132.7, 121.1, 21.1. Mass Spectrometry: HRMS (ESI-TOF) (m/z): calcd for $C_{12}H_{11}ClN_3O_3$ ($[M + H]^+$), 280.0483, found, 280.0483.

N-(2-(4-Chloro-3,5-dimethylphenoxy)pyrimidin-5-yl)-N-hydroxyacetamide (3d)

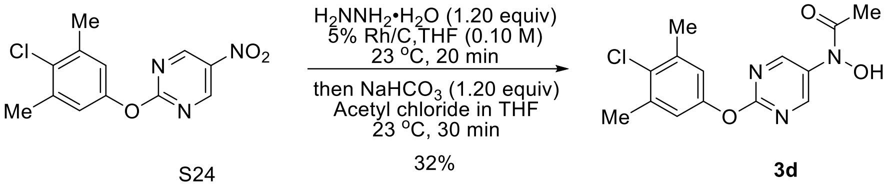

Under N_2 atmosphere, a suspension of 2-(4-chloro-3,5-dimethylphenoxy)-5-nitropyrimidine (140 mg, 0.500 mmol, 1.00 equiv) and 5% Rh/C (5.70 mg, 0.60 mol% Rh) in THF (5.00 mL, 0.100 M) was stirred at 23 °C. Hydrazine monohydrate (30.0 mg, 0.60 mmol, 1.20 equiv) was added dropwise. The reaction mixture was stirred at 23 °C for 20 min. $NaHCO_3$ (50.4 mg, 0.600 mmol, 1.20 equiv) was added, followed by dropwise addition of a solution of acetyl chloride (47.1 mg, 0.600 mmol, 1.20 equiv) in THF (5.00 mL, 0.120 M). The reaction mixture was stirred at 23 °C for 30 min and then poured to water (100 mL), extracted with EtOAc. The combined organic layers was dried ($MgSO_4$), filtered and concentrated *in vacuo*. The residue was purified by chromatography on silica gel, eluting with hexanes:EtOAc (1:1 to 0 :1(v/v)), to afford the title compound as a yellow solid (49.1 mg, 0.160 mmol, 32% yield).

R_f = 0.53 (EtOAc). NMR Spectroscopy: ^{1}H NMR (700 MHz, $(CD_3)_2SO$, 25 ºC, δ): 10.99 (br. s., H), 8.86 (s, 2H), 7.08 (s, 2H), 2.33 (s, 6H), 2.23 (br. s., 3H). ^{13}C NMR (175 MHz, $(CD_3)_2SO$, 25 ºC, δ): 170.9, 161.0, 151.0, 137.1, 133.0, 129.9, 121.6, 21.6, 20.3. Mass Spectrometry: HRMS (ESI-TOF) (m/z): calcd for $C_{14}H_{15}ClN_3O_3$ ([M + H]$^+$), 308.0802, found, 308.0797.

(8*R*,9*S*,13*S*,14*S*)-13-Methyl-3-((5-nitropyrimidin-2-yl)oxy)-6,7,8,9,11,12,13,14,15,16-decahydro-17*H*-Cyclopenta[a]phenanthren-17-one (S25)

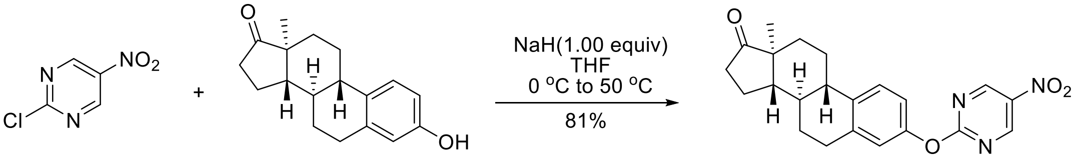

S25

Under N_2 atmosphere, estrone (200 mg, 0.740 mmol, 1.00 equiv) was dissolved in THF (3.70 mL, 0.200 M) and stirred at 0 °C. NaH (29.6 mg, 0.740 mmol, 1.00 equiv, 60 % dispersion in mineral oil) was added in portionwise. After 30 min, a solution of 2-chloro-5-nitropyrimidine (130 mg, 0.81 mmol. 1.10 equiv) in THF (1.30 mL, 0.623 M) was added and then the reaction mixture was slowly warmed to 50 °C for 12 h. The reaction mixture was poured to water (100 mL), extracted with EtOAc, washed with brine. The combined organic layers was dried ($MgSO_4$), filtered and concentrated *in vacuo*. The residue was purified by chromatography on silica gel, eluting with hexanes:EtOAc (5:1 to 2:1 (v/v)), to afford the title compound as a yellow solid (235 mg, 0.597 mmol, 81% yield).

R_f = 0.33 (hexanes:EtOAc 4:1 (v/v)). NMR Spectroscopy: ^{1}H NMR (700 MHz, $CDCl_3$, 25 ºC, δ): 9.32 (s, 2H), 7.38 (d, J = 8.60 Hz, 1H), 6.97 (dd, J = 8.60, 2.15 Hz, 1H), 6.92 (d, J = 2.15 Hz, 1H),

2.97–2.91 (m, 2H), 2.51 (dd, J = 19.36, 8.60 Hz, 1H), 2.46–2.40 (m, 1H), 2.34 (td, J = 11.08, 4.09 Hz, 1H), 2.19–2.11 (m, 1H), 2.10–2.02 (m, 2H), 1.98 (dt, J = 12.80, 2.85 Hz, 1H), 1.68–1.57 (m, 3H), 1.57–1.46 (m, 3 H), 0.92 (s, 3H). ^{13}C NMR (175 MHz, $CDCl_3$, 25 ºC, δ): 220.8, 167.3, 156.4, 150.2, 138.9, 138.3, 127.0, 121.2, 118.5, 50.5, 48.0, 44.3, 38.0, 35.9, 31.6, 29.6, 26.4, 25.8, 21.7, 13.9. Mass Spectrometry: HRMS (ESI-TOF) (m/z): calcd for $C_{22}H_{24}N_3O_4$ ([M + H]$^+$), 394.1761, found, 394.1758.

N-Hydroxy-*N*-(2-(((8*R*,9*S*,13*S*,14*S*)-13-methyl-17-oxo-7,8,9,11,12,13,14,15,16,17-decahydro-6*H*-cyclopenta[*a*]phenanthren-3-yl)oxy)pyrimidin-5-yl)acetamide (3e)

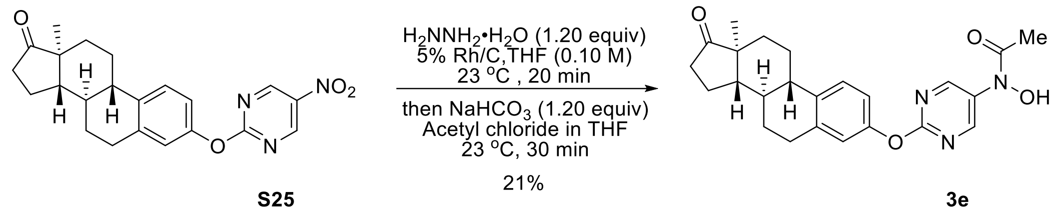

Under N_2 atmosphere, a suspension of (8*R*,9*S*,13*S*,14*S*)-13-methyl-3-((5-nitropyrimidin-2-yl)oxy)-6,7,8,9,11,12,13,14,15,16-decahydro-17*H*-cyclopenta[*a*]phenanthren-17-one (170 mg, 0.430 mmol, 1.00 equiv) and 5% Rh/C (4.90 mg, 0.60 mol% Rh) in THF (4.30 mL, 0.100 M) was stirred at 23 °C. Hydrazine monohydrate (26.0 mg, 0.52 mmol, 1.20 equiv) was added dropwise. The reaction mixture was stirred at 23 °C for 20 min. $NaHCO_3$ (43.7 mg, 0.520 mmol, 1.20 equiv) was added, followed by dropwise addition of acetyl chloride (40.8 mg, 0.520 mmol, 1.20 equiv) in THF (4.30 mL, 0.120 M). The reaction mixture was stirred at 23 °C for 30 min and then poured to water (100 mL), extracted with EtOAc. The combined organic layers was dried ($MgSO_4$), filtered and concentrated *in vacuo*. The residue was purified by chromatography on silica gel, eluting with hexanes:EtOAc (2:1 (v/v)), to afford the title compound as a yellow solid (38 mg, 0.0902 mmol, 21% yield).

R_f = 0.50 (EtOAc). NMR Spectroscopy: ^{1}H NMR (700 MHz, $(CD_3)_2SO$, 25 ºC, δ):10.98 (br. s., 1H), 8.83 (s, 1H), 7.33 (d, J = 8.17 Hz, 1H), 6.93 (dd, J = 8.60, 2.58 Hz, 1H), 6.89 (d, J = 2.58 Hz, 1H), 2.88–2.84 (m, 2H) 2.45 (dd, J = 19.15, 8.39 Hz, 1H), 2.42–2.38 (m, 1H), 2.30–2.25 (m, 1H), 2.23 (br. s., 3H), 2.11–2.05 (m, 1H), 2.00–1.93 (m, 2H) 1.80–1.76 (m, 1H), 1.62–1.36 (m, 6H), 0.86 (s, 3H). ^{13}C NMR (175 MHz, $(CD_3)_2SO$, δ): 219.7, 170.8, 161.3, 150.8, 138.0, 136.6, 132.8, 126.6, 121.2, 118.7, 49.6, 47.3, 43.6, 37.6, 35.4, 31.4, 29.0, 25.9, 25.4, 21.6, 21.2, 13.5. Mass Spectrometry: HRMS (ESI-TOF) (m/z): calcd for $C_{24}H_{28}N_3O_4$ ([M + H]$^+$), 422.2074, found, 422.2074.

Methyl (5-bromo-6-methoxy-2-(trifluoromethoxy)pyridin-3-yl)carbamate (2a)

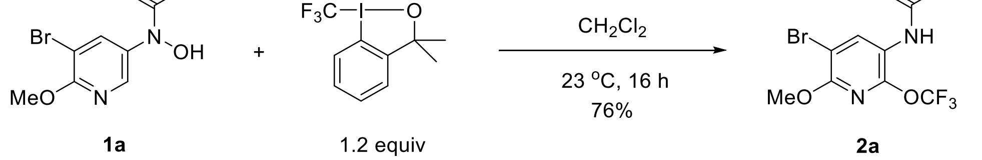

A solution of methyl (5-bromo-6-methoxypyridin-3-yl)(hydroxy)carbamate (**1a**) (50.0 mg, 0.180 mmol) and Togni reagent I (71.5 mg, 0.217 mmol, 1.20 equiv) in CH_2Cl_2 (1.80 mL, 0.100 M) was stirred at 23 °C under N_2 atmosphere for 16 h. The reaction mixture was purified by preparative TLC (thickness: 1 mm) using hexanes:EtOAc (19:1 (v/v)) for development (prep TLC was developed three times). The purification afforded the title compound as a white solid (47.3 mg, 0.137 mmol, 76% yield).

R_f = 0.69 (EtOAc:hexanes 1:4 (v/v)). NMR Spectroscopy: ^{1}H NMR (500 MHz, $CDCl_3$, 25 ºC, δ): 8.65 (br. s, 1H), 6.62 (br. s, 1H), 3.93 (s, 3H), 3.80 (s, 3H). ^{13}C NMR (125 MHz, $CDCl_3$, 25 ºC, δ): 153.8, 153.6, 141.9, 135.6, 120.1 (q, J = 261.6 Hz), 117.3, 102.6, 55.1, 53.0. ^{19}F NMR (376 MHz, $CDCl_3$, 25 ºC, δ): –56.6 (s). Mass Spectrometry: HRMS (ESI-TOF) (m/z): calcd for $C_9H_9BrF_3N_2O_4$ ([M + H]$^+$), 344.9692, found, 344.9705.

Methyl (5-iodo-6-methoxy-2-(trifluoromethoxy)pyridin-3-yl)carbamate (2b)

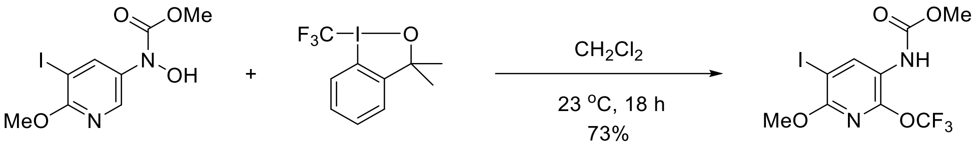

1b 1.2 equiv **2b**

A solution of methyl hydroxy(5-iodo-6-methoxypyridin-3-yl)carbamate (**1b**) (50.0 mg, 0.154 mmol) and Togni reagent I (61.1 mg, 0.185 mmol, 1.20 equiv) in CH_2Cl_2 (1.54 mL, 0.100 M) was stirred at 23 °C under N_2 atmosphere for 18 h. The reaction mixture was purified by preparative TLC (thickness: 1 mm) using hexanes:EtOAc (97:3 (v/v)) for development (prep TLC was developed six times). The purification afforded the title compound as a white solid (44.2 mg, 0.113 mmol, 73% yield).

R_f = 0.53 (EtOAc:hexanes 1:9 (v/v)). NMR Spectroscopy: ^{1}H NMR (500 MHz, $CDCl_3$, 25 ºC, δ): 8.81 (br. s, 1H), 6.58 (br. s., 1H), 3.91 (s, 3H), 3.80 (s, 3H). ^{13}C NMR (125 MHz, $CDCl_3$, 25 ºC, δ): 155.8, 153.8, 143.4, 141.6, 120.1 (q, J = 261.8 Hz), 117.4, 73.9, 55.4, 52.9. ^{19}F NMR (376 MHz, $CDCl_3$, 25 ºC, δ): –56.5 (s). Mass Spectrometry: HRMS (ESI-TOF) (m/z): calcd for $C_9H_9F_3IN_2O_4$ ([M + H]$^+$), 392.9554, found, 392.9556.

Methyl (6-chloro-4-methyl-2-(trifluoromethoxy)pyridin-3-yl)carbamate (2c)

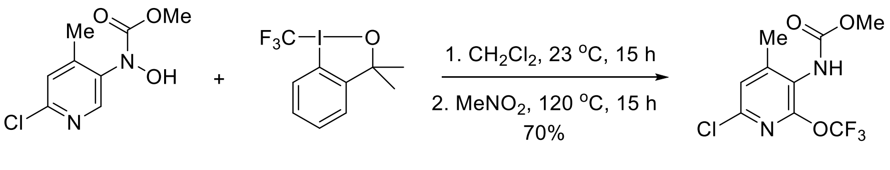

1c 1.2 equiv **2c**, 70%

A solution of methyl (6-chloro-4-methylpyridin-3-yl)(hydroxy)carbamate (**1c**) (108 mg, 0.500 mmol) and Togni reagent I (198 mg, 0.600 mmol, 1.20 equiv) in CH_2Cl_2 (5.00 mL, 0.100 M) was stirred at 23 °C under N_2 atmosphere for 15 h. The reaction mixture was concentrated *in vacuo*.

The residue was dissolved in $MeNO_2$ (5.00 mL, 0.100 M) and the reaction mixture was stirred at 120 °C for 15 h. The reaction mixture was concentrated *in vacuo* and purified by chromatography on silica gel, eluting with hexanes:EtOAc (8:1 to 4:1 (v/v)), to afford **2c** (99.0 mg, 0.348 mmol, 70% yield).

Data for **2c**: white solid; R_f = 0.45 (EtOAc:hexanes 1:4 (v/v)). NMR Spectroscopy: ^{1}H NMR (700 MHz, $CDCl_3$, 25 ºC, δ): 8.44 (d, J = 8.53 Hz, 1H), 7.38 (d, J = 8.53 Hz, 1H), 6.85 (br. s, 1H), 3.83 (s, 3H). ^{13}C NMR (175 MHz, $CDCl_3$, 25 ºC, δ): 154.4, 151.6, 150.6, 145.5, 124.1, 120.0 (q, J = 261.9 Hz), 119.9, 53.4 (d, J = 7.51 Hz), 18.4. ^{19}F NMR (376 MHz, DMSO, 90 ºC, δ): –57.3(s). Mass Spectrometry: HRMS (ESI-TOF) (m/z): calcd for $C_9H_9ClF_3N_2O_3$ ($[M + H]^+$), 285.0248, found, 285.0249.

Methyl (6-bromo-2-(trifluoromethoxy)pyridin-3-yl)carbamate (2d) and methyl (6-bromo-4-(trifluoromethoxy)pyridin-3-yl)carbamate (2d-II)

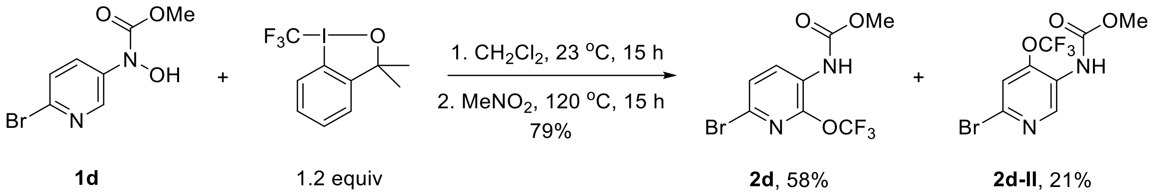

A solution of methyl (6-bromopyridin-3-yl)(hydroxy)carbamate (**1d**) (124 mg, 0.226 mmol) and Togni reagent I (198 mg, 0.600 mmol, 1.20 equiv) in CH_2Cl_2 (5.00 mL, 0.100 M) was stirred at 23 °C under N_2 atmosphere for 15 h. The reaction mixture was concentrated *in vacuo*. The residue was dissolved in $MeNO_2$ (5.00 mL, 0.100 M) and the reaction mixture was stirred at 120 °C for 15 h. The reaction mixture was concentrated *in vacuo* and purified by chromatography on silica gel, eluting with hexanes:EtOAc (19:1 to 9:1 (v/v)), to afford **2d** (91 mg, 0.289 mmol, 58% yield) and **2d-II** (33 mg, 0.105 mmol, 21% yield).

Data for **2d**: white solid; R_f = 0.57 (EtOAc:hexanes 1:4 (v/v)). NMR Spectroscopy: ^{1}H NMR (400 MHz, $CDCl_3$, 25 ºC, δ): 8.44 (d, J = 8.53 Hz, 1H), 7.38 (d, J = 8.53 Hz, 1H), 6.85 (br. s, 1H), 3.83 (s, 3H). ^{13}C NMR (175 MHz, $CDCl_3$, 25 ºC, δ): 153.4, 143.8, 130.3, 129.6, 126.6, 123.5, 120.0 (q, J = 265.2 Hz), 53.2. ^{19}F NMR (376 MHz, $CDCl_3$, 25 ºC, δ): –56.7 (s). Mass Spectrometry: HRMS (ESI-TOF) (m/z): calcd for $C_8H_7BrF_3N_2O_3$ ($[M + H]^+$), 314.9587, found, 314.9585.

Data for **2d-II**: white solid; R_f = 0.45 (EtOAc:hexanes 1:4 (v/v)) NMR Spectroscopy: ^{1}H NMR (400 MHz, $CDCl_3$, 25 ºC, δ): 9.23 (br. s, 1H), 7.35 (q, J = 2.01 Hz, 1H), 6.77 (br. s, 1H), 3.84 (s, 3H). ^{13}C NMR (175 MHz, $CDCl_3$, 25 ºC, δ): 153.1, 144.8, 142.7, 134.8, 126.5, 120.2 (q, J = 265.2 Hz), 116.9, 53.4. ^{19}F NMR (376 MHz, $CDCl_3$, 25 ºC, δ): –57.9 (s). Mass Spectrometry: HRMS (ESI-TOF) (m/z): calcd for $C_8H_7BrF_3N_2O_3$ ($[M + H]^+$), 314.9587, found, 314.9586.

Methyl (6-fluoro-2-(trifluoromethoxy)pyridin-3-yl)carbamate (2e) and methyl (6-fluoro-4-

(trifluoromethoxy)pyridin-3-yl)carbamate (2e-II)

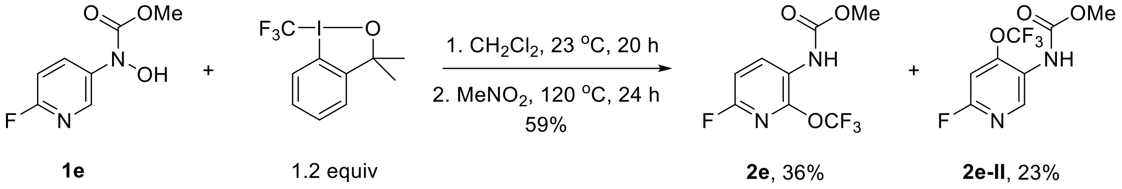

A solution of methyl (6-fluoropyridin-3-yl)(hydroxy)carbamate (**1e**) (93.1 mg, 0.500 mmol) and Togni reagent I (198 mg, 0.600 mmol, 1.20 equiv) in CH_2Cl_2 (5.00 mL, 0.100 M) was stirred at 23 °C under N_2 atmosphere for 20 h. The reaction mixture was concentrated *in vacuo*. The residue was dissolved in $MeNO_2$ (5.00 mL, 0.100 M) and the reaction mixture was stirred at 120 °C for 24 h. The crude residue was purified by flash chromatography eluting hexanes and then EtOAc:hexanes (1:4 (v/v)). The purification afforded a 1.6:1 mixture of **2e** and **2e-II**, which was further purified by preparative TLC (thickness: 1 mm) using hexanes:EtOAc (19:1 (v/v)) for development (prep TLC was developed five times). The purification afforded **2e** (45.8 mg, 0.180 mmol, 36% yield) and **2e-II** (28.7 mg, 0.113 mmol, 23% yield).

Data for **2e**: white solid; R_f = 0.48 (EtOAc:hexanes 1:4 (v/v)). NMR Spectroscopy: ^{1}H NMR (400 MHz, $CDCl_3$, 25 ºC, δ): 8.64 (br. s, 1H), 6.87 (dd, J = 8.66, 3.14 Hz, 1H), 6.79 (br. s, 1H), 3.83 (s, 3H). ^{13}C NMR (125 MHz, $CDCl_3$, 25 ºC, δ): 155.6 (d, J = 242.1 Hz), 153.7, 142.2, 133.5, 121.4, 120.0 (q, J = 262.7 Hz), 107.1 (d, J = 35.6 Hz), 53.1. ^{19}F NMR (376 MHz, $CDCl_3$, 25 ºC, δ): –56.8 (s), –75.7 (s). Mass Spectrometry: HRMS (ESI-TOF) (m/z): calcd for $C_8H_7F_4N_2O_3$ ($[M + H]^+$), 255.0387, found, 255.0387.

Data for **2e-II**: off-white solid; R_f = 0.44 (EtOAc:hexanes 1:4 (v/v)). NMR Spectroscopy: ^{1}H NMR (400 MHz, $CDCl_3$, 25 ºC, δ): 8.98 (br. s, 1H), 6.83 (quin, J = 2.13 Hz, 1H), 6.69 (br. s, 1H), 3.83 (s, 3H). ^{13}C NMR (125 MHz, $CDCl_3$, 25 ºC, δ): 159.2 (d, J = 234.5 Hz), 153.5, 147.5, 140.3, 124.6, 120.2 (q, J = 262.2 Hz), 99.2 (d, J = 45.2 Hz), 53.3. ^{19}F NMR (376 MHz, $CDCl_3$, 25 ºC, δ): –58.2 (s), –69.6 (s). Mass Spectrometry: HRMS (ESI-TOF) (m/z): calcd for $C_8H_7F_4N_2O_3$ ($[M + H]^+$), 255.0387, found, 255.0390.

Methyl (5-bromo-6-chloro-2-(trifluoromethoxy)pyridin-3-yl)carbamate (2f) and methyl (5-Bromo-6-chloro-4-(trifluoromethoxy)pyridin-3-yl)carbamate (2f-II)

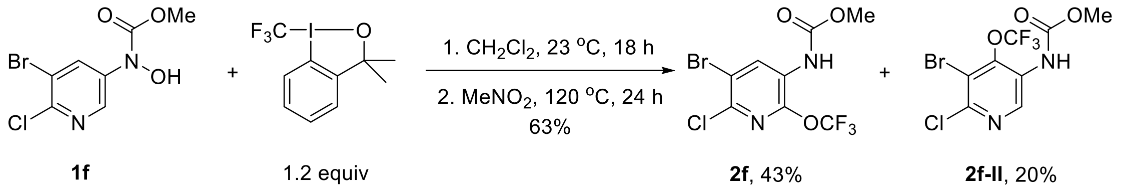

A solution of methyl (5-bromo-6-chloropyridin-3-yl)(hydroxy)carbamate (**1f**) (141 mg, 0.500 mmol) and Togni reagent I (198 mg, 0.600 mmol, 1.20 equiv) in CH_2Cl_2 (5.00 mL, 0.100 M) was stirred at 23 °C under N_2 atmosphere for 18 h. The reaction mixture was concentrated *in vacuo*.

The residue was dissolved in $MeNO_2$ (5.00 mL, 0.100 M) and the reaction mixture was stirred at 120 °C for 24 h. The crude residue was purified by flash chromatography eluting with EtOAc:hexanes (1:19 to 1:4 (v/v)), to afford **2f** (75.6 mg, 0.216 mmol, 43% yield) and **2f-II** (34.6 mg, 0.0990 mmol, 20% yield).

Data for **2f**: white solid; R_f = 0.52 (EtOAc:hexanes 1:4 (v/v)). NMR Spectroscopy: ^{1}H NMR (700 MHz, $CDCl_3$, 25 ºC, δ): 8.86 (s, 1H), 6.85 (br. s, 1H), 3.84 (s, 3H). ^{13}C NMR (175 MHz, $CDCl_3$, 25 ºC, δ): 153.2, 142.3, 139.7, 133.3, 123.7, 119.9 (q, J = 263.4 Hz), 117.6, 53.4. ^{19}F NMR (376 MHz, $CDCl_3$, 25 ºC, δ): –56.8 (s). Mass Spectrometry: HRMS (ESI-TOF) (m/z): calcd for $C_8H_6BrClF_3N_2O_3$ ([M + H]$^+$), 348.9197, found, 348.9196.

Data for **2f-II**: slightly yellow solid; R_f = 0.43 (EtOAc:hexanes 1:4 (v/v)). NMR Spectroscopy: ^{1}H NMR (500 MHz, $CDCl_3$, 25 ºC, δ): 9.19 (br. s, 1H), 6.78 (br. s, 1H), 3.84 (s, 3H). ^{13}C NMR (125 MHz, $CDCl_3$, 25 ºC, δ): 153.1, 146.4, 143.8, 140.9, 129.5, 120.5 (q, J = 263.7 Hz), 116.4, 53.6. ^{19}F NMR (376 MHz, $CDCl_3$, 25 ºC, δ): –55.5 (s). Mass Spectrometry: HRMS (ESI-TOF) (m/z): calcd for $C_8H_6BrClF_3N_2O_3$ ([M + H]$^+$), 348.9197, found, 348.9198.

Methyl (2-chloro-4-(trifluoromethoxy)pyridin-3-yl)carbamate (2g)

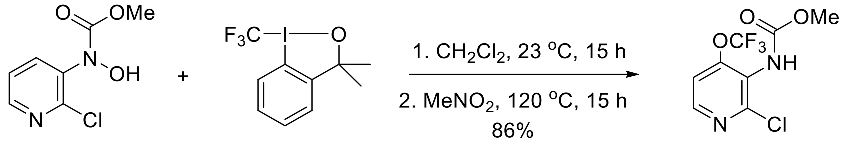

1g 1.2 equiv **2g**

A solution of methyl (2-chloropyridin-3-yl)(hydroxy)carbamate (**1g**) (101 mg, 0.500 mmol) and Togni reagent I (198 mg, 0.600 mmol, 1.20 equiv) in CH_2Cl_2 (5.00 mL, 0.100 M) was stirred at 23 °C under N_2 atmosphere for 15 h. The reaction mixture was concentrated *in vacuo*. The residue was dissolved in $MeNO_2$ (5.00 mL, 0.100 M) and the reaction mixture was stirred at 120 °C for 15 h. The reaction mixture was concentrated *in vacuo* and purified by chromatography on silica gel, eluting with hexanes:EtOAc (10:1 to 5:1 (v/v)), to afford **2g** (73.0 mg, 0.270 mmol, 53% yield) and **2g′** (45.0 mg, 0.166 mmol, 33% yield).

Data for **2g**: white solid; R_f= 0.72 (EtOAc:hexanes 1:4 (v/v)). NMR Spectroscopy: ^{1}H NMR (700 MHz, $CDCl_3$, 25 ºC, δ): 8.10 (d, J = 5.59 Hz, 1H) 7.31 (d, J = 5.16 Hz, 1H) 6.25 (br. s, 1H) 3.80 (s, 3H). ^{13}C NMR (175 MHz, $CDCl_3$, 25 ºC, δ): 153.9, 153.1, 145.0, 143.8, 123.3, 120.8, 120.1 (q, J = 261.6 Hz), 53.4. ^{19}F NMR (376 MHz, $CDCl_3$, 25 ºC, δ): –57.3 (s). Mass Spectrometry: HRMS (ESI-TOF) (m/z): calcd for $C_8H_7ClF_3N_2O_3$ ([M + H]$^+$), 271.0092, found, 271.0093.

Data for **2g′**: white solid; R_f= 0.48 (EtOAc:hexanes 1:4 (v/v)) NMR Spectroscopy: ^{1}H NMR (700 MHz, $CDCl_3$, 25 ºC, δ): 8.32 (d, J = 5.59 Hz, 1H) 7.23–7.20 (m, 1H) 6.28 (br. s, 1H) 3.80 (s, 3H). ^{13}C NMR (175 MHz, $CDCl_3$, 25 ºC, δ): 153.9, 152.8, 150.6, 148.2, 123.3, 120.2 (q, J = 261.5 Hz), 113.3, 53.5. ^{19}F NMR (376 MHz, $CDCl_3$, 25 ºC, δ): –58.4 (s). Mass Spectrometry: HRMS (ESI-TOF) (m/z): calcd for $C_8H_7ClF_3N_2O_3$ ([M + H]$^+$), 271.0092, found, 271.0095.

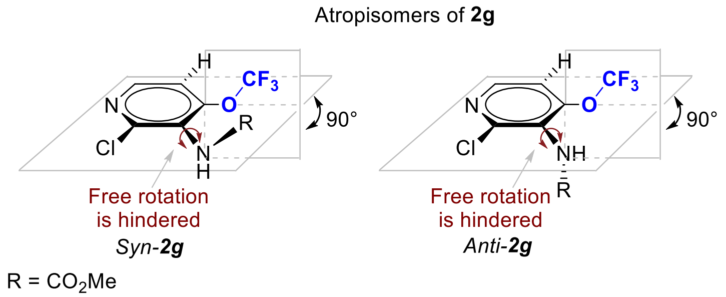

***N*-(2,6-Dichloro-4-(trifluoromethoxy)pyridin-3-yl)acetamide (2h)**

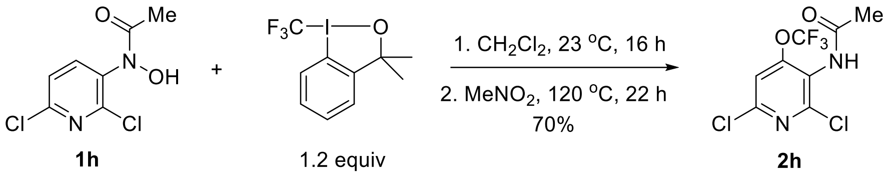

A solution of *N*-(2,6-dichloropyridin-3-yl)-*N*-hydroxyacetamide (**1h**) (50.0 mg, 0.226 mmol) and Togni reagent I (89.5 mg, 0.271 mmol, 1.20 equiv) in CH_2Cl_2 (2.26 mL, 0.100 M) was stirred at 23 °C under N_2 atmosphere for 16 h. The reaction mixture was concentrated *in vacuo*. The residue was dissolved in $MeNO_2$ (2.26 mL, 0.100 M) and the reaction mixture was stirred at 120 °C for 22 h. The reaction mixture was purified by preparative TLC (thickness: 1 mm) using hexanes:EtOAc (17:3 (v/v)) for development (prep TLC was developed five times) followed by recrystallization from Et_2O:hexanes. The purification afforded the title compound as a 1.4:1 mixture of atropisomers (45.8 mg, 0.158 mmol, 70% yield). The products could not readily be separated by silica gel chromatography or preparative TLC, so they were characterized as a mixture.

Data for the mixture of **2h**: white solid; R_f = 0.48 and 0.59 (EtOAc:hexanes 2:3 (v/v)). NMR Spectroscopy: ^{1}H NMR (500 MHz, $CDCl_3$, 25 ºC, δ): 7.36 (s, 1H), 7.24 (q, J = 1.8 Hz, 1H), 6.91 (br. s, 1H), 6.89 (br. s, 1H), 2.23 (s, 3H), 2.22 (s, 3H). ^{13}C NMR (125 MHz, $CDCl_3$, 25 ºC, δ): 168.5, 153.9, 151.8, 149.5, 149.1, 146.7, 145.9, 123.0, 122.4, 120.0 (q, J = 262.1 Hz), 119.9 (q, J = 263.1 Hz), 119.5, 113.6, 23.2. ^{19}F NMR (376 MHz, $CDCl_3$, 25 ºC, δ): –56.8 (s), –57.9 (s). Mass Spectrometry: HRMS (ESI-TOF) (m/z): calcd for $C_8H_6Cl_2F_3N_2O_2$ ($[M + H]^+$), 288.9753, found, 288.9756.

***N*-(6-Methoxy-4-methyl-2-(trifluoromethoxy)pyridin-3-yl)acetamide (2i)**

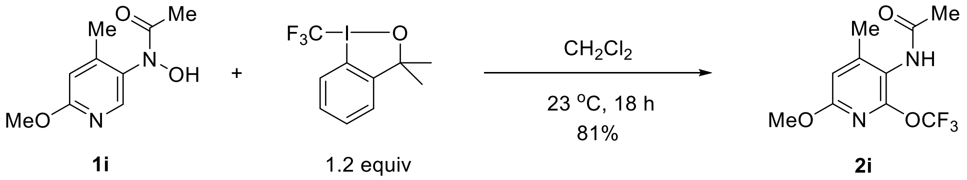

A solution of *N*-hydroxy-*N*-(6-methoxy-4-methylpyridin-3-yl)acetamide (**1i**) (50.0 mg, 0.255 mmol) and Togni reagent I (101 mg, 0.306 mmol, 1.20 equiv) in CH_2Cl_2 (2.55 mL, 0.100 M)

was stirred at 23 °C under N_2 atmosphere for 18 h. The reaction mixture was purified by preparative TLC (thickness: 1 mm) using hexanes:EtOAc (3:2 (v/v)) for development (prep TLC was developed five times). The purification afforded the title compound as a white solid (54.5 mg, 0.206 mmol, 81% yield).

R_f = 0.63 (EtOAc:hexanes 3:2 (v/v)). NMR Spectroscopy[4]: ^{1}H NMR (400 MHz, $CDCl_3$, 60 ºC, δ): 6.89 (br. s, 1H), 6.49 (br. s, 1H), 3.86 (s, 3H), 2.20 (br. s, 3H), 2.14 (br. s, 3H). ^{13}C NMR (100 MHz, $CDCl_3$, 60 ºC, δ): 169.3, 161.0, 152.1, 150.1, 120.4 (q, J = 260.2 Hz), 113.9, 109.2, 54.0, 23.0, 18.3. ^{19}F NMR (376 MHz, $CDCl_3$, 60 ºC, δ): –56.4 (s). Mass Spectrometry: HRMS (ESI-TOF) (m/z): calcd for $C_{10}H_{12}F_3N_2O_3$ ([M + H]$^+$), 265.0795, found, 265.0801.

***N*-(6-Methyl-2-(trifluoromethoxy)pyridin-3-yl)acetamide (2j)**

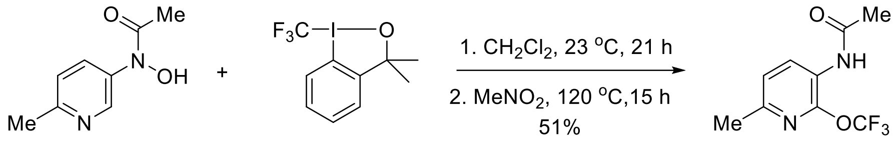

1j 1.2 equiv **2j**

A solution of *N*-hydroxy-*N*-(6-methylpyridin-3-yl)acetamide (**1j**) (50.0 mg, 0.301 mmol) and Togni reagent I (119.2 mg, 0.361 mmol, 1.20 equiv) in CH_2Cl_2 (3.01 mL, 0.100 M) was stirred at 23 °C under N_2 atmosphere for 21 h. The reaction mixture was concentrated *in vacuo*. The residue was dissolved in $MeNO_2$ (3.01 mL, 0.100 M) and the reaction mixture was stirred at 120 °C for 15 h. The reaction mixture was purified by preparative TLC (thickness: 1 mm) using hexanes:EtOAc (4:1 (v/v)) for development (prep TLC was developed four times). The purification afforded the title compound as a white solid (35.8 mg, 0.153 mmol, 51% yield).

R_f = 0.24 (EtOAc:hexanes 4:1 (v/v)). NMR Spectroscopy: ^{1}H NMR (400 MHz, $CDCl_3$, 25 ºC, δ): 8.57 (d, J = 8.28 Hz, 1H), 7.39 (br. s, 1H), 7.02 (d, J = 8.28 Hz, 1H), 2.44 (s, 3H), 2.22 (s, 3H). ^{13}C NMR (125 MHz, $CDCl_3$, 60 ºC, δ): 168.8, 151.1, 144.7, 130.5, 121.6, 121.1, 120.2 (q, J = 261.0 Hz), 24.8, 23.4. ^{19}F NMR (376 MHz, $CDCl_3$, 25 ºC, δ): –56.1 (s). Mass Spectrometry: HRMS (ESI-TOF) (m/z): calcd for $C_9H_{10}F_3N_2O_2$ ([M + H]$^+$), 235.0689, found, 235.0690.

Methyl (5-(2,4-difluorophenyl)-6-methoxy-2-(trifluoromethoxy)pyridin-3-yl)carbamate (2k)

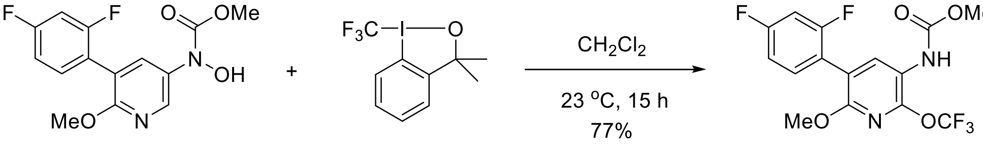

1k 1.2 equiv **2k**

A solution of methyl (5-(2,4-difluorophenyl)-6-methoxypyridin-3-yl)(hydroxy)carbamate (**1k**)

---

[4] At room temperature, a mixture of rotamers was observed.

(50.0 mg, 0.161 mmol) and Togni reagent I (63.8 mg, 0.193 mmol, 1.20 equiv) in CH_2Cl_2 (1.58 mL, 0.100 M) was stirred at 23 °C under N_2 atmosphere for 15 h. The reaction mixture was purified by preparative TLC (thickness: 1 mm) using hexanes:EtOAc (19:1 (v/v)) for development (prep TLC was developed three times). The purification afforded the title compound as a white solid (46.6 mg, 0.123 mmol, 77% yield).

R_f = 0.60 (EtOAc:hexanes 1:4 (v/v)). NMR Spectroscopy: ^{1}H NMR (700 MHz, $CDCl_3$, 25 ºC, δ): 8.38 (br. s., 1H), 7.35 (td, J = 8.41, 6.53 Hz, 1H), 6.97–6.85 (m, 2H), 6.65 (br. s, 1H), 3.88 (s, 3H), 3.79 (s, 3H). ^{13}C NMR (175 MHz, $CDCl_3$, 25 ºC, δ): 163.0 (dd, J = 248.1 Hz, J = 11.6 Hz), 160.2 (dd, J = 249.4 Hz, J = 11.5 Hz), 154.7, 154.0, 142.8, 134.3, 132.6 (m), 120.3 (q, J = 261.0 Hz), 119.4 (dd, J = 15.3 Hz, J = 3.8 Hz), 116.2, 115.6, 111.4 (dd, J = 21.2 Hz, J = 3.1 Hz), 104.3 (t, J = 25.5 Hz), 54.5, 52.9. ^{19}F NMR (376 MHz, $CDCl_3$, 25 ºC, δ): –56.4 (s), –110.3 (s), –110.5 (s). Mass Spectrometry: HRMS (ESI-TOF) (m/z): calcd for $C_{15}H_{12}F_5N_2O_4$ ([M + H]$^+$), 379.0712, found, 379.0719.

Methyl (6-(4-(*tert*-butyl)phenoxy)-2-(trifluoromethoxy)pyridin-3-yl)carbamate (2l)

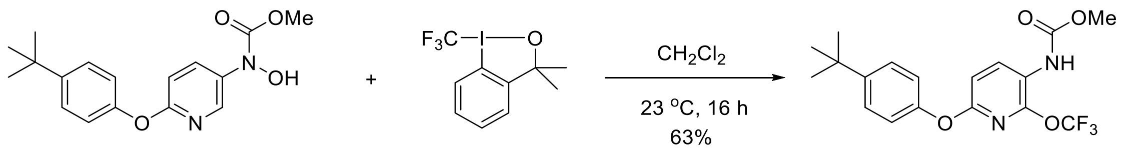

A solution of methyl (6-(4-(*tert*-butyl)phenoxy)pyridin-3-yl)(hydroxy)carbamate (**1l**) (50.0 mg, 0.158 mmol) and Togni reagent I (62.9 mg, 0.190 mmol, 1.20 equiv) in CH_2Cl_2 (1.58 mL, 0.100 M) was stirred at 23 °C under N_2 atmosphere for 16 h. The reaction mixture was purified by preparative TLC (thickness: 1 mm) using hexanes:EtOAc (19:1 (v/v)) for development (prep TLC was developed four times). The purification afforded the title compound as a colorless oil (38.1 mg, 0.0991 mmol, 63% yield).

R_f = 0.57 (EtOAc:hexanes 1:4 (v/v)). NMR Spectroscopy: ^{1}H NMR (400 MHz, $CDCl_3$, 25 ºC, δ): 8.43 (br. s, 1H), 7.45–7.33 (m, 2H), 7.12–7.00 (m, 2H), 6.71 (app. d, J = 8.8 Hz, 2H), 3.80 (s, 3H), 1.33 (s, 9H). ^{13}C NMR (175 MHz, $CDCl_3$, 25 ºC, δ): 156.7, 153.9, 151.6, 147.9, 143.1, 132.9, 126.6, 120.2, 120.1 (q, J = 261.6 Hz), 118.4, 108.3, 52.9, 34.6, 31.6. ^{19}F NMR (376 MHz, $CDCl_3$, 25 ºC, δ): –56.6 (s). Mass Spectrometry: HRMS (ESI-TOF) (m/z): calcd for $C_{18}H_{20}F_3N_2O_4$ ([M + H]$^+$), 385.1370, found, 385.1375.

Methyl (5-(5-formylfuran-2-yl)-6-methoxy-2-(trifluoromethoxy)pyridin-3-yl)carbamate

(2m)

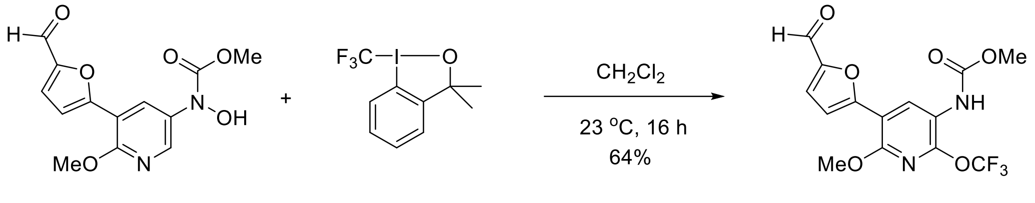

A solution of methyl (5-(5-formylfuran-2-yl)-6-methoxypyridin-3-yl)(hydroxy)carbamate (**1m**) (31.6 mg, 0.108 mmol) and Togni reagent I (42.9 mg, 0.130 mmol, 1.20 equiv) in CH_2Cl_2 (1.08 mL, 0.100 M) was stirred at 23 °C under N_2 atmosphere for 16 h. The reaction mixture was purified by preparative TLC (thickness: 1 mm) using hexanes:EtOAc (3:2 (v/v)) for development (prep TLC was developed twice). The purification afforded the title compound as an off-white solid (24.8 mg, 0.0688 mmol, 64% yield).

R_f = 0.50 (EtOAc:hexanes 3:7 (v/v)). NMR Spectroscopy: ^{1}H NMR (700 MHz, $CDCl_3$, 25 ºC, δ): 9.70 (s, 1H), 8.94 (br. s, 1H), 7.32 (d, J = 3.44 Hz, 1H), 7.13 (d, J = 3.44 Hz, 1H), 6.68 (br. s, 1H), 4.03 (s, 3H), 3.83 (s, 3H). ^{13}C NMR (175 MHz, $CDCl_3$, 25 ºC, δ): 177.9, 154.0, 153.9, 153.2, 152.0, 143.2, 130.2, 122.4, 120.1 (q, J = 261.8 Hz), 116.7, 113.1, 110.6, 54.7, 53.0. ^{19}F NMR (376 MHz, $CDCl_3$, 25 ºC, δ): –56.4 (s). Mass Spectrometry: HRMS (ESI-TOF) (m/z): calcd for $C_{14}H_{12}F_3N_2O_6$ ([M + H]$^+$), 361.0642, found, 361.0643.

***N*-(6-(1*H*-Pyrazol-1-yl)-2-(trifluoromethoxy)pyridin-3-yl)acetamide (2n)**

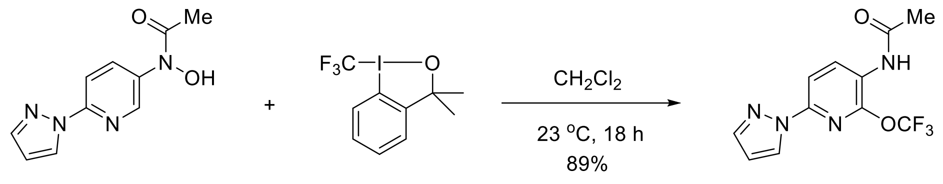

A solution of *N*-(6-(1*H*-pyrazol-1-yl)pyridin-3-yl)-*N*-hydroxyacetamide (**1n**) (50.0 mg, 0.229 mmol) and Togni reagent I (90.8 mg, 0.275 mmol, 1.20 equiv) in CH_2Cl_2 (2.29 mL, 0.100 M) was stirred at 23 °C under N_2 atmosphere for 18 h. The reaction mixture was purified by preparative TLC (thickness: 1 mm) using hexanes:EtOAc (9:1 (v/v)) for development (prep TLC was developed three times). The purification afforded the title compound as a white solid (58.0 mg, 0.203 mmol, 89% yield).

R_f = 0.30 (EtOAc:hexanes 3:7 (v/v)). NMR Spectroscopy: ^{1}H NMR (400 MHz, $CDCl_3$, 25 ºC, δ): 8.85 (d, J = 8.60 Hz, 1H), 8.34 (d, J = 2.58 Hz, 1H), 7.83 (d, J = 9.03 Hz, 1H), 7.70 (s, 1H), 7.43 (br. s, 1H), 6.43 (m, 1H), 2.25 (s, 3H). ^{13}C NMR (175 MHz, $CDCl_3$, 25 ºC, δ): 168.6, 143.9, 143.5, 142.5, 133.2, 127.2, 120.9, 120.2 (q, J = 262.1 Hz), 109.8, 108.2, 24.8. ^{19}F NMR (376 MHz, $CDCl_3$, 25 ºC, δ): –56.5 (s). Mass Spectrometry: HRMS (ESI-TOF) (m/z): calcd for $C_{11}H_{10}F_3N_4O_2$ ([M + H]$^+$), 287.0750, found, 275.0754.

Methyl (6-(1*H*-1,2,4-triazol-1-yl)-2-(trifluoromethoxy)pyridin-3-yl)carbamate (2o) and methyl (6-(1*H*-1,2,4-triazol-1-yl)-4-(trifluoromethoxy)pyridin-3-yl)carbamate (2o-II)

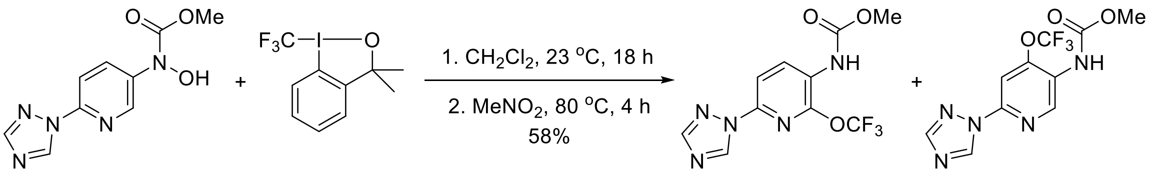

1o 1.2 equiv **2o**, 52% **2o-II**, 6%

A solution of methyl (6-(1*H*-1,2,4-triazol-1-yl)pyridin-3-yl)(hydroxy)carbamate (**1o**) (100 mg, 0.425 mmol) and Togni reagent I (168 mg, 0.510 mmol, 1.20 equiv) in CH_2Cl_2 (4.25 mL, 0.100 M) was stirred at 23 °C under N_2 atmosphere for 18 h. The reaction mixture was concentrated *in vacuo*. The residue was dissolved in $MeNO_2$ (4.25 mL, 0.100 M) and the reaction mixture was stirred at 80 °C for 4 h. The reaction mixture was placed in the freezer. The solid was filtered and washed with hexanes. Filtration afforded **2o** (61.7 mg, 0.203 mmol, 48% yield) as a slightly brown solid. The filtrate was concentrated *in vacuo* and purified by preparative TLC (thickness: 1 mm) using hexanes:Et_2O (7:3 (v/v)) for development (prep TLC was developed six times). The purification afforded **2o** (3.7 mg, 0.0122 mmol, 3%) and 9.3 mg (0.0307 mmol, 7% yield) of a 1:4.6 mixture of **2o** and **2o-II**. The combined reaction yield was 58%. The characterization data for **2o-II** was obtained by further purification of the mixture by preparative TLC using hexanes:EtOAc (7:3 (v/v)) for development (prep TLC was developed six times).

Data for **2o**: white solid; R_f = 0.41 (EtOAc:hexanes 1:1 (v/v)). NMR Spectroscopy: ^{1}H NMR (500 MHz, $CDCl_3$, 25 ºC, δ): 8.95 (s, 1H), 8.75 (d, *J* = 7.93 Hz, 1H), 8.08 (s, 1H), 7.80 (d, *J* = 8.54 Hz, 1H), 7.00 (br. s, 1H), 3.85 (s, 3H). ^{13}C NMR (125 MHz, $CDCl_3$, 25 ºC, δ): 153.5, 153.2, 143.2, 141.4, 140.6, 131.4, 123.0, 120.1 (q, *J* = 262.8 Hz), 110.9, 53.2. ^{19}F NMR (376 MHz, $CDCl_3$, 25 ºC, δ): –56.7 (s). Mass Spectrometry: HRMS (ESI-TOF) (m/z): calcd for $C_{10}H_9F_3N_5O_3$ ($[M + H]^+$), 304.0652, found, 304.0658.

Data for **2o-II**: white solid; R_f = 0.27 (EtOAc:hexanes 1:1 (v/v)). NMR Spectroscopy: ^{1}H NMR (700 MHz, $CDCl_3$, 25 ºC, δ): 9.30 (br. s, 1H), 9.14 (s, 1H), 8.08 (s, 1H), 7.81 (s, 1H), 6.88 (br. s, 1H), 3.83 (s, 3H). ^{13}C NMR (175 MHz, $CDCl_3$, 25 ºC, δ): 153.3, 153.0, 145.9, 145.3, 141.8, 141.2, 126.1, 120.3 (q, *J* = 262.0 Hz), 102.7, 53.4. ^{19}F NMR (376 MHz, $CDCl_3$, 25 ºC, δ): –57.3 (d). Mass Spectrometry: HRMS (ESI-TOF) (m/z): calcd for $C_{10}H_9F_3N_5O_3$ ($[M + H]^+$), 304.0652, found, 304.0657.

N-(6-(1H-Benzo[d]imidazol-1-yl)-2-(trifluoromethoxy)pyridin-3-yl)acetamide (2p)

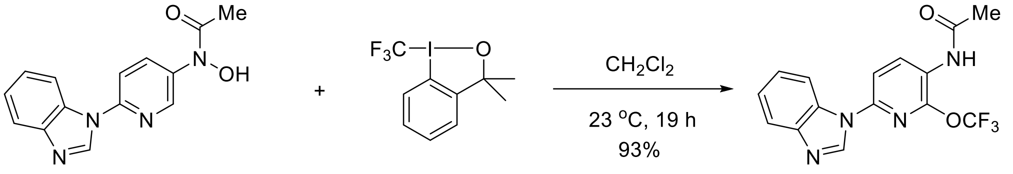

1p 1.2 equiv **2p**

A solution of *N*-(6-(1*H*-benzo[*d*]imidazol-1-yl)pyridin-3-yl)-*N*-hydroxyacetamide (**1p**) (100 mg, 0.373 mmol) and Togni reagent I (148 mg, 0.448 mmol, 1.20 equiv) in CH_2Cl_2 (3.73 mL, 0.100 M) was stirred at 23 °C under N_2 atmosphere for 19 h. The reaction mixture was concentrated *in vacuo* (to about 10% initial volume) and the residue was triturated with hexanes. The purification afforded the title compound as a beige solid (117 mg, 0.348 mmol, 93% yield).

R_f = 0.21 (EtOAc:hexanes 4:1 (v/v)). NMR Spectroscopy: ^{1}H NMR (700 MHz, $CDCl_3$, 25 ºC, δ): 9.00 (d, *J* = 8.60 Hz, 1H), 8.52 (s, 1H), 8.04 (d, *J* = 8.17 Hz, 1H), 7.86 (d, *J* = 7.74 Hz, 1H), 7.60 (br. s, 1H), 7.49 (d, *J* = 8.60 Hz, 1H), 7.43–7.35 (m, 2H), 2.31 (s, 3H). ^{13}C NMR (175 MHz, $CDCl_3$, 25 ºC, δ): 168.9, 144.3, 144.2, 141.8, 140.9, 133.1, 131.9, 124.8, 123.9, 121.5, 120.7, 120.2 (q, *J* = 262.4 Hz), 112.8, 111.6, 24.9. ^{19}F NMR (376 MHz, $CDCl_3$, 25 ºC, δ): –56.5 (s). Mass Spectrometry: HRMS (ESI-TOF) (m/z): calcd for $C_{15}H_{12}F_3N_4O_2$ ([M + H]$^+$), 337.0907, found, 337.0910.

N-(6-(1H-Benzo[d][1,2,3]triazol-1-yl)-2-(trifluoromethoxy)pyridin-3-yl)acetamide (2q)

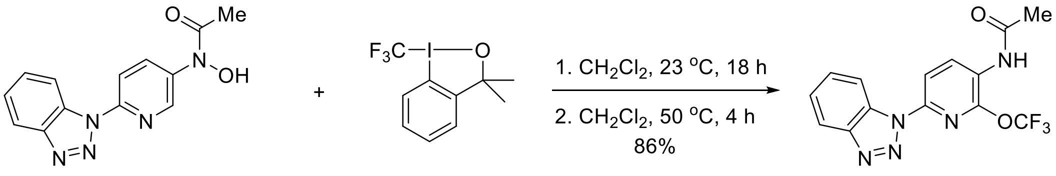

1q 1.2 equiv **2q**

A solution of *N*-(6-(1*H*-benzo[*d*][1,2,3]triazol-1-yl)pyridin-3-yl)-*N*-hydroxyacetamide (**1q**) (100 mg, 0.371 mmol) and Togni reagent I (147 mg, 0.445 mmol, 1.20 equiv) in CH_2Cl_2 (3.71 mL, 0.100 M) was stirred at 23 °C under N_2 atmosphere for 18 h. The reaction mixture was then stirred at 50 °C for 4h. The reaction mixture was concentrated *in vacuo* and the residue was triturated with hexanes. The purification afforded the title compound as a slightly yellow solid (108 mg, 0.319 mmol, 86% yield).

R_f = 0.37 (EtOAc:hexanes 2:3 (v/v)). NMR Spectroscopy: ^{1}H NMR (400 MHz, $(CD_3)_2SO$, 25 ºC, δ): 10.04 (s, 1H), 8.73 (d, *J* = 8.53 Hz, 1H), 8.42–8.32 (m, 1H), 8.30–8.18 (m, 2H), 7.77 (t, *J* = 7.65 Hz, 1H), 7.63–7.51 (m, 1H), 2.18 (s, 3H). ^{13}C NMR (175 MHz, $(CD_3)_2SO$, 25 ºC, δ): 169.4, 146.0, 144.9, 142.7, 136.8, 130.5, 129.6, 125.5, 122.5, 119.9, 119.8 (q, *J* = 260.0 Hz), 113.0, 112.6, 23.6. ^{19}F NMR (376 MHz, $(CD_3)_2SO$, 25 ºC, δ): –57.0 (s). Mass Spectrometry: HRMS (ESI-TOF) (m/z): calcd for $C_{14}H_{11}F_3N_5O_2$ ([M + H]$^+$), 338.0859, found, 338.0860.

N-(6-(5-Fluoro-1H-indol-1-yl)-2-(trifluoromethoxy)pyridin-3-yl)acetamide (2r)

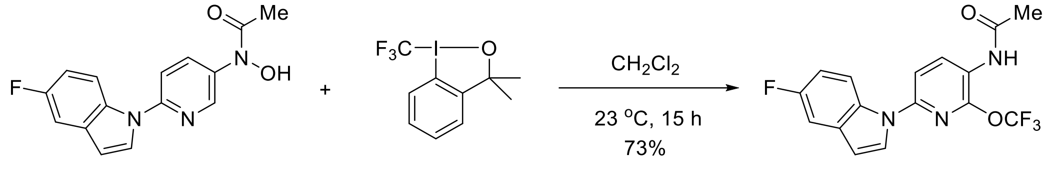

A solution of N-(6-(5-fluoro-1H-indol-1-yl)pyridin-3-yl)-N-hydroxyacetamide (**1r**) (50.0 mg, 0.175 mmol) and Togni reagent I (69.3 mg, 0.210 mmol, 1.20 equiv) in CH_2Cl_2 (1.75 mL, 0.100 M) was stirred at 23 °C under N_2 atmosphere for 15 h. The reaction mixture was concentrated *in vacuo*. The crude residue was triturated with hexanes. The crude residue was purified by flash chromatography eluting with EtOAc:hexanes (3:17 to 1:1(v/v). The purification afforded the title compound as a yellow soild (45.0 mg, 0.127 mmol, 73% yield).

R_f = 0.36 (EtOAc:hexanes 2:3 (v/v)). NMR Spectroscopy: ^{1}H NMR (400 MHz, $CDCl_3$, 25 ºC, δ): 8.89 (d, J = 8.78 Hz, 1H), 8.20 (dd, J = 9.03, 4.52 Hz, 1H), 7.64 (d, J = 3.51 Hz, 1H), 7.35 (br. s, 1H), 7.32 (d, J = 8.78 Hz, 2H), 7.28 (dd, J = 9.29, 2.51 Hz, 1H), 7.04 (td, J = 9.10, 2.64 Hz, 1H), 6.67 (d, J = 3.51 Hz, 1H), 2.29 (s, 3H). ^{13}C NMR (175 MHz, $CDCl_3$, 25 ºC, δ): 168.7, 158.8 (d, J = 236.1 Hz), 144.8, 143.9, 133.0, 131.6, 131.1 (d, J = 10.0 Hz), 126.9, 120.2 (q, J = 262.1 Hz), 119.6, 114.4 (d, J = 9.1 Hz), 111.7 (d, J = 25.2 Hz), 111.0, 106.3 (m), 106.2, 24.9. ^{19}F NMR (376 MHz, $CDCl_3$, 25 ºC, δ): –56.4 (s), –122.9 (m). Mass Spectrometry: HRMS (ESI-TOF) (m/z): calcd for $C_{16}H_{12}F_4N_3O_2$ ([M + H]$^+$), 354.0860, found, 354.0865.

N-(6-(5-Bromo-1H-pyrrolo[2,3-b]pyridin-1-yl)-2-(trifluoromethoxy)pyridin-3-yl)acetamide (2s)

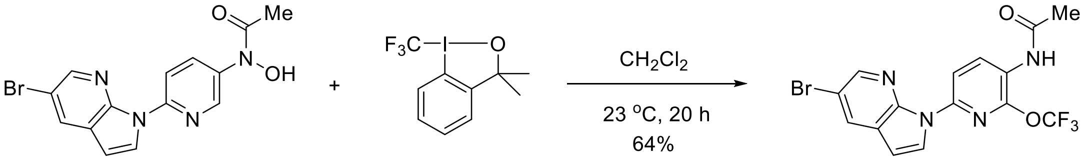

A solution of N-(6-(5-bromo-1H-pyrrolo[2,3-b]pyridin-1-yl)pyridin-3-yl)-N-hydroxyacetamide (**1s**) (50.0 mg, 0.144 mmol) and Togni reagent I (57.1 mg, 0.172 mmol, 1.20 equiv) in CH_2Cl_2 (1.44 mL, 0.100 M) was stirred at 23 °C under N_2 atmosphere for 20 h. The reaction mixture was concentrated *in vacuo*. The crude residue was purified by flash chromatography eluting with EtOAc:hexanes (1:19 to 2:3(v/v)). The crude product was triturated with haxanes. The purification afforded the title compound as a white solid (38.2 mg, 0.0920 mmol, 64% yield).

R_f = 0.39 (EtOAc:hexanes 3:7 (v/v)). NMR Spectroscopy: ^{1}H NMR (700 MHz, $CDCl_3$, 25 ºC, δ): 8.89 (d, J = 9.03 Hz, 1H), 8.81 (d, J = 8.60 Hz, 1H), 8.41 (d, J = 2.15 Hz, 1H), 8.19 (d, J = 3.87 Hz, 1H), 8.05 (d, J = 2.15 Hz, 1H), 7.34 (br. s, 1H), 6.57 (d, J = 3.87 Hz, 1H), 2.28 (s, 3H). ^{13}C NMR (175 MHz, $CDCl_3$, 25 ºC, δ): 168.5, 145.8, 144.0, 143.5, 142.6, 133.0, 131.4, 127.5, 125.0, 120.2

(q, J = 261.8 Hz), 120.0, 113.6, 112.7, 102.6, 24.9. ^{19}F NMR (376 MHz, $CDCl_3$, 25 ºC, δ): –56.4 (s). Mass Spectrometry: HRMS (ESI-TOF) (m/z): calcd for $C_{15}H_{11}BrF_3N_4O_2$ ($[M + H]^+$), 415.0012, found, 415.0018.

_N_-(6-(2-(Thiazol-4-yl)-1_H_-benzo[_d_]imidazol-1-yl)-2-(trifluoromethoxy)pyridin-3-yl)acetamide (2t)

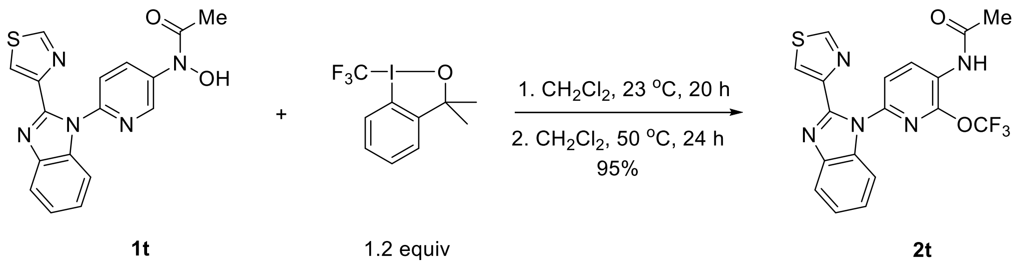

A solution of _N_-hydroxy-_N_-(6-(2-(thiazol-4-yl)-1_H_-benzo[_d_]imidazol-1-yl)pyridin-3-yl)acetamide (**1t**) (100 mg, 0.285 mmol) and Togni reagent I (113 mg, 0.188 mmol, 1.20 equiv) in CH_2Cl_2 (2.85 mL, 0.100 M) was stirred at 23 °C under N_2 atmosphere for 20 h. The reaction mixture was then stirred at 50 °C for 4h. The reaction mixture was concentrated _in vacuo_ and the residue was triturated with hexanes. The purification afforded the title compound as a beige solid (113 mg, 0.270 mmol, 95% yield).

R_f = 0.42 (EtOAc). NMR Spectroscopy: ^{1}H NMR (700 MHz, $(CD_3)_2SO$, 25 ºC, δ): 10.00 (s, 1H), 9.05 (s, 1H), 8.64 (d, J = 8.60 Hz, 1H), 8.48 (s, 1H), 7.82 (d, J = 7.31 Hz, 1H), 7.55 (d, J = 8.17 Hz, 1H), 7.47 (d, J = 7.31 Hz, 1H), 7.40–7.30 (m, 2H), 2.18 (s, 3H). ^{13}C NMR (175 MHz, $(CD_3)_2SO$, 25 ºC, δ): 169.5, 154.8, 146.6, 145.8, 145.0, 142.3, 141.2, 135.2, 135.1, 124.1, 123.5, 123.4, 122.7, 119.8, 119.6, 119.5 (q, J = 260.0 Hz), 110.8, 23.6. ^{19}F NMR (376 MHz, $(CD_3)_2SO$, 25 ºC, δ): –57.2 (s). Mass Spectrometry: HRMS (ESI-TOF) (m/z): calcd for $C_{18}H_{13}F_3N_5O_2S$ ($[M + H]^+$), 420.0737, found, 420.0738.

_N_-(6-(2,6-Dichloro-9_H_-purin-9-yl)-2-(trifluoromethoxy)pyridin-3-yl)acetamide (2u) and _N_-

(6-(2,6-dichloro-9*H*-purin-9-yl)-4-(trifluoromethoxy)pyridin-3-yl)acetamide (2u-II)

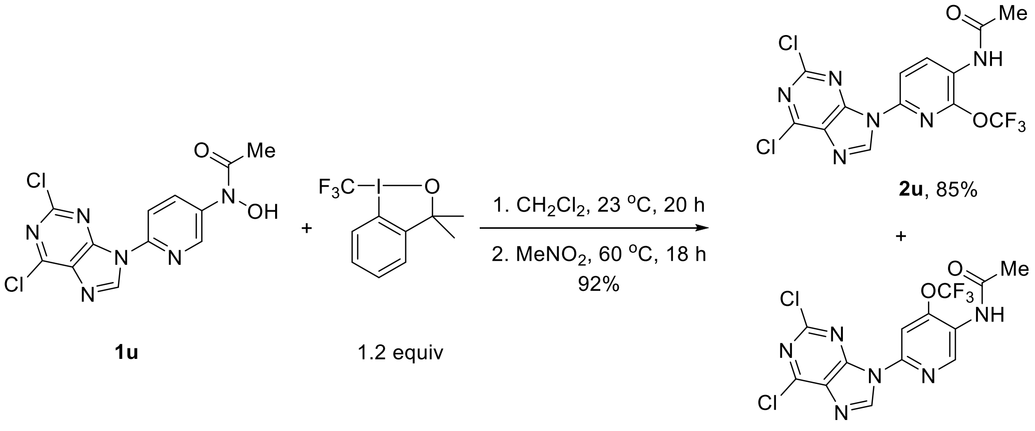

A solution of *N*-(6-(2,6-dichloro-9*H*-purin-9-yl)pyridin-3-yl)-*N*-hydroxyacetamide (**1u**) (38.4 mg, 0.113 mmol) and Togni reagent I (44.9 mg, 0.136 mmol, 1.20 equiv) in CH_2Cl_2 (1.13 mL, 0.100 M) was stirred at 23 °C under N_2 atmosphere for 20 h. The reaction mixture was concentrated *in vacuo*. The residue was dissolved in $MeNO_2$ (1.13 mL, 0.100 M) and the reaction mixture was stirred at 60 °C for 18 h. The reaction mixture was concentrated *in vacuo*. The residue was purified by preparative TLC (thickness: 1 mm) using hexanes:EtOAc (1:1 (v/v)) for development (prep TLC was developed three times). The purification afforded **2u** (39.0 mg, 0.0968 mmol, 85% yield) and **2u-II** (3.3 mg, 0.0081 mmol, 7% yield).

Data for **2u**: white solid; R_f = 0.44 (EtOAc:hexanes 1:1 (v/v)). NMR Spectroscopy: ^{1}H NMR (700 MHz, $CDCl_3$, 25 ºC, δ): 9.11 (d, *J* = 8.60 Hz, 1H), 8.94 (s, 1H), 8.47 (d, *J* = 8.60 Hz, 1H), 7.53 (s, 1H), 2.31 (s, 3H). ^{13}C NMR (175 MHz, $CDCl_3$, 25 ºC, δ): 168.8, 153.9, 152.6, 151.7, 143.6, 143.5, 138.4, 133.1, 132.3, 123.2, 120.1 (q, *J* = 263.4 Hz), 113.1, 24.9. ^{19}F NMR (376 MHz, $CDCl_3$, 25 ºC, δ): –56.7 (s). Mass Spectrometry: HRMS (ESI-TOF) (m/z): calcd for $C_{13}H_8Cl_2F_3N_6O_2$ ($[M + H]^+$), 407.0032, found, 407.0037.

Data for **2u-II**: white solid; R_f = 0.22 (EtOAc:hexanes 1:1 (v/v)). NMR Spectroscopy: ^{1}H NMR (700 MHz, $CDCl_3$, 25 ºC, δ): 9.60 (s, 1H), 9.20 (s, 1H), 8.67 (d, *J* = 1.72 Hz, 1H), 7.39 (br. s, 1H), 2.33 (s, 3H). ^{13}C NMR (175 MHz, $CDCl_3$, 25 ºC, δ): 168.4, 153.9, 152.7, 151.6, 145.8, 143.9, 143.3, 142.9, 132.4, 126.2, 120.4 (q, *J* = 262.3 Hz), 104.6, 24.7. ^{19}F NMR (376 MHz, $CDCl_3$, 25 ºC, δ): –57.9 (s). Mass Spectrometry: HRMS (ESI-TOF) (m/z): calcd for $C_{13}H_8Cl_2F_3N_6O_2$ ($[M + H]^+$), 407.0032, found, 407.0035.

Ethyl (*E*)-3-(4-fluoro-3-(2-methoxy-5-((methoxycarbonyl)amino)-6-

(trifluoromethoxy)pyridin-3-yl)phenyl)acrylate (2v)

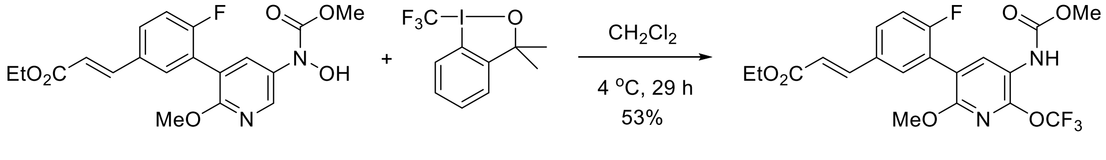

1v 1.2 equiv **2v**

A solution of ethyl (*E*)-3-(4-fluoro-3-(5-(hydroxy(methoxycarbonyl)amino)-2-methoxypyridin-3-yl)phenyl)acrylate (**1v**) (46.1 mg, 0.118 mmol) in CH_2Cl_2 (11.3 mL) was cooled to 4 °C. A solution of Togni reagent I (46.9 mg, 0.142 mmol, 1.20 equiv) in CH_2Cl_2 (0.500 mL) was then added dropwise and the reaction mixture was stirred at 4 °C for 29 h. The reaction mixture was concentrated *in vacuo*. The residue was purified by preparative TLC (thickness: 1 mm) using hexanes:EtOAc (9:1 (v/v)) for development (prep TLC was developed four times). The purification afforded the title compound as a white solid (28.5 mg, 0.0622 mmol, 53% yield).

R_f = 0.62 (EtOAc:hexanes 3:7 (v/v)). NMR Spectroscopy: ^{1}H NMR (700 MHz, $CDCl_3$, 25 ºC, δ): 8.44 (br. s, 1H), 7.66 (d, *J* = 15.92 Hz, 1H), 7.57–7.50 (m, 2H), 7.15 (t, *J* = 9.03 Hz, 1H), 6.68 (br. s, 1H), 6.38 (d, *J* = 15.92 Hz, 1H), 4.26 (q, *J* = 6.88 Hz, 2H), 3.89 (s, 3H), 3.80 (s, 3H), 1.33 (t, *J* = 7.10 Hz, 3H). ^{13}C NMR (175 MHz, $CDCl_3$, 25 ºC, δ): 166.9, 161.1 (d, *J* = 252.7 Hz), 154.6, 154.0, 143.2, 134.2, 131.7 (d, *J* = 2.6 Hz), 130.9 (d, *J* = 3.3 Hz), 129.6 (d, *J* = 8.7 Hz), 124.0 (*J* = 15.6 Hz), 120.3 (q, *J* = 261.5 Hz), 118.6 (d, *J* = 1.8 Hz), 116.7, 116.6, 116.3, 115.7, 60.7, 54.5, 52.9, 14.4. ^{19}F NMR (376 MHz, $CDCl_3$, 25 ºC, δ): –56.4 (s), –111.4 (s). Mass Spectrometry: HRMS (ESI-TOF) (m/z): calcd for $C_{20}H_{19}F_4N_2O_6$ ($[M + H]^+$), 459.1174, found, 459.1184.

Methyl 4-((4-(2-methoxy-5-((methoxycarbonyl)amino)-6-(trifluoromethoxy)pyridin-3-yl)phenyl)ethynyl)benzoate (2w)

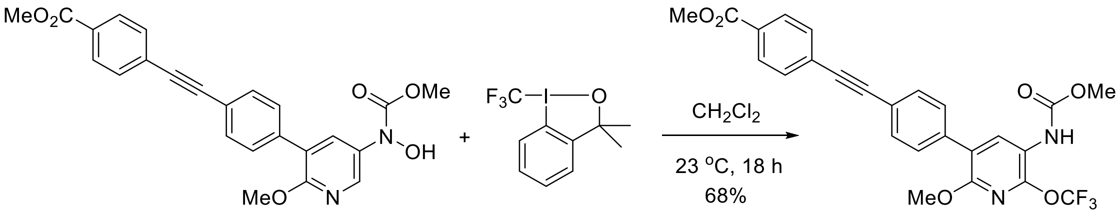

1w 1.2 equiv **2**
w

A solution of methyl 4-((4-(5-(hydroxy(methoxycarbonyl)amino)-2-methoxypyridin-3-yl)phenyl)ethynyl)benzoate (**1w**) (50.0 mg, 0.116 mmol) and Togni reagent I (45.9 mg, 0.139 mmol, 1.20 equiv) in CH_2Cl_2 (1.16 mL, 0.100 M) was stirred at 23 °C under N_2 atmosphere for 18 h. The reaction mixture was purified by preparative TLC (thickness: 1 mm) using CH_2Cl_2 for development (prep TLC was developed twice). The purification afforded the title compound as a white solid (39.6 mg, 0.0791 mmol, 68% yield).

R_f = 0.27 (CH_2Cl_2). NMR Spectroscopy: ^{1}H NMR (700 MHz, $CDCl_3$, 25 ºC, δ): 8.51 (br. s, 1H),

8.03 (d, J = 8.17 Hz, 2H), 7.68–7.50 (m, 6H), 6.68 (br. s, 1 H), 3.93 (s, 3H), 3.92 (s, 3H), 3.81 (s, 3H). ^{13}C NMR (175 MHz, $CDCl_3$, 25 ºC, δ): 166.7, 154.1, 142.3, 135.8, 133.1, 131.8, 131.7, 129.7, 129.6, 129.3, 128.1, 122.2, 121.8 (q, J = 260.8 Hz), 121.3, 119.5, 116.7, 92.4, 89.5, 54.4, 52.9, 52.4. ^{19}F NMR (376 MHz, $CDCl_3$, 25 ºC, δ): –56.4 (s). Mass Spectrometry: HRMS (ESI-TOF) (m/z): calcd for $C_{25}H_{20}F_3N_2O_6$ ([M + H]$^+$), 501.1268, found, 501.1273.

N-(6-(((8R,9S,13S,14S)-13-Methyl-17-oxo-7,8,9,11,12,13,14,15,16,17-decahydro-6H-cyclopenta[a]phenanthren-3-yl)oxy)-2-(trifluoromethoxy)pyridin-3-yl)acetamide (2x)

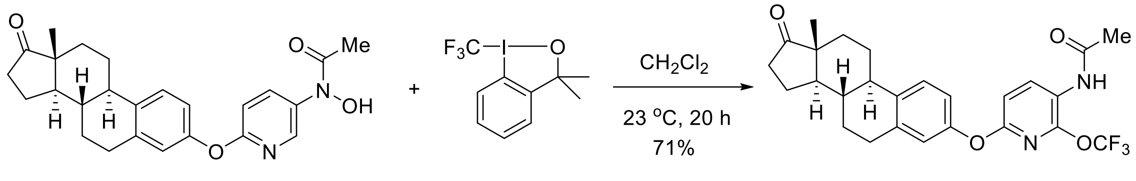

A solution of N-hydroxy-N-(6-(((8R,9S,13S,14S)-13-methyl-17-oxo-7,8,9,11,12,13,14,15,16,17-decahydro-6H-cyclopenta[a]phenanthren-3-yl)oxy)pyridin-3-yl)acetamide (**1x**) (66.0 mg, 0.157 mmol) and Togni reagent I (62.1 mg, 0.188 mmol, 1.20 equiv) in CH_2Cl_2 (1.57 mL, 0.100 M) was stirred at 23 °C under N_2 atmosphere for 20 h. The reaction mixture was purified by preparative TLC (thickness: 1 mm) using hexanes:EtOAc (3:2 (v/v)) for development (prep TLC was developed three times). The purification afforded the title compound as a white foamy solid (54.0 mg, 0.111 mmol, 71% yield).

R_f = 0.49 (EtOAc:hexanes 1:1 (v/v)). NMR Spectroscopy: ^{1}H NMR (700 MHz, $CDCl_3$, 25 ºC, δ): 8.62 (d, J = 8.61 Hz, 1H), 7.37 (s, 1H), 7.27 (d, J = 8.37 Hz, 1H), 6.91 (dd, J = 8.39, 2.37 Hz, 1H), 6.88 (d, J = 2.15 Hz, 1H), 6.68 (d, J = 9.03 Hz, 1H), 2.92–2.86 (m, 2H), 2.51 (dd, J = 18.93, 8.60 Hz, 1H), 2.44–2.37 (m, 1H), 2.30 (td, J = 11.19, 3.44 Hz, 1H), 2.22 (s, 3H), 2.18–2.11 (m, 1H), 2.09–2.04 (m, 1H), 2.04–1.99 (m, 1H), 1.96 (dt, J = 12.48, 3.01 Hz, 1H), 1.67–1.59 (m, 2H), 1.59–1.42 (m, 4H), 0.92 (s, 3H). ^{13}C NMR (175 MHz, $CDCl_3$, 25 ºC, δ): 221.0, 168.6, 156.9, 151.7, 143.5, 138.2, 136.5, 134.7, 126.6, 120.8, 120.1 (q, J = 261.5 Hz), 118.1, 118.0, 108.1, 50.6, 48.1, 44.2, 38.2, 36.0, 31.7, 29.5, 26.5, 25.9, 24.6, 21.7, 14.0. ^{19}F NMR (376 MHz, $CDCl_3$, 25 ºC, δ): –56.4 (s). Mass Spectrometry: HRMS (ESI-TOF) (m/z): calcd for $C_{26}H_{28}F_3N_2O_4$ ([M + H]$^+$), 489.1996, found, 489.2004.

N-(6-((6R,12aR)-6-(Benzo[d][1,3]dioxol-5-yl)-2-methyl-1,4-dioxo-1,3,4,6,12,12a-hexahydropyrazino[1',2':1,6]pyrido[3,4-b]indol-7(2H)-yl)-2-(trifluoromethoxy)pyridin-3-

yl)acetamide (2y)

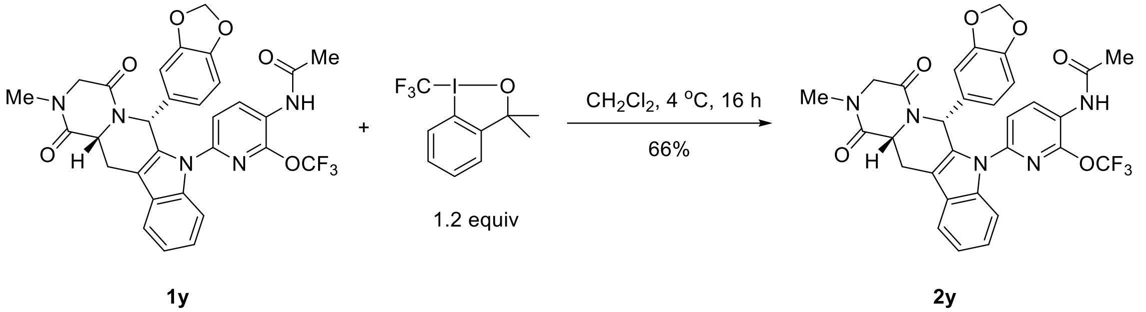

A solution of *N*-(6-((6*R*,12a*R*)-6-(benzo[*d*][1,3]dioxol-5-yl)-2-methyl-1,4-dioxo-1,3,4,6,12,12a-hexahydropyrazino[1',2':1,6]pyrido[3,4-*b*]indol-7(2*H*)-yl)-2-(trifluoromethoxy)pyridin-3-yl)acetamide (**1y**) (50.0 mg, 0.0927 mmol) in CH_2Cl_2 (8.77 mL) was cooled to 4 °C. A solution of Togni reagent I (39.3 mg, 0.119 mmol, 1.20 equiv) in CH_2Cl_2 (0.500 mL) was then added dropwise and the reaction mixture was stirred at 4 °C for 16 h. The reaction mixture was concentrated *in vacuo*. The residue was purified by preparative TLC (thickness: 1 mm) using hexanes:EtOAc (1:4 (v/v)) for development (prep TLC was developed twice). The purification afforded the title compound as a white solid (37.3 mg, 0.0614 mmol, 66% yield).

R_f = 0.48 (EtOAc). NMR Spectroscopy: ^{1}H NMR (700 MHz, $(CD_3)_2SO$, 25 ºC, δ): 9.99 (s, 1H), 8.53 (d, *J* = 8.53 Hz, 1H), 7.80–7.72 (m, 1H), 7.42–7.36 (m, 1H), 7.34 (d, *J* = 8.53 Hz, 1H), 7.25–7.19 (m, 2H), 6.59 (s, 1H), 6.51 (d, *J* = 8.03 Hz, 1H), 6.32 (d, *J* = 1.76 Hz, 1H), 6.23 (dd, *J* = 8.03, 1.76 Hz, 1H), 5.83 (s, 2H), 4.55 (dd, *J* = 11.54, 4.77 Hz, 1H), 4.24–4.15 (m, 1H), 3.92 (d, *J* = 17.32 Hz, 1H), 3.65 (dd, *J* = 16.19, 4.64 Hz, 1H), 3.12 (dd, *J* = 15.81, 12.30 Hz, 1H), 2.90 (s, 3H), 2.18 (s, 3H). ^{13}C NMR (175 MHz, $(CD_3)_2SO$, 25 ºC, δ): 169.4, 166.9, 166.3, 146.7, 146.0, 145.4, 141.7, 136.4, 136.0, 134.9, 134.2, 126.1, 123.4, 122.3, 121.3, 120.7, 119.7 (q, *J* = 262.6 Hz), 119.1, 118.4, 110.4, 109.9, 107.5, 107.4, 100.9, 54.7, 53.9, 51.4, 32.8, 23.6, 23.2. ^{19}F NMR (376 MHz, $(CD_3)_2SO$, 25 ºC, δ): –56.6 (s). Mass Spectrometry: HRMS (ESI-TOF) (m/z): calcd for $C_{30}H_{25}F_3N_5O_6$ ([M + H]$^+$), 608.1751, found, 608.1761.

***N*-(2-(2-(Thiazol-4-yl)-1*H*-benzo[*d*]imidazol-1-yl)-4-(trifluoromethoxy)pyrimidin-5-yl)acetamide (4a)**

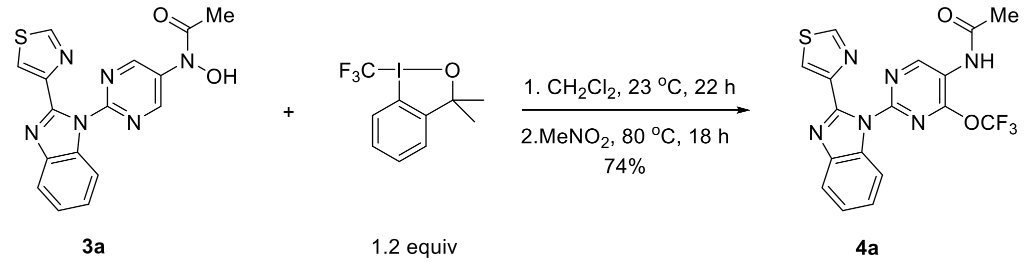

A solution of *N*-hydroxy-*N*-(2-(2-(thiazol-4-yl)-1*H*-benzo[*d*]imidazol-1-yl)pyrimidin-5-yl)acetamide (**3a**) (52.0 mg, 0.148 mmol) and Togni reagent I (58.8 mg, 0.178 mmol, 1.20 equiv)

in CH_2Cl_2 (1.48 mL, 0.100 M) was stirred at 23 °C under N_2 atmosphere for 22 h. The reaction mixture was concentrated *in vacuo*. The residue was dissolved in $MeNO_2$ (1.48 mL, 0.100 M) and the reaction mixture was stirred at 80 °C for 18 h. The reaction mixture was concentrated *in vacuo*. The residue was purified by preparative TLC (thickness: 1 mm) using hexanes:EtOAc (1:4 (v/v)) for development (prep TLC was developed twice). The purification afforded the title compound as an off-white solid (45.9 mg, 0.109 mmol, 74% yield).

R_f = 0.59 (EtOAc). NMR Spectroscopy: ^{1}H NMR (700 MHz, $CDCl_3$, 25 ºC, δ): 9.70 (s, 1H), 8.66 (d, *J* = 2.15 Hz, 1H), 8.14 (d, *J* = 2.15 Hz, 1H), 7.97 (dd, *J* = 6.24, 3.23 Hz, 1H), 7.86–7.82 (m, 1H), 7.80 (s, 1H), 7.42–7.36 (m, 2H), 2.23 (s, 3H). ^{13}C NMR (175 MHz, $CDCl_3$, 25 ºC, δ): 168.8, 152.7, 152.1, 151.5, 149.4, 147.3, 147.1, 142.7, 134.6, 125.1, 124.4, 120.8, 120.1, 119.6, 119.5 (q, *J* = 265.7 Hz), 112.8, 24.4. ^{19}F NMR (376 MHz, $CDCl_3$, 25 ºC, δ): –57.0 (s). Mass Spectrometry: HRMS (ESI-TOF) (m/z): calcd for $C_{17}H_{12}F_3N_6O_2S$ ([M + H]$^+$), 421.0689, found, 421.0693.

N-(2-(5-Chloro-1*H*-indol-1-yl)-4-(trifluoromethoxy)pyrimidin-5-yl)acetamide (4b)

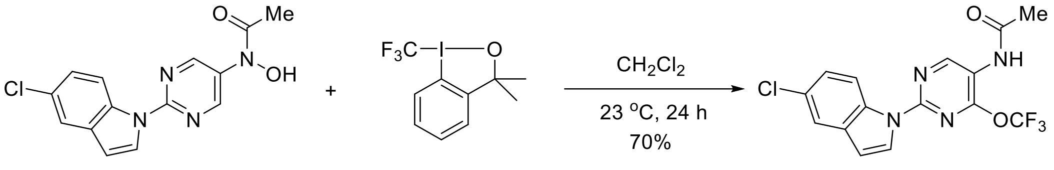

3b 1.2 equiv **4b**

A solution of *N*-(2-(5-chloro-1*H*-indol-1-yl)pyrimidin-5-yl)-*N*-hydroxyacetamide (**3b**) (50.0 mg, 0.165 mmol) and Togni reagent I (65.4 mg, 0.198 mmol, 1.20 equiv) in CH_2Cl_2 (16.5 mL, 0.010 M) was stirred at 23 °C under N_2 atmosphere for 24 h. The reaction mixture was concentrated *in vacuo*. The residue was purified by preparative TLC (thickness: 1 mm) using hexanes:EtOAc (3:2 (v/v)) for development (prep TLC was developed twice). The purification afforded the title compound as an off-white solid (42.8 mg, 0.115 mmol, 70% yield).

R_f = 0.61 (EtOAc:hexanes 1:1 (v/v)). NMR Spectroscopy: ^{1}H NMR (700 MHz, $CDCl_3$, 25 ºC, δ): 9.55 (s, 1H), 8.54 (d, *J* = 9.03 Hz, 1H), 8.13 (d, *J* = 3.44 Hz, 1H), 7.56 (d, *J* = 2.15 Hz, 1H), 7.28 (dd, *J* = 8.60, 2.15 Hz, 1H), 7.18 (s, 1H), 6.62 (d, *J* = 3.44 Hz, 1H), 2.29 (s, 3H). ^{13}C NMR (175 MHz, $CDCl_3$, 25 ºC, δ): 168.5, 152.6, 152.4, 151.1, 133.5, 132.5, 128.2, 127.1, 124.2, 120.6, 120.0 (q, *J* = 265.0 Hz), 116.8, 116.6, 107.0, 24.4. ^{19}F NMR (376 MHz, $CDCl_3$, 25 ºC, δ): –56.6 (s). Mass Spectrometry: HRMS (ESI-TOF) (m/z): calcd for $C_{15}H_{11}ClF_3N_4O_2$ ([M + H]$^+$), 371.0517, found, 371.0523.

N-(2-Methoxy-4-(trifluoromethoxy)pyrimidin-5-yl)acetamide (4c)

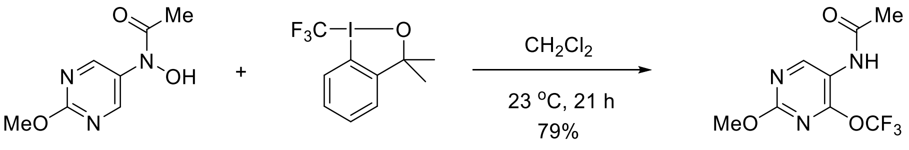

3c 1.2 equiv **4c**

A solution of *N*-hydroxy-*N*-(2-methoxypyrimidin-5-yl)acetamide (**3c**) (45.0 mg, 0.246 mmol) and Togni reagent I (97.4 mg, 0.295 mmol, 1.20 equiv) in CH_2Cl_2 (2.46 mL, 0.100 M) was stirred at 23 °C under N_2 atmosphere for 21 h. The reaction mixture was concentrated *in vacuo*. The residue was purified by preparative TLC (thickness: 1 mm) using hexanes:EtOAc (1:1 (v/v)) for development (prep TLC was developed twice). The purification afforded the title compound as a white solid (48.7 mg, 0.194 mmol, 79% yield).

R_f = 0.38 (EtOAc:hexanes 1:1 (v/v)). NMR Spectroscopy: ^1H NMR (700 MHz, $CDCl_3$, 25 ºC, δ): 9.22 (s, 1H), 7.46 (br. s, 1H), 3.96 (s, 3H), 2.22 (s, 3H). ^{13}C NMR (175 MHz, $CDCl_3$, 25 ºC, δ): 168.8, 160.4, 154.5, 154.3, 119.7 (q, J = 264.3 Hz), 115.3, 55.6, 24.0. ^{19}F NMR (376 MHz, $CDCl_3$, 25 ºC, δ): –56.6 (s). Mass Spectrometry: HRMS (ESI-TOF) (m/z): calcd for $C_8H_9F_3N_3O_3$ ([M + H]$^+$), 252.0591, found, 252.0593.

N-(2-(4-Chloro-3,5-dimethylphenoxy)-4-(trifluoromethoxy)pyrimidin-5-yl)acetamide (4d)

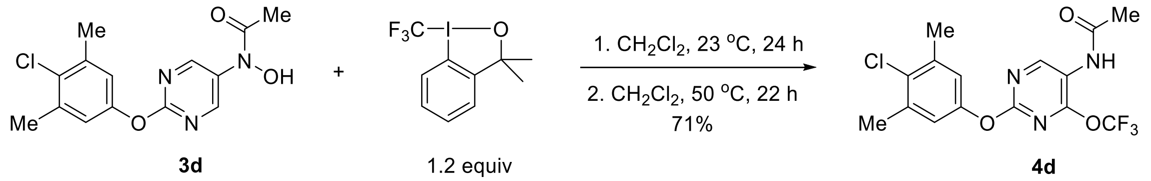

A solution of *N*-(2-(4-chloro-3,5-dimethylphenoxy)pyrimidin-5-yl)-*N*-hydroxyacetamide (**3d**) (30.5 mg, 0.0991 mmol) and Togni reagent I (39.3 mg, 0.119 mmol, 1.20 equiv) in CH_2Cl_2 (2.97 mL, 0.334 M) was stirred at 23 °C under N_2 atmosphere for 24 h. The reaction mixture was then stirred at 50 °C for 22 h. The reaction mixture was concentrated *in vacuo*. The residue was purified by preparative TLC (thickness: 1 mm) using hexanes:EtOAc (3:2 (v/v)) for development (prep TLC was developed once). The purification afforded the title compound as a white solid (26.5 mg, 0.0705 mmol, 71% yield).

R_f = 0.40 (EtOAc:hexanes 2:3 (v/v)). NMR Spectroscopy: ^1H NMR (400 MHz, $CDCl_3$, 25 ºC, δ): 9.32 (s, 1H), 7.16 (br. s, 1H), 6.92 (s, 2H), 2.38 (s, 6H), 2.25 (s, 3H). ^{13}C NMR (175 MHz, $CDCl_3$, 25 ºC, δ): 168.5, 159.3, 154.2, 153.5, 150.2, 137.8, 131.7, 121.3, 119.8 (q, J = 265.1 Hz), 116.7, 24.3, 21.0. ^{19}F NMR (376 MHz, $CDCl_3$, 25 ºC, δ): –56.7 (s). Mass Spectrometry: HRMS (ESI-TOF) (m/z): calcd for $C_{15}H_{14}ClF_3N_3O_3$ ([M + H]$^+$), 376.0670, found, 376.0677.

N-(2-(((8*S*,9*R*,13*R*,14*R*)-13-Methyl-17-oxo-7,8,9,11,12,13,14,15,16,17-decahydro-6*H*-cyclopenta[*a*]phenanthren-3-yl)oxy)-4-(trifluoromethoxy)pyrimidin-5-yl)acetamide (4e)

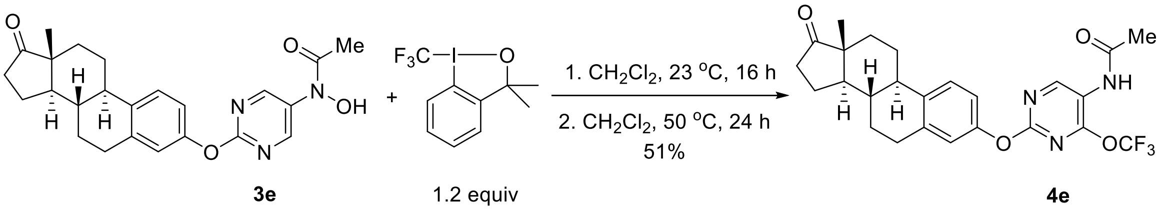

A solution of *N*-hydroxy-*N*-(2-(((8*S*,9*R*,13*R*,14*R*)-13-methyl-17-oxo-7,8,9,11,12,13,14,15,16,17-decahydro-6*H*-cyclopenta[*a*]phenanthren-3-yl)oxy)pyrimidin-5-yl)acetamide (**3e**) (25.0 mg, 0.0593 mmol) and Togni reagent I (23.5 mg, 0.0712 mmol, 1.20 equiv) in CH_2Cl_2 (0.593 mL, 0.100 M) was stirred at 23 °C under N_2 atmosphere for 16 h. The reaction mixture was then stirred at 50 °C for 24 h. The reaction mixture was concentrated *in vacuo*. The residue was purified by preparative TLC (thickness: 1 mm) using hexanes:EtOAc (3:2 (v/v)) for development (prep TLC was developed twice). The purification afforded the title compound as a white solid (14.9 mg, 0.0304 mmol, 51% yield).

R_f = 0.47 (EtOAc:hexanes 3:2 (v/v)). NMR Spectroscopy: ^{1}H NMR (700 MHz, $CDCl_3$, 25 ºC, δ): 9.32 (s, 1H), 7.32 (d, *J* = 8.60 Hz, 1H), 7.14 (s, 1H), 6.96 (dd, *J* = 8.39, 2.37 Hz, 1H), 6.92 (d, *J* = 2.58 Hz, 1H), 2.95–2.90 (m, 2H), 2.51 (dd, *J* = 18.93, 8.60 Hz, 1H), 2.45–2.39 (m, 1H), 2.31 (td, *J* = 11.19, 3.87 Hz, 1H), 2.25 (s, 3H), 2.19–2.11 (m, 1H), 2.09–2.05 (m, 1H), 2.04–2.00 (m, 1H), 1.97 (dt, *J* = 12.58, 3.17 Hz, 1H), 1.67–1.60 (m, 2H), 1.58 (dd, *J* = 12.48, 3.87 Hz, 1H), 1.56–1.43 (m, 3H), 0.92 (s, 3H). ^{13}C NMR (175 MHz, $CDCl_3$, 25 ºC, δ): 221.0, 168.4, 159.6, 154.3, 153.5, 150.5, 138.3, 137.4, 126.7, 121.5, 119.8 (q, *J* = 265.1 Hz), 118.7, 116.5, 50.6, 48.1, 44.3, 38.1, 36.0, 31.7, 29.6, 26.5, 25.8, 24.3, 21.7, 14.0. ^{19}F NMR (376 MHz, $CDCl_3$, 25 ºC, δ): –56.6 (s). Mass Spectrometry: HRMS (ESI-TOF) (m/z): calcd for $C_{25}H_{27}F_3N_3O_4$ ([M + H]$^+$), 490.1948, found, 490.1957.

Methyl (6-bromopyridin-3-yl)(trifluoromethoxy)carbamate (1d')

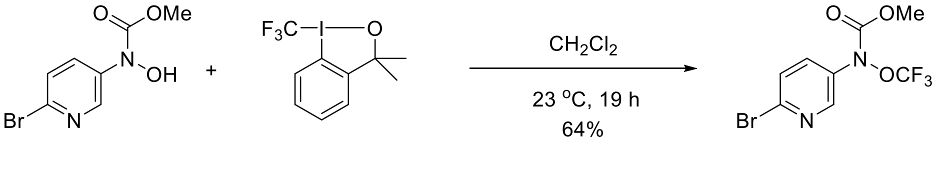

A solution of methyl (6-bromopyridin-3-yl)(hydroxy)carbamate (**1d**) (120.0 mg, 0.486 mmol) and Togni reagent I (192.4 mg, 0.583 mmol, 1.20 equiv) in CH_2Cl_2 (4.86 mL, 0.100 M) was stirred at 23 °C under N_2 atmosphere for 19 h. The reaction mixture was concentrated *in vacuo*. The residue was purified by preparative TLC (thickness: 1 mm) using hexanes:EtOAc (97:3 (v/v)) for development (prep TLC was developed four times). The band corresponding to 2-(2-iodophenyl)propan-2-ol was on the bottom of the band corresponding to the product, so only top of the band corresponding to the product was scraped of the PLC plate. The purification afforded the title compound as a white solid (97.4 mg, 0.309 mmol, 64% yield).

R_f = 0.49 (EtOAc:hexanes 1:9 (v/v)). NMR Spectroscopy: ^{1}H NMR (700 MHz, $CDCl_3$, 25 ºC, δ): 8.45 (br. s, 1H), 7.60 (m, 1H), 7.56 (d, *J* = 8.60 Hz, 1H), 3.90 (s, 3H). ^{13}C NMR (175 MHz, $CDCl_3$, 60 ºC, δ): 155.2, 145.9, 141.2, 137.3, 133.9, 128.5, 122.7 (q, *J* = 263.7 Hz), 55.3. ^{19}F NMR (376 MHz, $CDCl_3$, 25 ºC, δ): –66.2(s). Mass Spectrometry: HRMS (ESI-TOF) (m/z): calcd for $C_8H_7BrF_3N_2O_3$ ([M + H]$^+$), 314.9587, found, 314.9592.

5-Bromo-6-methoxy-2-(trifluoromethoxy)pyridin-3-amine (2a')

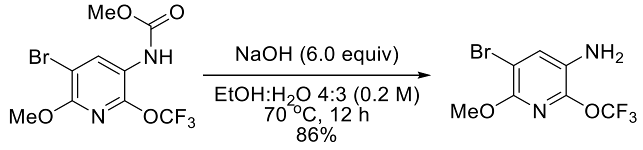

To a stirred suspension of (5-bromo-6-methoxypyridin-3-yl)(hydroxy)carbamate (0.370 g, 1.07 mmol, 1.00 equiv) and sodium hydroxide (0.257 g, 6.43 mmol, 6.00 equiv) in EtOH:H_2O 4:3 (6.32 mL, 0.200 M) was heated at 70 °C overnight, cool to -20 °C, diluted with water and the crystals formed were filtered off to afford the deprotection of methyl carbamates as pure slightly light yellow solid (0.263 g, 0.92 mmol, 86% yield).

R_f = 0.69 (EtOAc:hexanes 3:7 (v/v)). NMR Spectroscopy: ^{1}H NMR (500 MHz, $(CD_3)_2SO$, 25 ºC, δ): 7.55 (s, 1H), 5.17 (s, 2H), 3.77 (s, 3H). ^{13}C NMR (175 MHz, $(CD_3)_2SO$, 25 ºC, δ): 147.73, 137.95, 131.06, 129.42, 120.01 (q, J = 257.3 Hz), 102.74, 54.17. ^{19}F NMR (376 MHz, $CDCl_3$, 25 ºC, δ): –56.9 (s). Mass Spectrometry: HRMS (ESI-TOF) (m/z): calcd for $C_7H_7BrF_3N_2O_2$ ($[M + H]^+$), 288.9618, found, 288.9629.

***N*-(5-Bromo-6-methoxy-2-(trifluoromethoxy)pyridin-3-yl)-2-(thiophen-2-yl)acetamide (5a)**

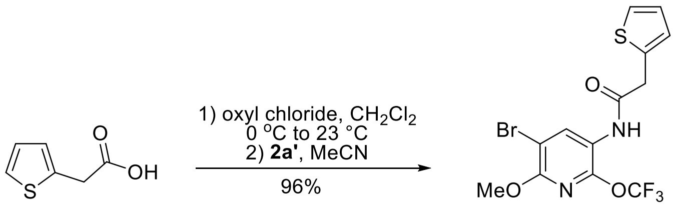

To a solution of 2-(thiophen-2-yl)acetic acid (49.5 mg, 0.348 mmol, 2.0 equiv), DMF (1 drop) and DCM (3.50 mL, 0.100 M) at 0 °C under N_2 atmosphere was added dropwise oxalyl chloride (44.2 mg, 0.348 mmol, 2.0 equiv) via a syringe. The resulting mixture was stirred at 23 °C for 2 h, concentrated to afford crude acide chloride, which was used in the subsequent step without further purification.

The above crude acid chloride was dissolved in CH_3CN (1.5 mL, 0.0232 M) and added to a solution of 5-bromo-6-methoxy-2-(trifluoromethoxy)pyridin-3-amine (**2a′**) (50 mg, 0.174 mmol, 1.0 equiv) in CH_3CN (3.5 mL, 0.0497 M) at 23 °C under N_2 atmosphere. The reaction mixture was stirred at 23 °C for another 2 h, concentrated *in vacuo* and purified by chromatography on silica gel, eluting with hexanes:EtOAc (5:1 (v/v)), to afford the title compound as a yellow solid (69.0 mg, 0.167 mmol, 96% yield).

R_f = 0.20 (EtOAc:hexanes 1:4 (v/v)). NMR Spectroscopy: ^{1}H NMR (500 MHz, $CDCl_3$, 25 ºC, δ): 8.87 (s, 1H), 7.40 (br. s., 1H), 7.34 (dd, J = 5.04, 1.07 Hz, 1H), 7.09–7.03 (m, 2H), 3.97 (s, 2H), 3.92 (s, 3H). ^{13}C NMR (125 MHz, $CDCl_3$, 25 ºC, δ): 168.1, 154.2, 142.2, 136.5, 134.8, 128.3, 127.9,

126.6, 119.9 (q, J = 261.8 Hz), 116.8, 102.6, 55.2, 38.4. ^{19}F NMR (376 MHz, $CDCl_3$, 25 ºC, δ): –57.1(s). Mass Spectrometry: HRMS (ESI-TOF) (m/z): calcd for $C_{13}H_{11}BrF_3N_2O_3S$ ([M + H]$^+$), 412.9600, found, 412.9605.

***tert*-Butyl 4-(3-(2-methoxy-5-((methoxycarbonyl)amino)-6-(trifluoromethoxy)pyridin-3-yl)benzoyl)piperazine-1-carboxylate (6a)**

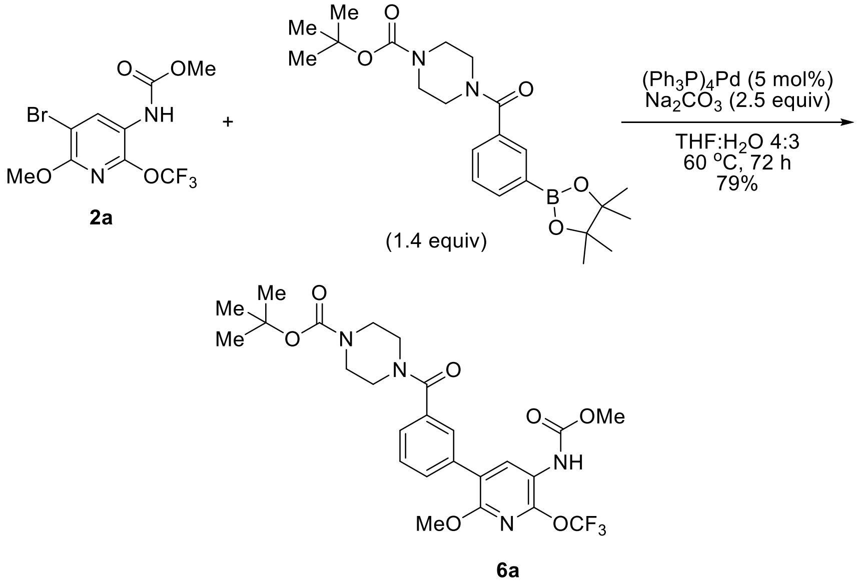

Methyl (5-bromo-6-methoxy-2-(trifluoromethoxy)pyridin-3-yl)carbamate (**2a**) (0.0600 g, 0.170 mmol, 1.00 equiv), methyl tert-butyl 4-(3-(4,4,5,5-tetramethyl-1,3,2-dioxaborolan-2-yl)benzoyl)piperazine-1-carboxylate (0.103 g, 0.240 mmol, 1.40 equiv), Na_2CO_3 (0.287 g, 0.430 mmol, 2.5 equiv), THF:H_2O 4:3 (1.01 mL, 0.200 M), and palladium-tetrakis(triphenylphosphine) (0.002 g, 0.020 mmol, 0.0500 equiv) were degassed via three freeze-pump-thaw cycles. The resulting mixture was heated at 60 °C for 72 hours and then allowed to cool to room temperature after which water was added (twice the volume of THF:H_2O 4:3 used). The mixture was then extracted with ethyl acetate (twice the volume of THF:H_2O 4:3 used) and the organic extracts was dried with $MgSO_4$, filtered purified by preparative TLC (thickness: 1 mm) using EtOAc:hexanes (2:3 (v/v)) for development (prep TLC was developed three times) to afford the pure cross-coupled product as a white solid (0.077 g, 0.14 mmol, 79% yield).

R_f = 0.20 (EtOAc:hexanes 2:3 (v/v)). NMR Spectroscopy: ^{1}H NMR (700 MHz, $CDCl_3$, 25 ºC, δ): 8.47 (br. s, 1H), 7.64–7.59 (m, 2H), 7.47 (t, J = 8.17 Hz, 1H), 7.40 (d, J = 7.31 Hz, 1H), 6.68 (br. s, 1H), 3.90 (s, 3H), 3.80 (s, 3H), 3.76 (br. s., 2H), 3.60–3.34 (m, 6H), 1.47 (s, 9H). ^{13}C NMR (175 MHz, $CDCl_3$, 25 ºC, δ): 170.4, 154.7, 154.1, 142.5, 135.7, 135.5, 133.4, 130.7, 128.9, 127.9, 126.7, 121.0, 120.2 (q, J = 261.2 Hz), 116.7, 80.5, 54.4, 52.9, 47.7, 43.8, 42.2, 28.5. ^{19}F NMR (376 MHz, $CDCl_3$, 25 ºC, δ): –56.4 (s). Mass Spectrometry: HRMS (ESI-TOF) (m/z): calcd for $C_{25}H_{29}F_3N_4O_7$ ([M + H]$^+$), 555.2061, found, 555.2060.

Methyl 2-((5-bromo-6-methoxy-2-(trifluoromethoxy)pyridin-3-yl)amino)benzoate (5a)

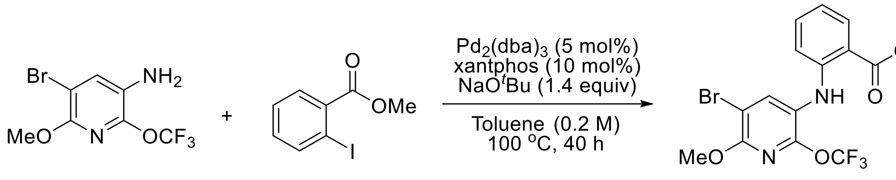

2a' **7a**

A mixture of 5-bromo-6-methoxy-2-(trifluoromethoxy)pyridin-3-amine (**2a′**) (50.0 mg, 0.200 mmol, 1.20 equiv), methyl 2-iodobenzoate (44.5 mg, 0.170 mmol, 1.00 equiv), xantphos (10.0 mg, 0.0170 mmol, 0.100 equiv), NaO*ᵗ*Bu (2.30 mg, 0.238 mmol, 1.40 equiv), and $Pd_2(dba)_3$ (7.80 mg, 0.00850 mmol, 0.05 equiv) in toluene (0.850 mL, 0.200 M) was stirred at 100 °C under N_2 for 40 hours and then allowed to cool to room temperature. The reaction was purified by preparative TLC (thickness: 1 mm) using EtOAc:hexanes (1:10 (v/v)) for development (prep TLC was developed two times) to afford title compound as a colorless liquid (61.0 mg, 0.145 mmol, 85% yield).

R_f = 0.71 (EtOAc:hexanes 1:10 (v/v)). NMR Spectroscopy: ^{1}H NMR (700 MHz, $CDCl_3$, 25 ºC, δ): 9.23 (s, 1H), 8.01–7.97 (m, 1H), 7.95 (s, 1H), 7.36 (t, *J* = 7.74 Hz, 1H), 6.87 (d, *J* = 8.60 Hz, 1H), 6.82– 6.79 (m, 1H), 3.99 (s, 3H), 3.92 (s, 3 H). ^{13}C NMR (125 MHz, $CDCl_3$, 25 ºC, δ): 168.9, 154.5, 147.2, 146.8, 139.7, 134.5, 131.9, 120.2 (q, *J* = 260.9 Hz), 119.7, 118.3, 113.4, 112.8, 102.1, 52.3, 52.1. ^{19}F NMR (376 MHz, $CDCl_3$, 25 ºC, δ): –56.5 (s). Mass Spectrometry: HRMS (ESI-TOF) (m/z): calcd for $C_{15}H_{13}BrF_3N_2O_4$ ($[M + H]^+$), 421.0005, found, 421.0010.

Methyl (6-methoxy-5-((4-methoxyphenyl)ethynyl)-2-(trifluoromethoxy)pyridin-3-yl)carbamate (8a)

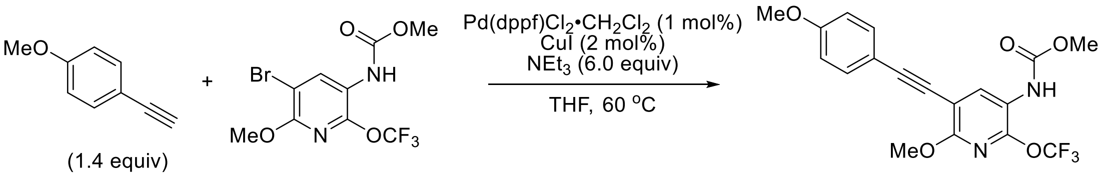

(1.4 equiv) **2a** **8a**

Under N_2 atmosphere methyl (5-bromo-6-methoxy-2-(trifluoromethoxy)pyridin-3-yl)carbamate (**2a**) (0.0500 g, 0.140 mmol, 1.00 equiv), 1-ethynyl-4-methoxybenzene (0.0270 g, 0.200 mmol, 1.40 equiv), copper(I) iodide (0.550 mg, 2.90 μmol, 0.0200 equiv), and [1,1′-bis(diphenylphosphino)ferrocene]dichloropalladium(II), complex with dichloromethane (1.20 mg, 1.40 μmol, 0.0200 equiv in THF (0.72 mL, 0.200 M), was heated at 60 °C overnight and then allowed to cool to room temperature after which water was added (twice the volume of THF). The mixture was then extracted with ethyl acetate (twice the volume of THF:H_2O 4:3 used) and the organic extracts was dried with $MgSO_4$, filtered purified by preparative TLC (thickness: 1 mm) using EtOAc:hexanes (1:9 (v/v)) for development (prep TLC was developed three times) to afford

the pure cross-coupled product as a white solid (0.045 g, 0.11 mmol, 78% yield).

R$_f$ = 0.38 (EtOAc:hexanes 3:16 (v/v)). NMR Spectroscopy: ^{1}H NMR (700 MHz, $CDCl_3$, 25 ºC, δ): 8.54 (br. s, 1H), 7.50–7.46 (m, 2H), 6.90–6.85 (m, 2H), 6.60 (br. s, 1H), 3.96 (s, 3H), 3.83 (s, 3H), 3.81 (s, 3H). ^{13}C NMR (175 MHz, $CDCl_3$, 25 ºC, δ): 160.0, 157.3, 153.9, 142.1, 135.4, 133.4, 120.3 (q, J = 261.6 Hz), 116.1, 115.0, 114.1, 105.3, 95.0, 81.7, 55.5, 54.8, 52.9. ^{19}F NMR (376 MHz, $CDCl_3$, 25 ºC, δ): –56.4 (s). Mass Spectrometry: HRMS (ESI-TOF) (m/z): calcd for $C_{18}H_{16}F_3N_2O_5$ ($[M + H]^+$), 397.1006, found, 397.1002.

Procedure for the large scale trifluoromethoxylation

Methyl (5-bromo-6-methoxy-2-(trifluoromethoxy)pyridin-3-yl)carbamate (2a)

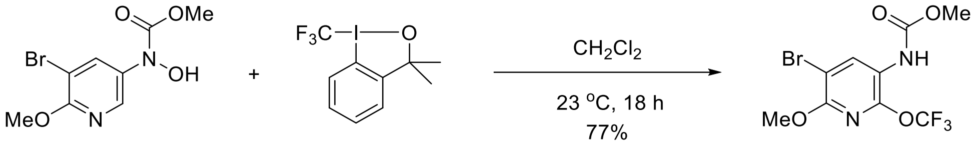

1a 1.2 equiv **2a**

A solution of methyl (5-bromo-6-methoxypyridin-3-yl)(hydroxy)carbamate (**1a**) (1.39 g, 5.00 mmol) and Togni reagent I (1.98 g, 6.00 mmol, 1.20 equiv) in CH_2Cl_2 (50.0 mL, 0.100 M) was stirred at 23 °C under N_2 atmosphere for 18 h. The reaction mixture was concentrated *in vacuo.* The residue was purified by flash chromatography eluting with hexanes and then with EtOAc:hexanes (3:17 (v/v)).The purification afforded the title compound (2.51 g, 9.05 mmol, 95% yield), which was spectroscopically identical to the compound prepared according to the standard procedure (*vide supra*).

O-Trifluoromethylation in the presence of a radical trap

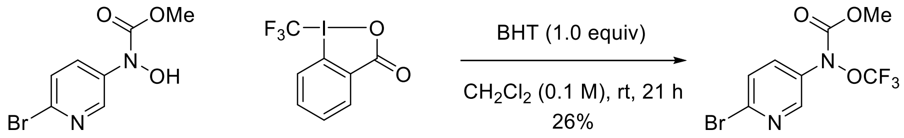

1d 1.2 equiv **1d'**

A solution of methyl (6-bromopyridin-3-yl)(hydroxy)carbamate (**1d**) (12.4 mg, 0.0502 mmol, 1.00 equiv), BHT (11.0 mg, 0.0500 mmol, 1.00 equiv) and Togni reagent I (19.8 mg, 0.0600 mmol, 1.2 equiv) in CH_2Cl_2 (0.500 mL) was stirred at 23 °C under N_2 atmosphere for 21 h. Trifluorotoluene (6.14 µL) and $CDCl_3$ (0.250 mL) were added and the reaction mixture was analyzed by ^{19}F NMR. The ^{19}F NMR analysis indicated that the yield of *O*-trifluoromethylation of **1d** in the presence of BHT (26%) was much lower than in the absence of the radical trap (83%).

O-CF_3 Migration in the presence of a radical trap

w/o BHT:

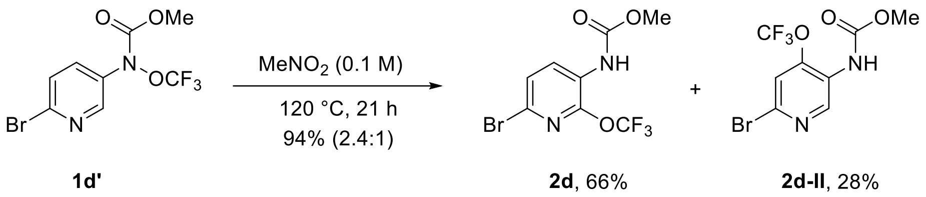

with BHT:

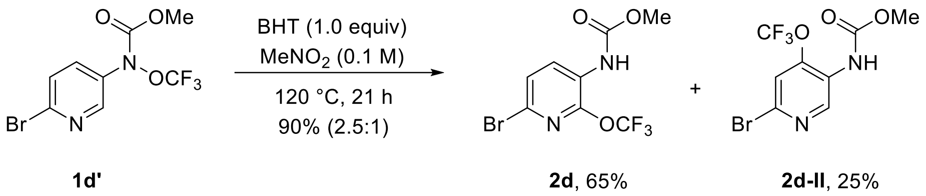

Reaction without BHT: Under N_2 atmosphere, a solution of methyl (6-bromopyridin-3-yl)(trifluoromethoxy)carbamate (9.45 mg, 30.0 µmol) (**1d'**) in $MeNO_2$ (0.300 mL, 0.100 M) was heated at 120 °C for 21 h. Trifluorotoluene (3.68 µL) and $CDCl_3$ (0.400 mL) were added and the reaction mixture was analyzed by ^{19}F NMR. The ^{19}F NMR analysis indicated that the yield of OCF_3-migration reaction was 94% (2.4:1).

Reaction with BHT: Under N_2 atmosphere, a solution of methyl (6-bromopyridin-3-yl)(trifluoromethoxy)carbamate (**1d'**) (9.45 mg, 30.0 µmol, 1.00 equiv) and BHT (11.0 mg, 50.0 µmol, 1.00 equiv) in $MeNO_2$ (0.300 mL, 0.100 M) was heated at 120 °C for 21 h. Trifluorotoluene (3.68 µL) and $CDCl_3$ (0.400 mL) were added and the reaction mixture was analyzed by ^{19}F NMR. The ^{19}F NMR analysis indicated that the yield of OCF_3-migration reaction was 90% (2.5:1).

Spectroscopic Data

^{1}H NMR ($(CD_3)_2SO$, 25 ºC) of **1a**

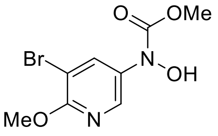

1a

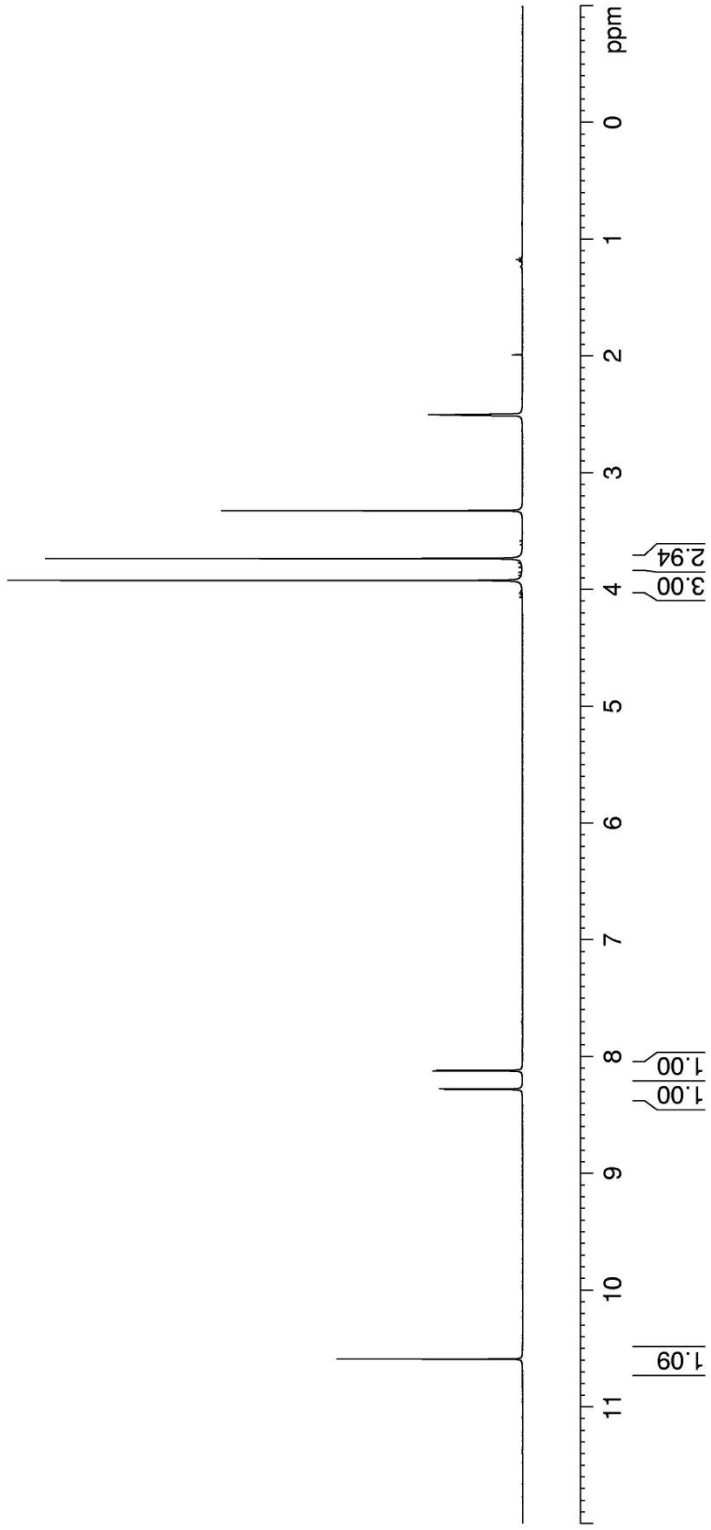

^{13}C NMR ($(CD_3)_2SO$, 25 ºC) of **1a**

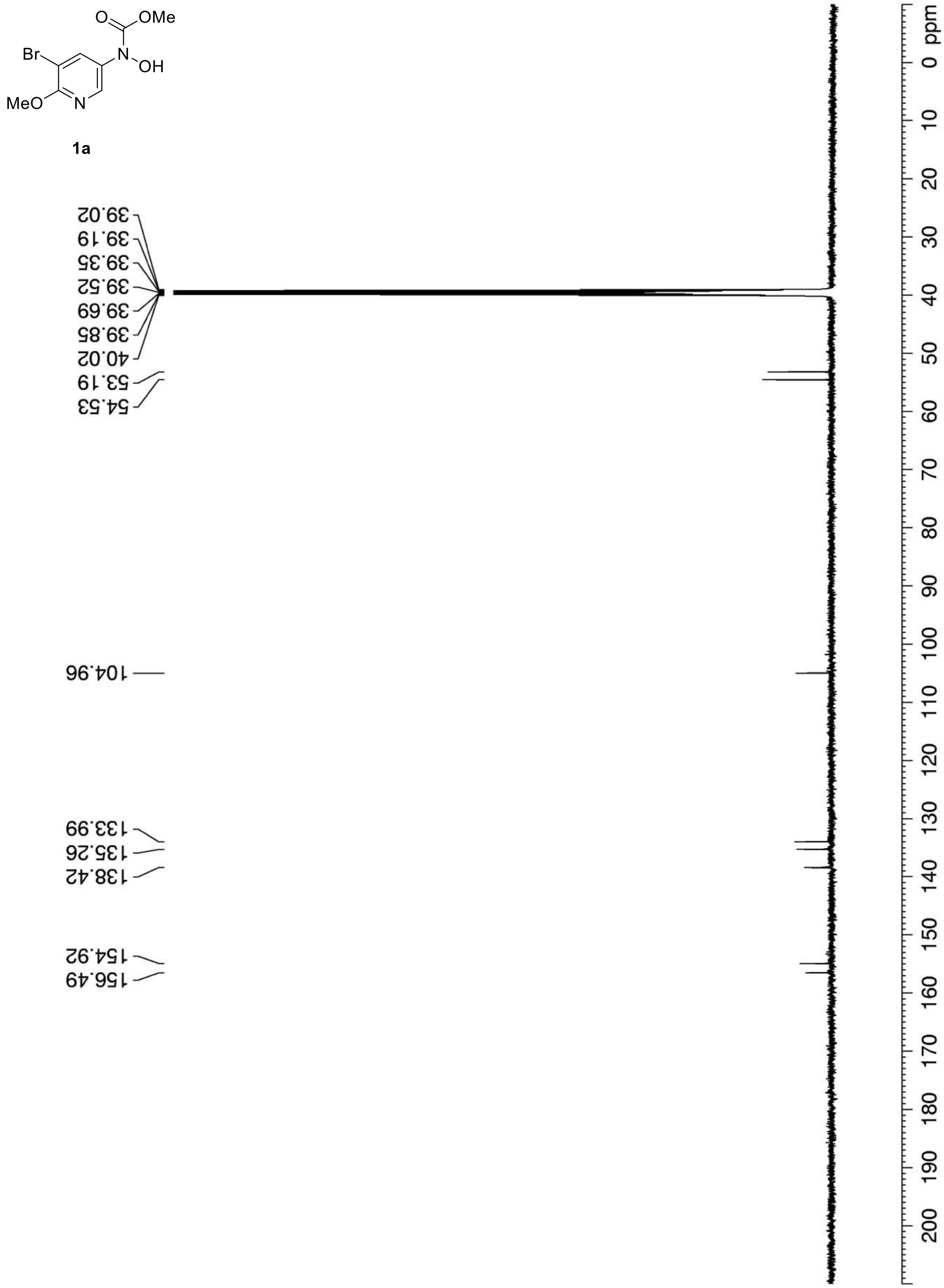

^{1}H NMR ($(CD_3)_2SO$, 25 ºC) of **S1**

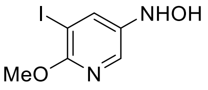

S1

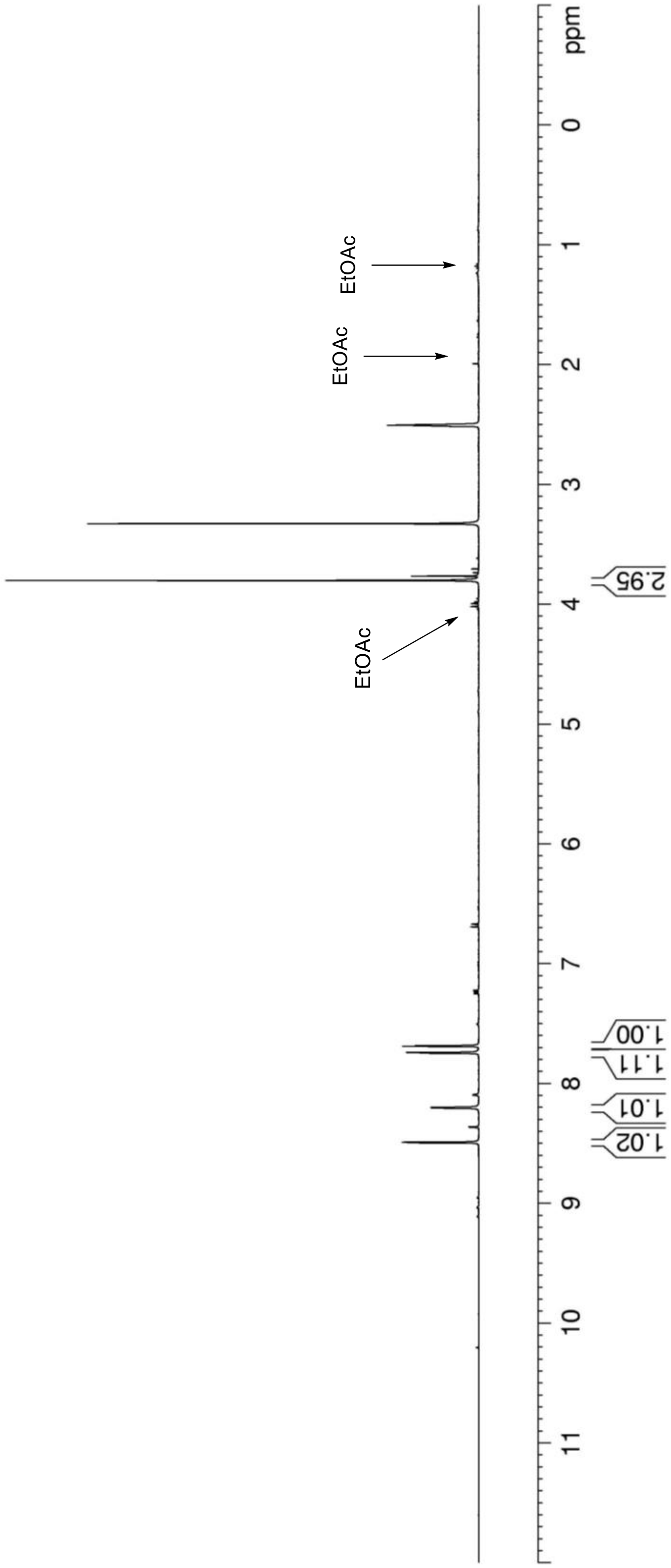

^{13}C NMR ((CD$_3$)$_2$SO, 25 ºC) of **S1**

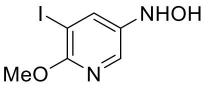

S1

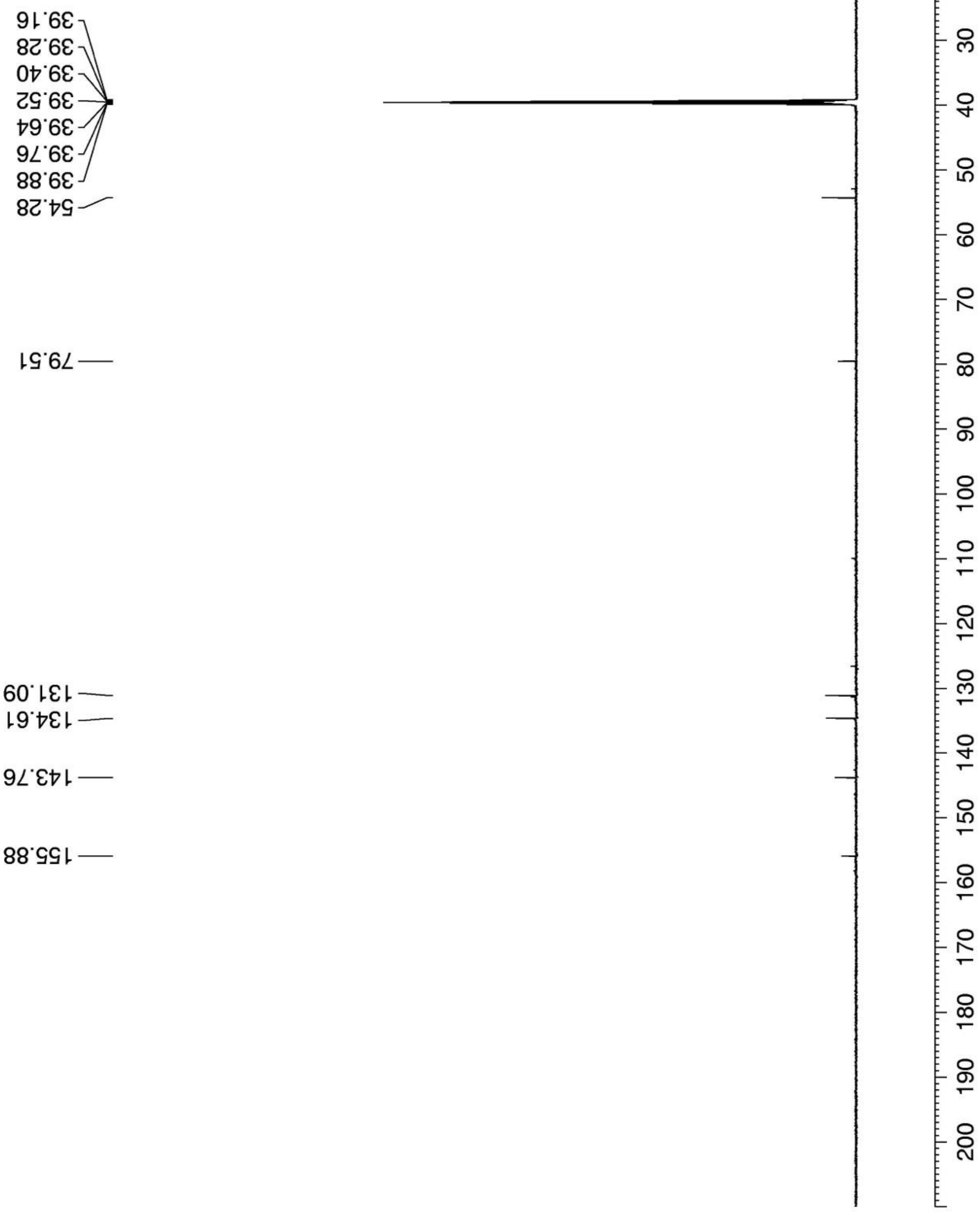

^{1}H NMR ((CD_3)$_2$SO, 25 ºC) of **1b**

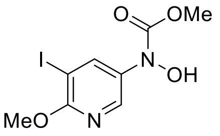

1b

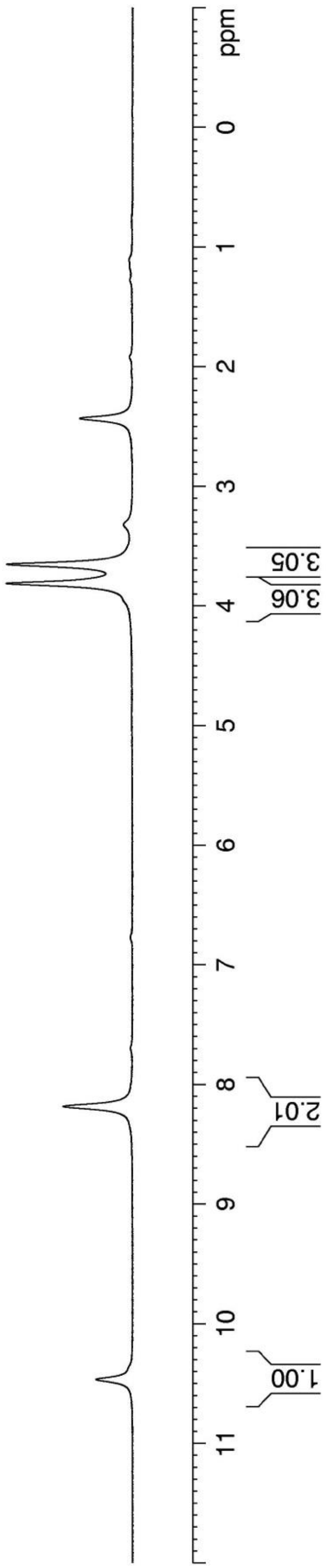

^{13}C NMR ((CD$_3$)$_2$SO, 25 ºC) of **1b**

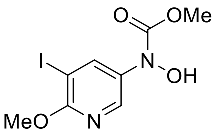

1b

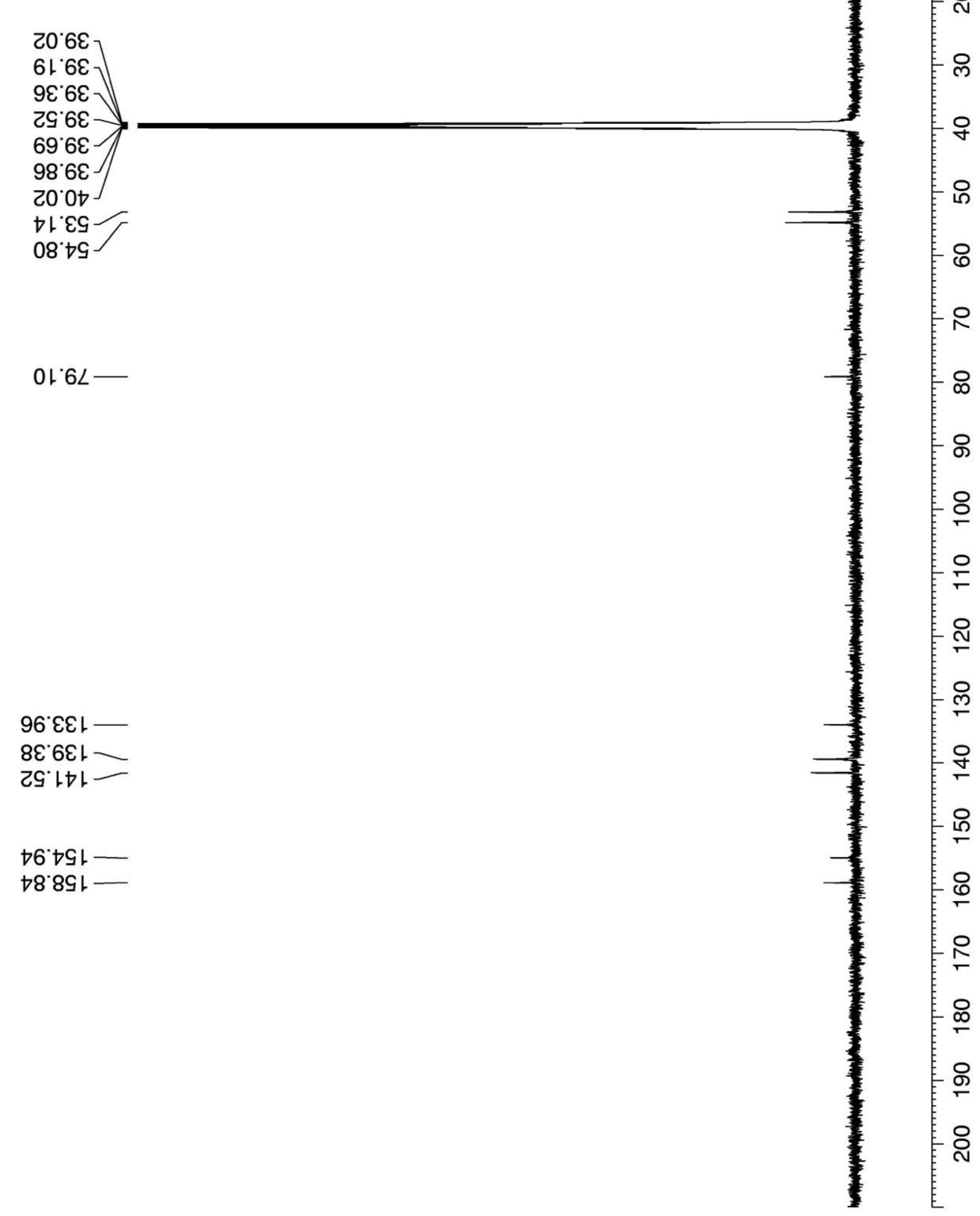

^{1}H NMR ($(CD_3)_2SO$, 25 ºC) of **S2**

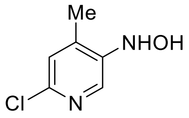

S2

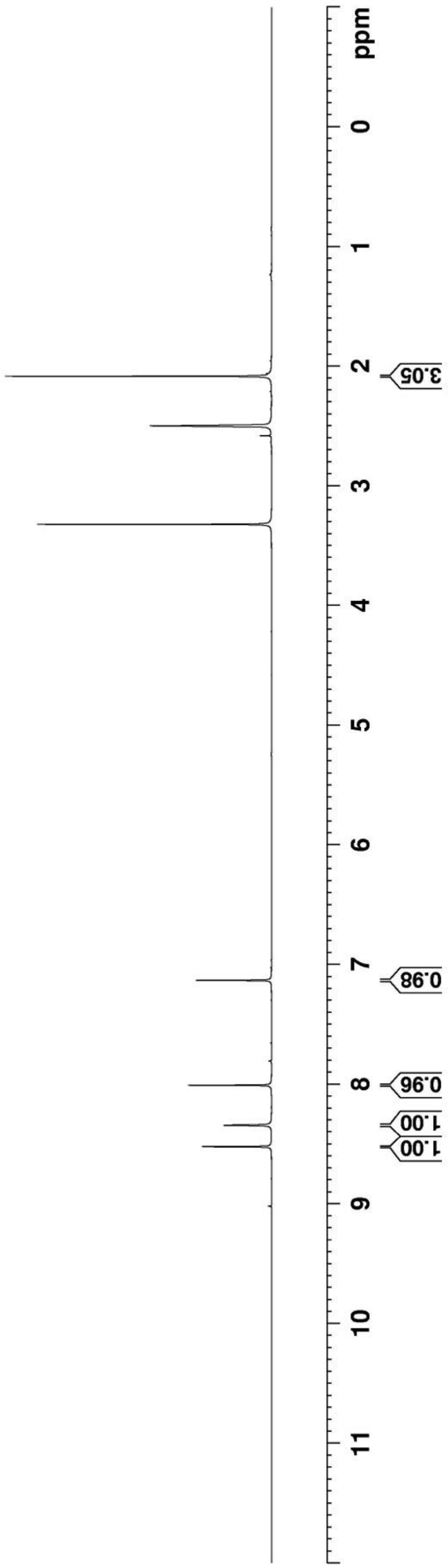

^{13}C NMR $((CD_3)_2SO$, 25 ºC) of **S2**

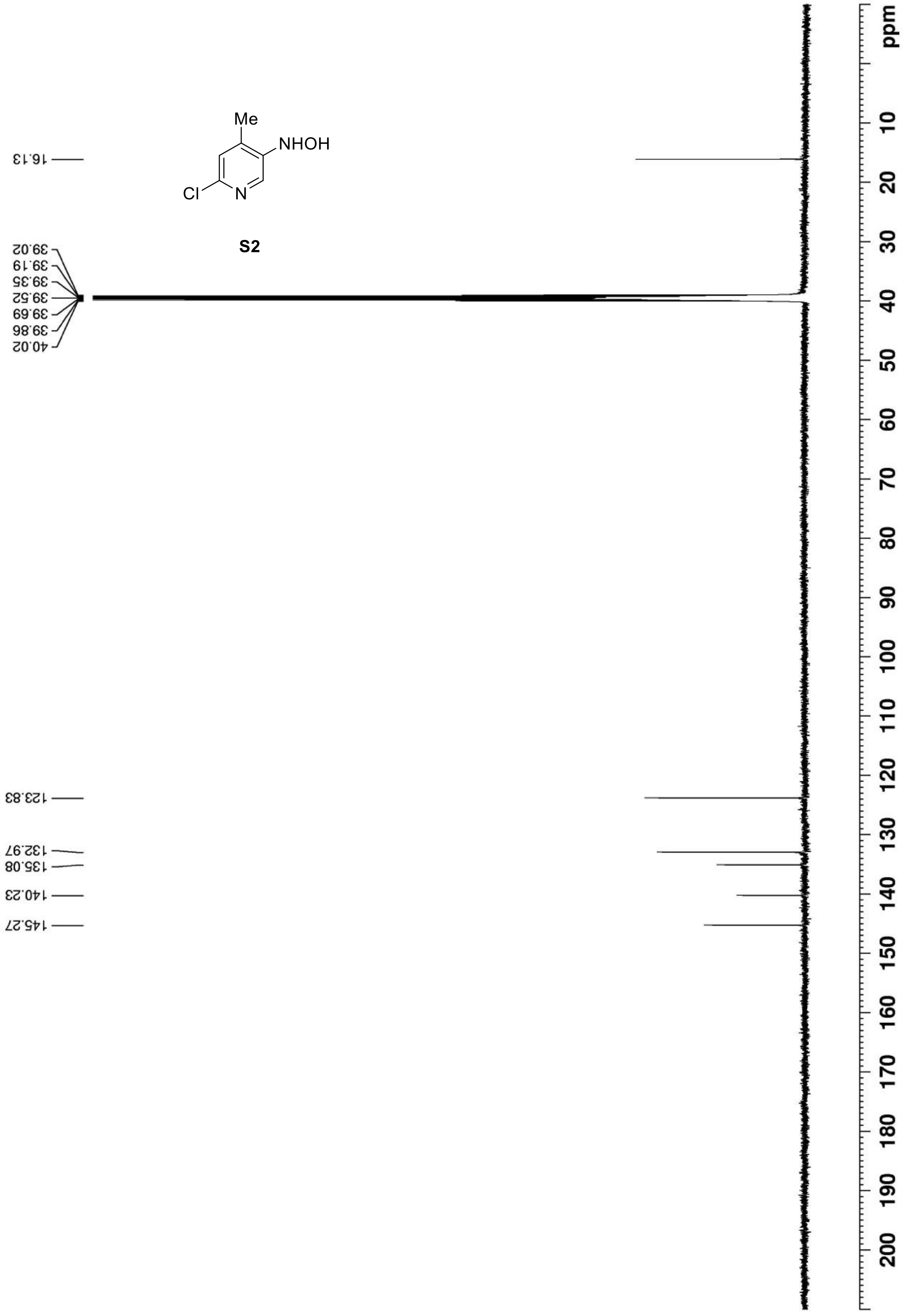

^{1}H NMR ($(CD_3)_2SO$, 25 ºC) of **1c**

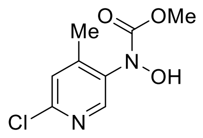

1c

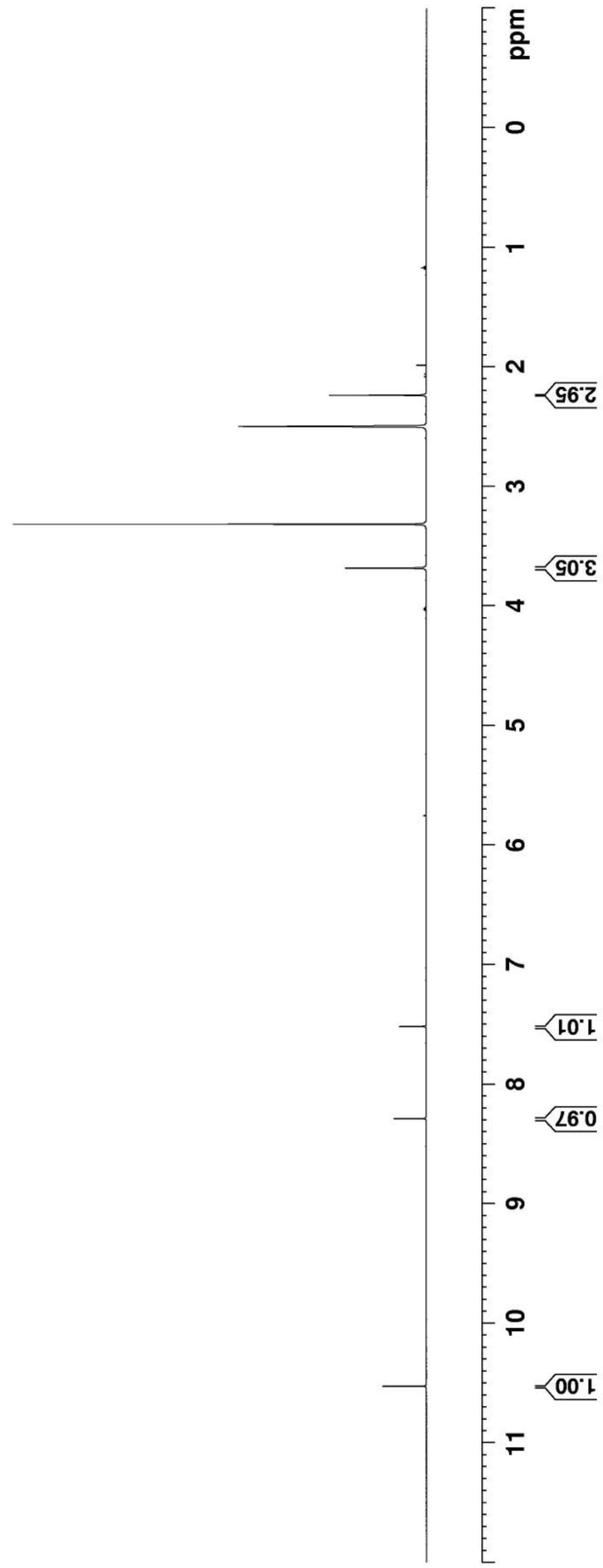

^{13}C NMR ($(CD_3)_2SO$, 25 ºC) of **1c**

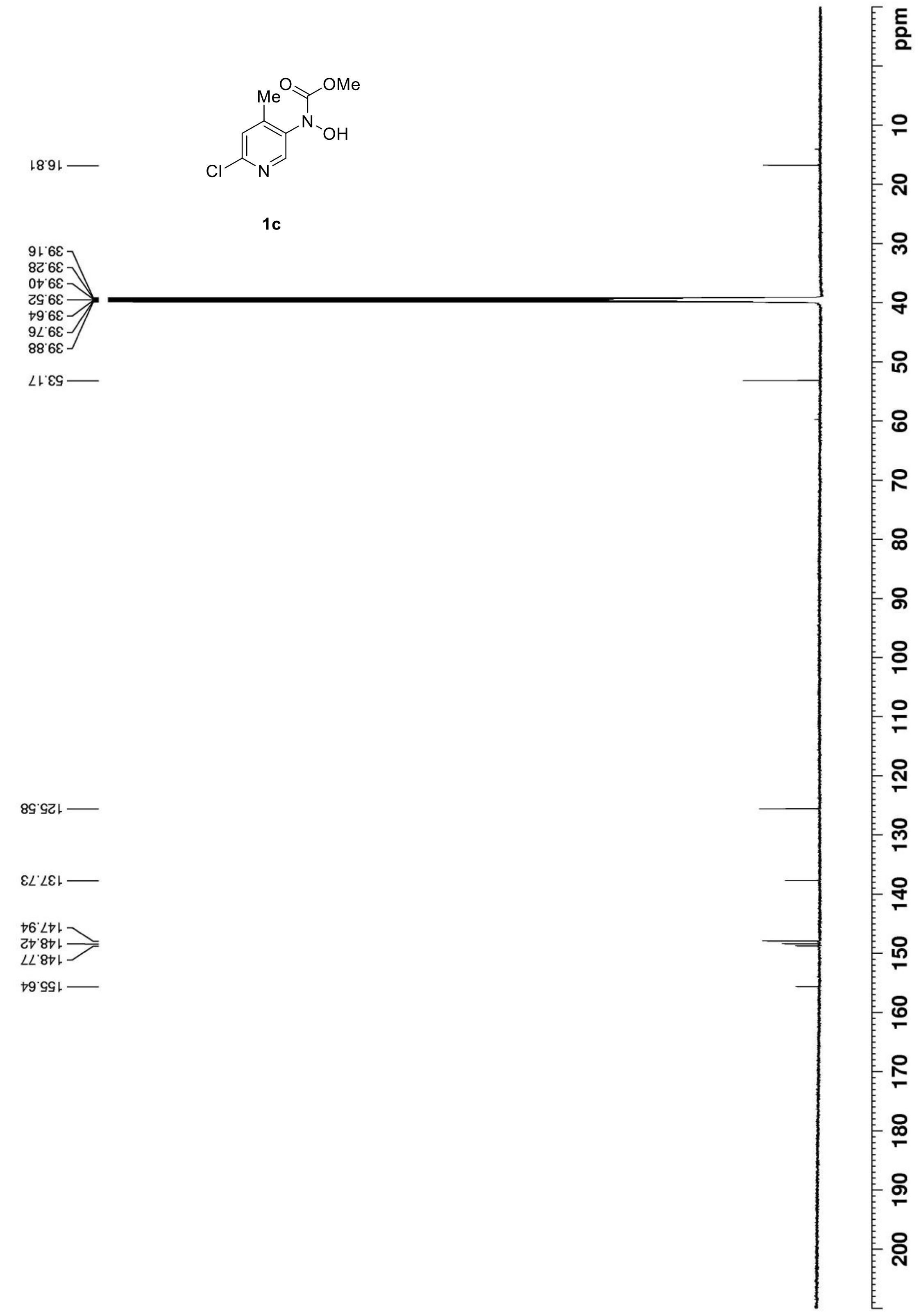

^{1}H NMR ($(CD_3)_2SO$, 25 ºC) of **S3**

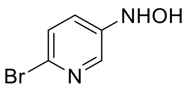

S3

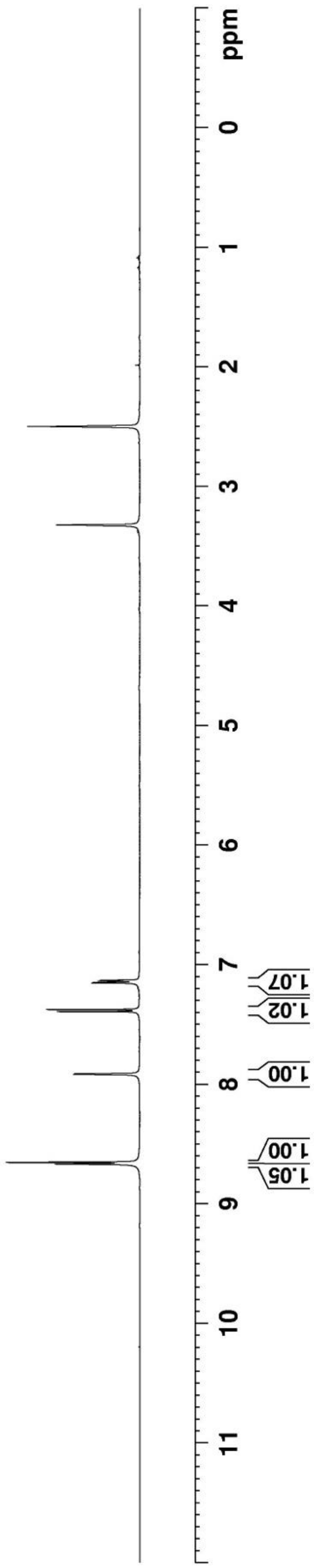

^{13}C NMR $((CD_3)_2SO$, 25 ºC) of **S3**

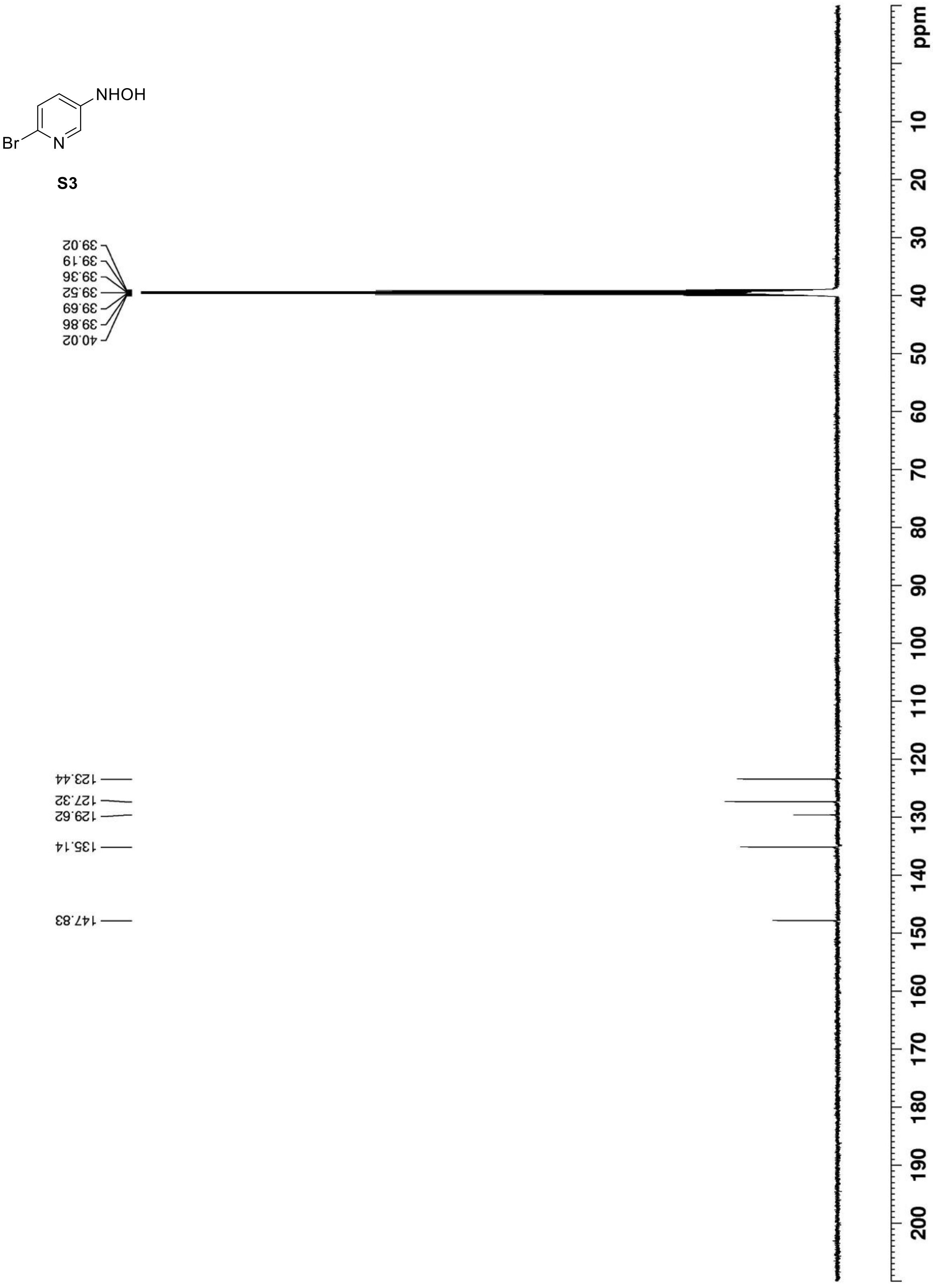

^{1}H NMR ((CD_3)$_2$SO, 25 ºC) of **1d**

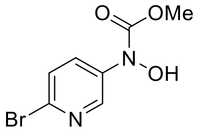

1d

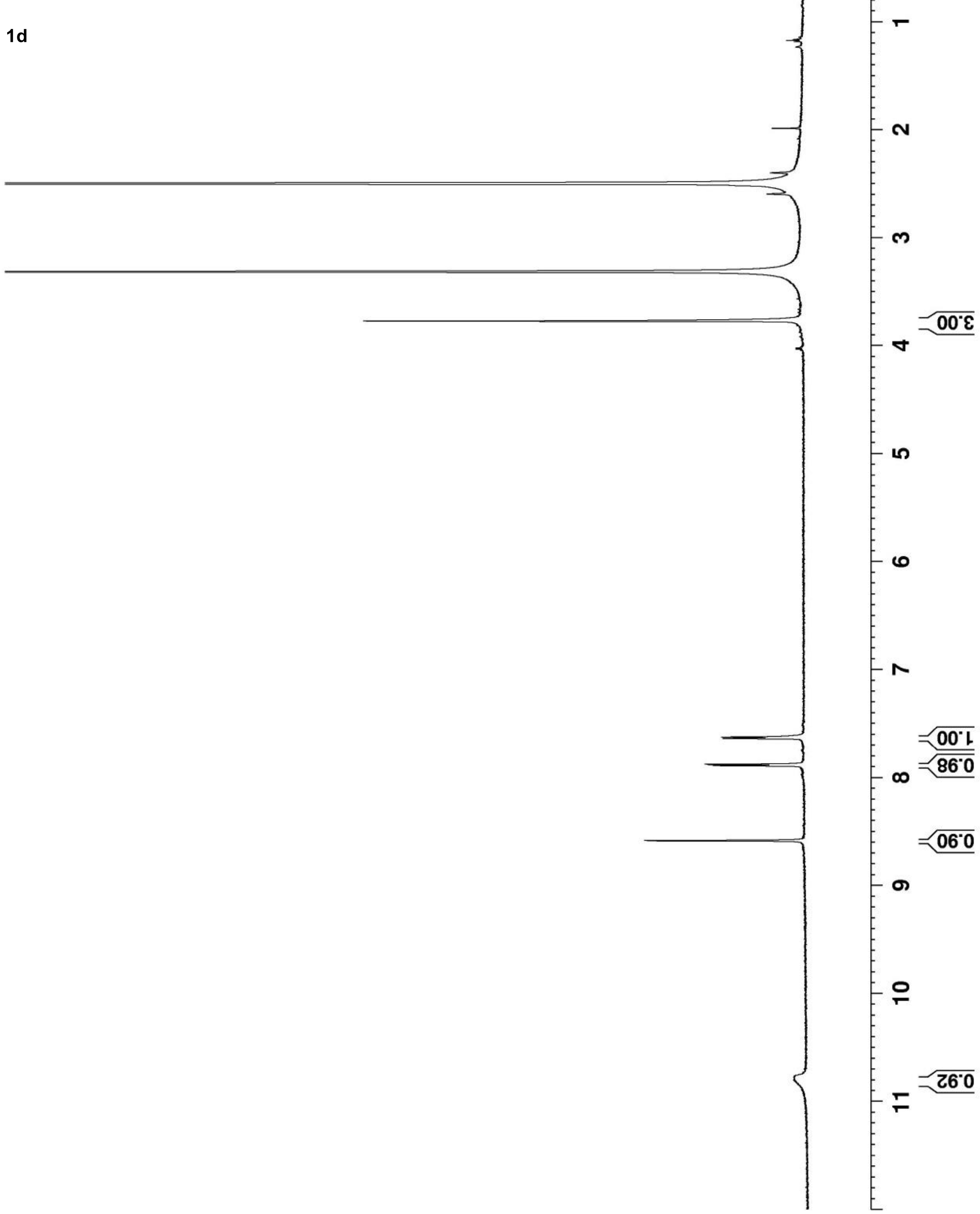

^{13}C NMR (($(CD_3)_2SO$, 25 ºC) of **1d**

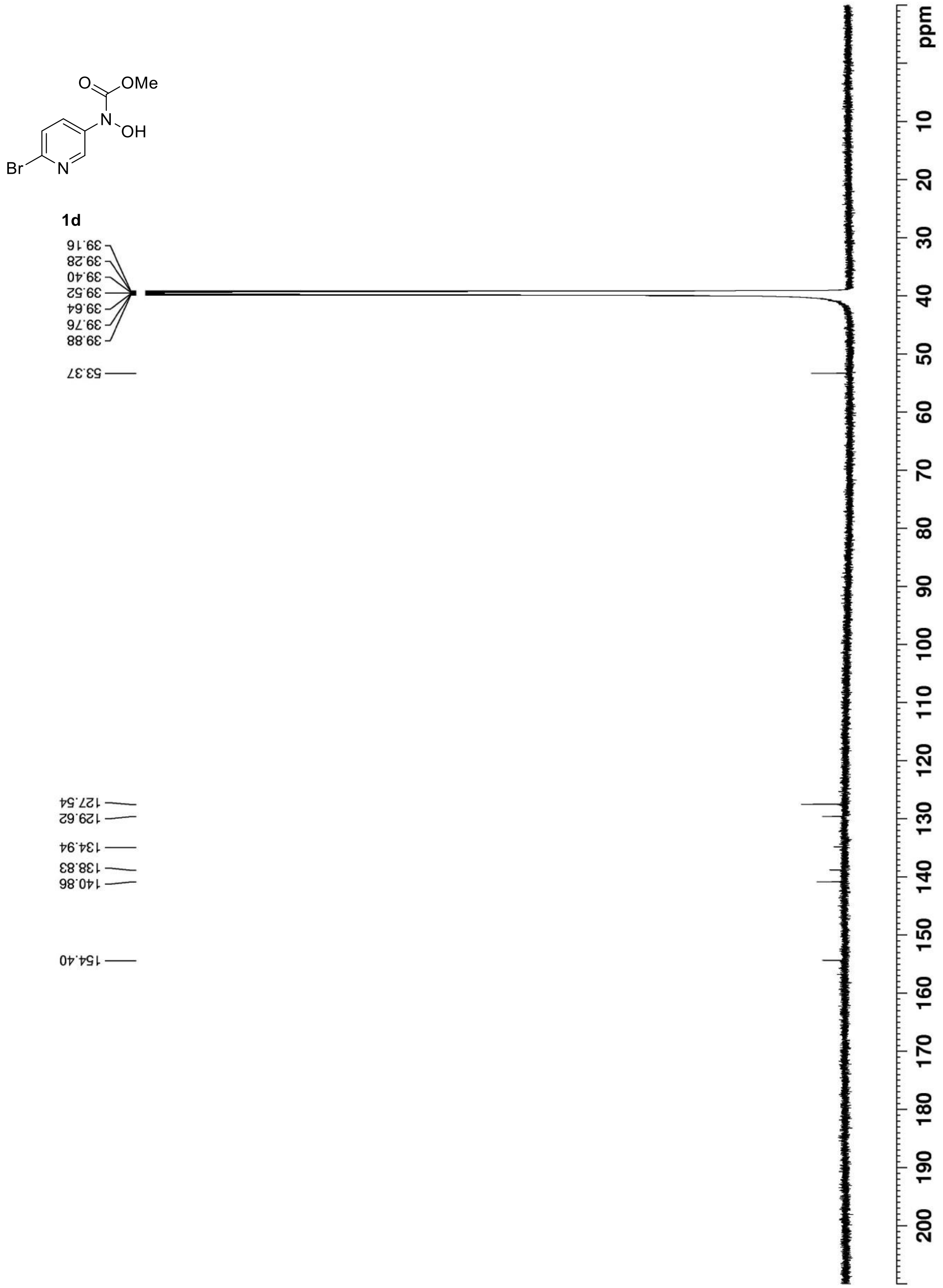

^{1}H NMR ($(CD_3)_2SO$, 25 ºC) of **1e**

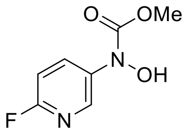

1e

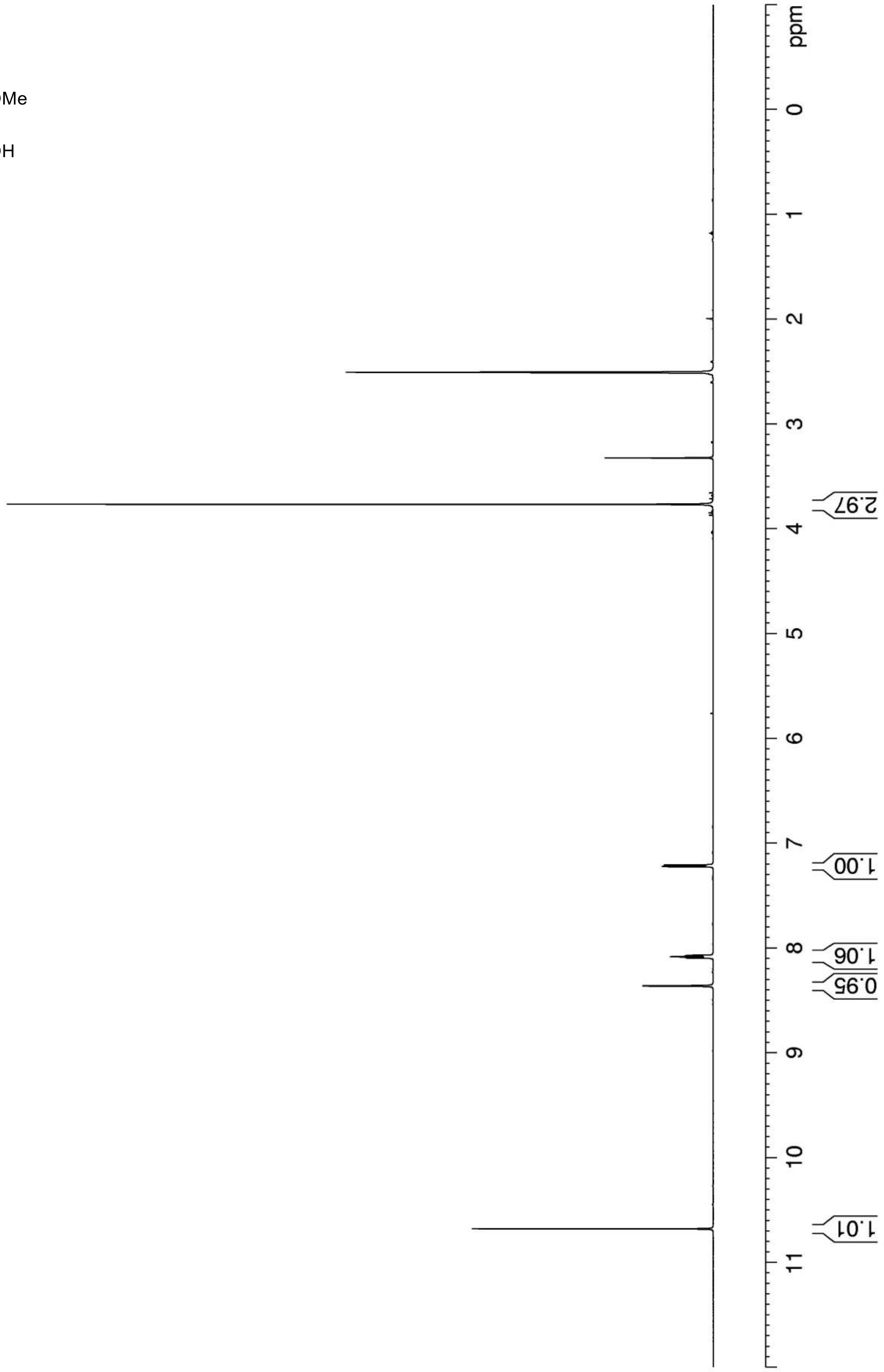

^{13}C NMR (($(CD_3)_2SO$, 25 ºC) of **1e**

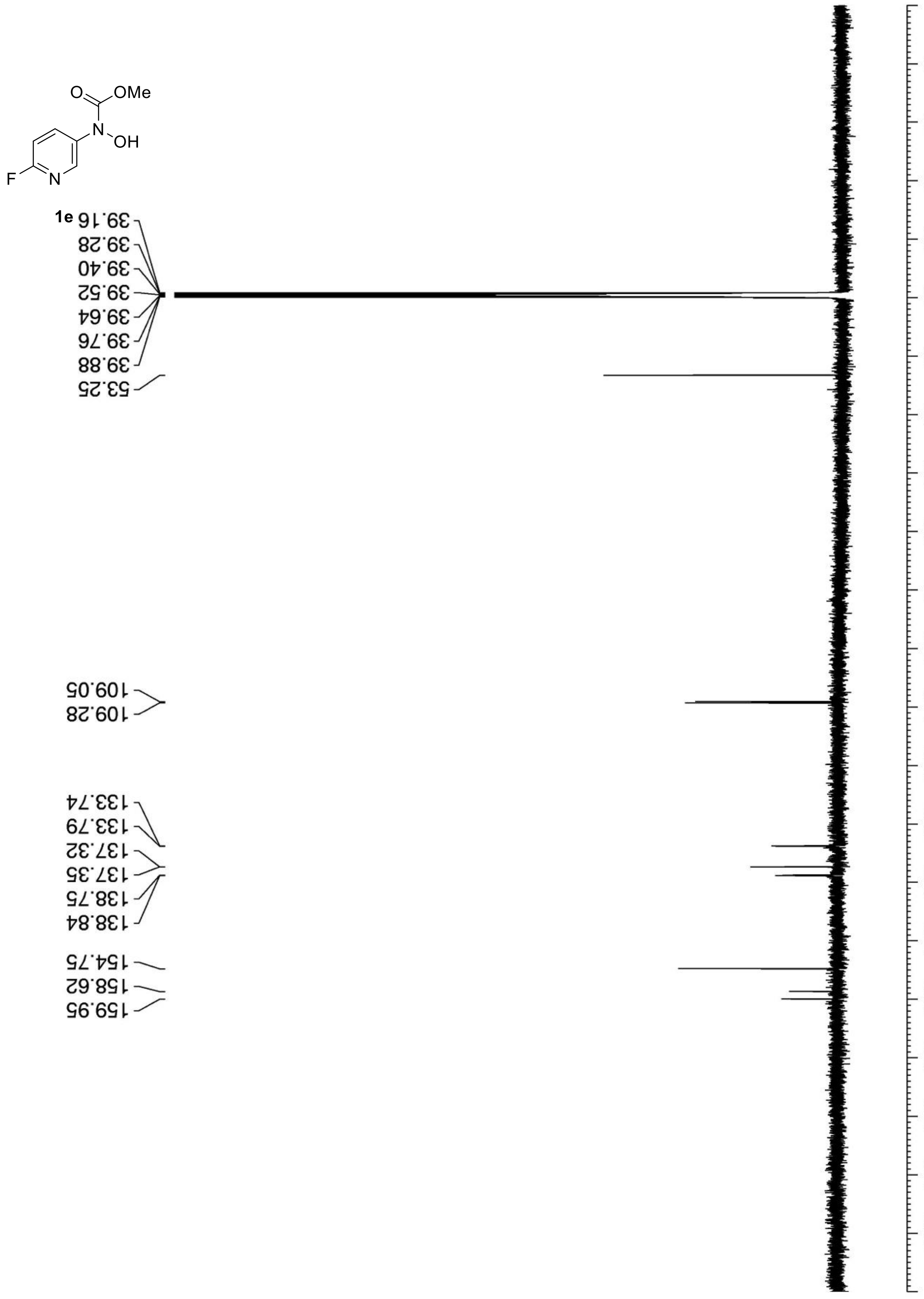

^{19}F NMR ((CD$_3$)$_2$SO, 25 ºC) of **1e**

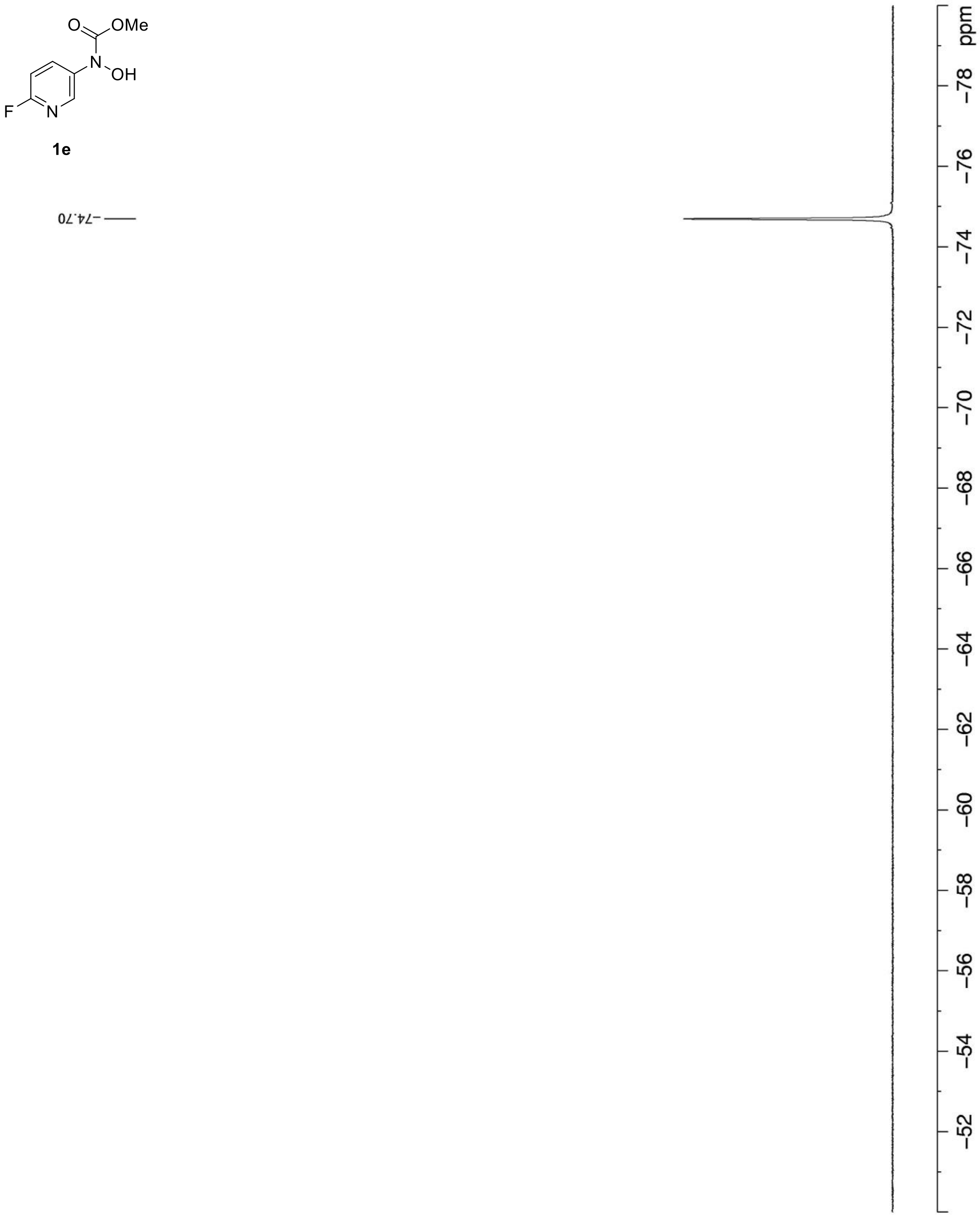

^{1}H NMR ($(CD_3)_2SO$, 25 ºC) of **S4**

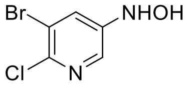

S4

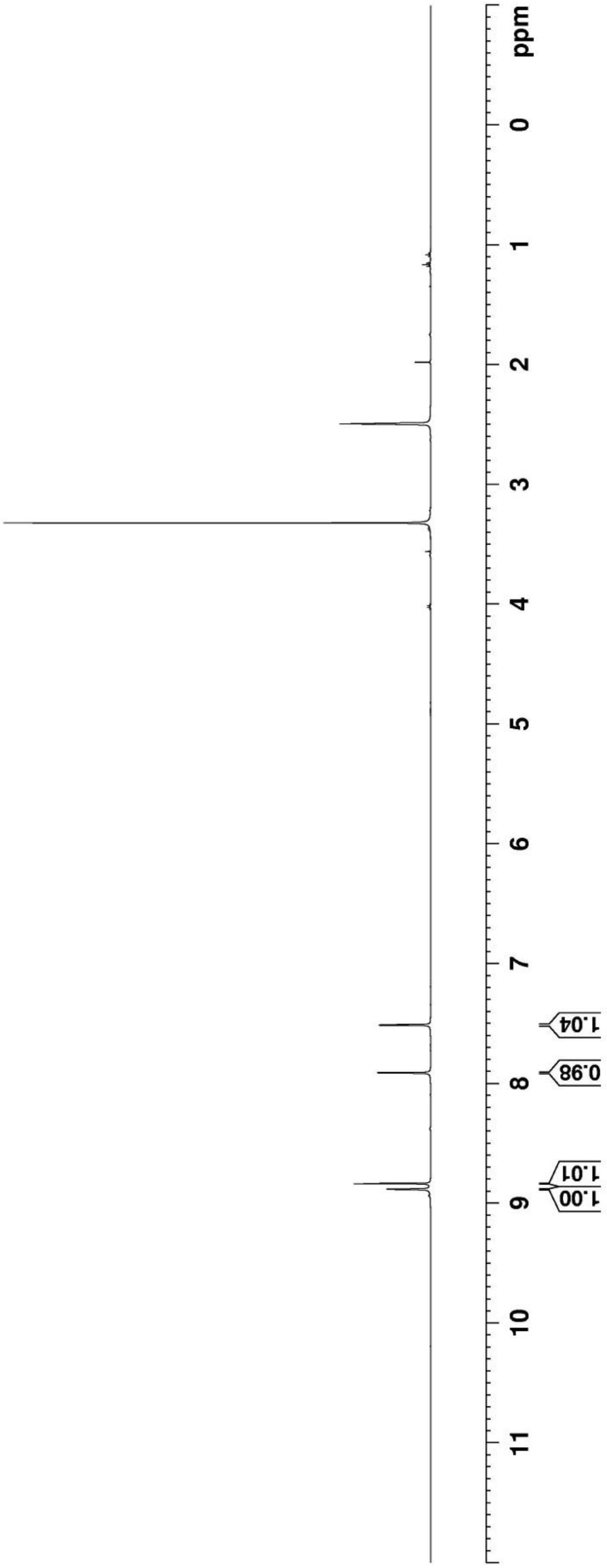

^{13}C NMR ((CD_3)$_2$SO, 25 ºC) of **S4**

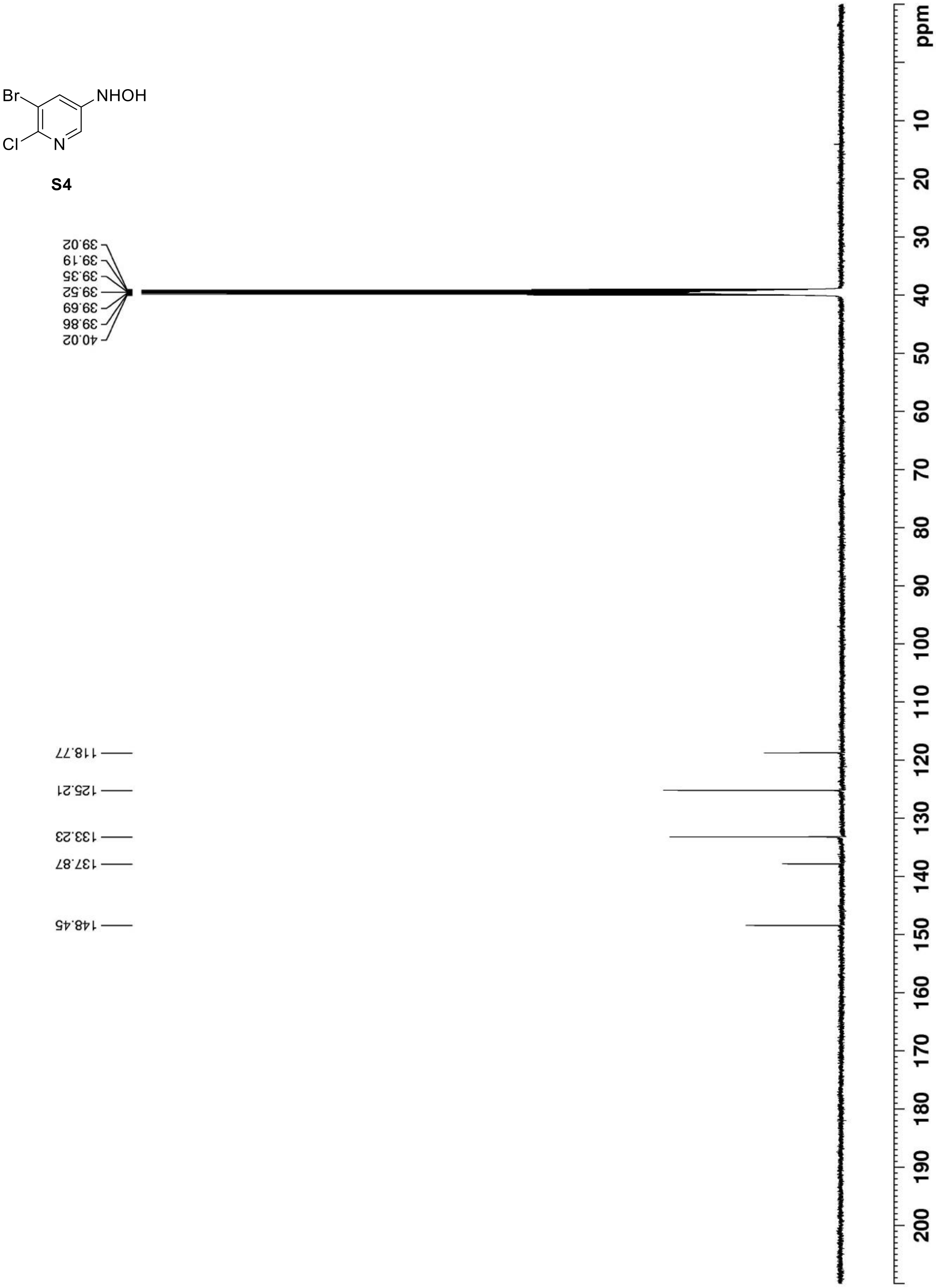

S4

^{1}H NMR ($(CD_3)_2SO$, 25 ºC) of **1f**

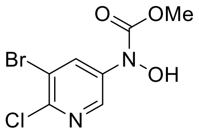

1f

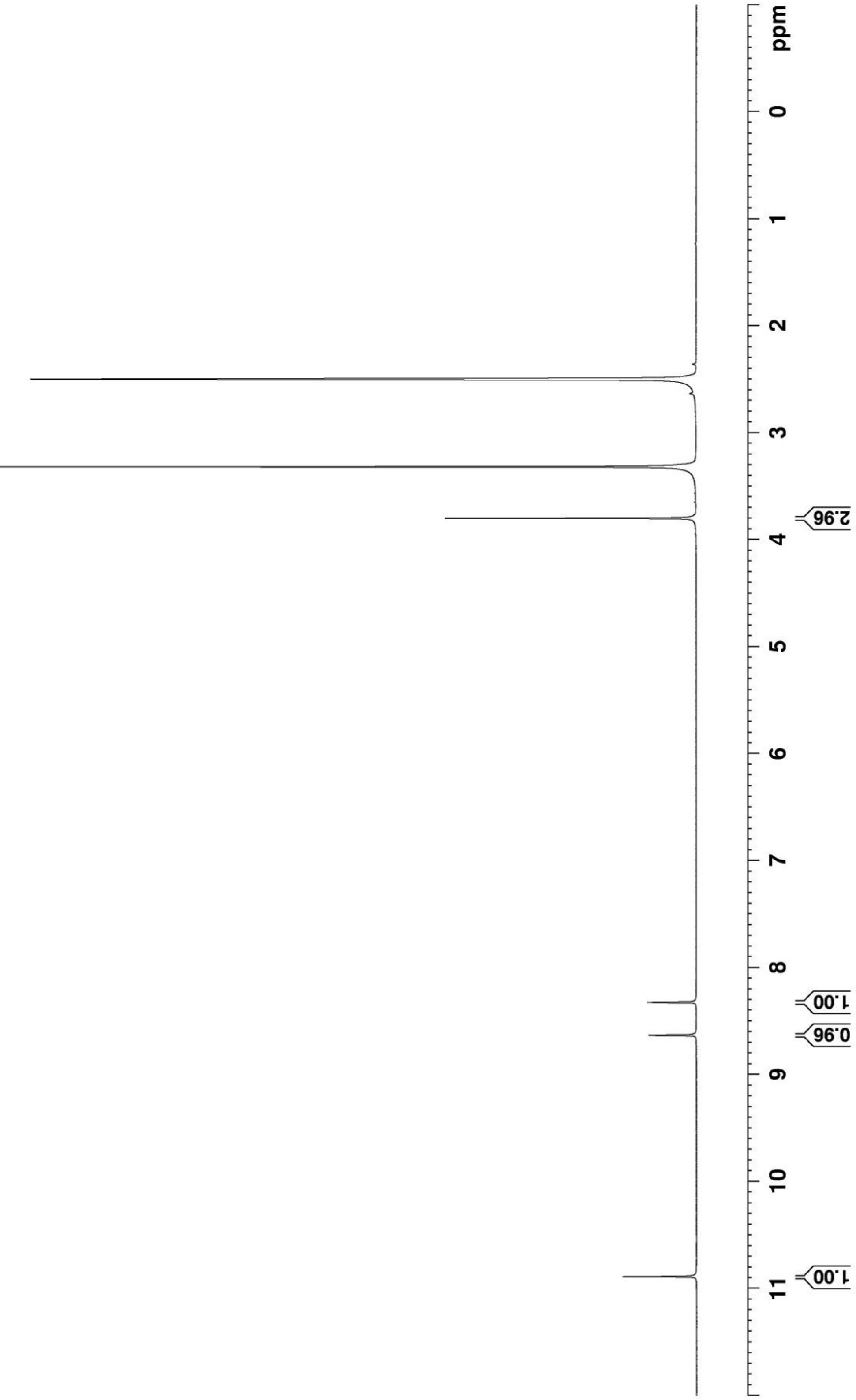

^{13}C NMR ($(CD_3)_2SO$, 25 ºC) of **1f**

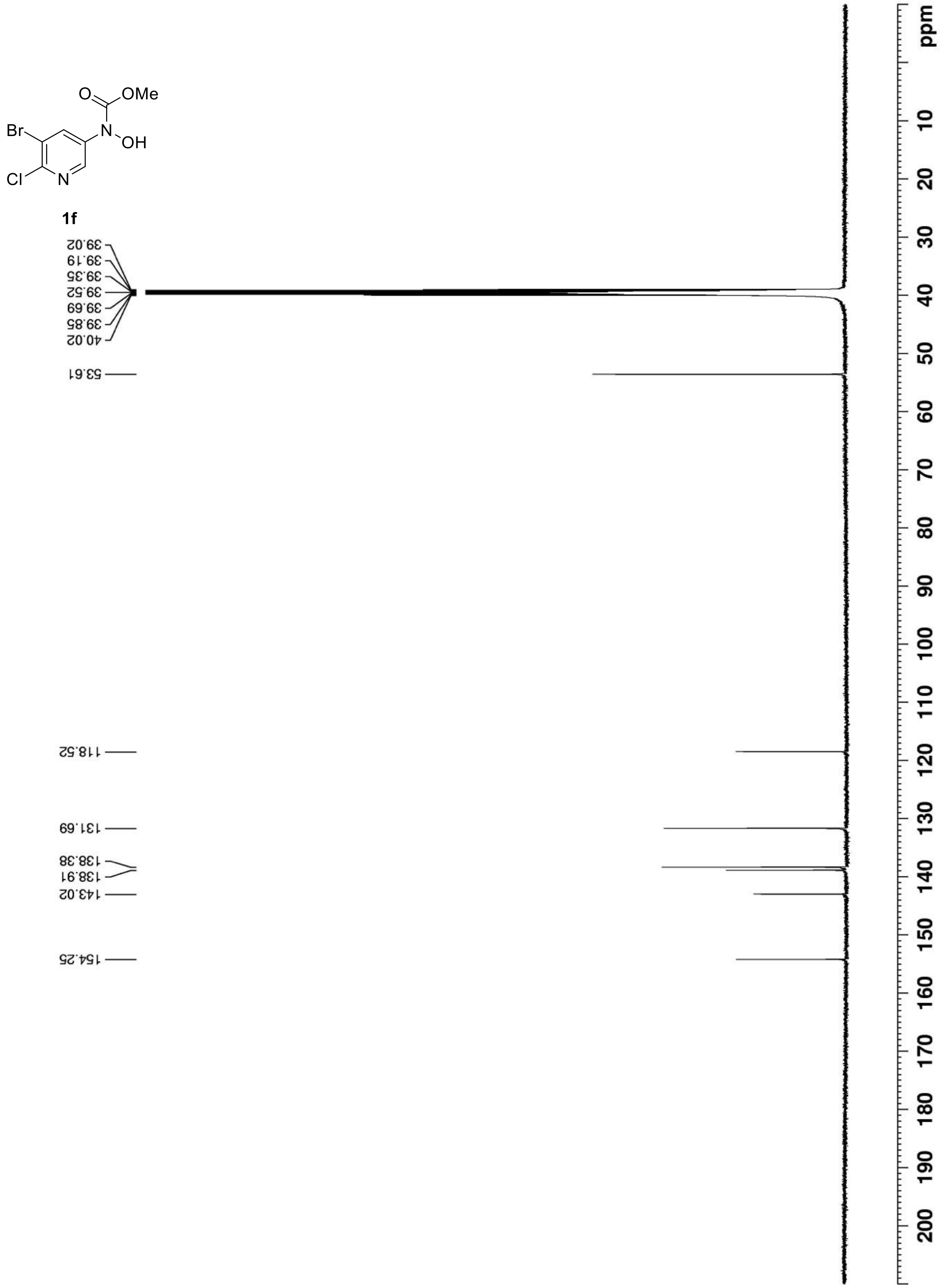

^{1}H NMR ($(CD_3)_2SO$, 25 ºC) of **S5**

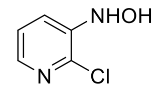

S5

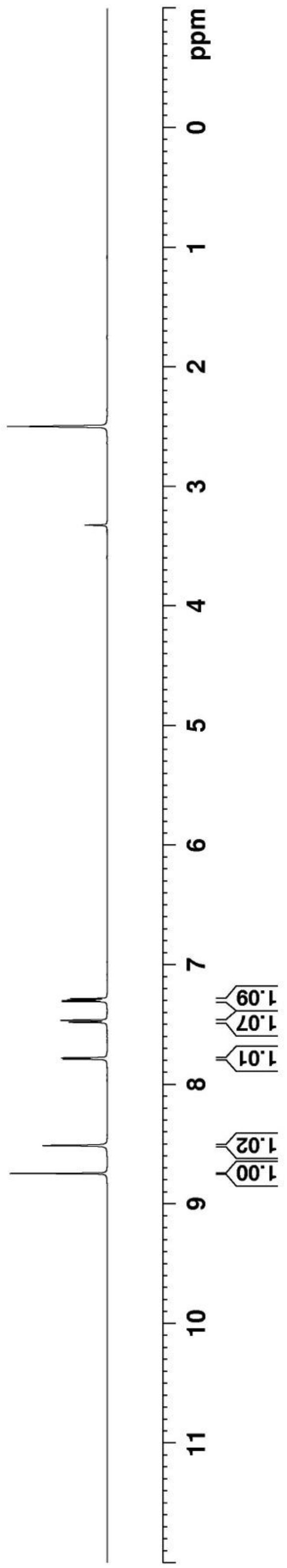

^{13}C NMR (($(CD_3)_2SO$, 25 ºC) of **S5**

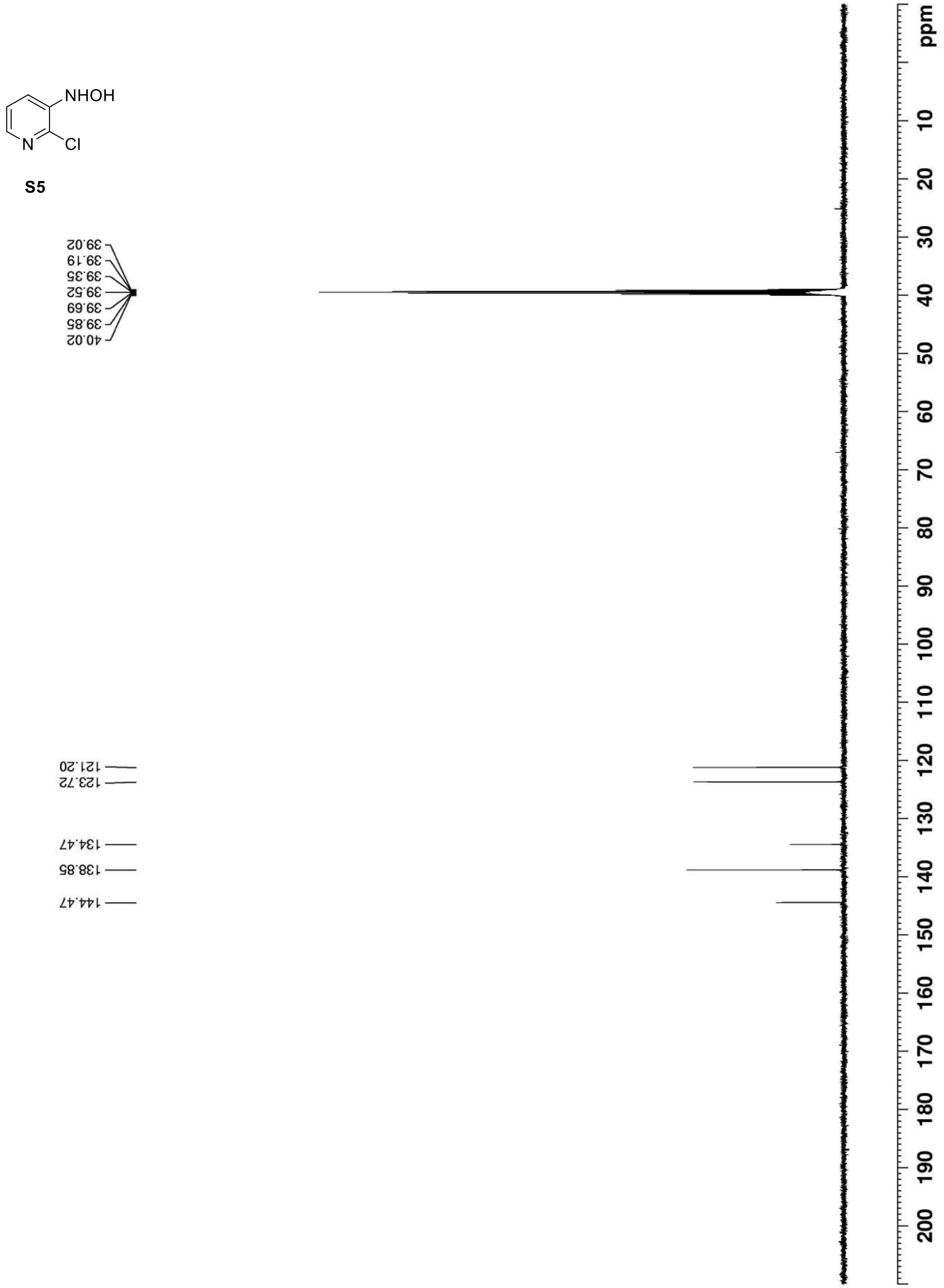

^{1}H NMR ($(CD_3)_2SO$, 25 ºC) of **1g**

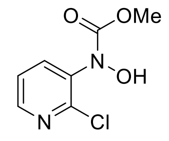

1g

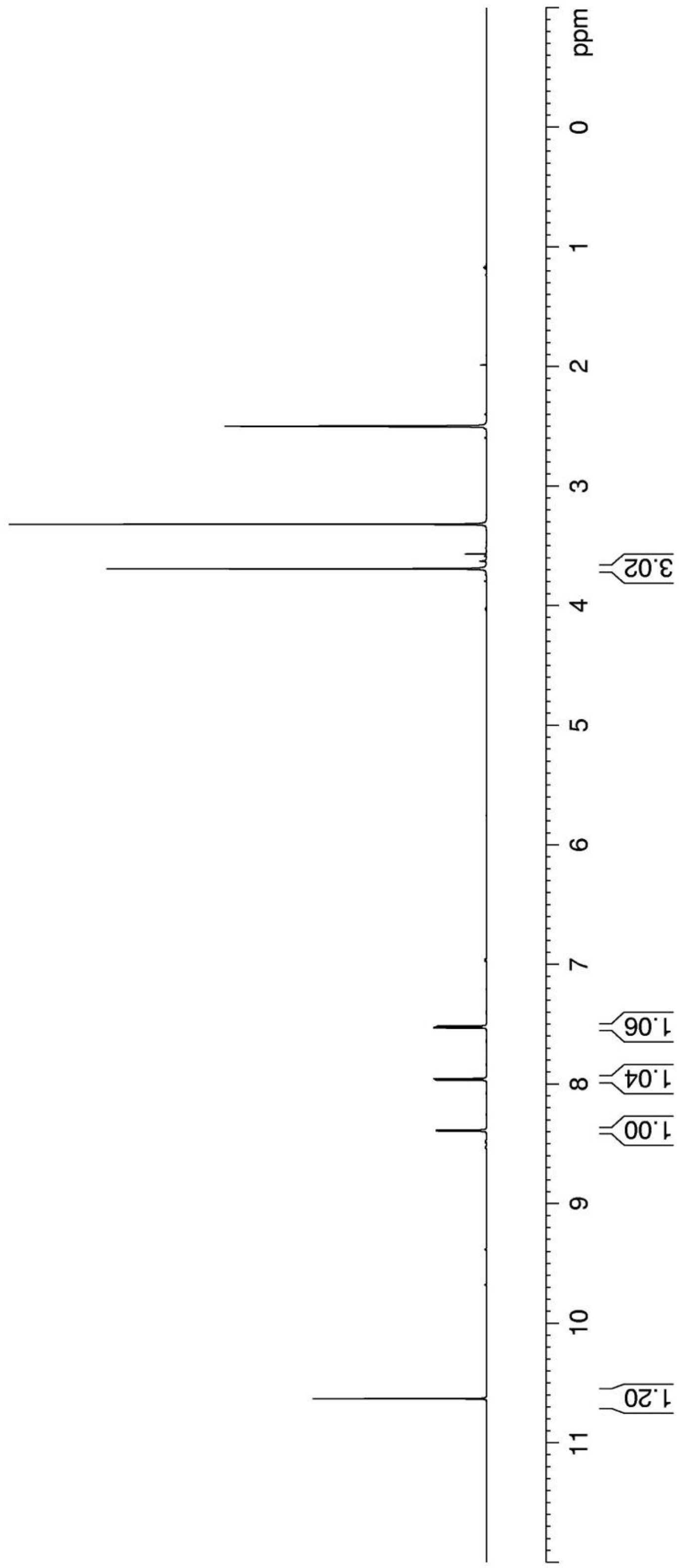

^{13}C NMR ((CD_3)$_2$SO, 25 ºC) of **1g**

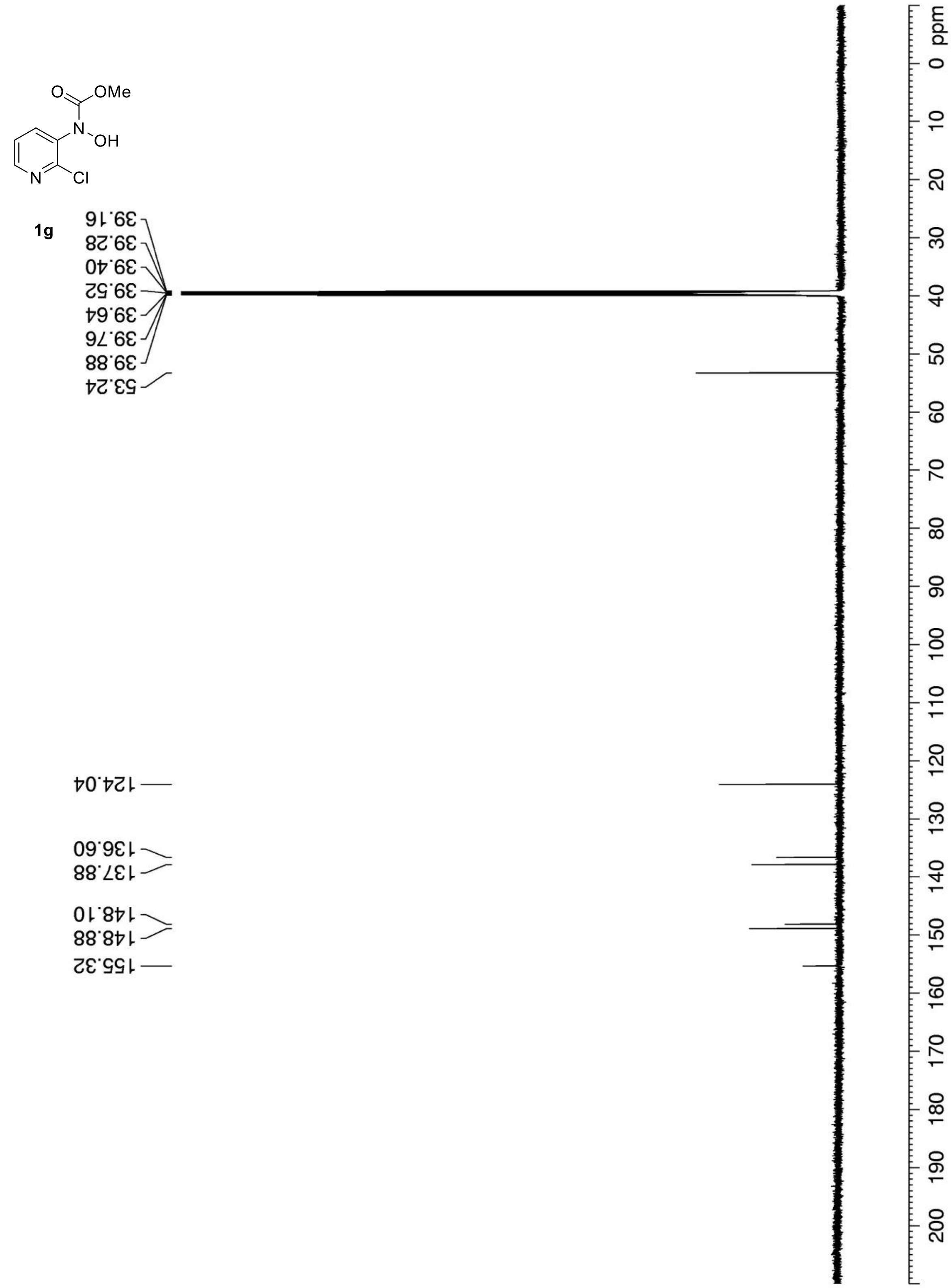

^{1}H NMR ($(CD_3)_2SO$, 25 ºC) of **1h**

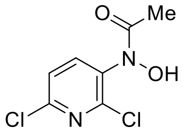

1h

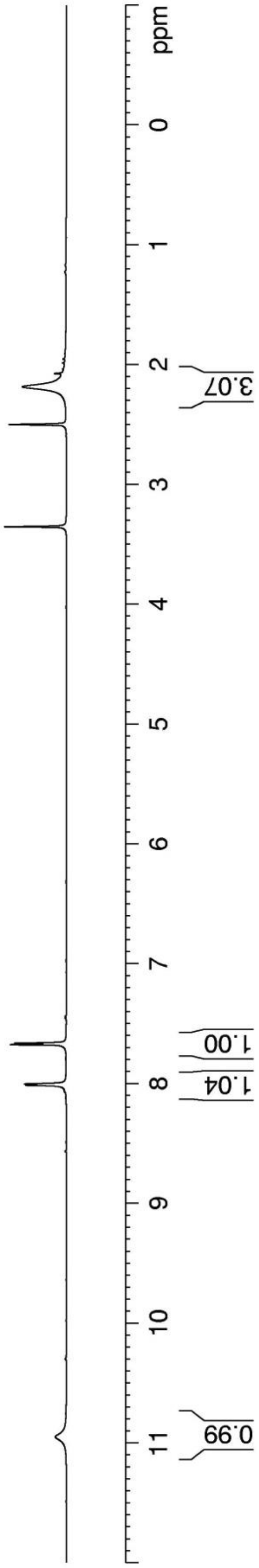

^{13}C NMR (($(CD_3)_2SO$, 25 ºC) of **1h**

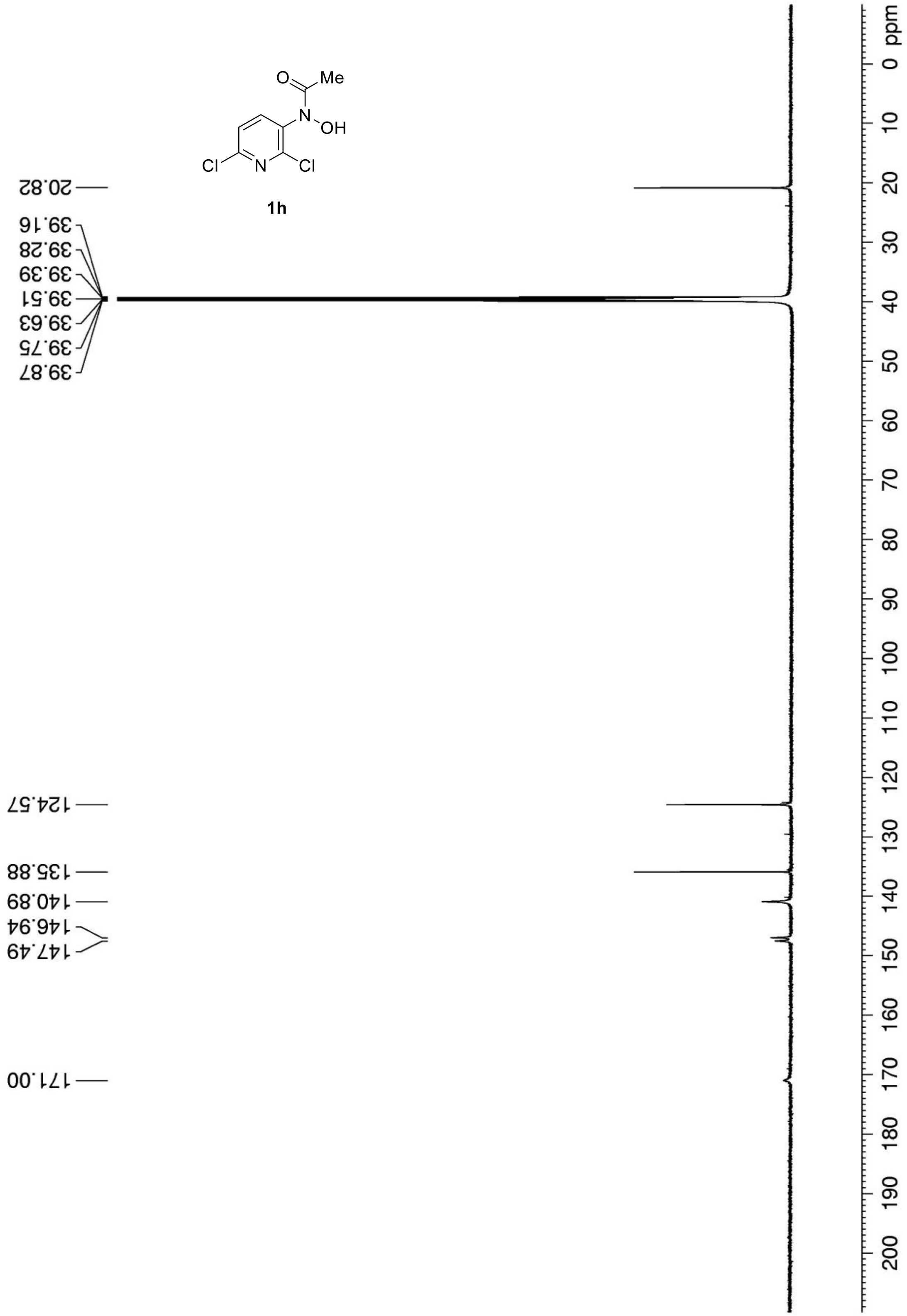

^{1}H NMR ($(CD_3)_2SO$, 25 ºC) of **1i**

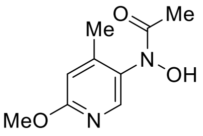

1i

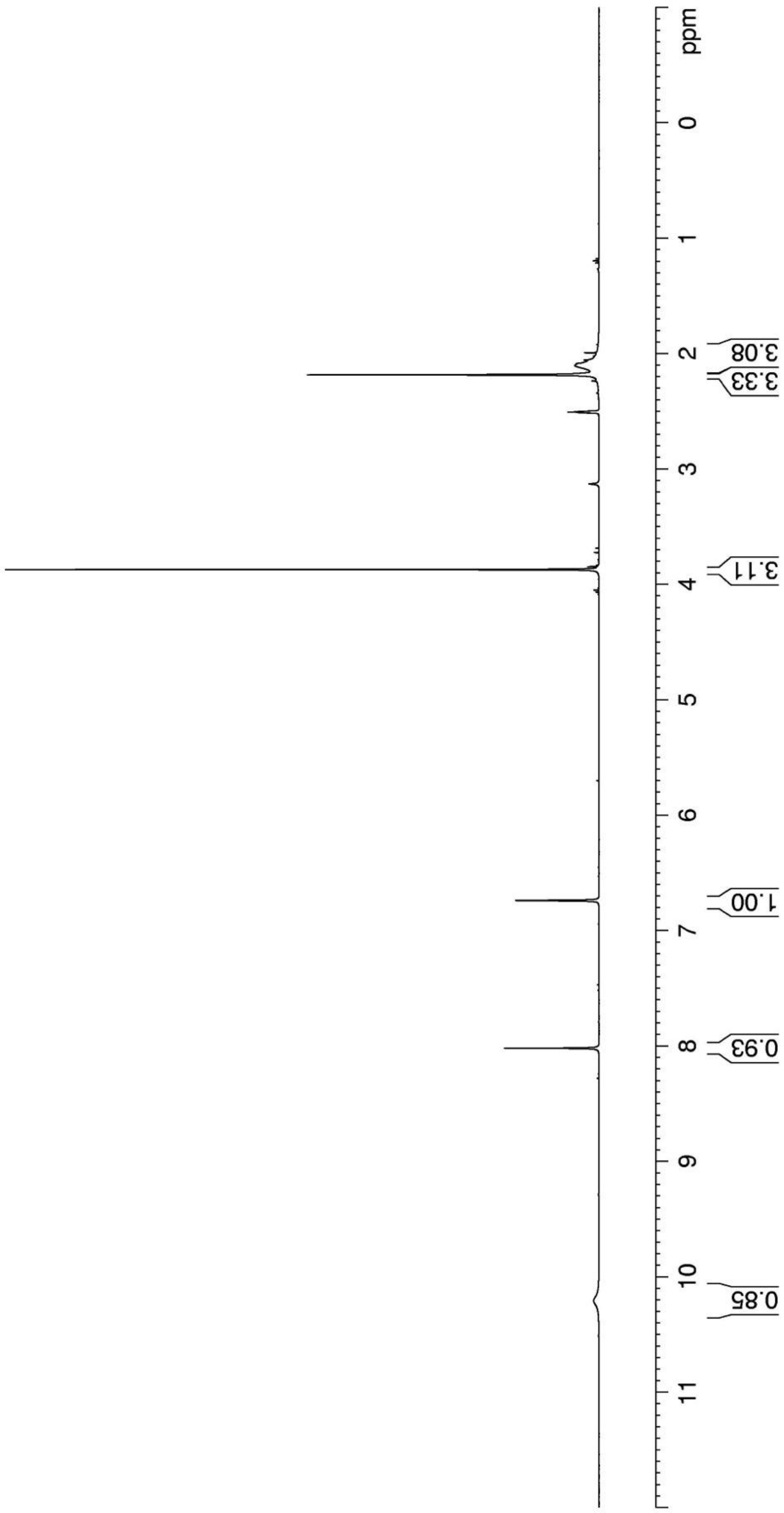

^{13}C NMR ($((CD_3)_2SO$, 25 ºC) of **1i**

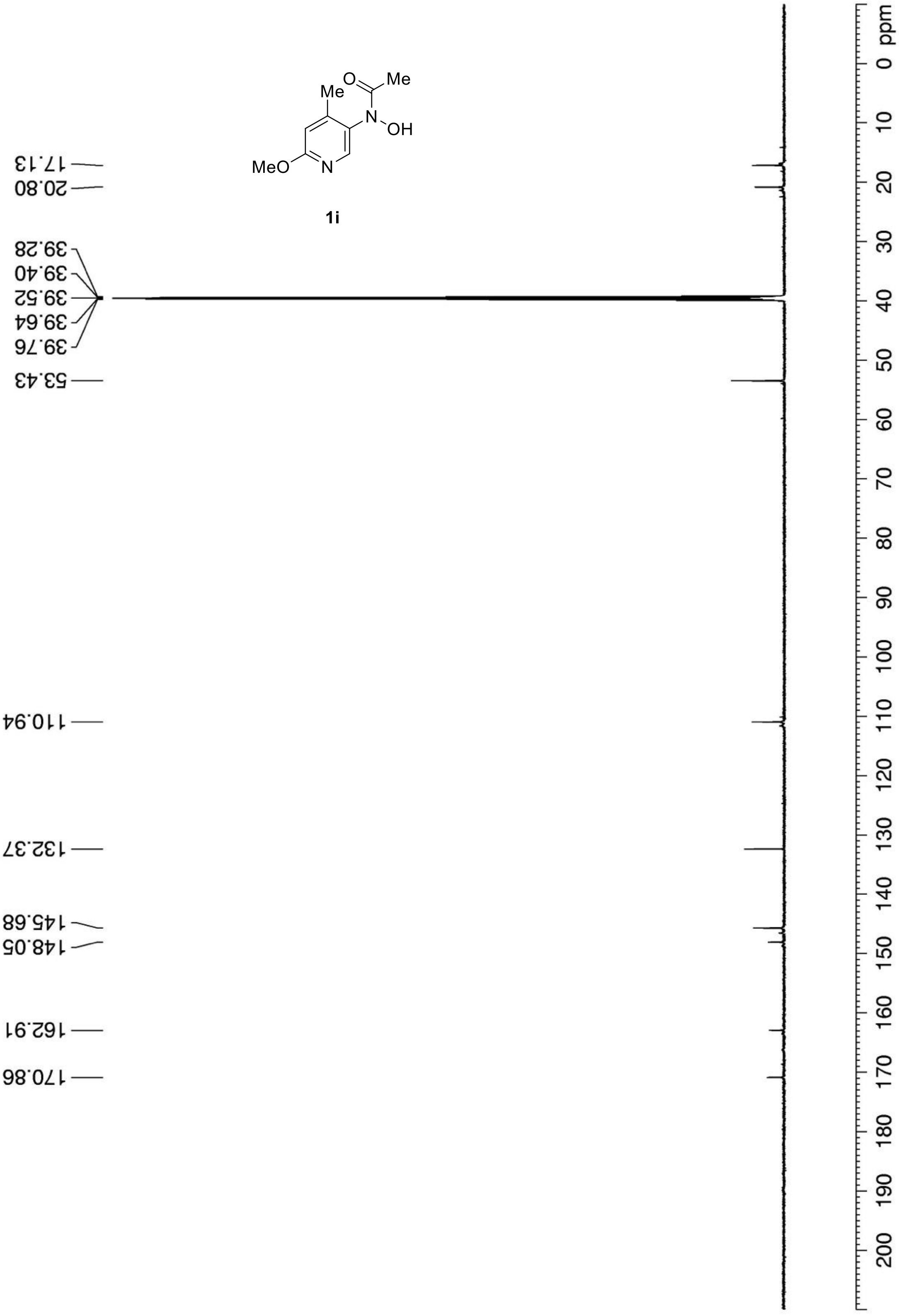

^{1}H NMR (($(CD_3)_2SO$, 25 ºC) of **S6**

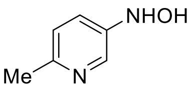

S6

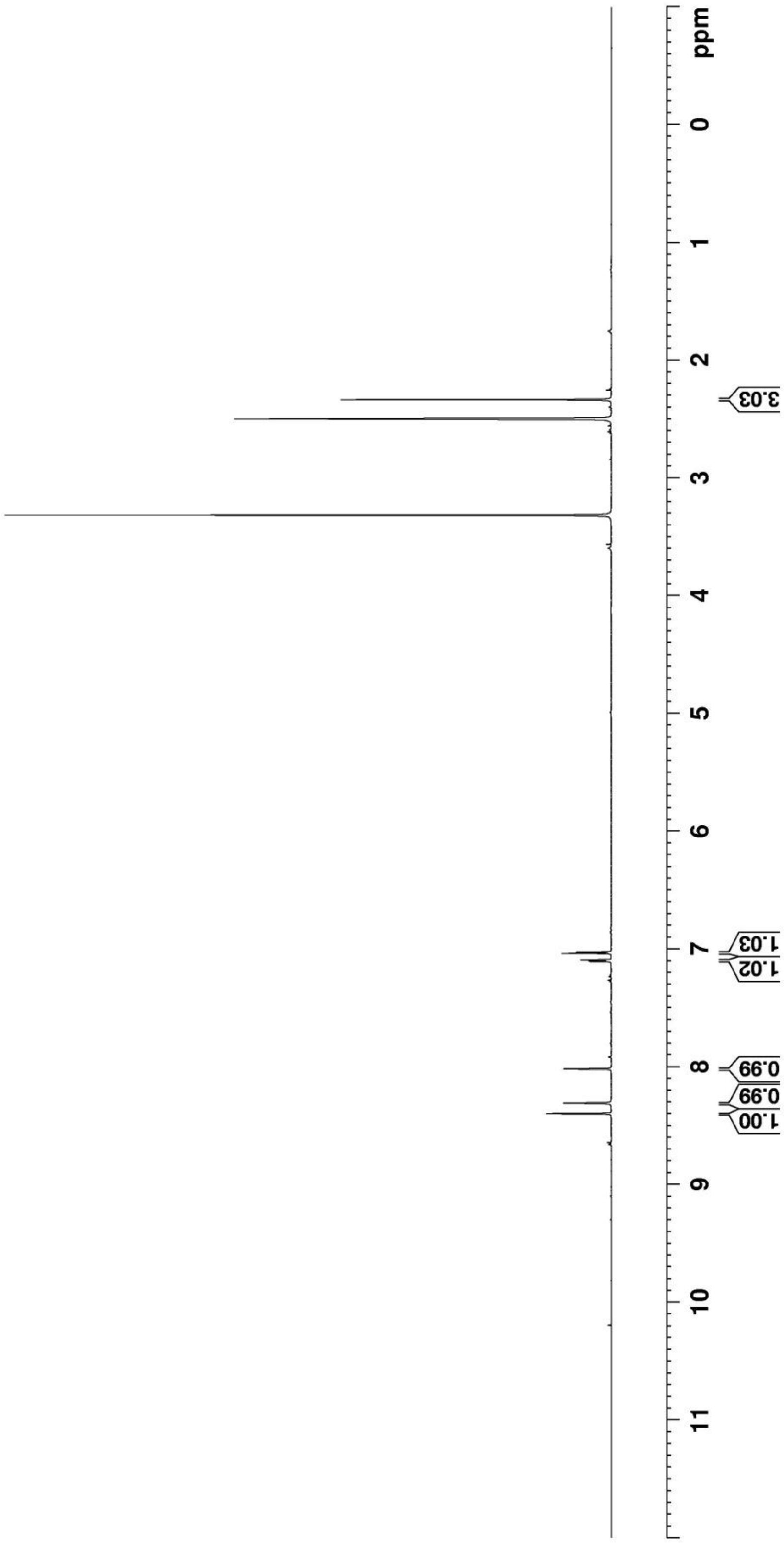

^{13}C NMR ($(CD_3)_2SO$, 25 ºC) of **S6**

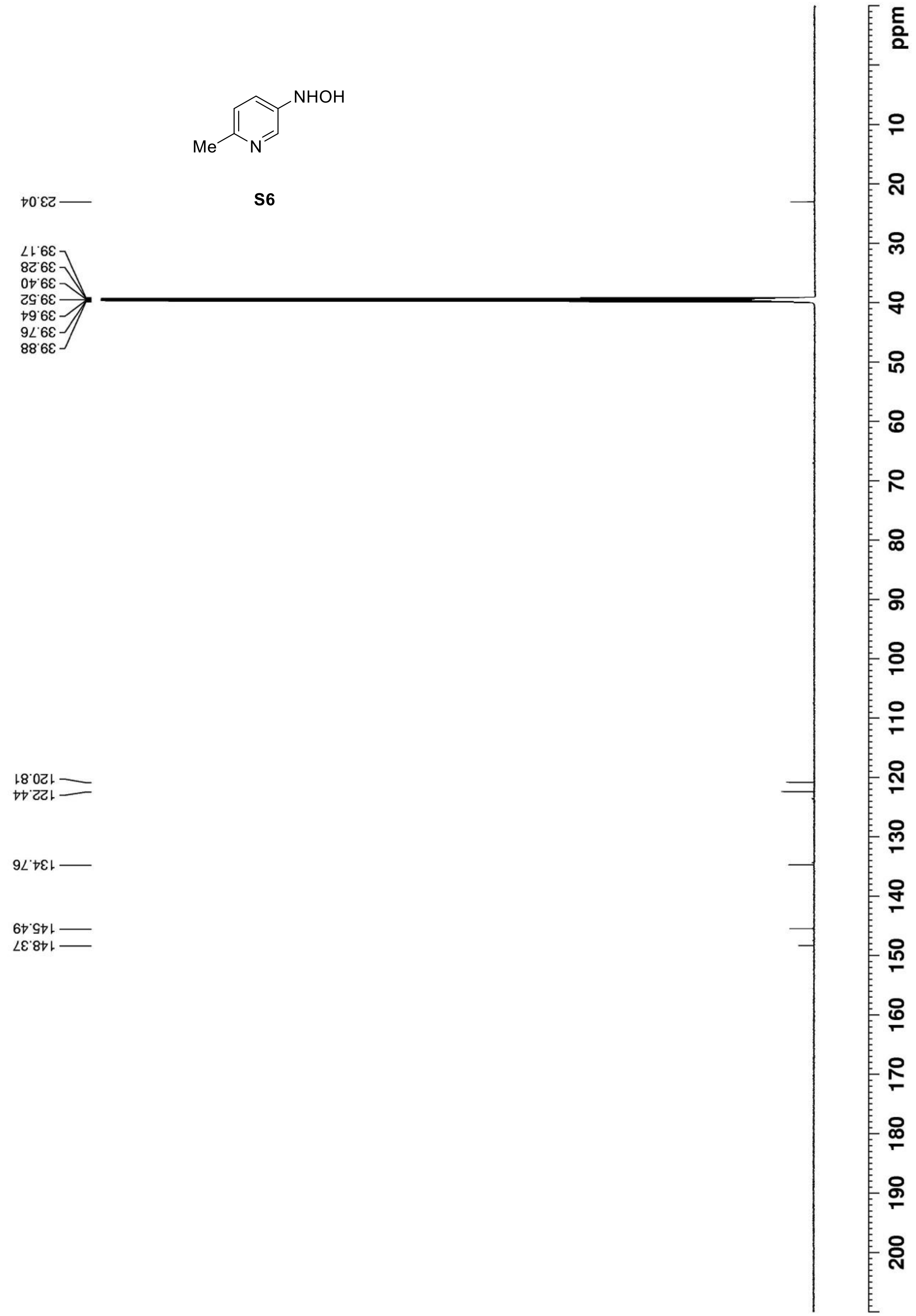

^{1}H NMR ($(CD_3)_2SO$, 25 ºC) of **1j**

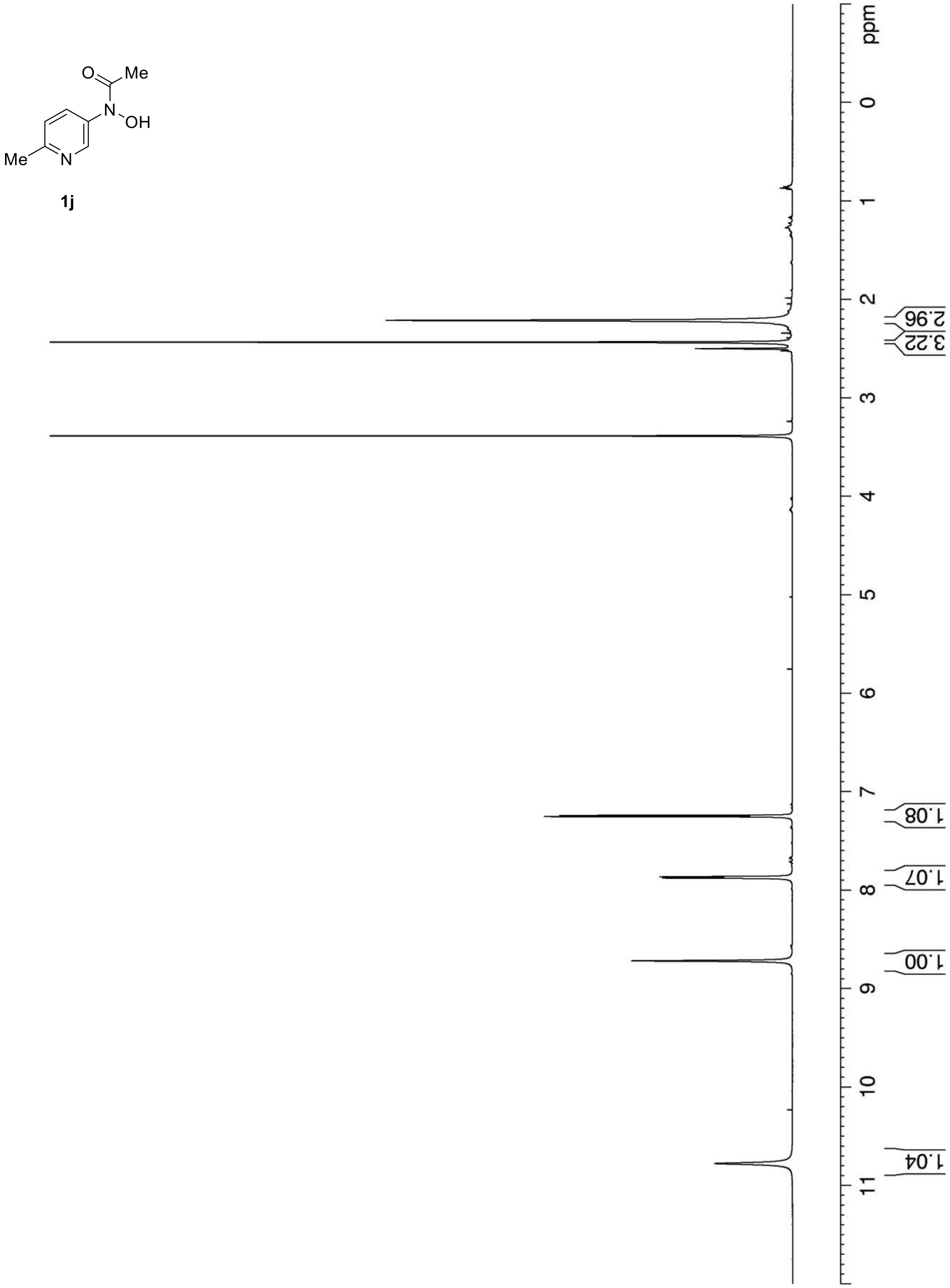

^{13}C NMR ($(CD_3)_2SO$, 25 ºC) of **1j**

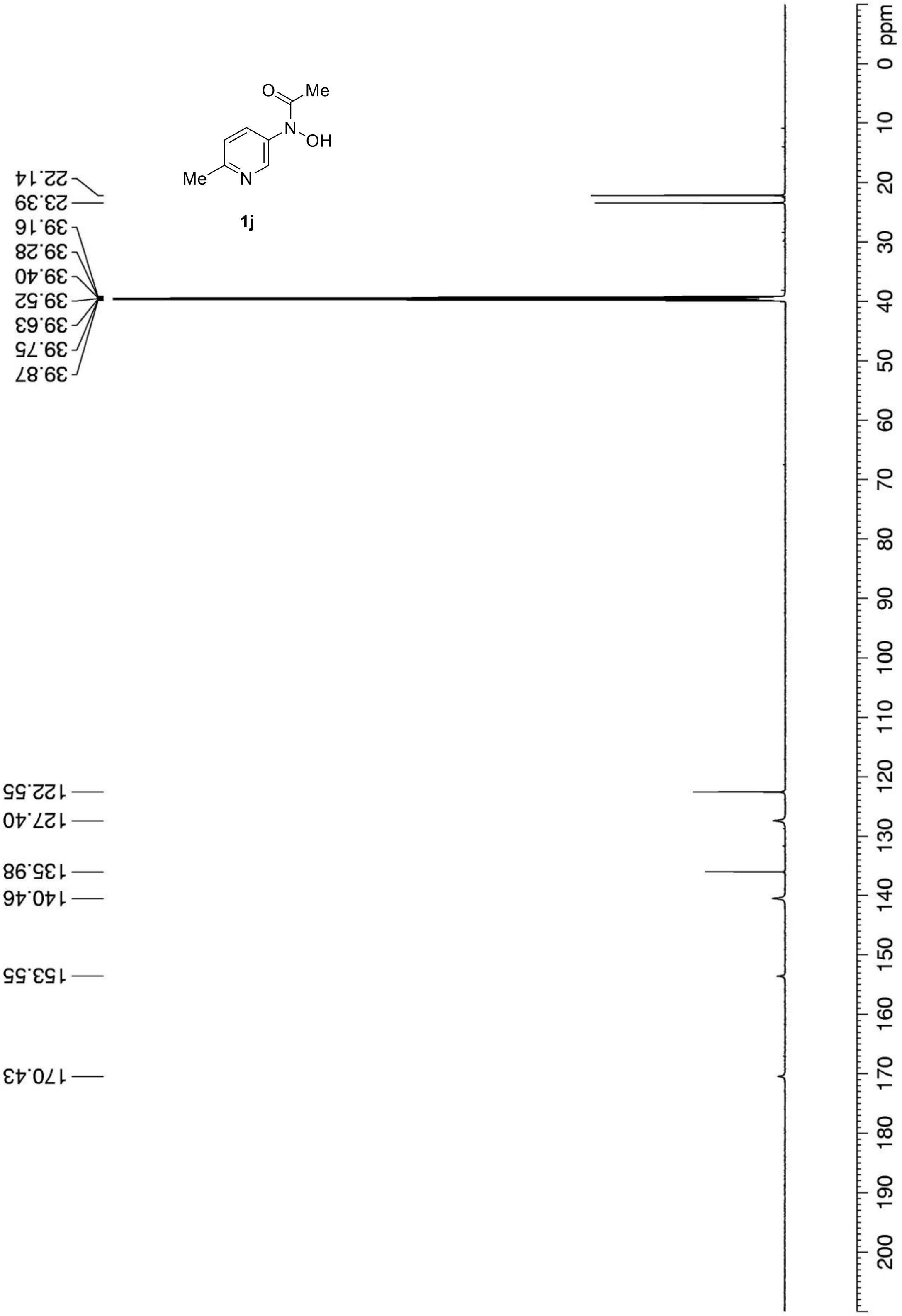

^{1}H NMR ($CDCl_3$, 25 ºC) of **S7**

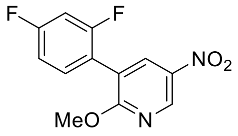

S7

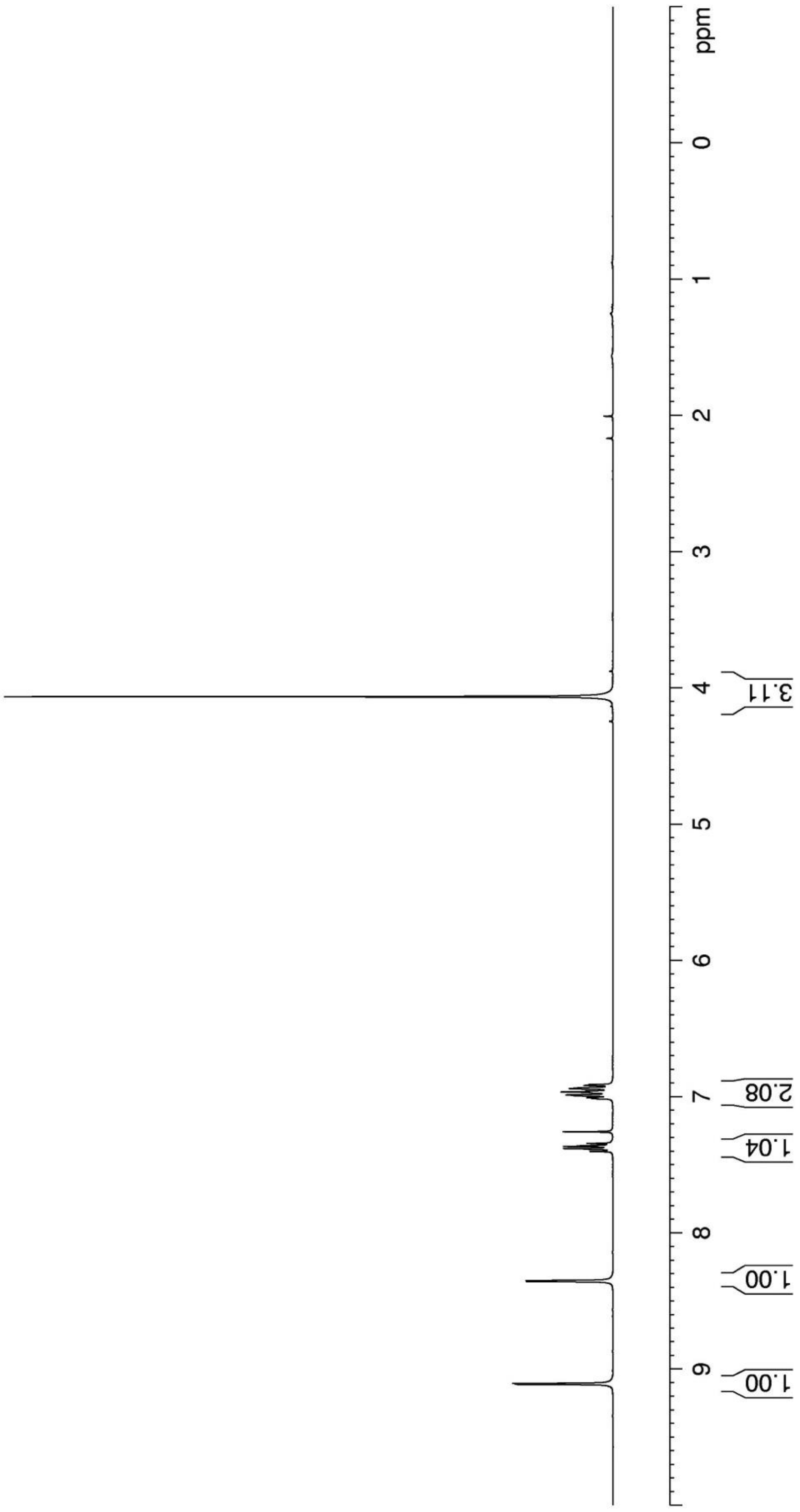

^{13}C NMR ($CDCl_3$, 25 ºC) of **S7**

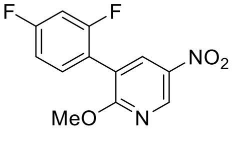

S7

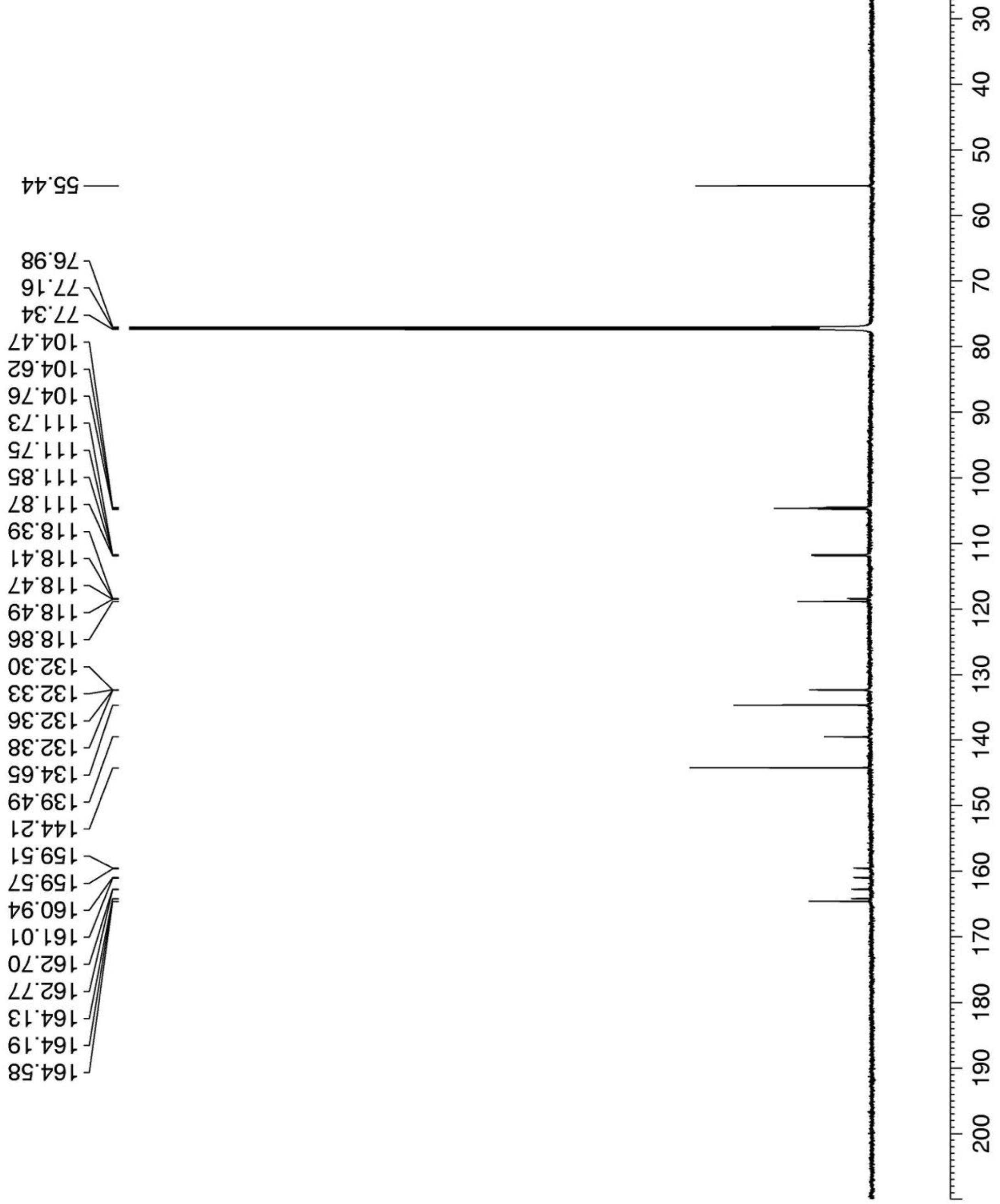

^{19}F NMR ($CDCl_3$, 25 ºC) of **S7**

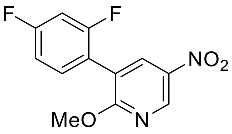

S7

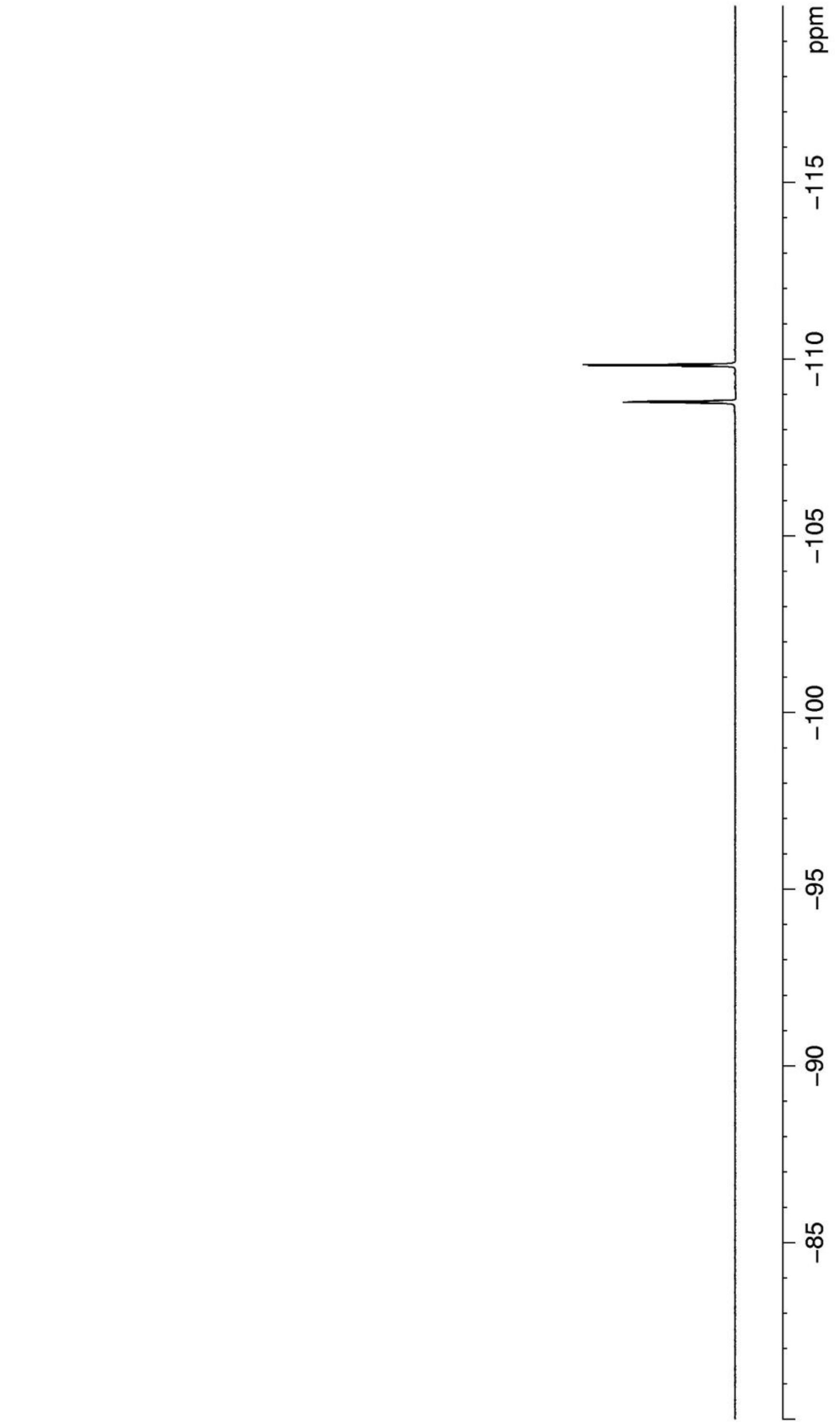

^{1}H NMR (($(CD_3)_2SO$, 25 ºC) of **S8**

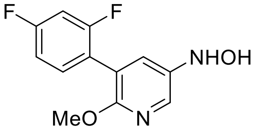

S8

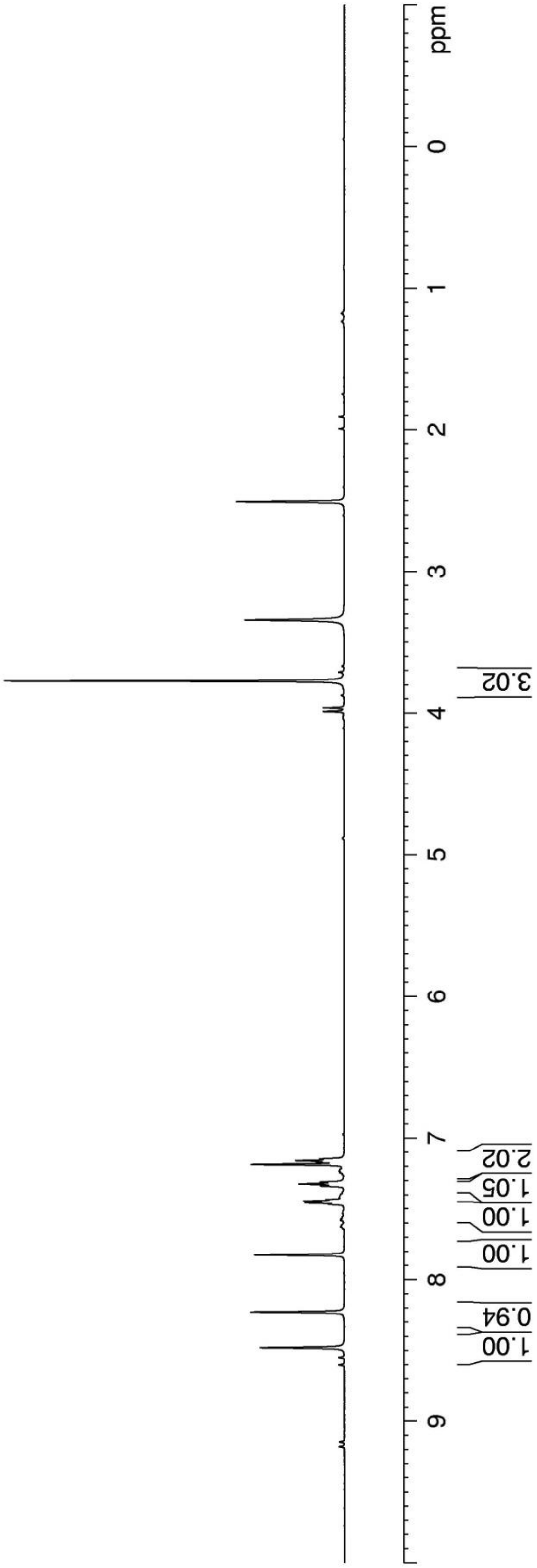

^{13}C NMR (($(CD_3)_2SO$, 25 ºC) of **S8**

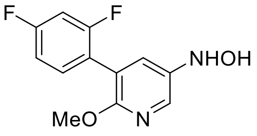

S8

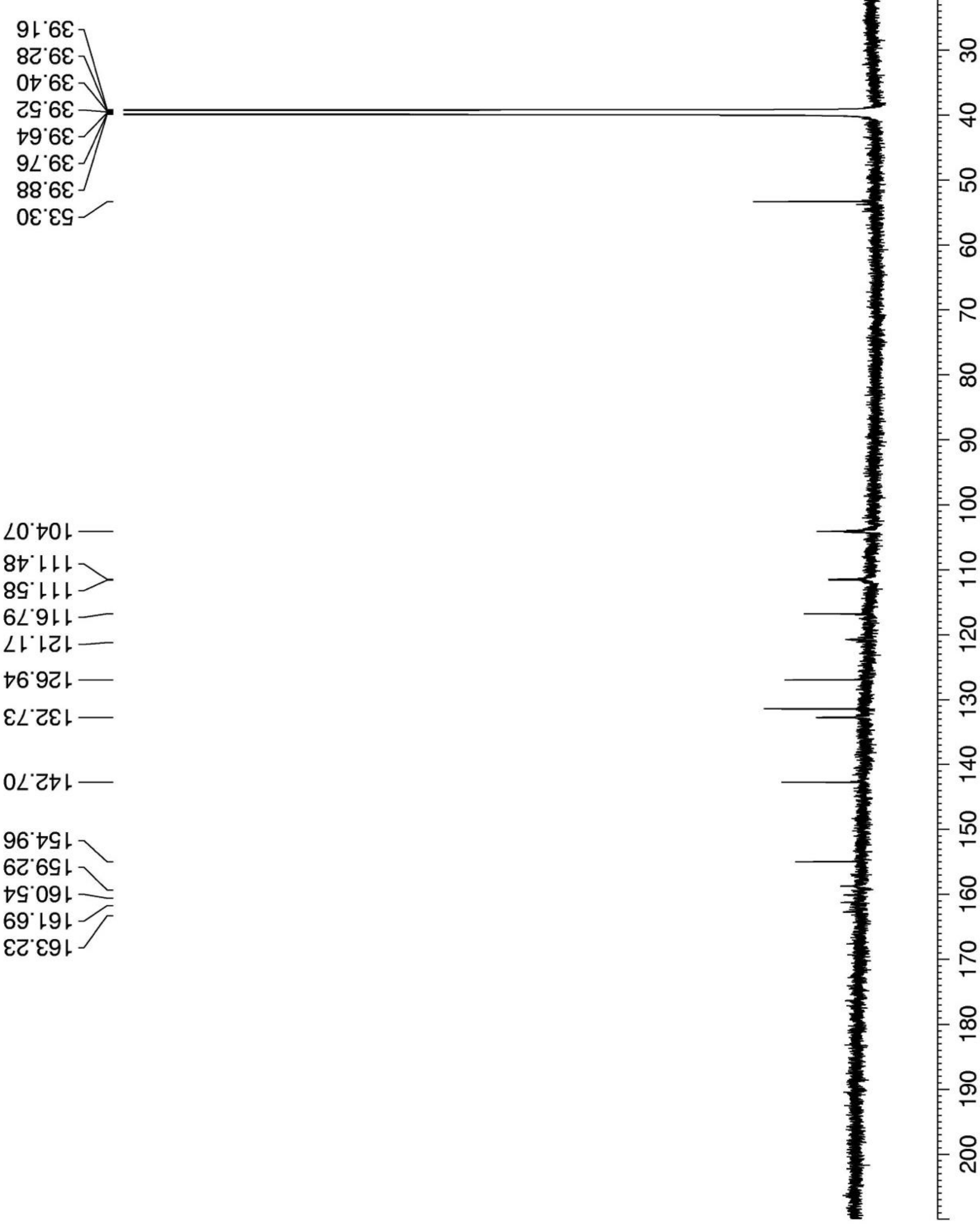

^{19}F NMR ((CD$_3$)$_2$SO, 25 ºC) of **S8**

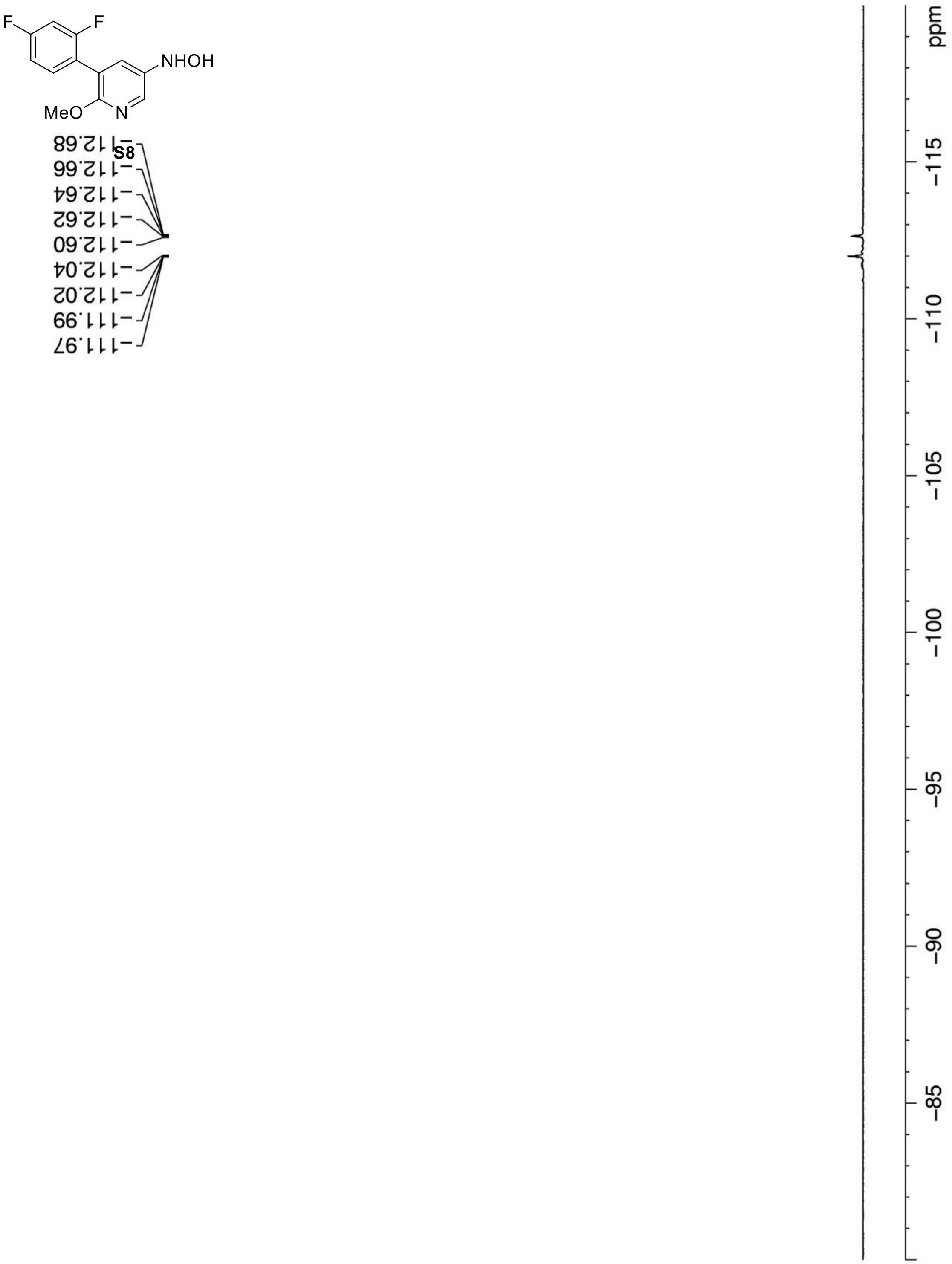

^{1}H NMR ($(CD_3)_2SO$, 25 ºC) of **1k**

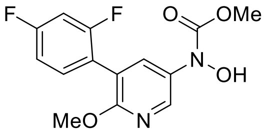

1k

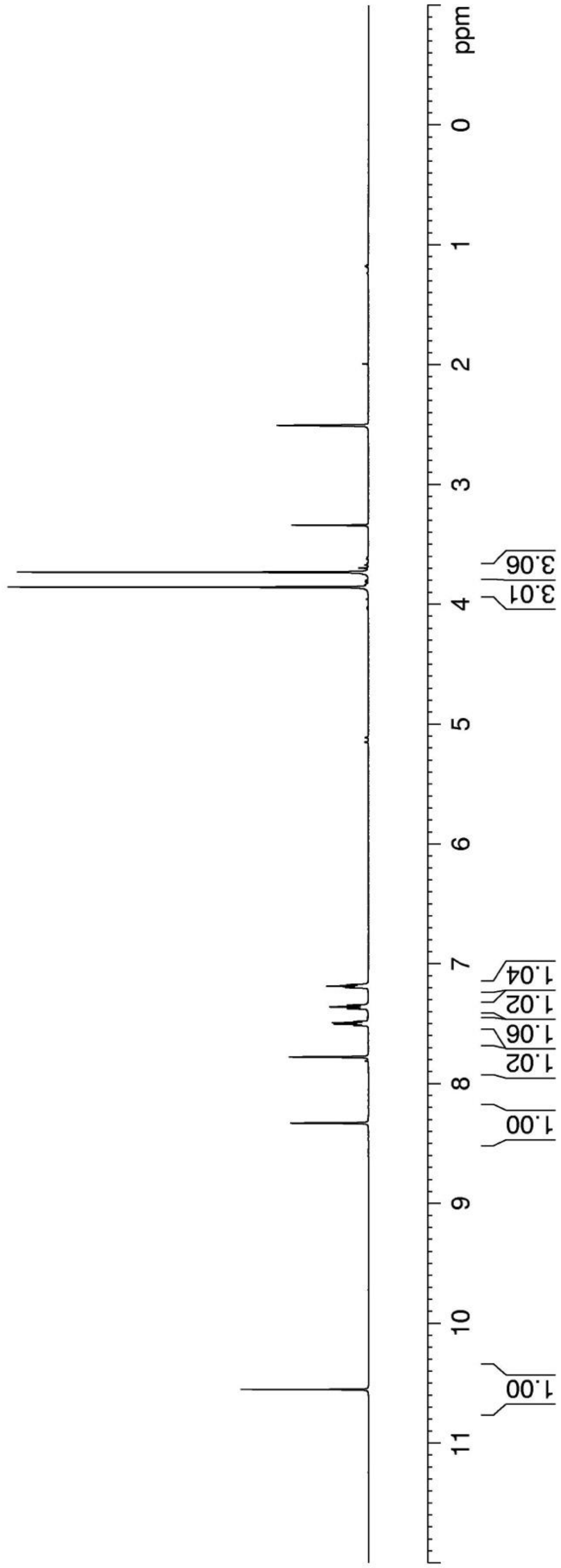

^{13}C NMR ($(CD_3)_2SO$, 25 ºC) of **1k**

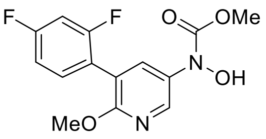

1k

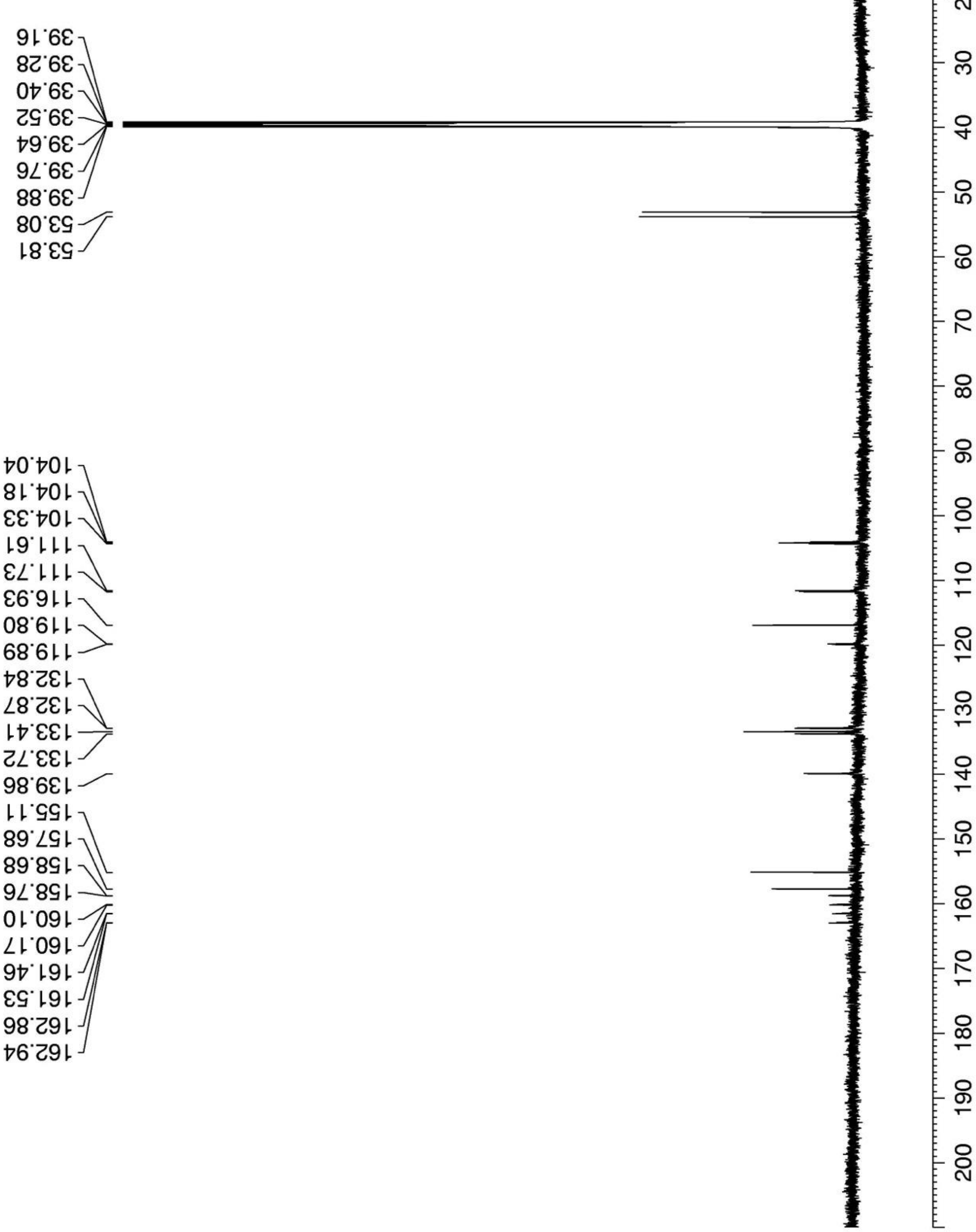

^{19}F NMR ($(CD_3)_2SO$, 25 ºC) of **1k**

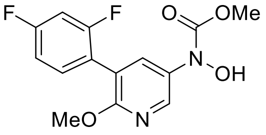

1k

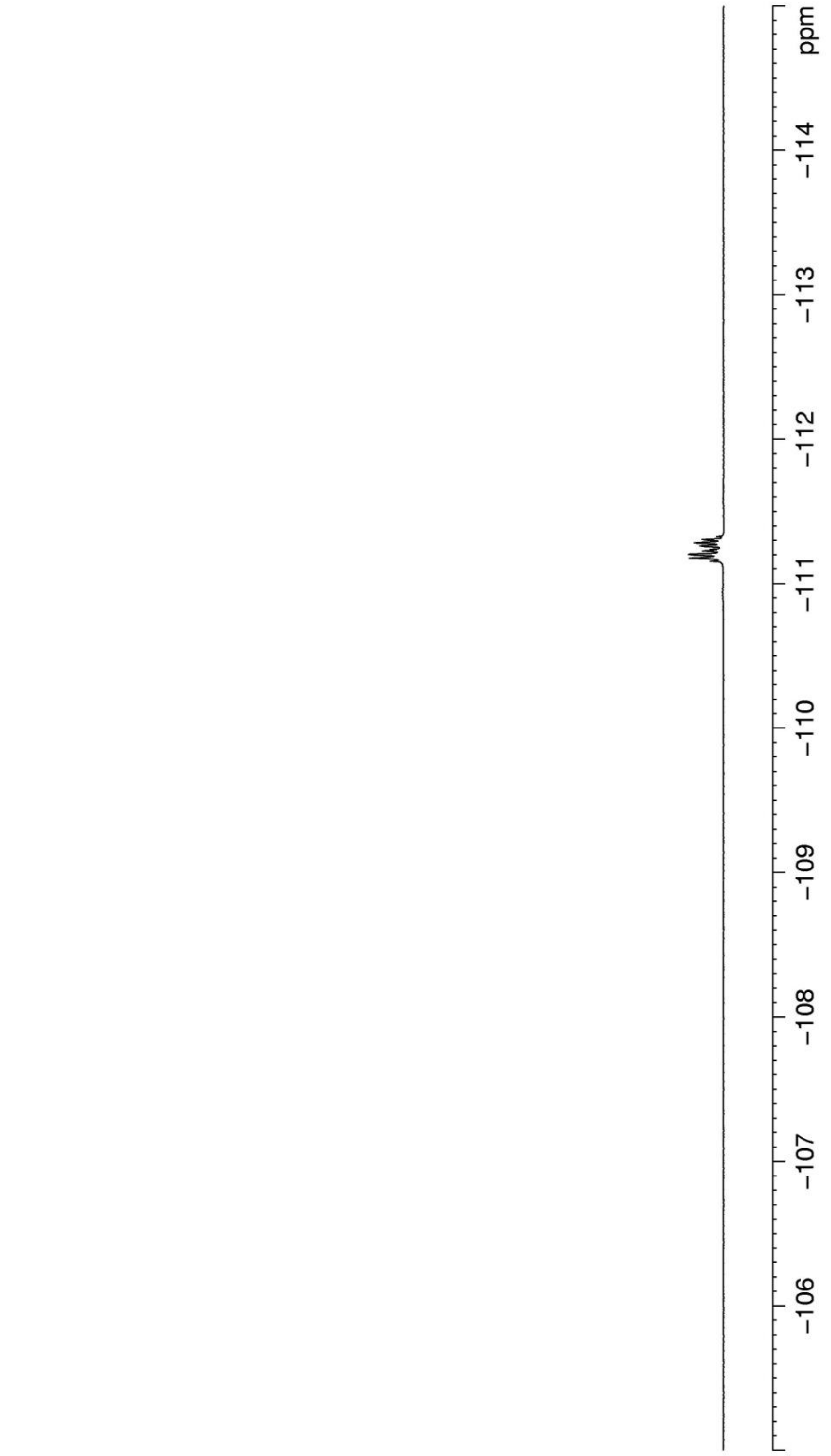

^{1}H NMR ($(CD_3)_2SO$, 25 ºC) of **S9**

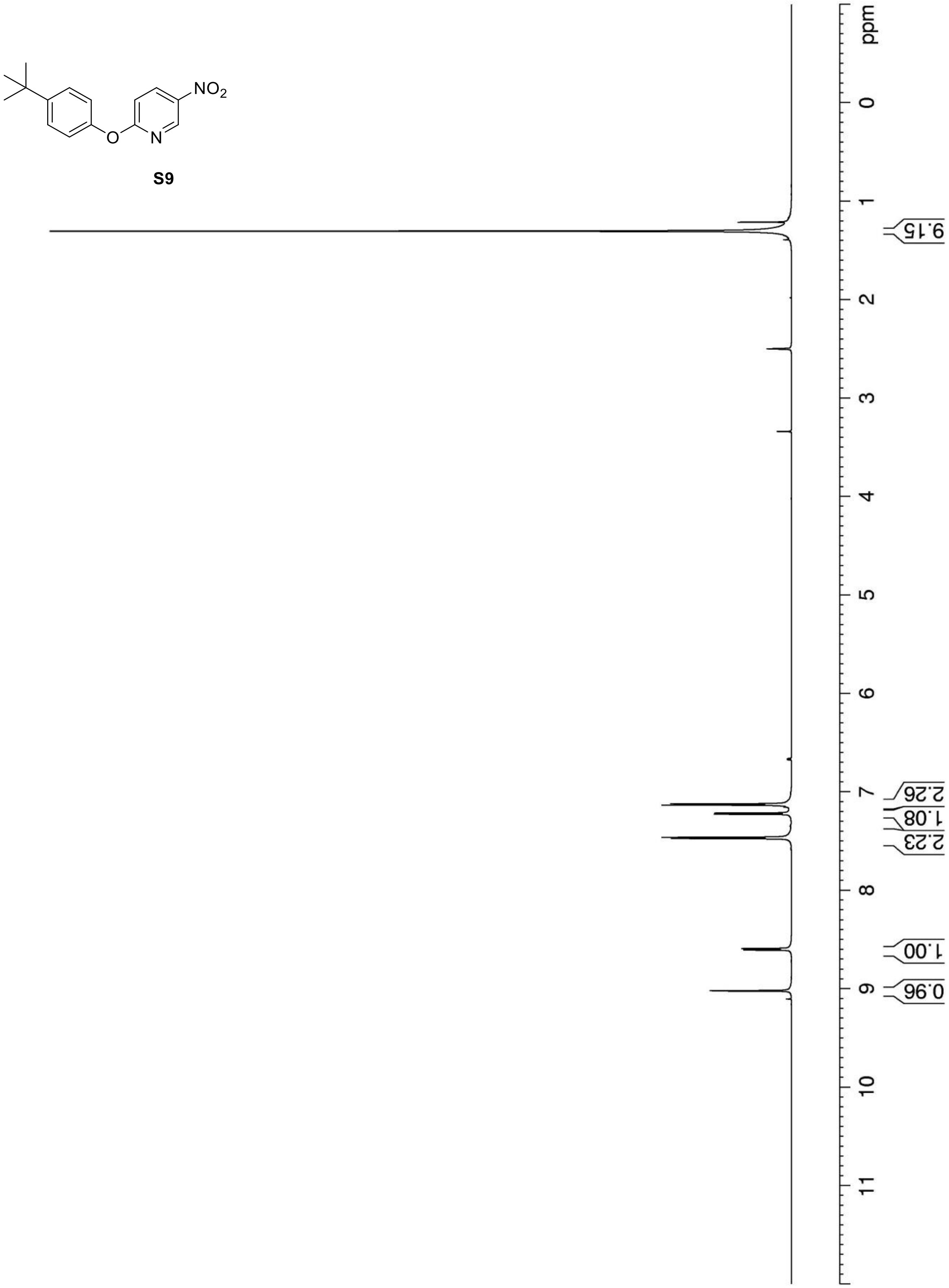

S9

^{13}C NMR (($(CD_3)_2SO$, 25 ºC) of **S9**

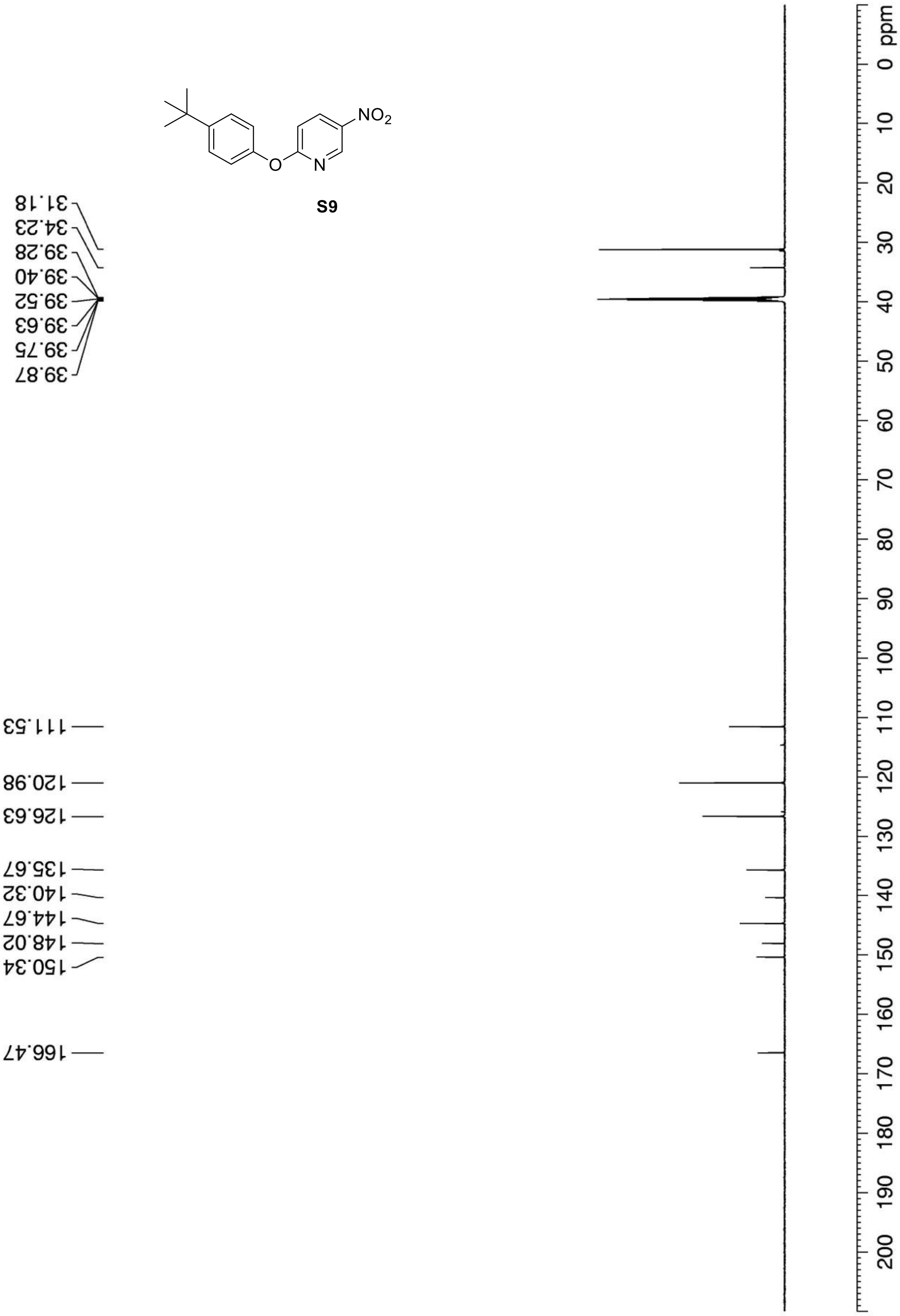

^{1}H NMR ($(CD_3)_2SO$, 25 ºC) of **S10**

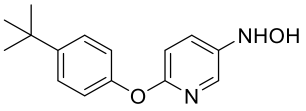

S10

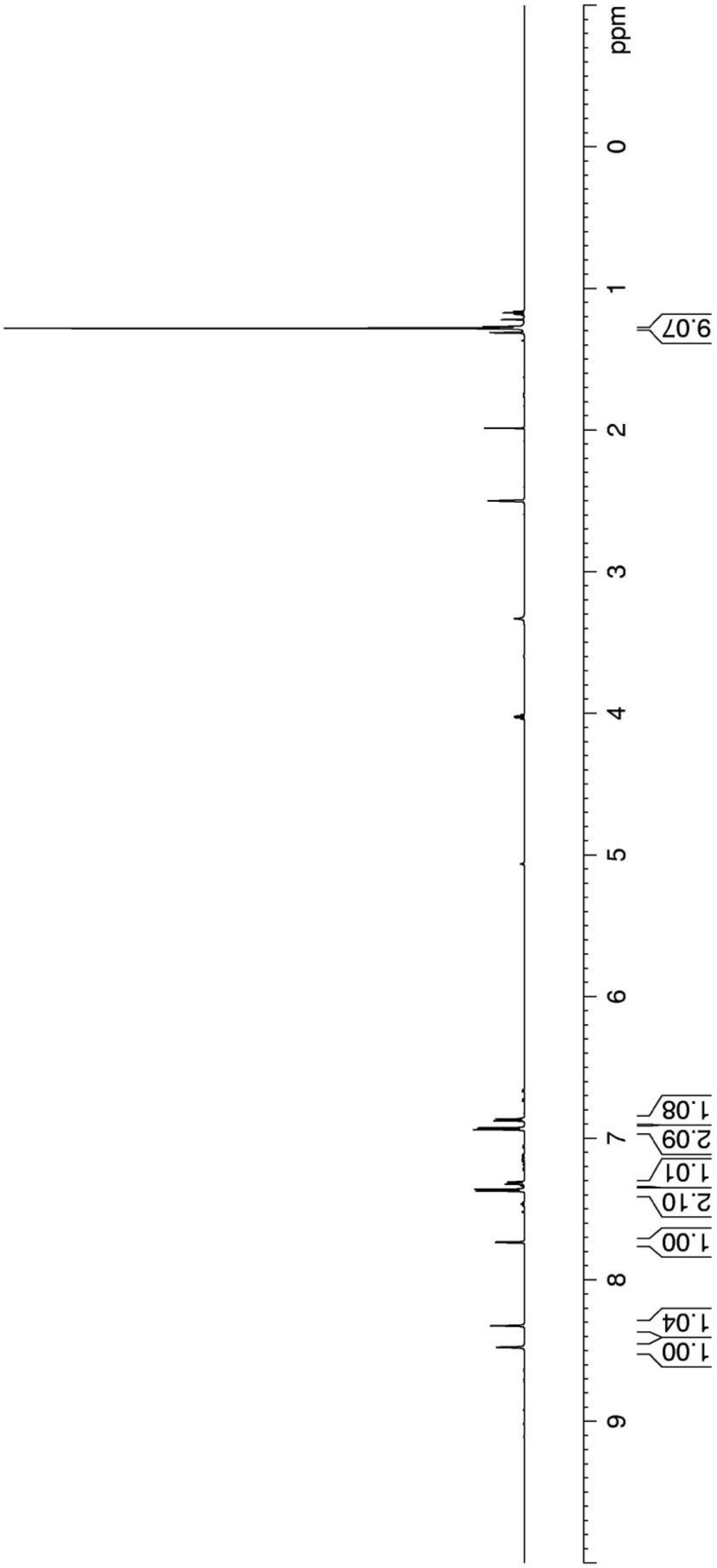

^{13}C NMR $((CD_3)_2SO$, 25 ºC) of **S10**

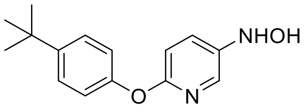

S10

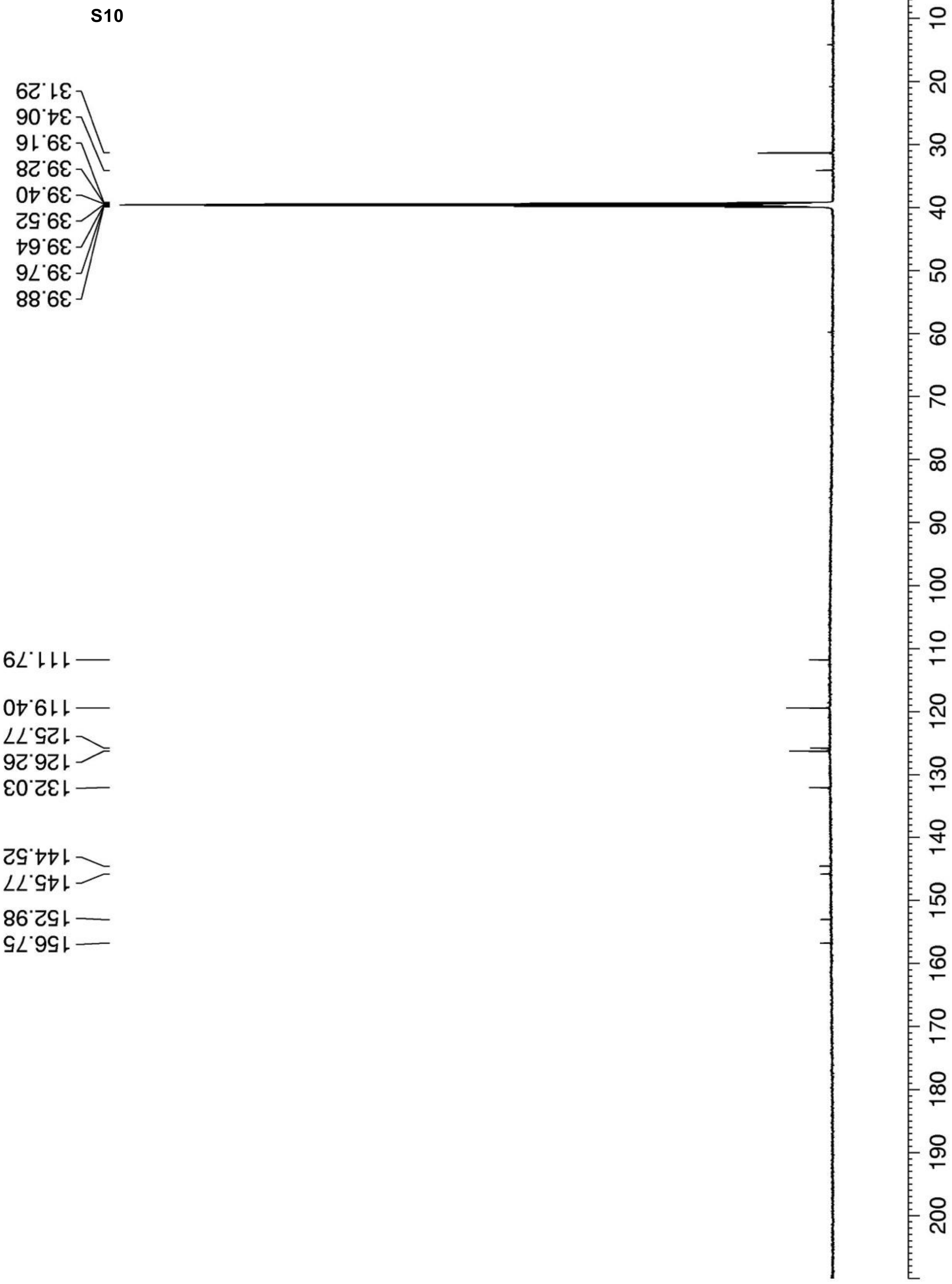

¹H NMR ($(CD_3)_2SO$, 25 ºC) of **1l**

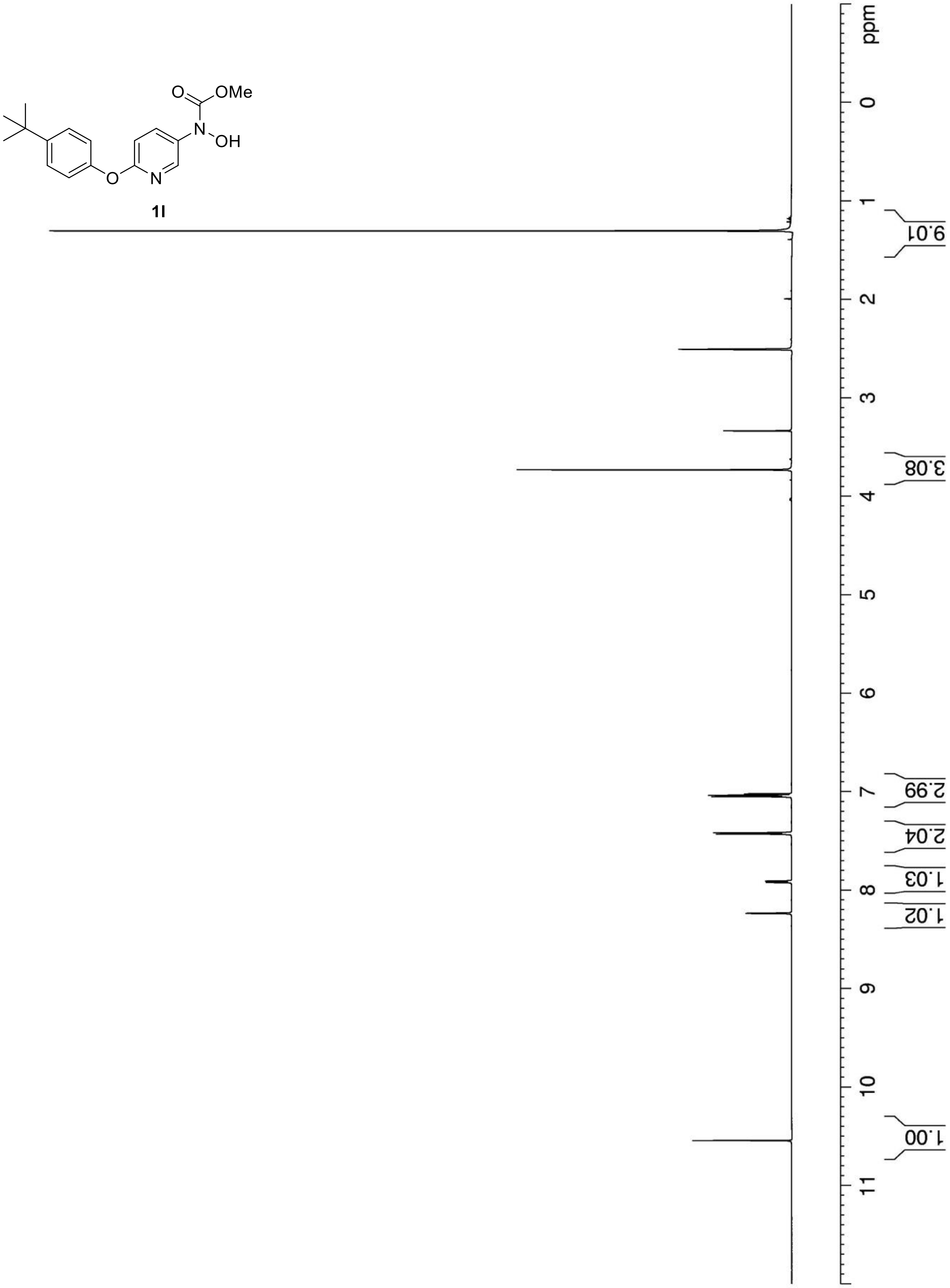

^{13}C NMR ($(CD_3)_2SO$, 25 ºC) of **1l**

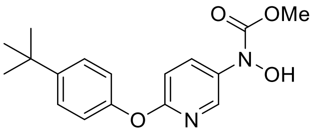

1l

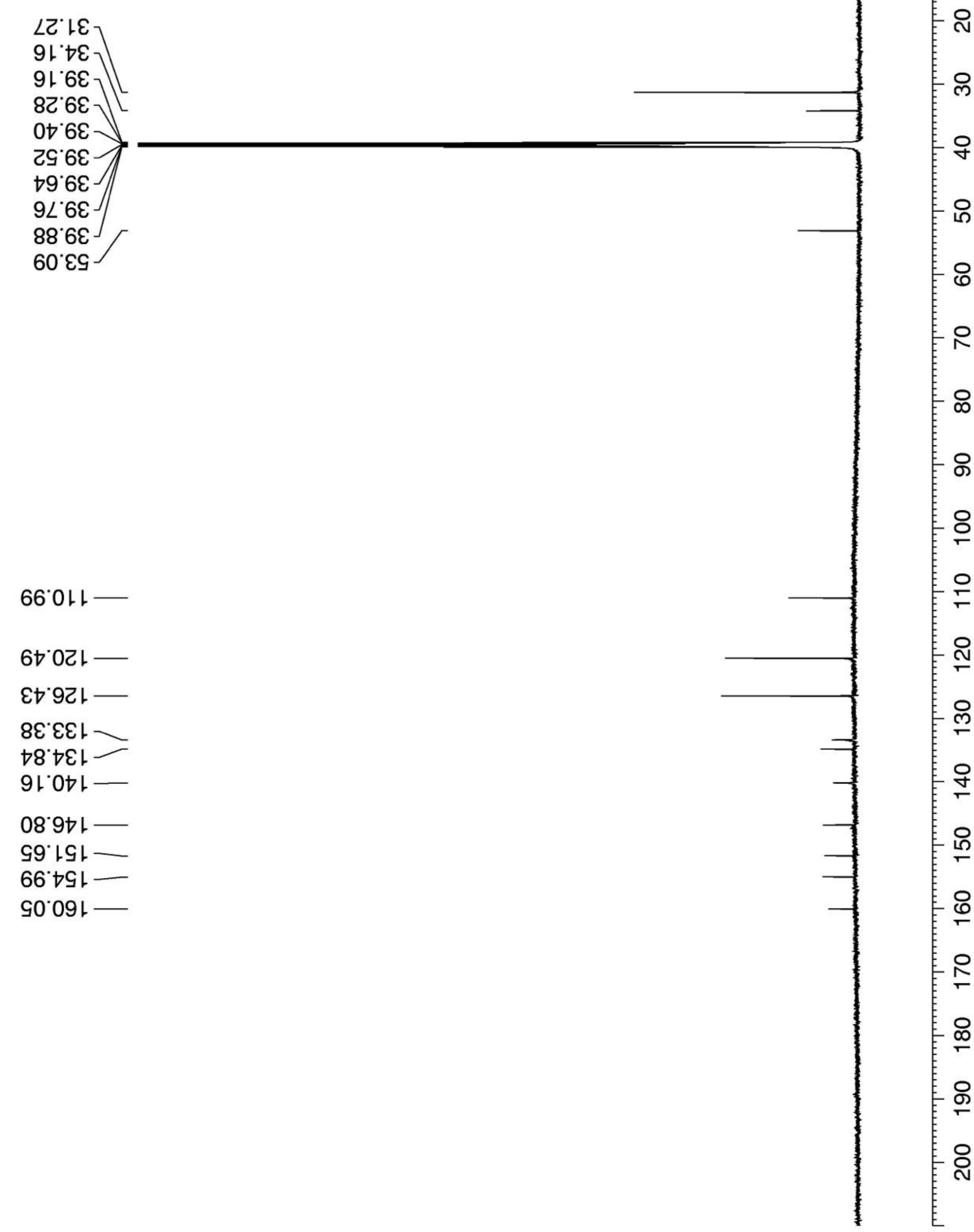

^{1}H NMR (($(CD_3)_2SO$, 25 ºC) of **1m**

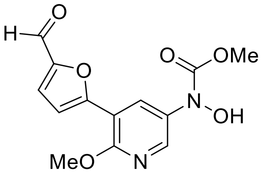

1m

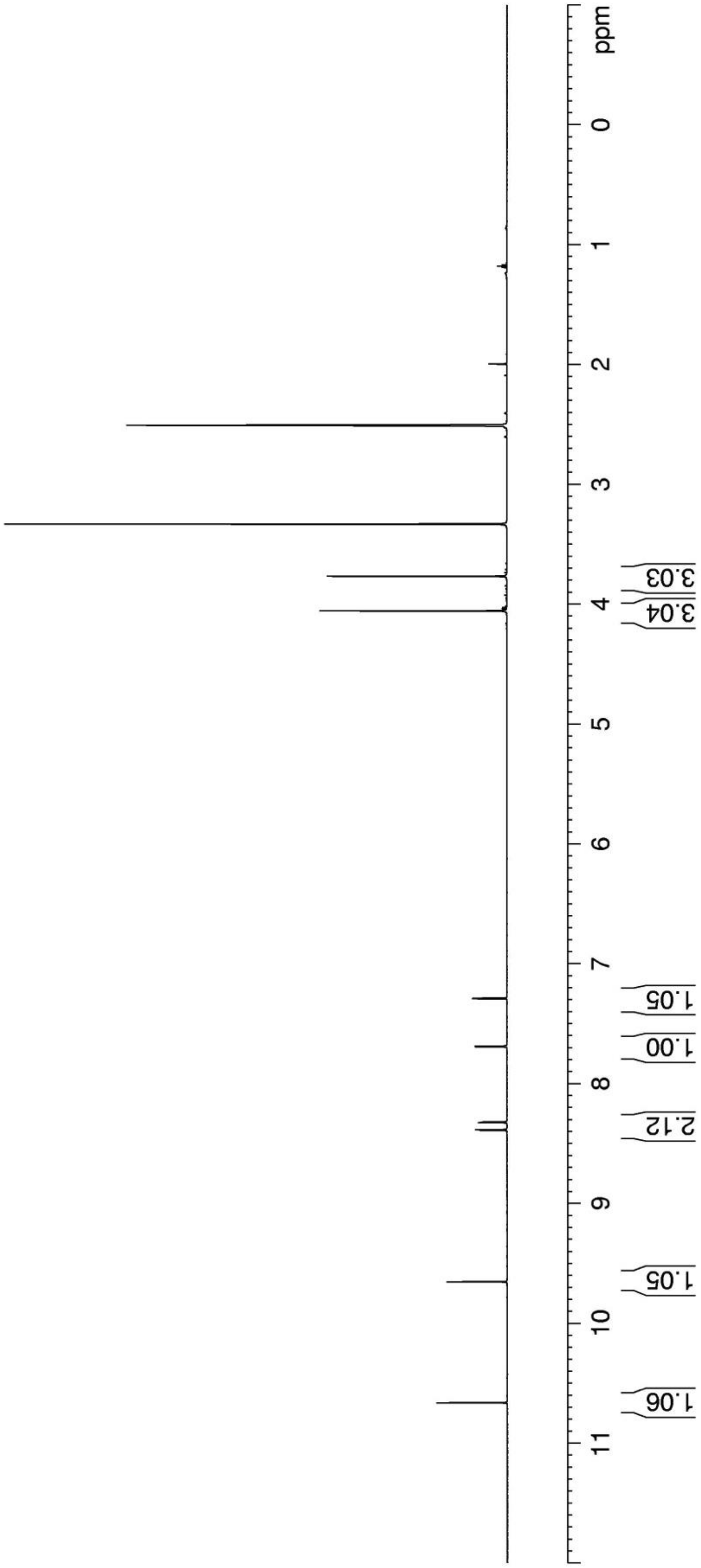

^{13}C NMR (($CD_3)_2SO$, 25 ºC) of **1m**

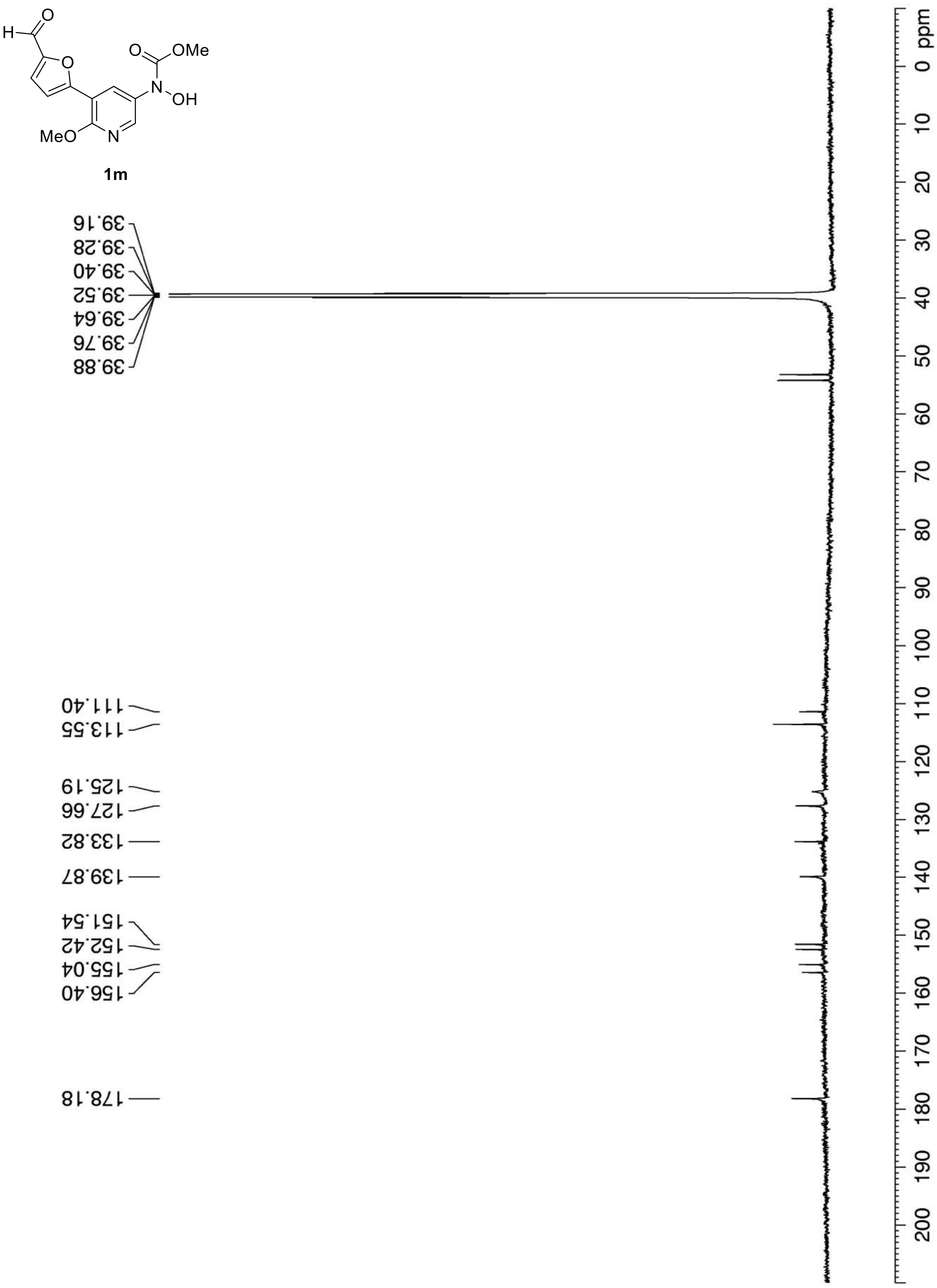

^{1}H NMR ($(CD_3)_2SO$, 25 ºC) of **S11**

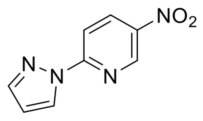

S11

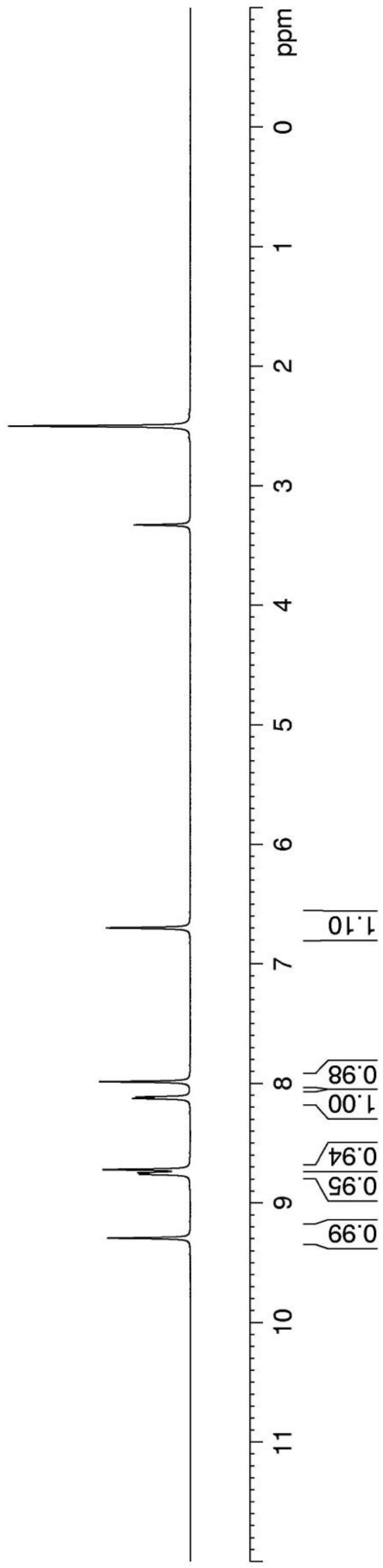

^{13}C NMR (($(CD_3)_2SO$, 25 ºC) of **S11**

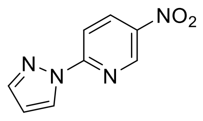

S11

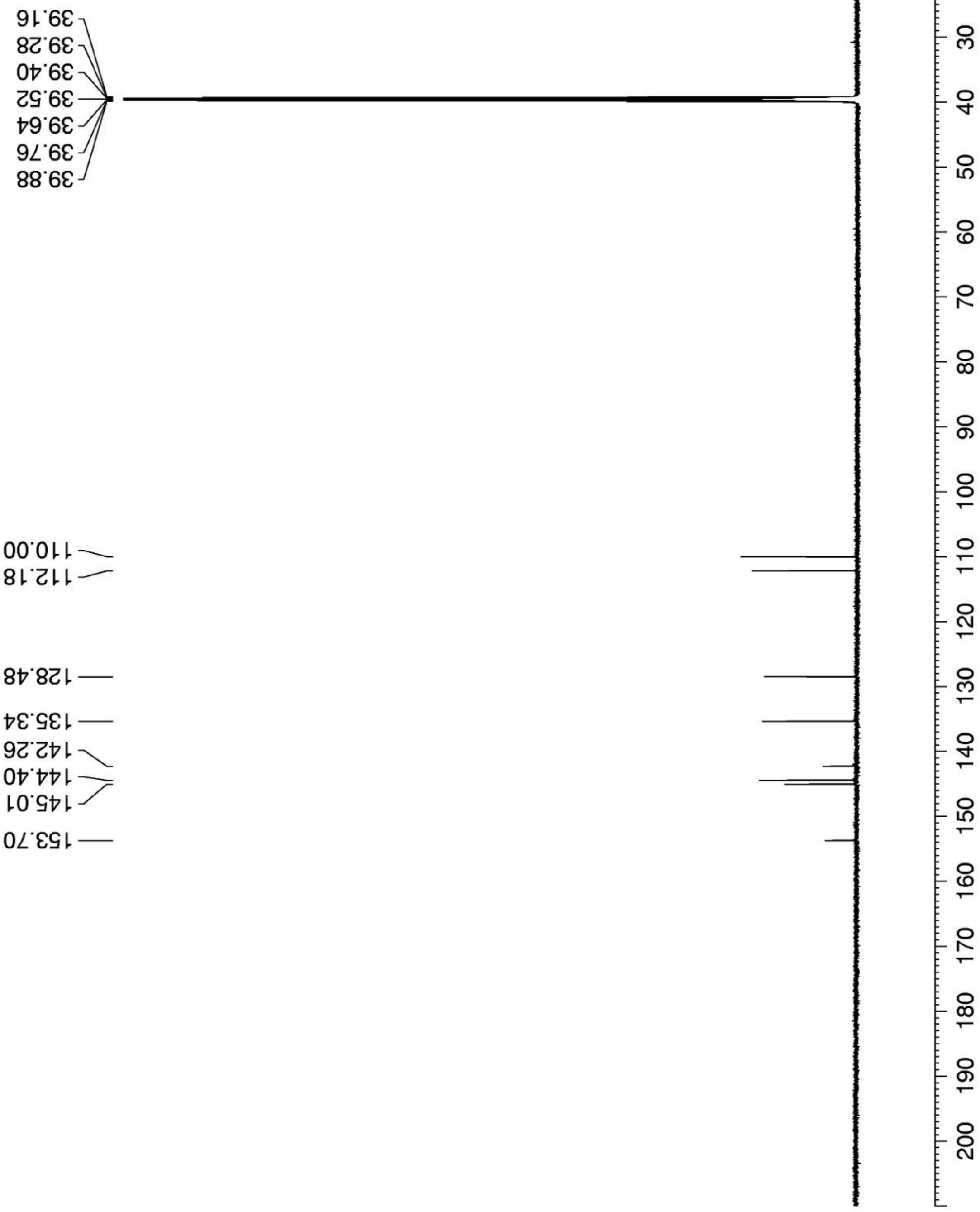

^{1}H NMR ($(CD_3)_2SO$, 25 ºC) of **1n**

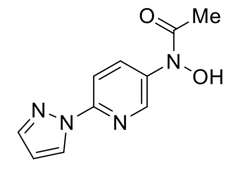

1n

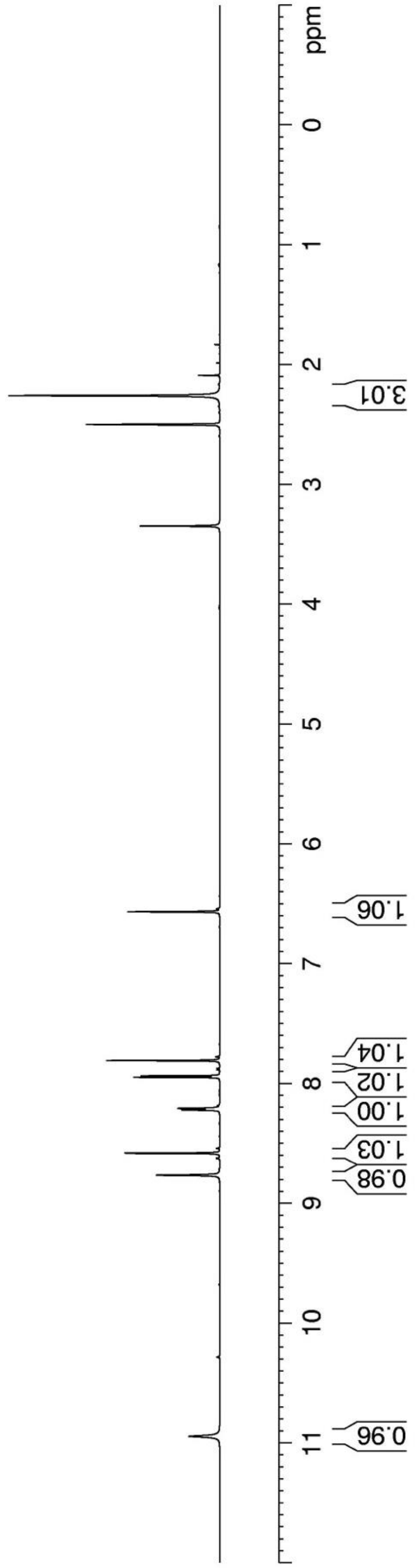

^{13}C NMR (($(CD_3)_2SO$, 25 ºC) of **1n**

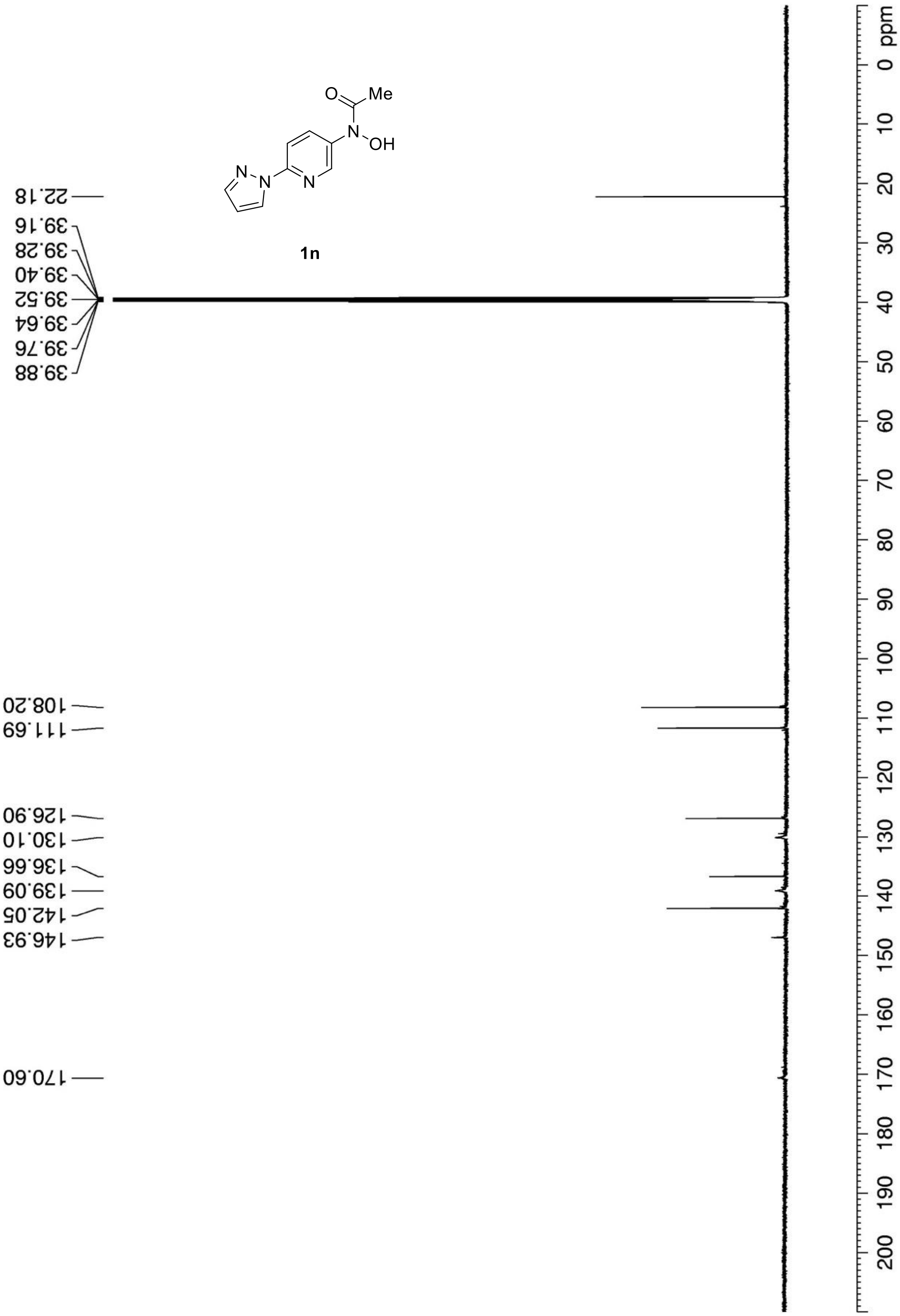

^{1}H NMR ($(CD_3)_2SO$, 25 ºC) of **S12**

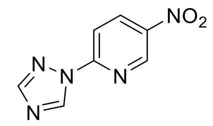

S12

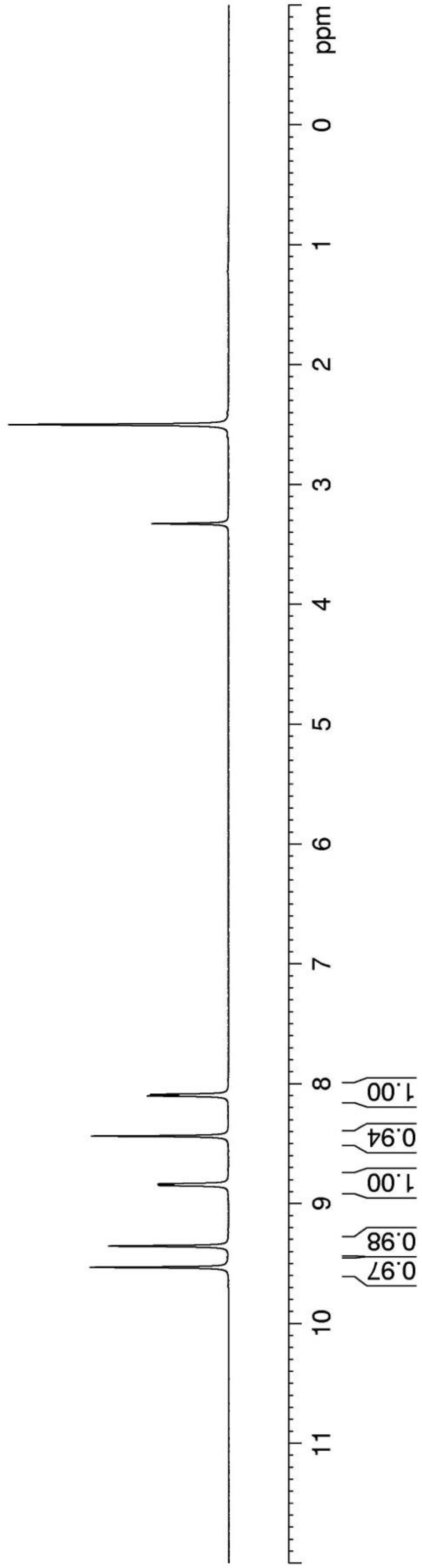

^{13}C NMR ($(CD_3)_2SO$, 25 ºC) of **S12**

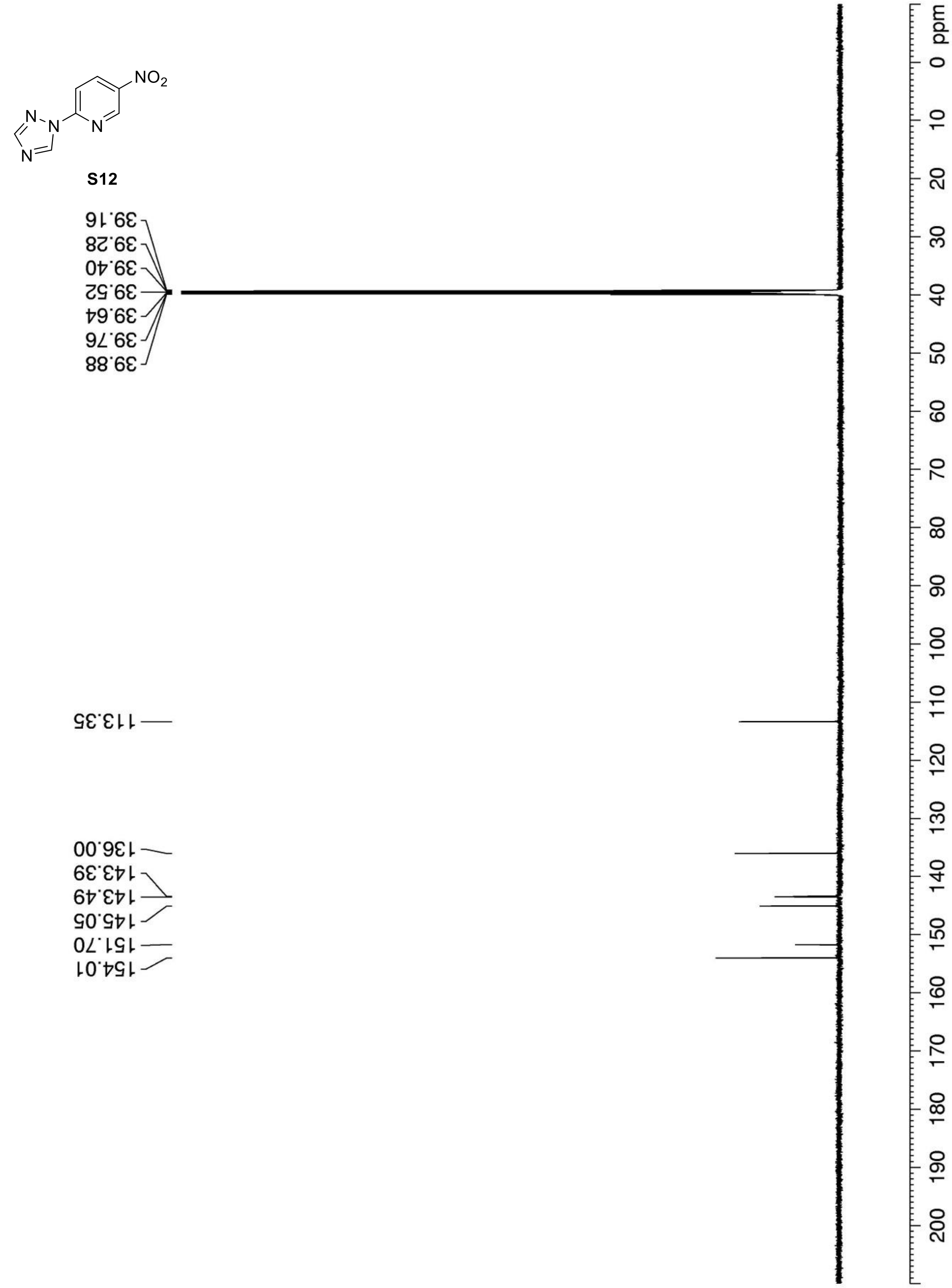

^{1}H NMR ($(CD_3)_2SO$, 25 ºC) of **S13**

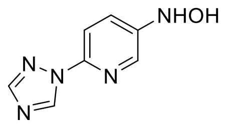

S13

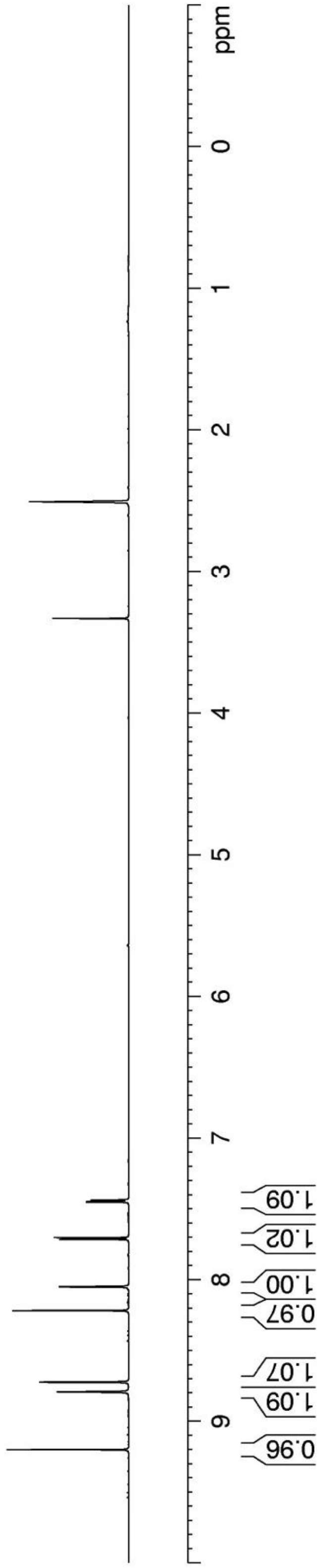

^{13}C NMR ($(CD_3)_2SO$, 25 ºC) of **S13**

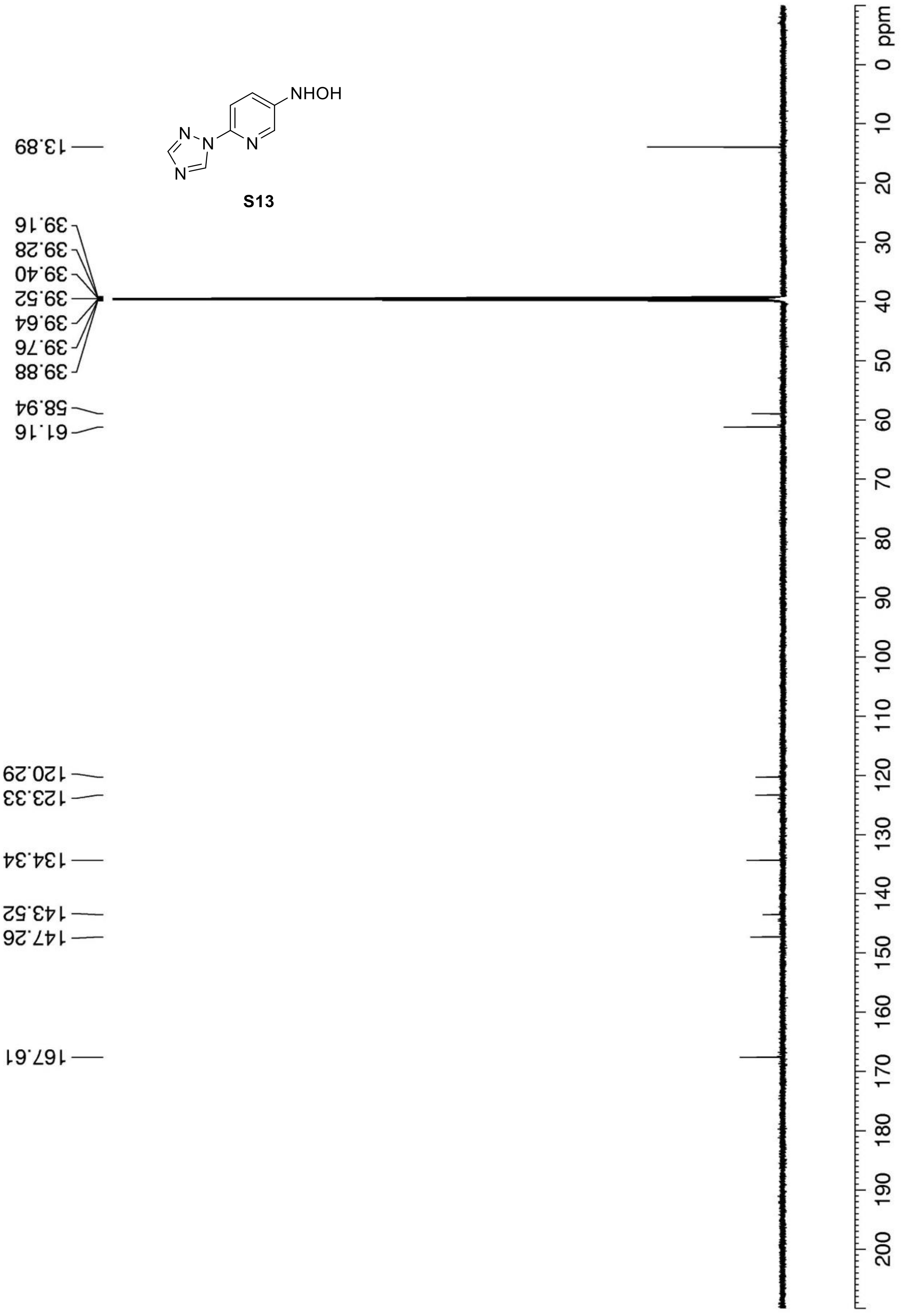

^{1}H NMR ($(CD_3)_2SO$, 25 ºC) of **1o**

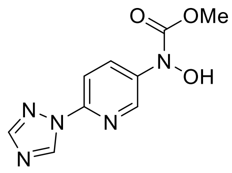

1o

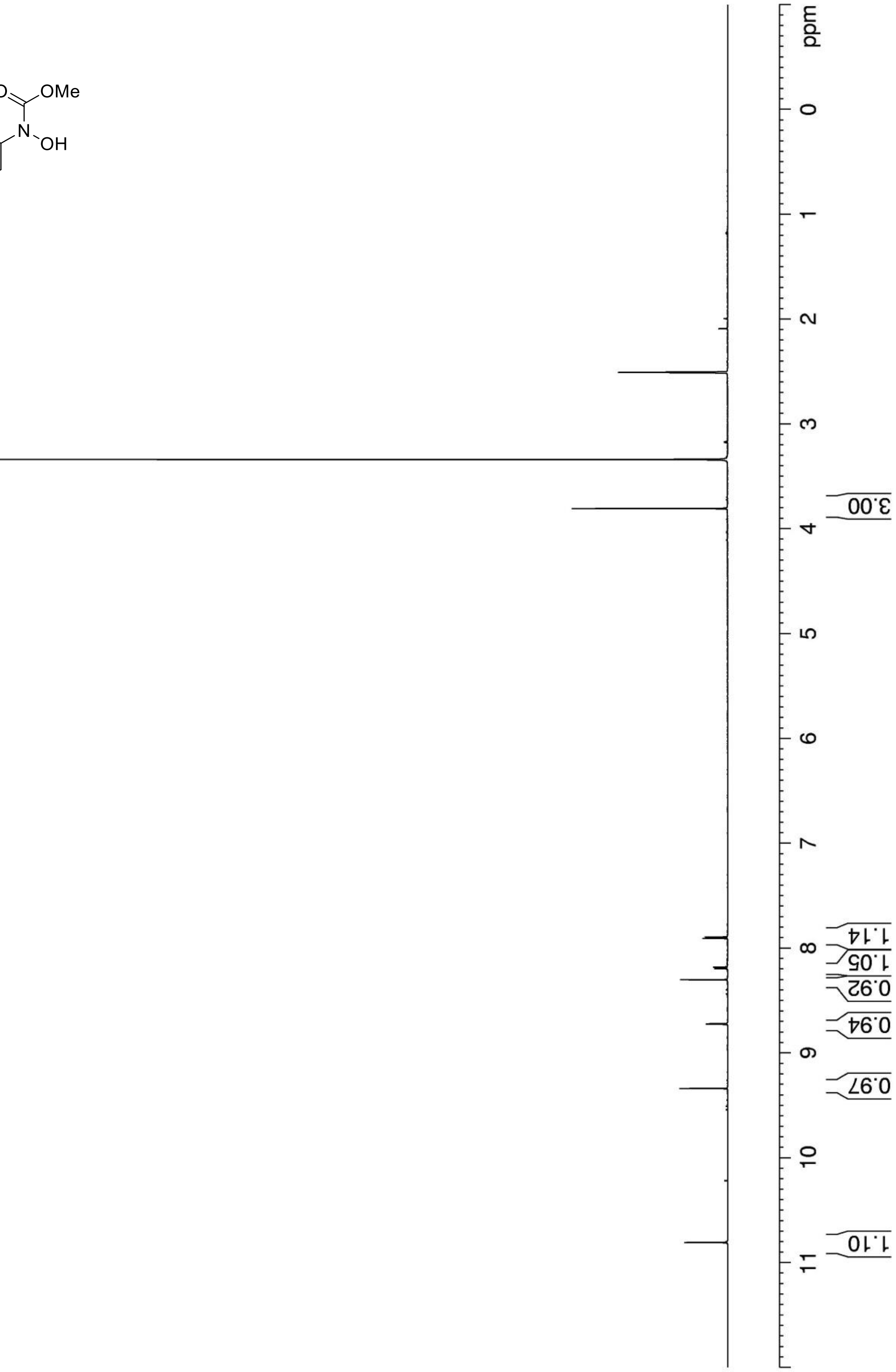

^{13}C NMR (($(CD_3)_2SO$, 25 ºC) of **1o**

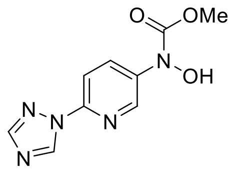

1o

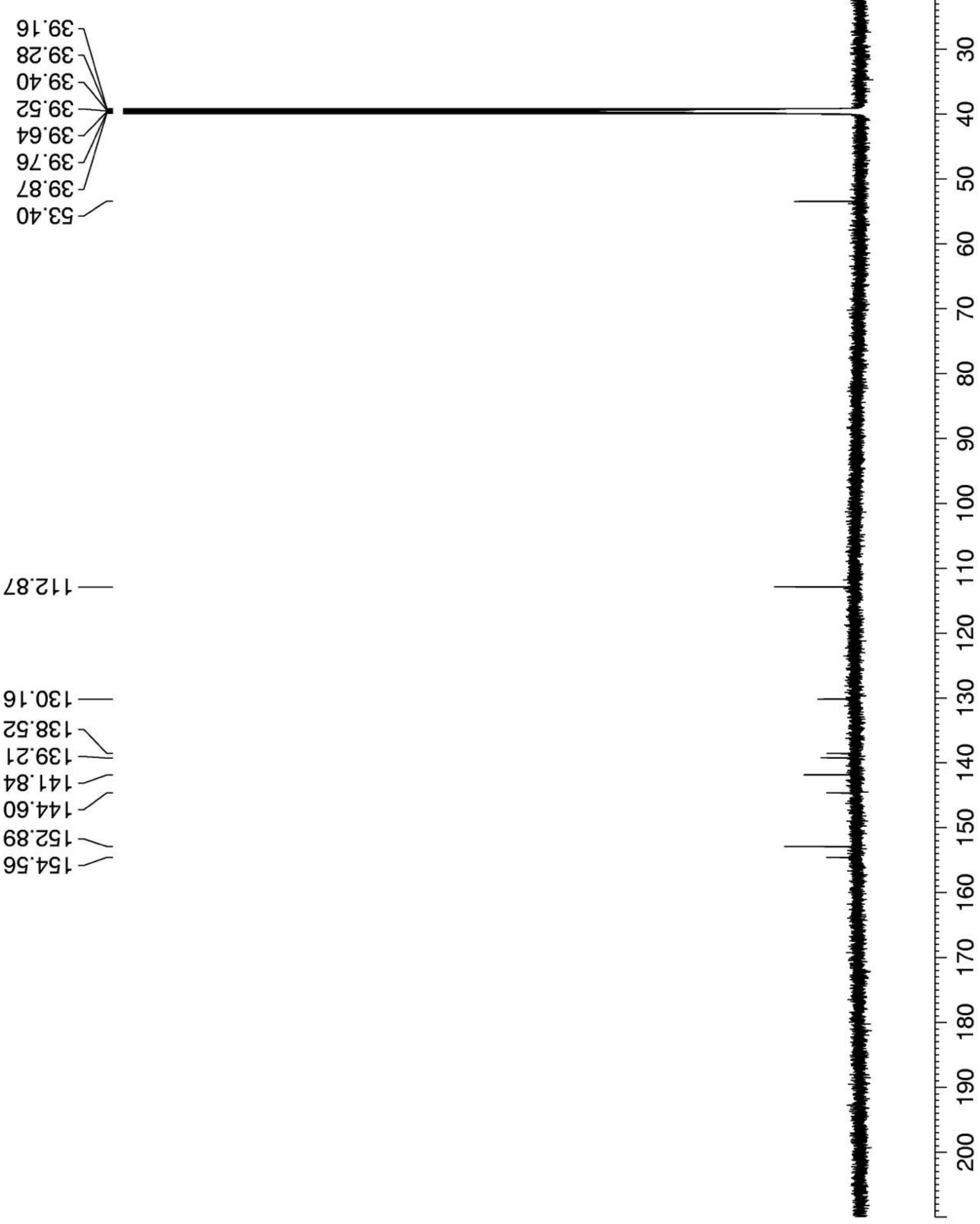

^{1}H NMR ($CDCl_3$, 25 ºC) of **S14**

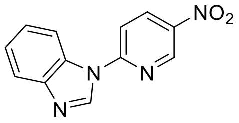

S14

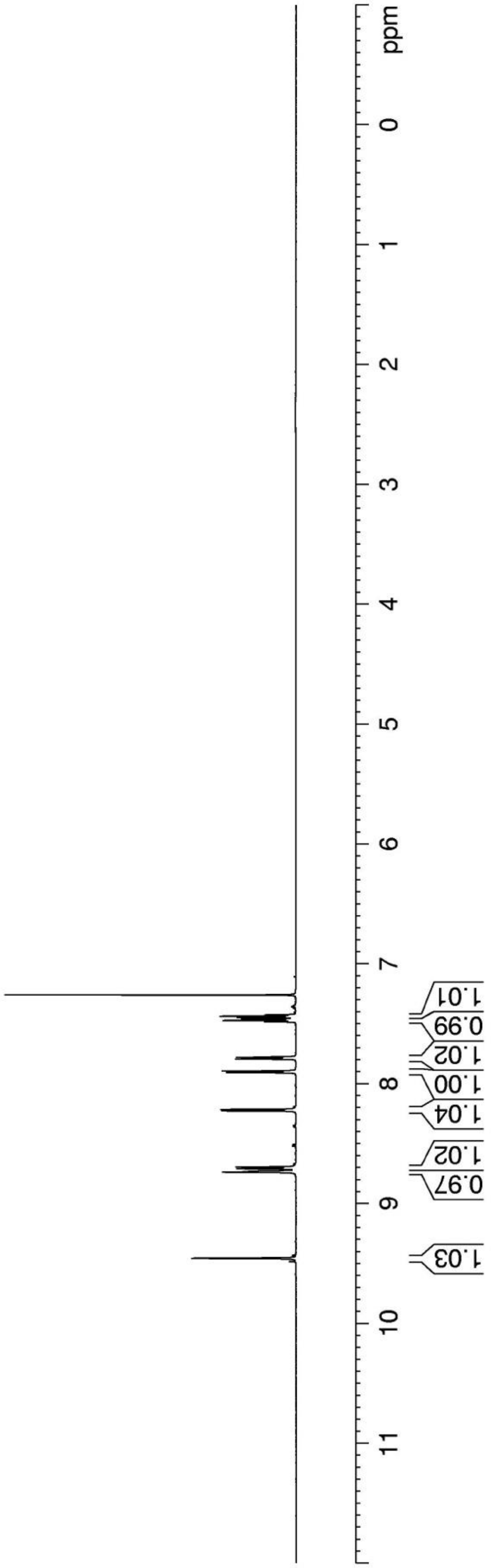

^{13}C NMR ($CDCl_3$, 25 ºC) of **S14**

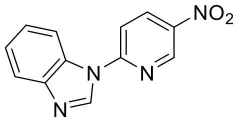

S14

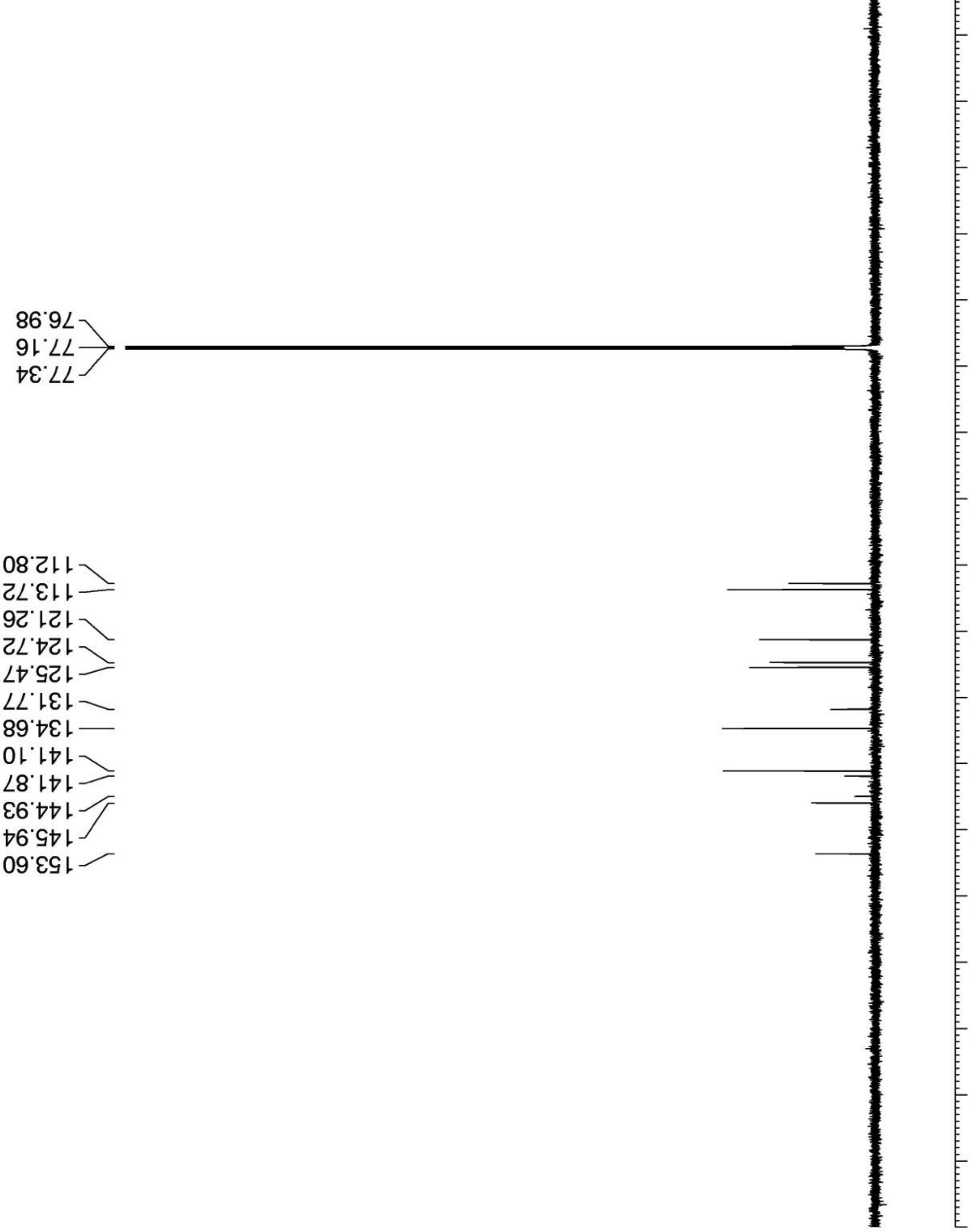

^{1}H NMR ((CD_3)$_2$SO, 25 ºC) of **1p**

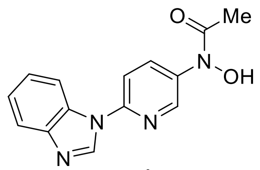

1p

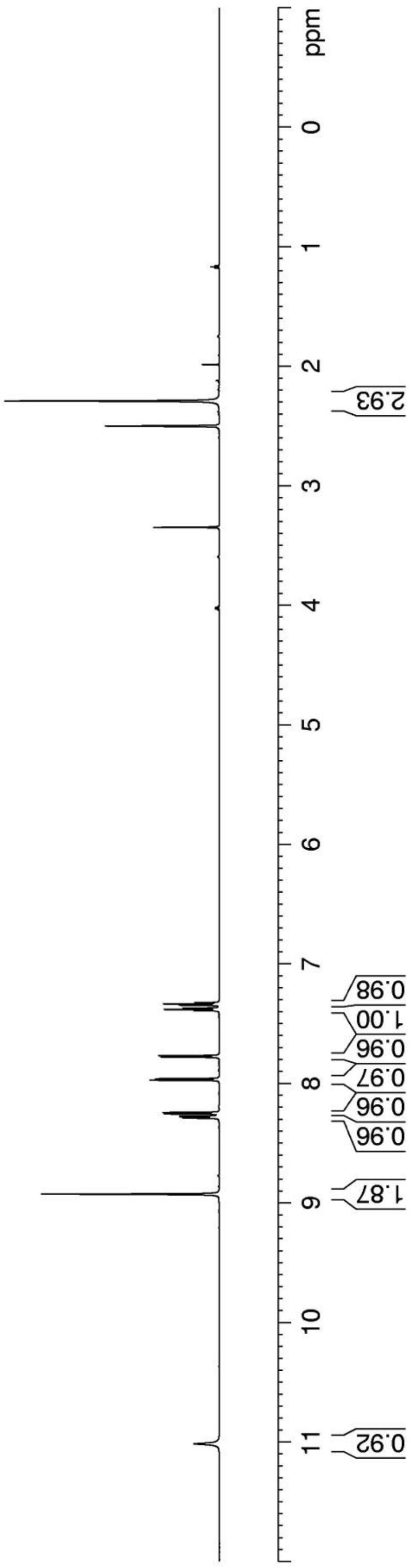

^{13}C NMR ($(CD_3)_2SO$, 25 ºC) of **1p**

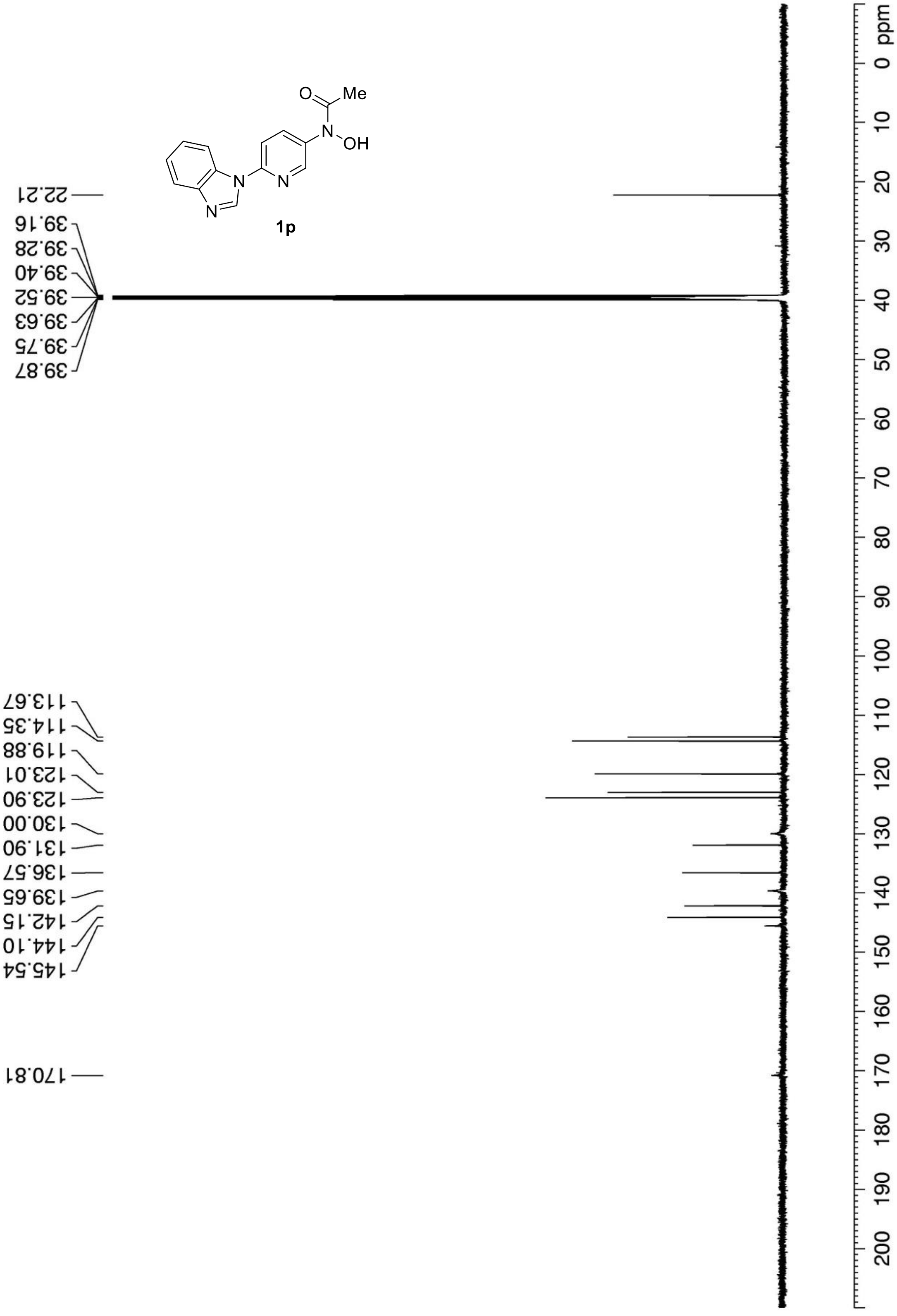

^{1}H NMR ($(CD_3)_2SO$, 25 ºC) of **S15**

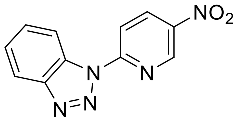

S15

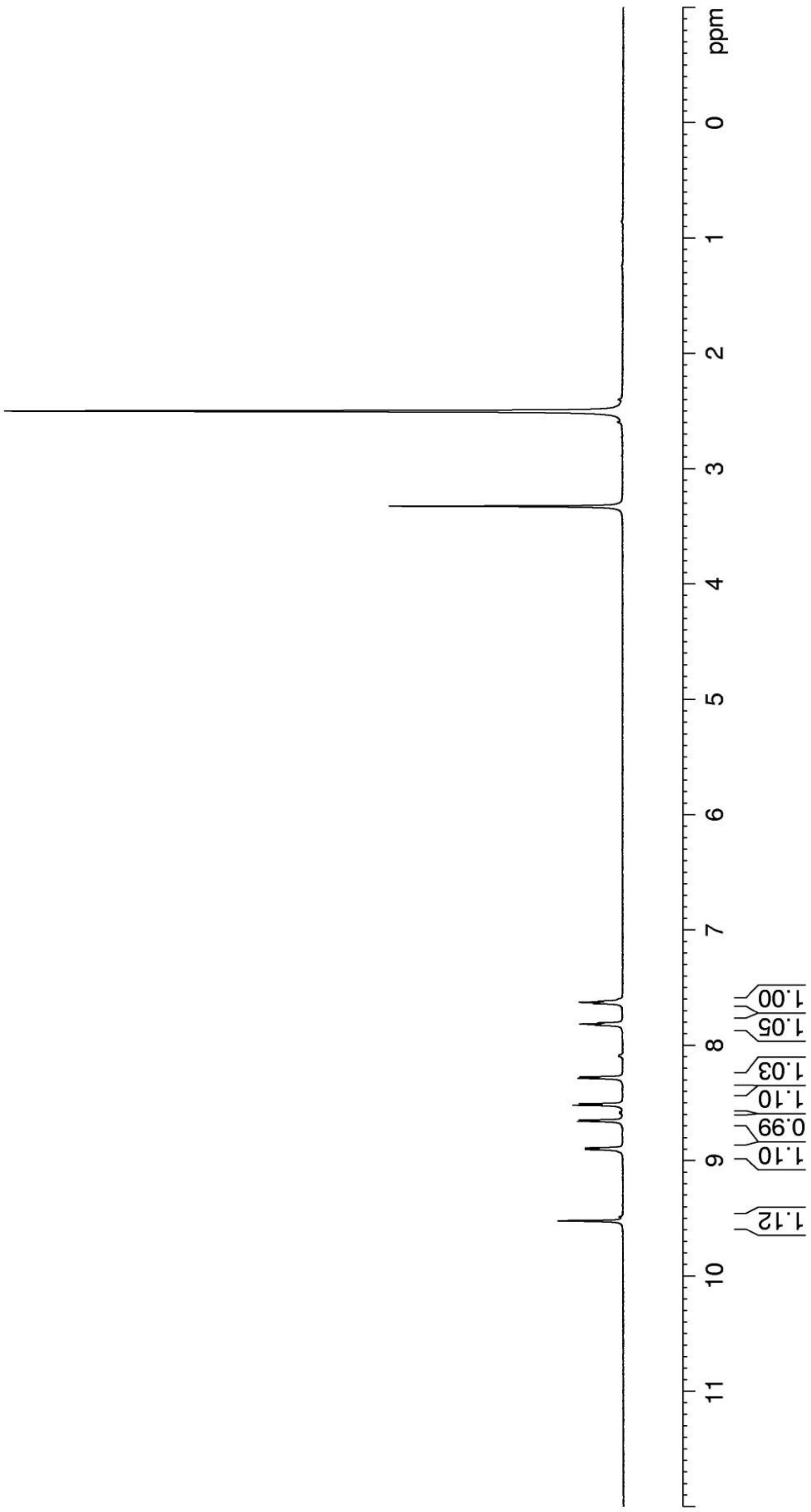

^{13}C NMR ($(CD_3)_2SO$, 25 ºC) of **S15**

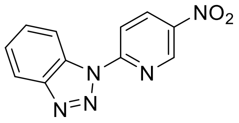

S15

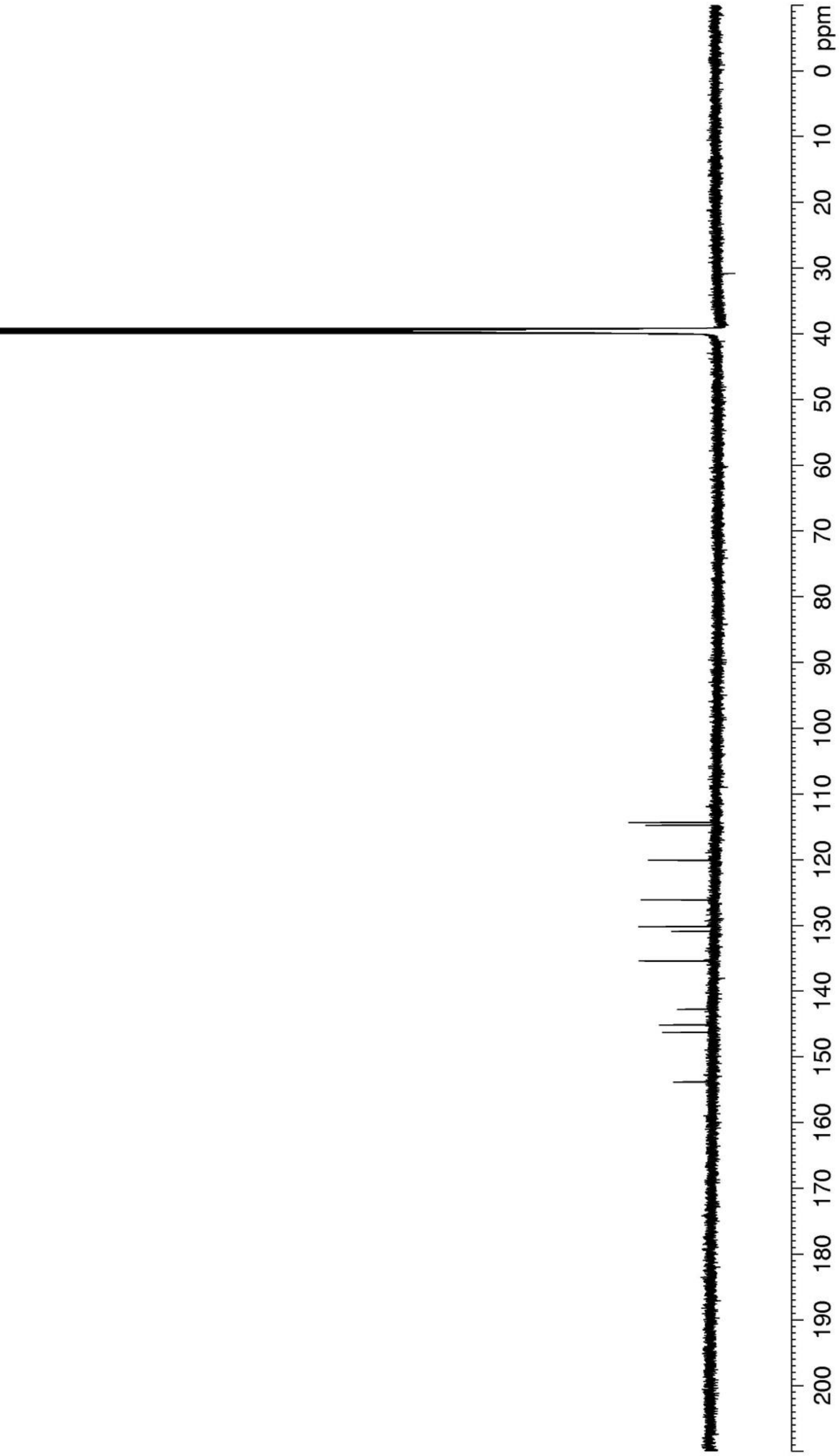

^{1}H NMR ((CD_3)$_2$SO, 25 ºC) of **1q**

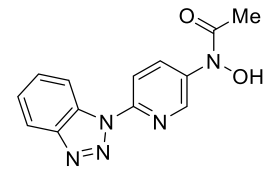

1q

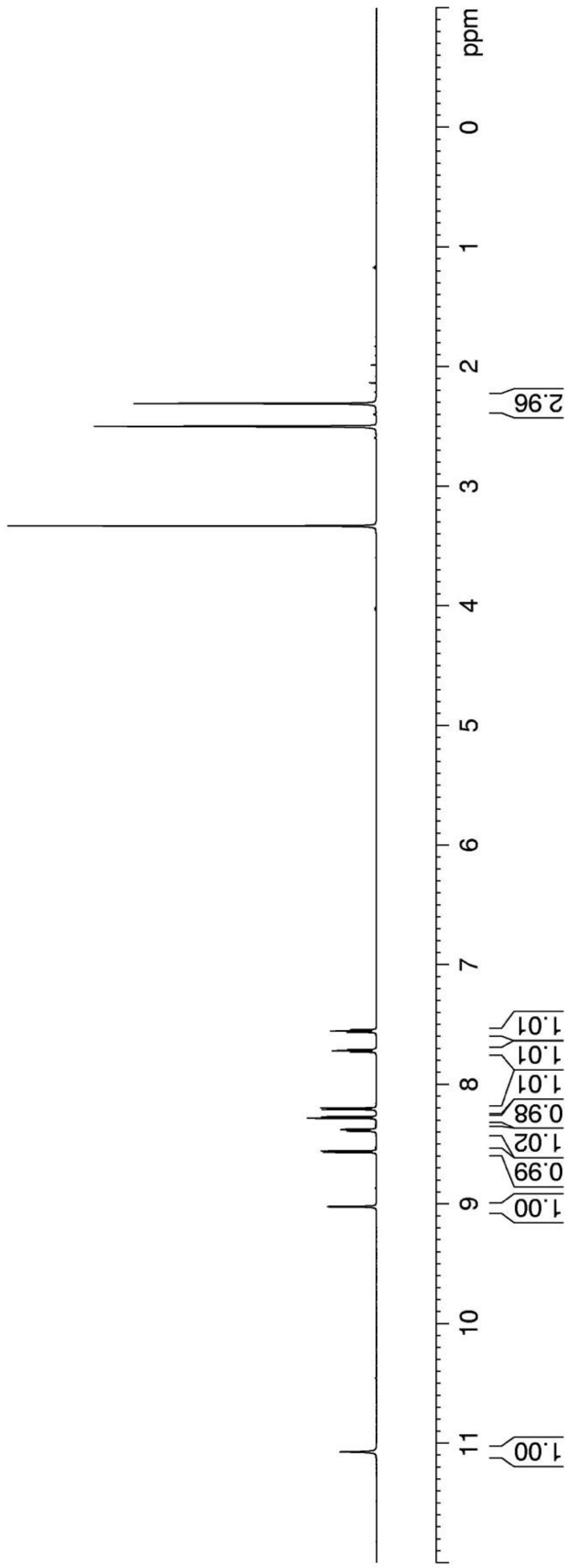

^{13}C NMR (($(CD_3)_2SO$, 25 ºC) of **1q**

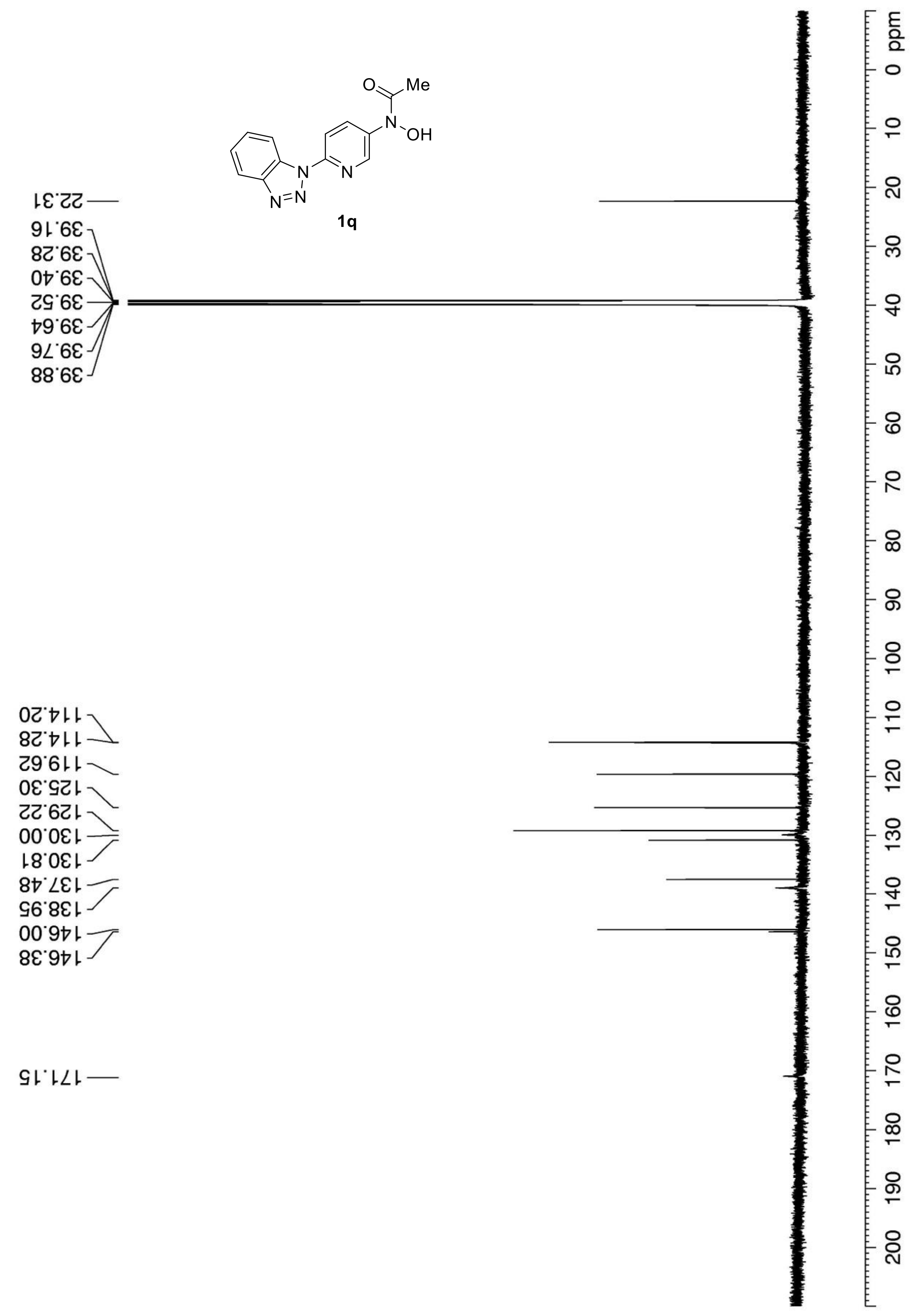

^{1}H NMR ($(CD_3)_2SO$, 25 ºC) of **S16**

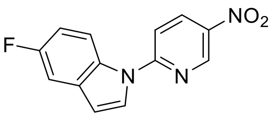

S16

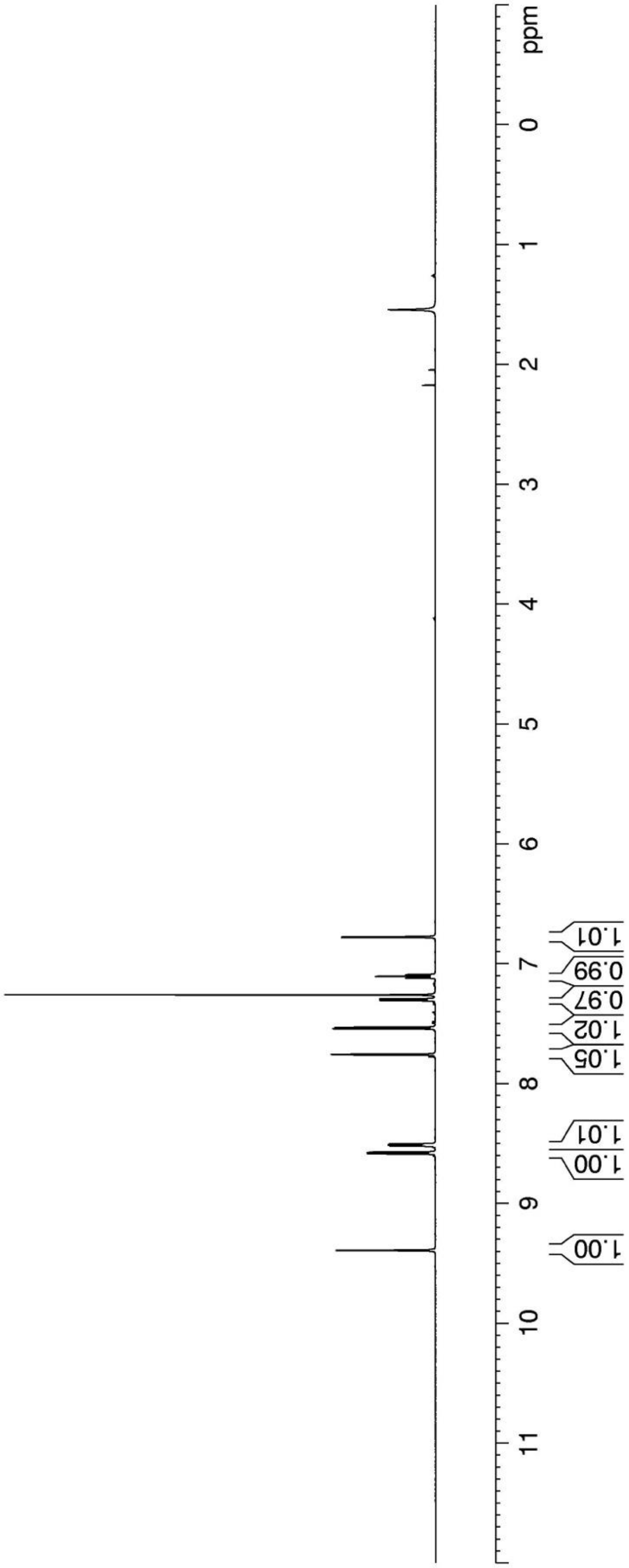

^{13}C NMR ((CD_3)$_2$SO, 25 ºC) of **S16**

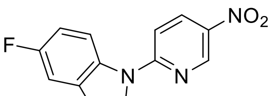

S16

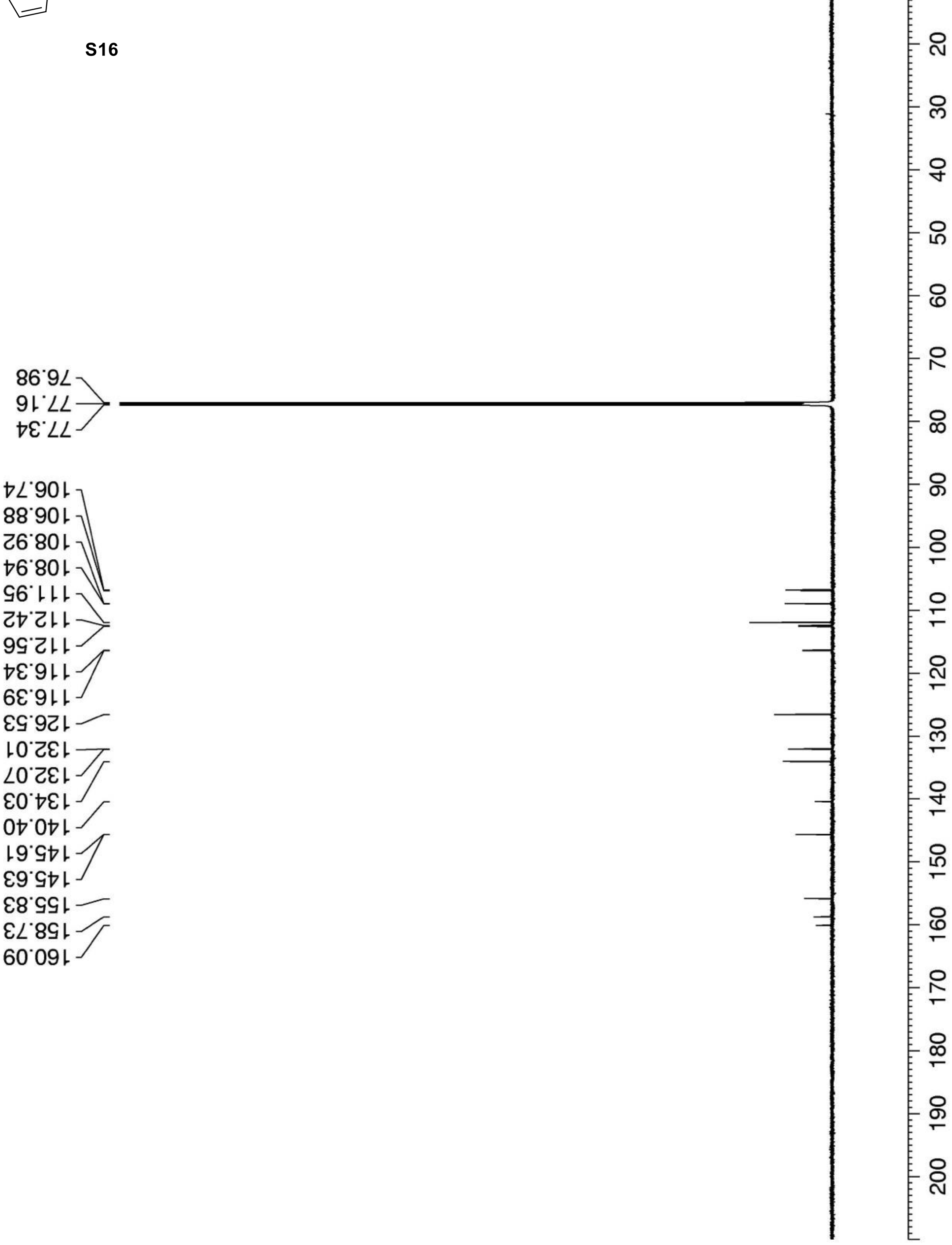

^{19}F NMR ($(CD_3)_2SO$, 25 ºC) of **S16**

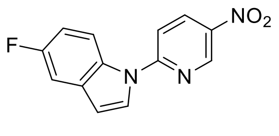

S16

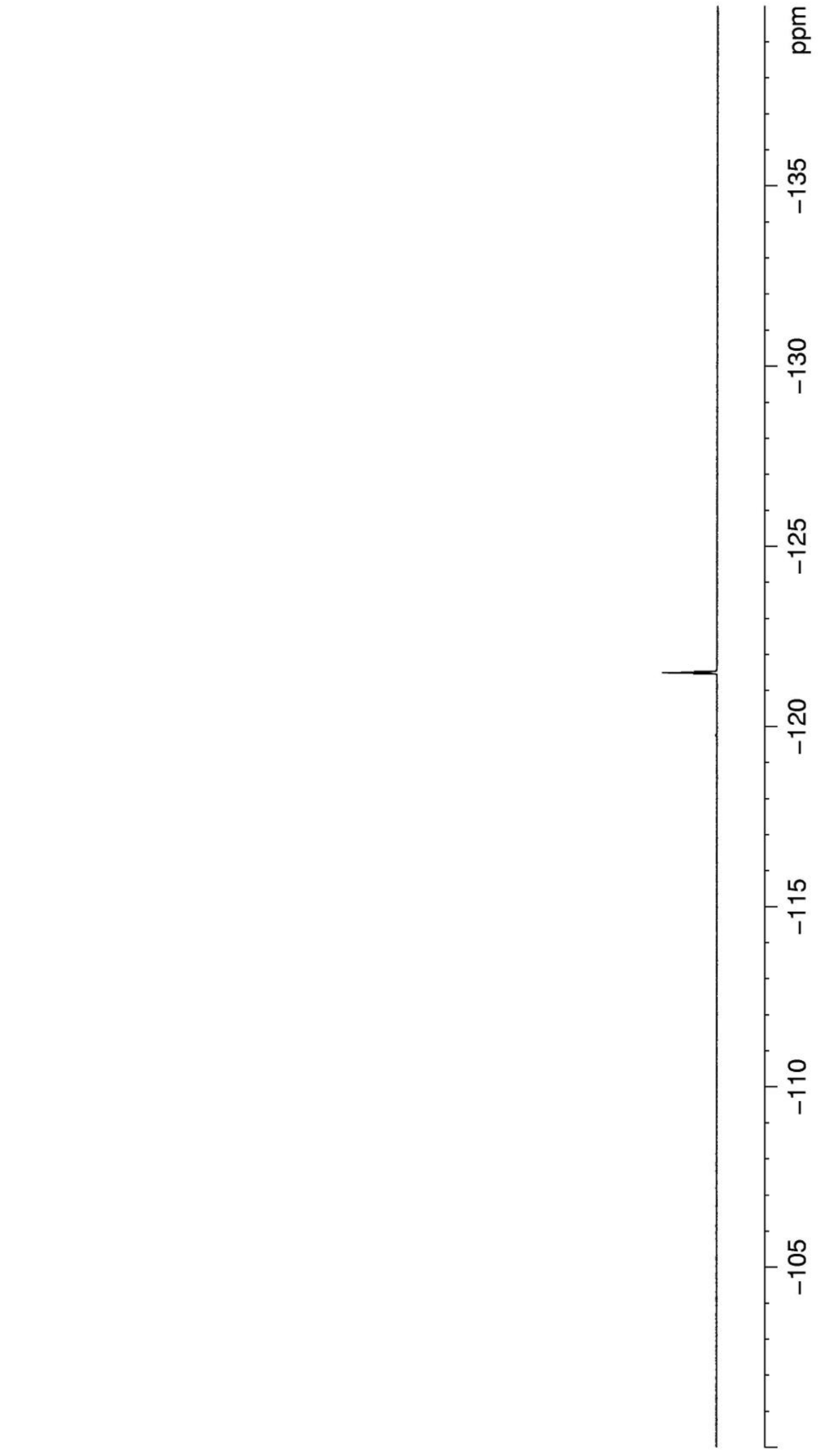

^{1}H NMR ($(CD_3)_2SO$, 25 ºC) of **1r**

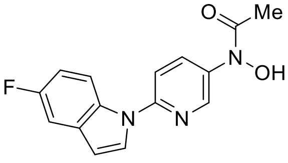

1r

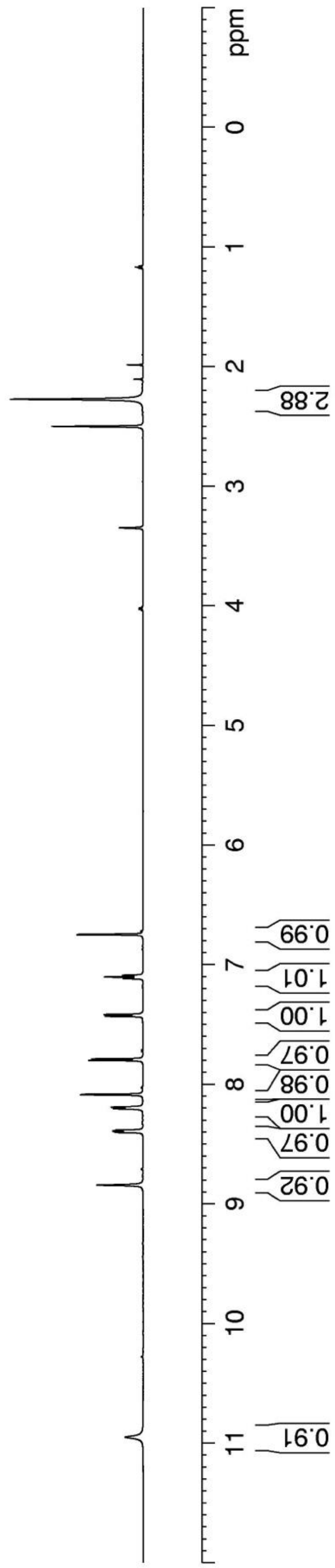

^{13}C NMR ($(CD_3)_2SO$, 25 ºC) of **1r**

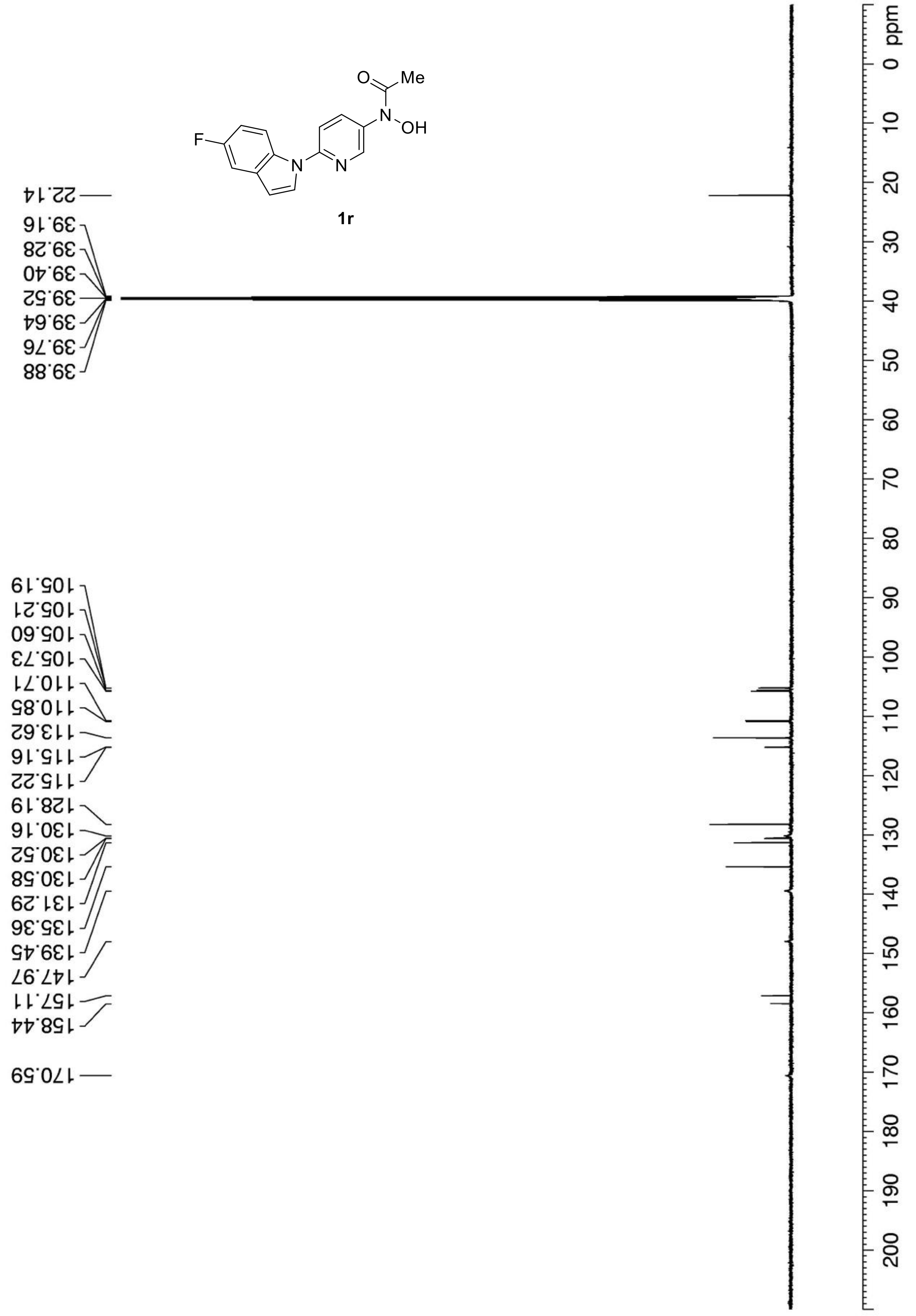

^{19}F NMR ((CD_3)$_2$SO, 25 ºC) of **1r**

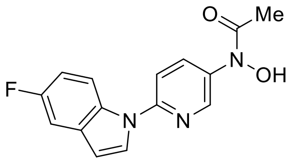

1r

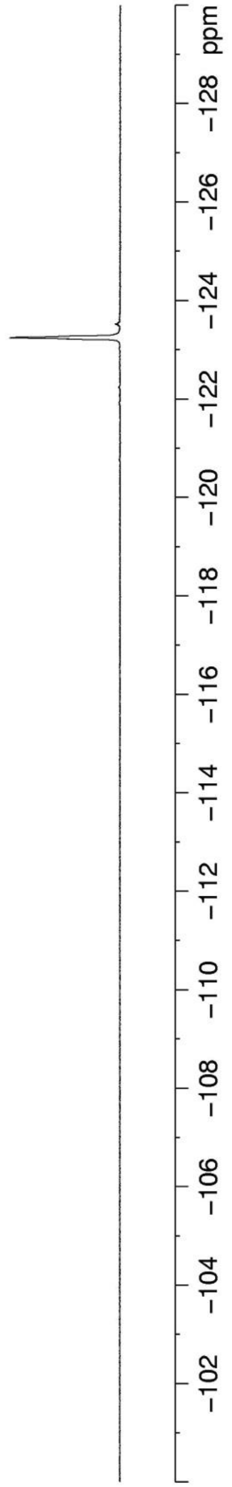

^{1}H NMR ($CDCl_3$, 25 ºC) of **S17**

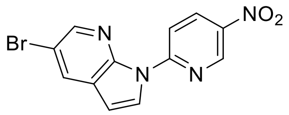

S17

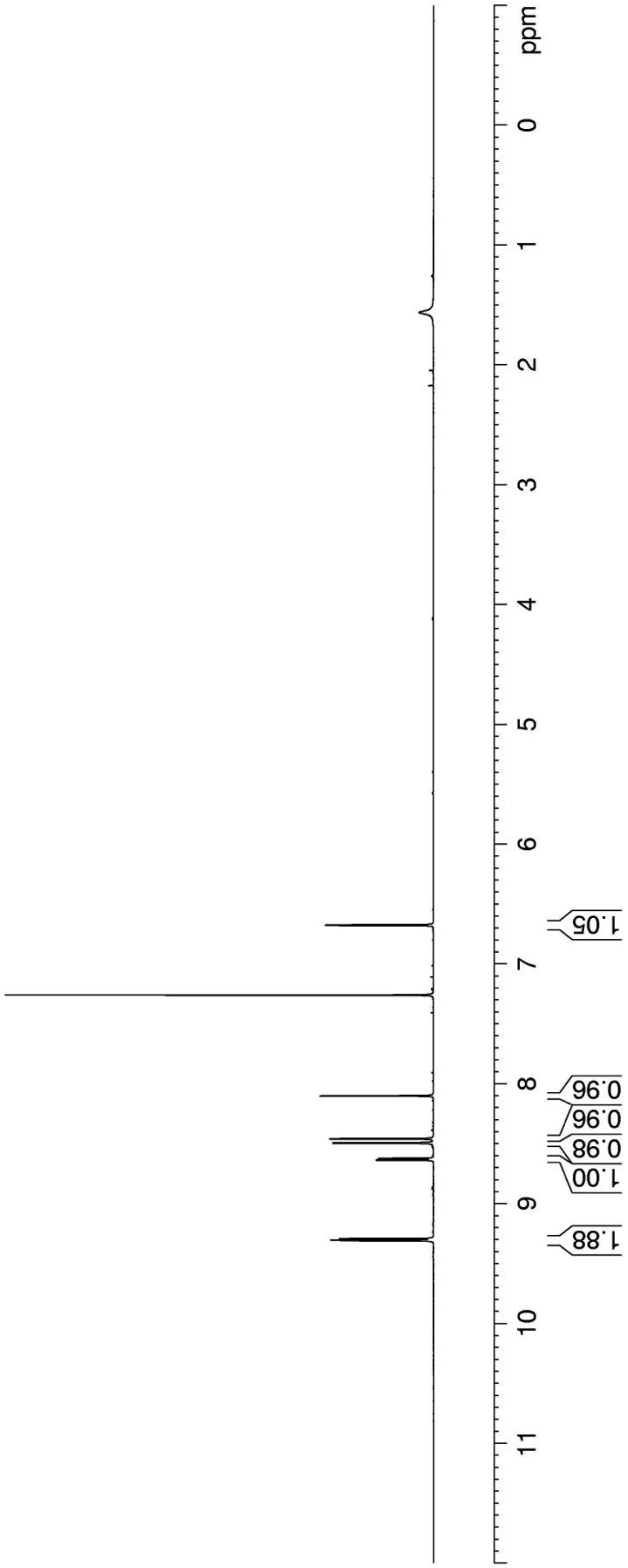

^{13}C NMR ($CDCl_3$, 25 ºC) of **S17**

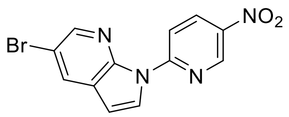

S17

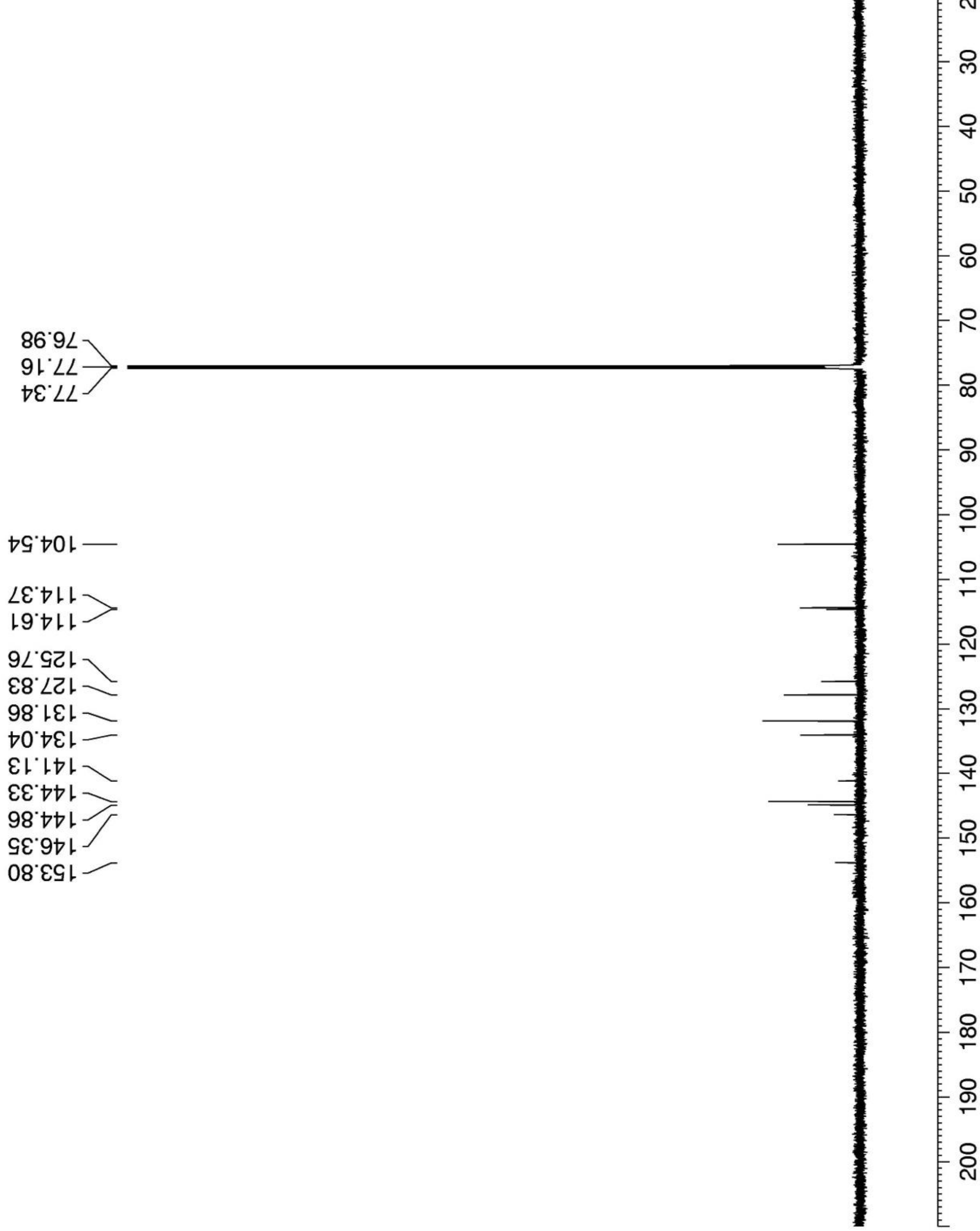

^{1}H NMR ($(CD_3)_2SO$, 25 ºC) of **1s**

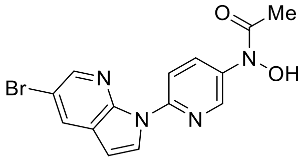

1s

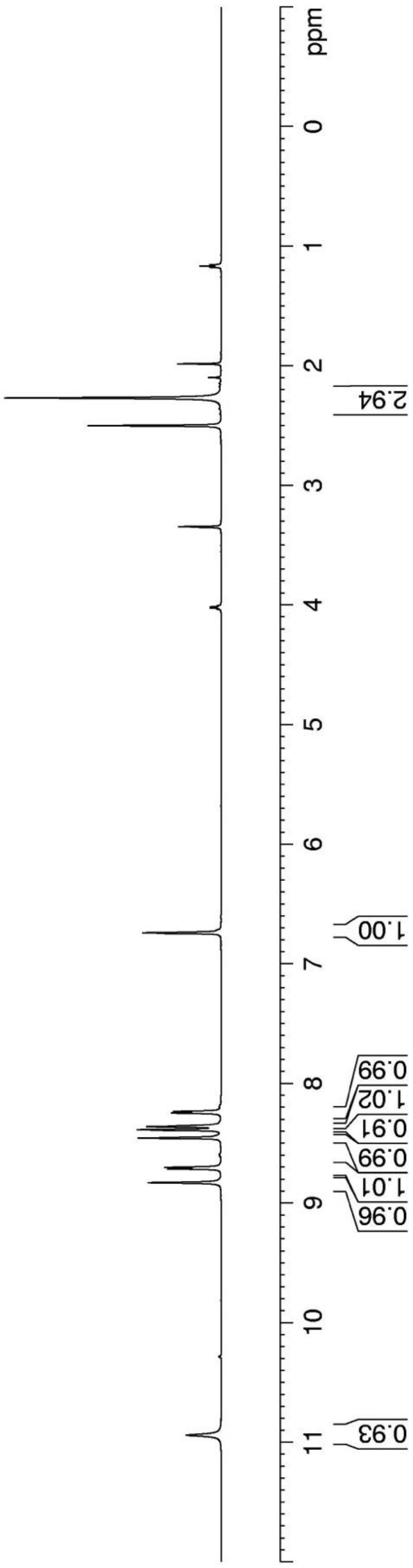

^{13}C NMR ($(CD_3)_2SO$, 25 ºC) of **1s**

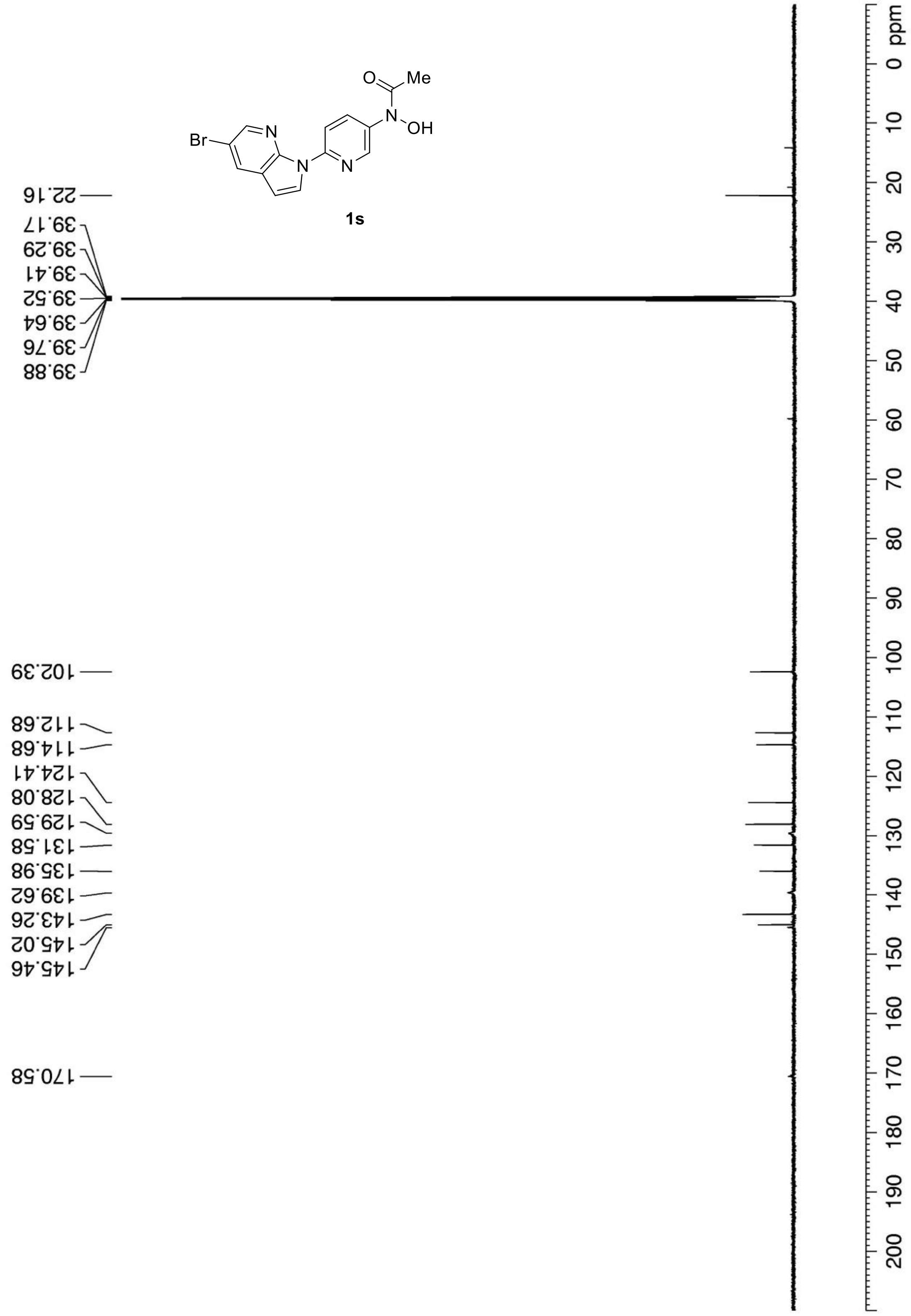

^{1}H NMR ($CDCl_3$, 25 ºC) of **S18**

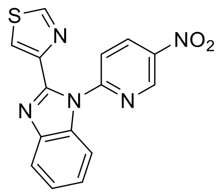

S18

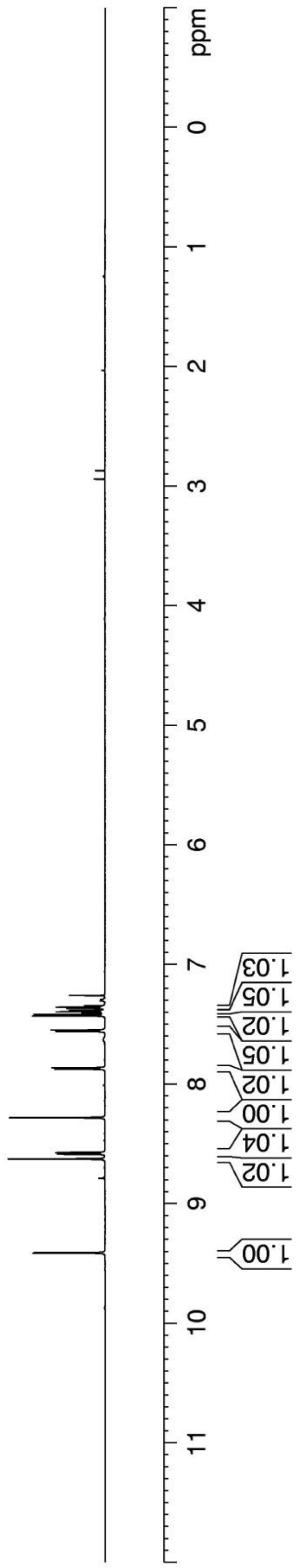

^{13}C NMR ($CDCl_3$, 25 ºC) of **S18**

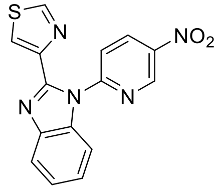

S18

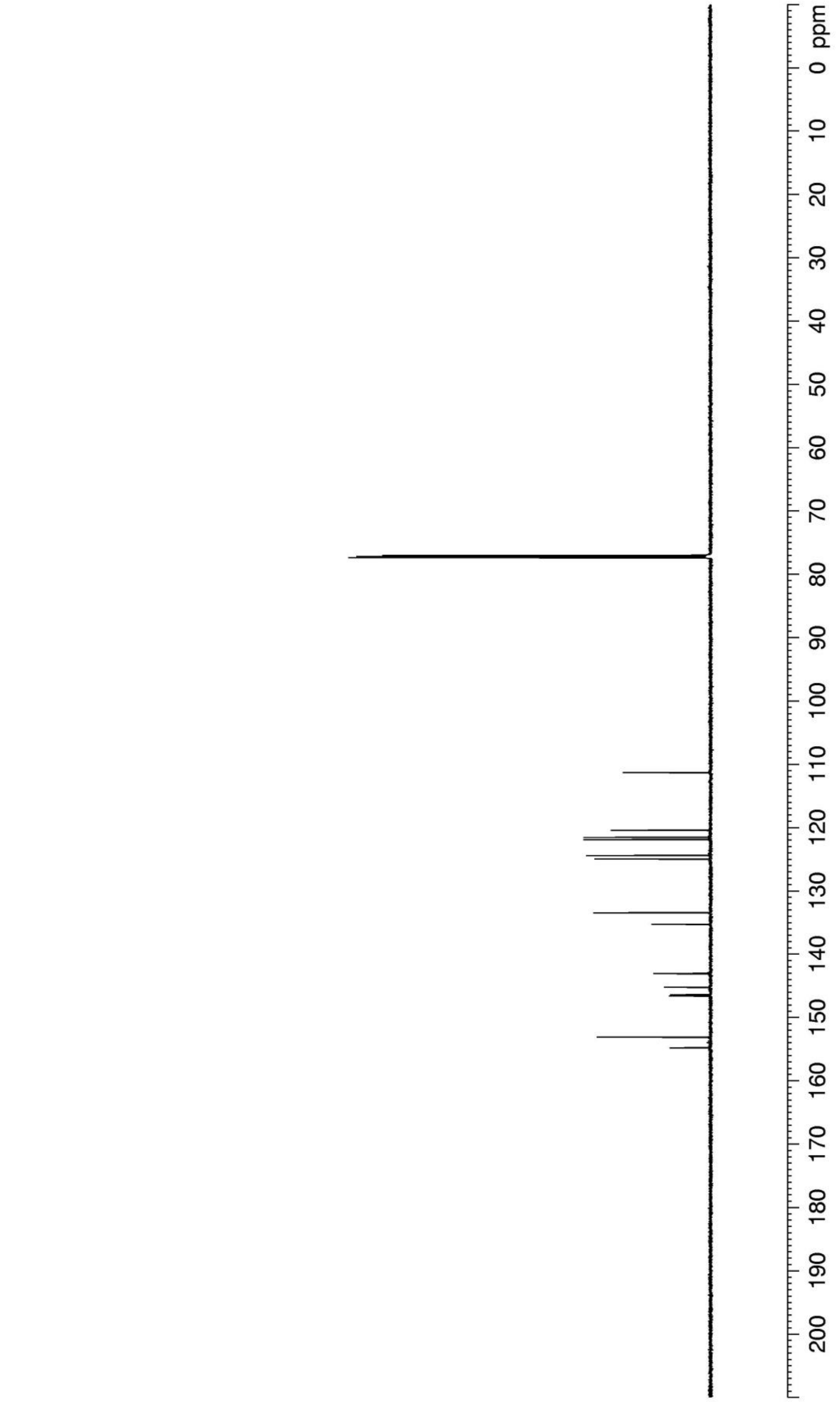

^{1}H NMR ($(CD_3)_2SO$, 25 ºC) of **1t**

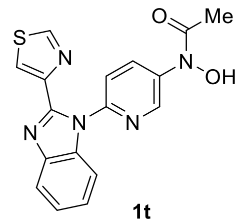

1t

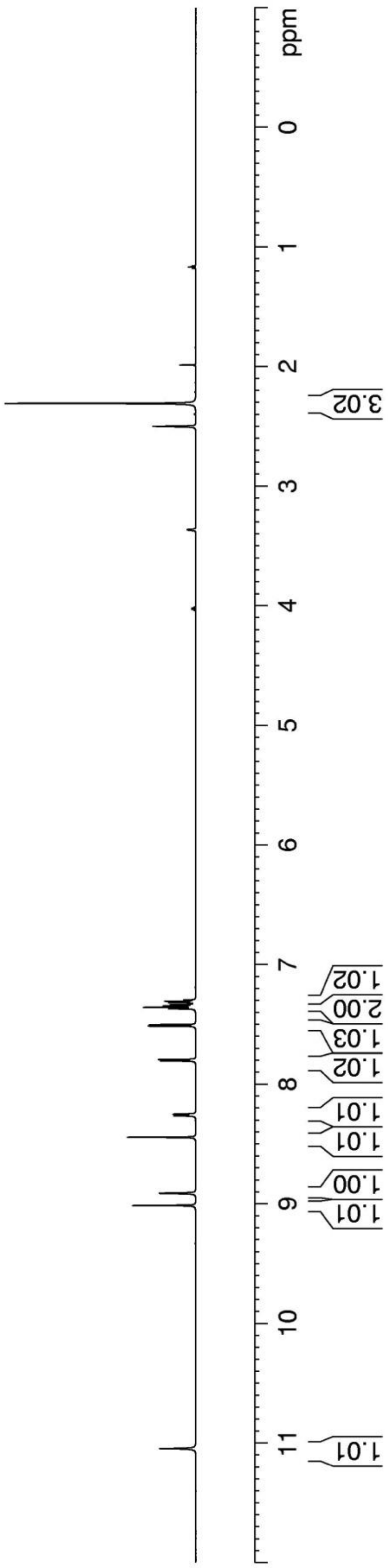

^{13}C NMR ((CD$_3$)$_2$SO, 25 ºC) of **1t**

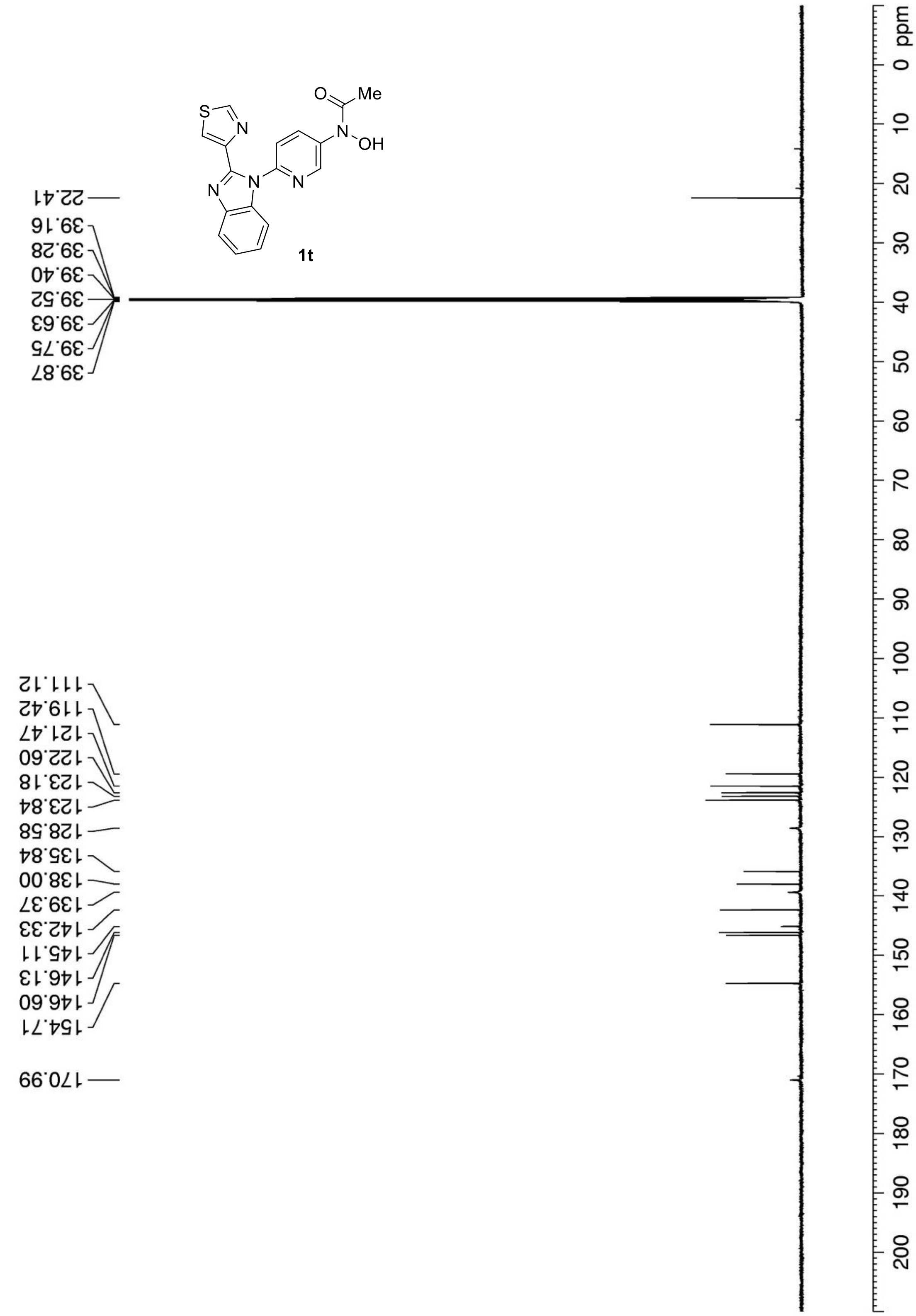

^{1}H NMR ($CDCl_3$, 25 ºC) of **S19**

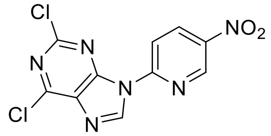

S19

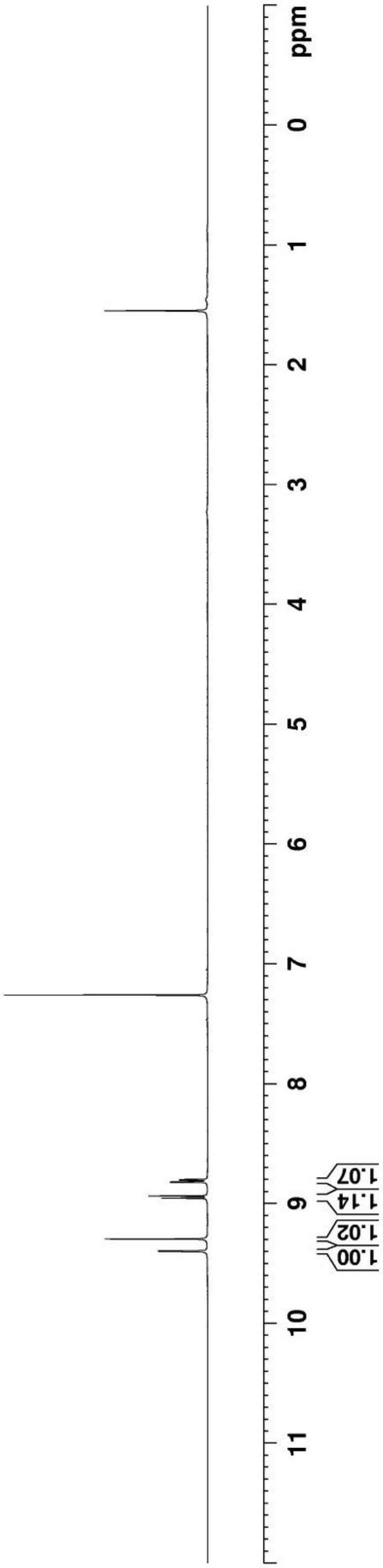

^{13}C NMR ($CDCl_3$, 25 ºC) of **S19**

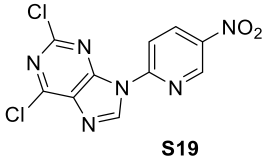

S19

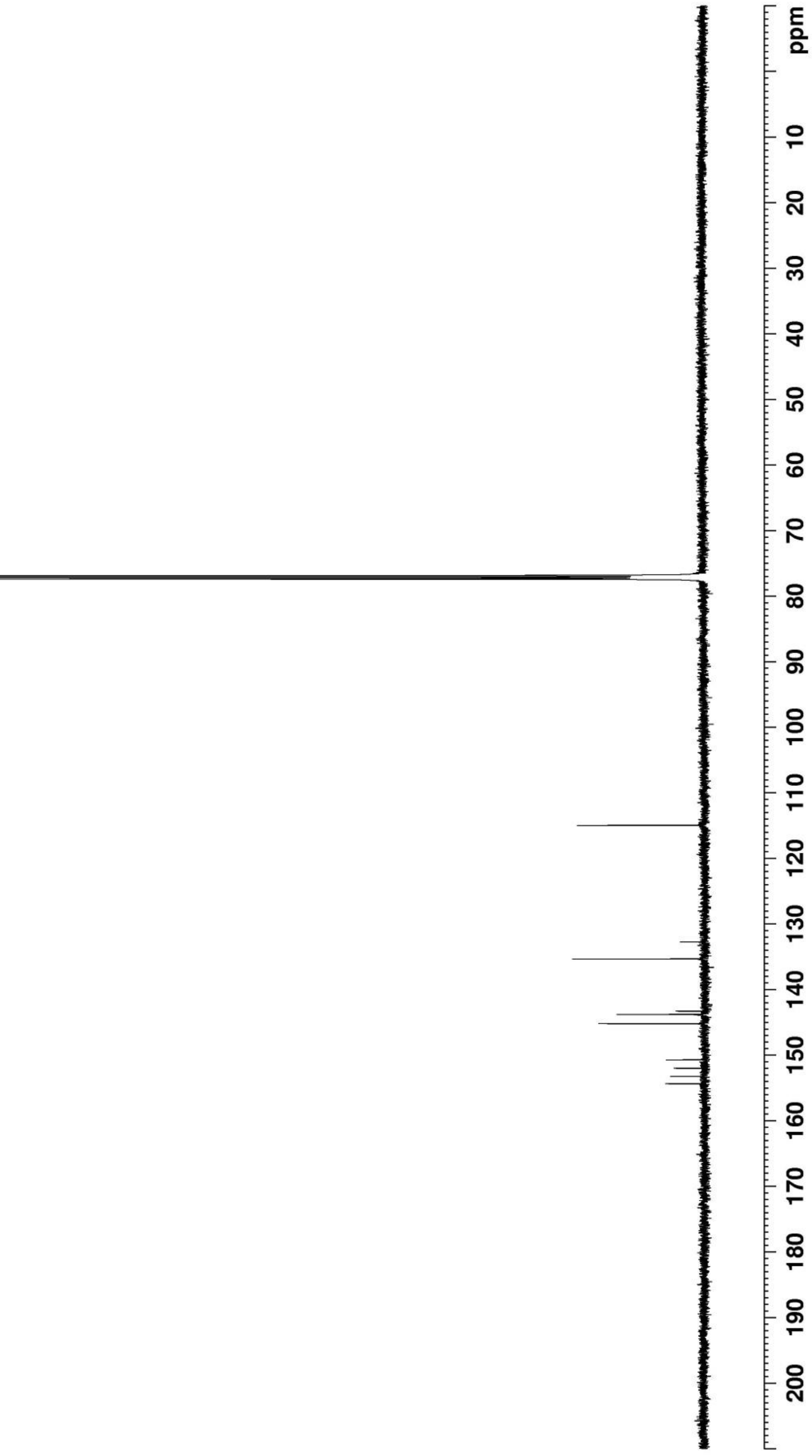

^{1}H NMR (($(CD_3)_2SO$, 25 ºC) of **1u**

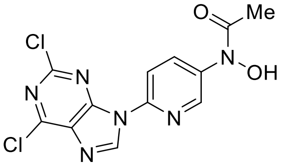

1u

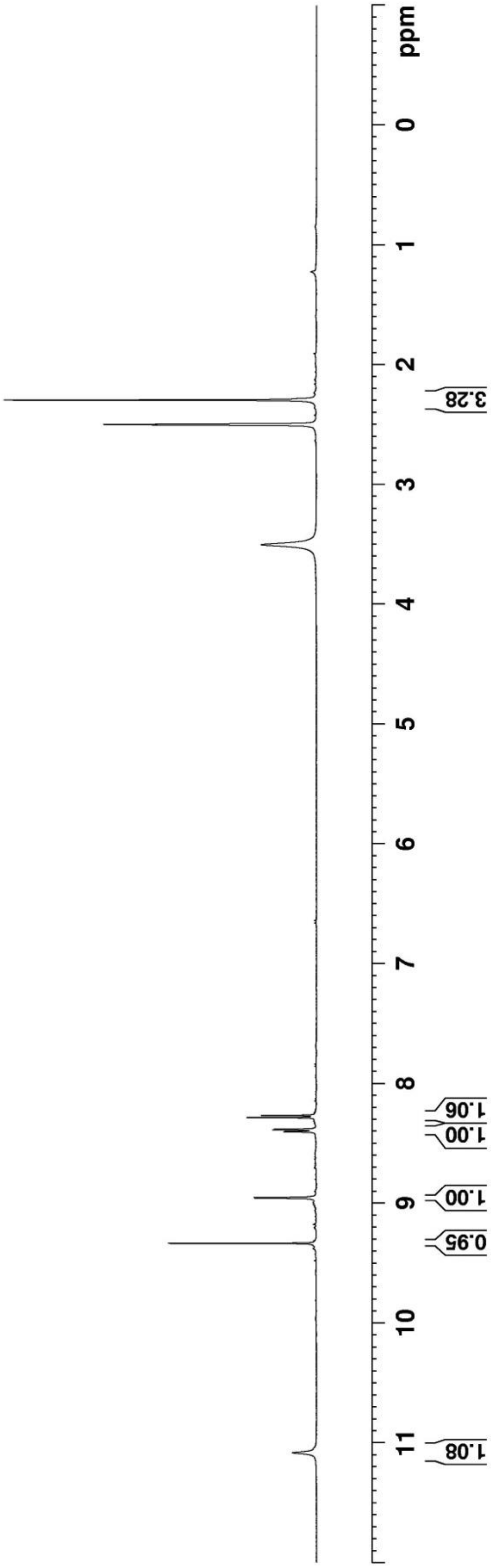

^{13}C NMR ($(CD_3)_2SO$, 25 ºC) of **1u**

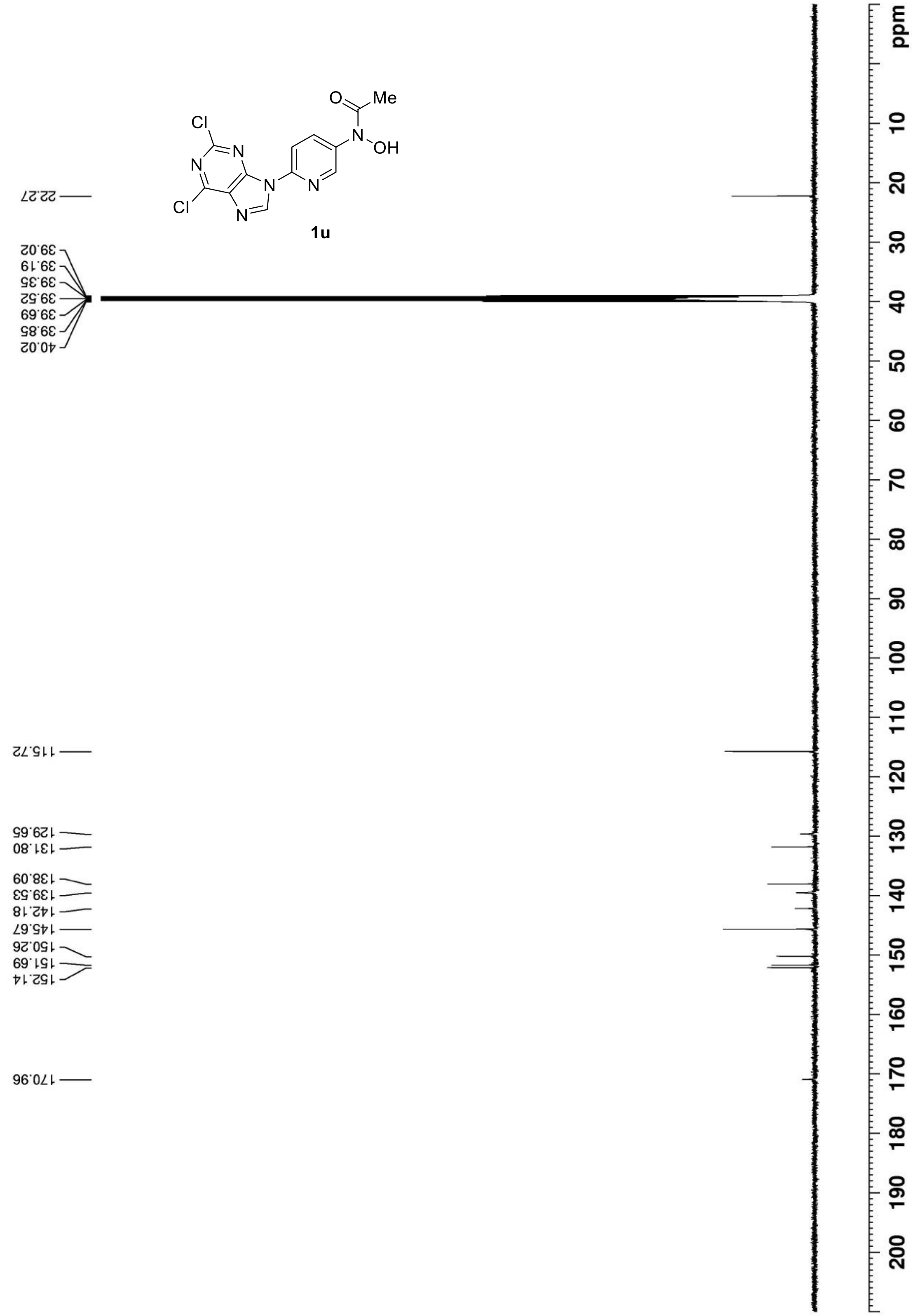

^{1}H NMR (($(CD_3)_2SO$, 25 ºC) of **1v**

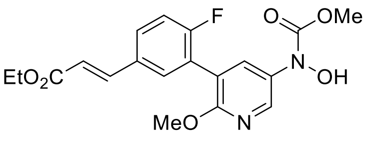

1v

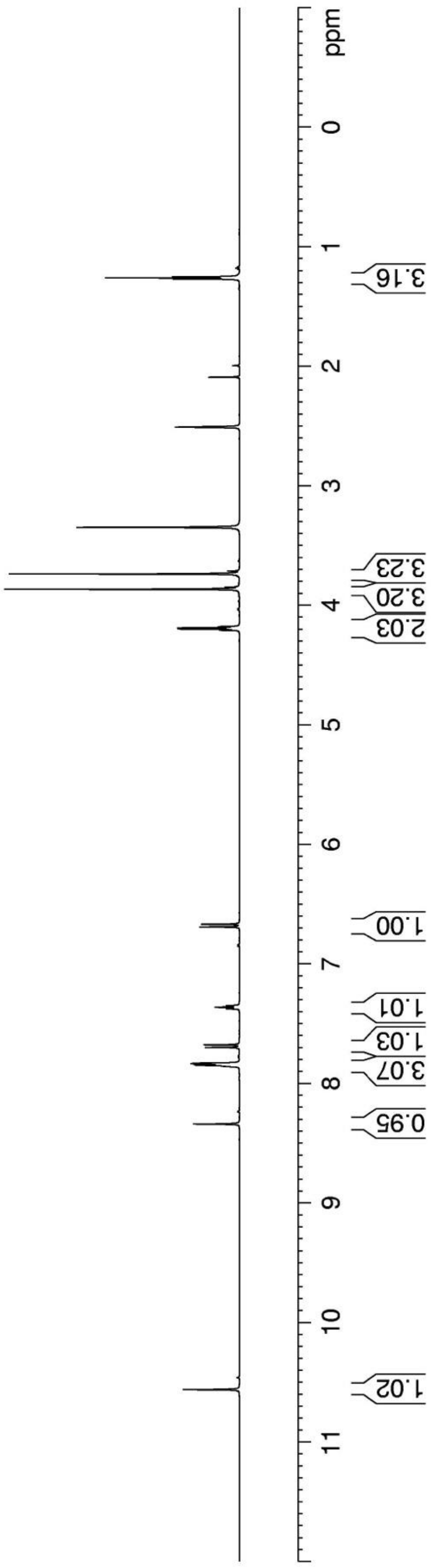

^{13}C NMR ($(CD_3)_2SO$, 25 ºC) of **1v**

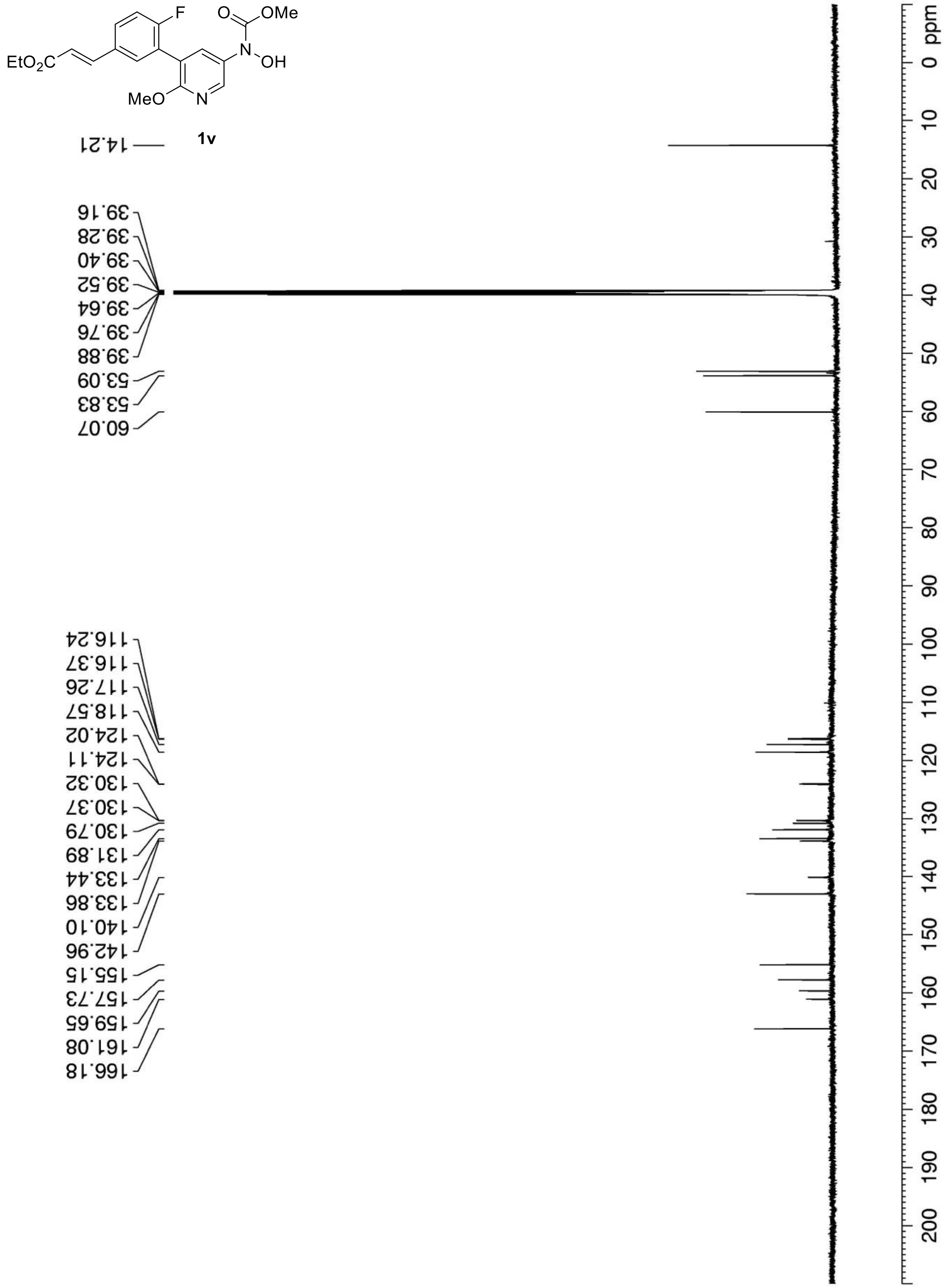

^{19}F NMR ($(CD_3)_2SO$, 25 ºC) of **1v**

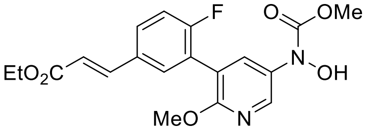

1v

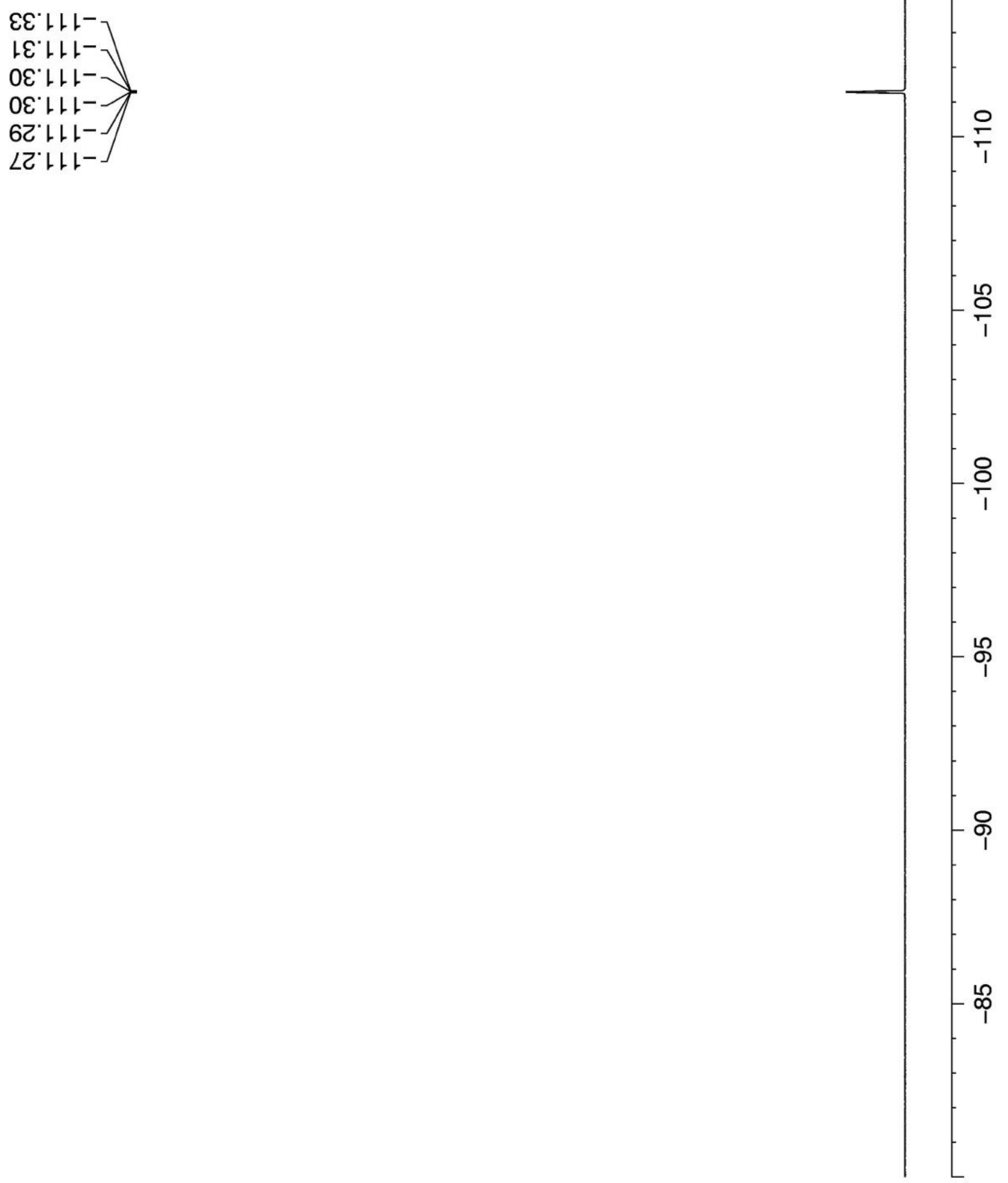

^{1}H NMR ($(CD_3)_2SO$, 25 ºC) of **1w**

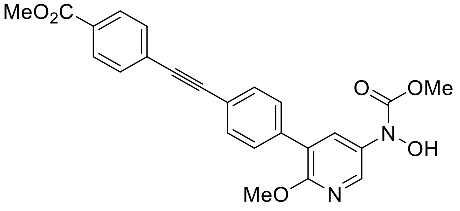

1w

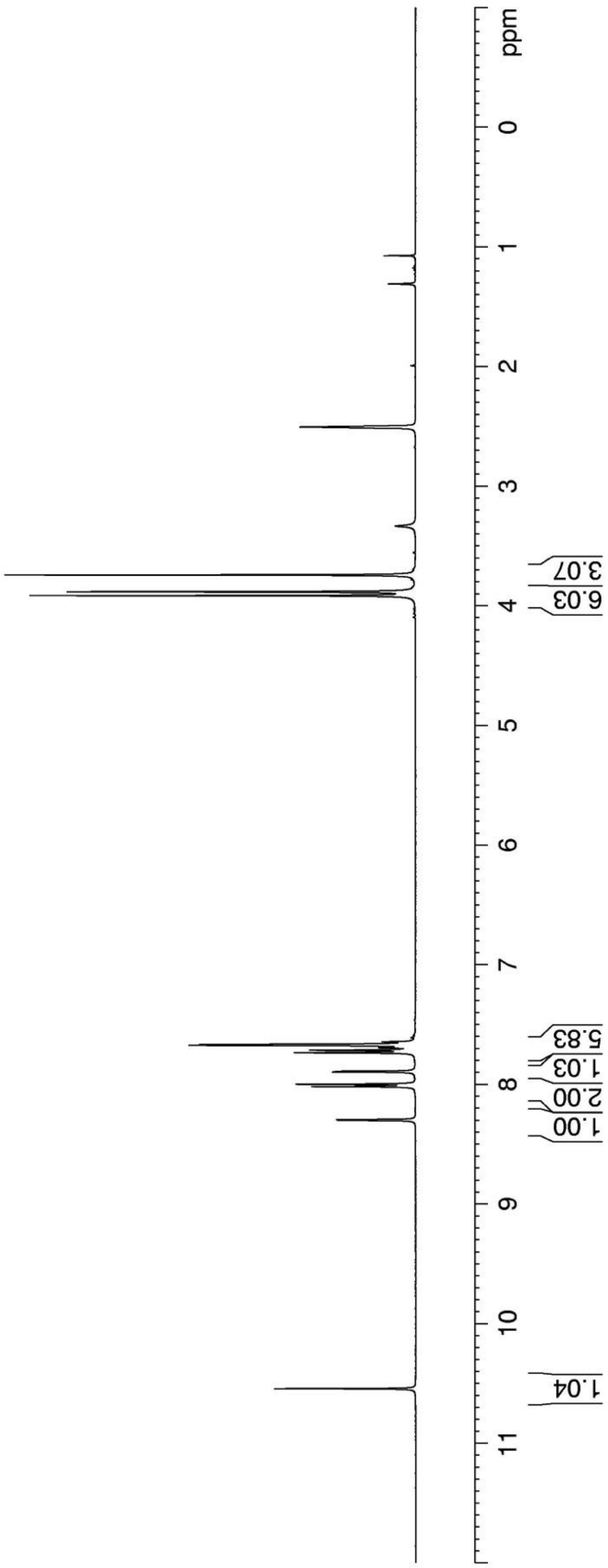

^{13}C NMR ((CD$_3$)$_2$SO, 25 ºC) of **1w**

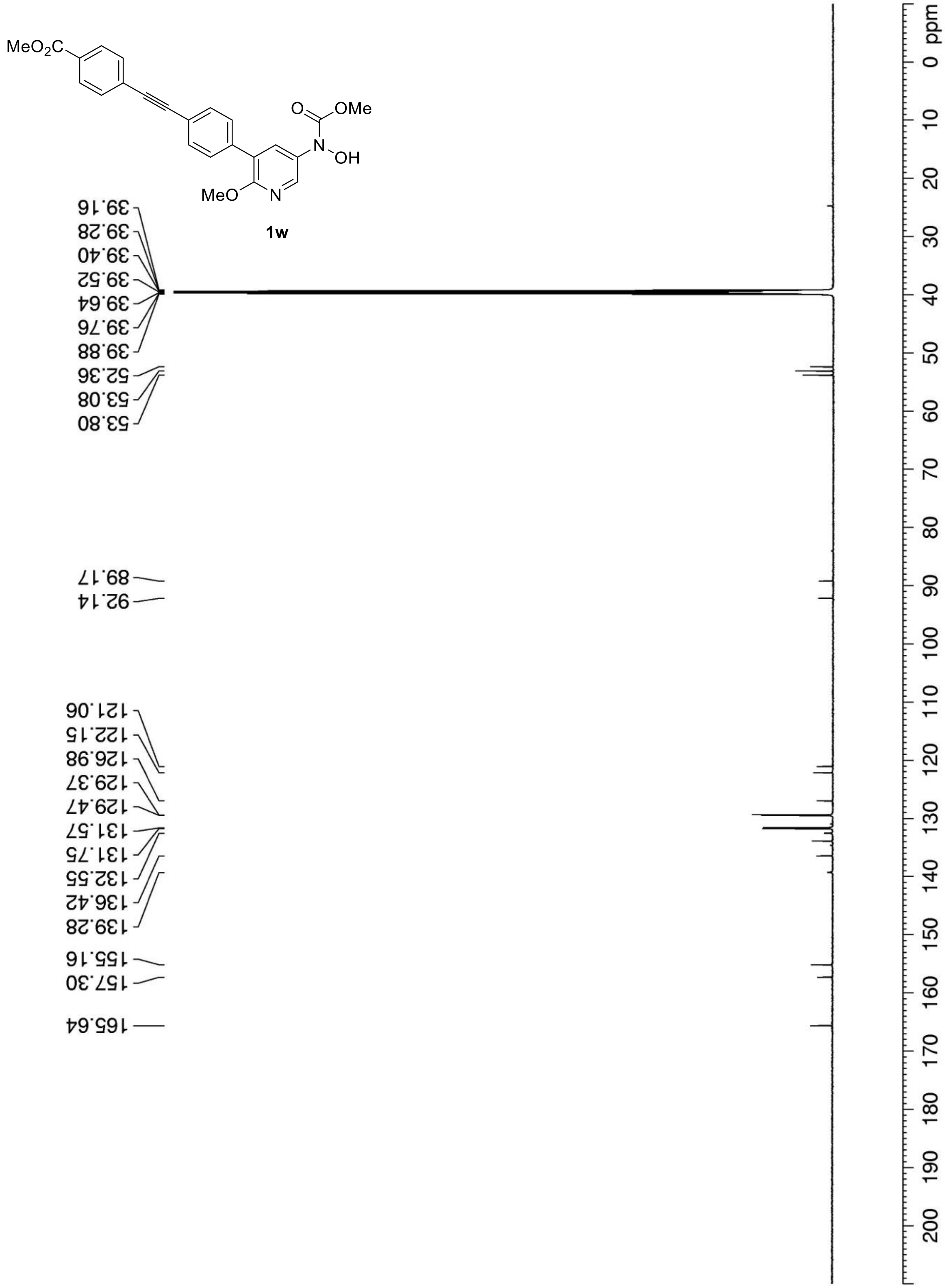

^{1}H NMR ($CDCl_3$, 25 ºC) of **S20**

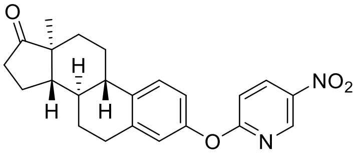

S20

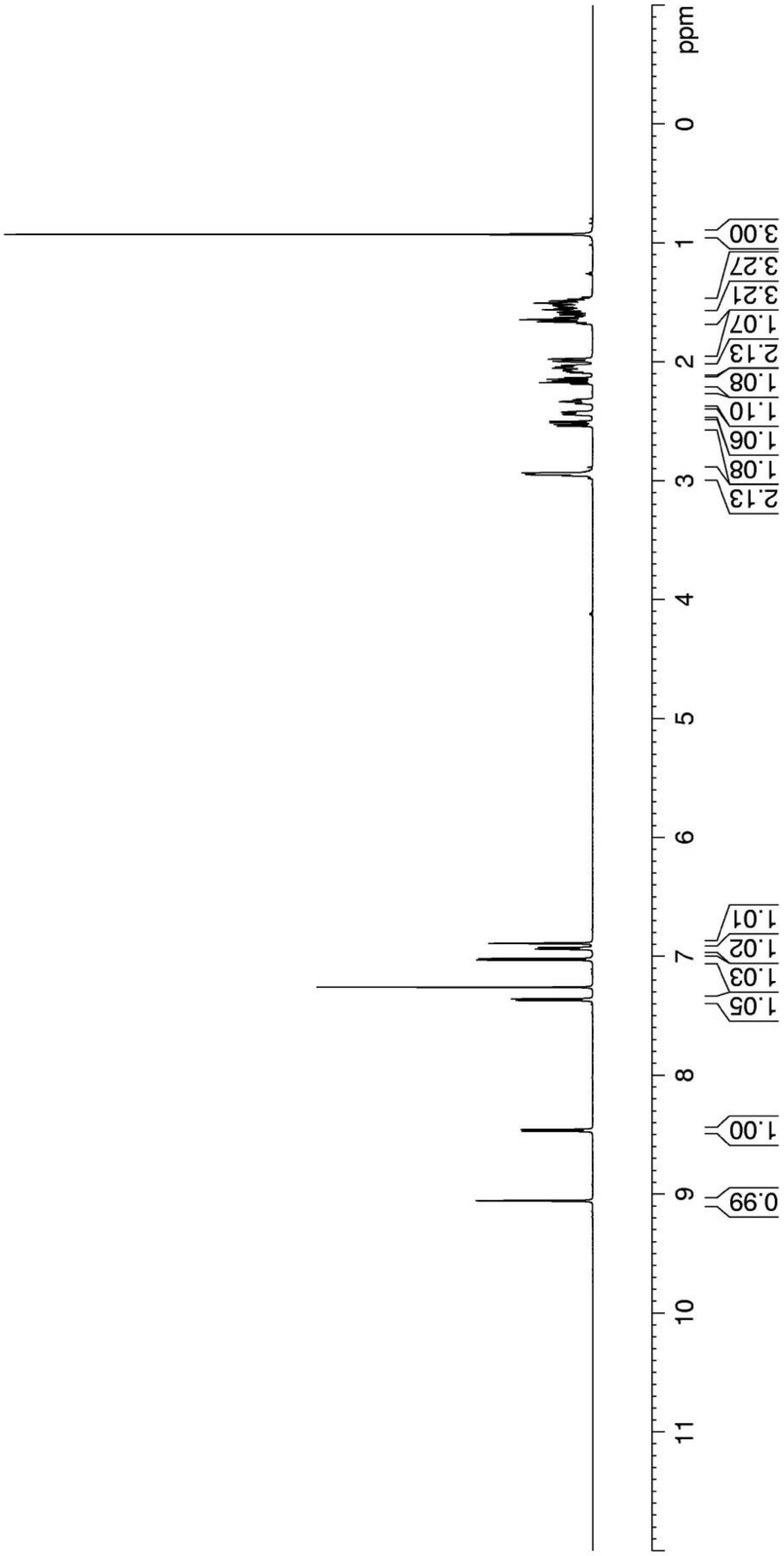

^{13}C NMR ($CDCl_3$, 25 ºC) of **S20**

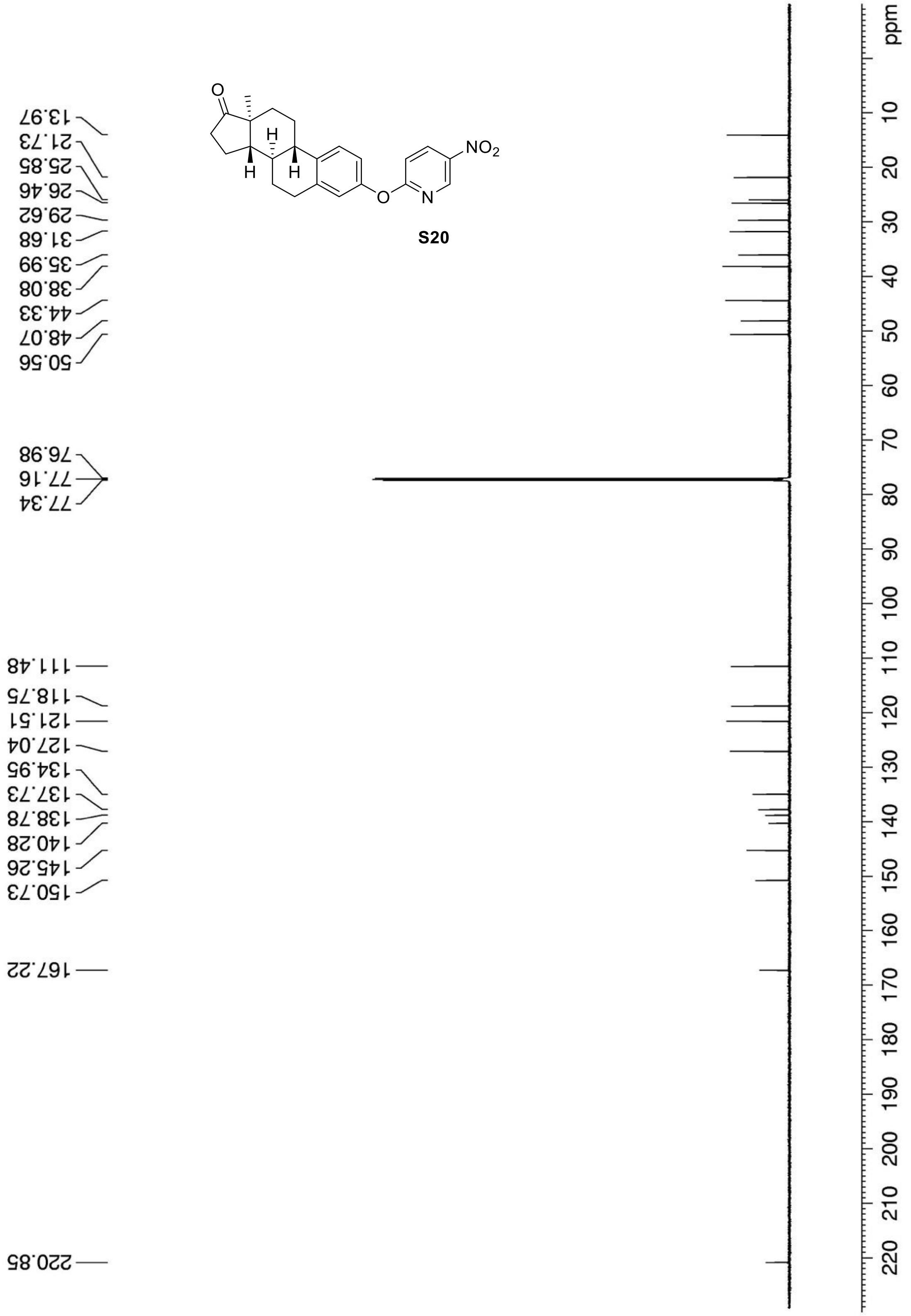

^{1}H NMR (($(CD_3)_2SO$, 25 ºC) of **1x**

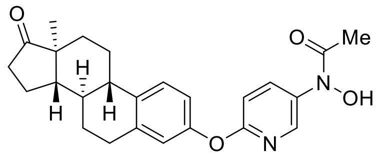

1x

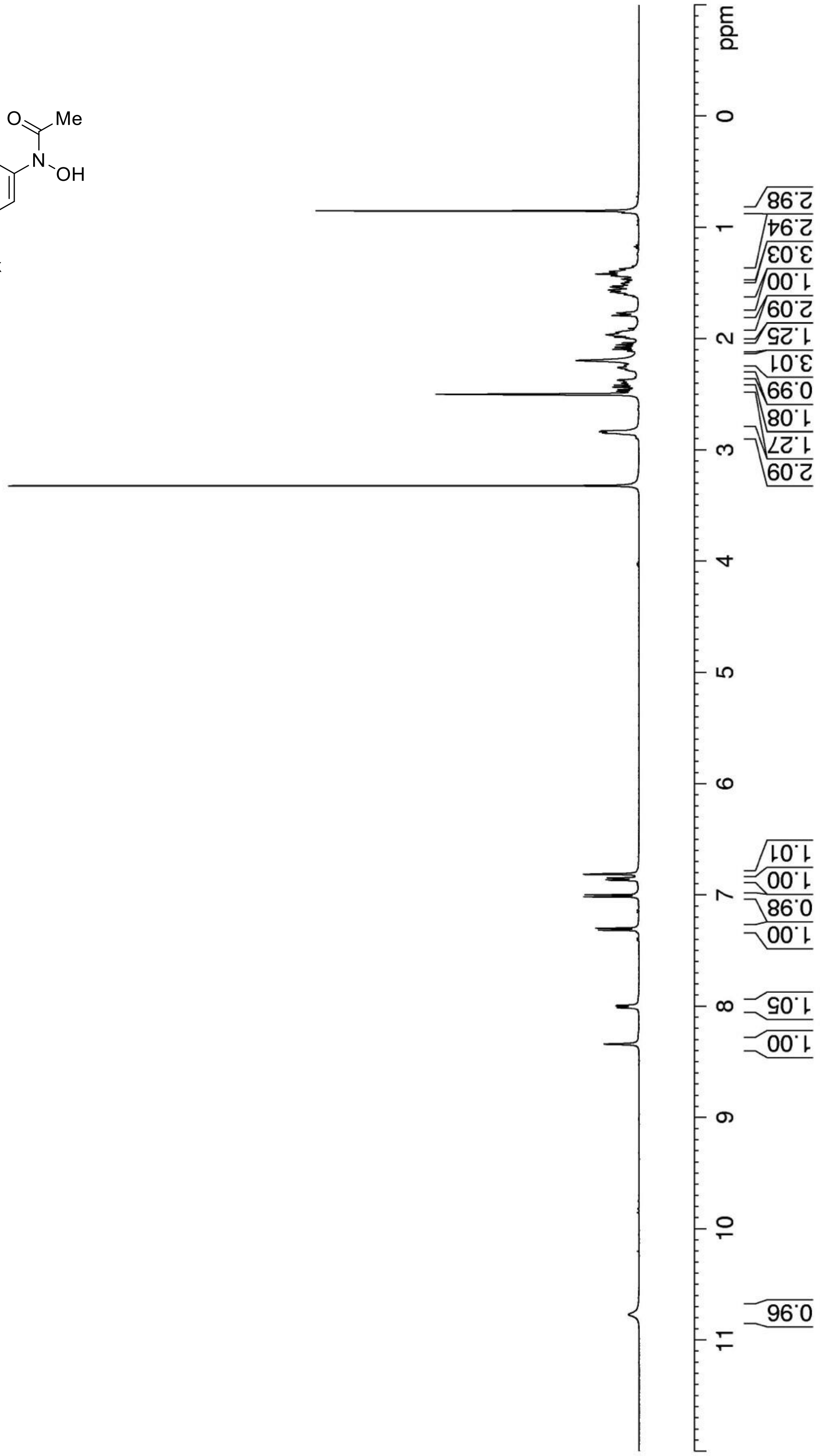

^{13}C NMR (($(CD_3)_2SO$, 25 ºC) of **1x**

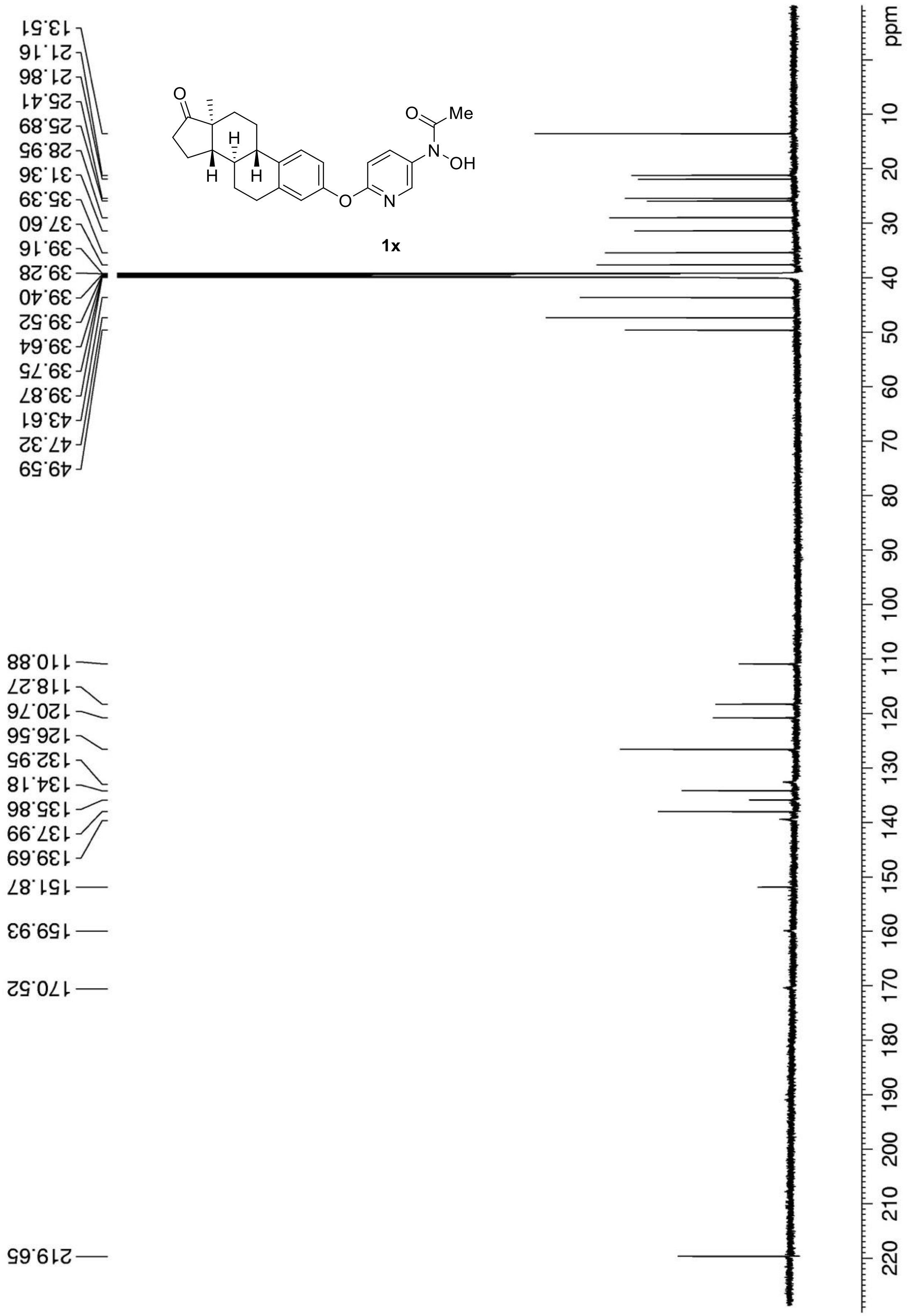

^{1}H NMR ($CDCl_3$, 25 ºC) of **S21**

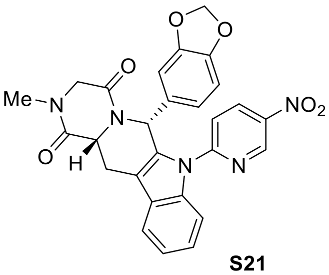

S21

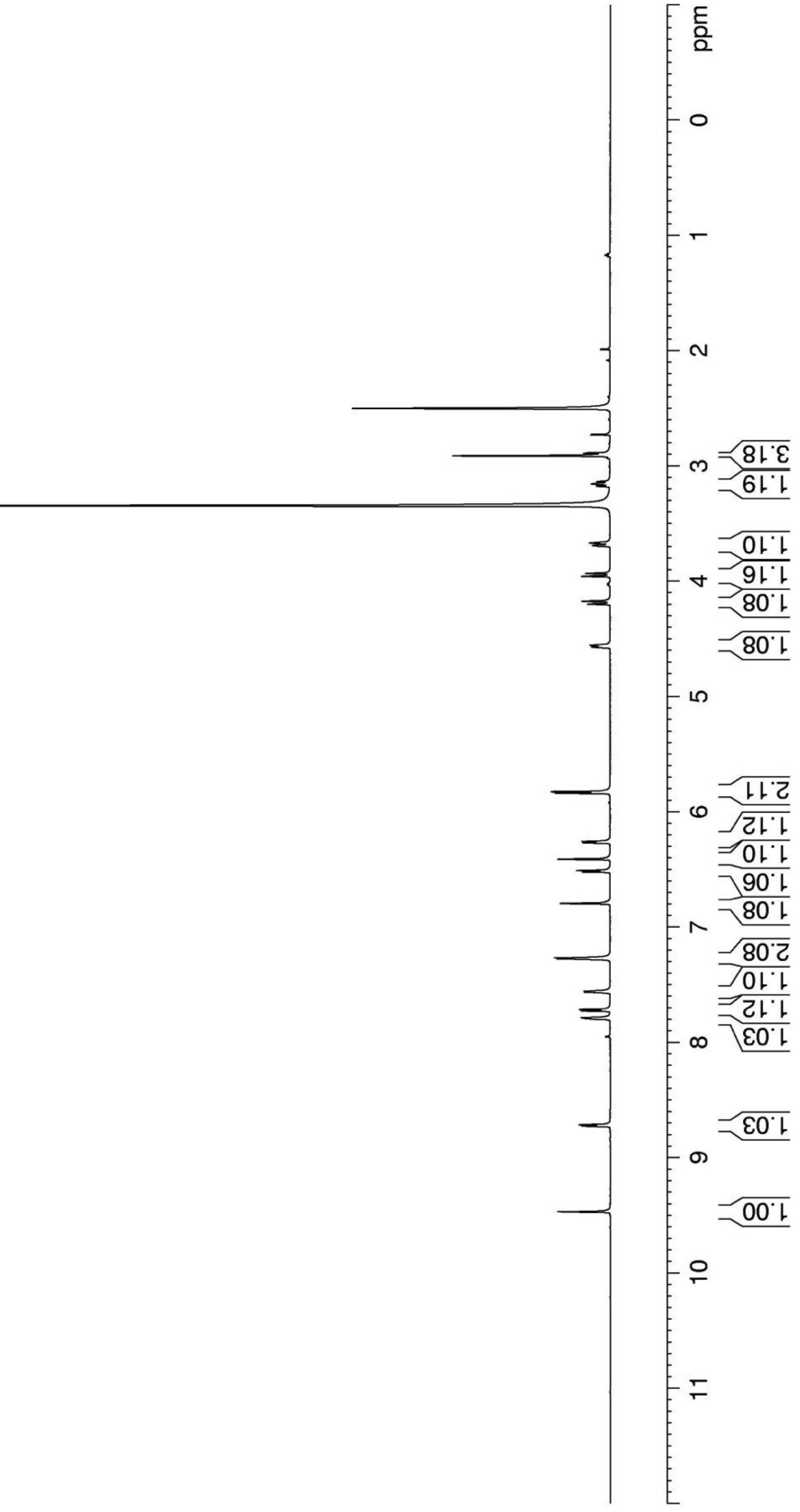

^{13}C NMR ($CDCl_3$, 25 ºC) of **S21**

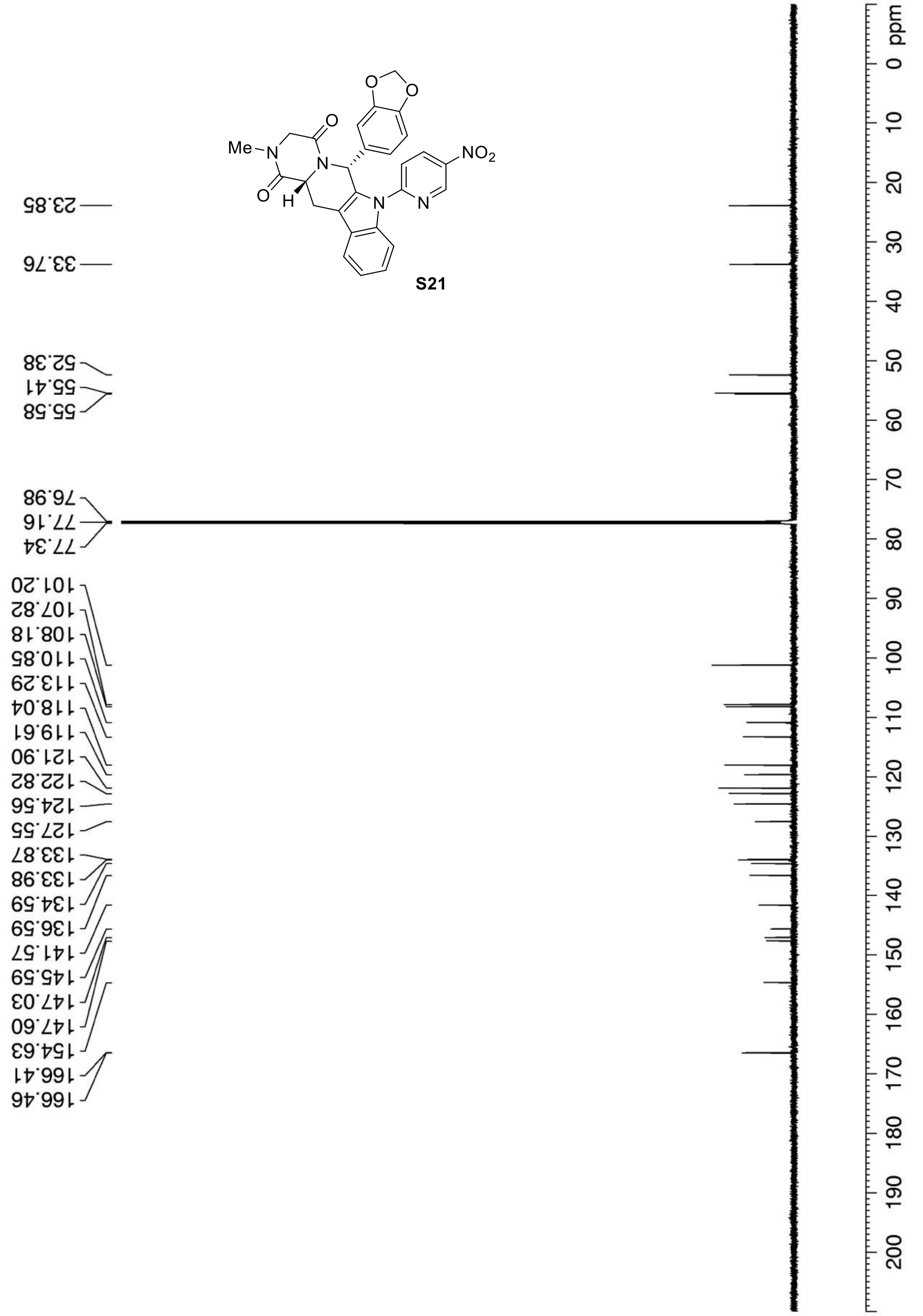

^{1}H NMR ($(CD_3)_2SO$, 25 ºC) of **1y**

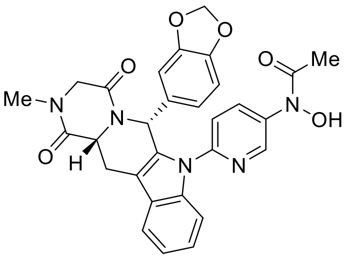

1y

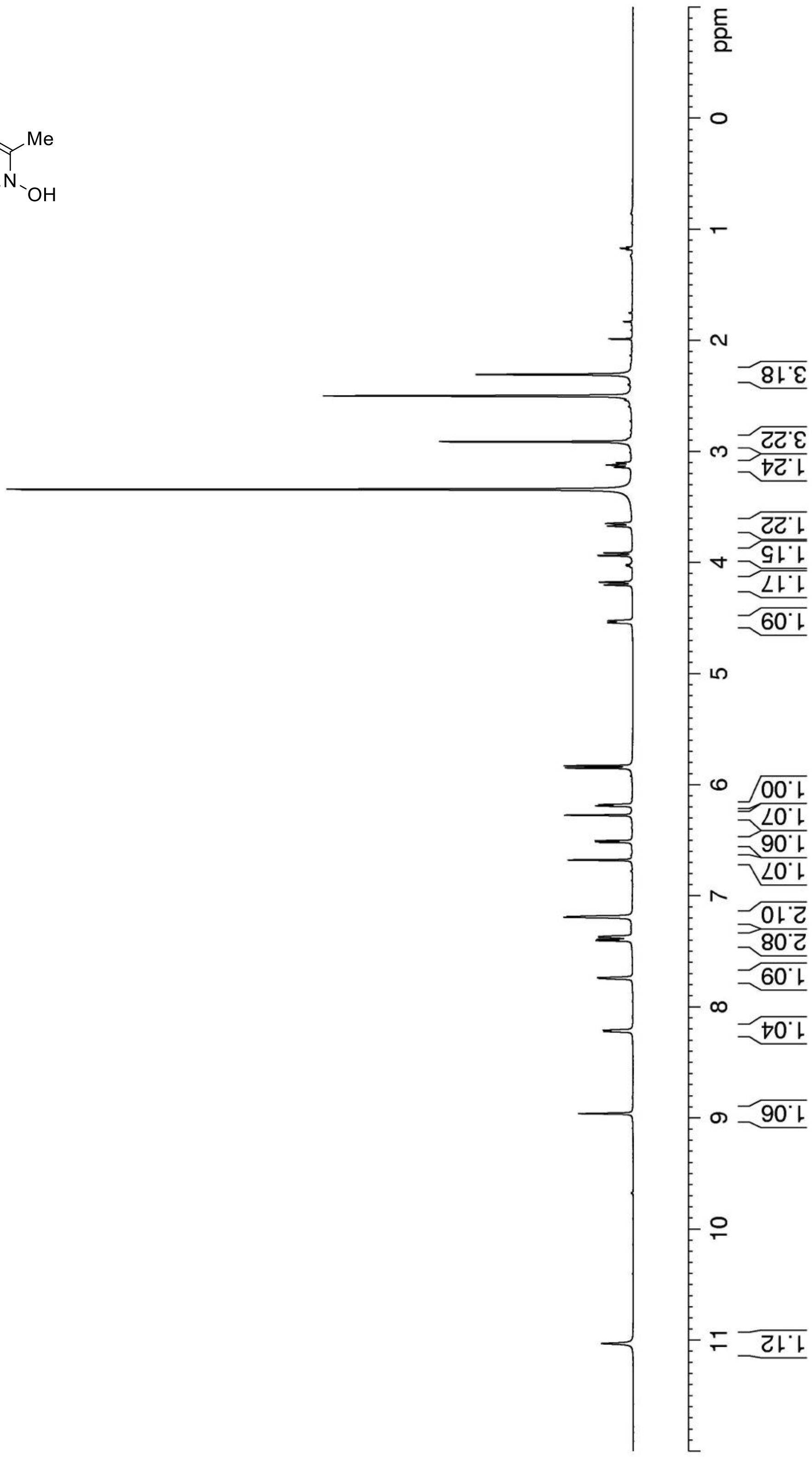

^{13}C NMR ($(CD_3)_2SO$, 25 ºC) of **1y**

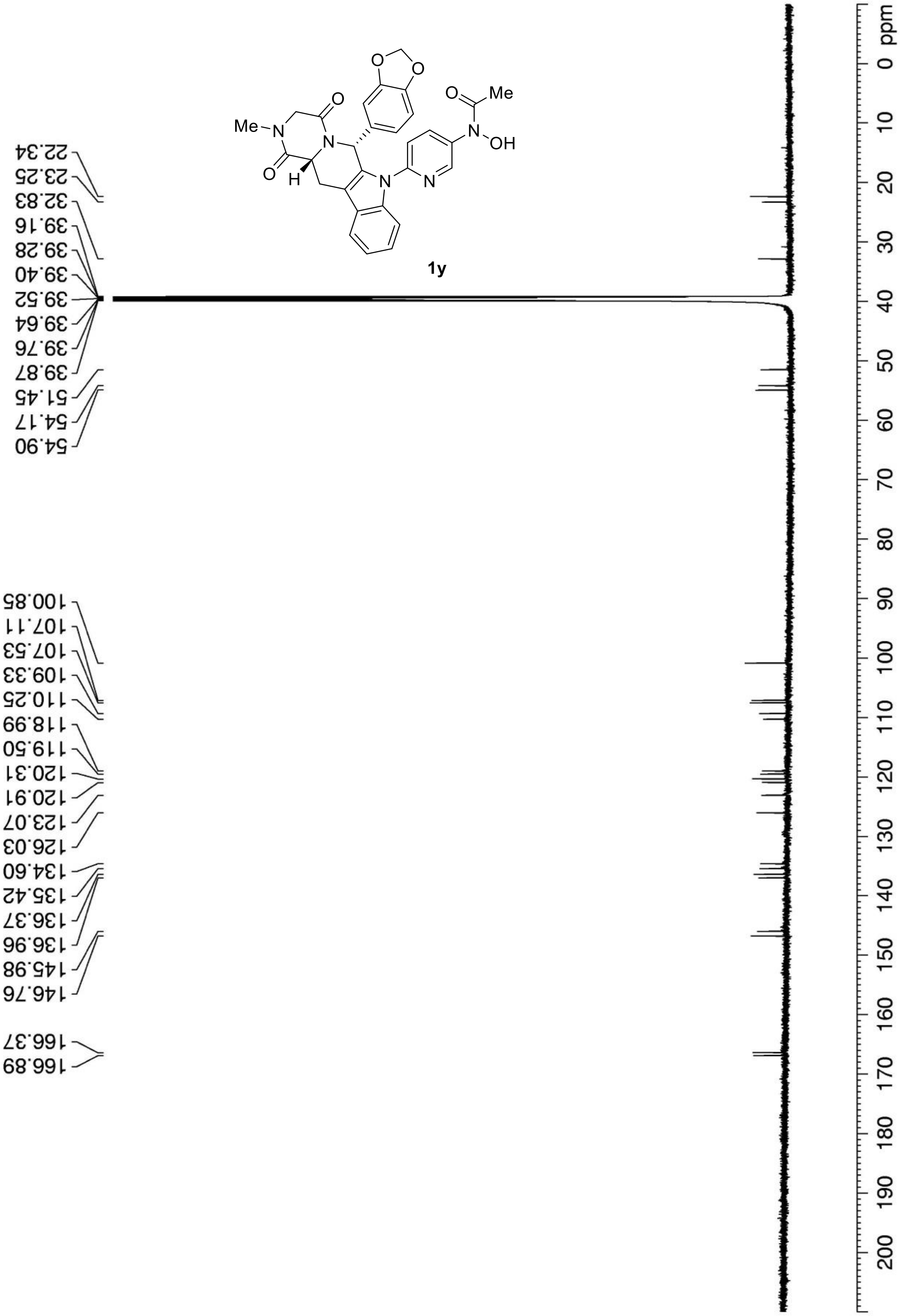

^{1}H NMR ($CDCl_3$, 25 ºC) of **S22**

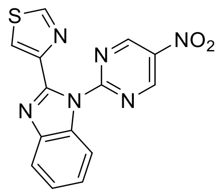

S22

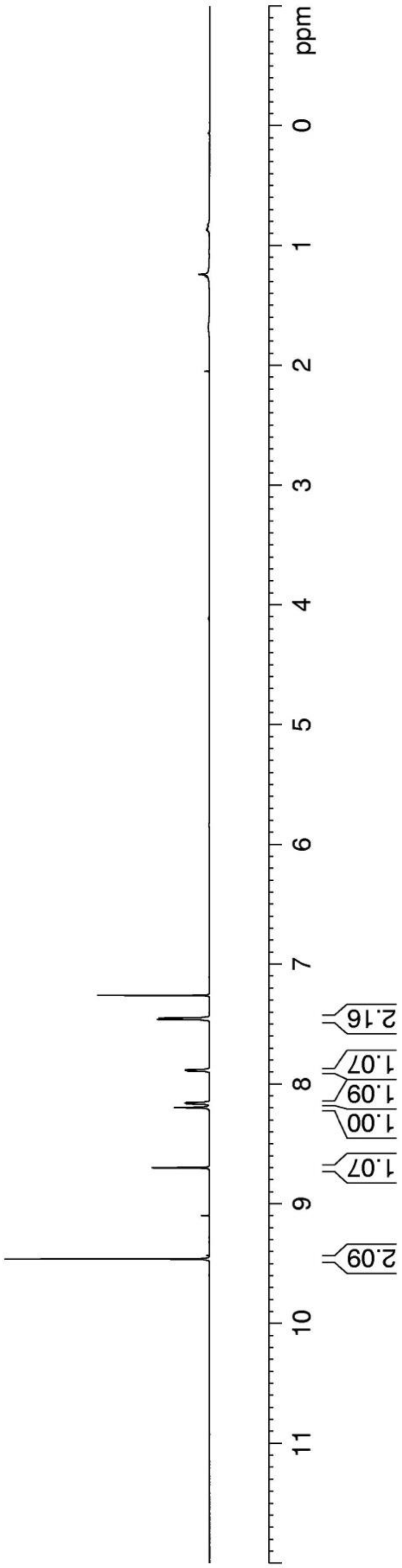

^{13}C NMR ($CDCl_3$, 25 ºC) of **S22**

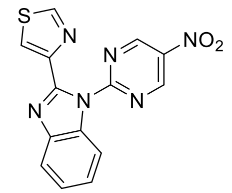

S22

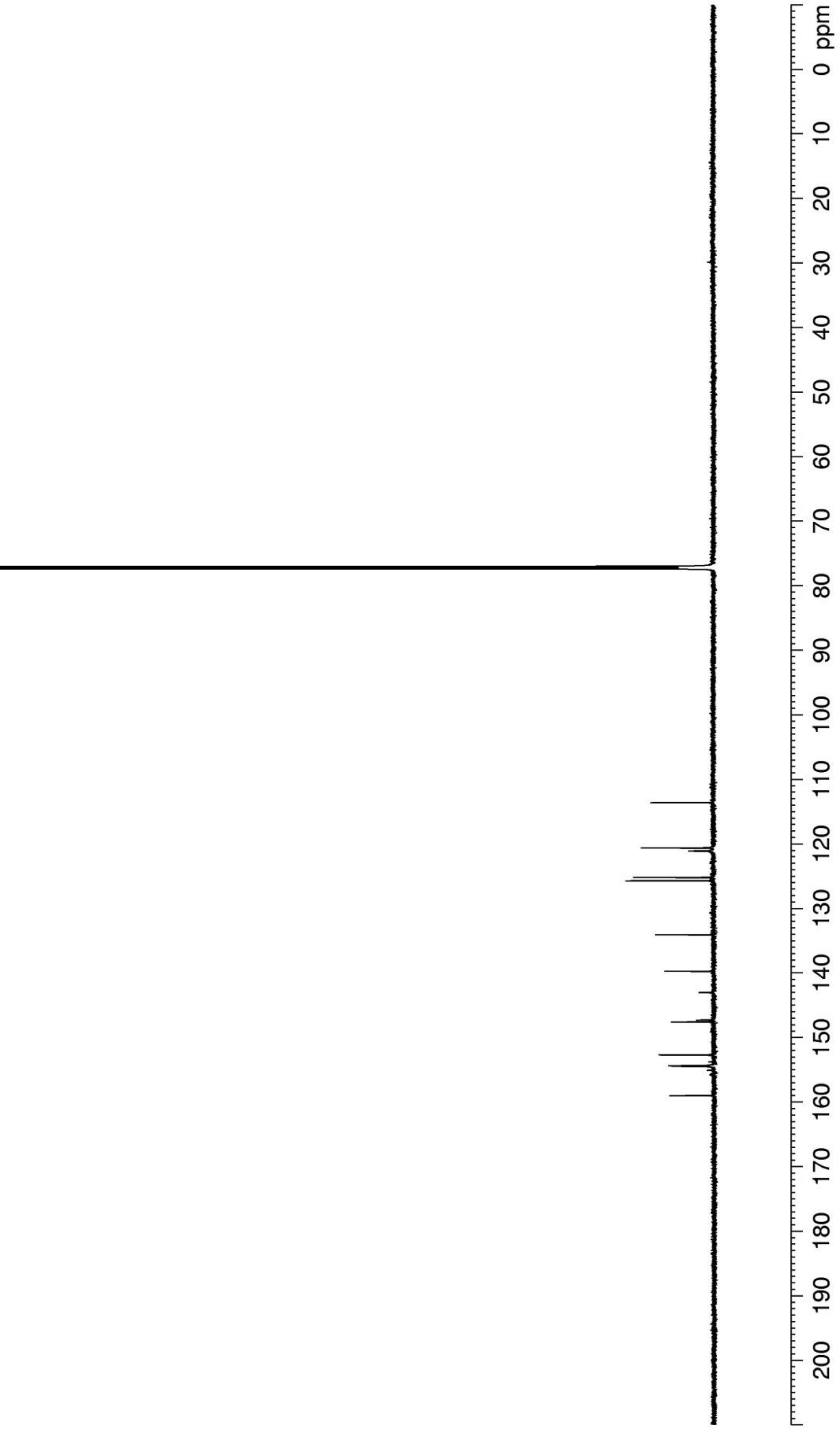

^{1}H NMR ($(CD_3)_2SO$, 25 ºC) of **3a**

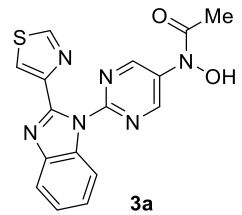

3a

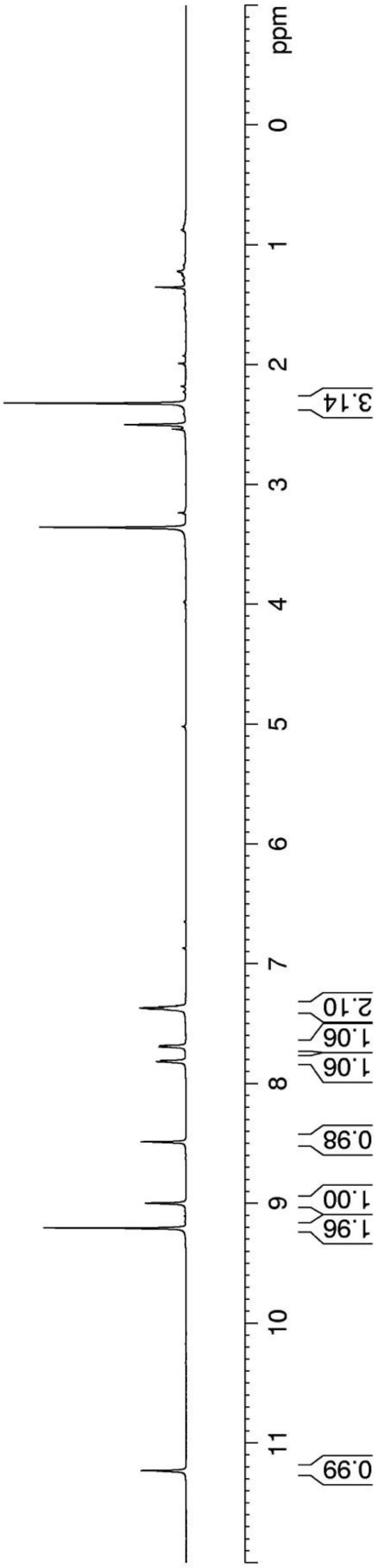

^{13}C NMR (($(CD_3)_2SO$, 25 ºC) of **3a**

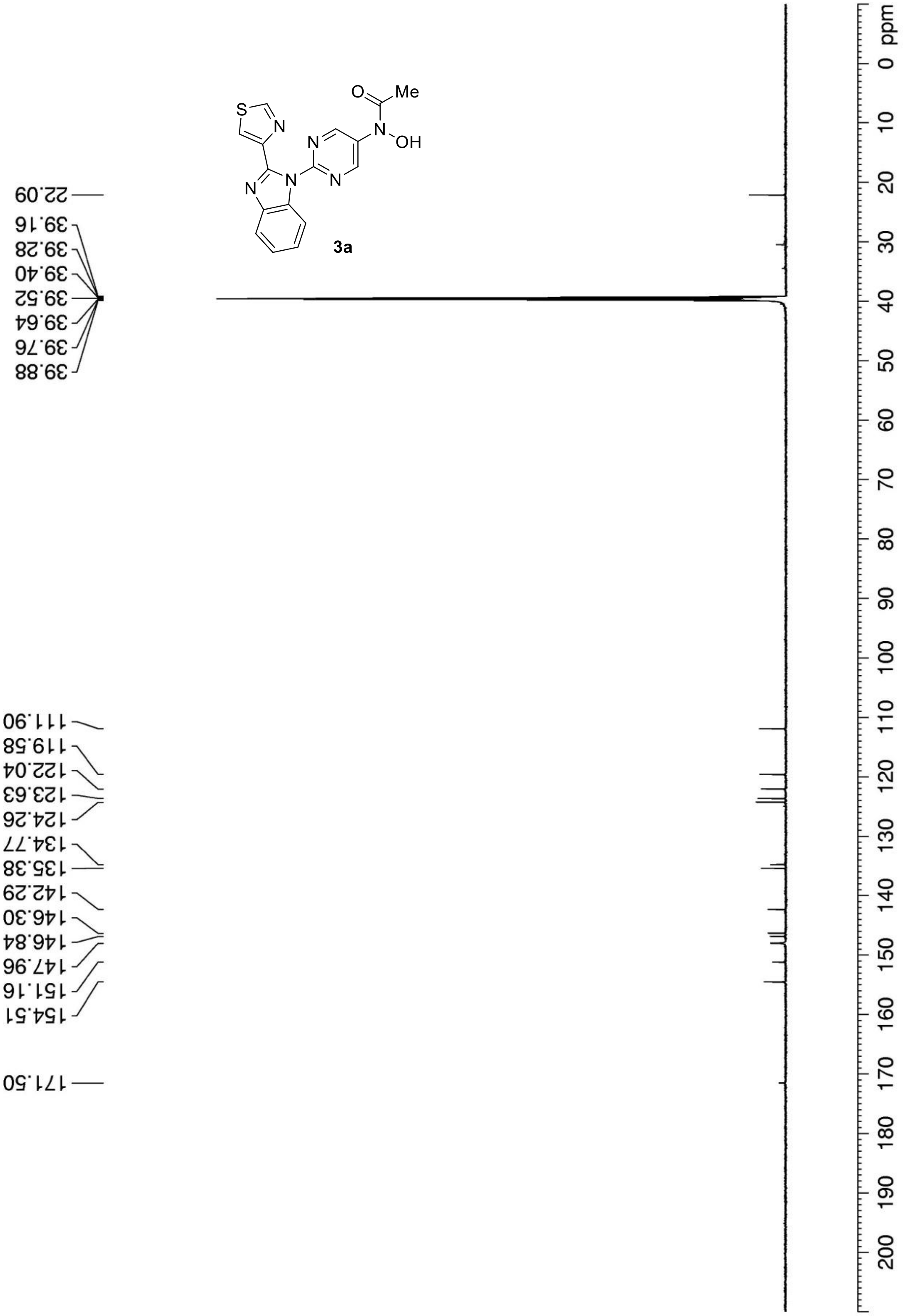

^{1}H NMR ($CDCl_3$, 25 ºC) of **S23**

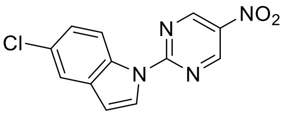

S23

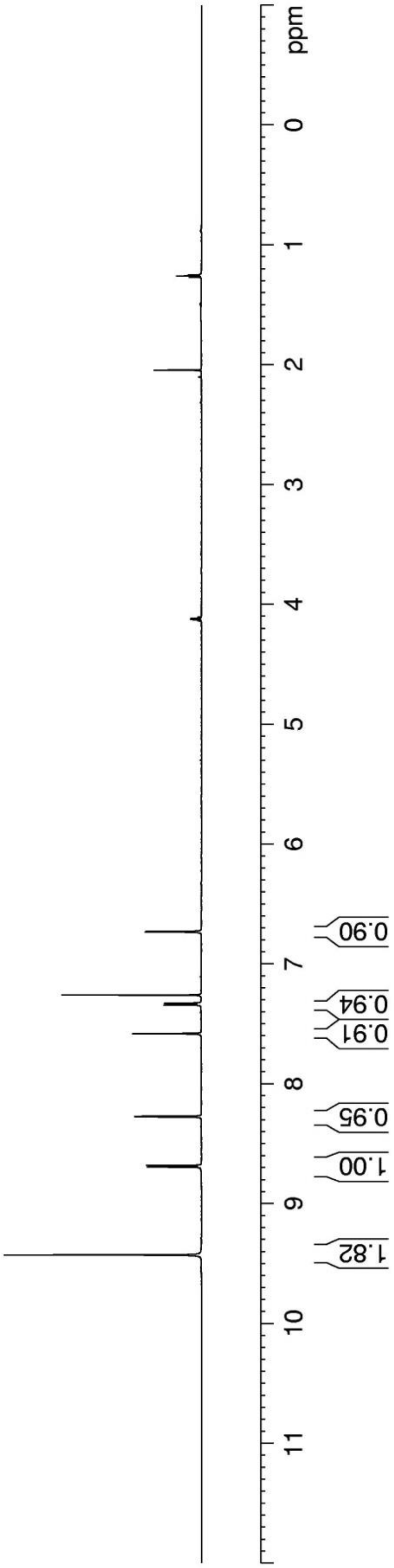

^{13}C NMR ($CDCl_3$, 25 ºC) of **S23**

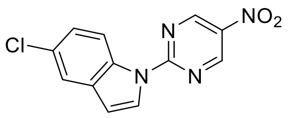

S23

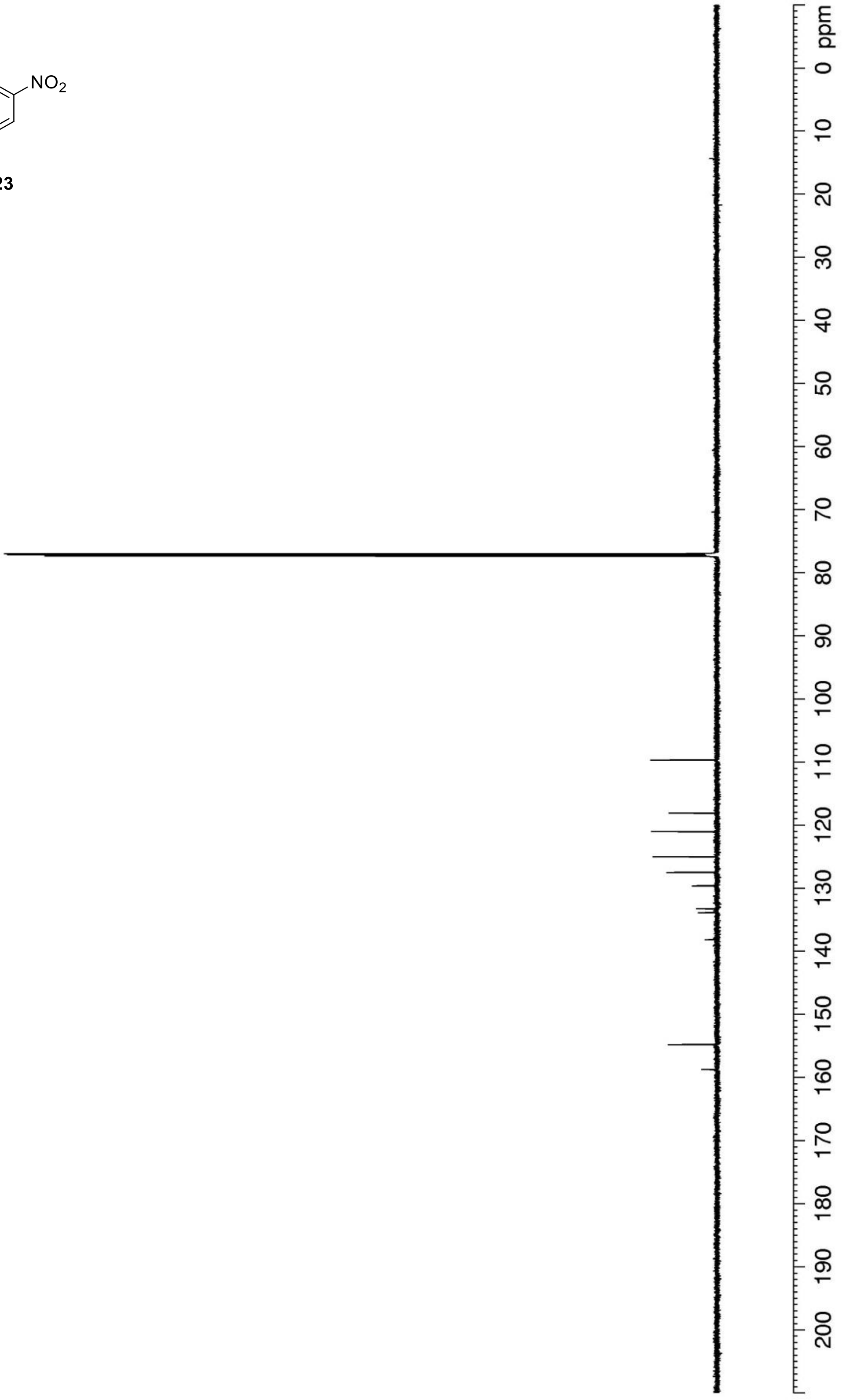

^{1}H NMR (($(CD_3)_2SO$, 25 ºC) of **3b**

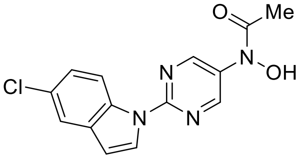

3b

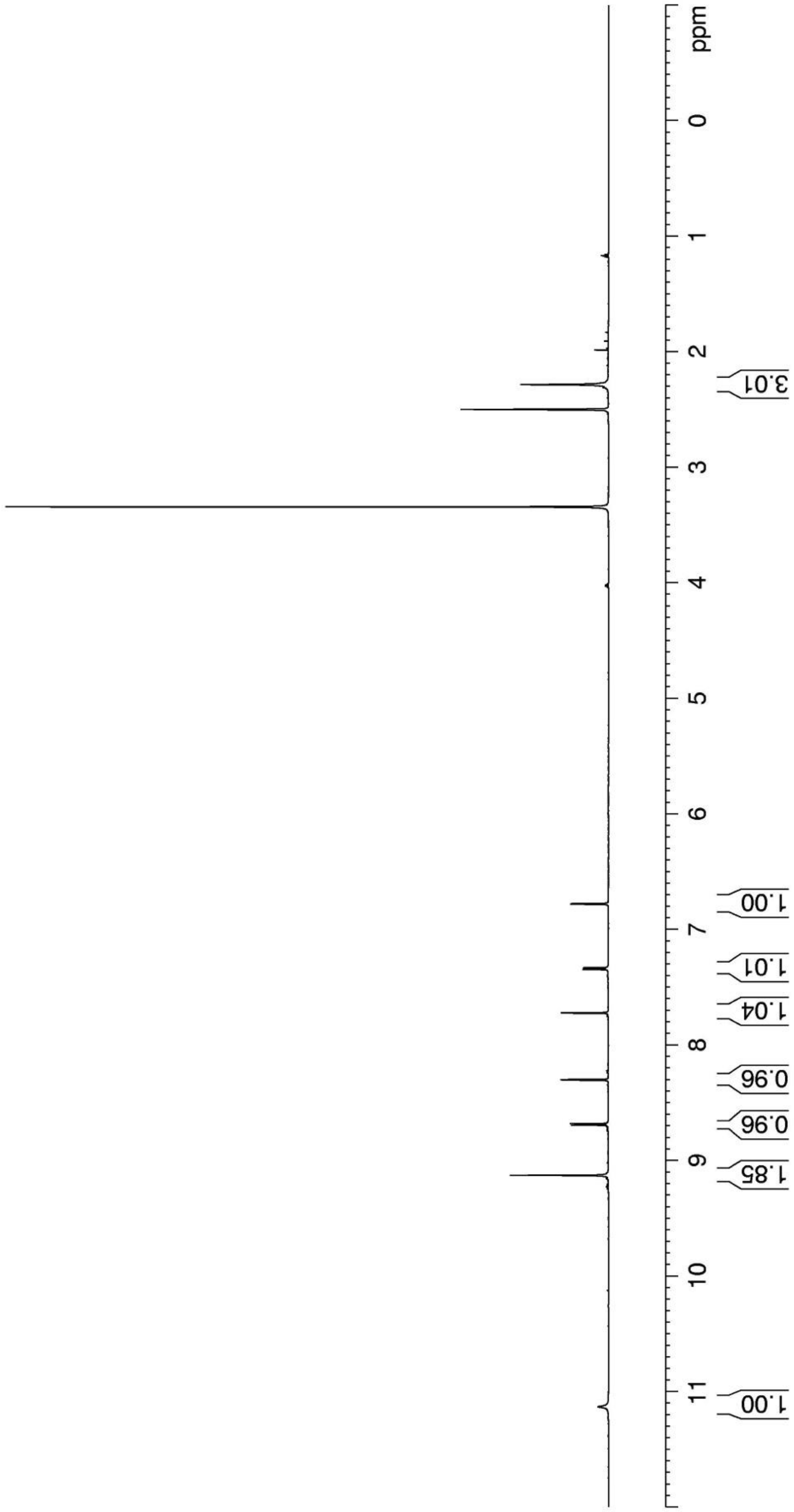

^{13}C NMR ((CD_3)$_2$SO, 25 ºC) of **3b**

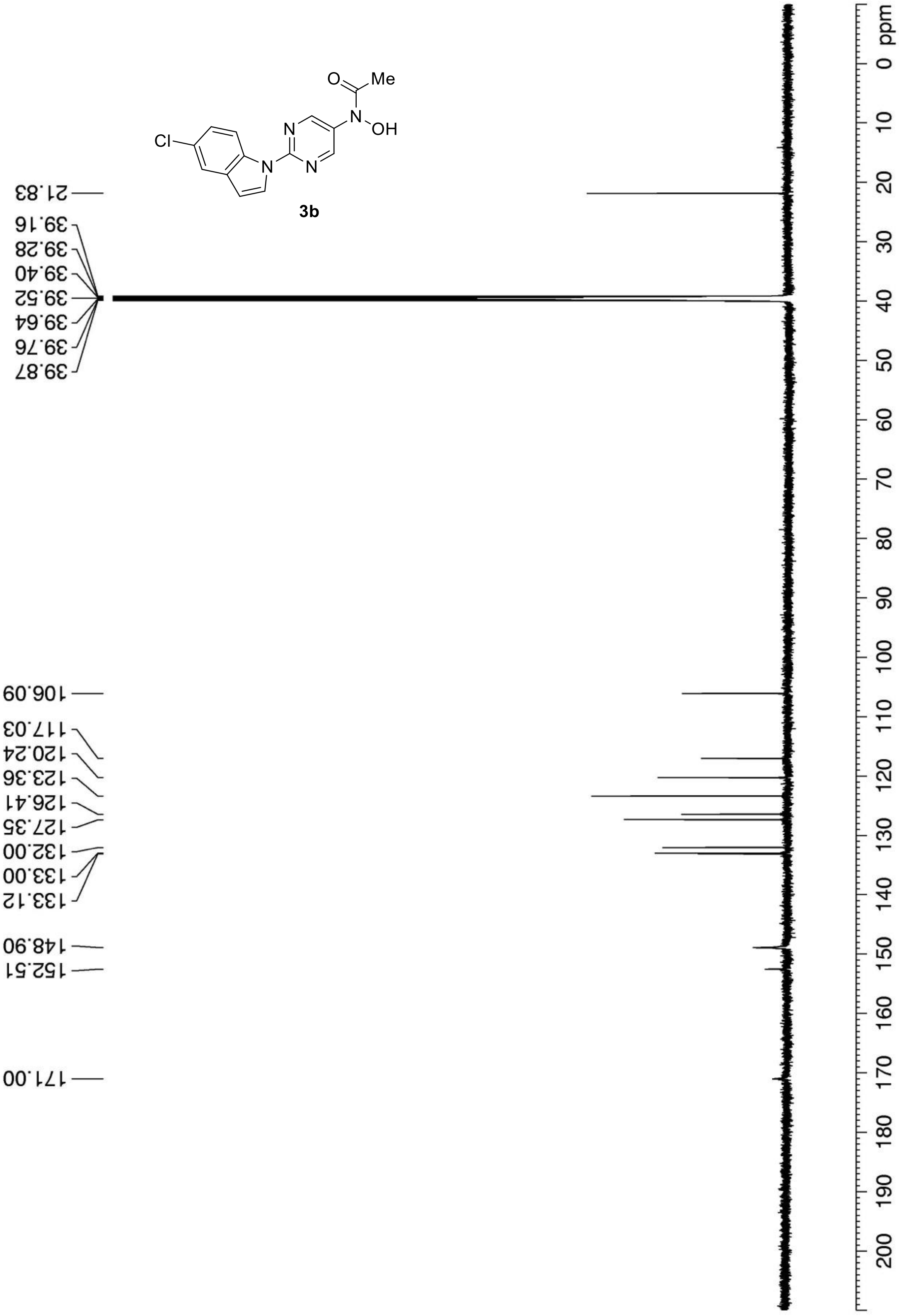

^{1}H NMR ((CD$_3$)$_2$SO, 25 ºC) of **3c**

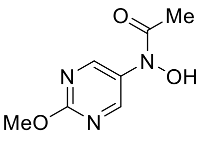

3c

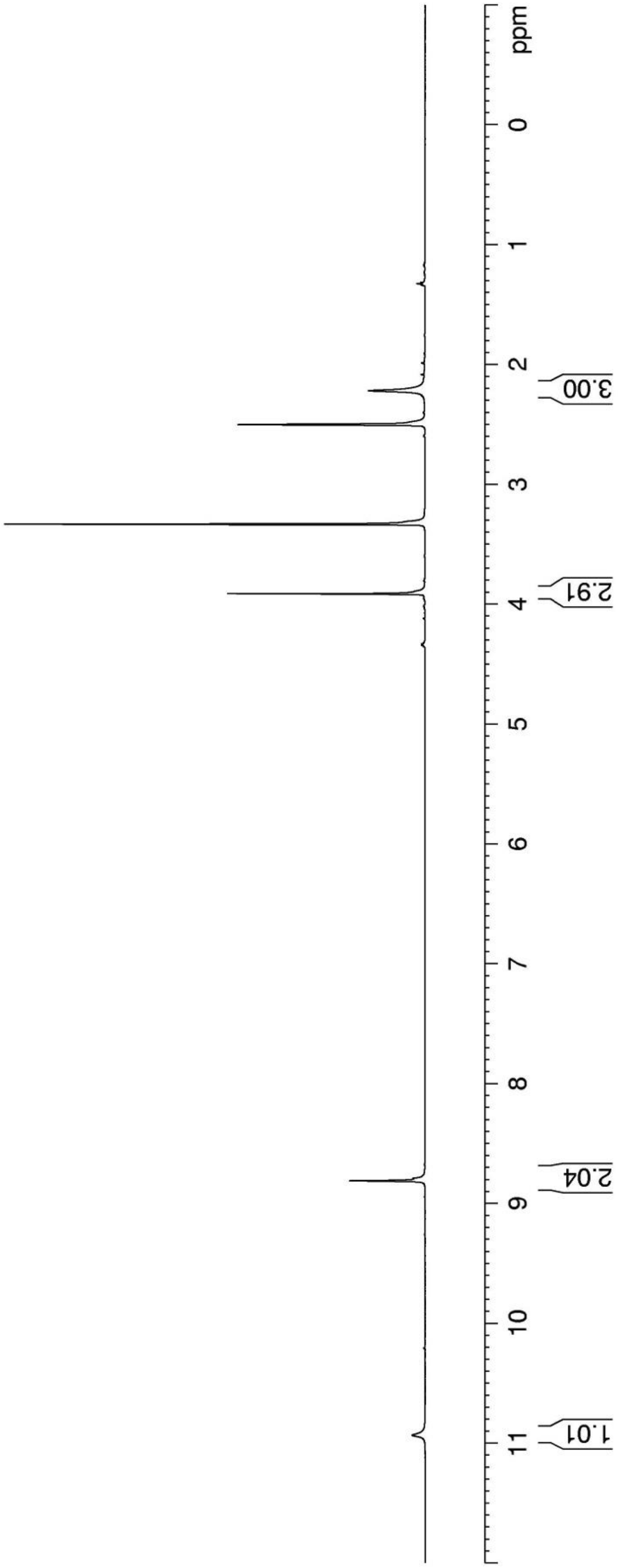

^{13}C NMR ((CD$_3$)$_2$SO, 25 ºC) of **3c**

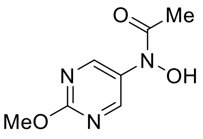

3c

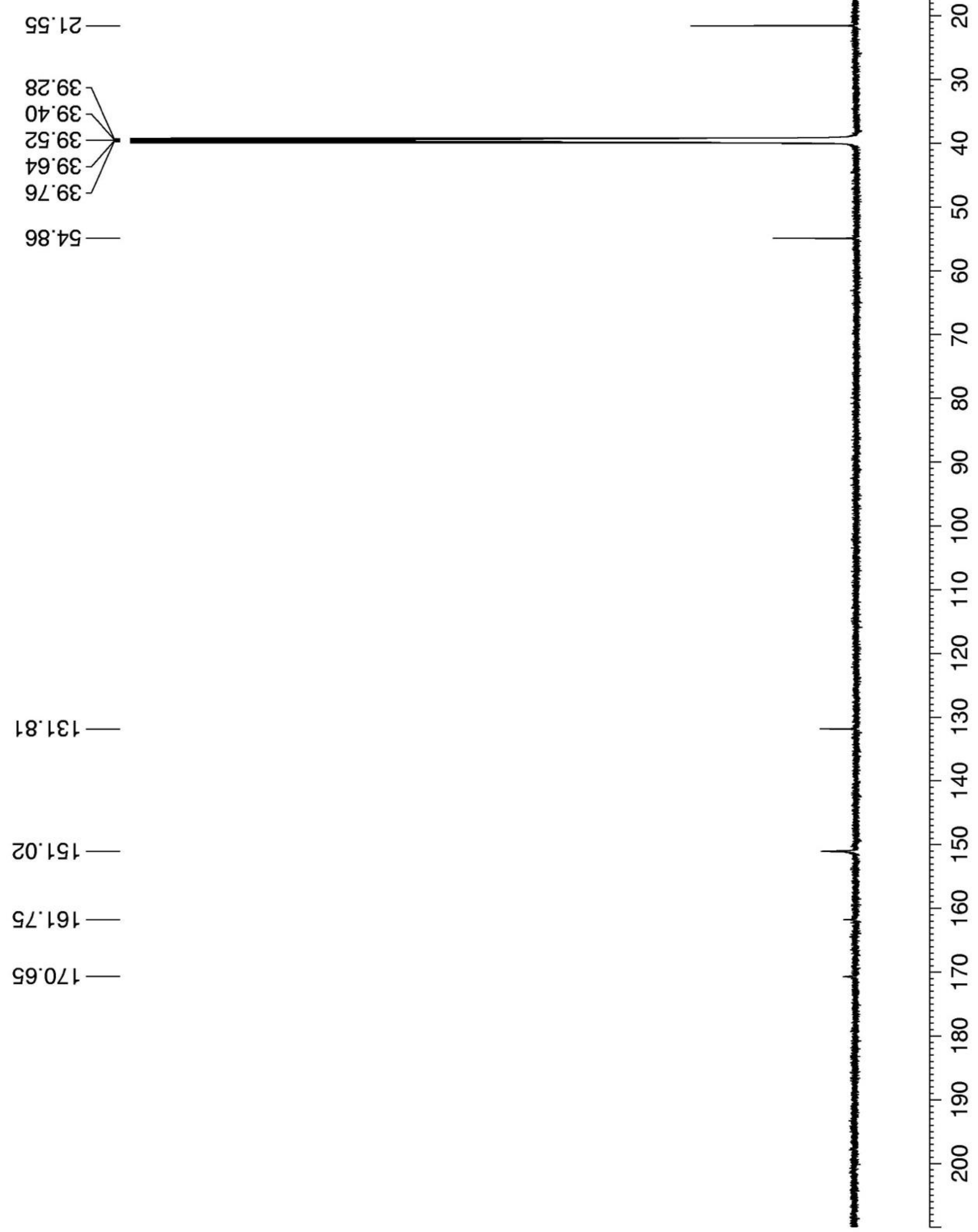

^{1}H NMR ($CDCl_3$, 25 ºC) of **S24**

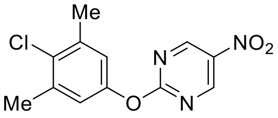

S24

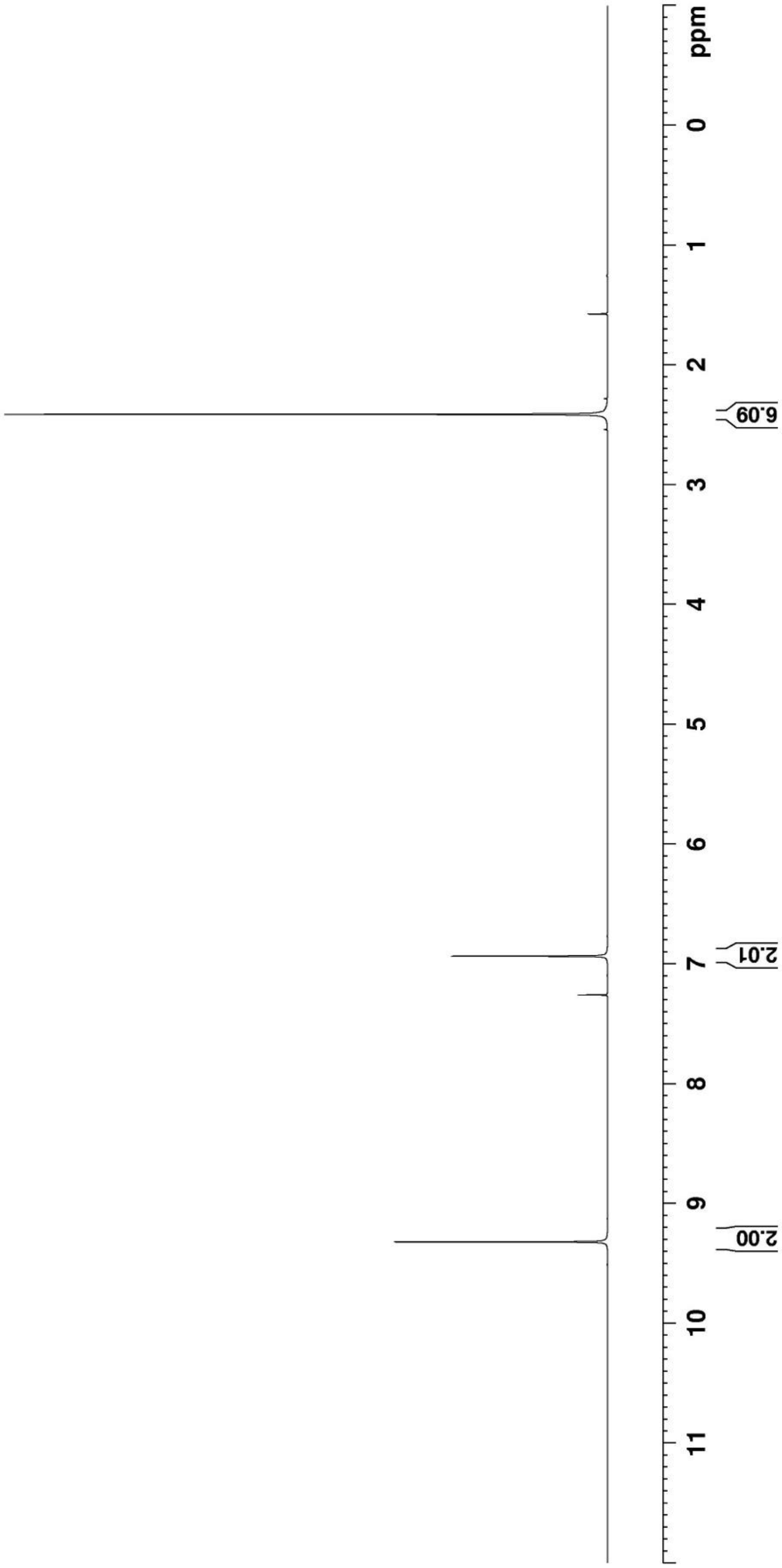

^{13}C NMR ($CDCl_3$, 25 ºC) of **S24**

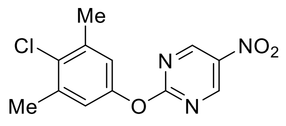

S24

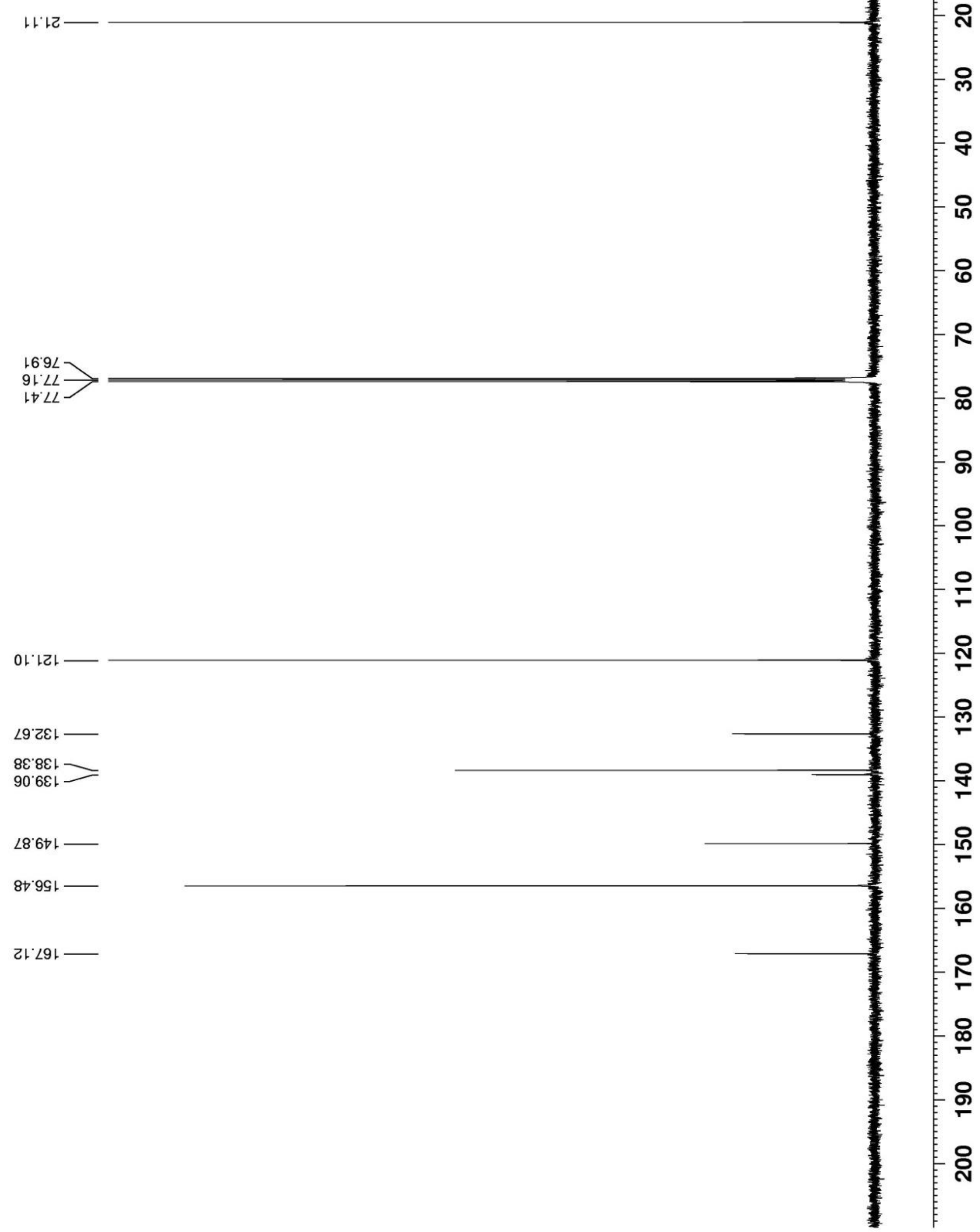

^{1}H NMR ($(CD_3)_2SO$, 25 ºC) of **3d**

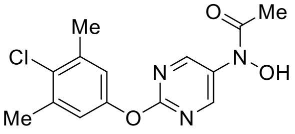

3d

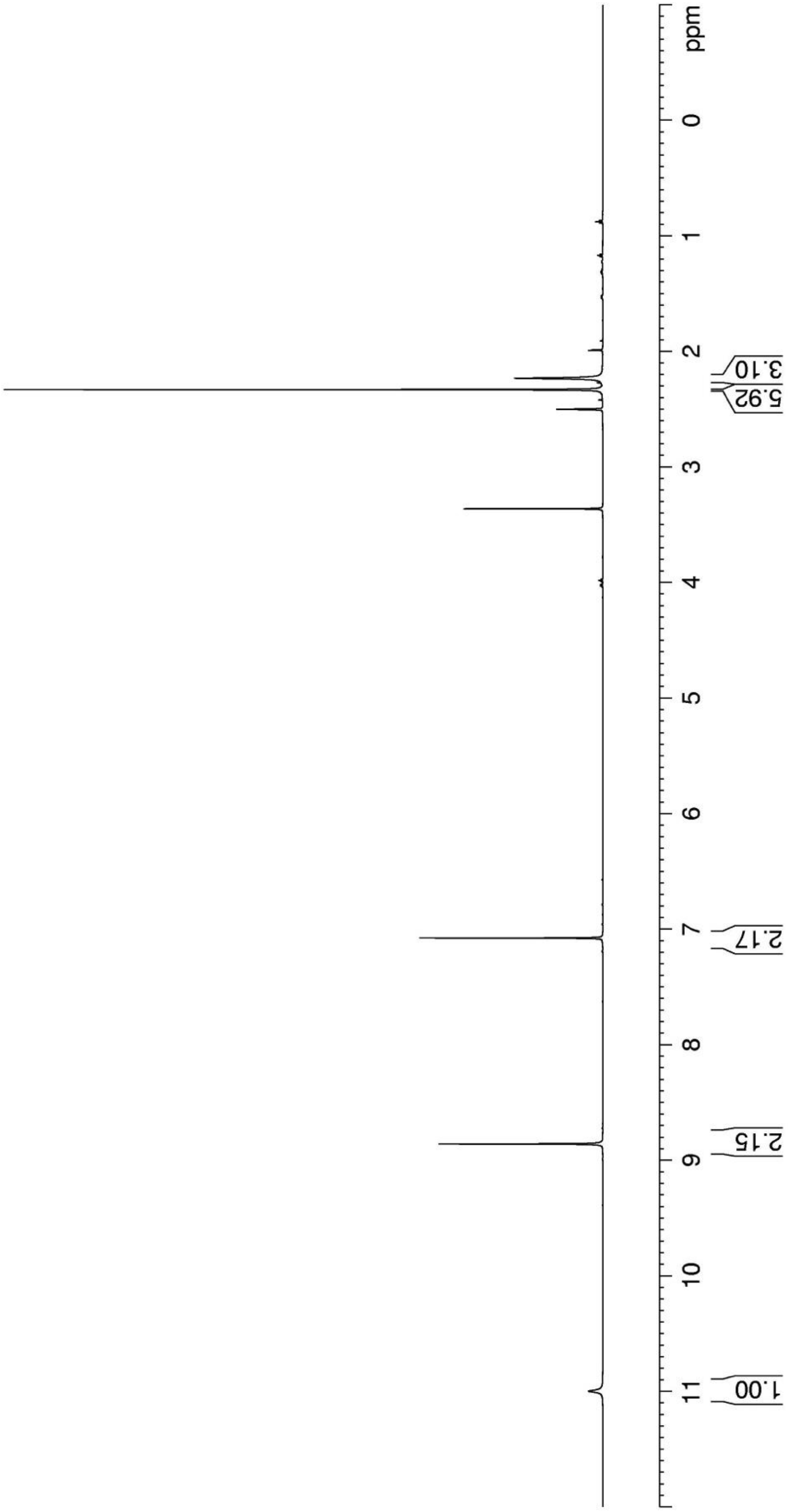

^{13}C NMR (($(CD_3)_2SO$, 25 ºC) of **3d**

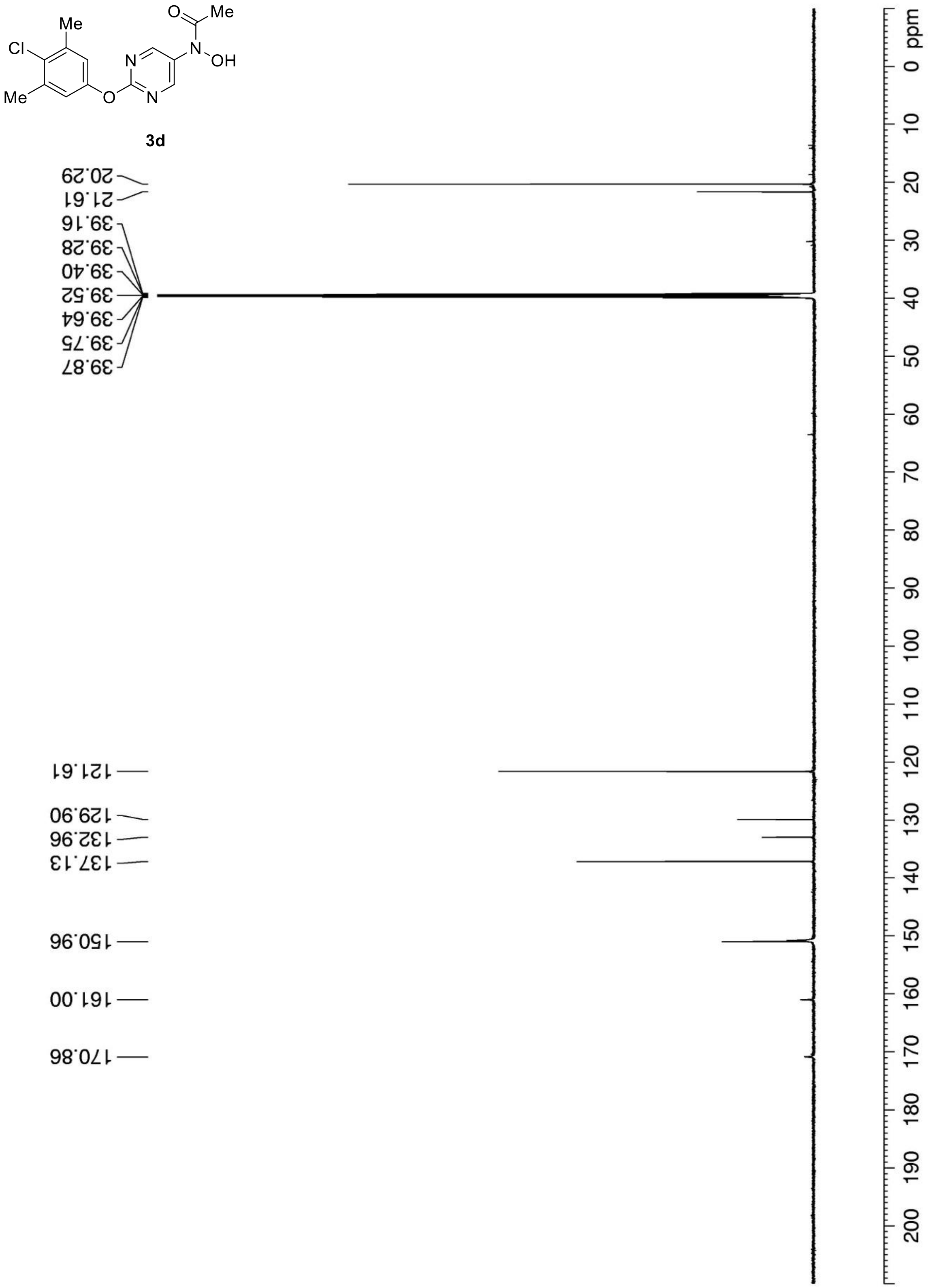

3d

^{1}H NMR ($CDCl_3$, 25 ºC) of **S25**

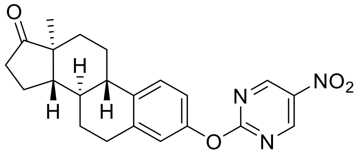

S25

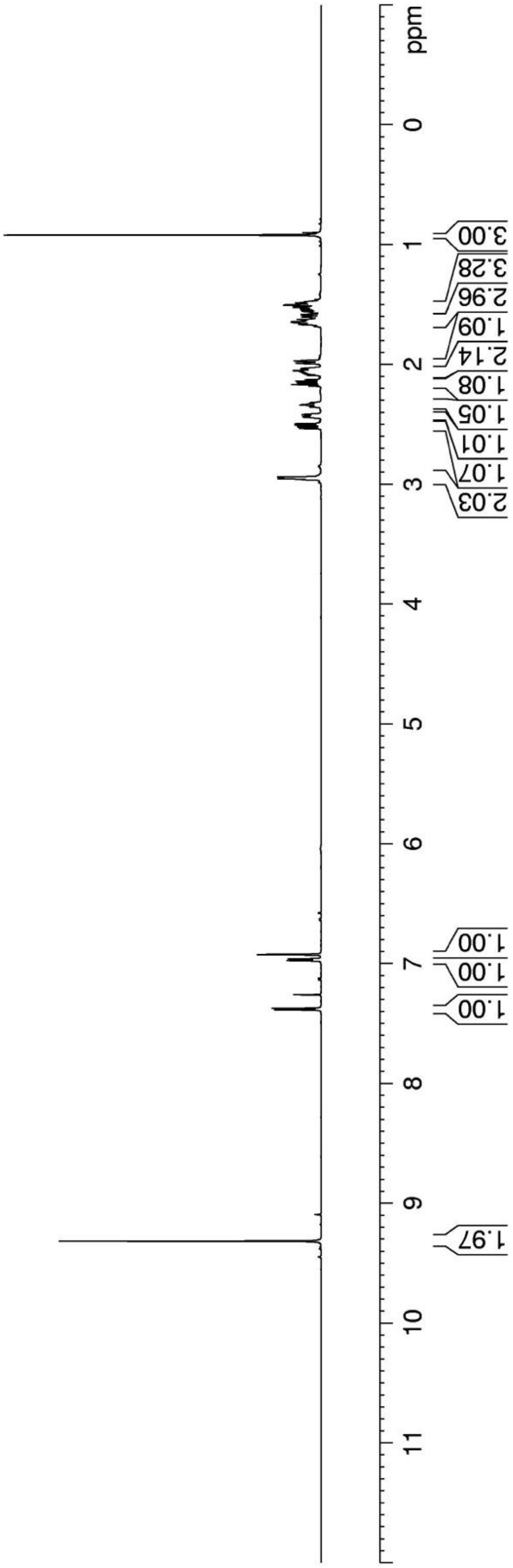

^{13}C NMR ($CDCl_3$, 25 ºC) of **S25**

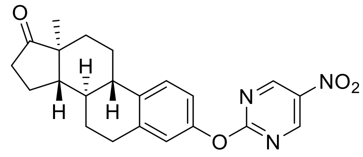

S25

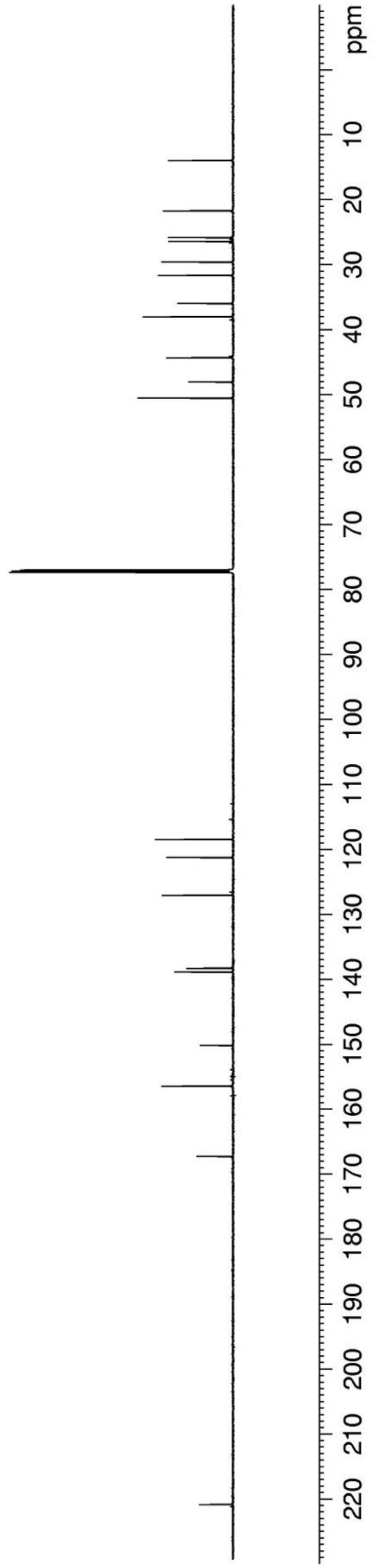

^{1}H NMR ($(CD_3)_2SO$, 25 ºC) of **3e**

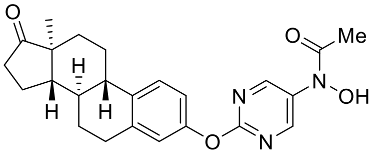

3e

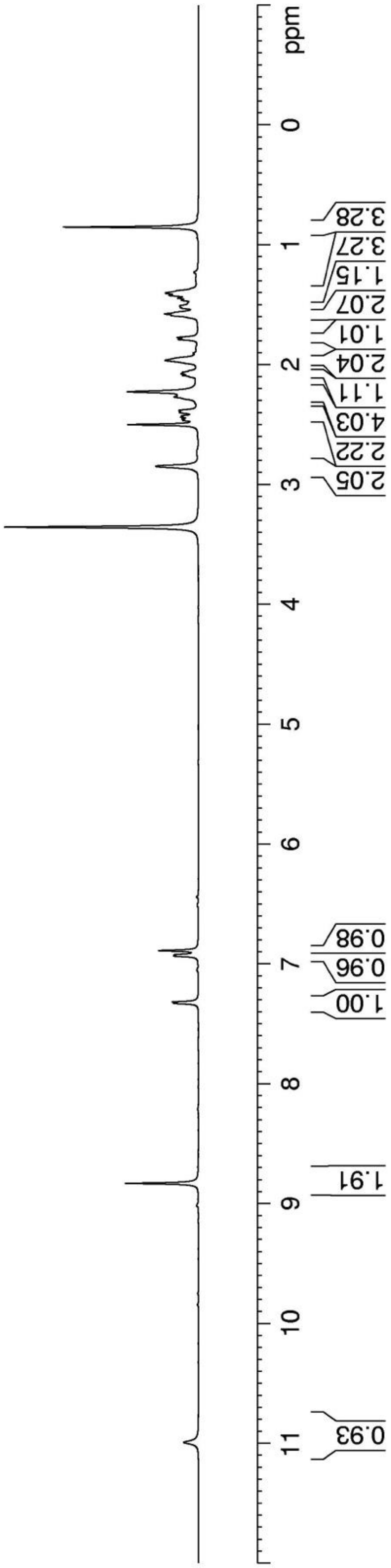

^{13}C NMR ((CD$_3$)$_2$SO, 25 ºC) of **3e**

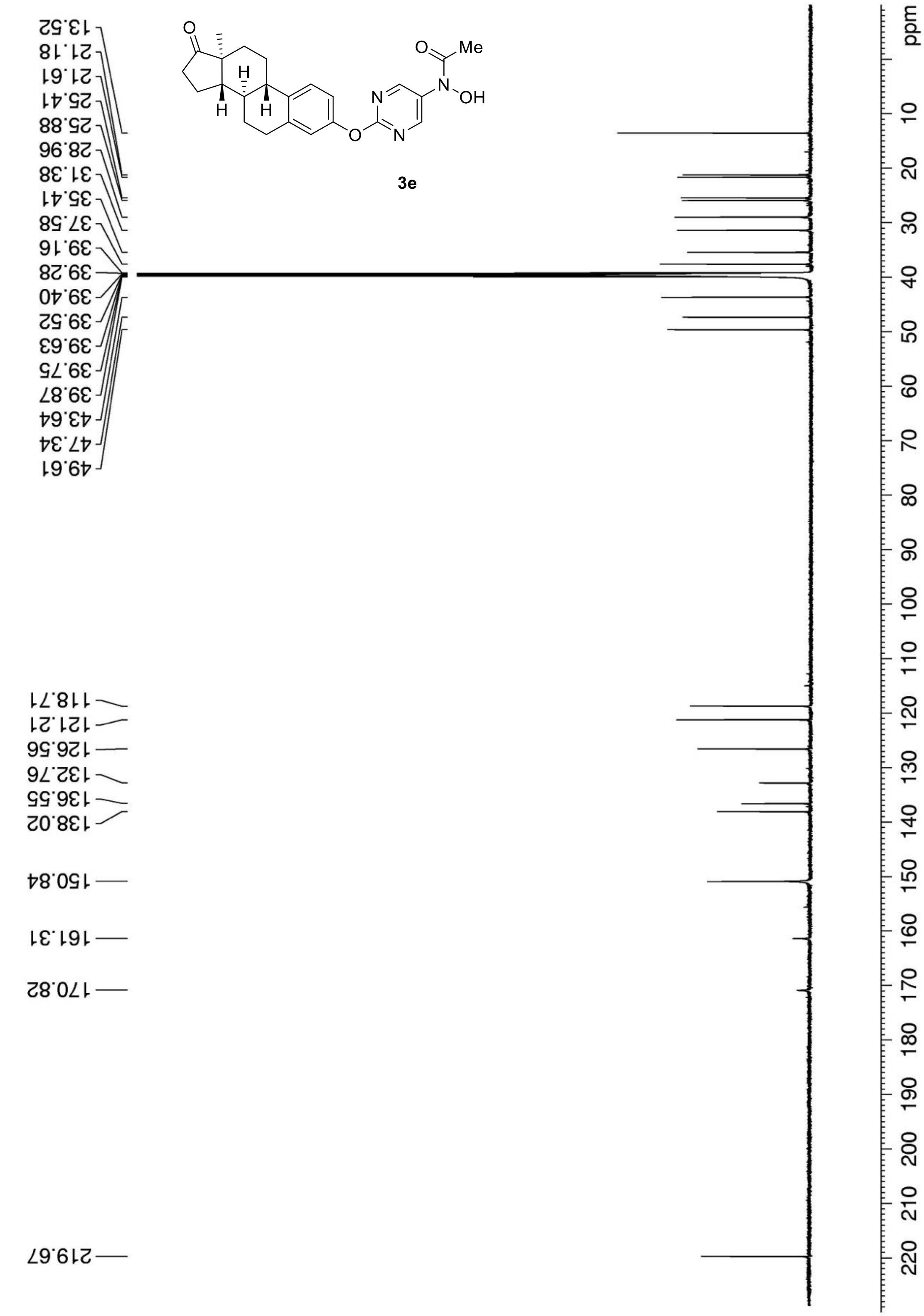

^{1}H NMR ($CDCl_3$, 25 ºC) of **2a**

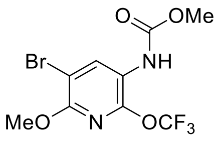

2a

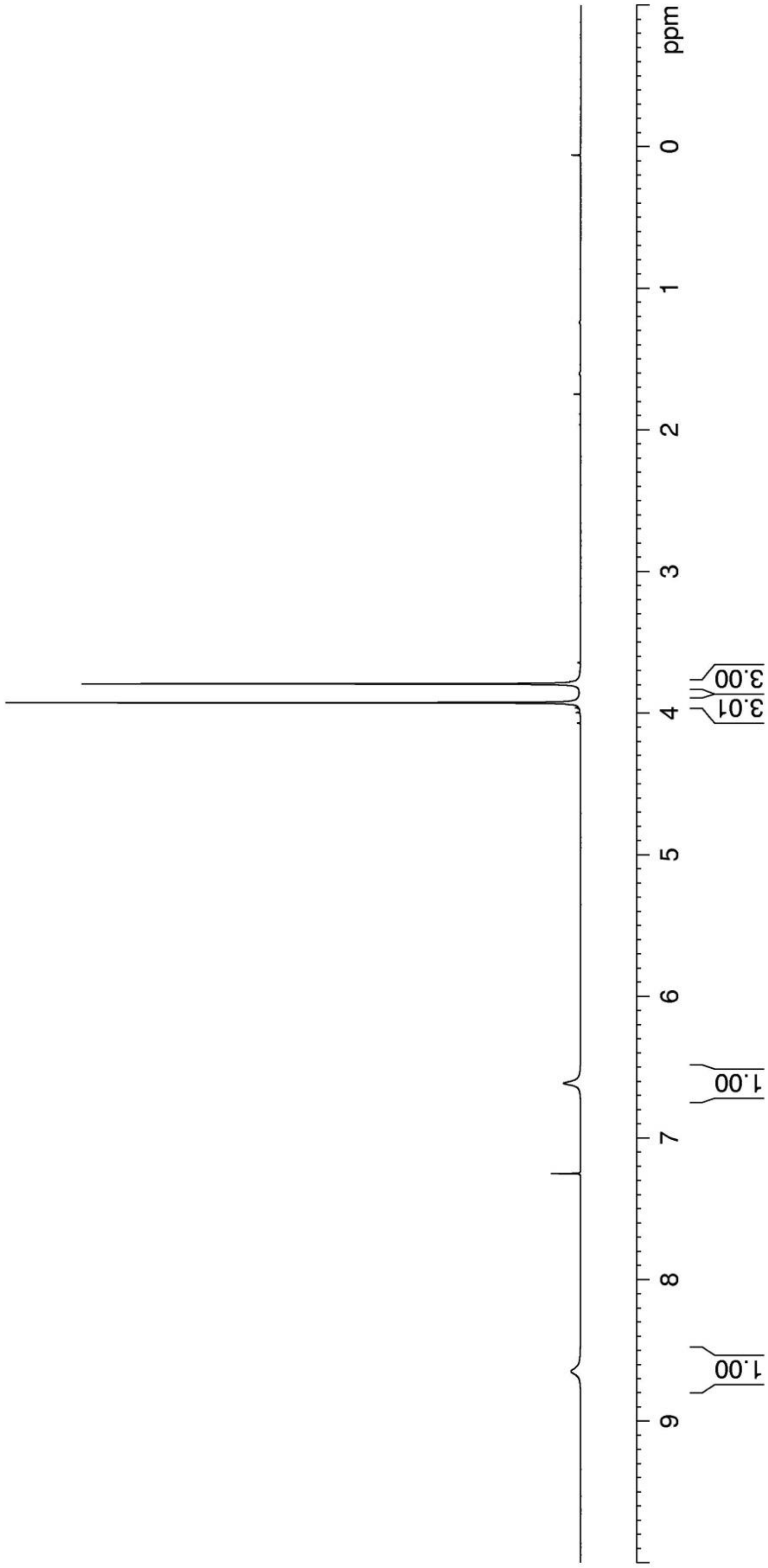

^{13}C NMR ($CDCl_3$, 25 ºC) of **2a**

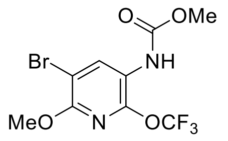

2a

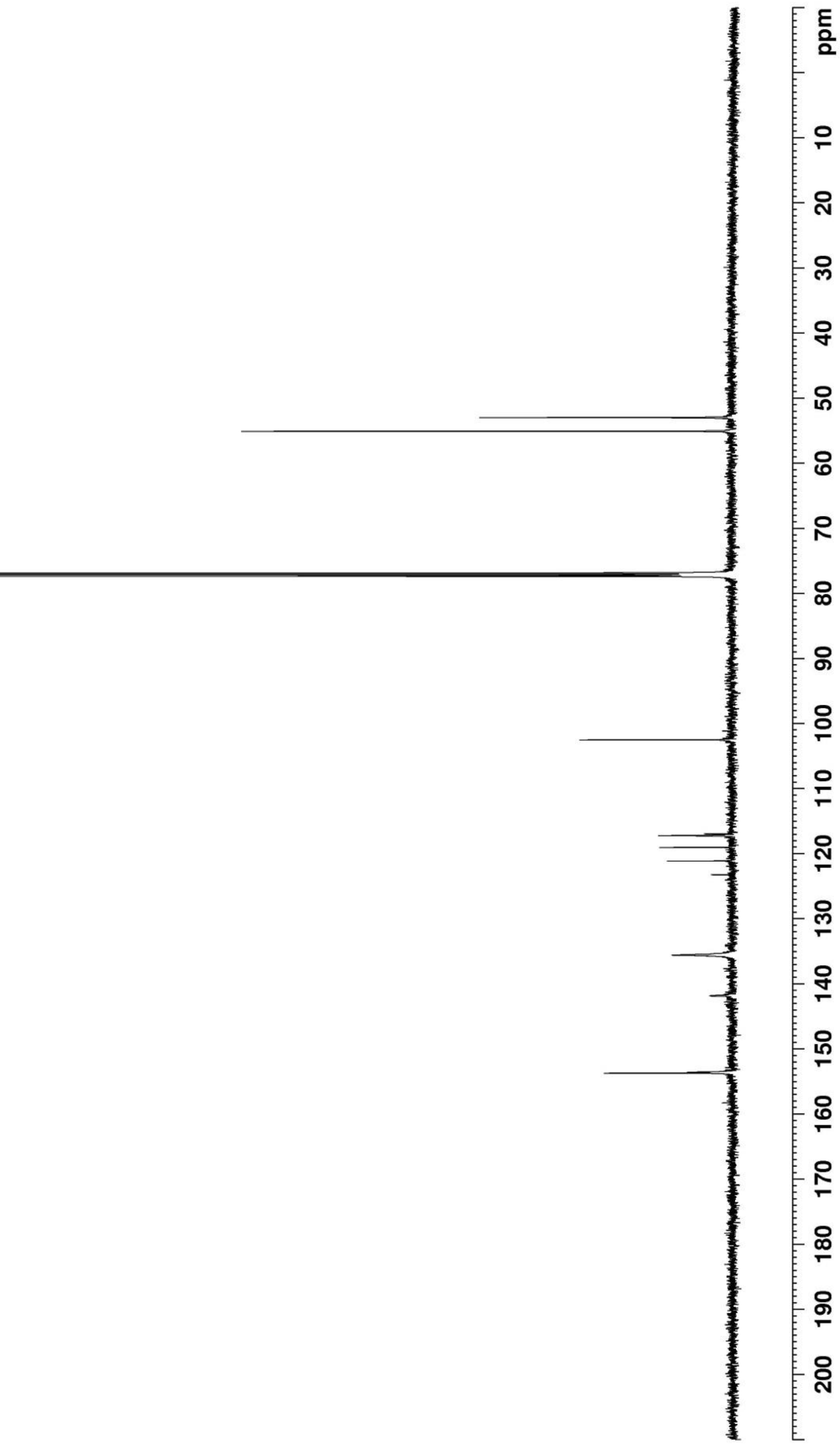

^{19}F NMR ($CDCl_3$, 25 ºC) of **2a**

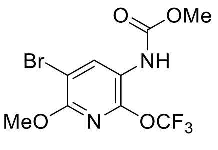

2a

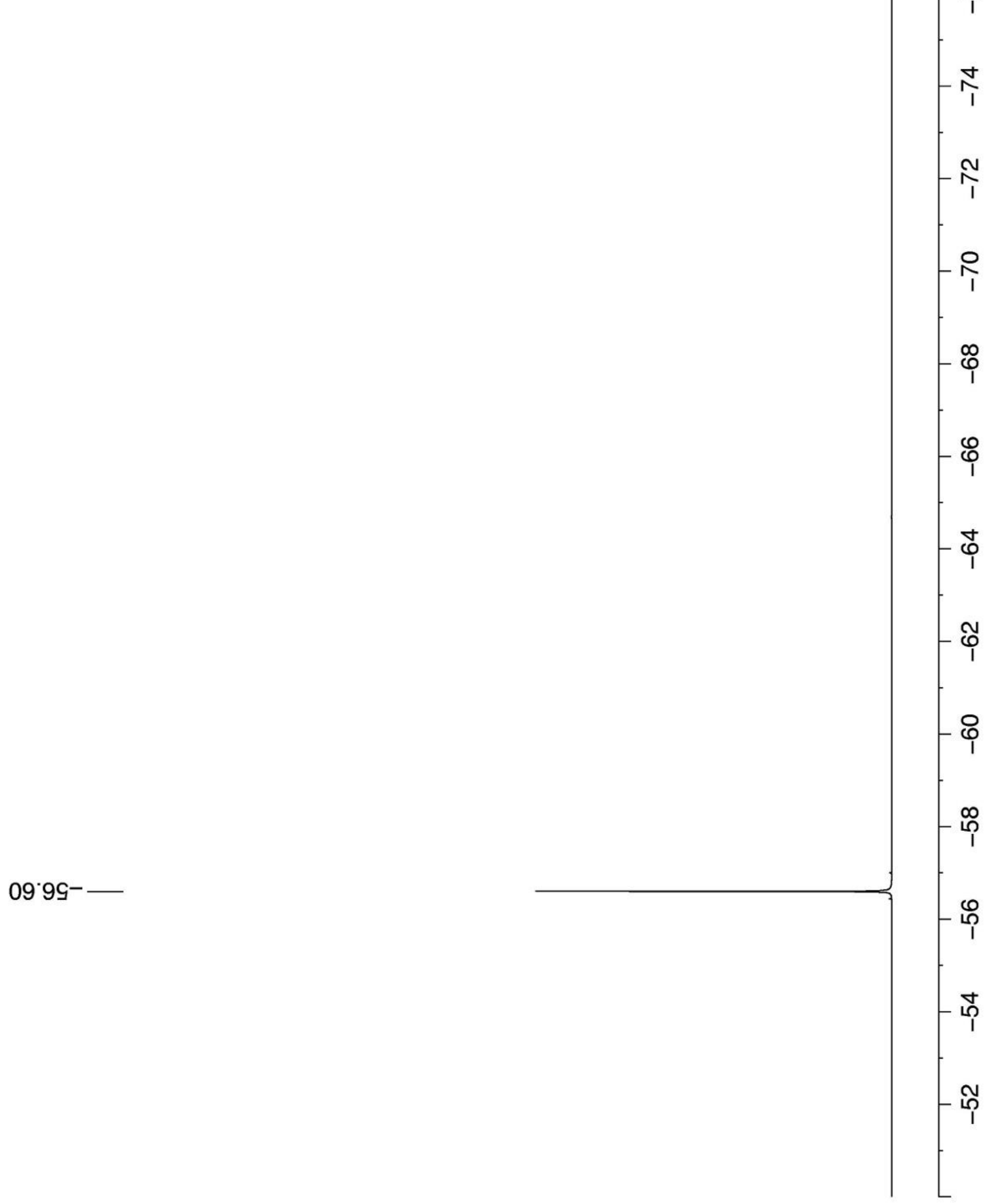

^{1}H NMR ($CDCl_3$, 25 ºC) of **2b**

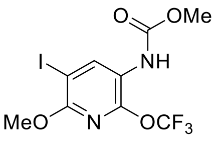

2b

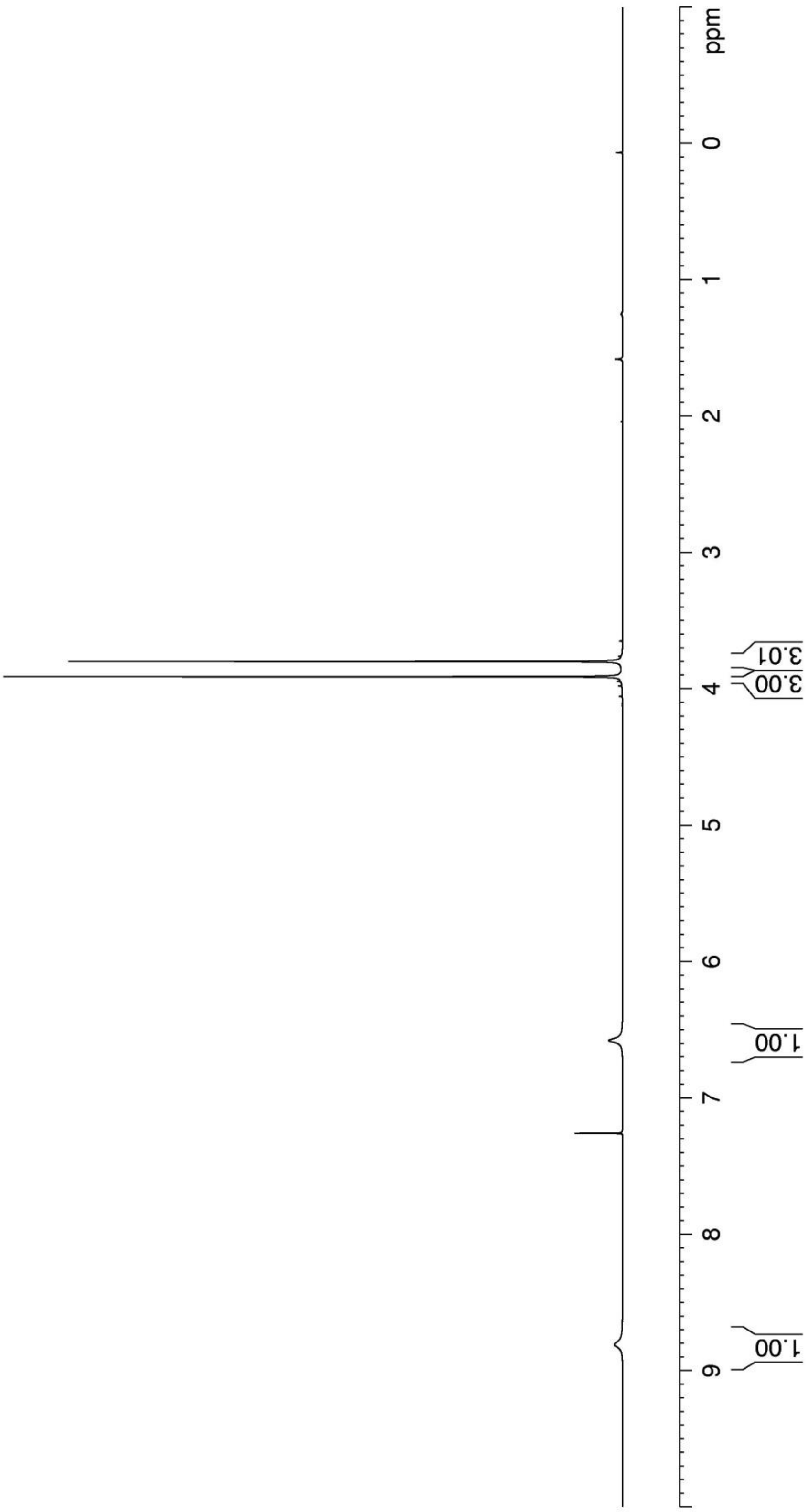

^{13}C NMR ($CDCl_3$, 25 ºC) of **2b**

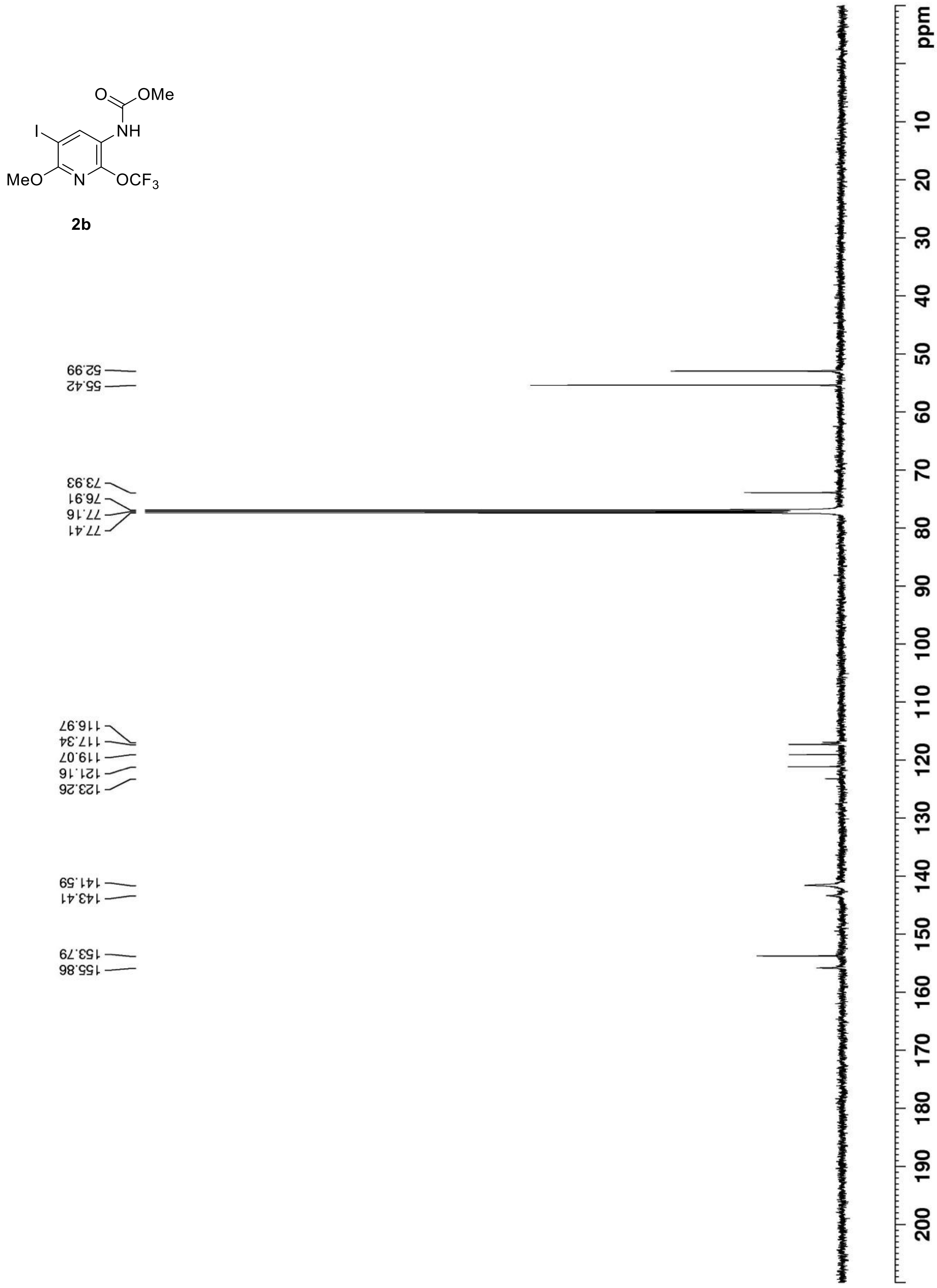

^{19}F NMR ($CDCl_3$, 25 ºC) of **2b**

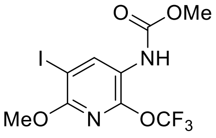

2b

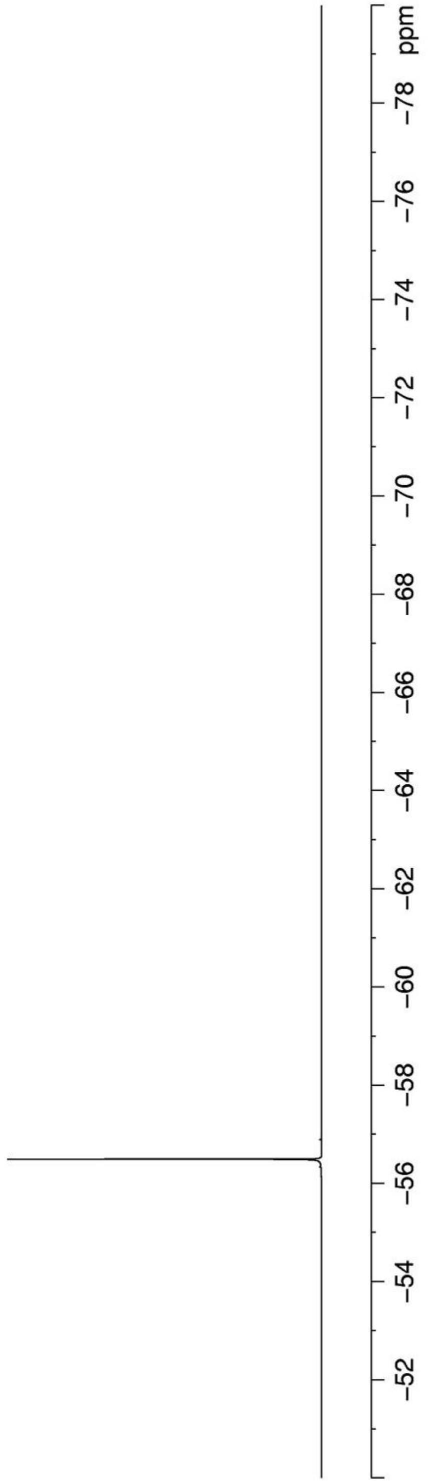

^{1}H NMR ($CDCl_3$, 25 ºC) of **2c**

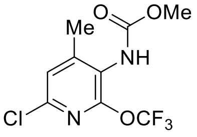

2c

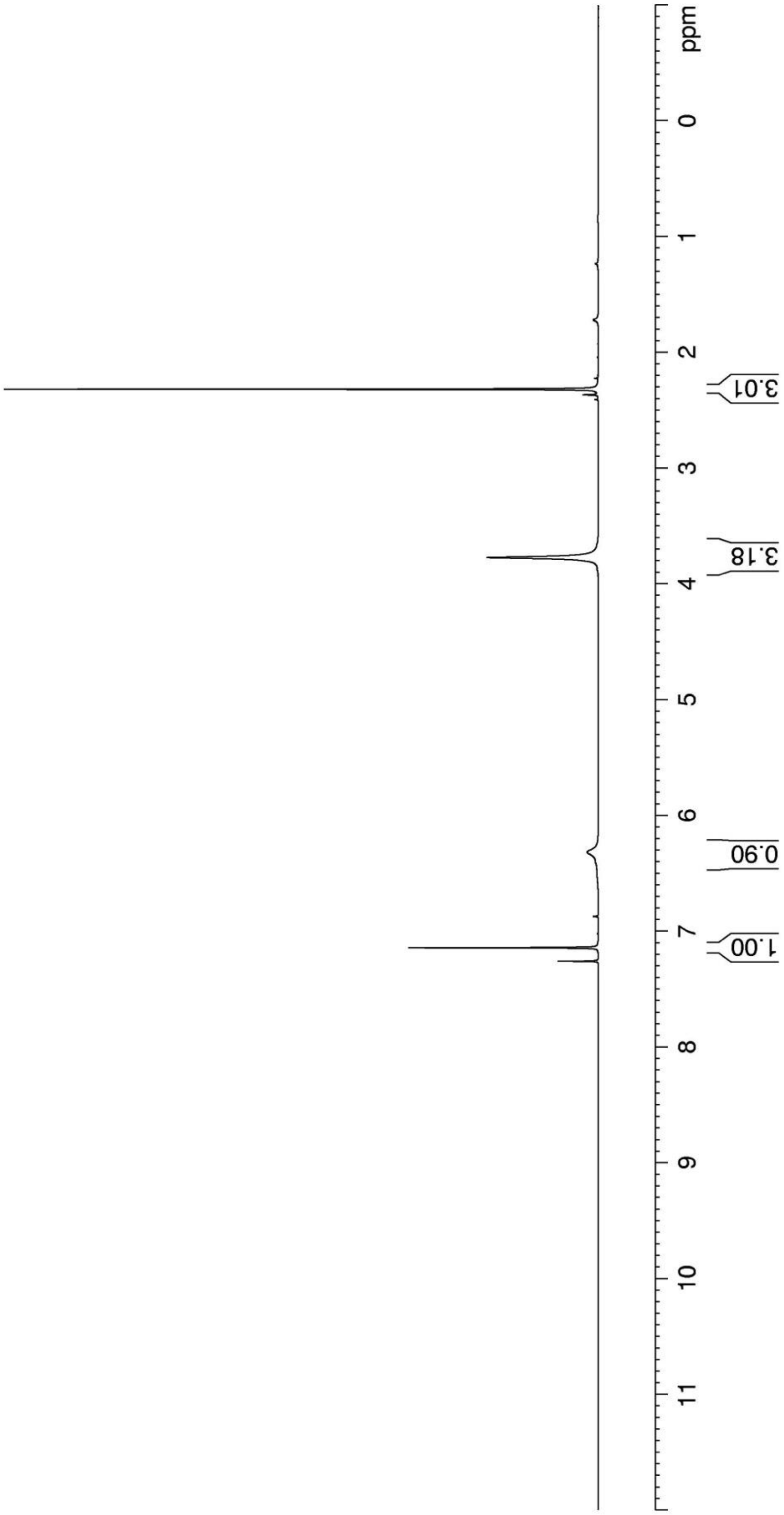

^{13}C NMR ($CDCl_3$, 25 ºC) of **2c**

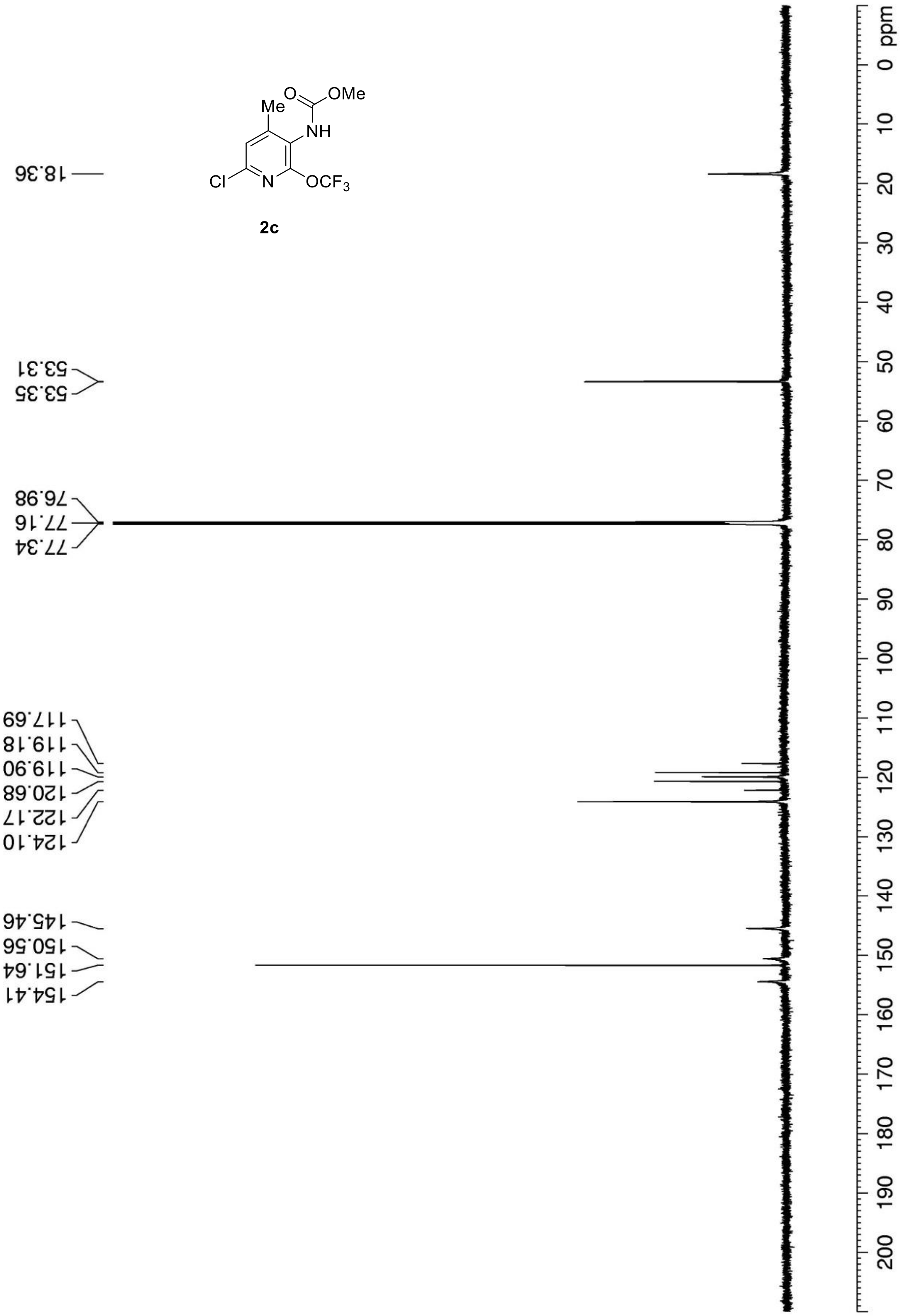

^{19}F NMR (DMSO, 90 ºC) of **2c**

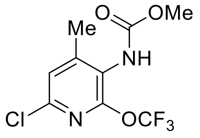

2c

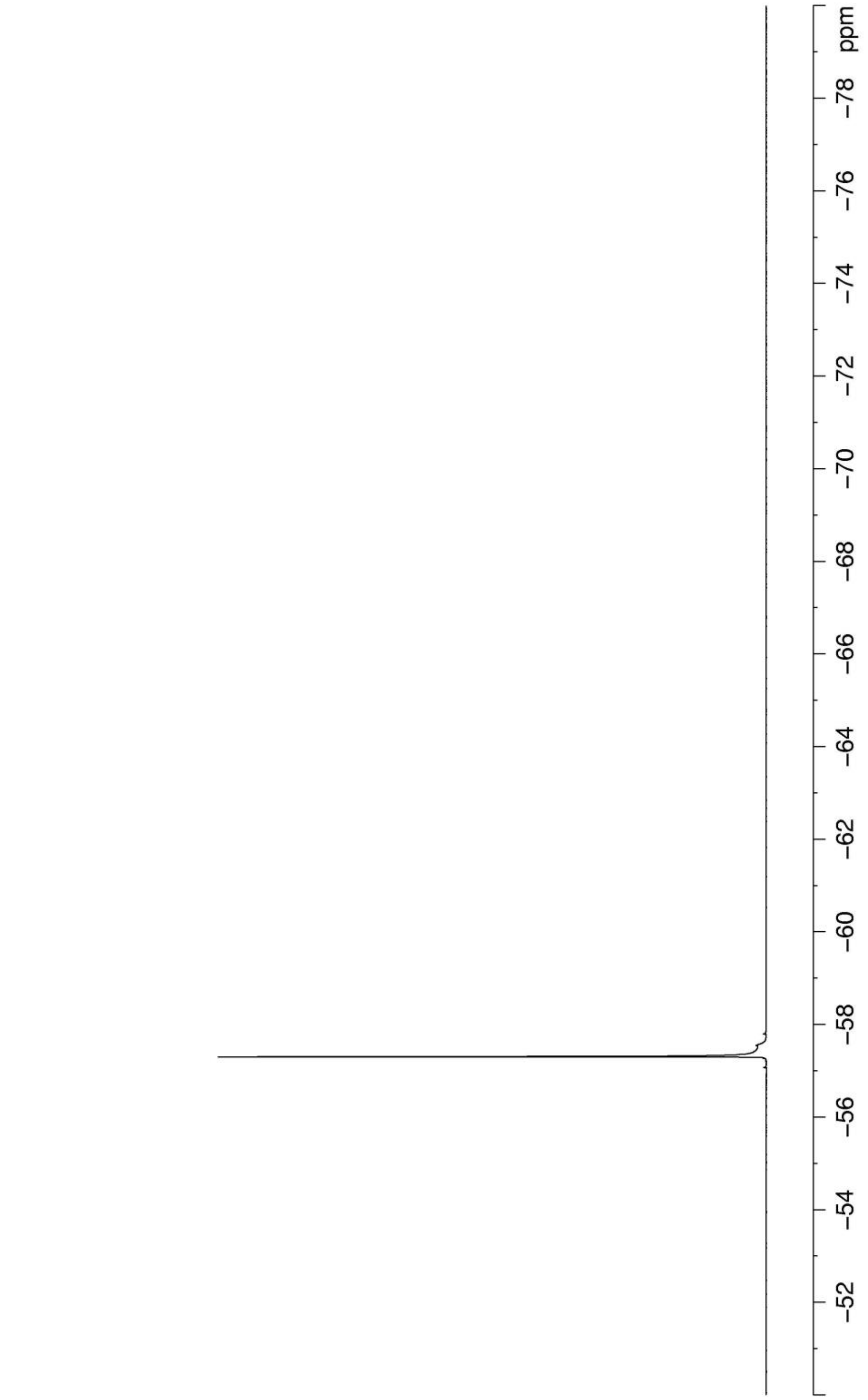

^{1}H NMR ($CDCl_3$, 25 ºC) of **2d**

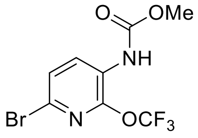

2d

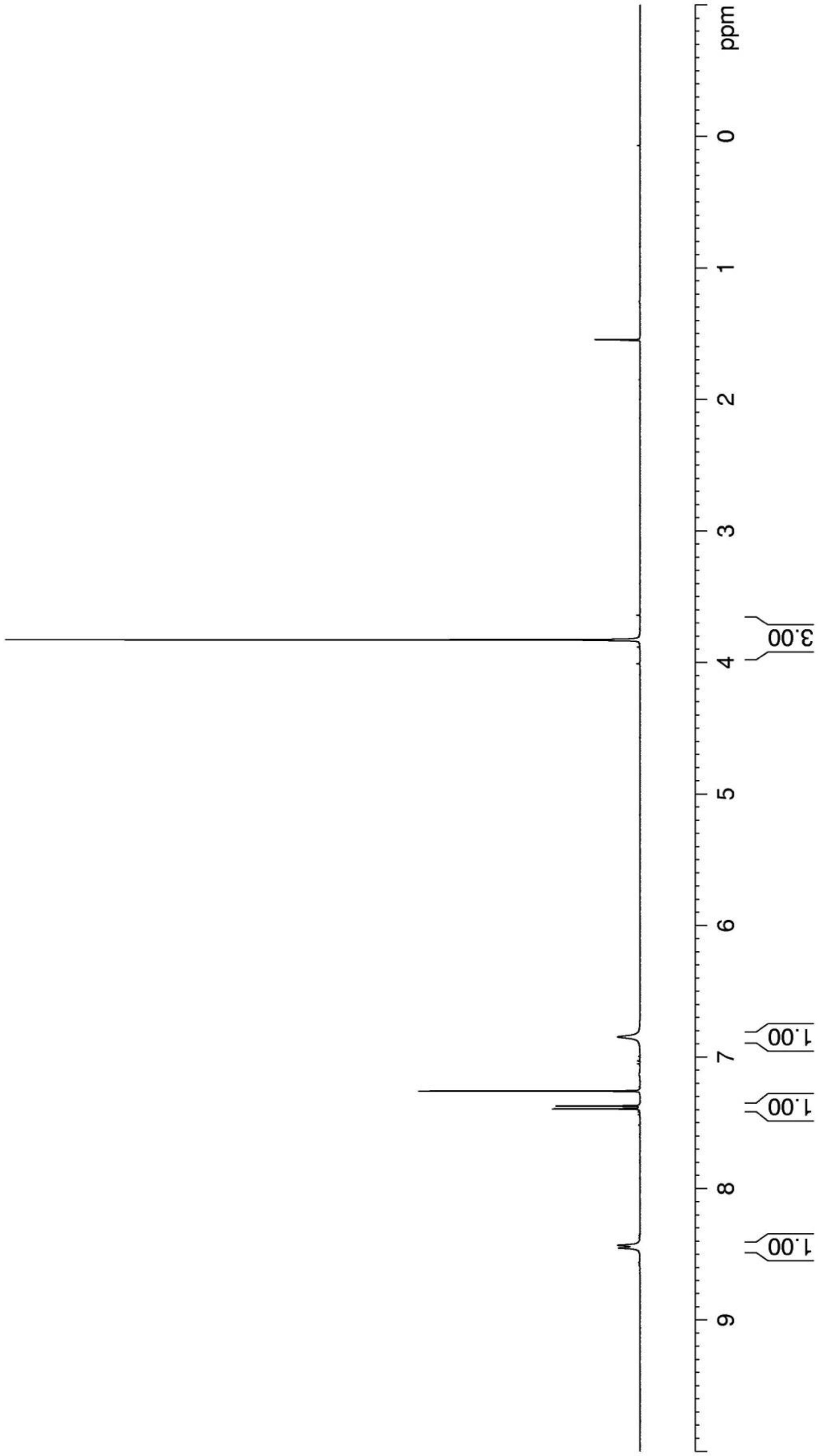

^{13}C NMR ($CDCl_3$, 25 ºC) of **2d**

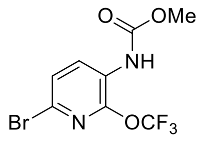

2d

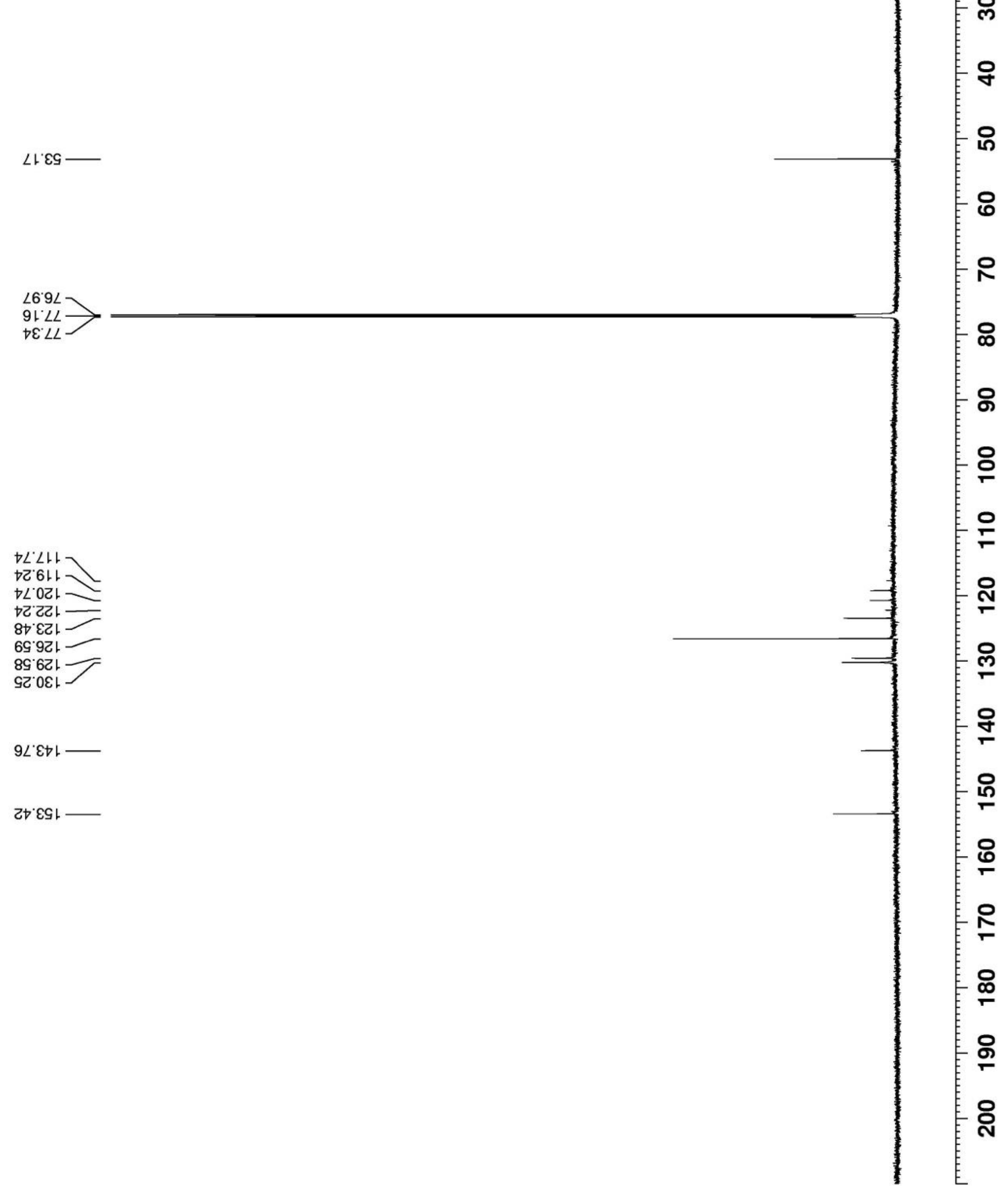

^{19}F NMR ($CDCl_3$, 25 ºC) of **2d**

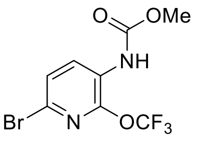

2d

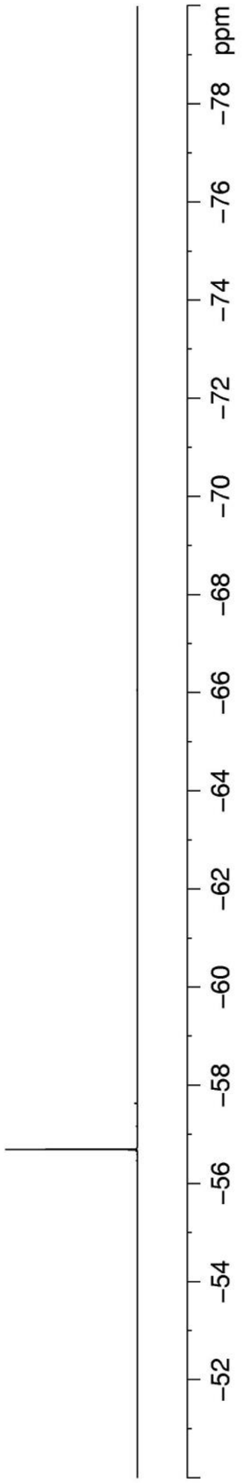

^{1}H NMR ($CDCl_3$, 25 ºC) of **2d-II**

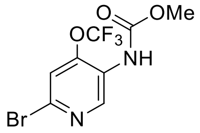

2d-II

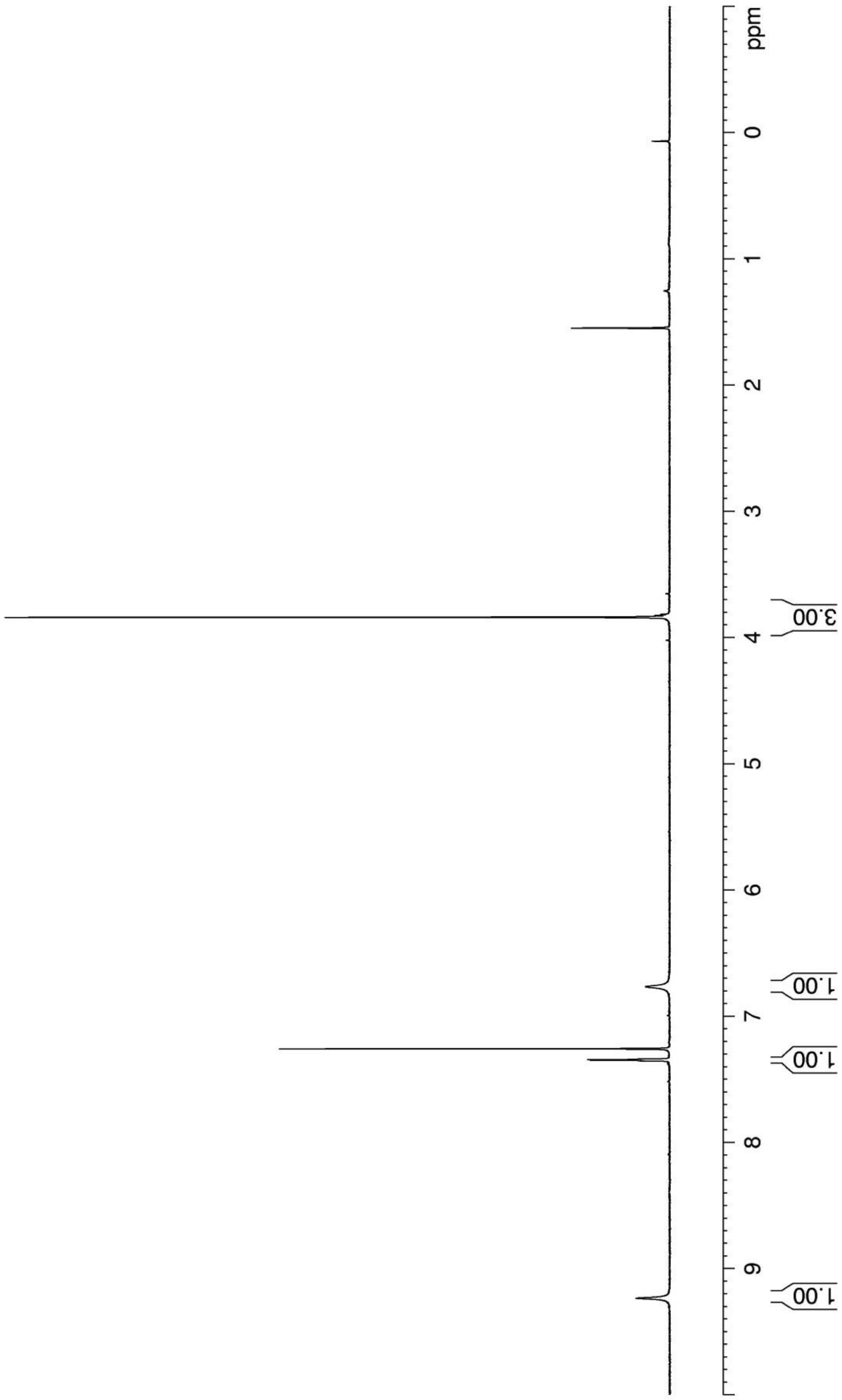

^{13}C NMR ($CDCl_3$, 25 ºC) of **2d-II**

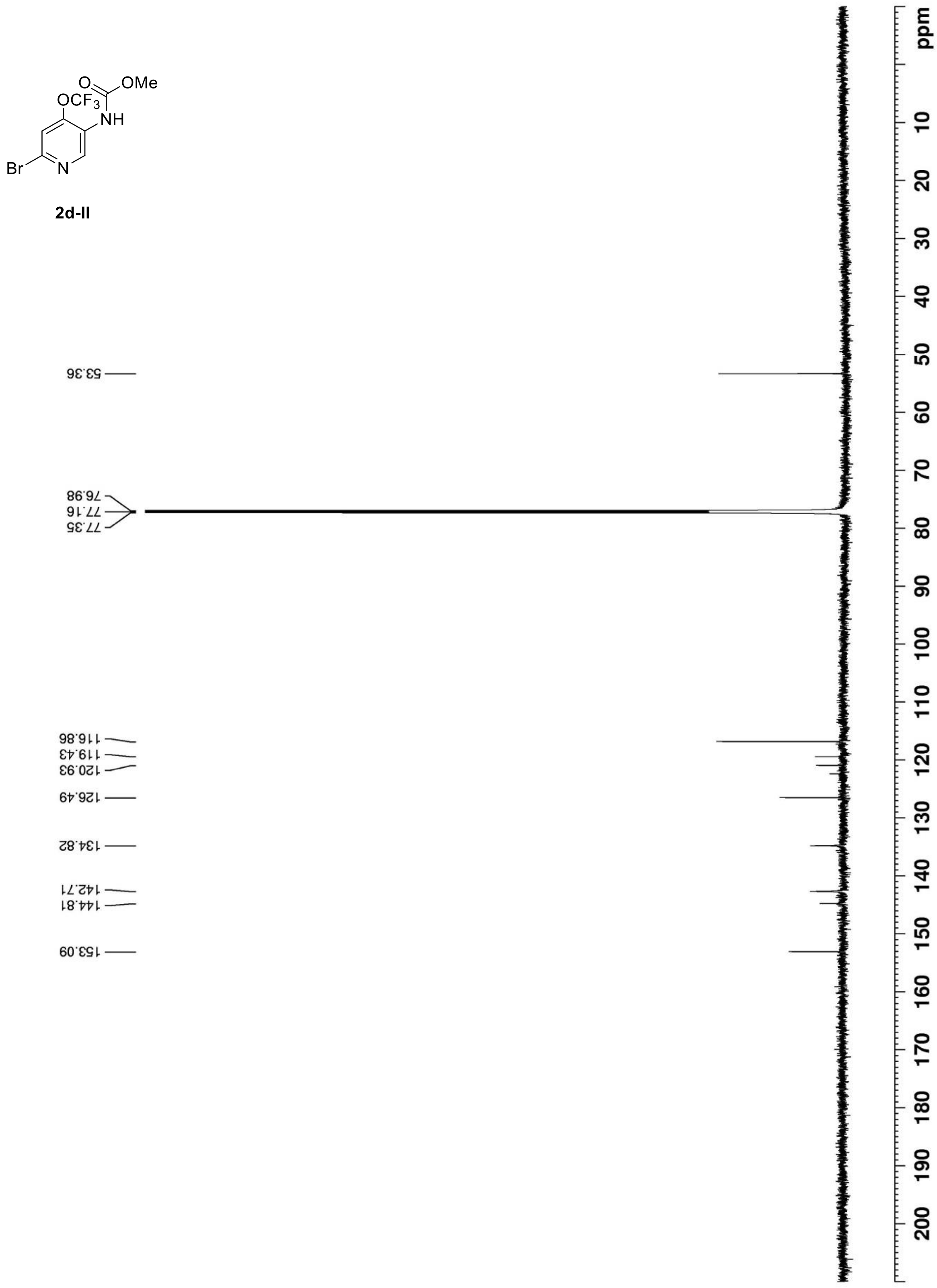

^{19}F NMR ($CDCl_3$, 25 ºC) of **2d-II**

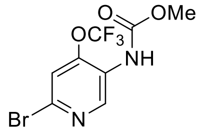

2d-II

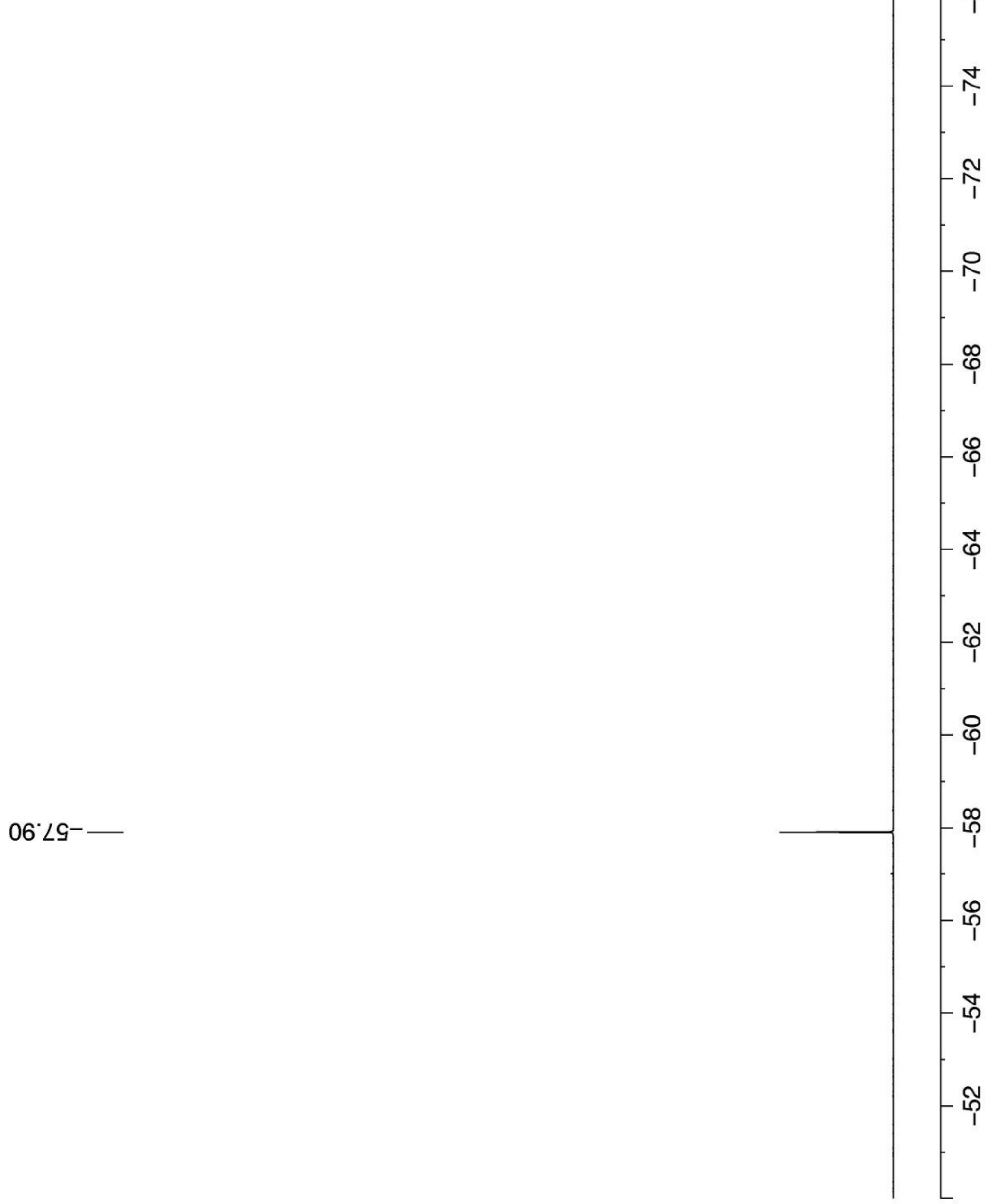

^{1}H NMR ($CDCl_3$, 25 ºC) of **2e**

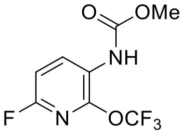

2e

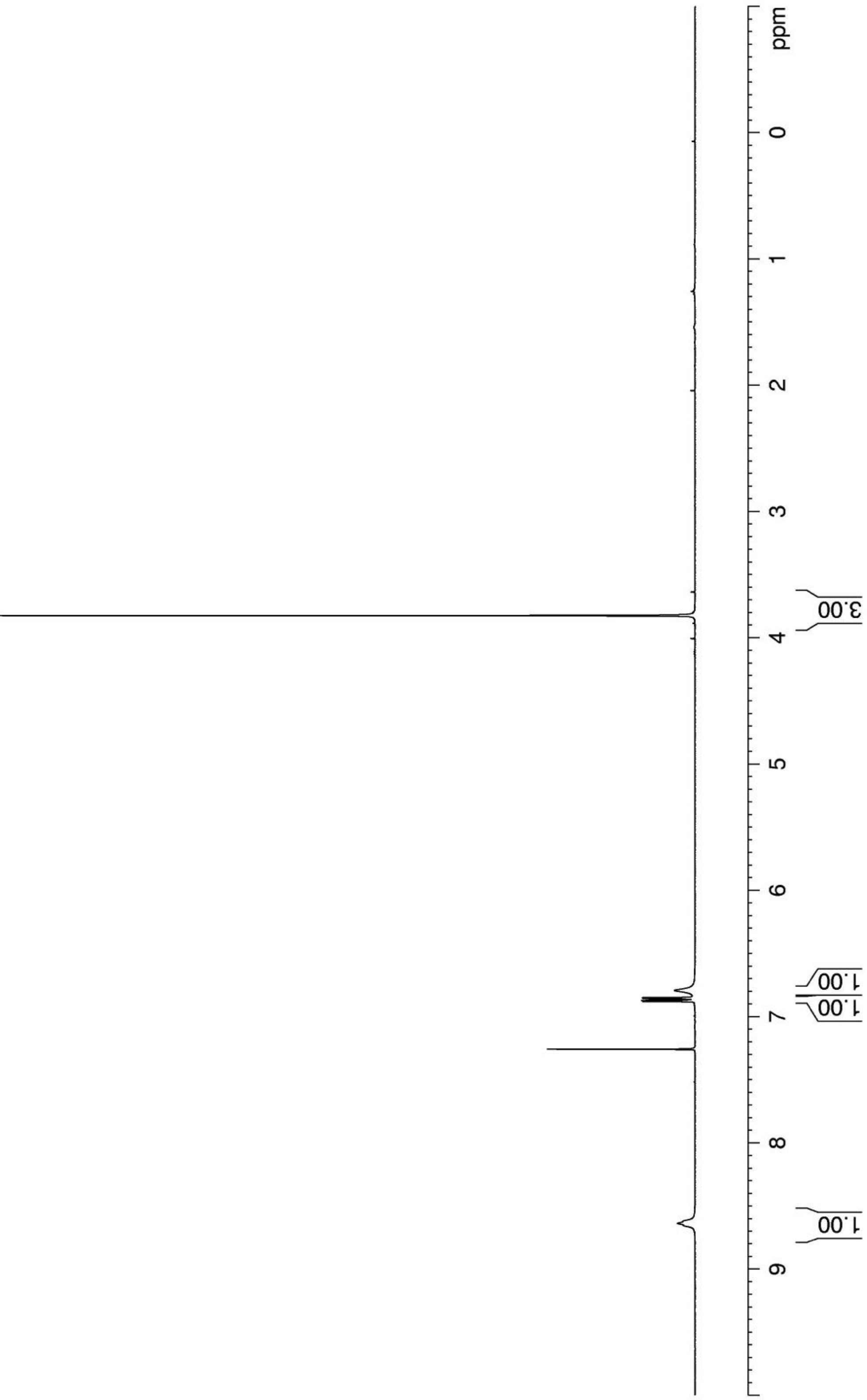

^{13}C NMR ($CDCl_3$, 25 ºC) of **2e**

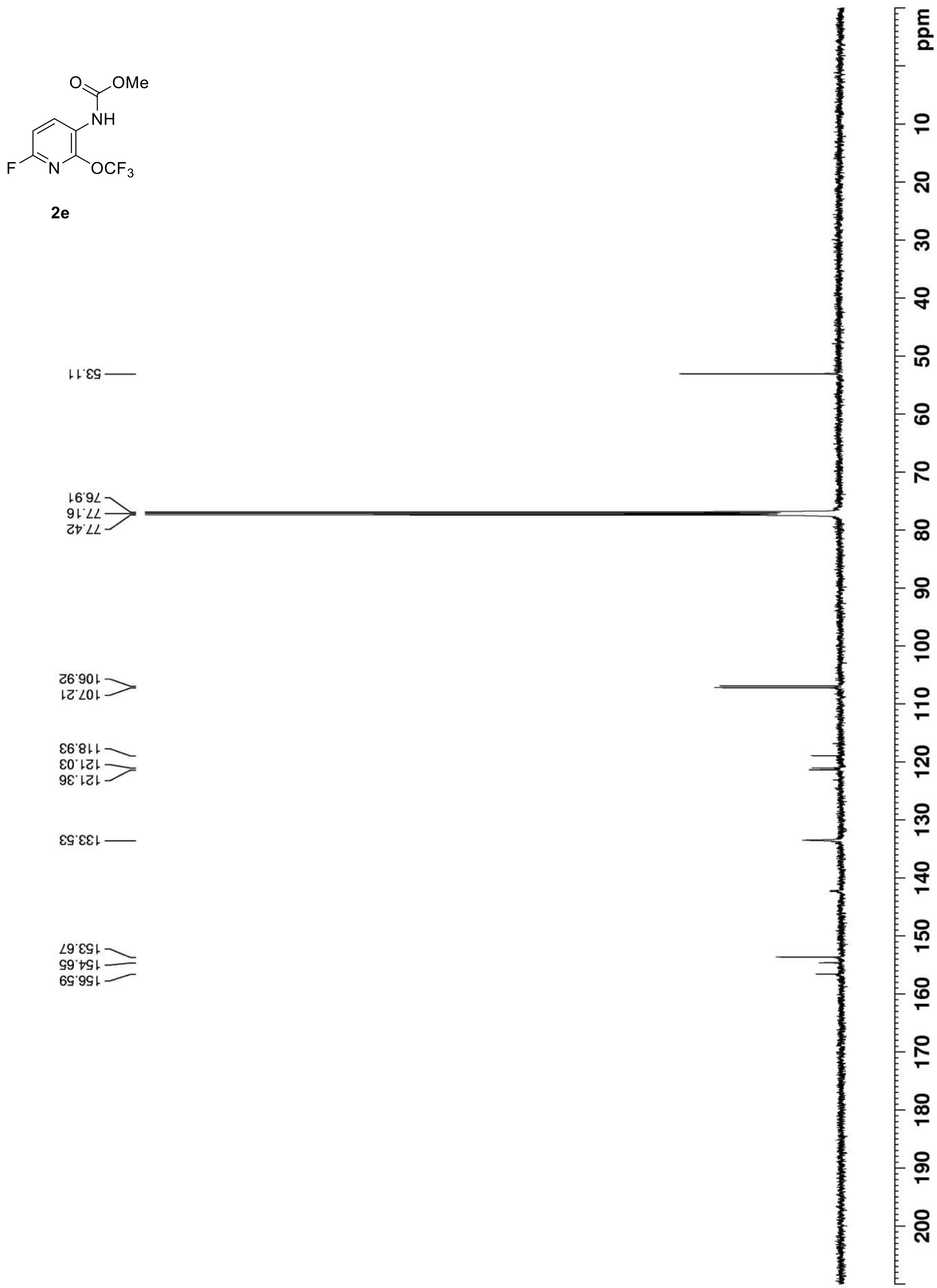

^{19}F NMR ($CDCl_3$, 25 ºC) of **2e**

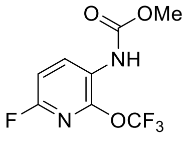

2e

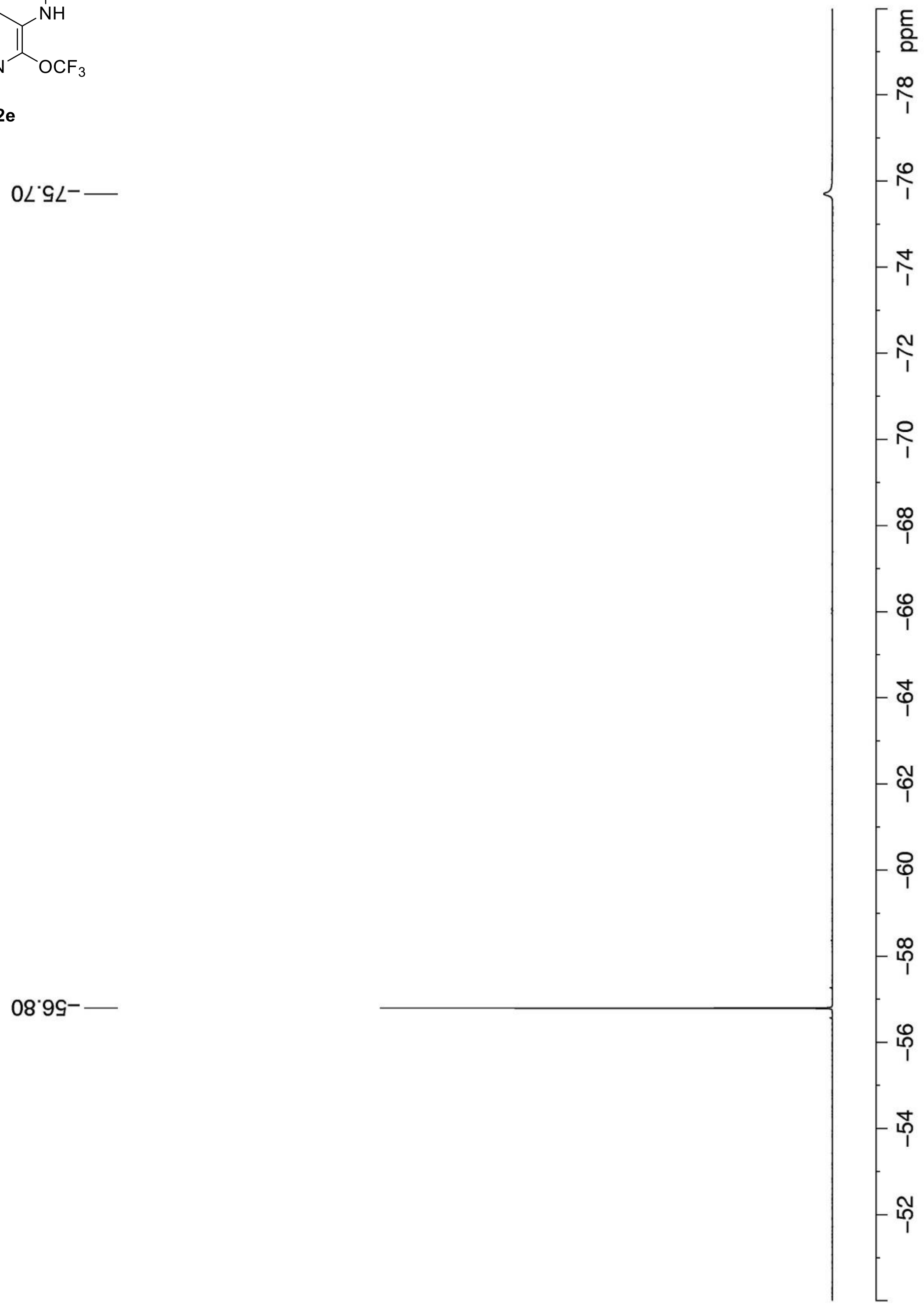

^{1}H NMR ($CDCl_3$, 25 ºC) of **2e-II**

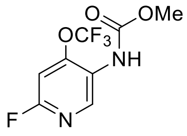

2e-II

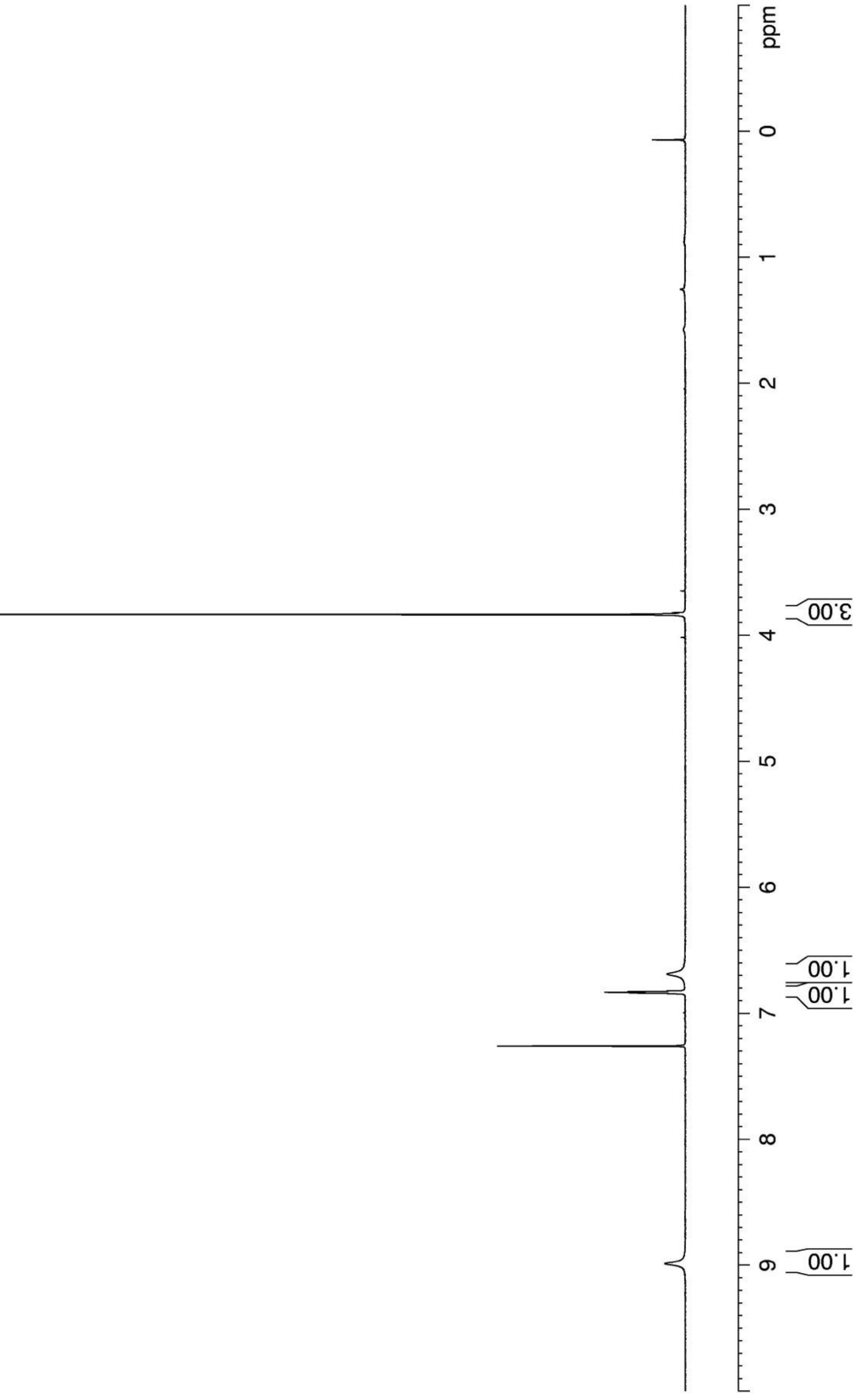

^{13}C NMR ($CDCl_3$, 25 ºC) of **2e-II**

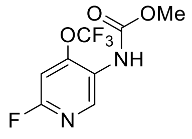

2e-II

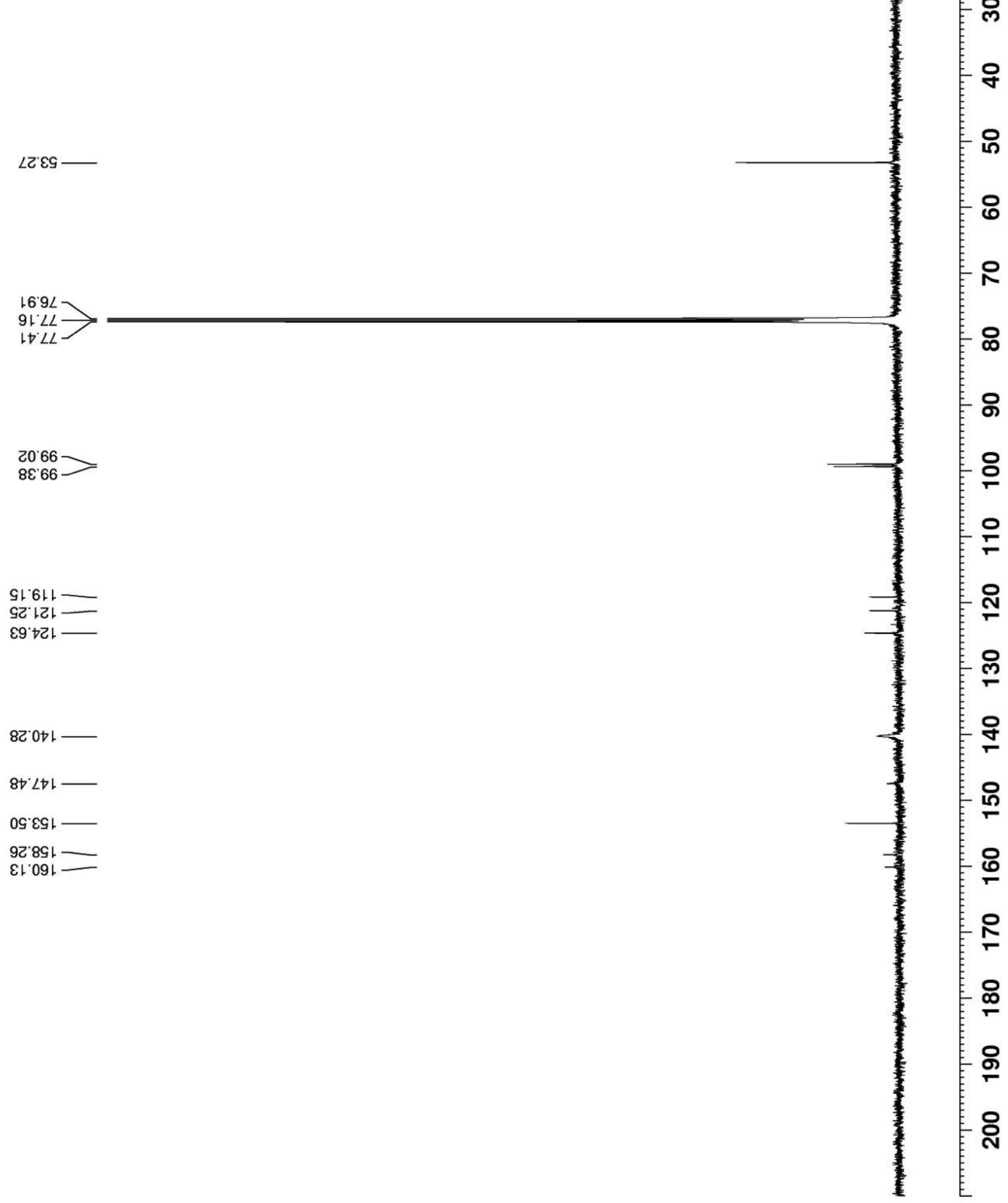

^{19}F NMR ($CDCl_3$, 25 ºC) of **2e-II**

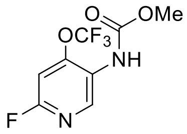

2e-II

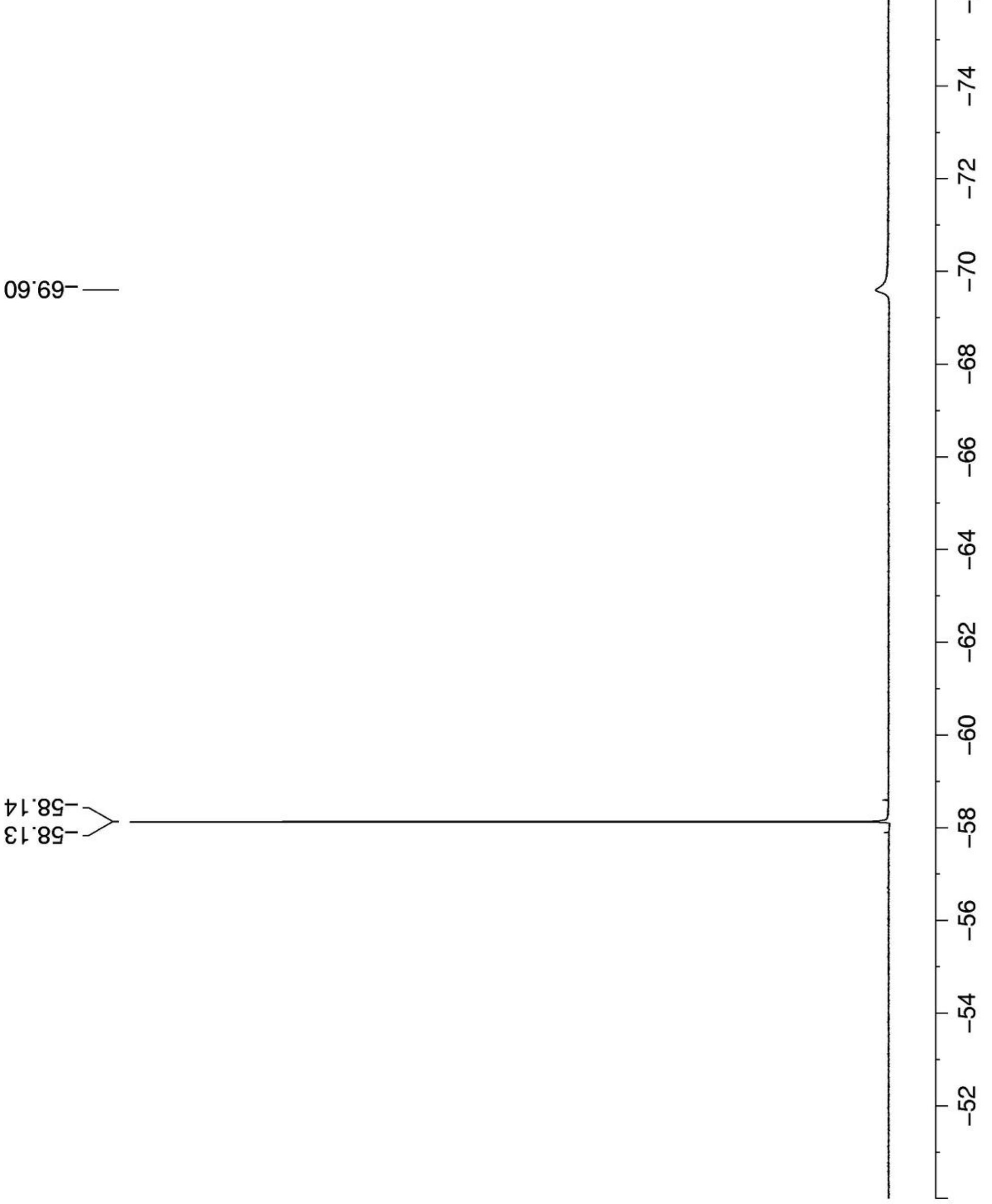

^{1}H NMR ($CDCl_3$, 25 ºC) of **2f**

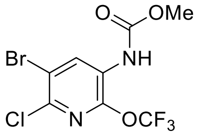

2f

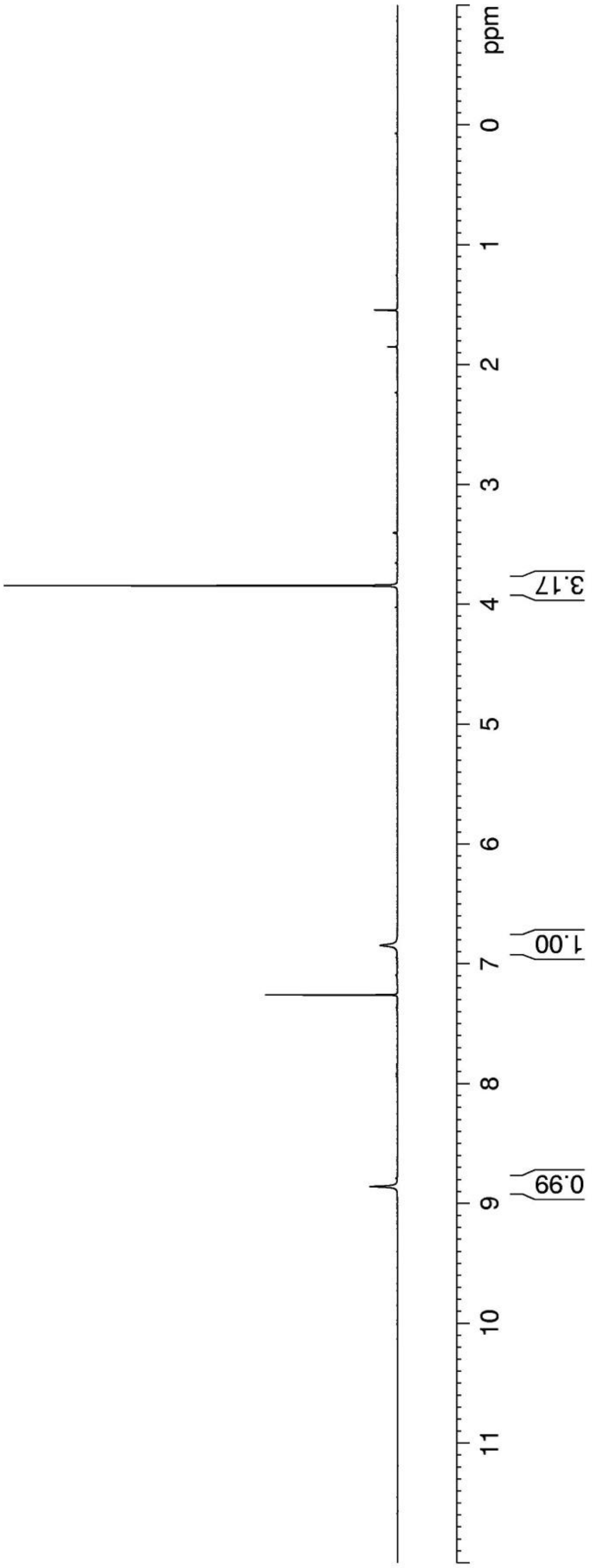

^{13}C NMR ($CDCl_3$, 25 ºC) of **2f**

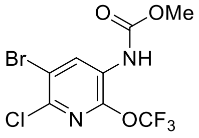

2f

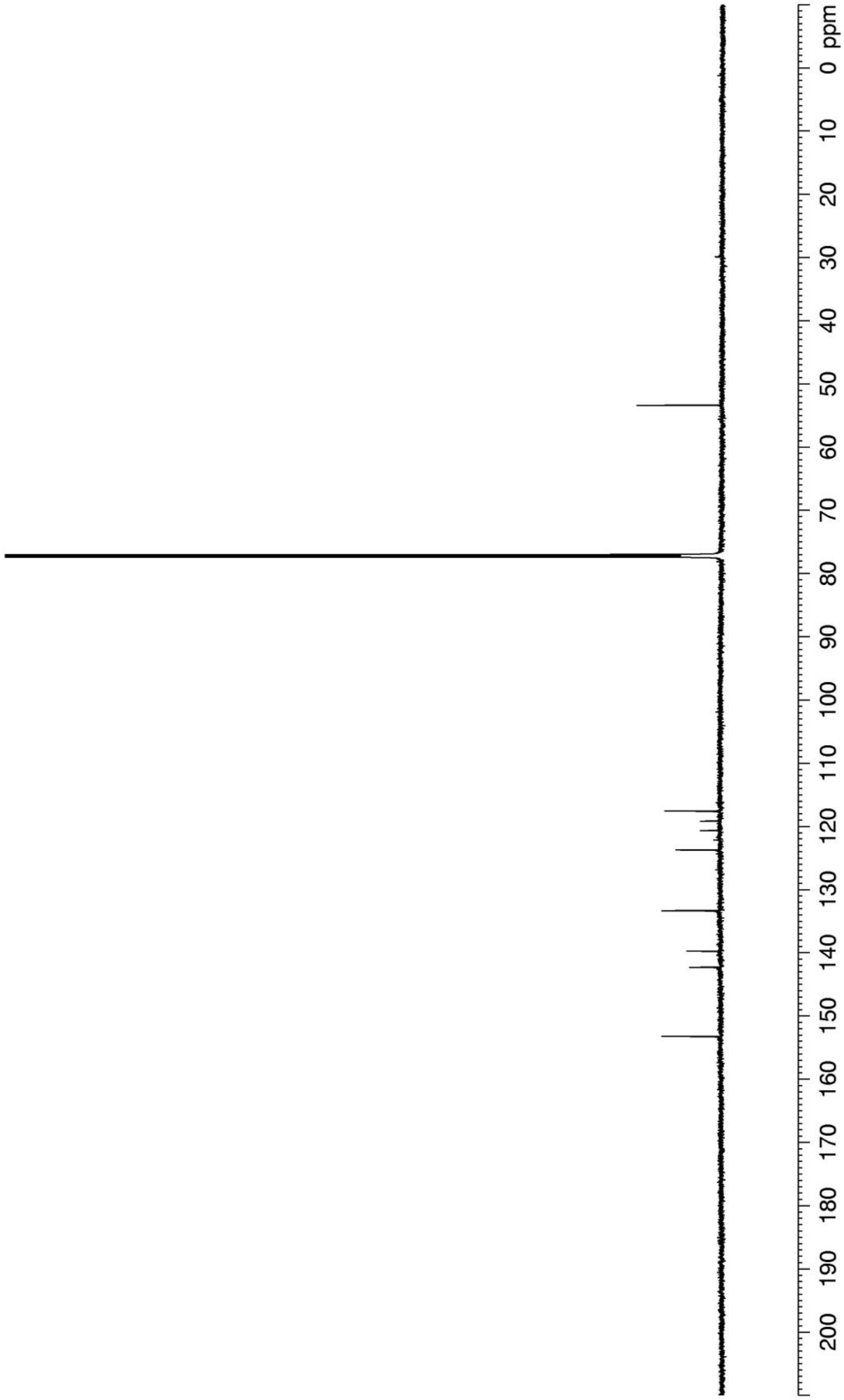

^{19}F NMR ($CDCl_3$, 25 ºC) of **2f**

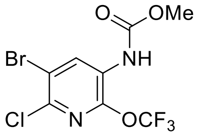

2f

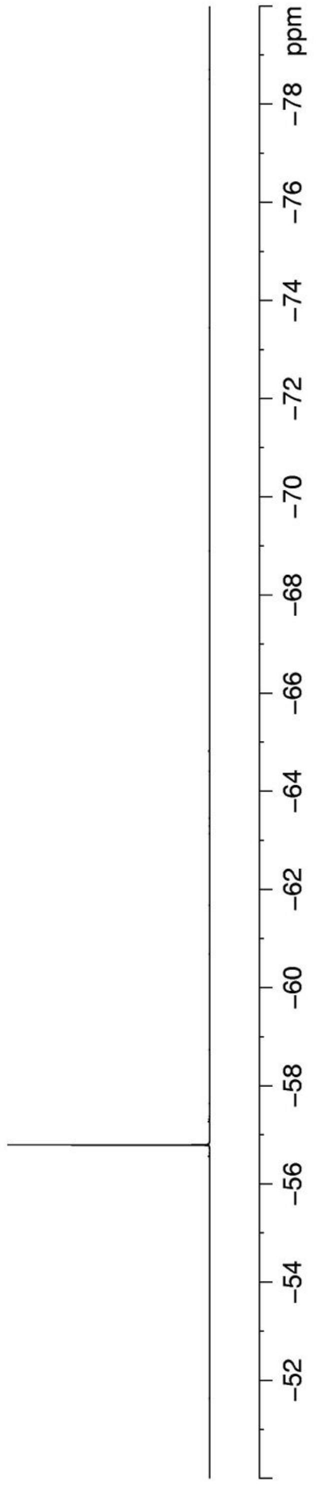

^{1}H NMR ($CDCl_3$, 25 ºC) of **2f-II**

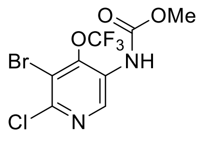

2f-II

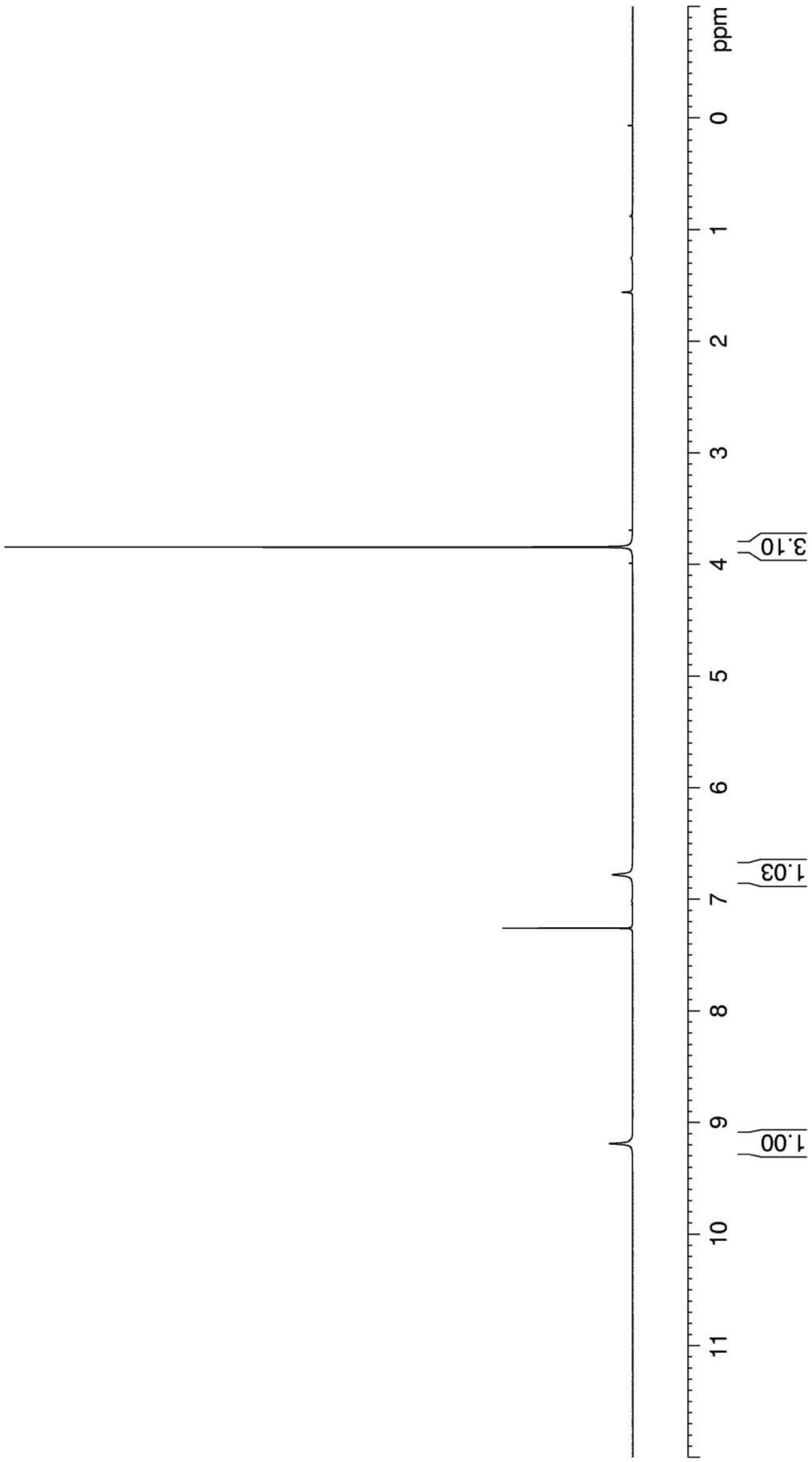

^{13}C NMR ($CDCl_3$, 25 ºC) of **2f-II**

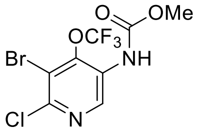

2f-II

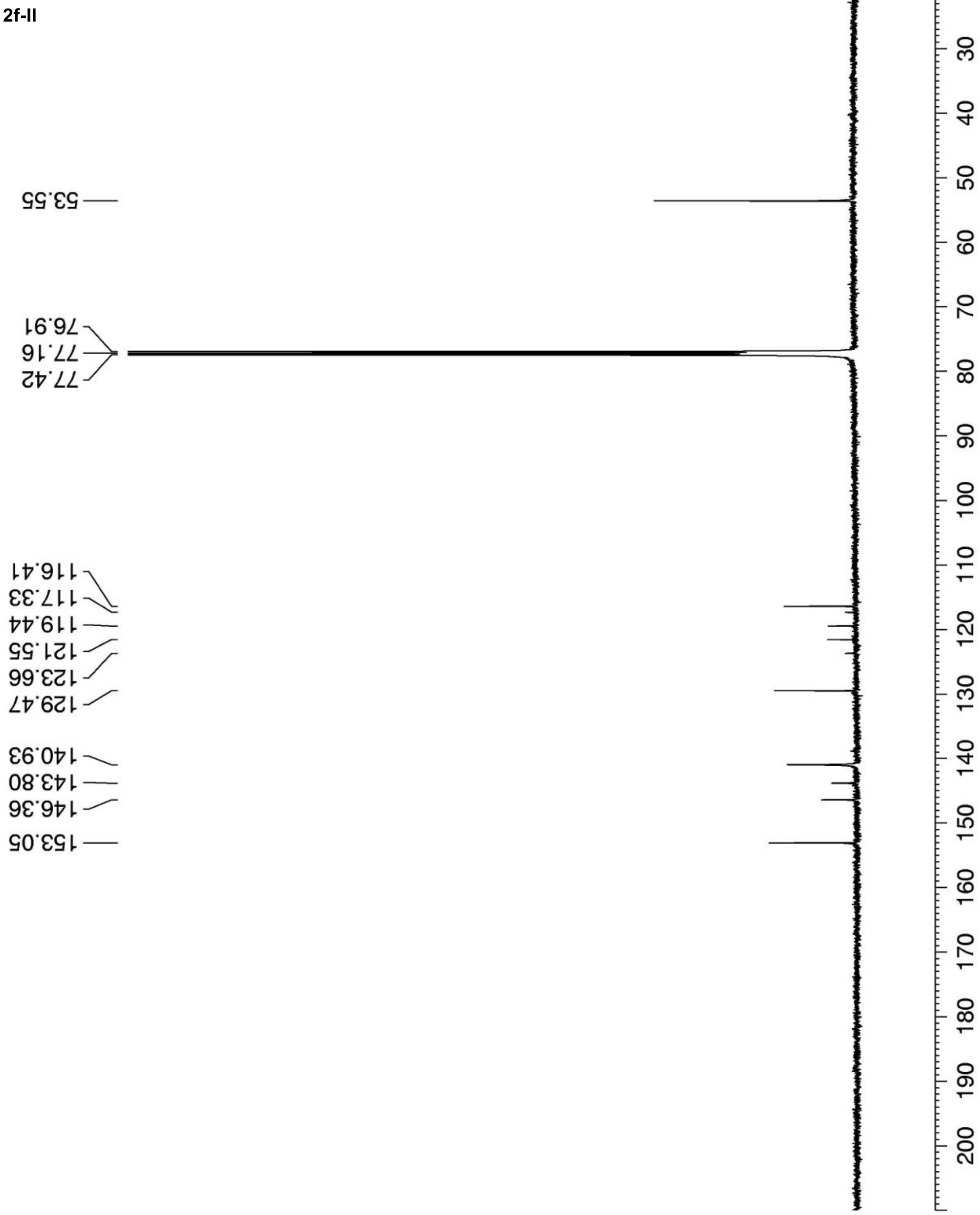

^{19}F NMR ($CDCl_3$, 25 ºC) of **2f-II**

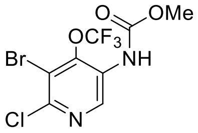

2f-II

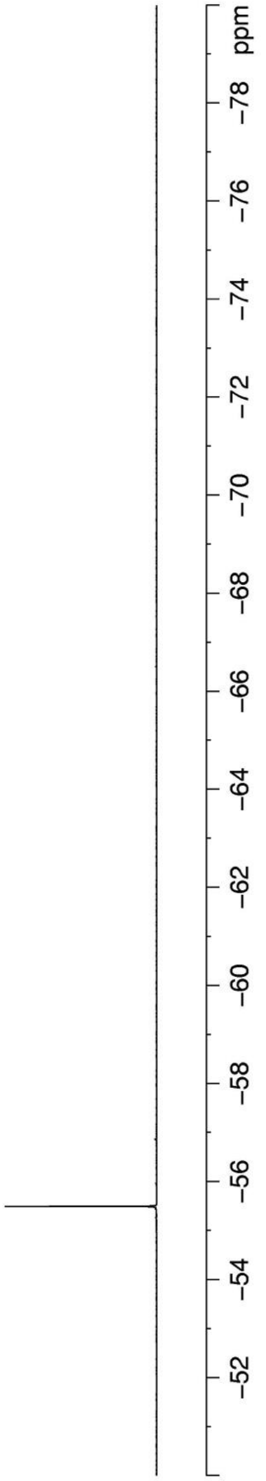

^{1}H NMR ($CDCl_3$, 25 ºC) of **2g**

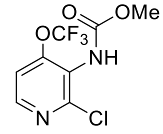

2g

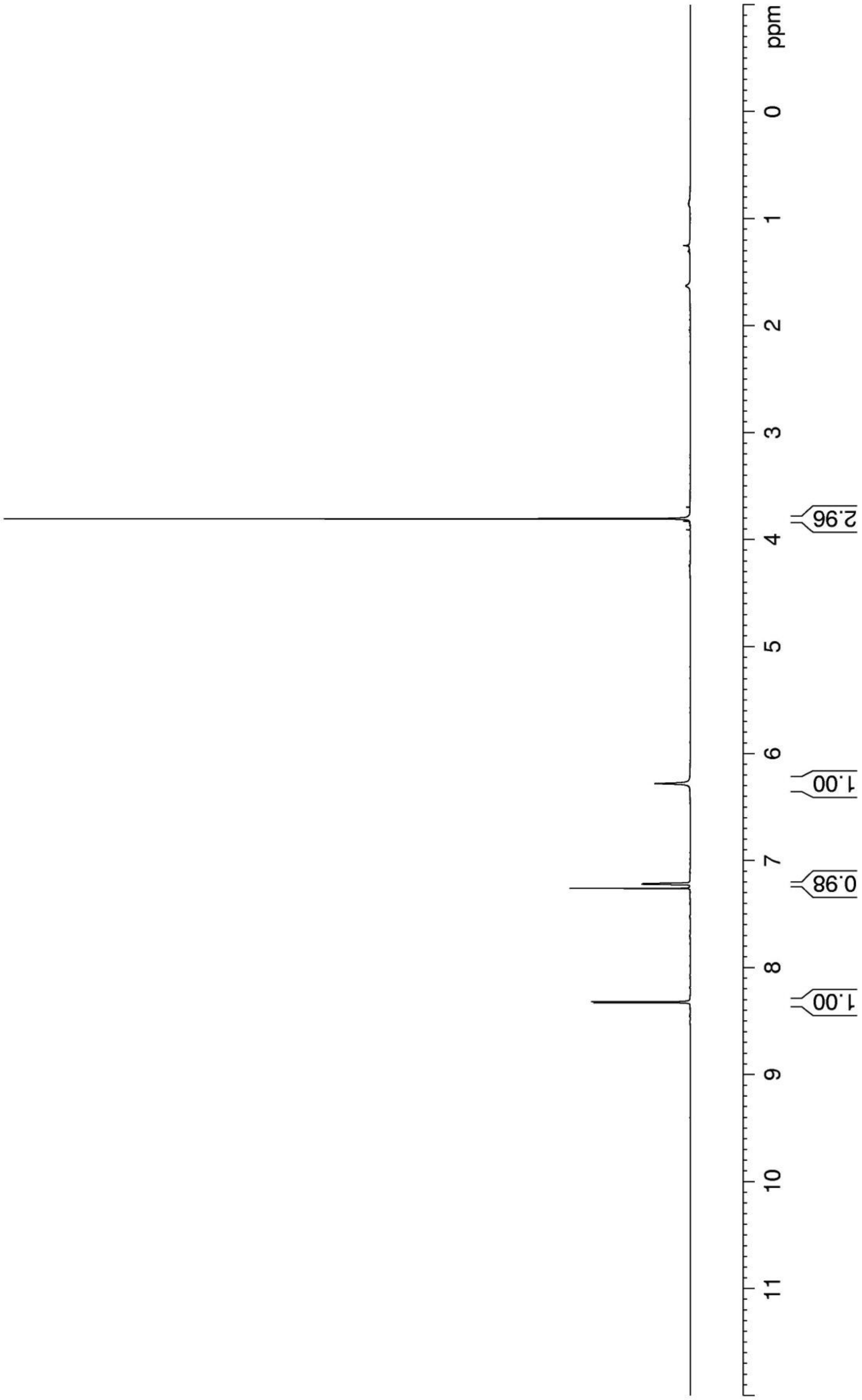

^{13}C NMR ($CDCl_3$, 25 ºC) of **2g**

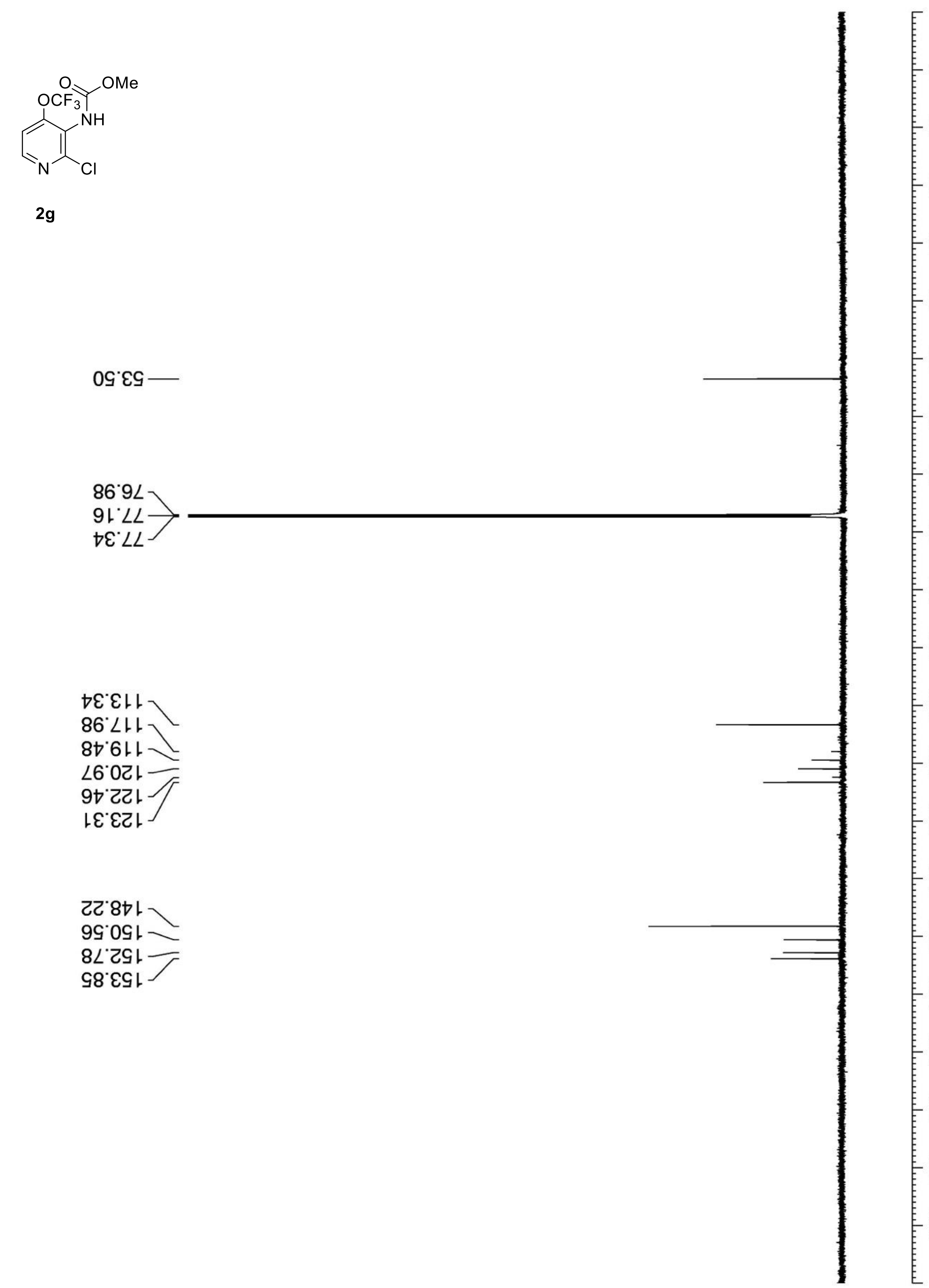

^{19}F NMR ($CDCl_3$, 25 ºC) of **2g**

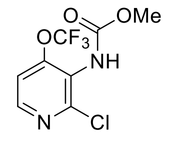

2g

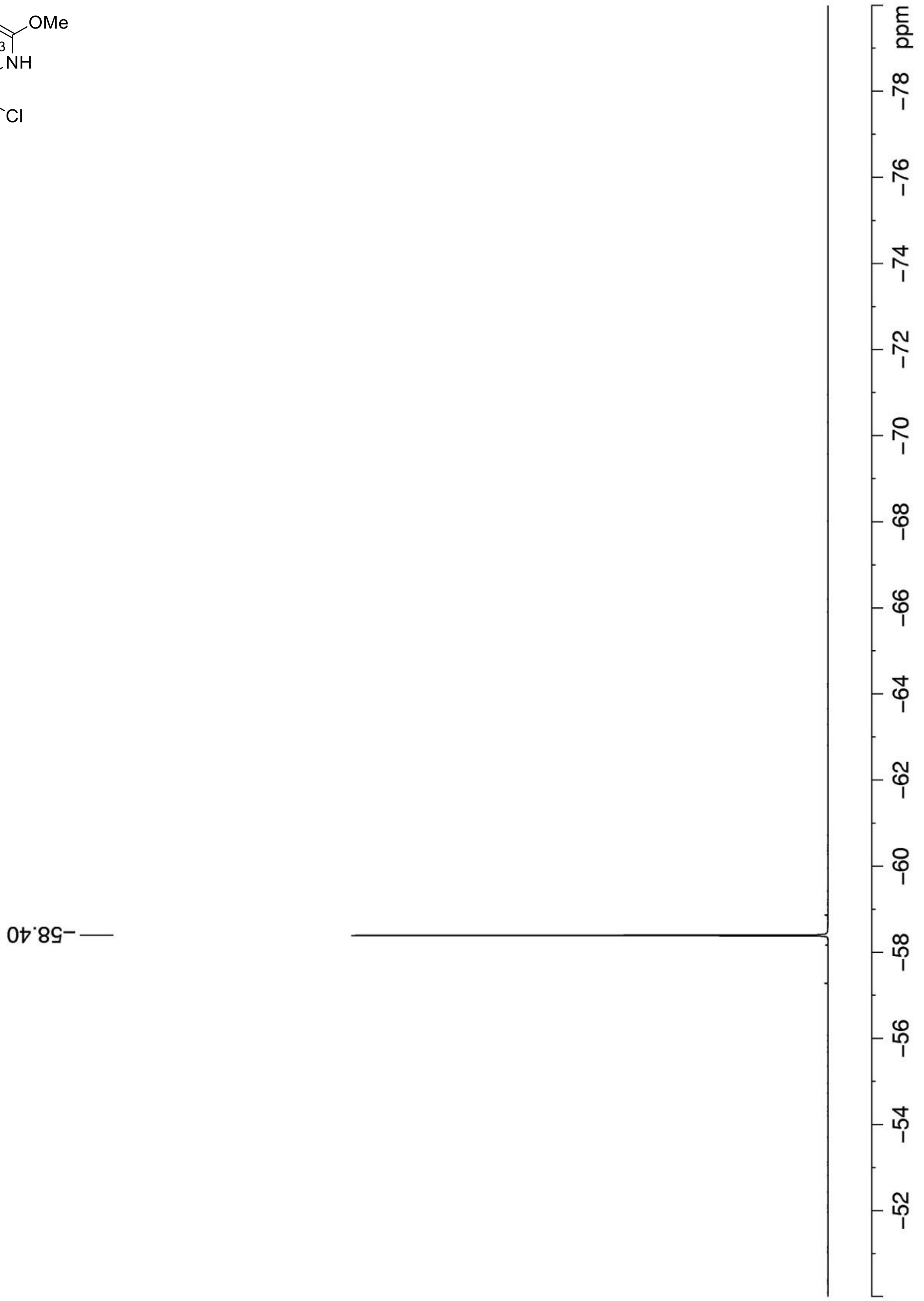

^{1}H NMR ($CDCl_3$, 25 ºC) of **2g-II**

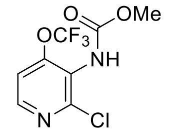

2g

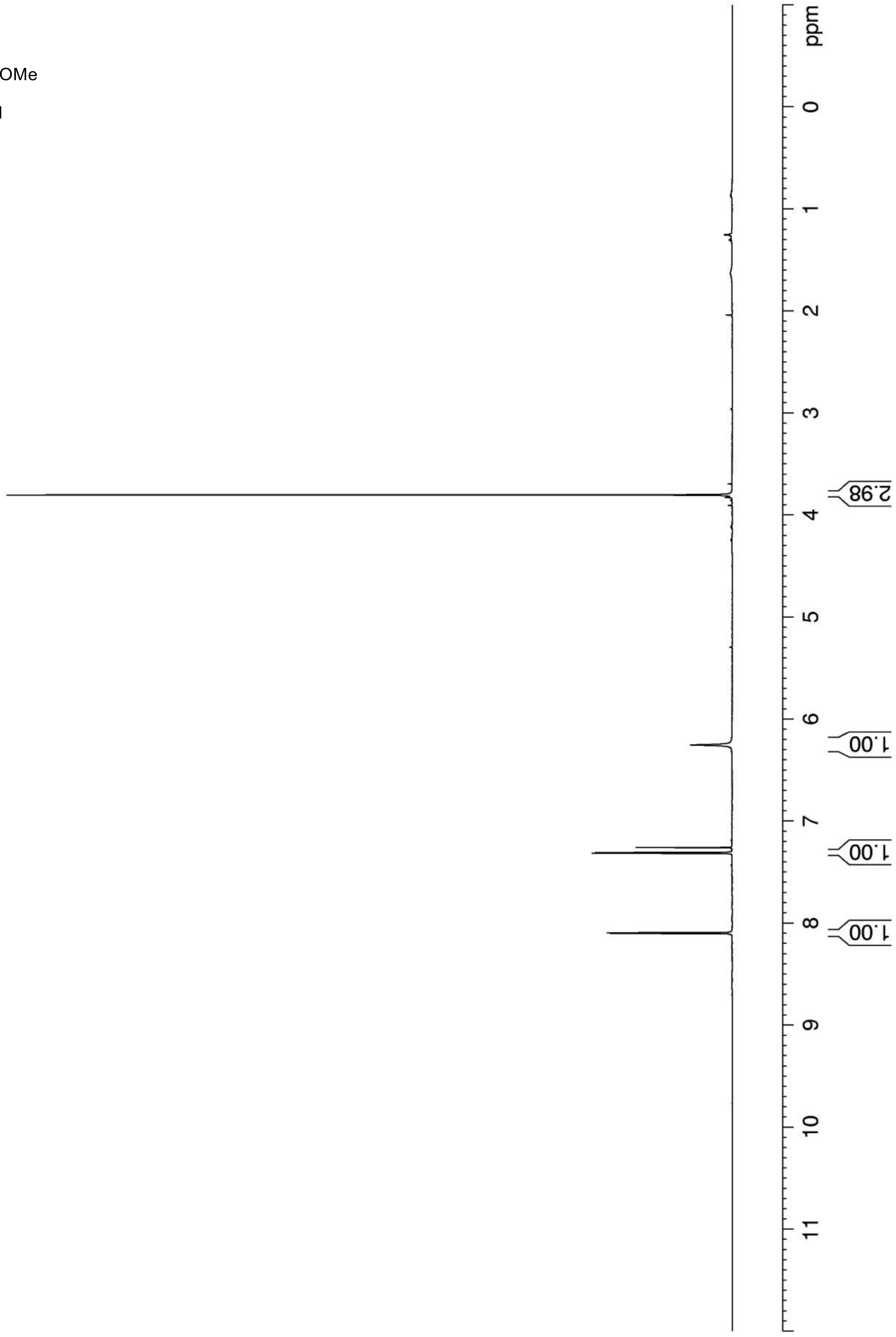

^{13}C NMR ($CDCl_3$, 25 ºC) of **2g-II**

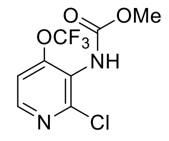

2g

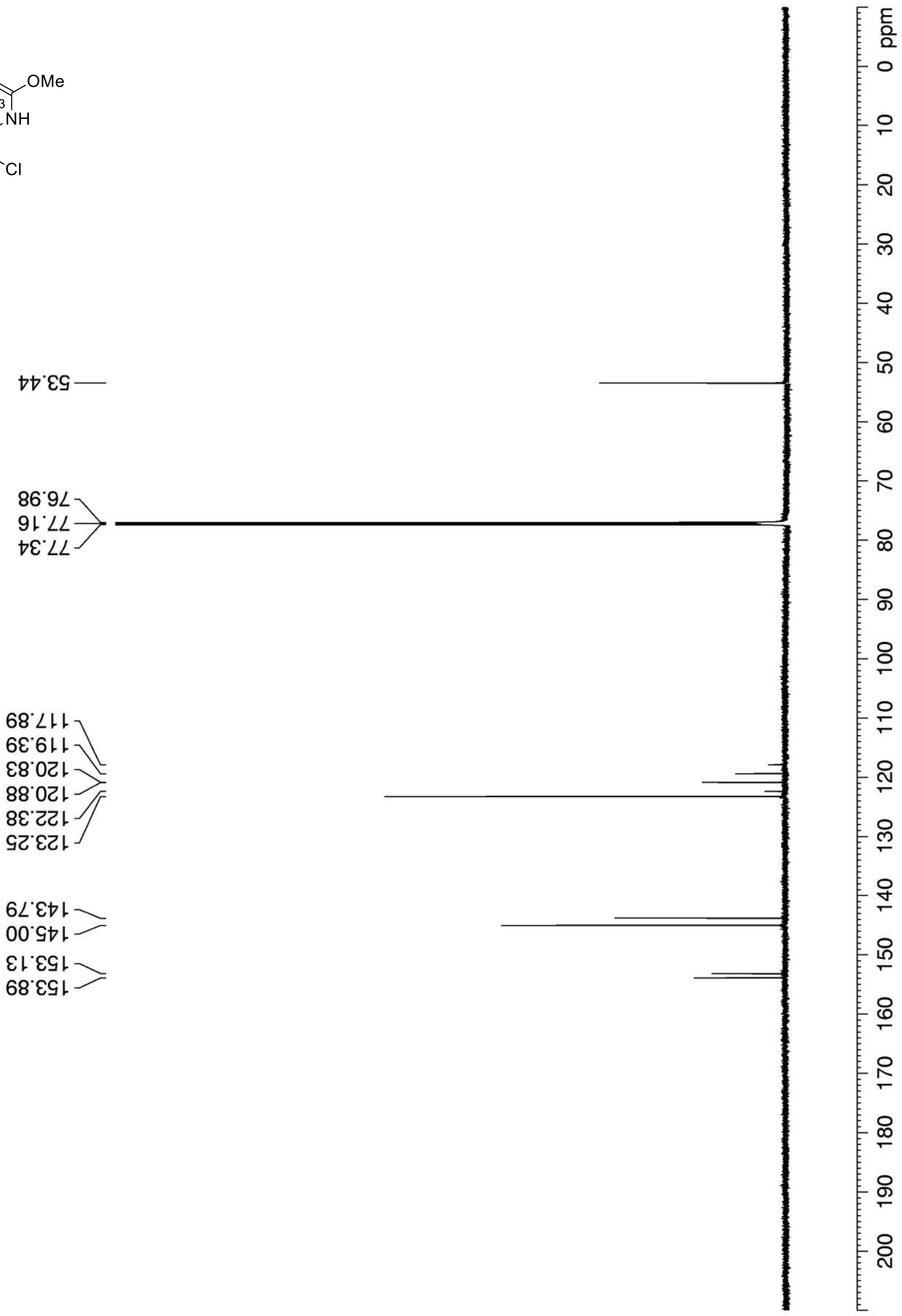

^{19}F NMR ($CDCl_3$, 25 ºC) of **2g-II**

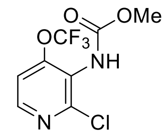

2g

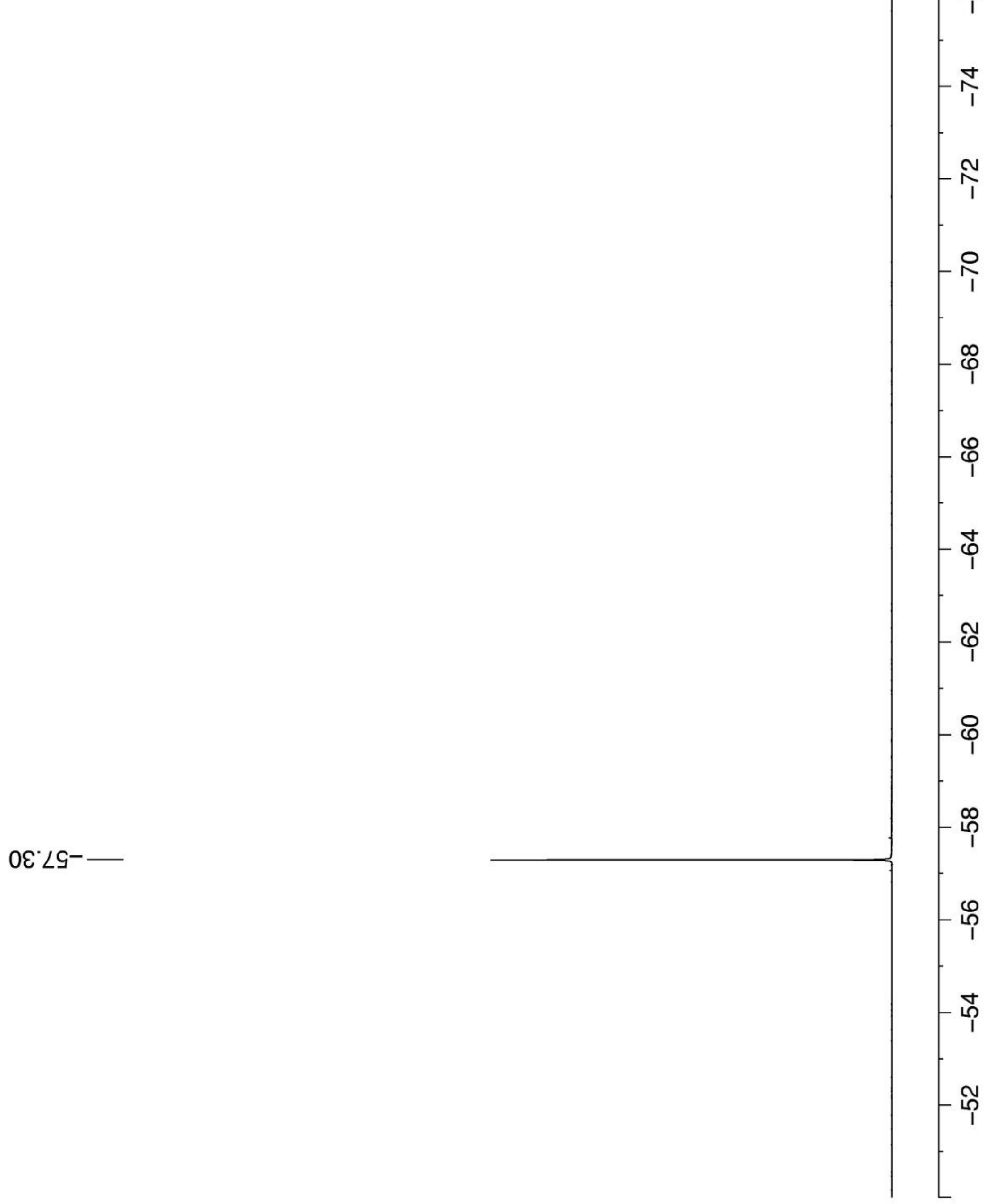

^{1}H NMR ($CDCl_3$, 25 ºC) of **2h**

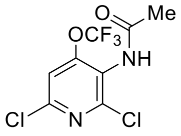

2h

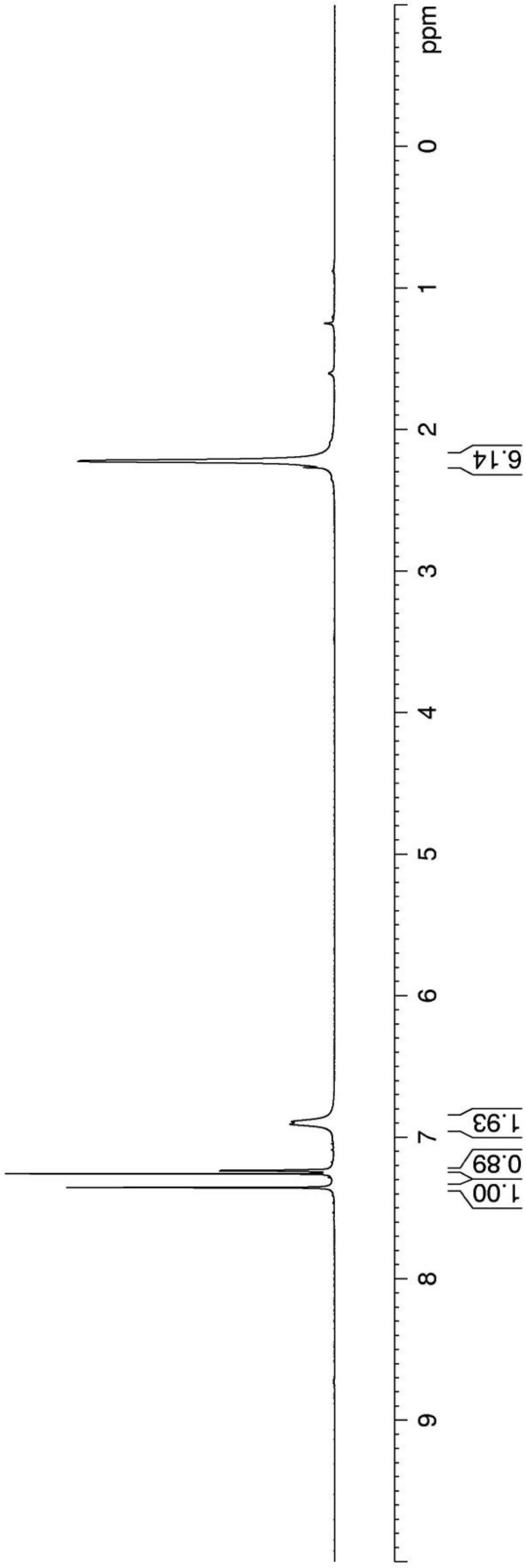

^{13}C NMR ($CDCl_3$, 25 ºC) of **2h**

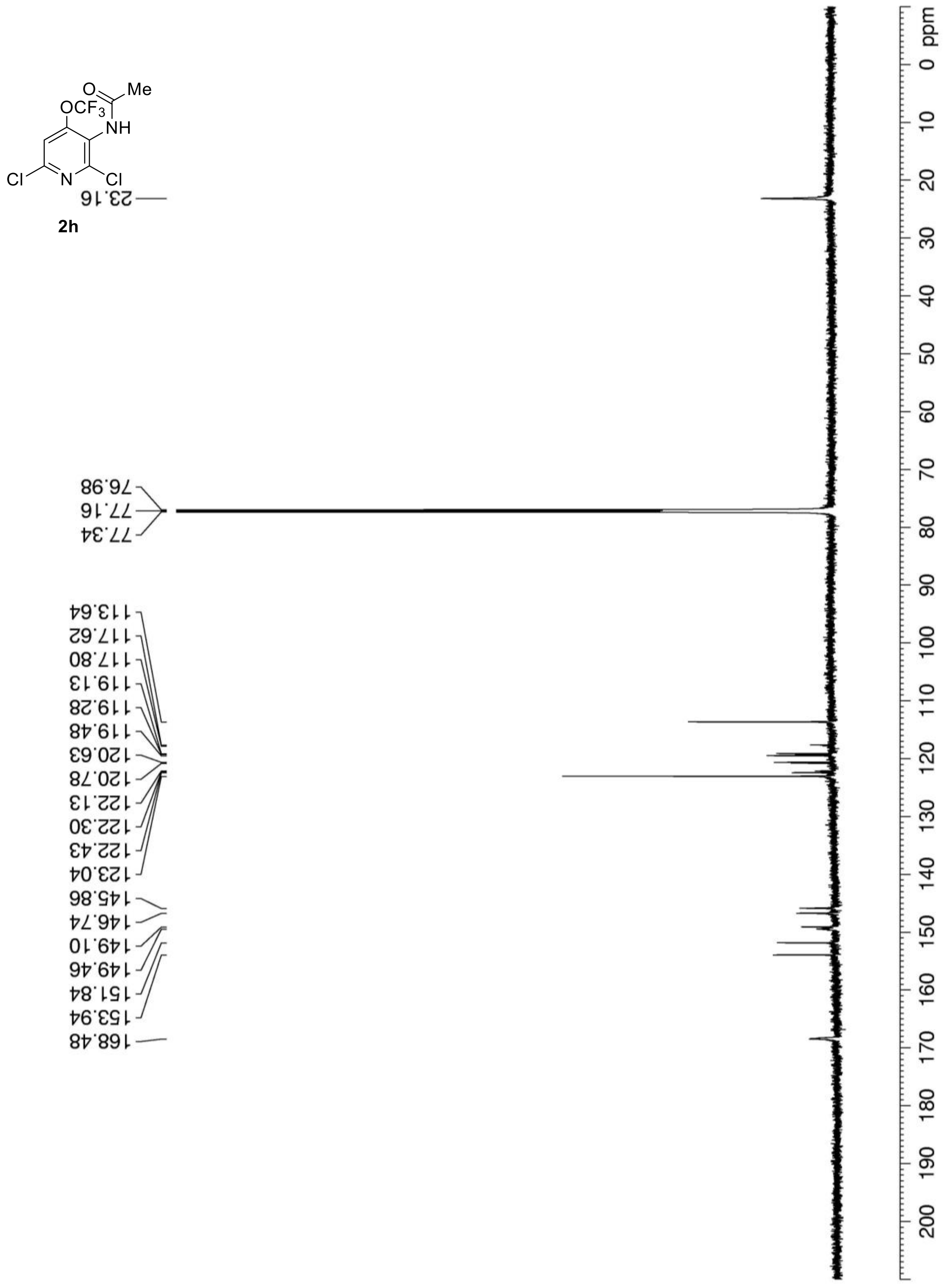

^{19}F NMR ($CDCl_3$, 25 ºC) of **2h**

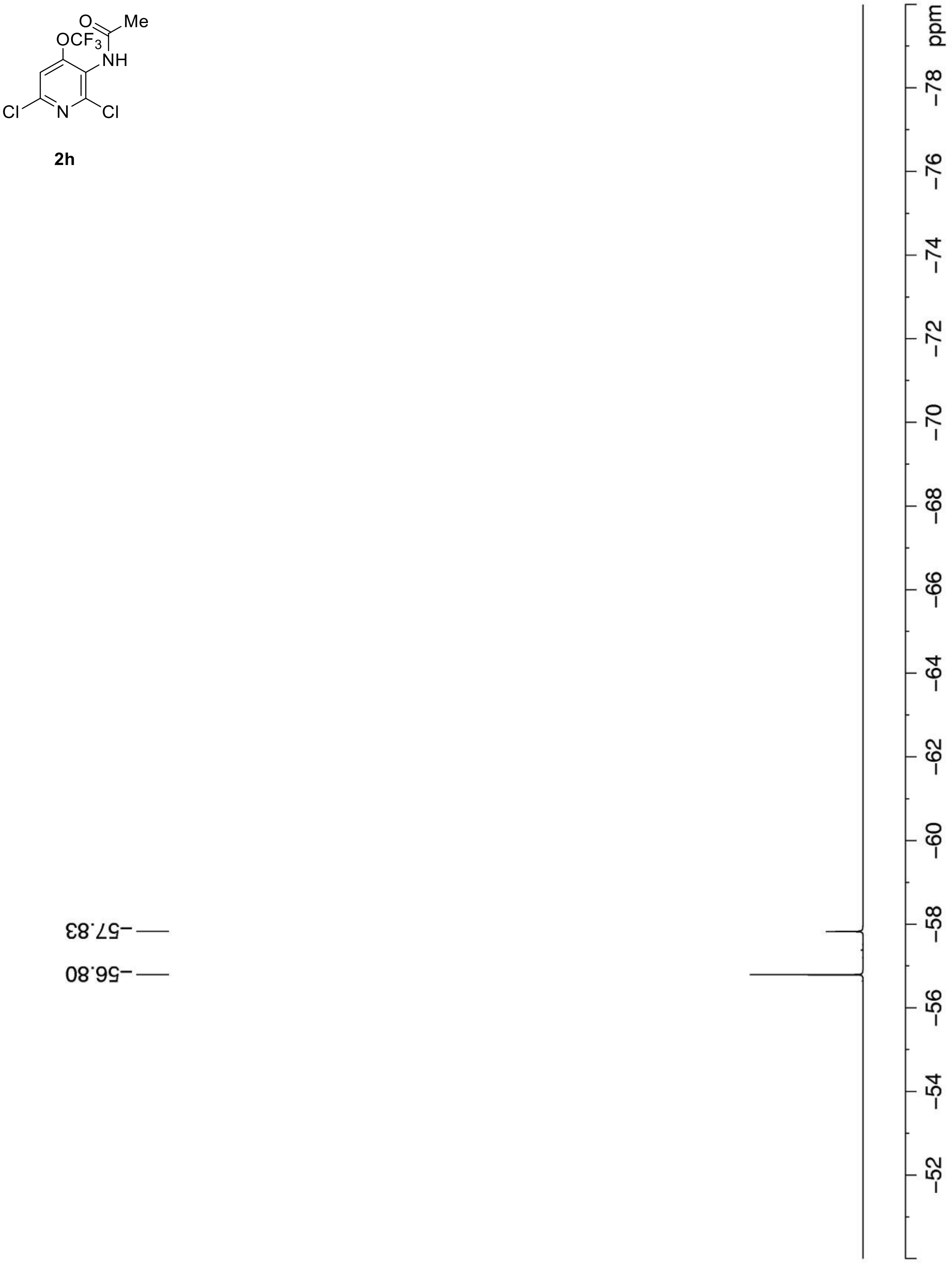
OCF3
O
Me
NH
Cl
N
Cl
-57.83
-56.80
ppm
-78
-76
-74
-72
-70
-68
-66
-64
-62
-60
-58
-56
-54
-52

2h

^{1}H NMR ($CDCl_3$, 60 ºC) of **2i**

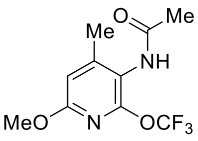

2i

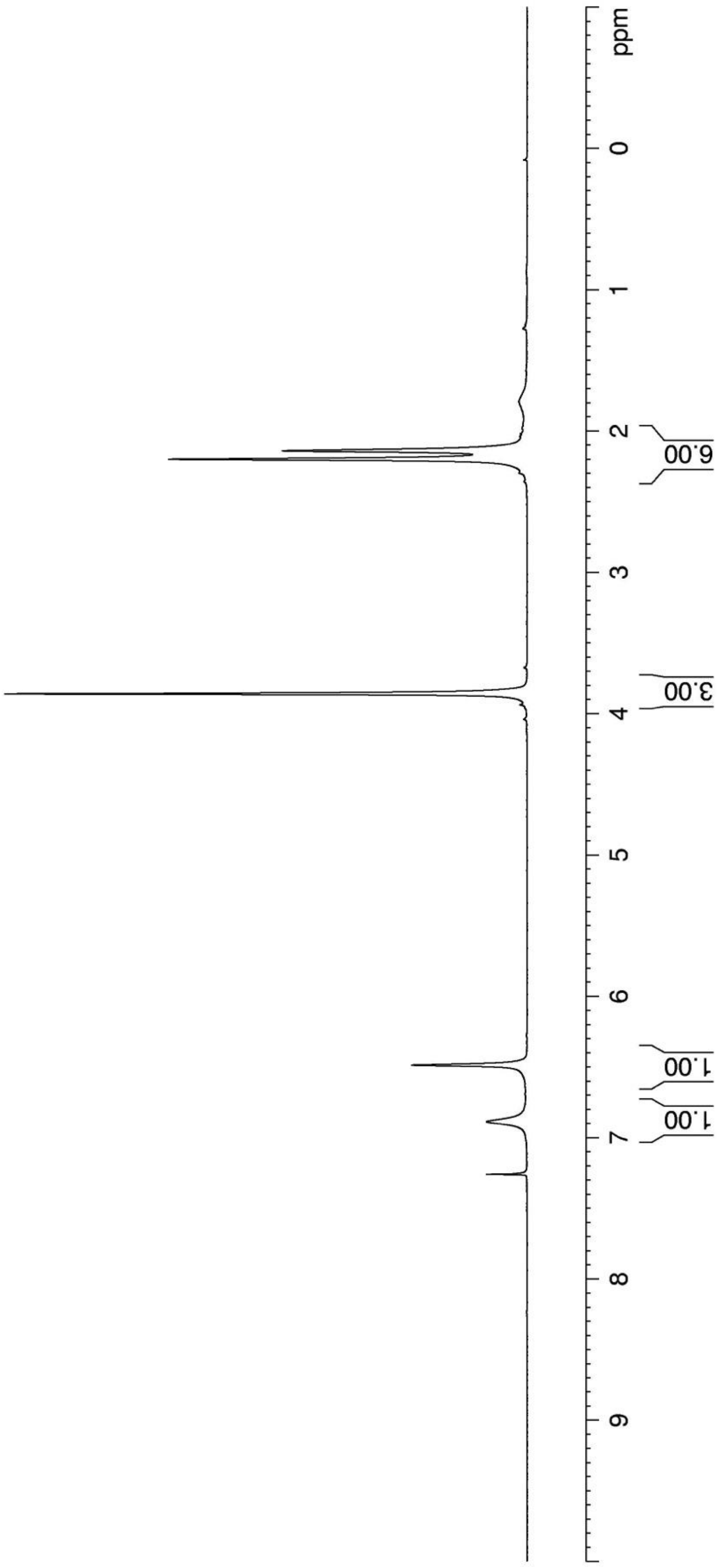

^{13}C NMR ($CDCl_3$, 60 ºC) of **2i**

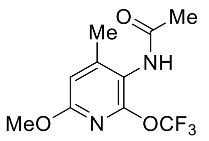

2i

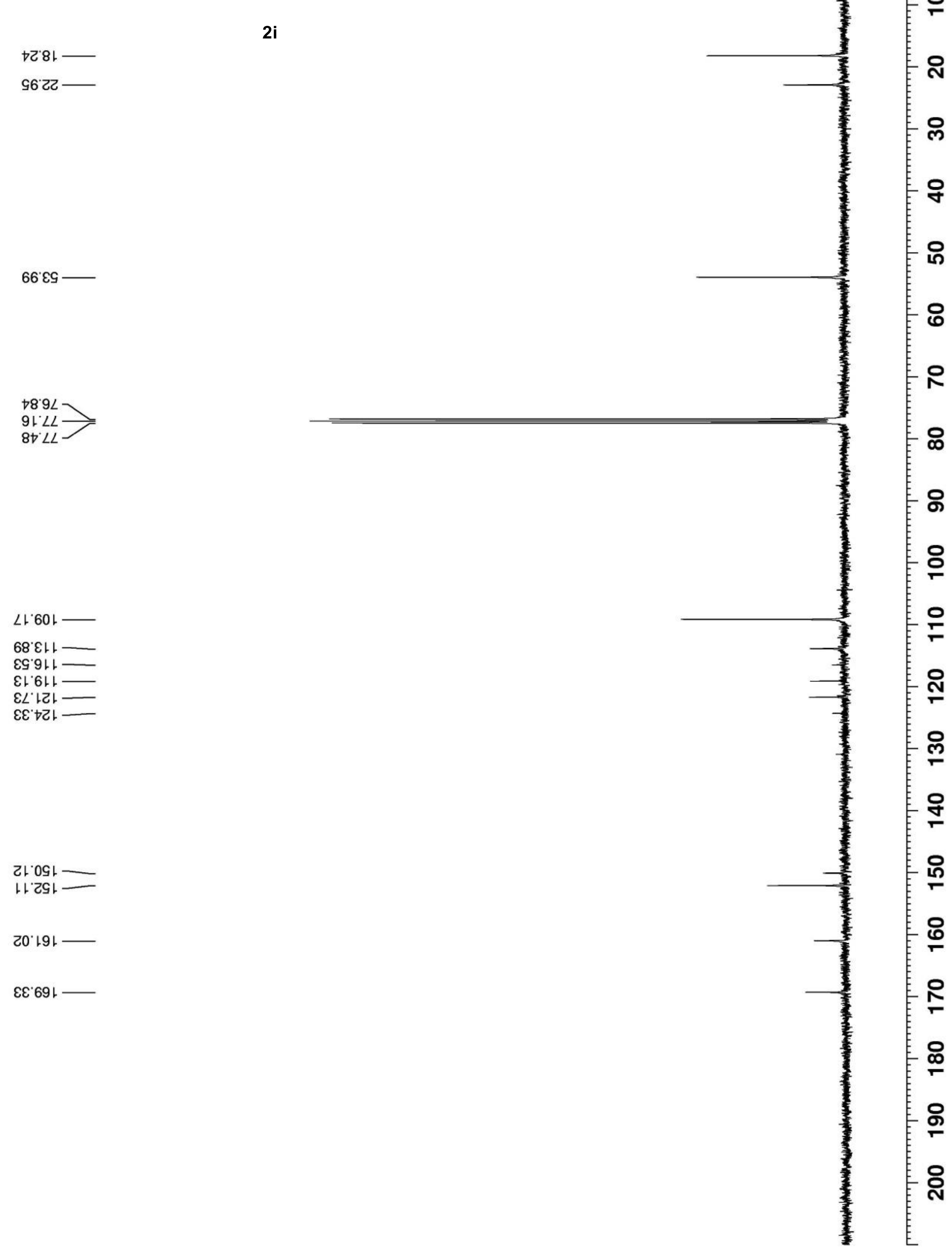

^{19}F NMR ($CDCl_3$, 60 ºC) of **2i**

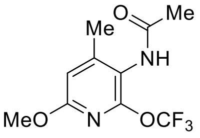

2i

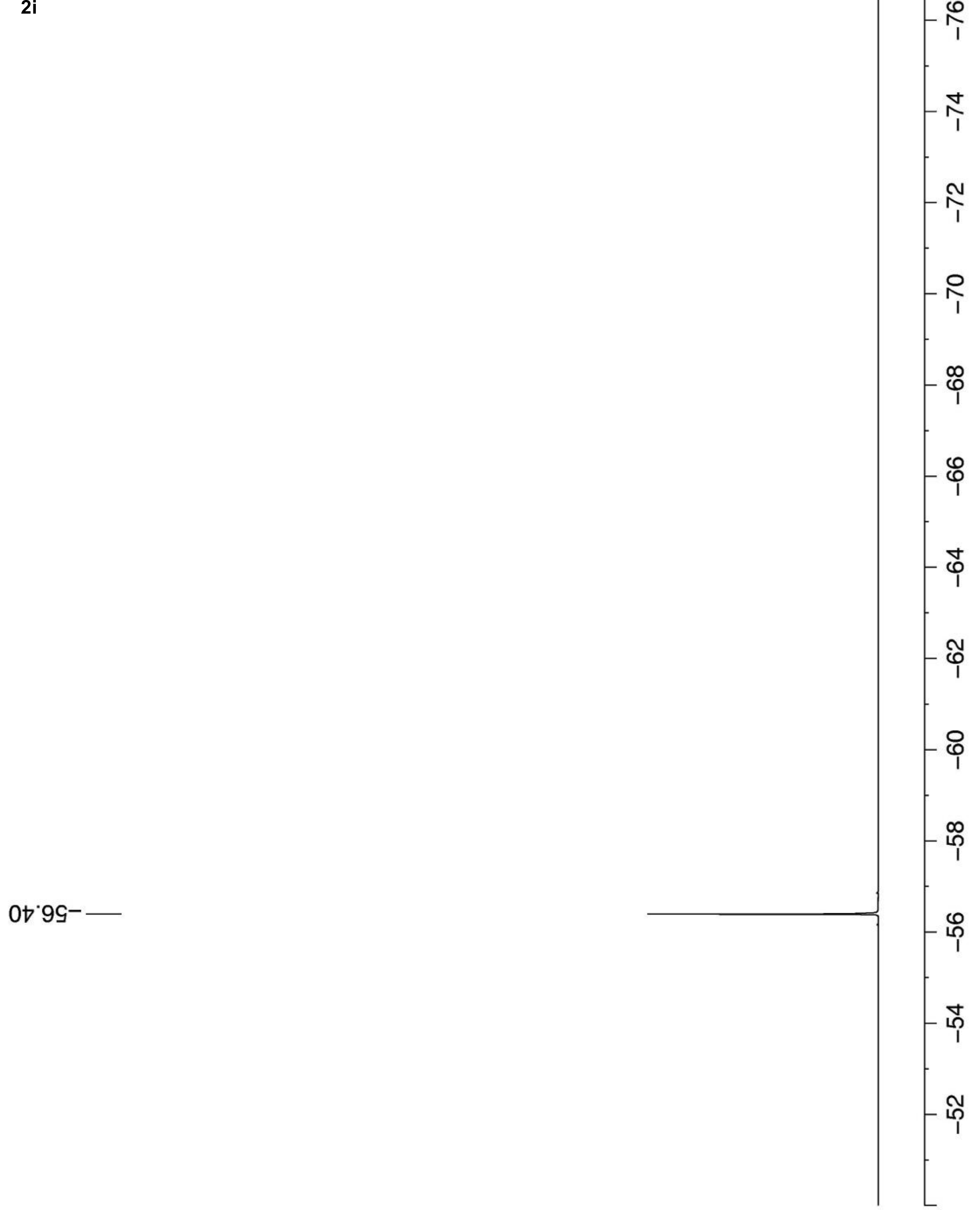

^{1}H NMR ($CDCl_3$, 25 ºC) of **2j**

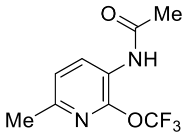

2j

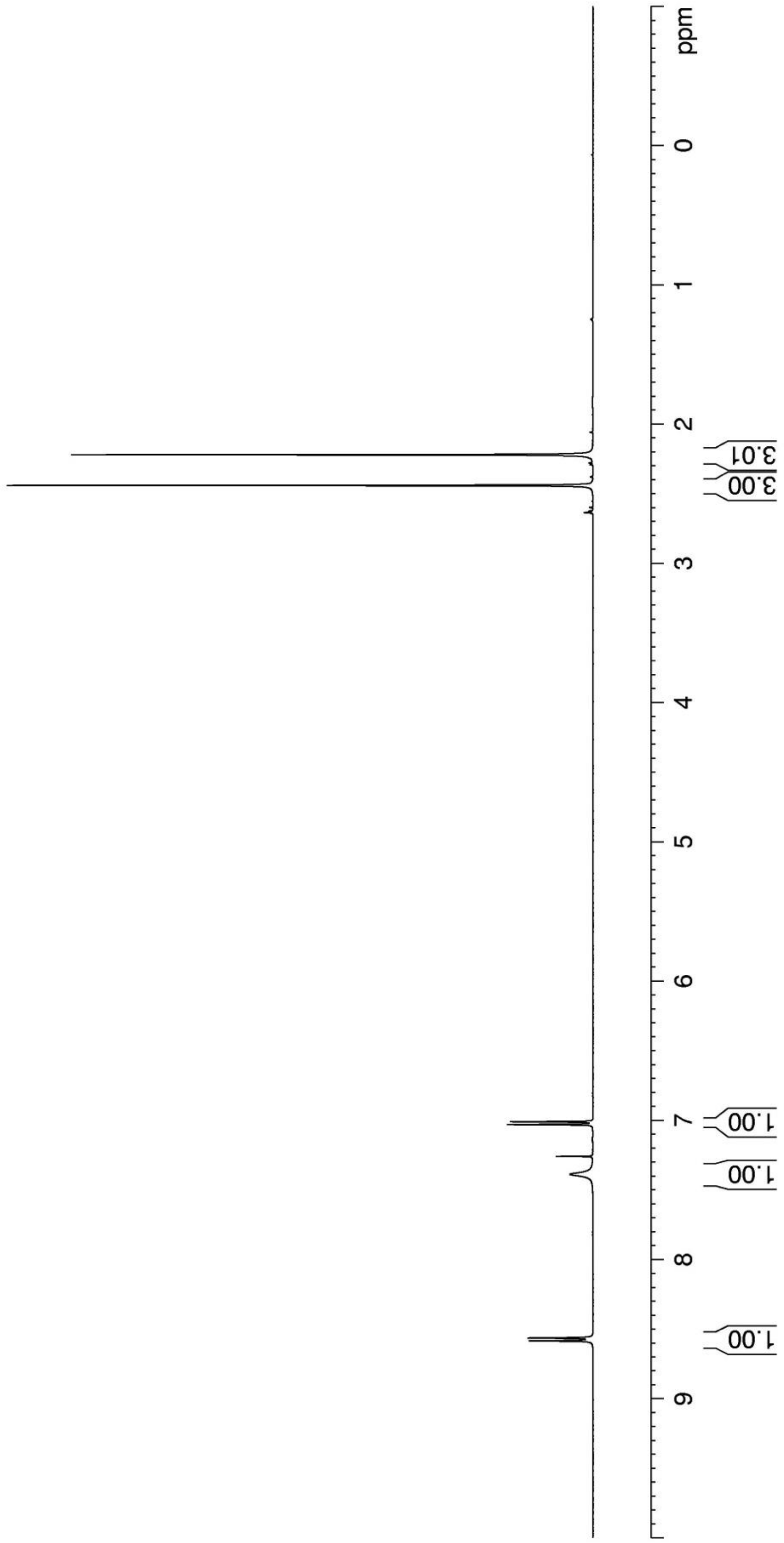

^{13}C NMR ($CDCl_3$, 25 ºC) of **2j**

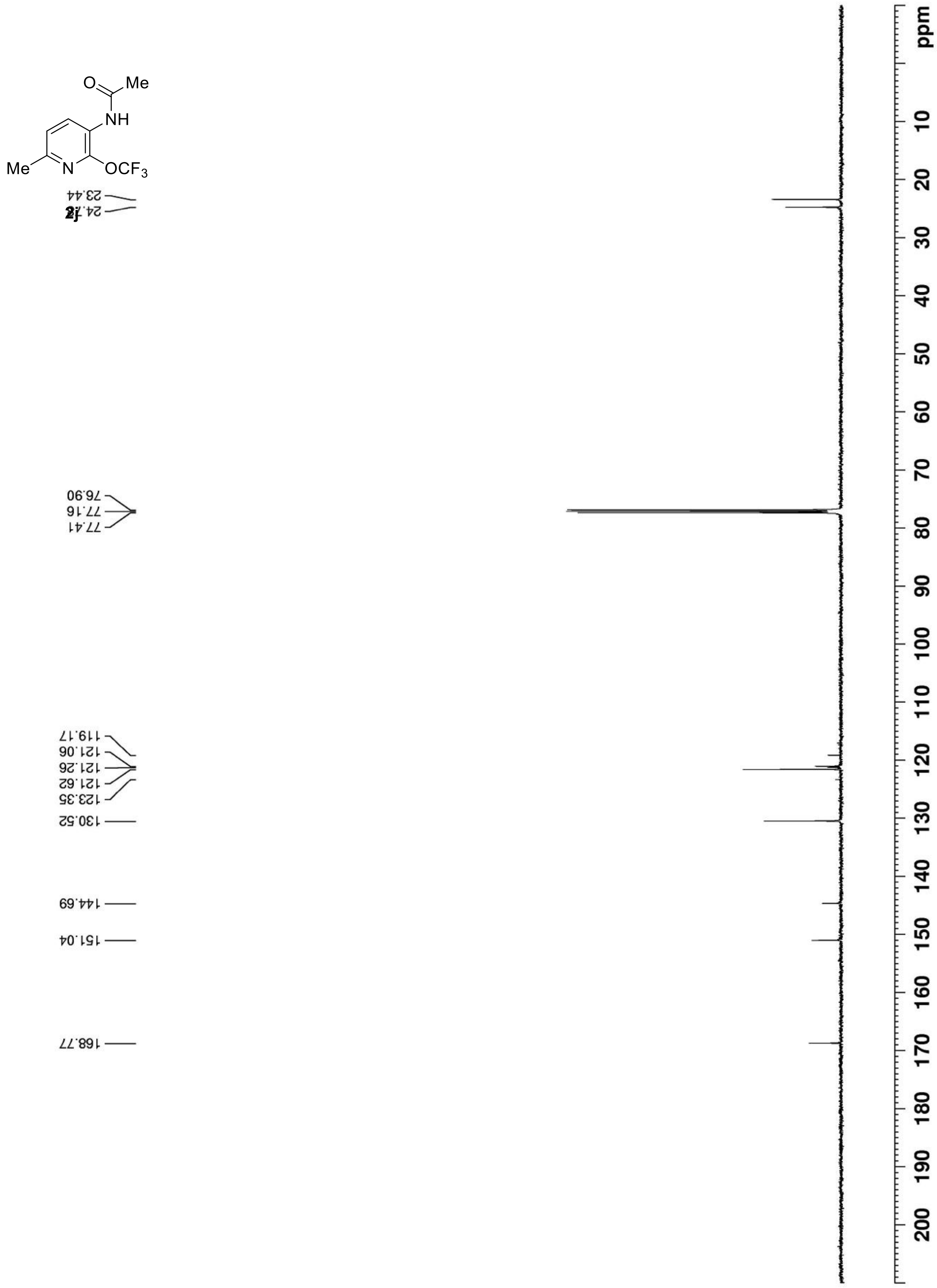

^{19}F NMR ($CDCl_3$, 25 ºC) of **2j**

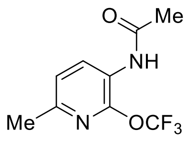

2j

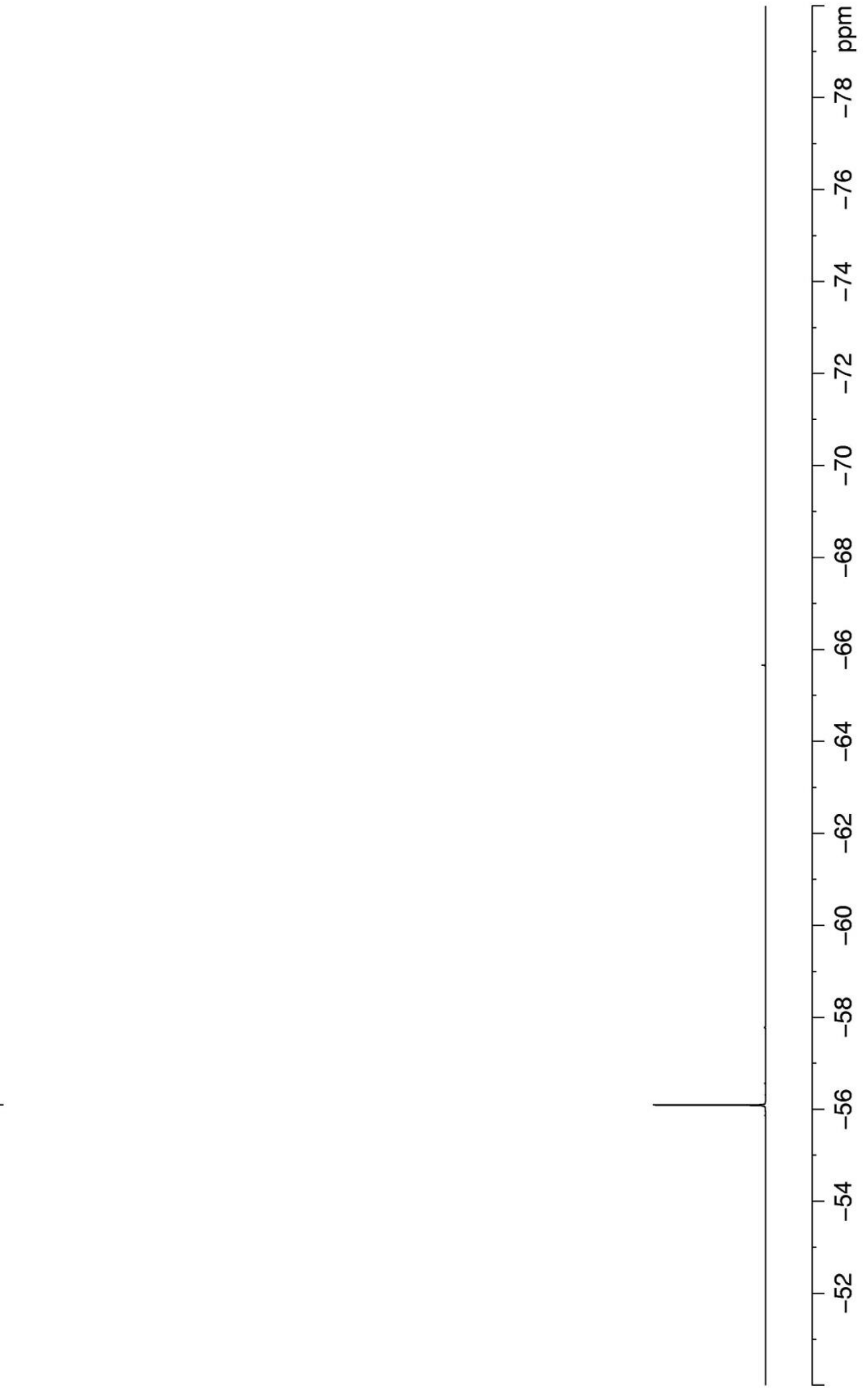

^{1}H NMR ($CDCl_3$, 25 ºC) of **2k**

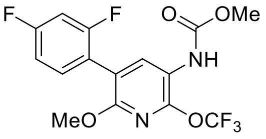

2k

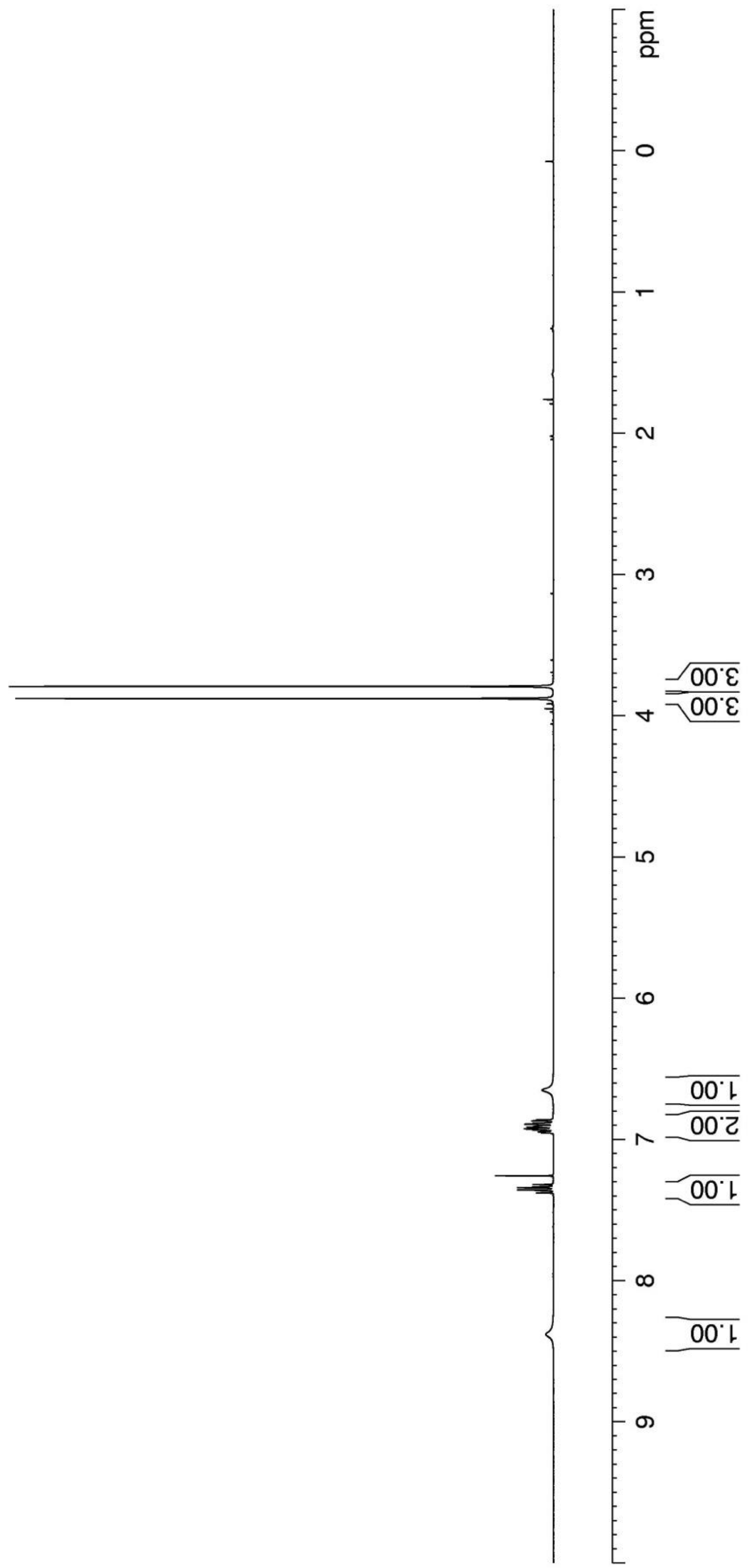

^{13}C NMR ($CDCl_3$, 25 ºC) of **2k**

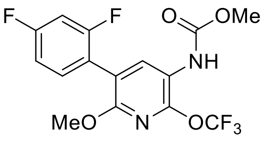

2k

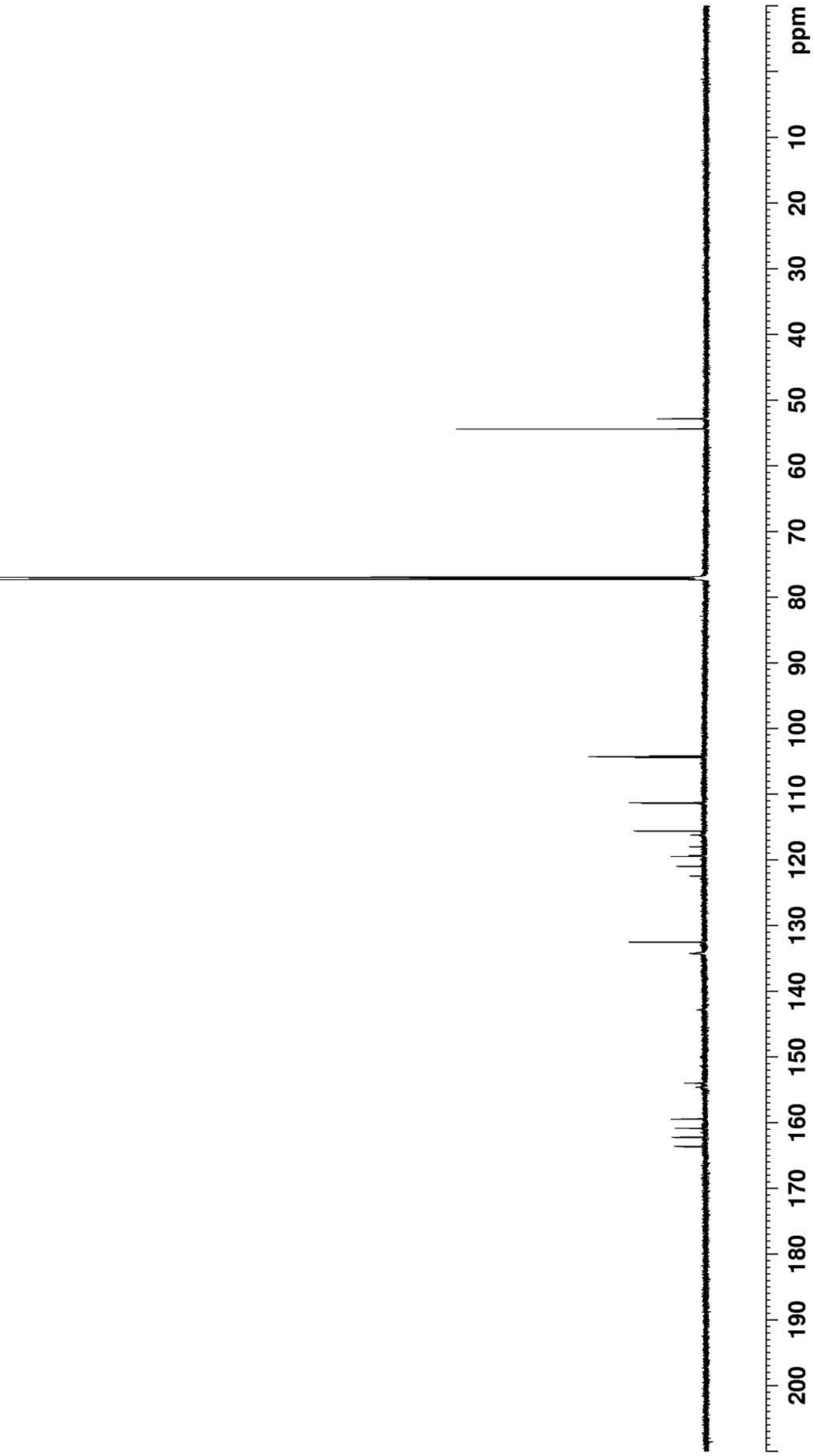

^{19}F NMR ($CDCl_3$, 25 ºC) of **2k**

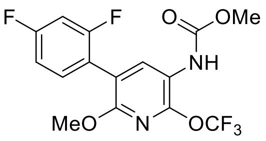

2k

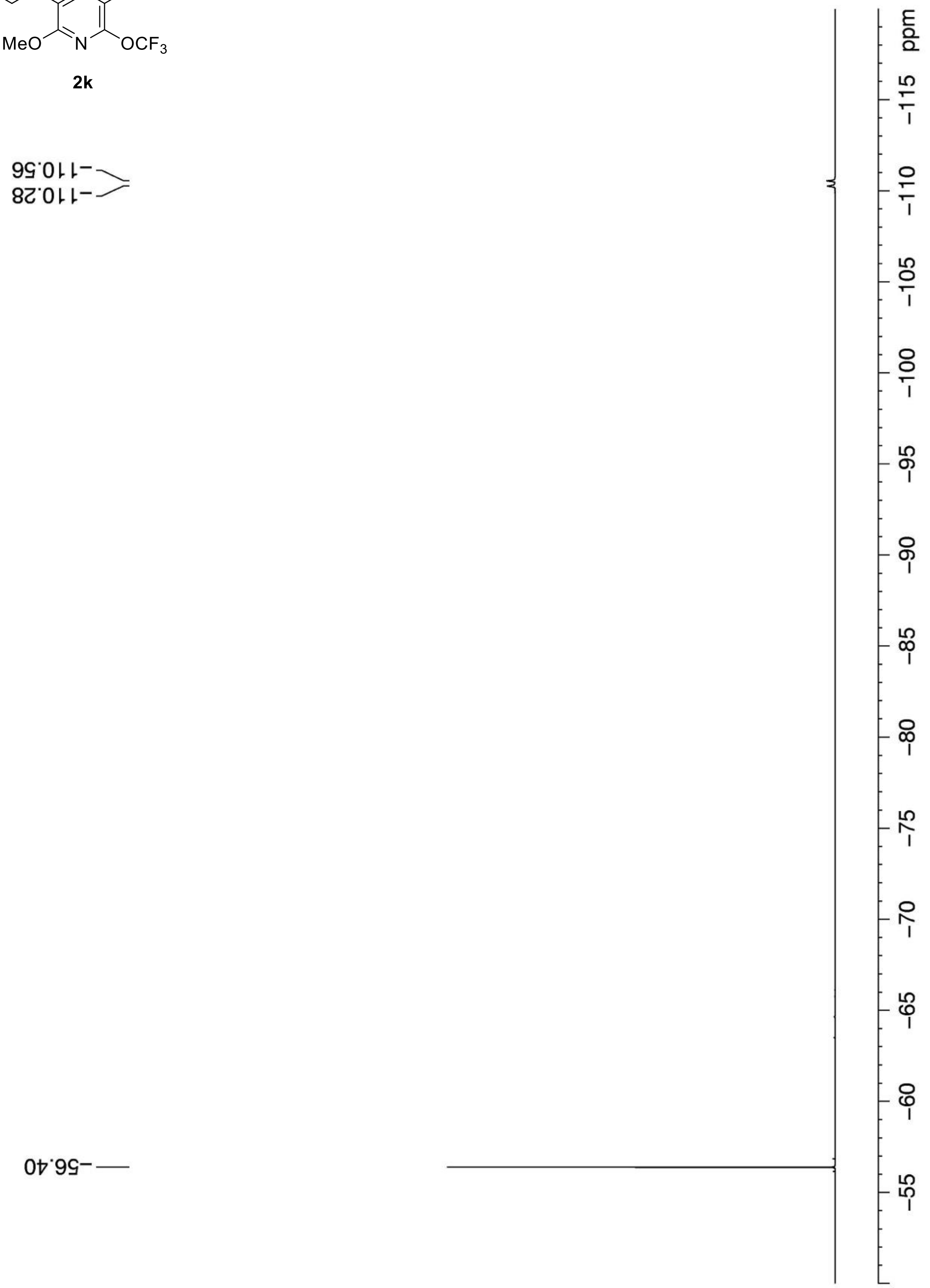

^{1}H NMR ($CDCl_3$, 25 ºC) of **2l**

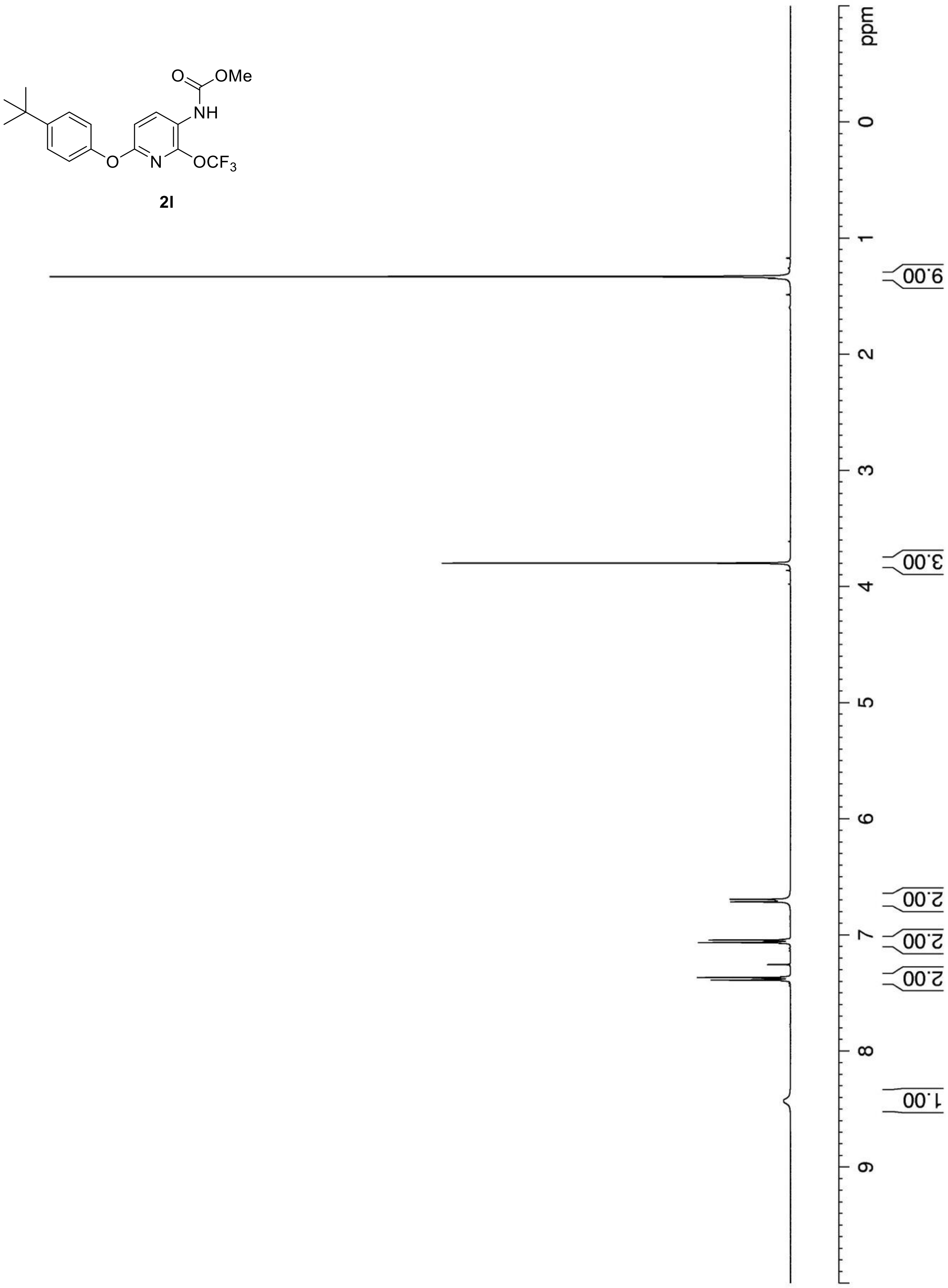

^{13}C NMR ($CDCl_3$, 25 ºC) of **2l**

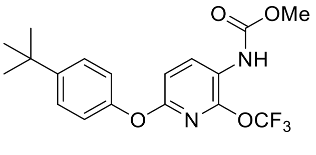

2l

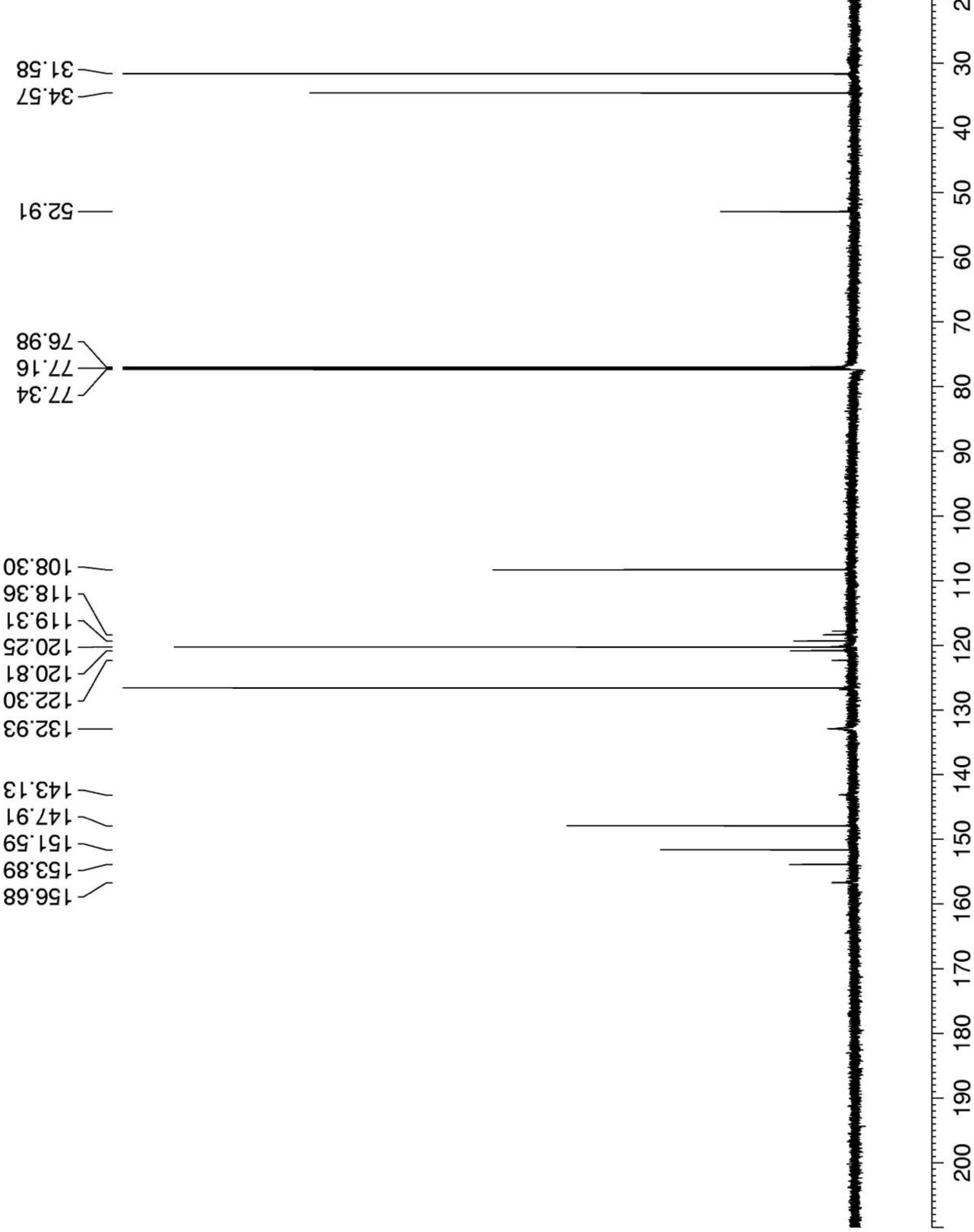

^{19}F NMR ($CDCl_3$, 25 ºC) of **2l**

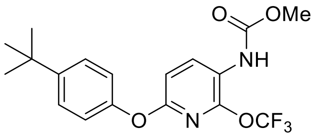

2l

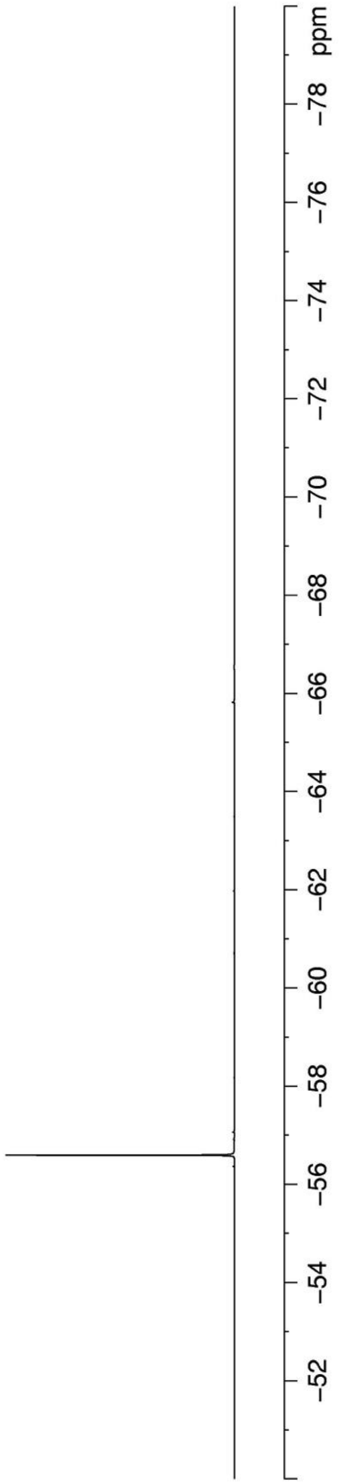

^{1}H NMR ($CDCl_3$, 25 ºC) of **2m**

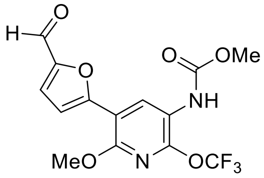

2m

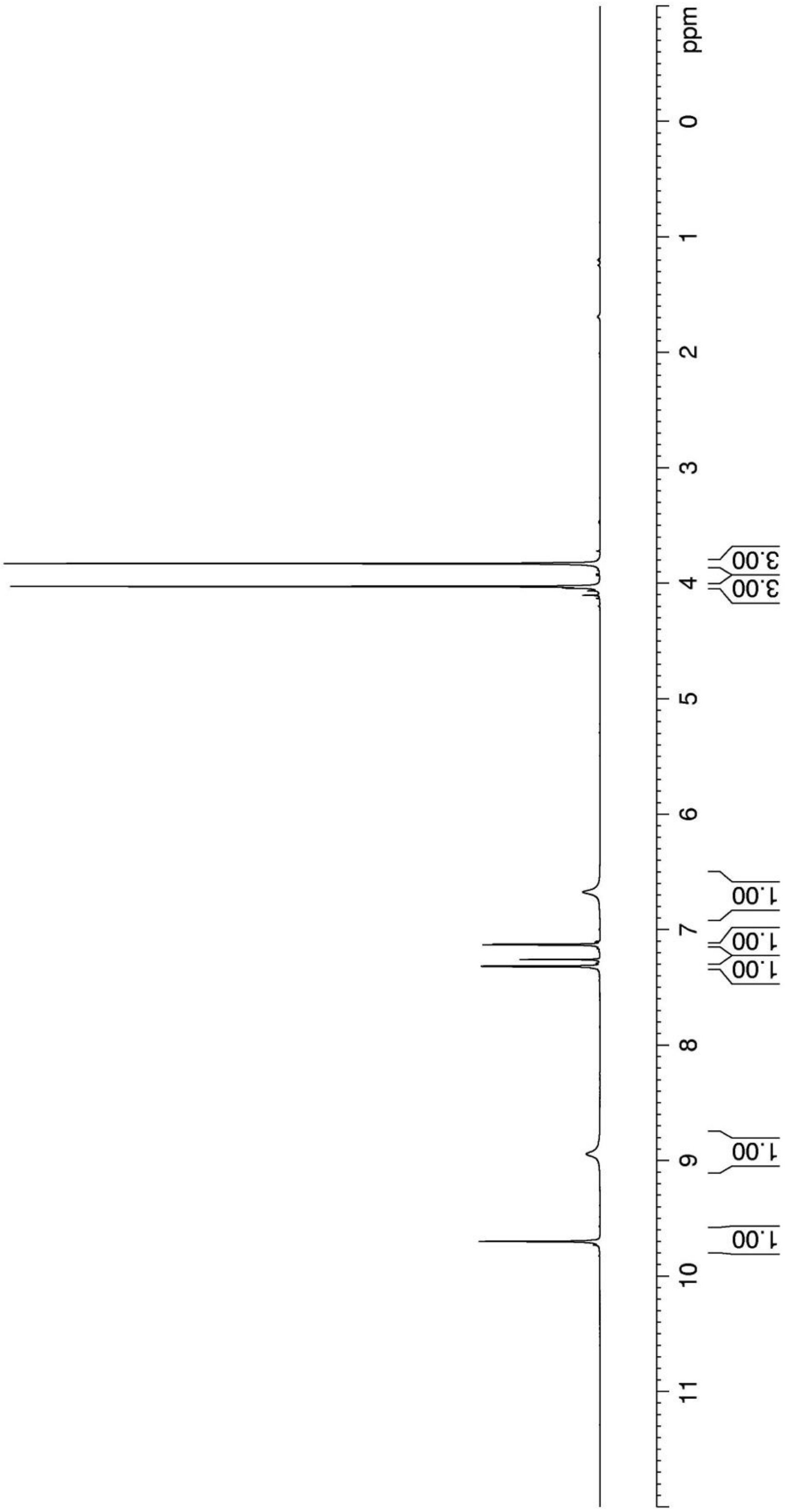

^{13}C NMR ($CDCl_3$, 25 ºC) of **2m**

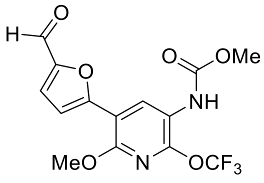

2m

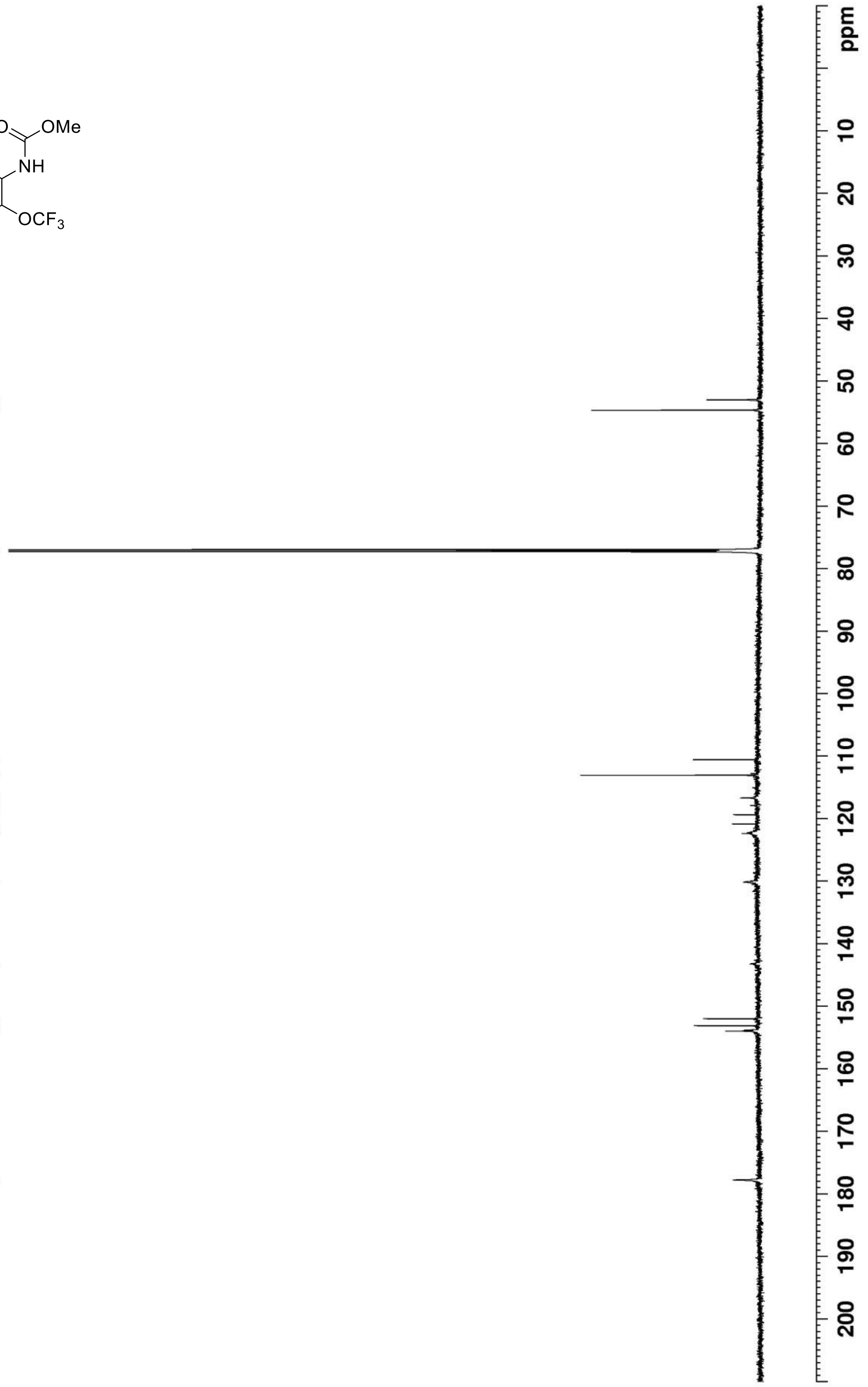

^{19}F NMR ($CDCl_3$, 25 ºC) of **2m**

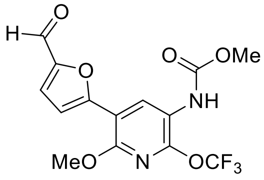

2m

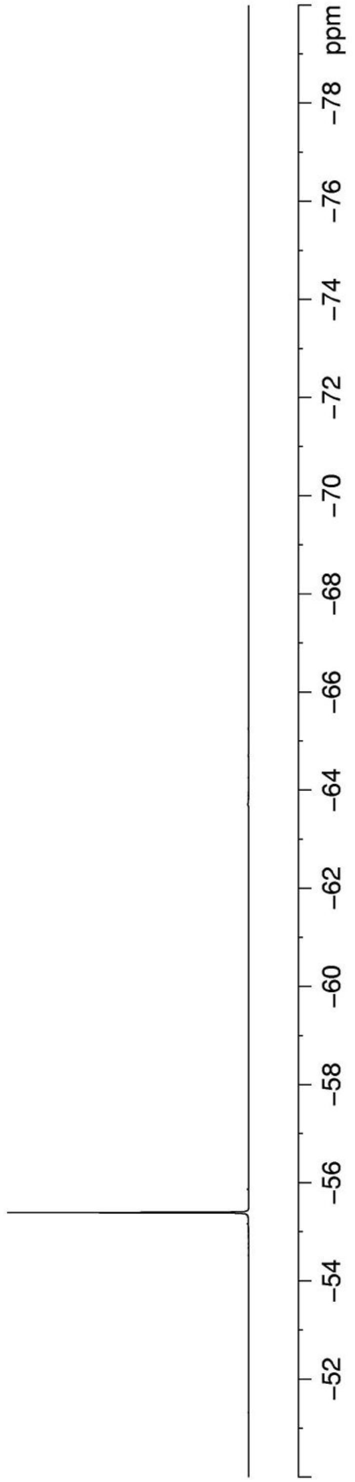

^{1}H NMR ($CDCl_3$, 25 ºC) of **2n**

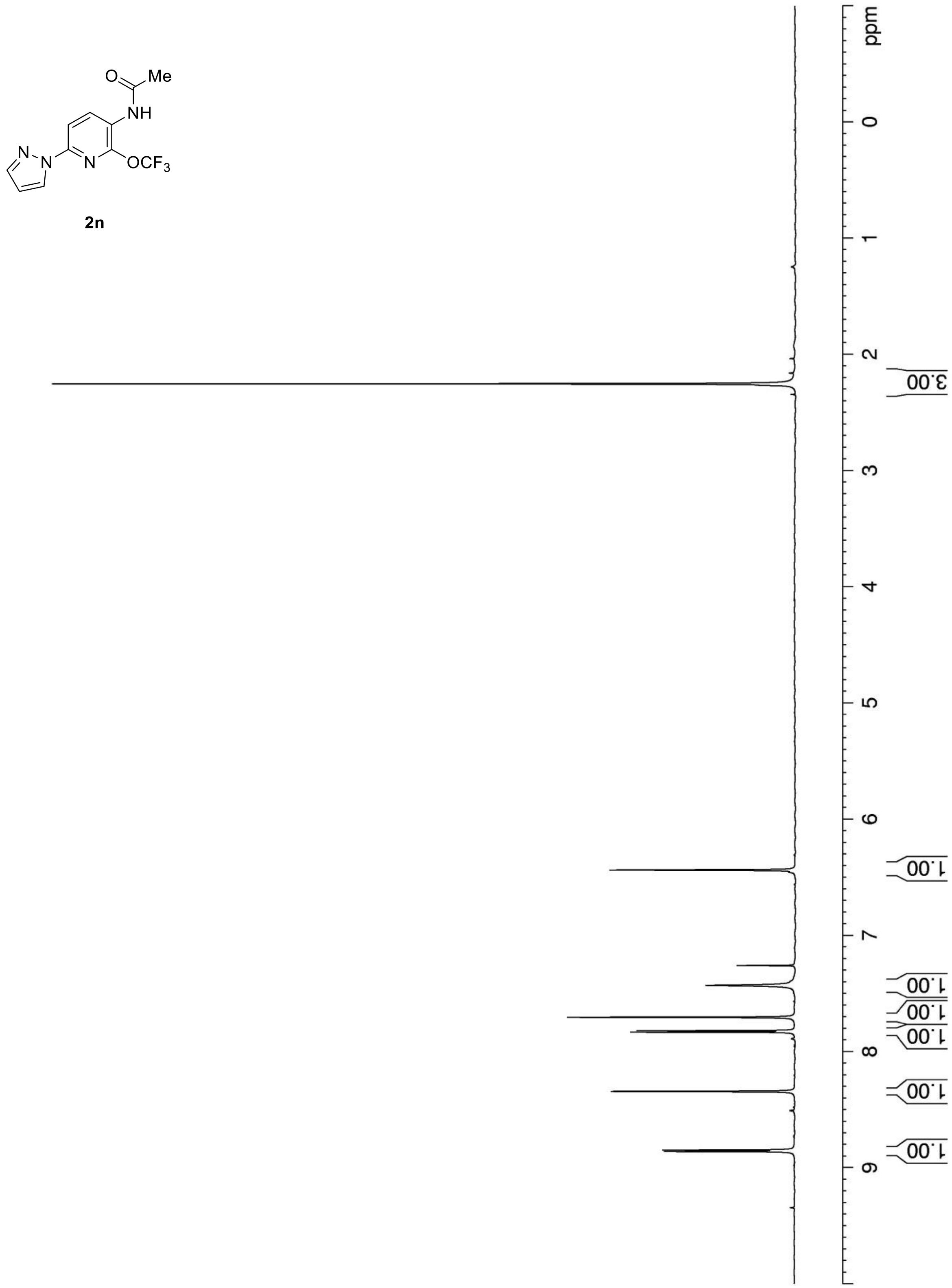

^{13}C NMR ($CDCl_3$, 25 ºC) of **2n**

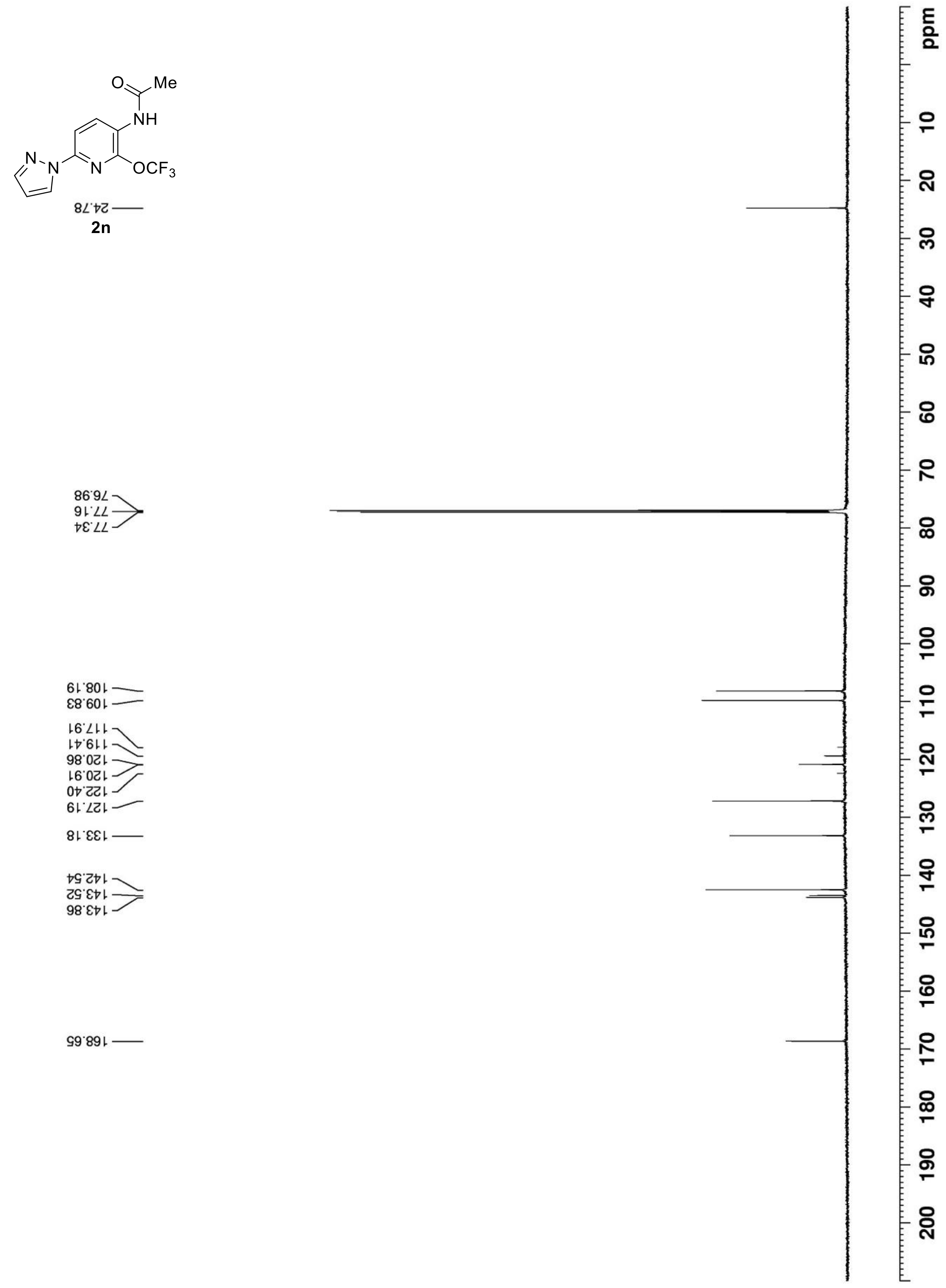

2n

^{19}F NMR ($CDCl_3$, 25 ºC) of **2n**

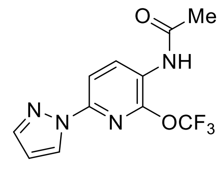

2n

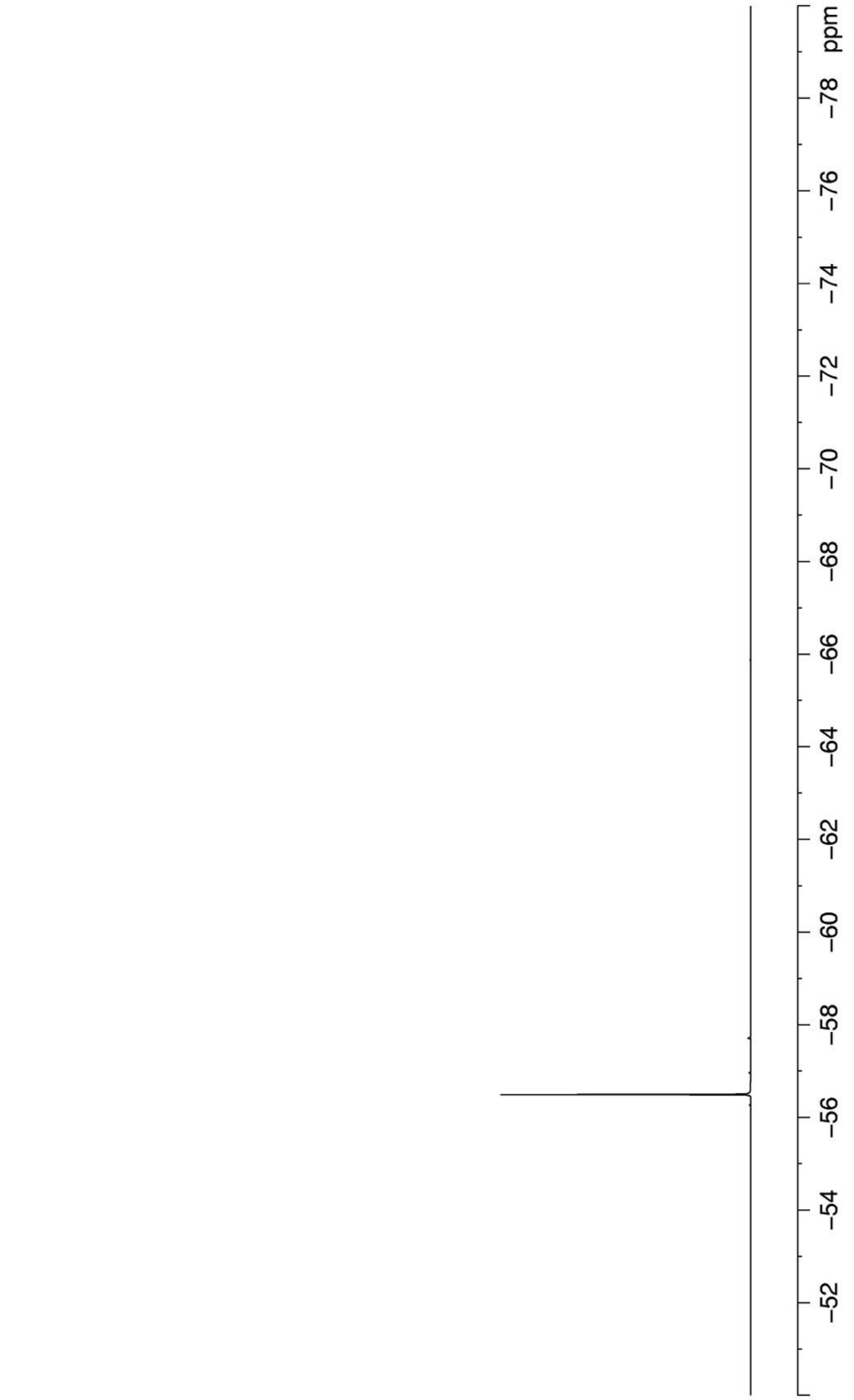

^{1}H NMR ($CDCl_3$, 25 ºC) of **2o**

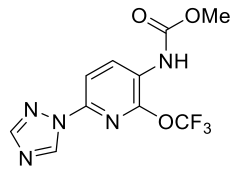

2o

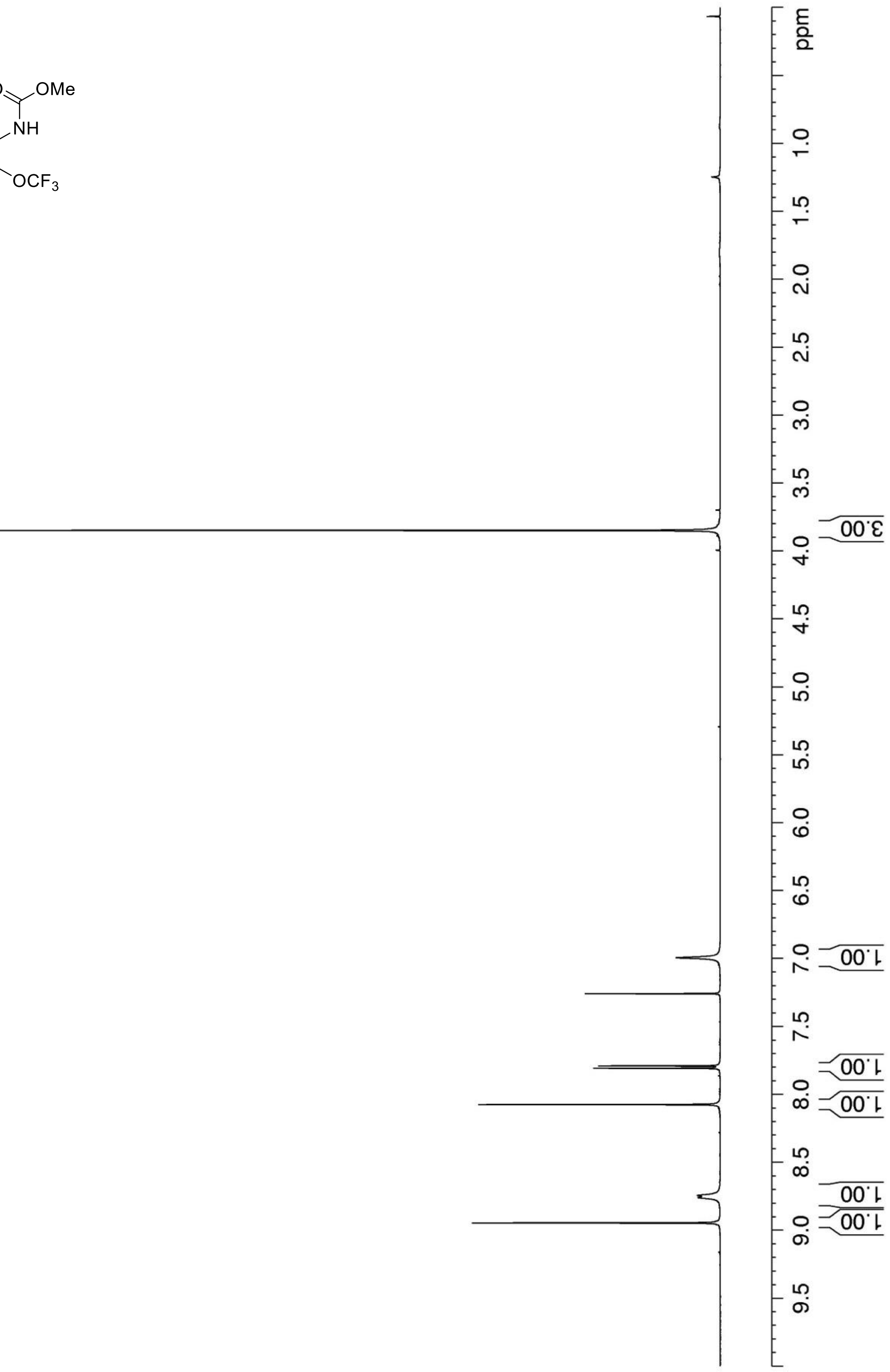

^{13}C NMR ($CDCl_3$, 25 ºC) of **2o**

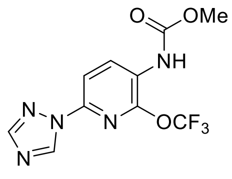

2o

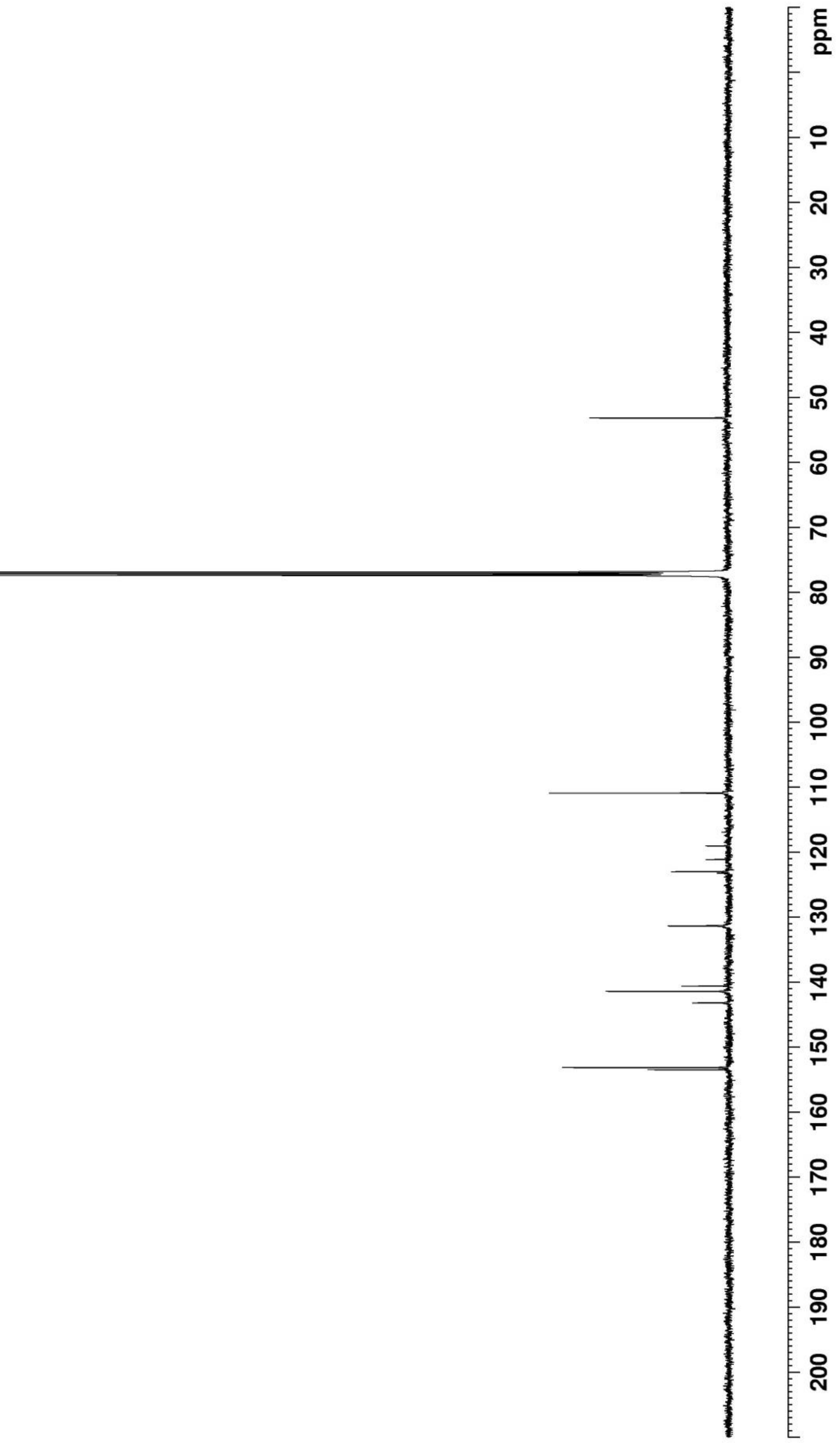

^{19}F NMR ($CDCl_3$, 25 ºC) of **2o**

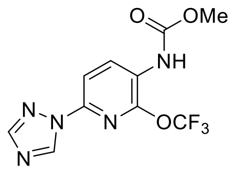

2o

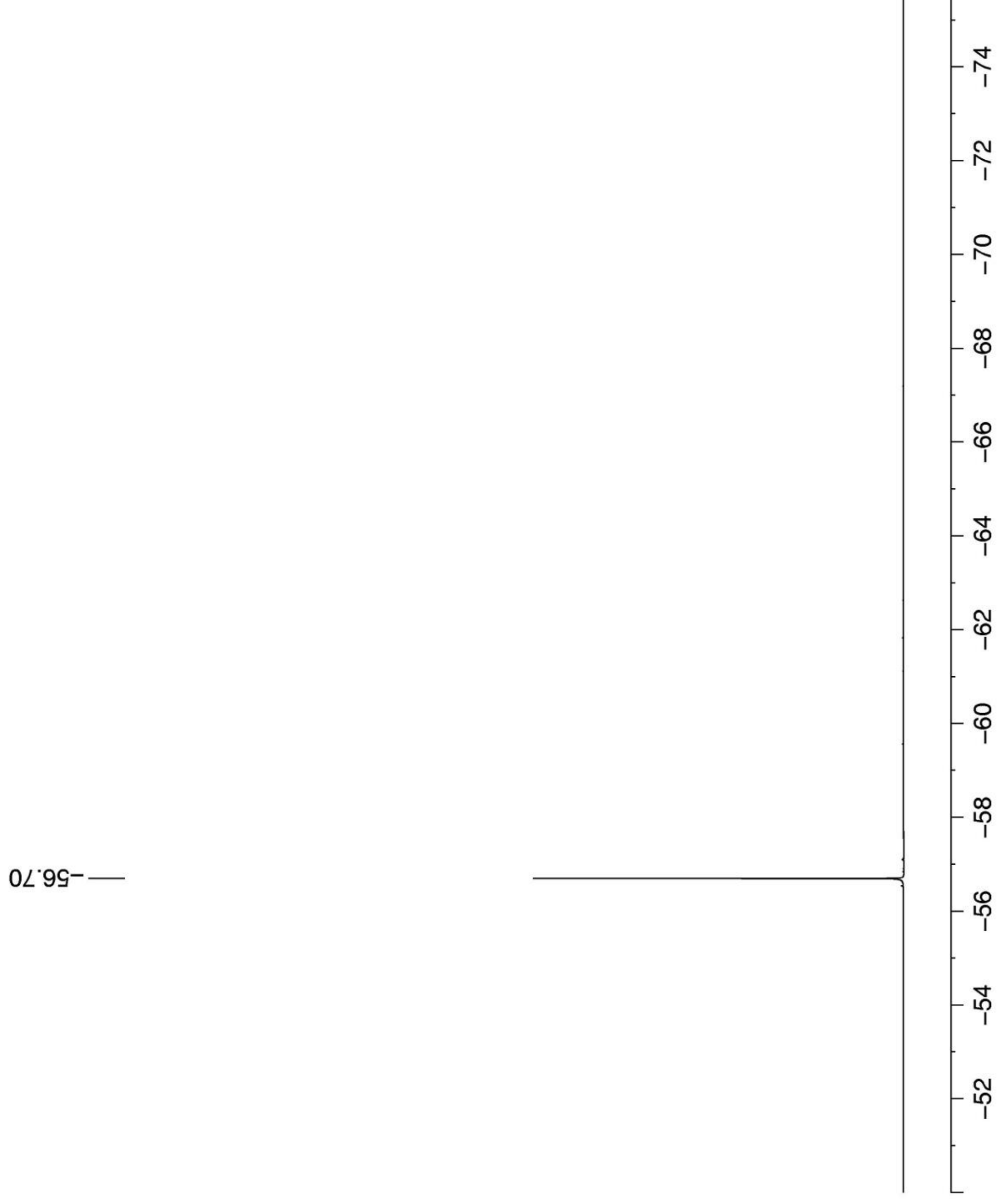

^{1}H NMR ($CDCl_3$, 25 ºC) of **2o-II**

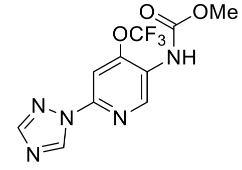

2o-II

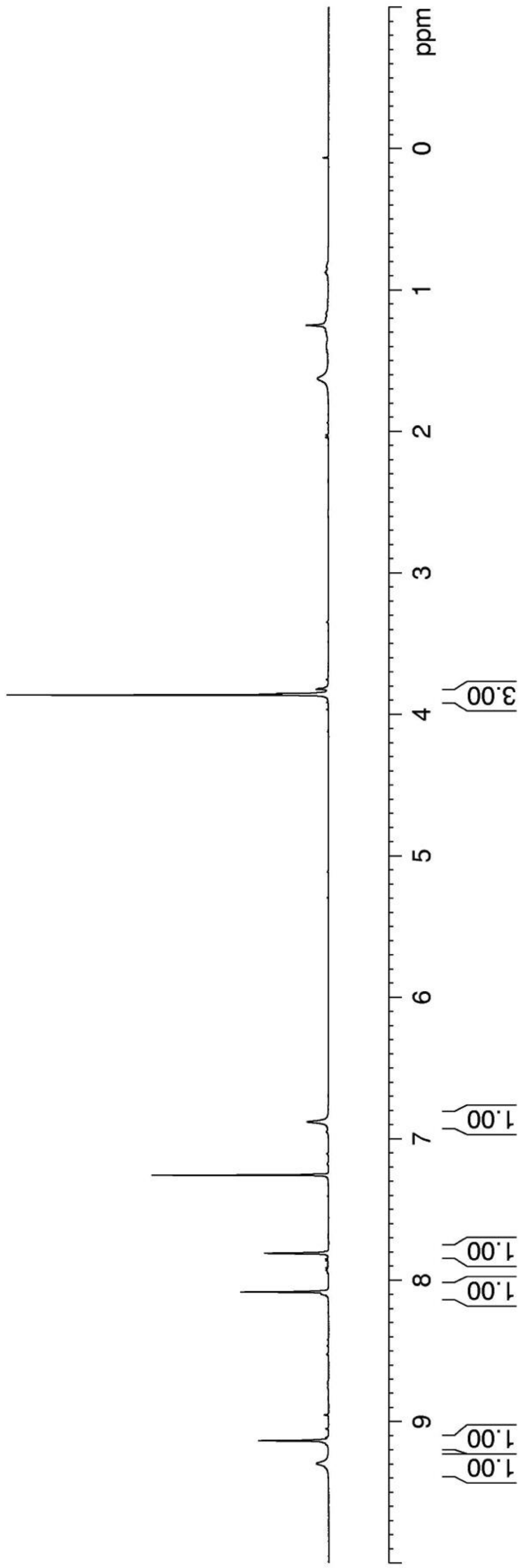

^{13}C NMR ($CDCl_3$, 25 ºC) of **2o-II**

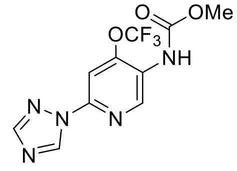

2o-II

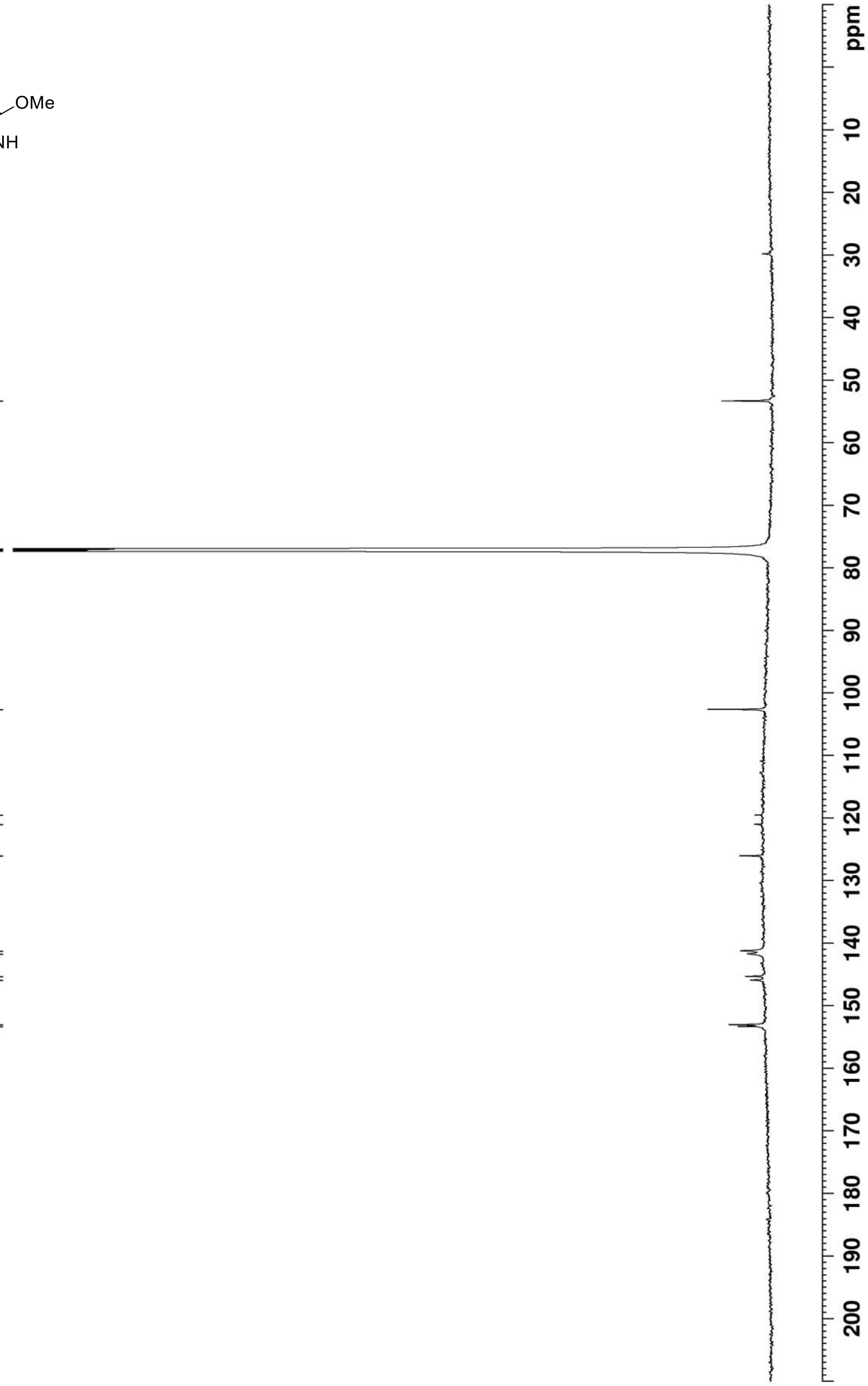

^{19}F NMR ($CDCl_3$, 25 ºC) of **2o-II**

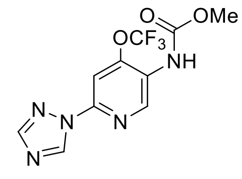

2o-II

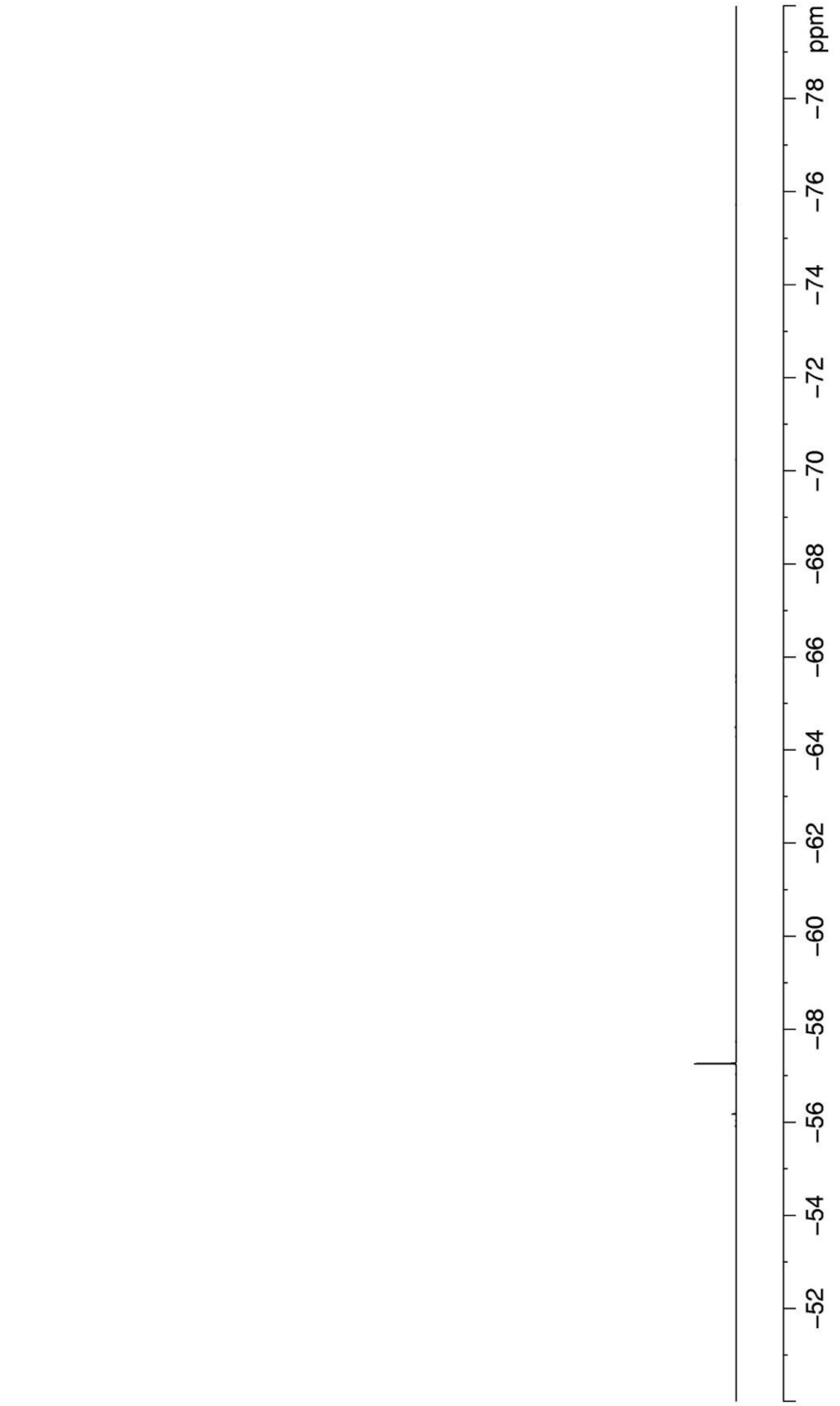

^{1}H NMR ($CDCl_3$, 25 ºC) of **2p**

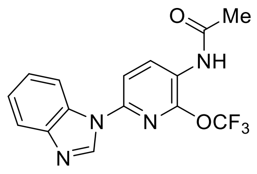

2p

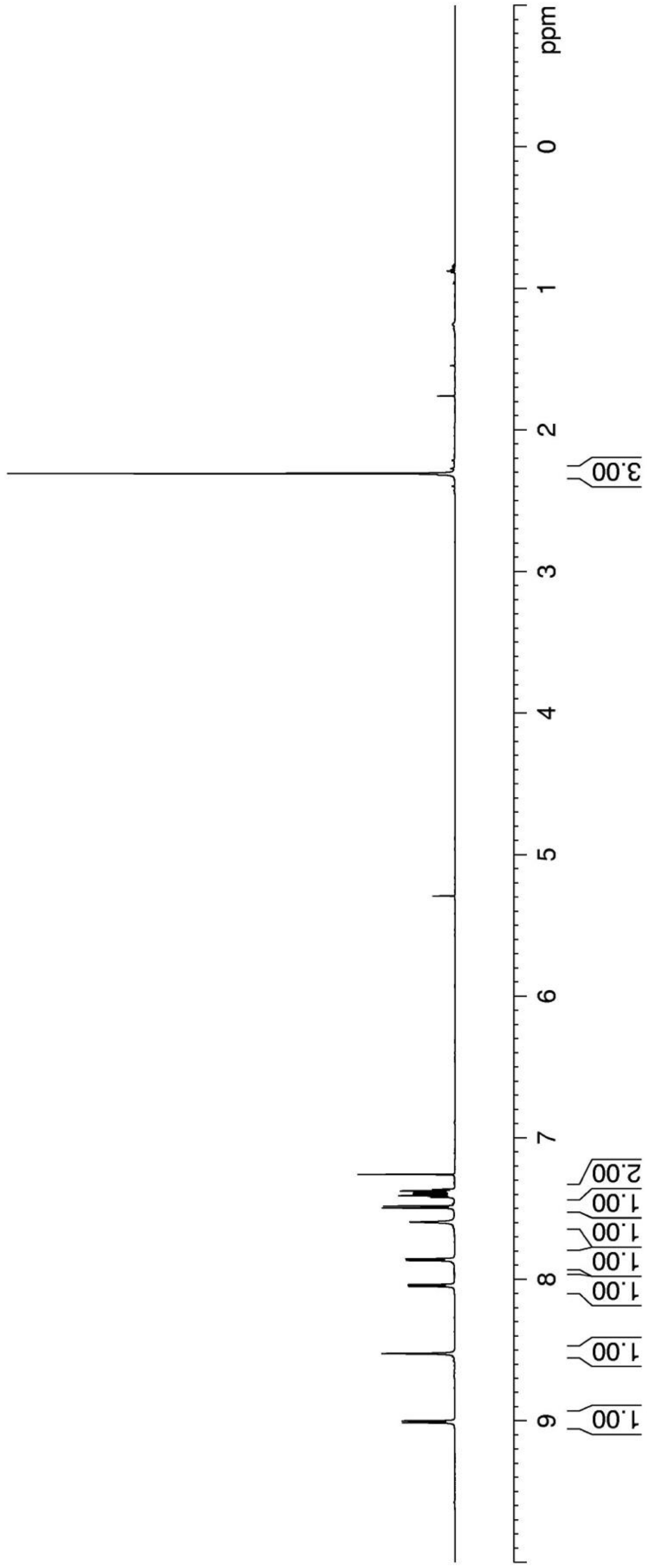

^{13}C NMR ($CDCl_3$, 25 ºC) of **2p**

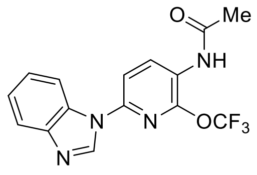

2p

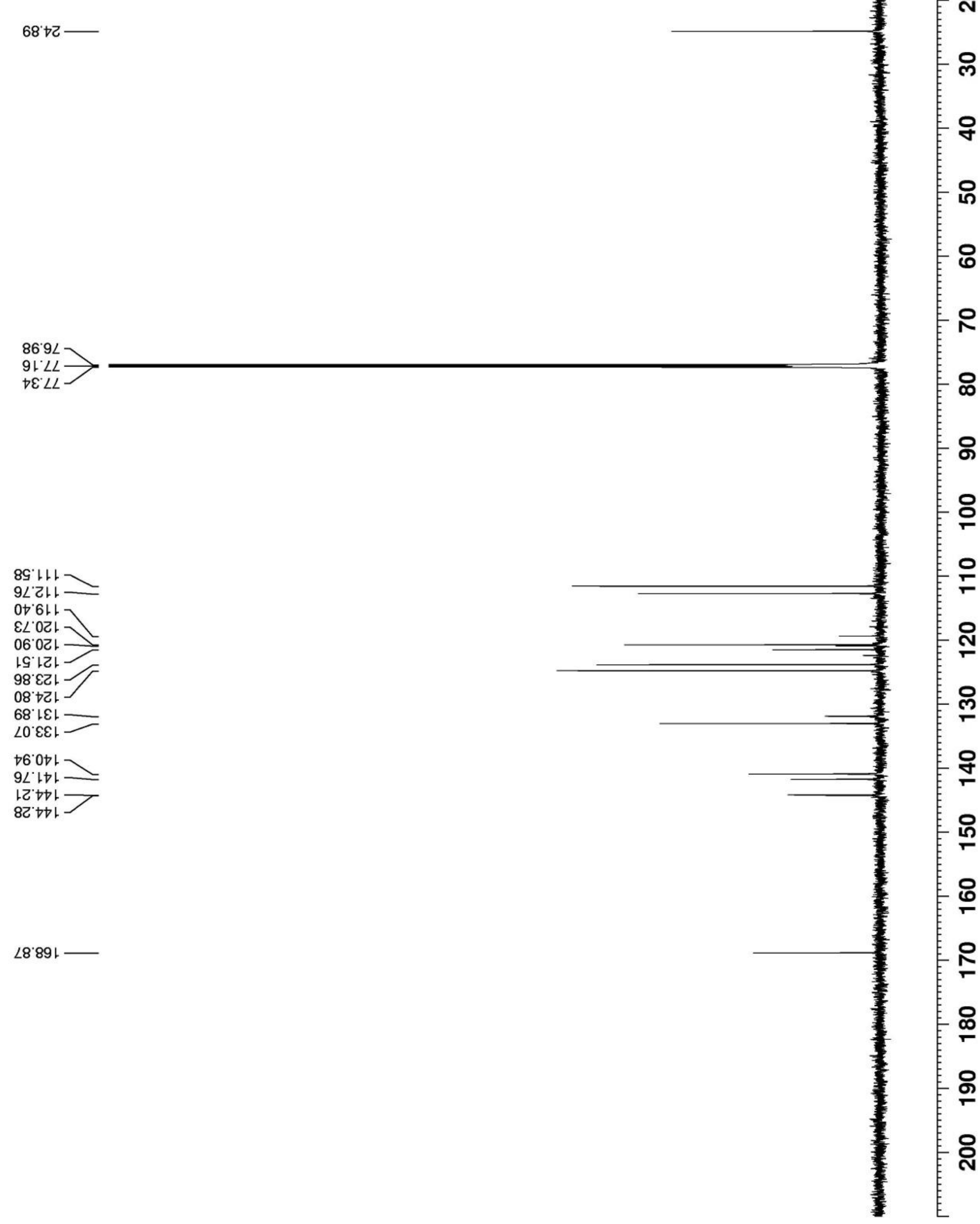

^{19}F NMR ($CDCl_3$, 25 ºC) of **2p**

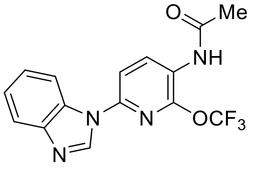

2p

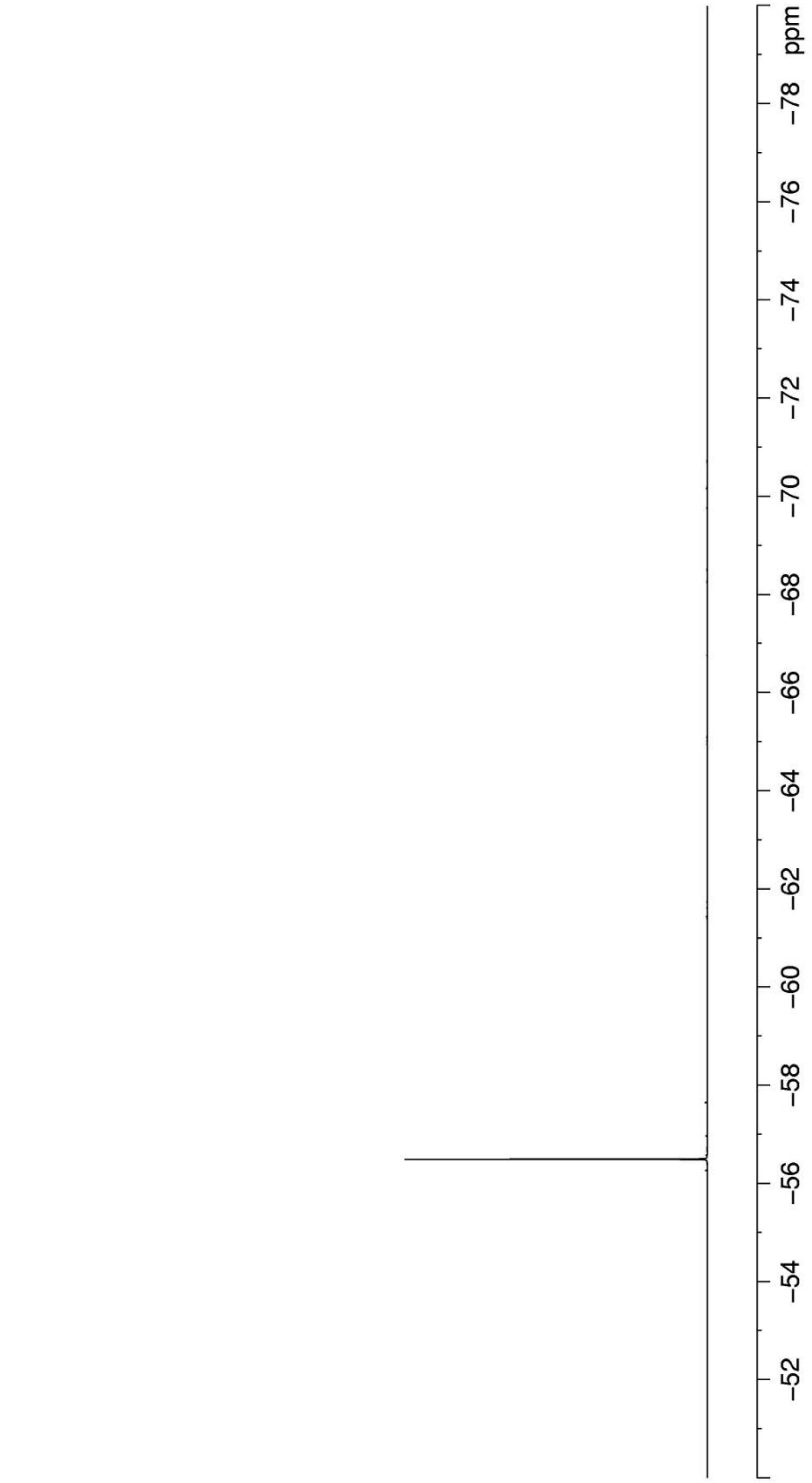

^{1}H NMR ((CD_3)$_2$SO, 25 ºC) of **2q**

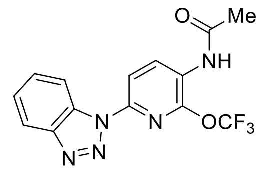

2q

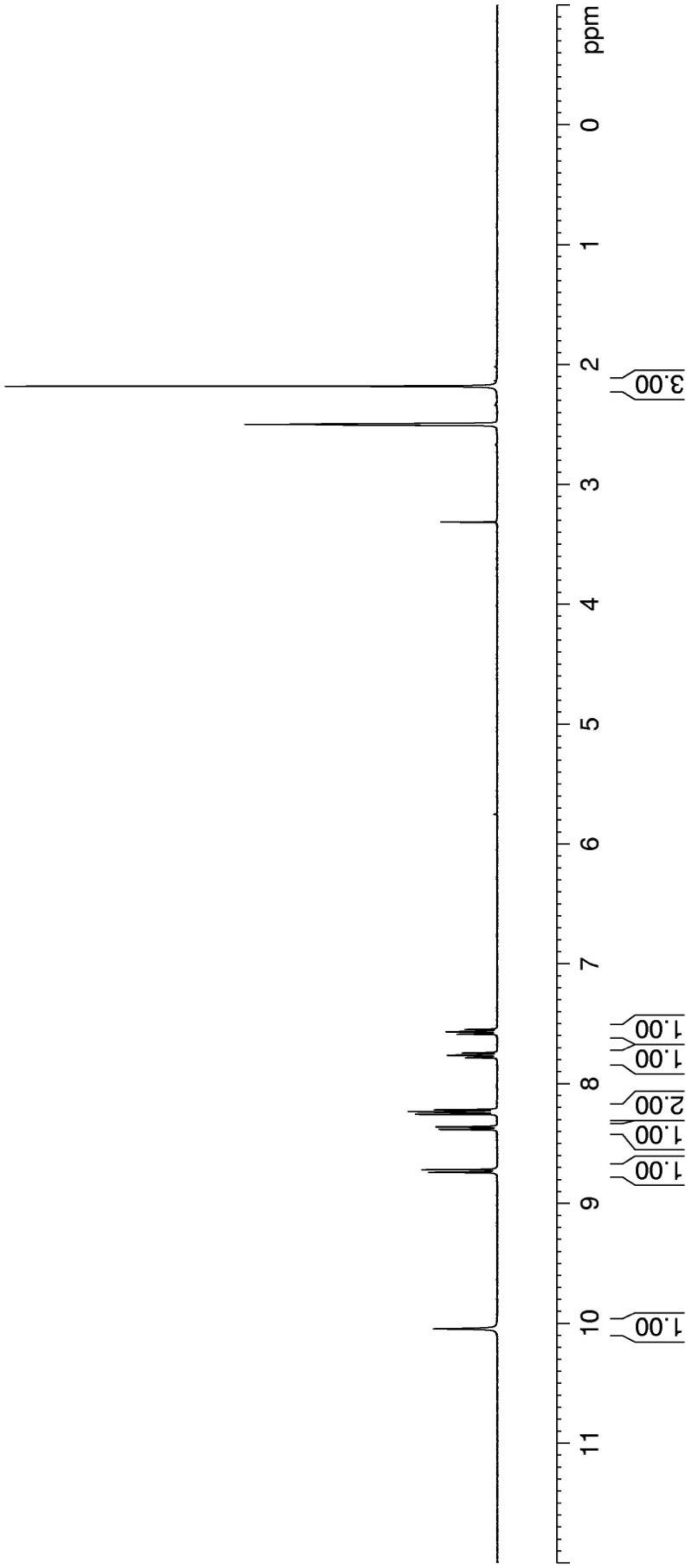

^{13}C NMR ($(CD_3)_2SO$, 25 ºC) of **2q**

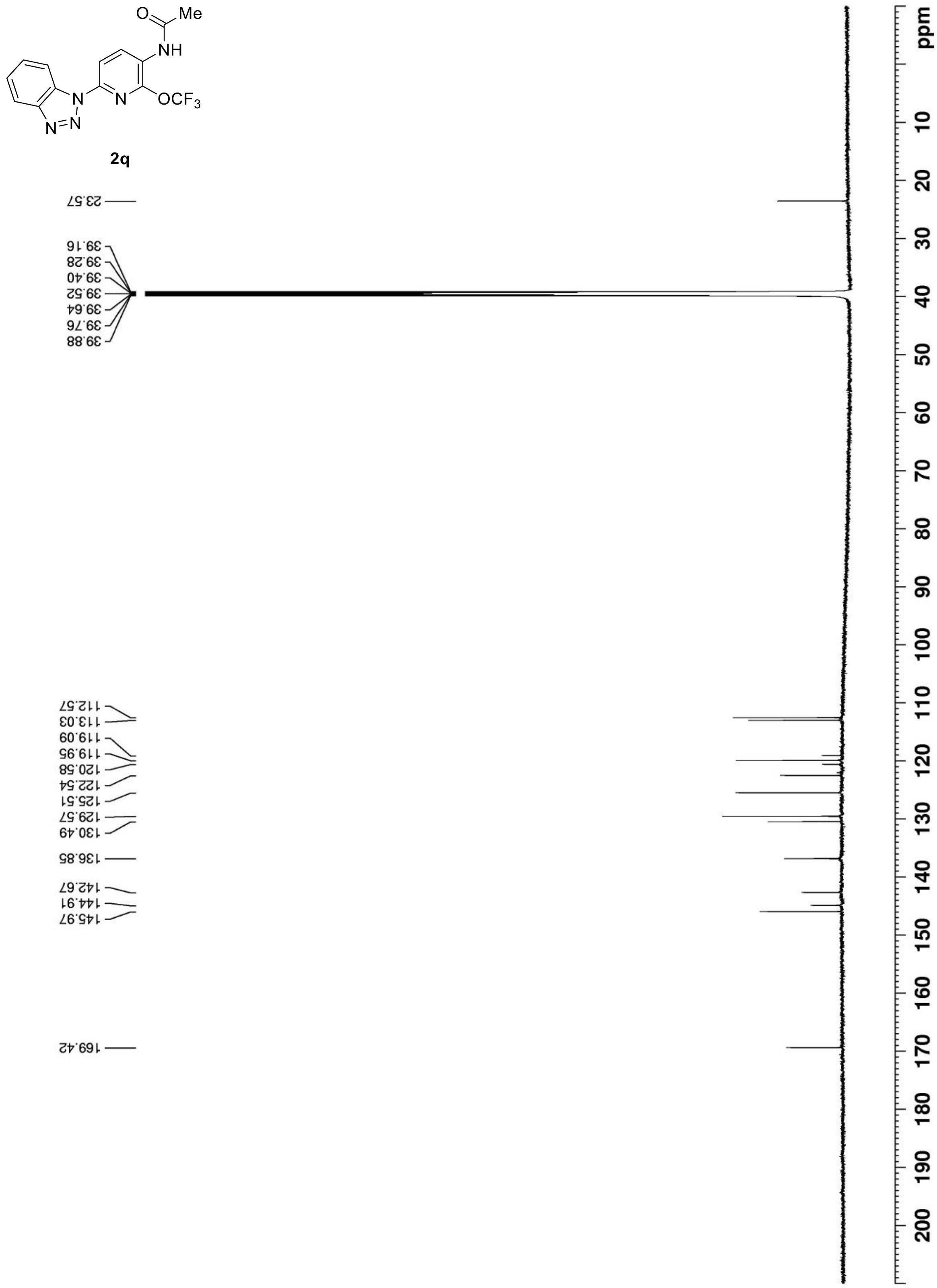

^{19}F NMR ($(CD_3)_2SO$, 25 ºC) of **2q**

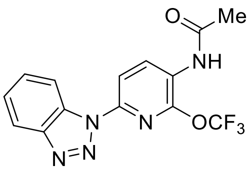

2q

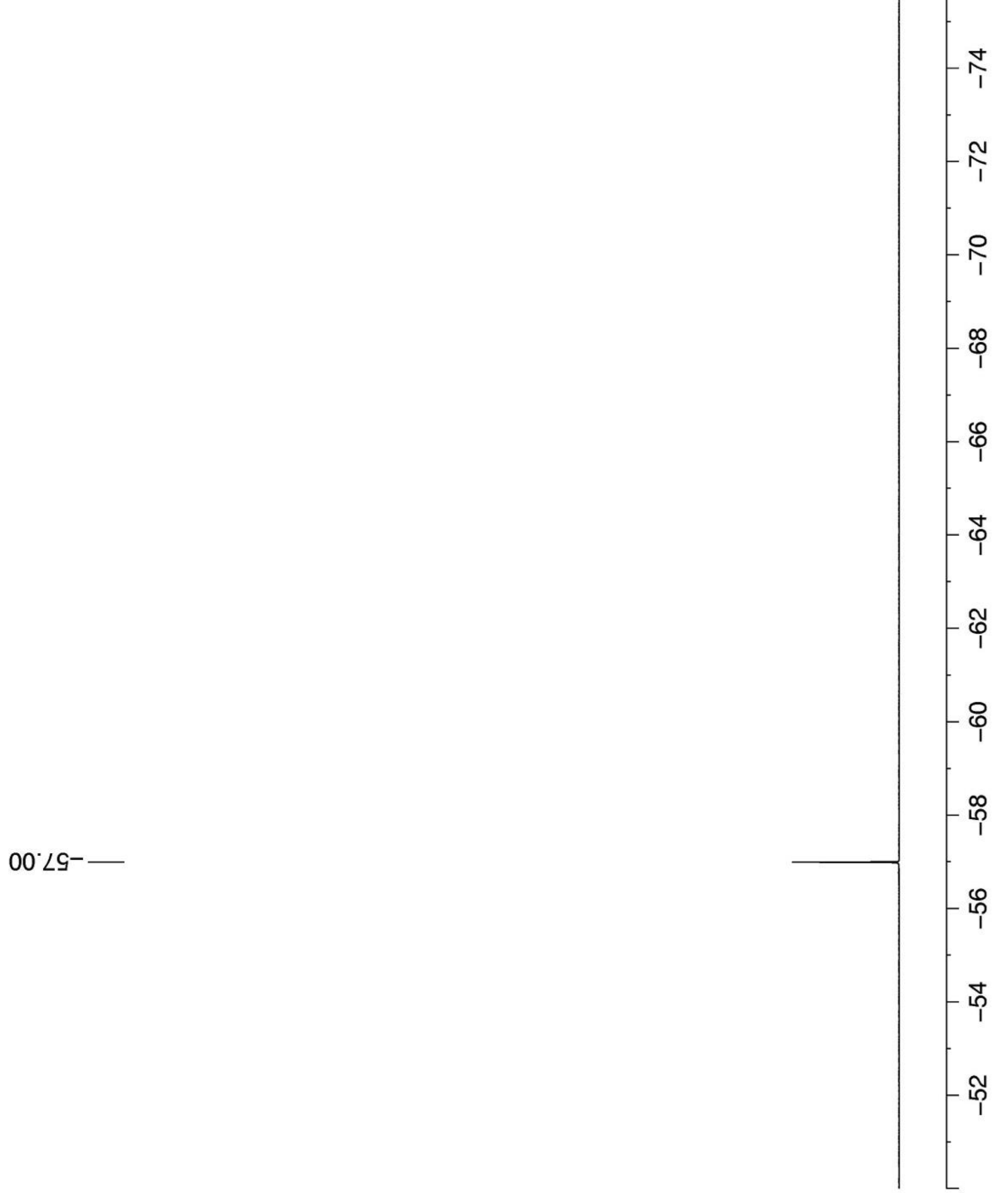

^{1}H NMR ($CDCl_3$, 25 ºC) of **2r**

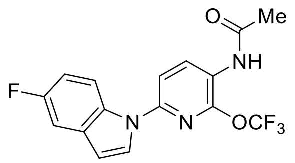

2r

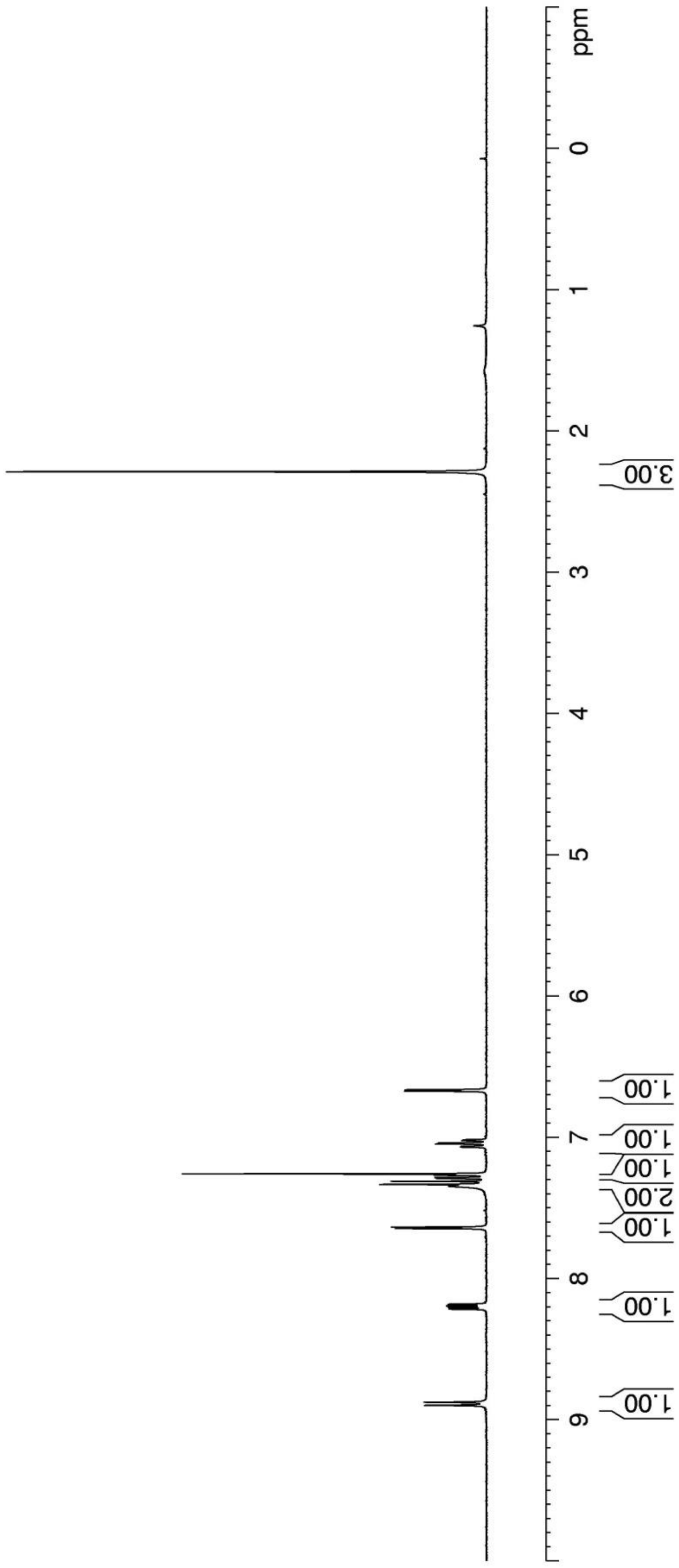

^{13}C NMR ($CDCl_3$, 25 ºC) of **2r**

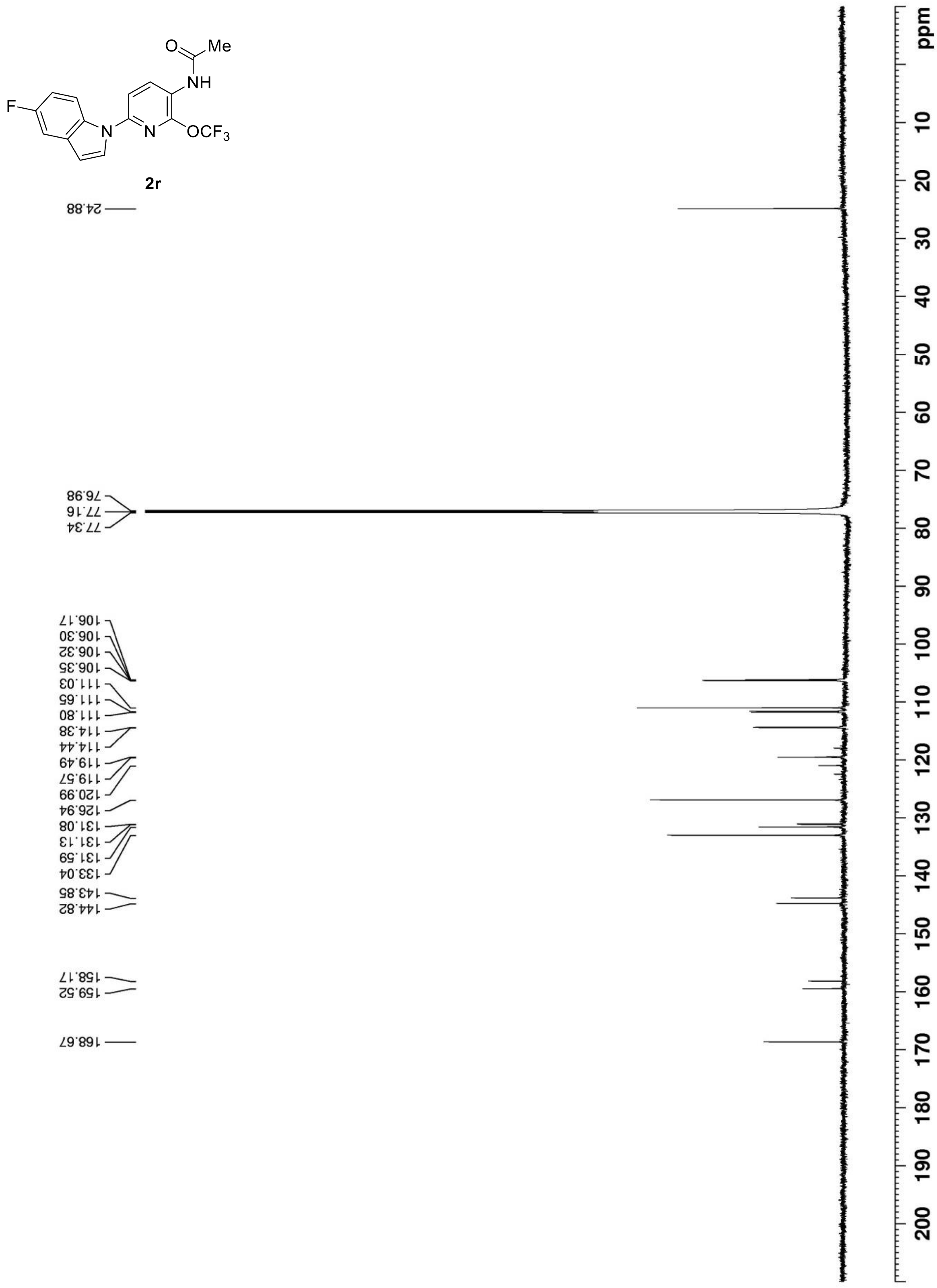

^{19}F NMR ($CDCl_3$, 25 ºC) of **2r**

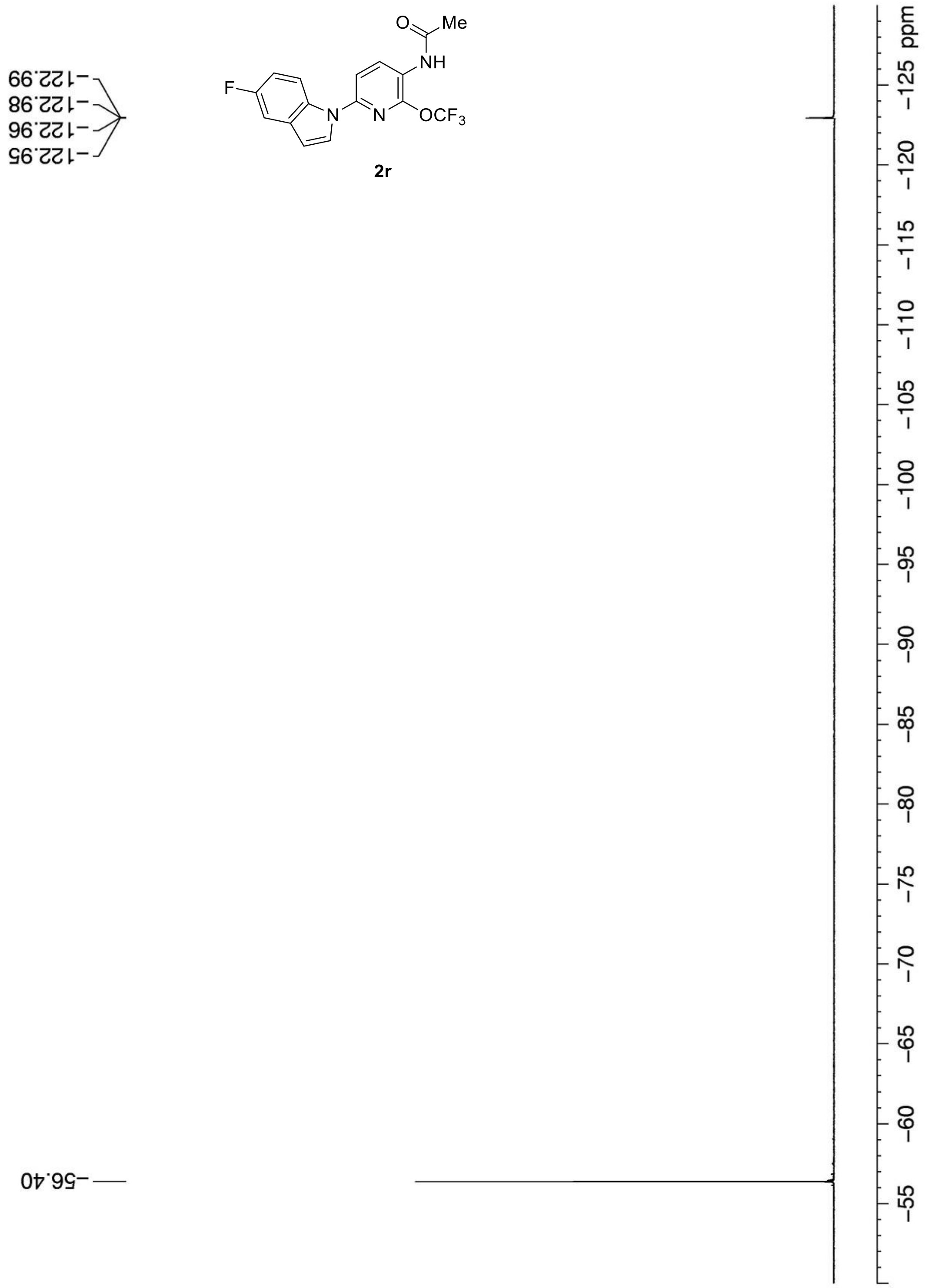

^{1}H NMR ($CDCl_3$, 25 ºC) of **2s**

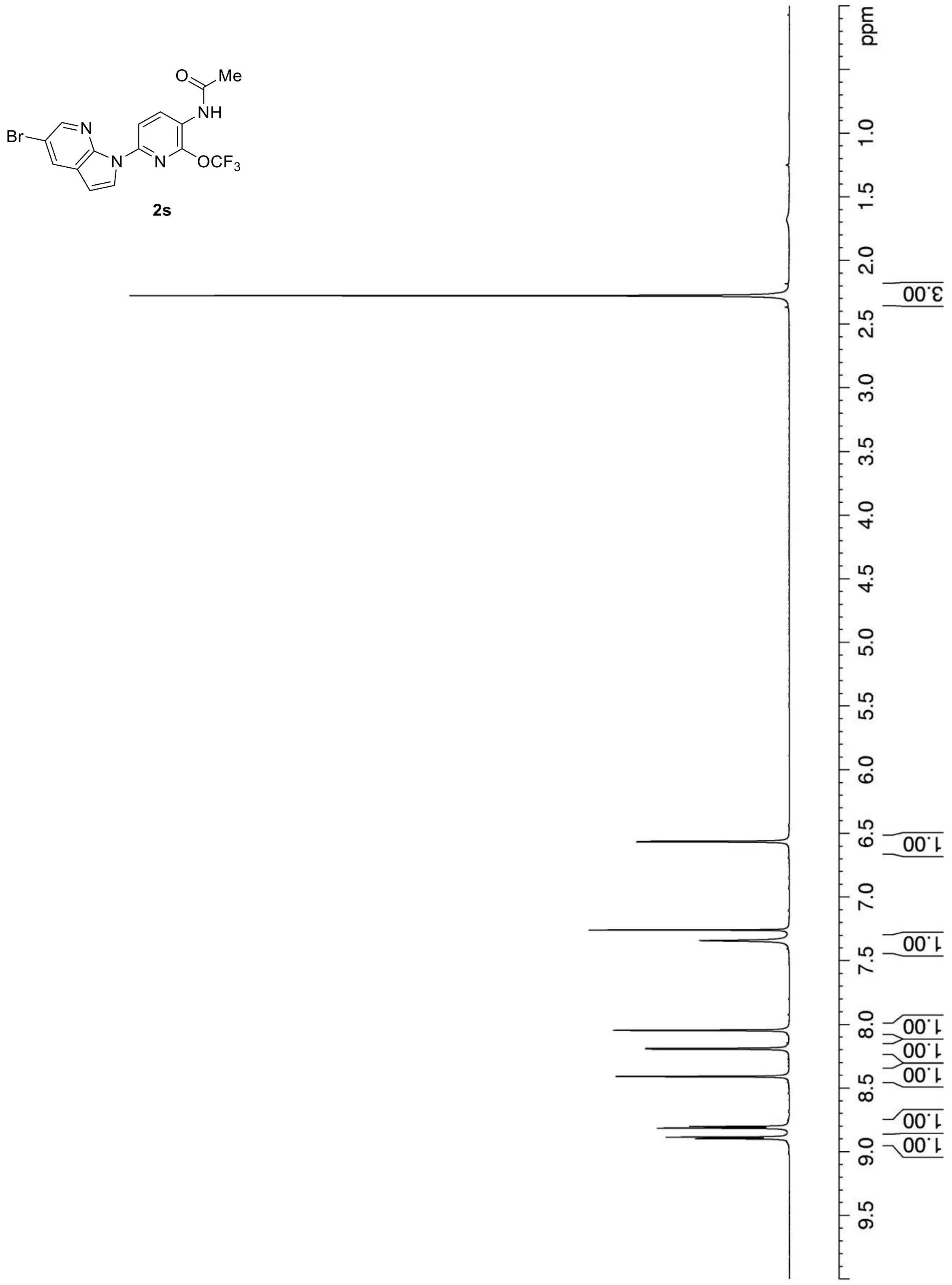

^{13}C NMR ($CDCl_3$, 25 ºC) of **2s**

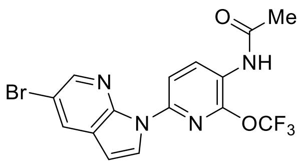

2s

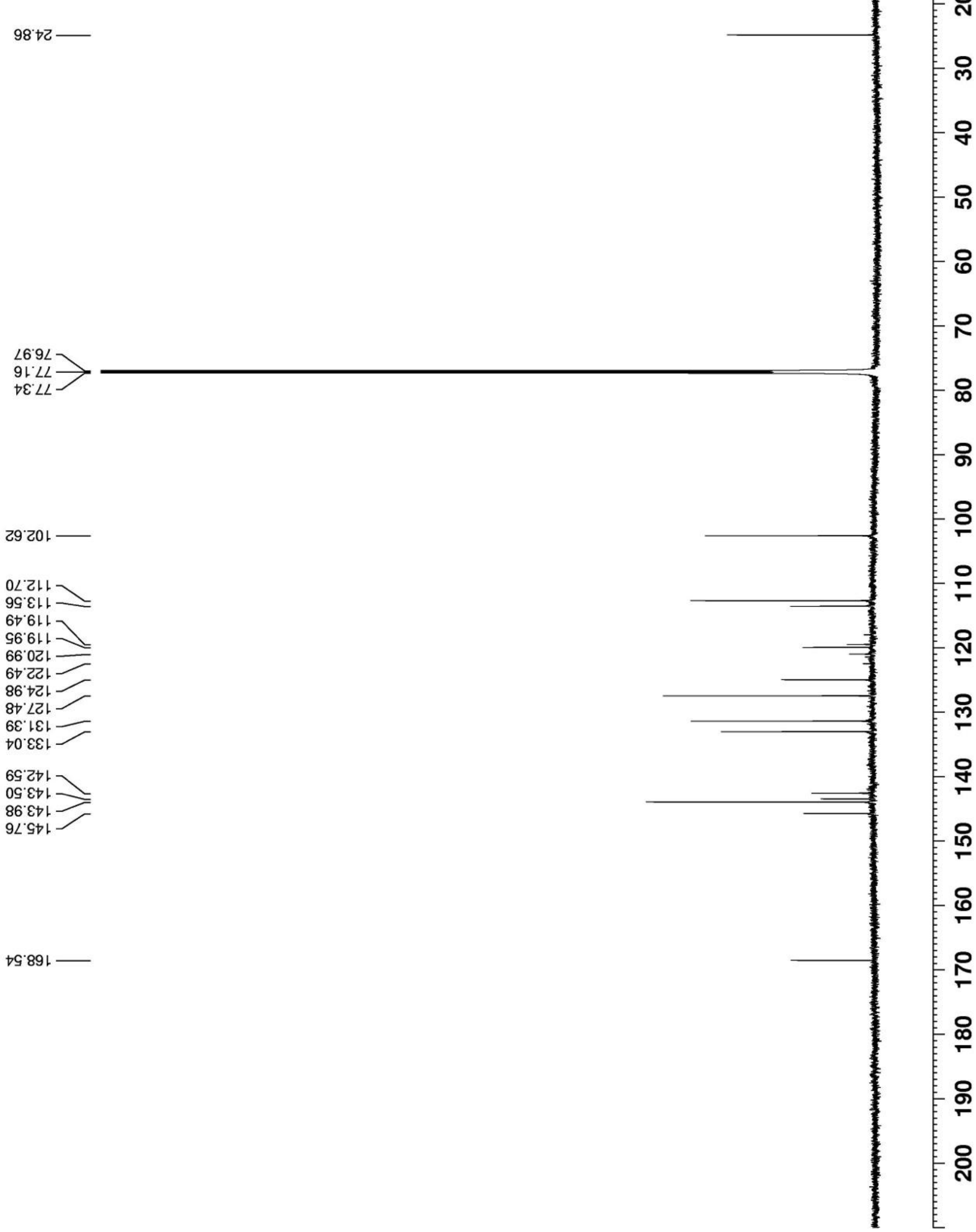

^{19}F NMR ($CDCl_3$, 25 ºC) of **2s**

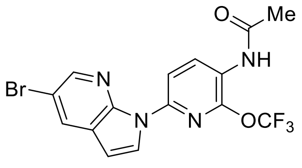

2s

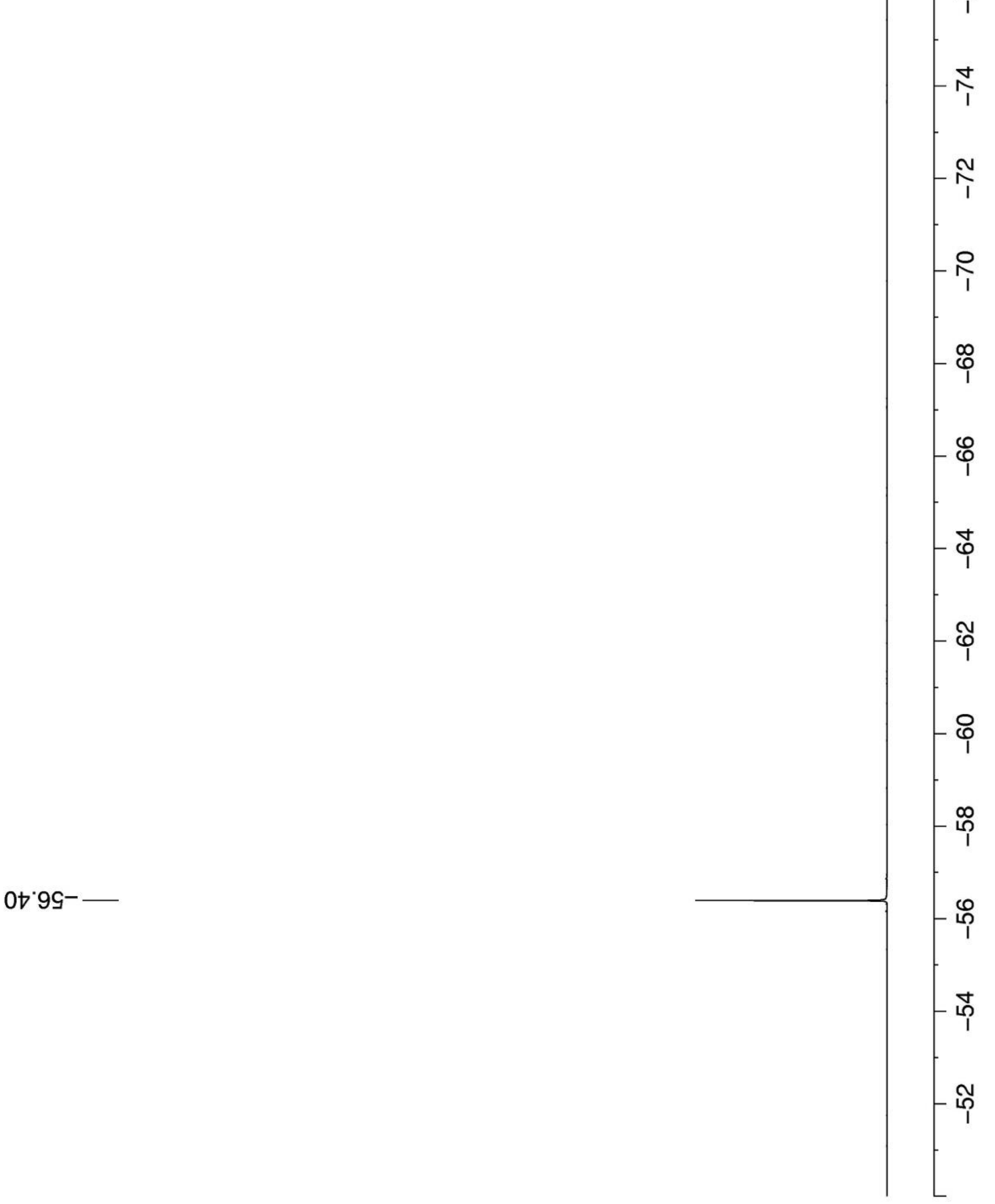

^{1}H NMR ($(CD_3)_2SO$, 25 ºC) of **2t**

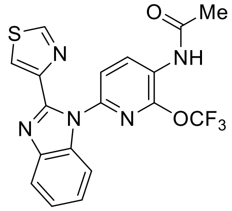

2t

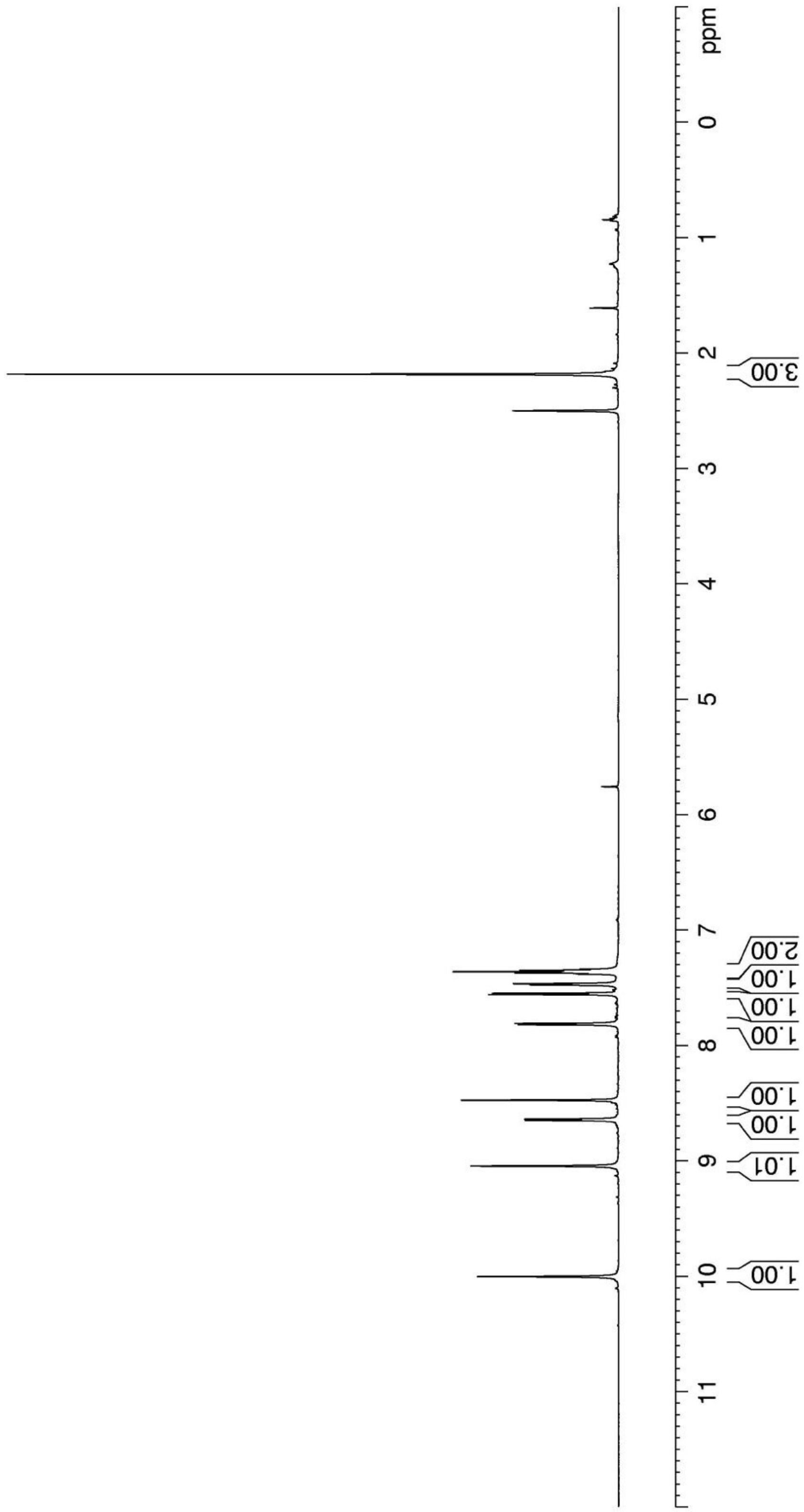

^{13}C NMR (($(CD_3)_2SO$, 25 ºC) of **2t**

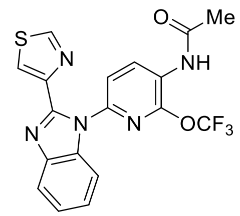

2t

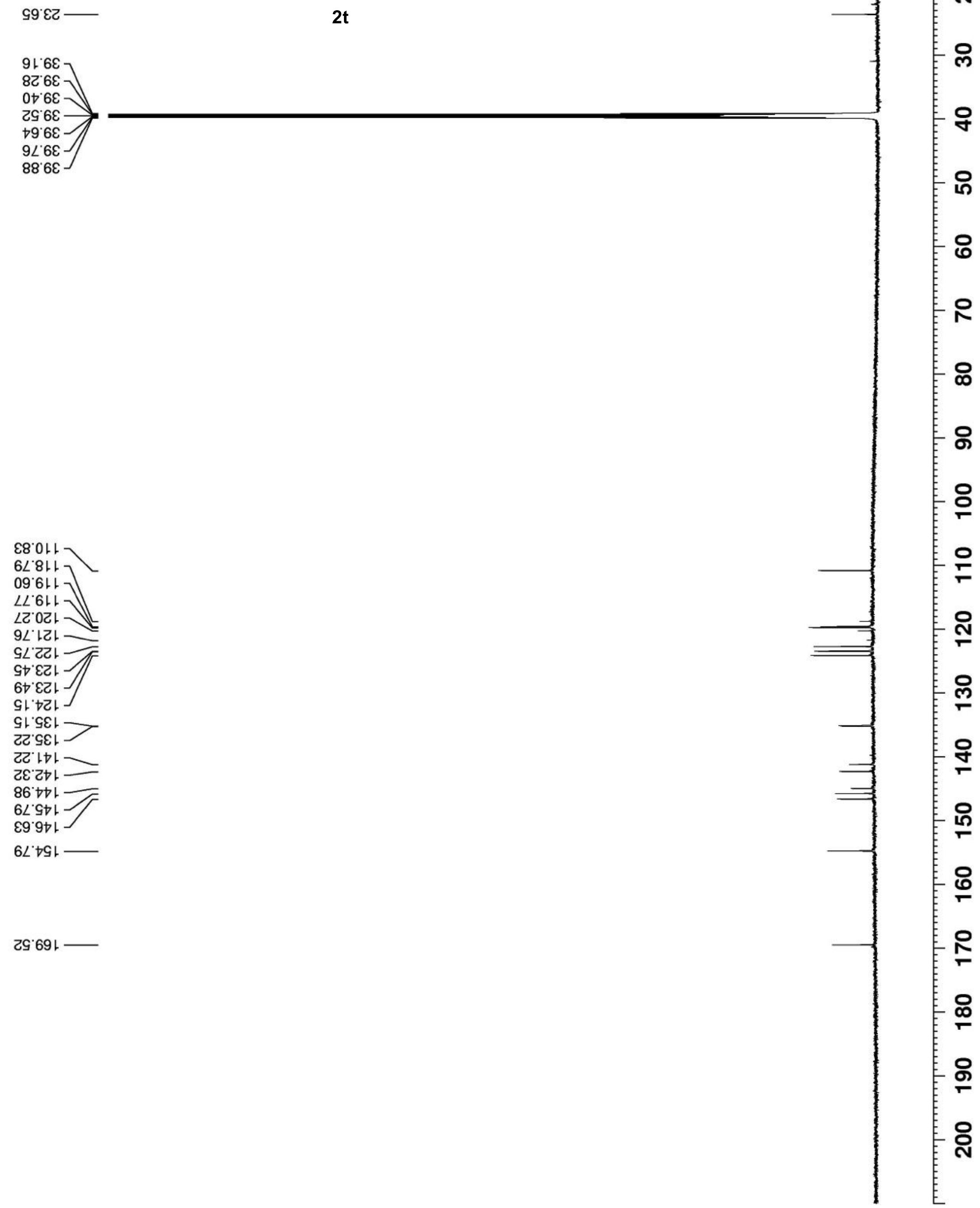

^{19}F NMR ((CD_3)$_2$SO, 25 ºC) of **2t**

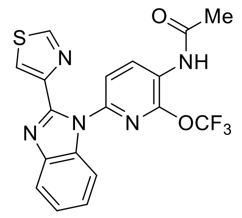

2t

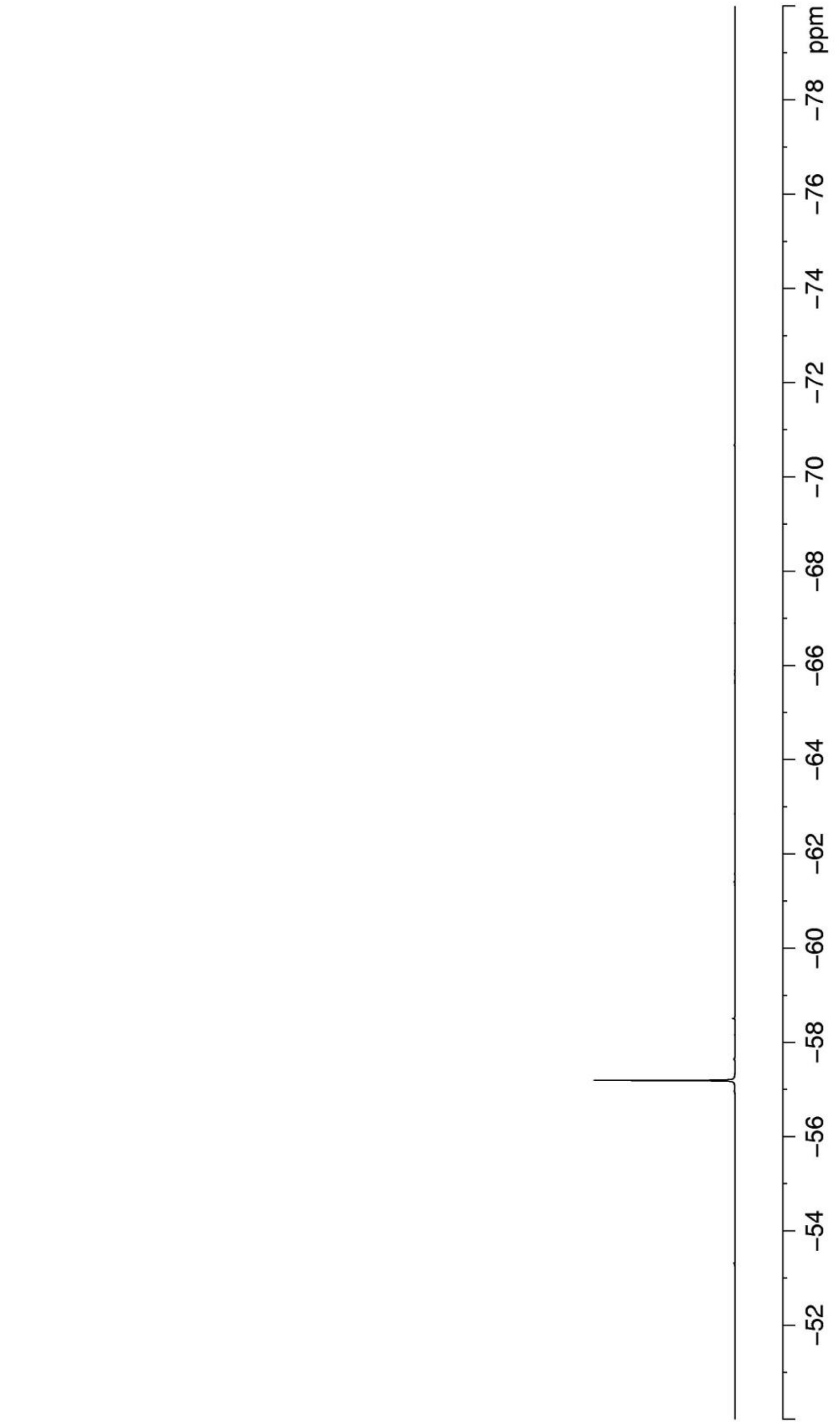

^{1}H NMR ($CDCl_3$, 25 ºC) of **2u**

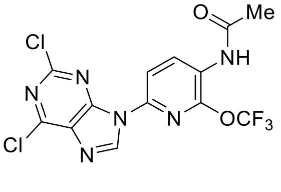

2u

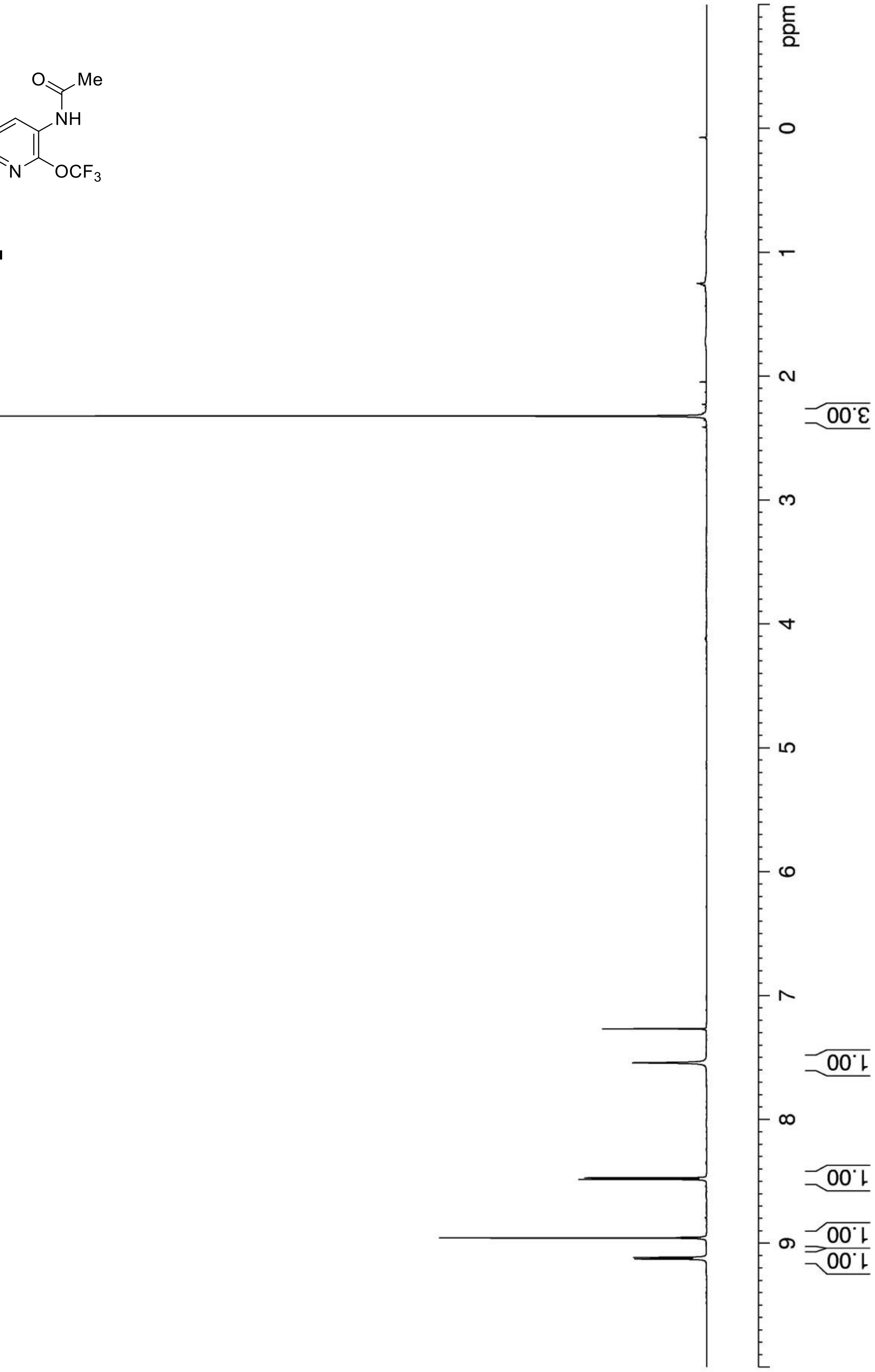

^{13}C NMR ($CDCl_3$, 25 ºC) of **2u**

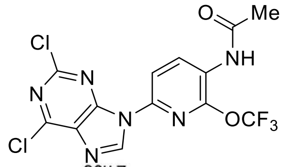

2u

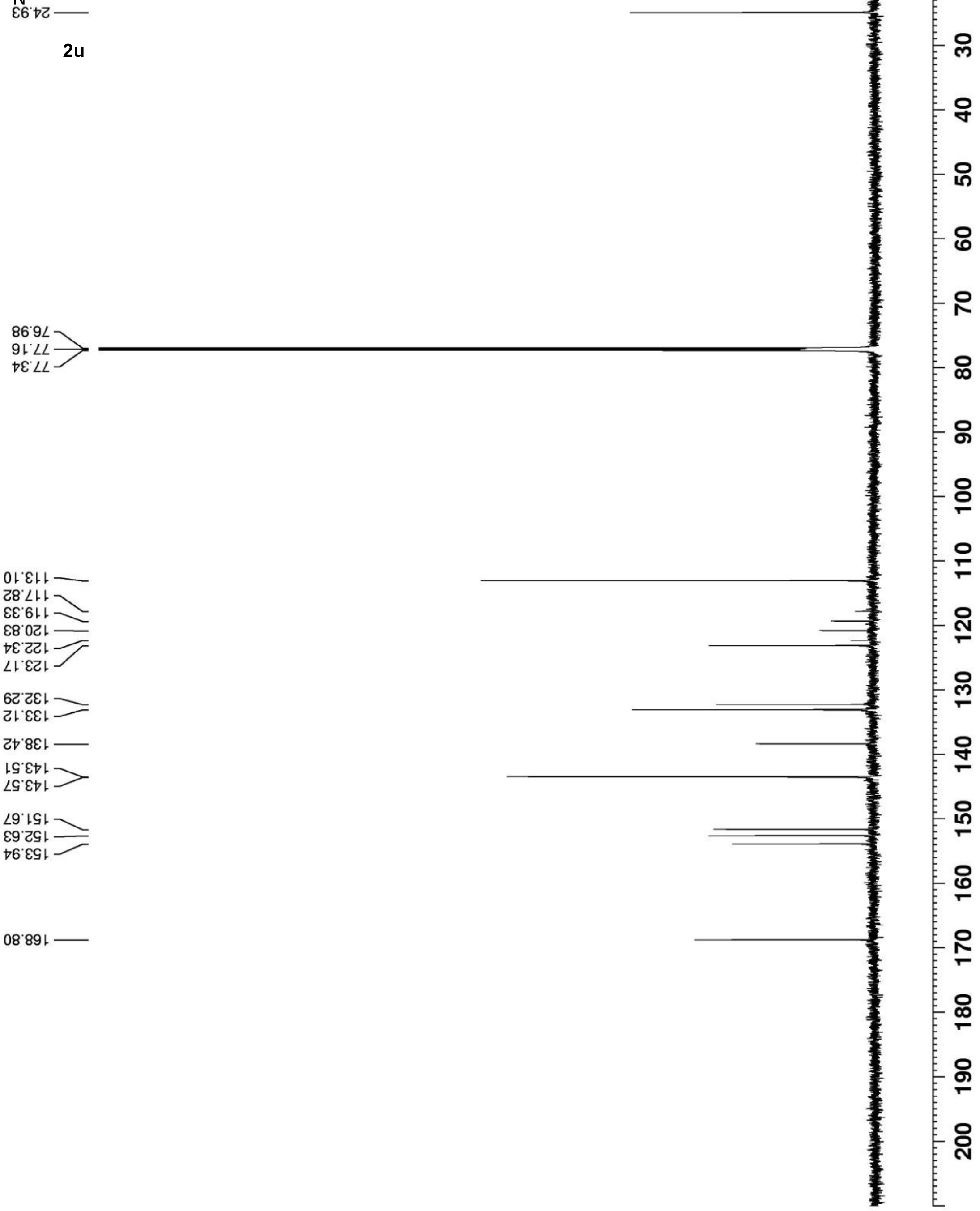

^{19}F NMR ($CDCl_3$, 25 ºC) of **2u**

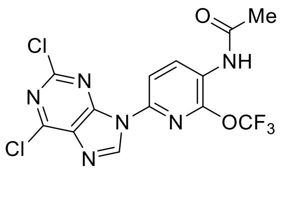

2u

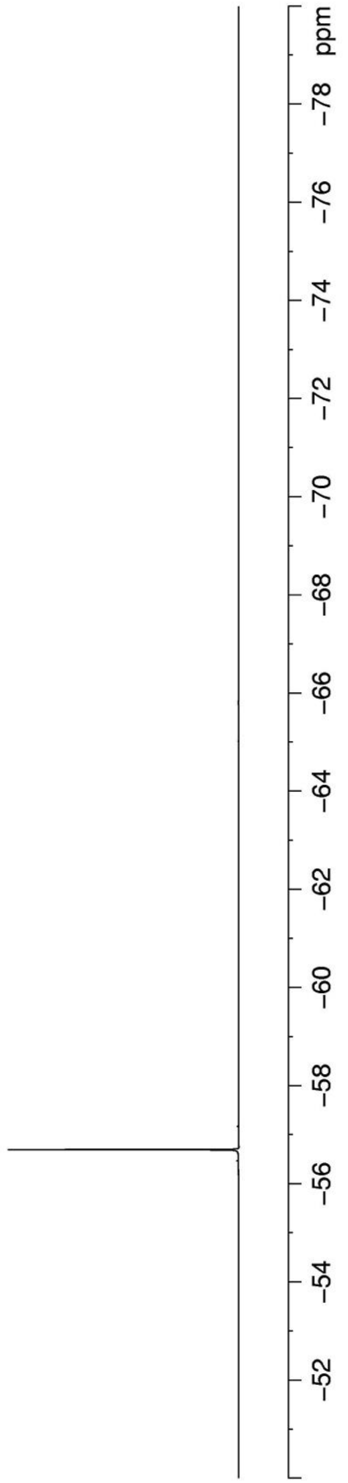

^{1}H NMR ($CDCl_3$, 25 ºC) of **2u-II**

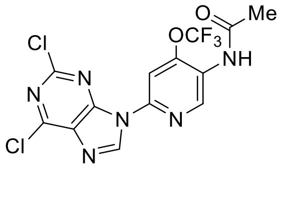

2u-II

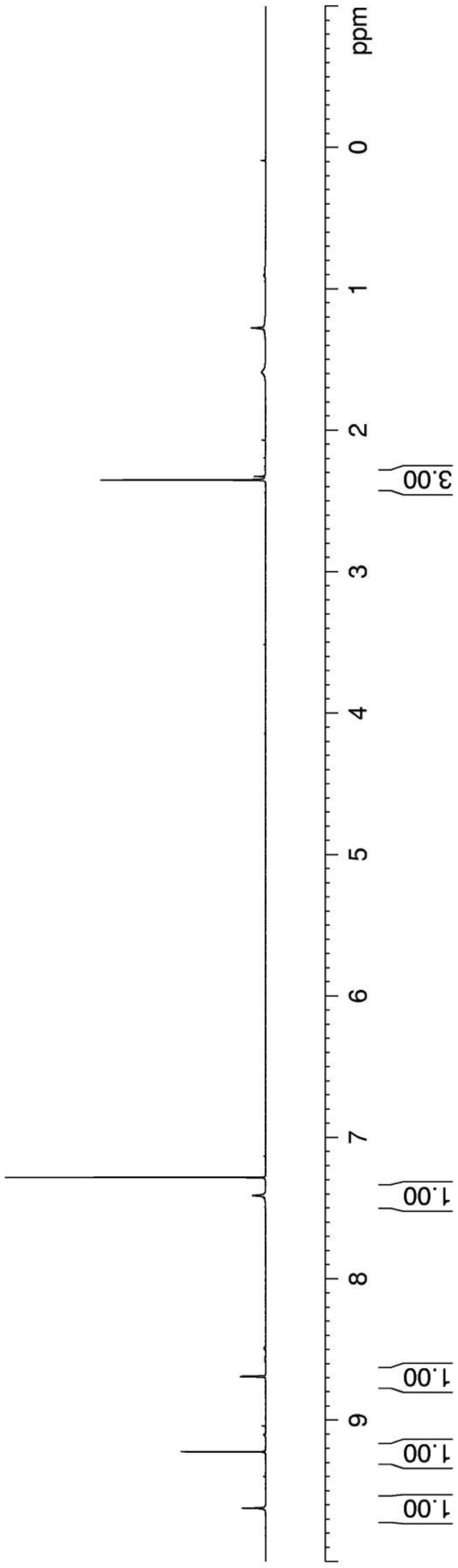

^{13}C NMR ($CDCl_3$, 25 ºC) of **2u-II**

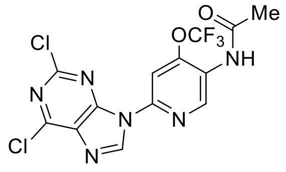

2u-II

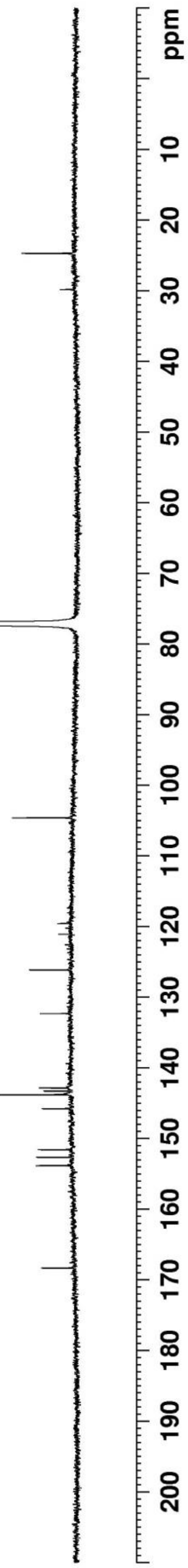

^{19}F NMR ($CDCl_3$, 25 ºC) of **2u-II**

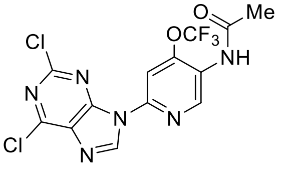

2u-II

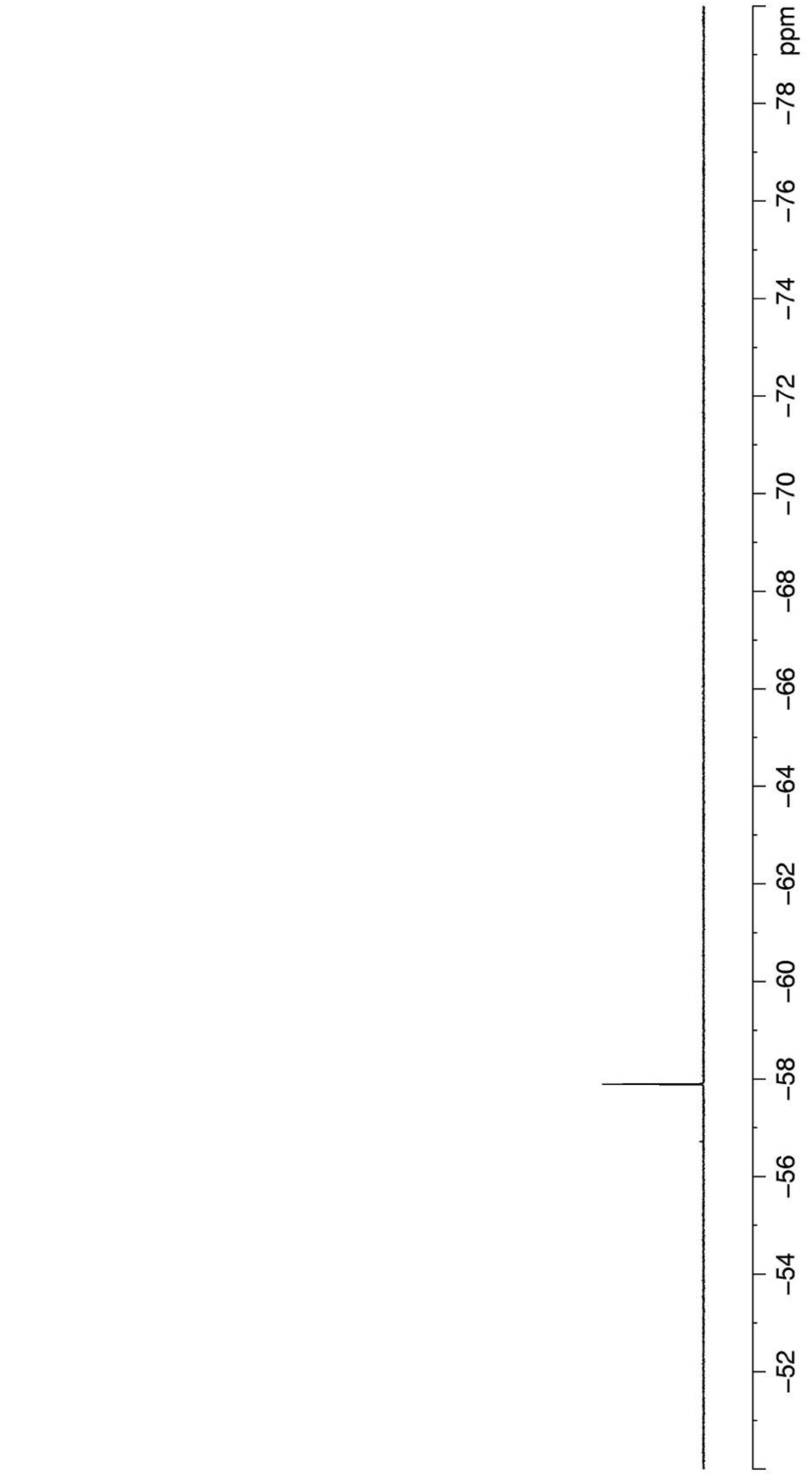

^{1}H NMR ($CDCl_3$, 25 ºC) of **2v**

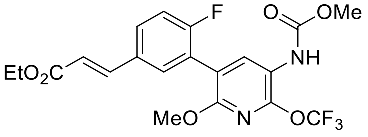

2v

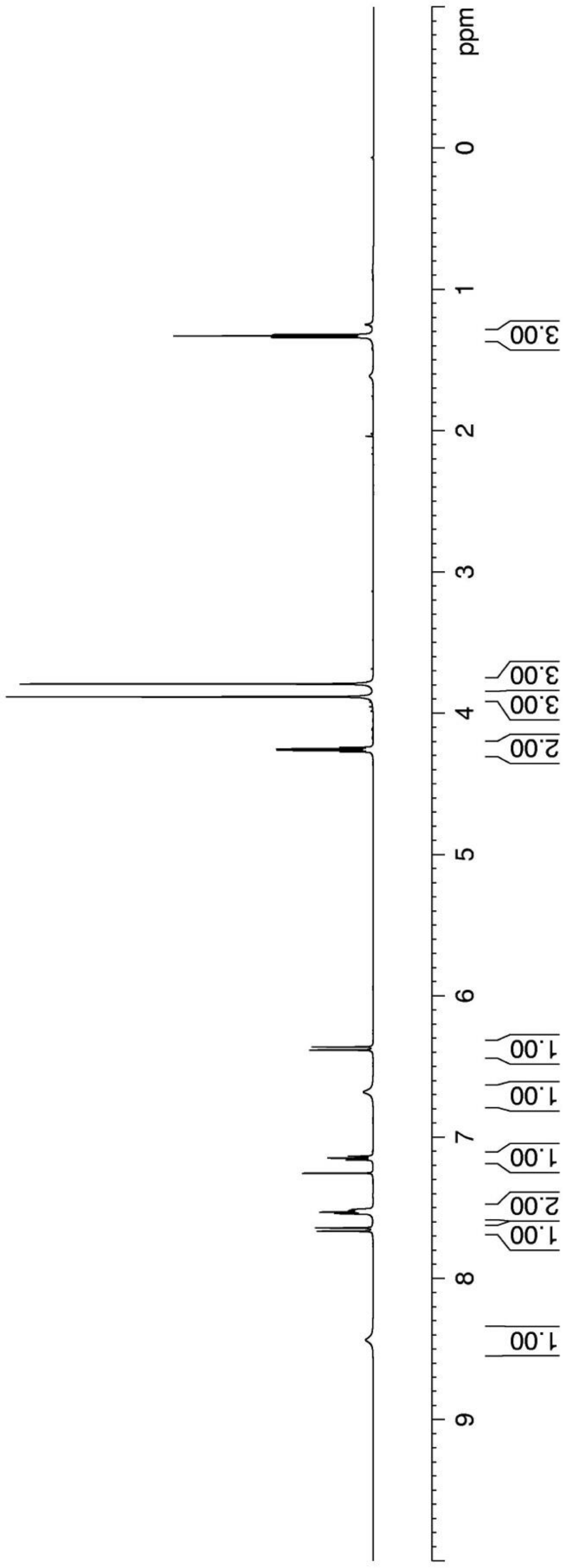

^{13}C NMR ($CDCl_3$, 25 ºC) of **2v**

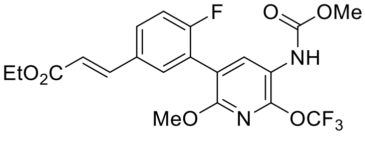

2v

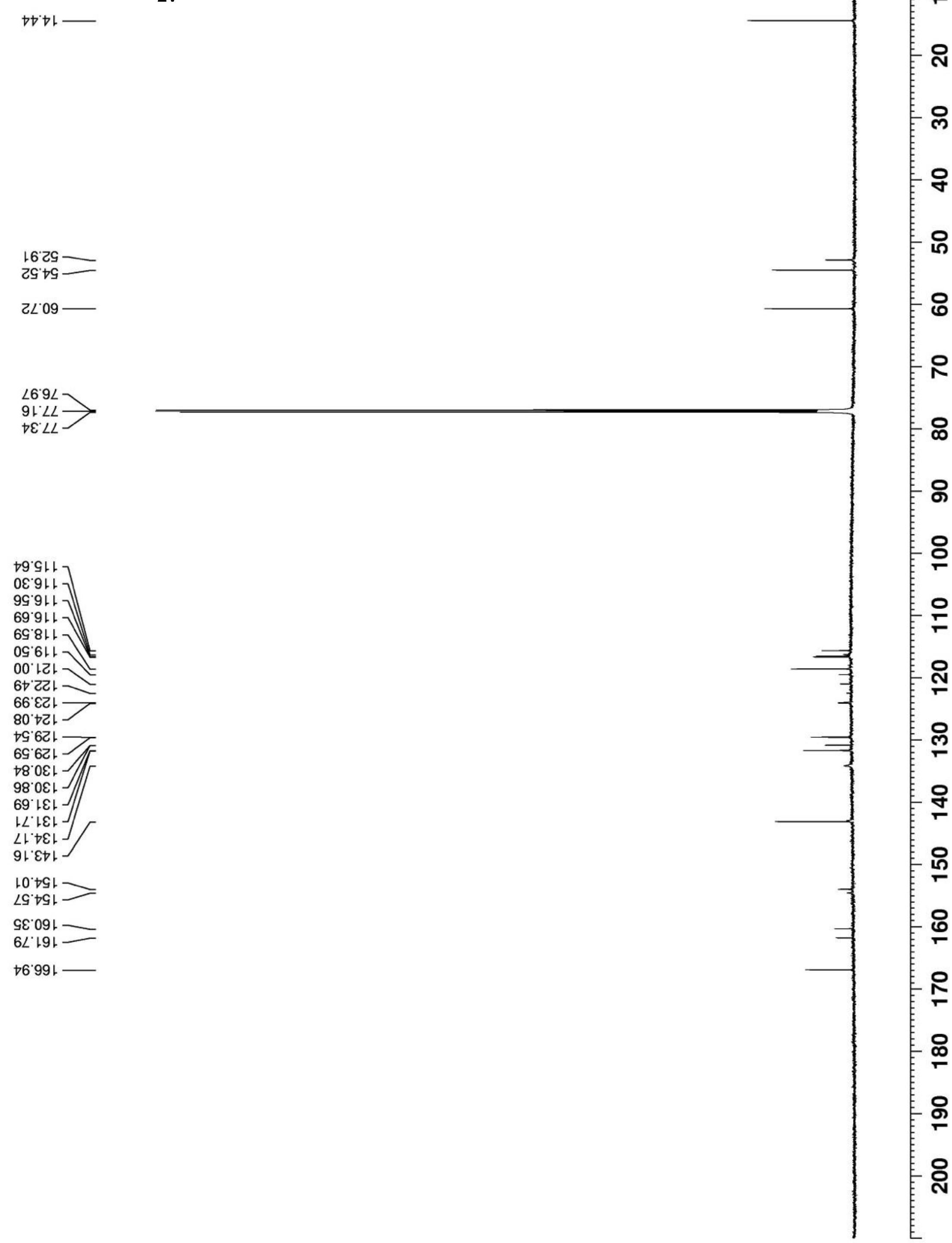

^{19}F NMR ($CDCl_3$, 25 ºC) of **2v**

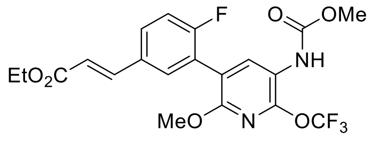

2v

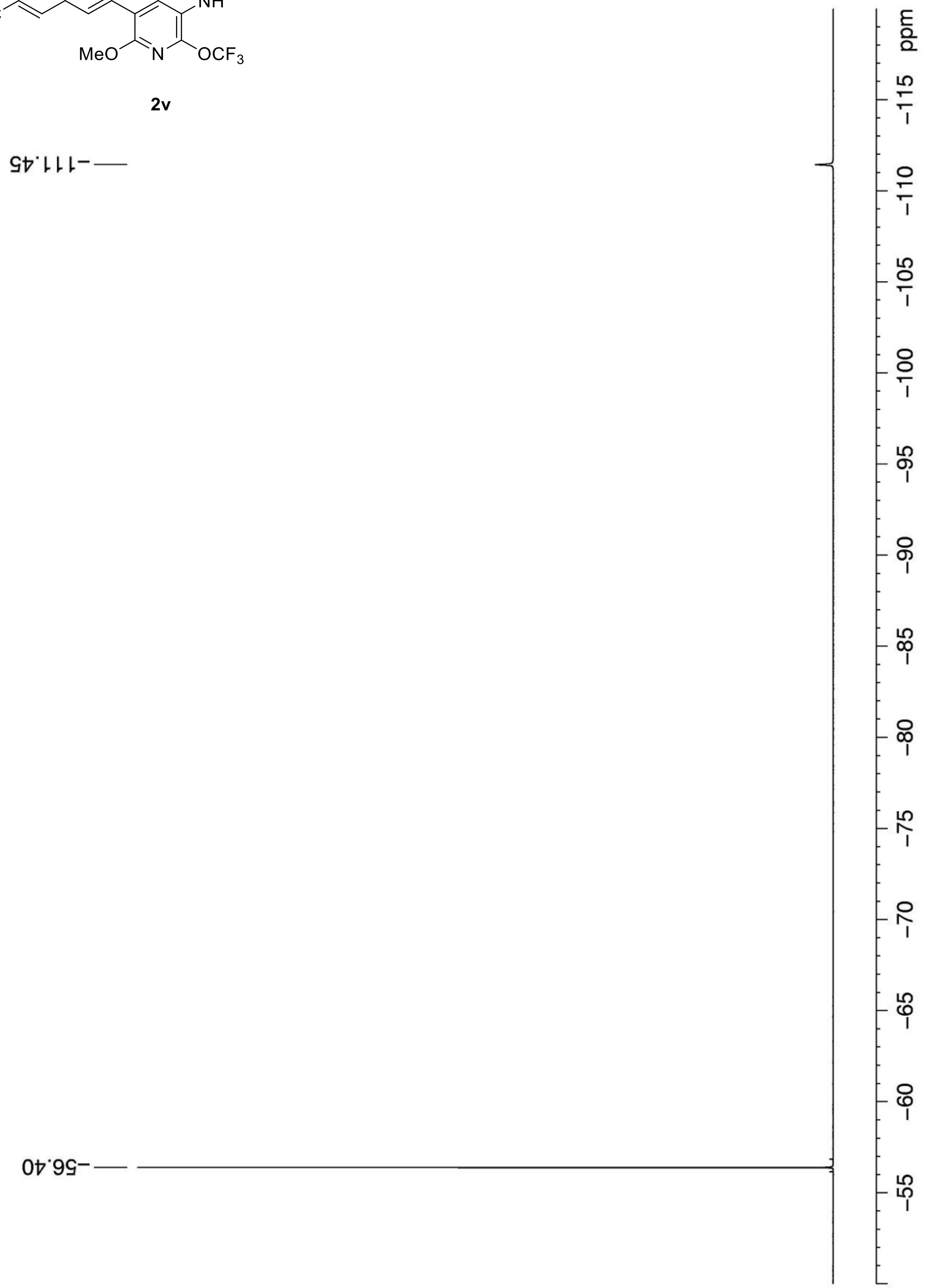

^{1}H NMR ($CDCl_3$, 25 ºC) of **2w**

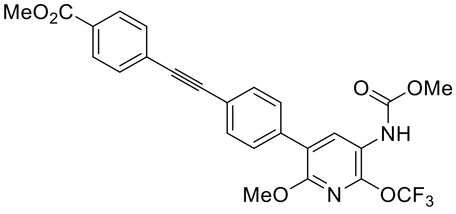

2w

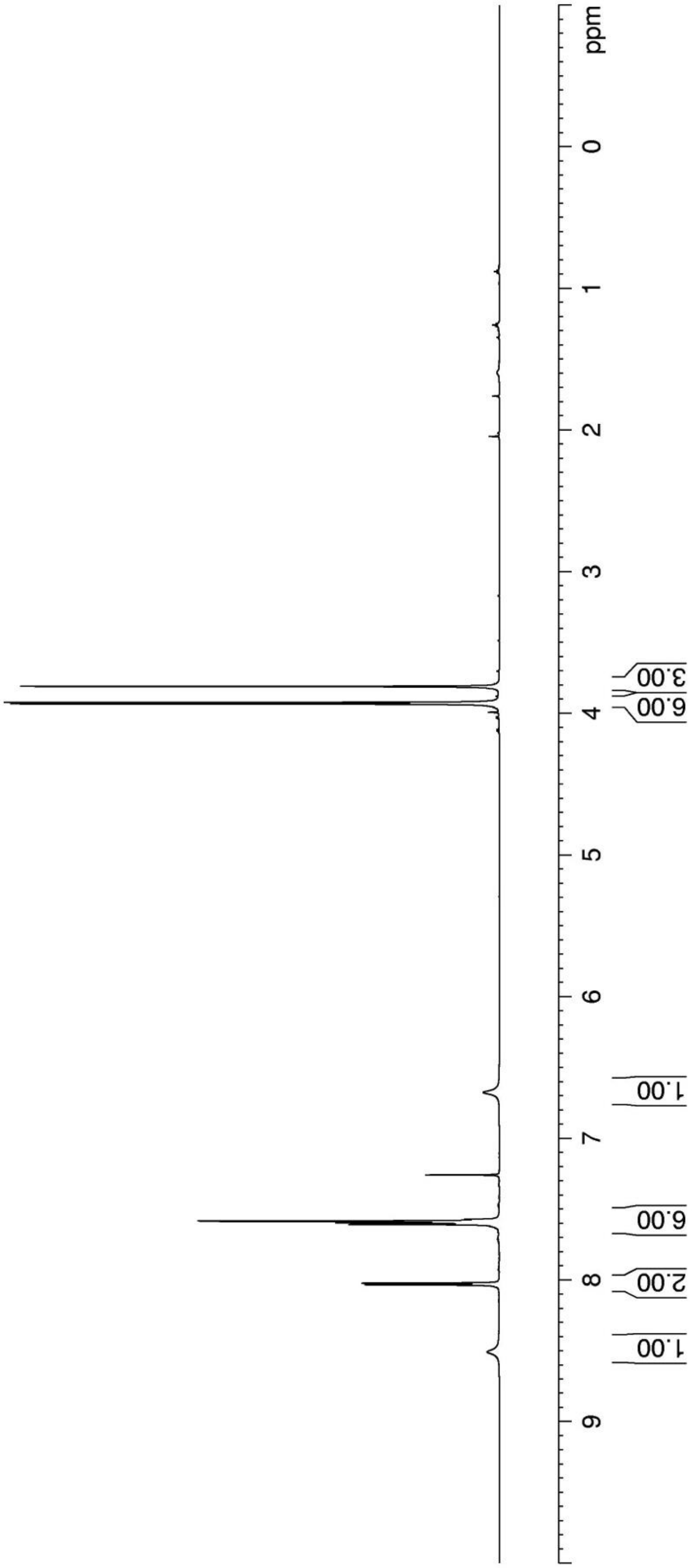

^{13}C NMR ($CDCl_3$, 25 ºC) of **2w**

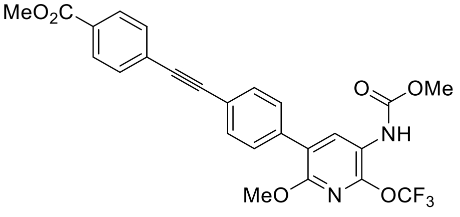

2w

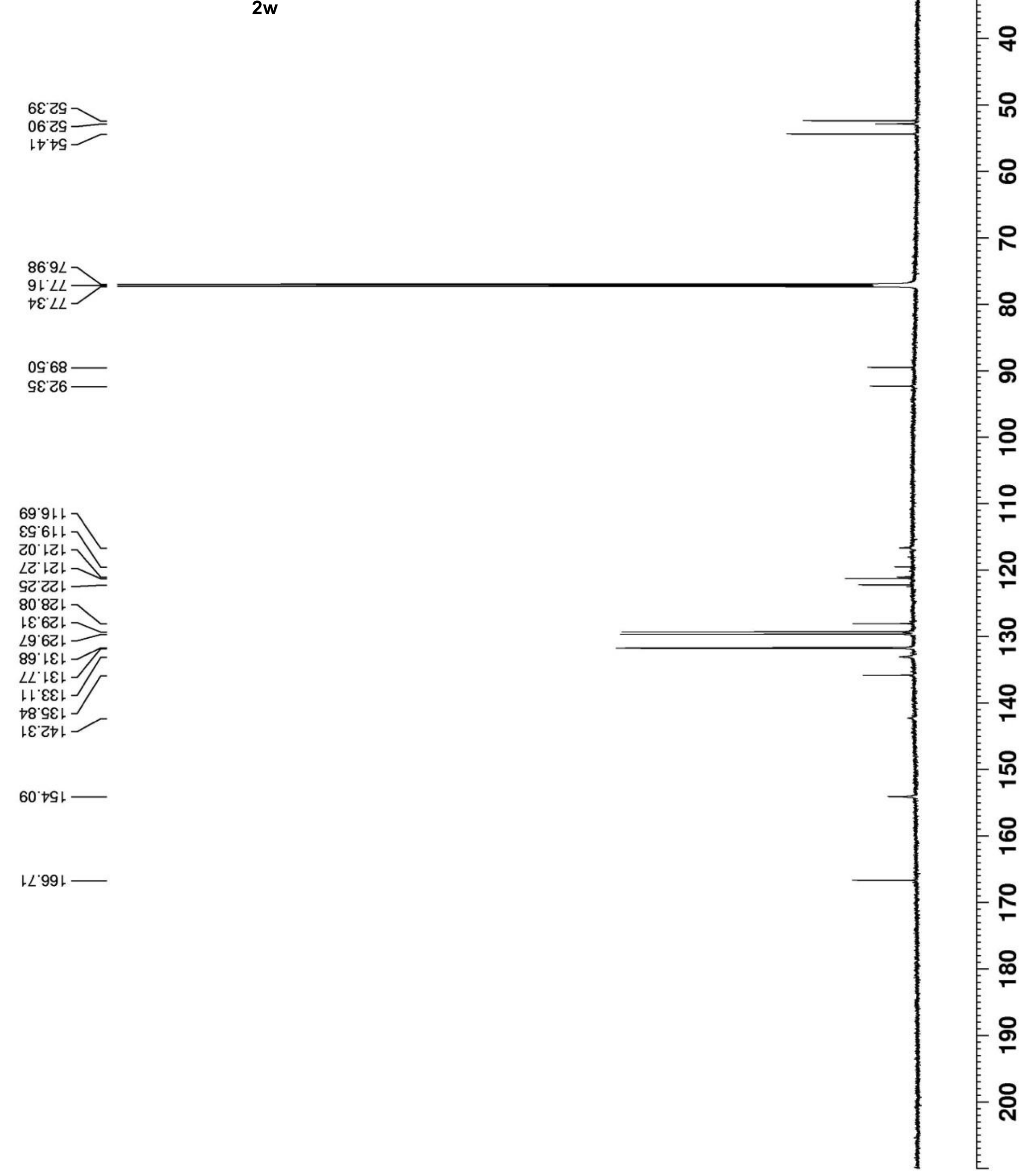

^{19}F NMR ($CDCl_3$, 25 ºC) of **2w**

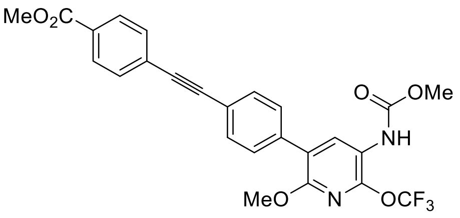

2w

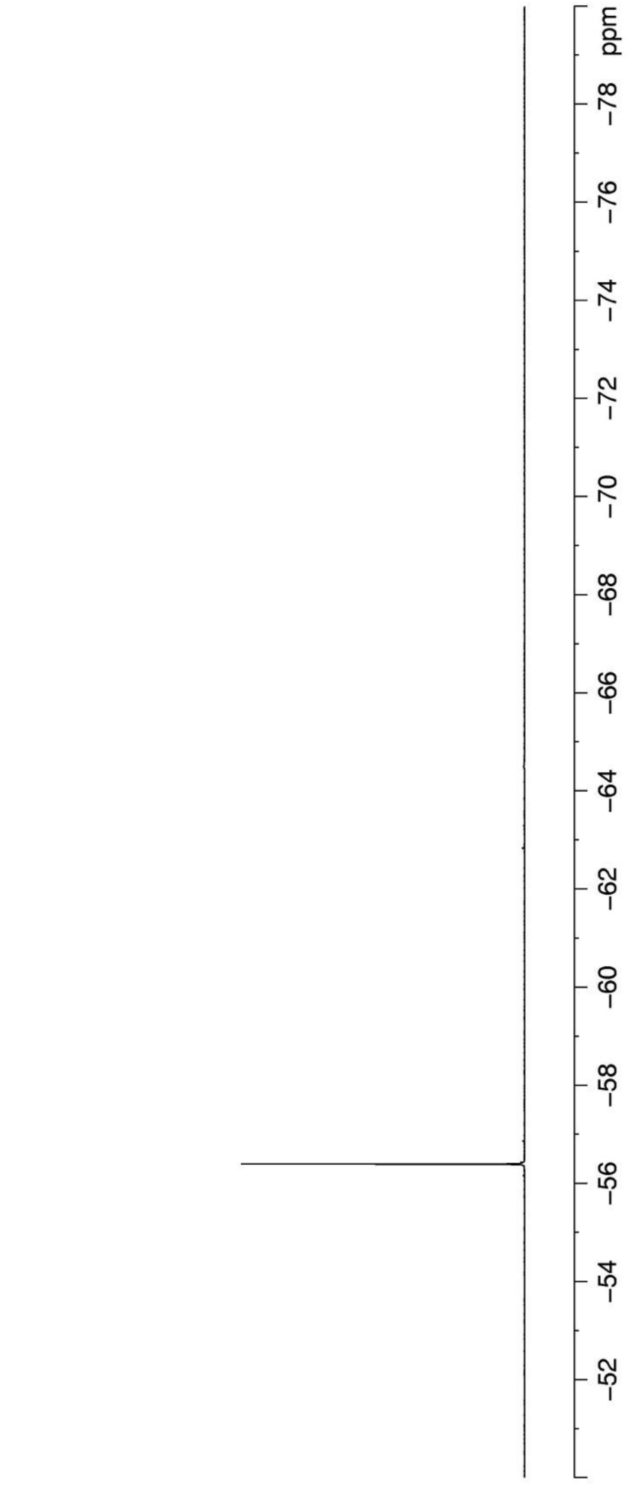

^{1}H NMR ($CDCl_3$, 25 ºC) of **2x**

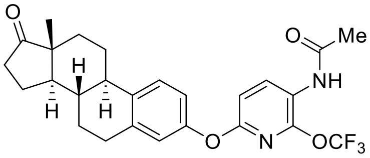

2x

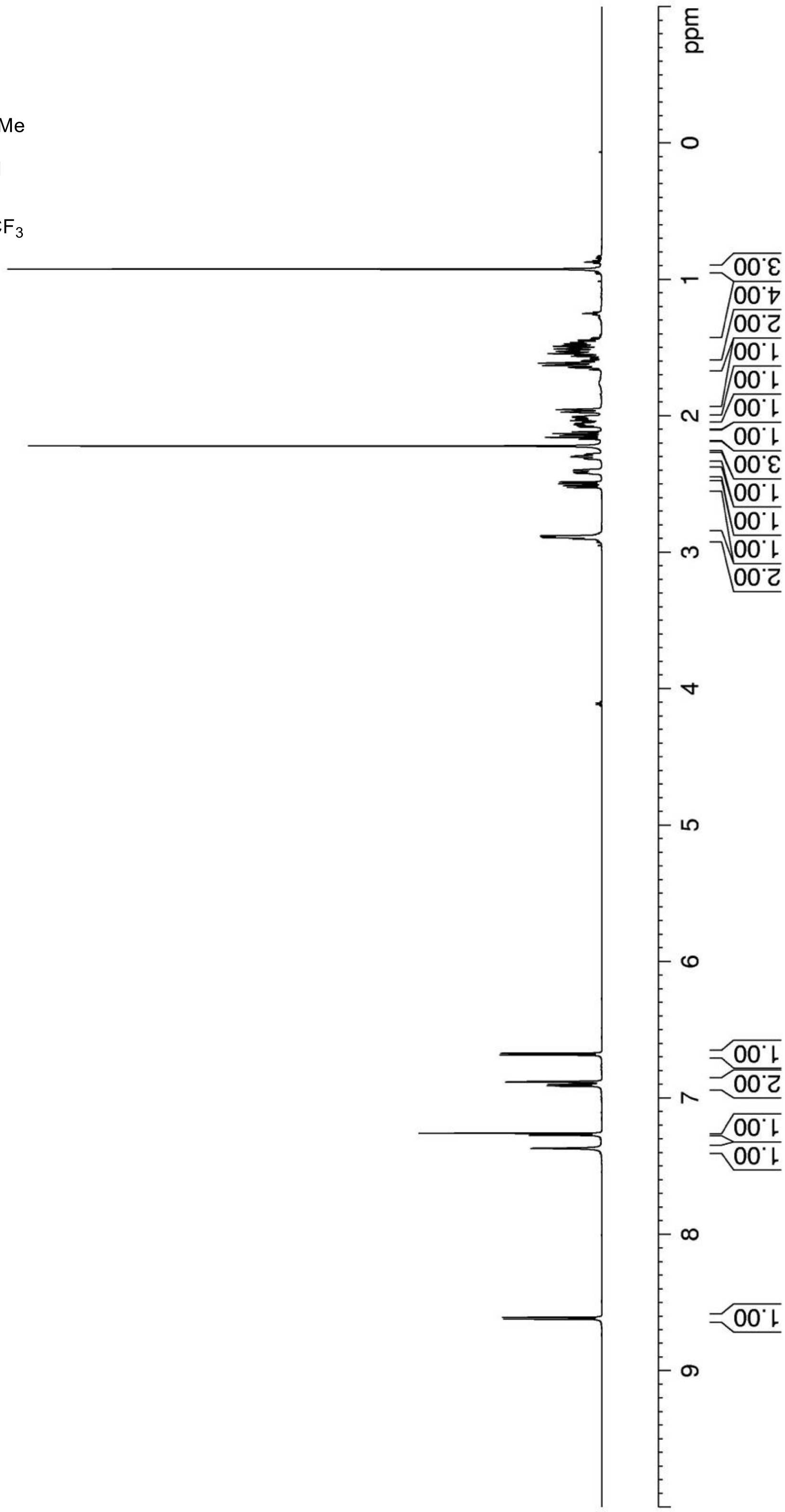

^{13}C NMR ($CDCl_3$, 25 ºC) of **2x**

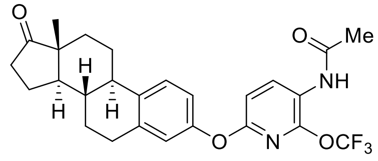

2x

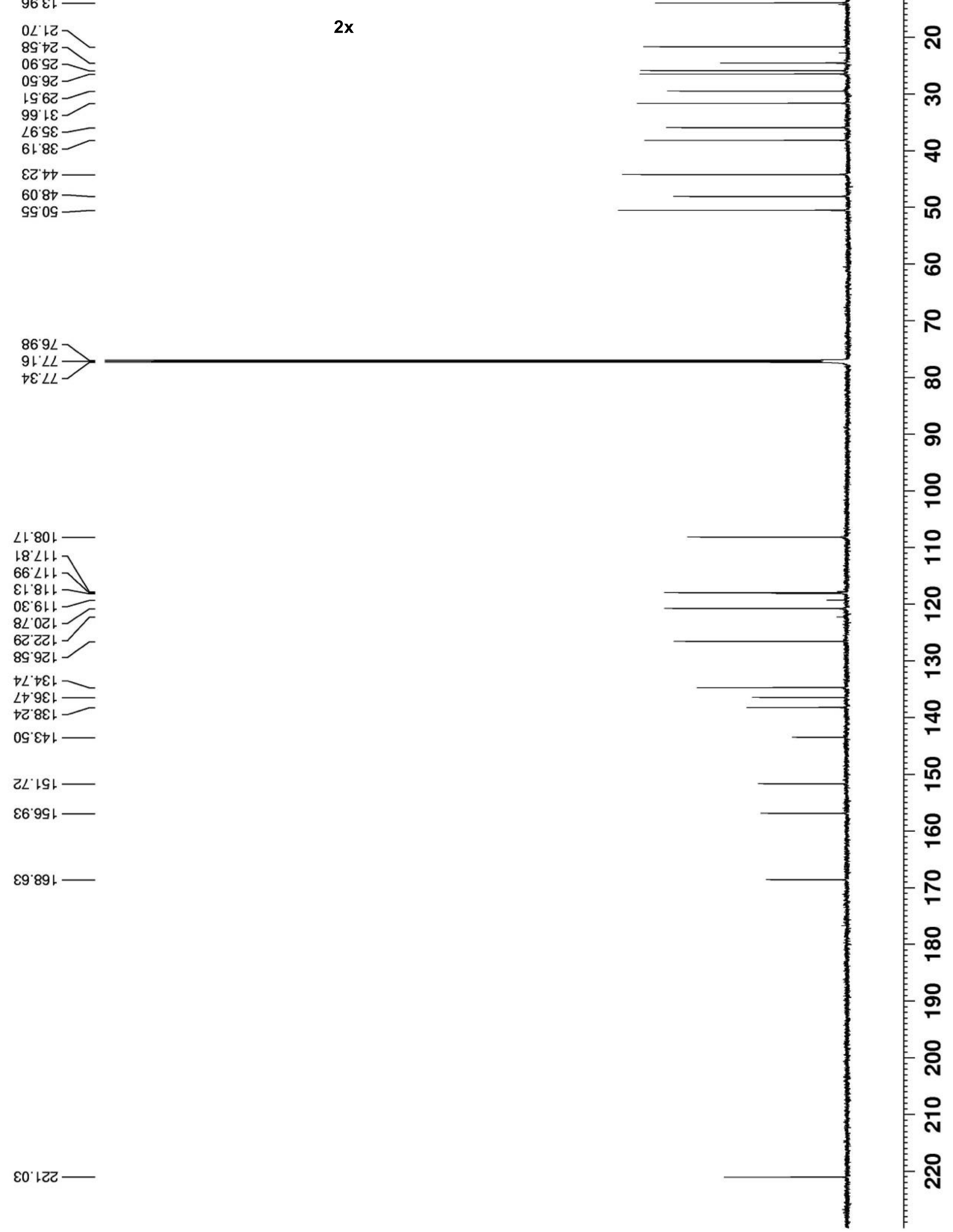

^{19}F NMR ($CDCl_3$, 25 ºC) of **2x**

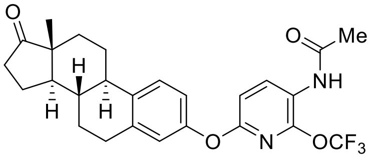

2x

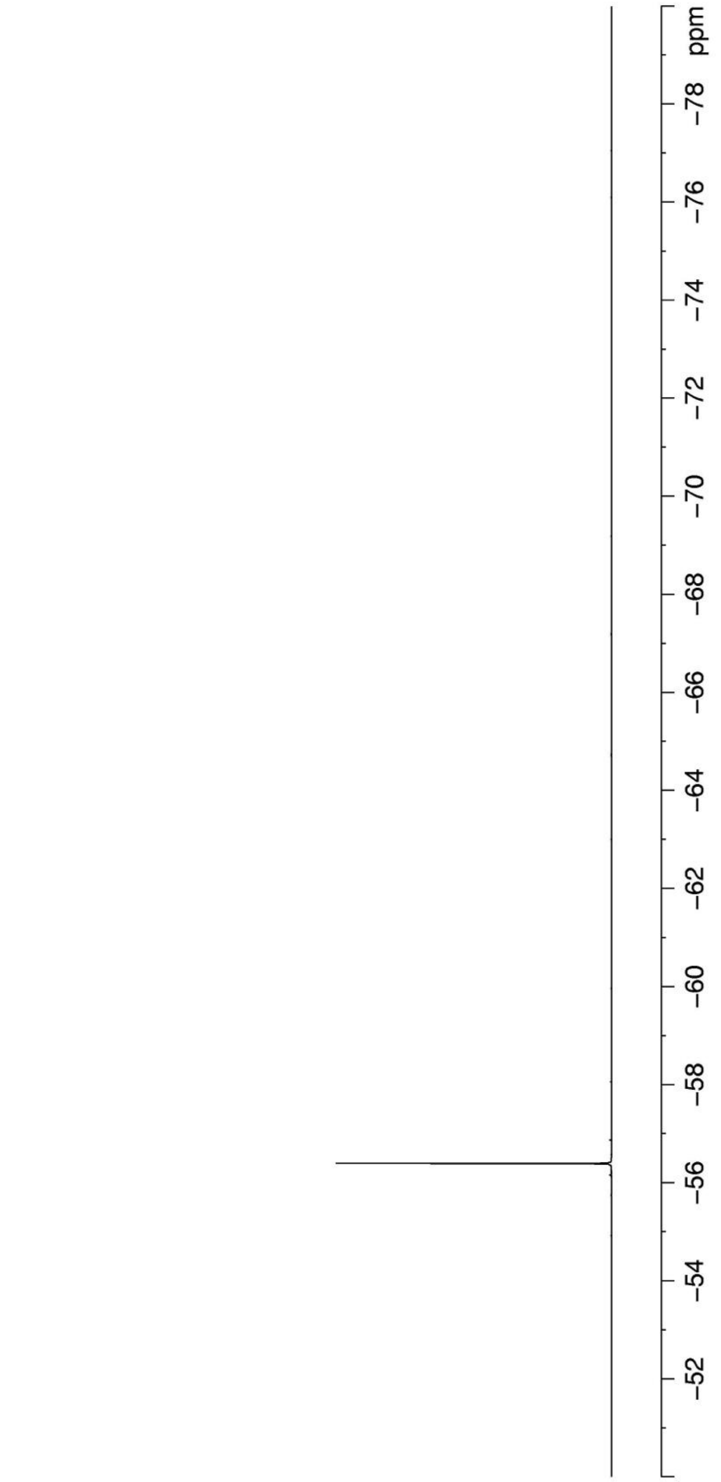

^{1}H NMR ($(CD_3)_2SO$, 25 ºC) of **2y**

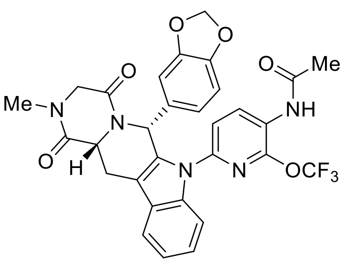

2y

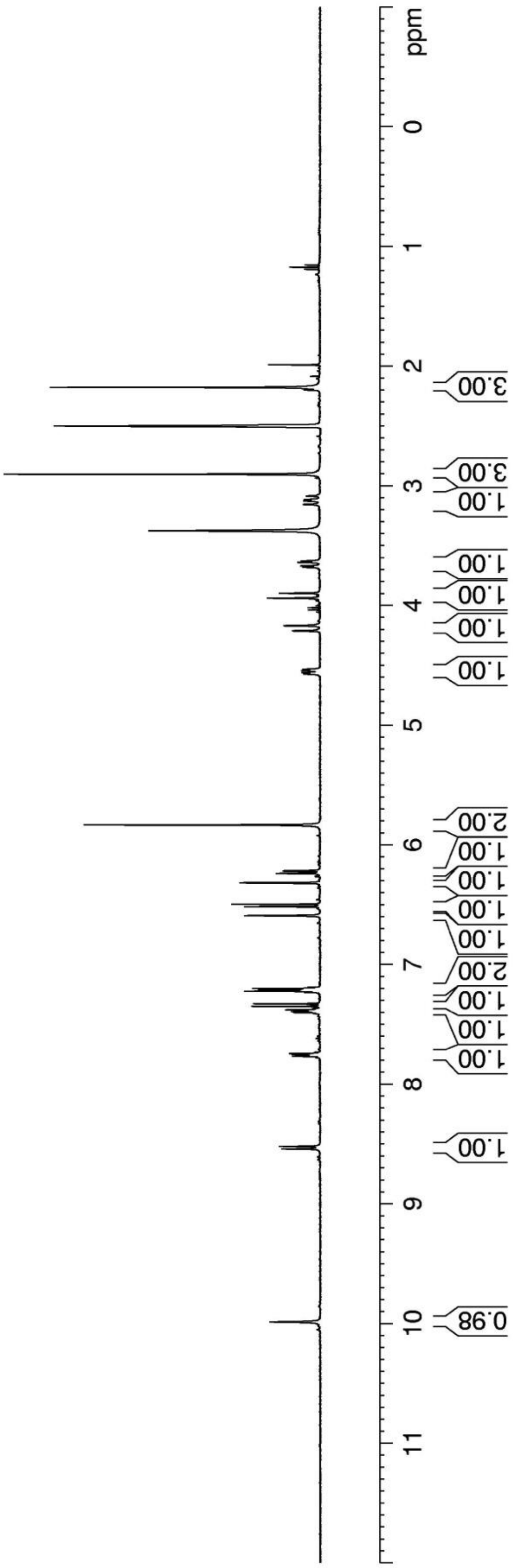

^{13}C NMR ($(CD_3)_2SO$, 25 ºC) of **2y**

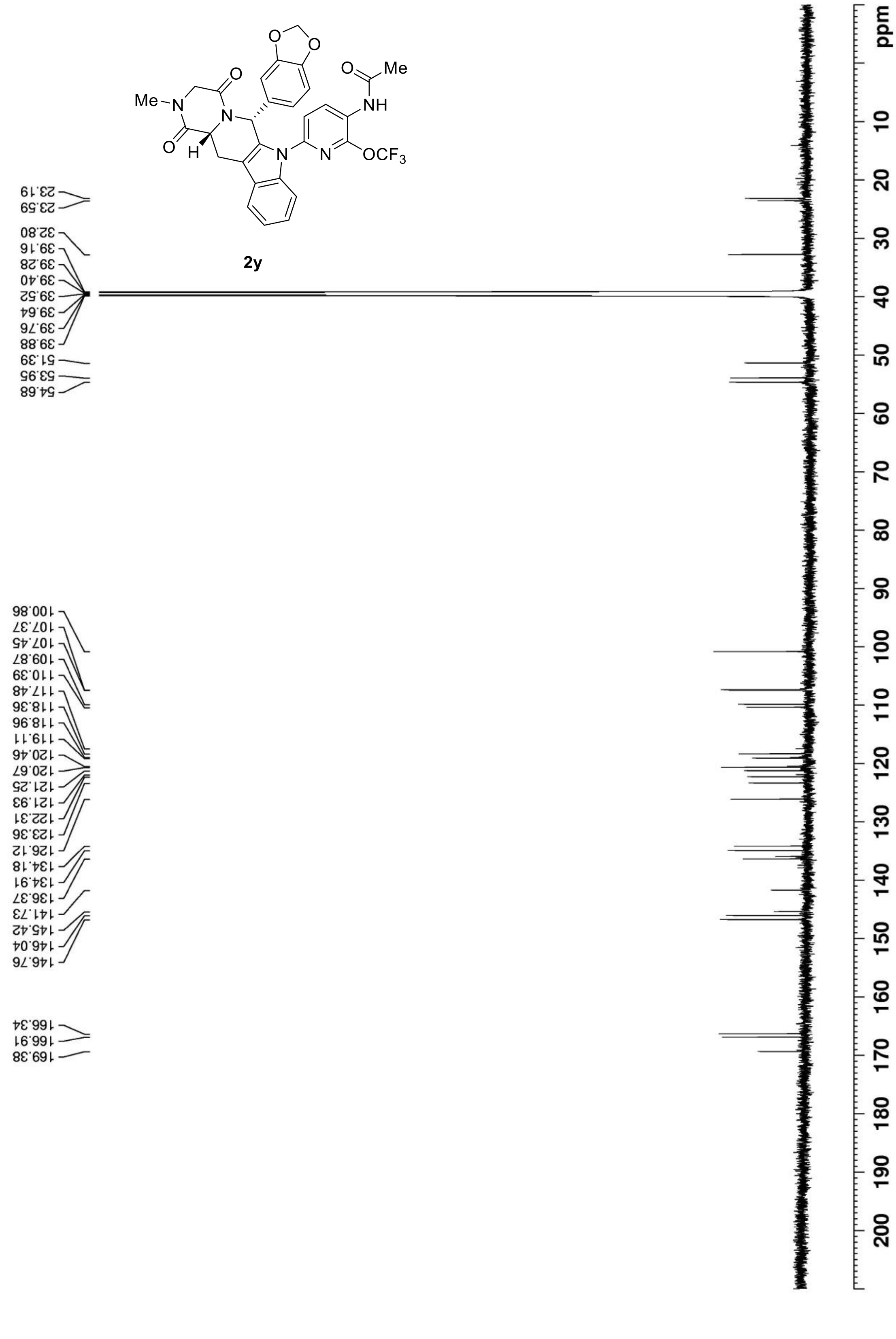

^{19}F NMR ((CD$_3$)$_2$SO, 25 ºC) of **2y**

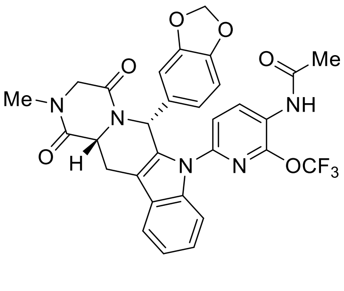

2y

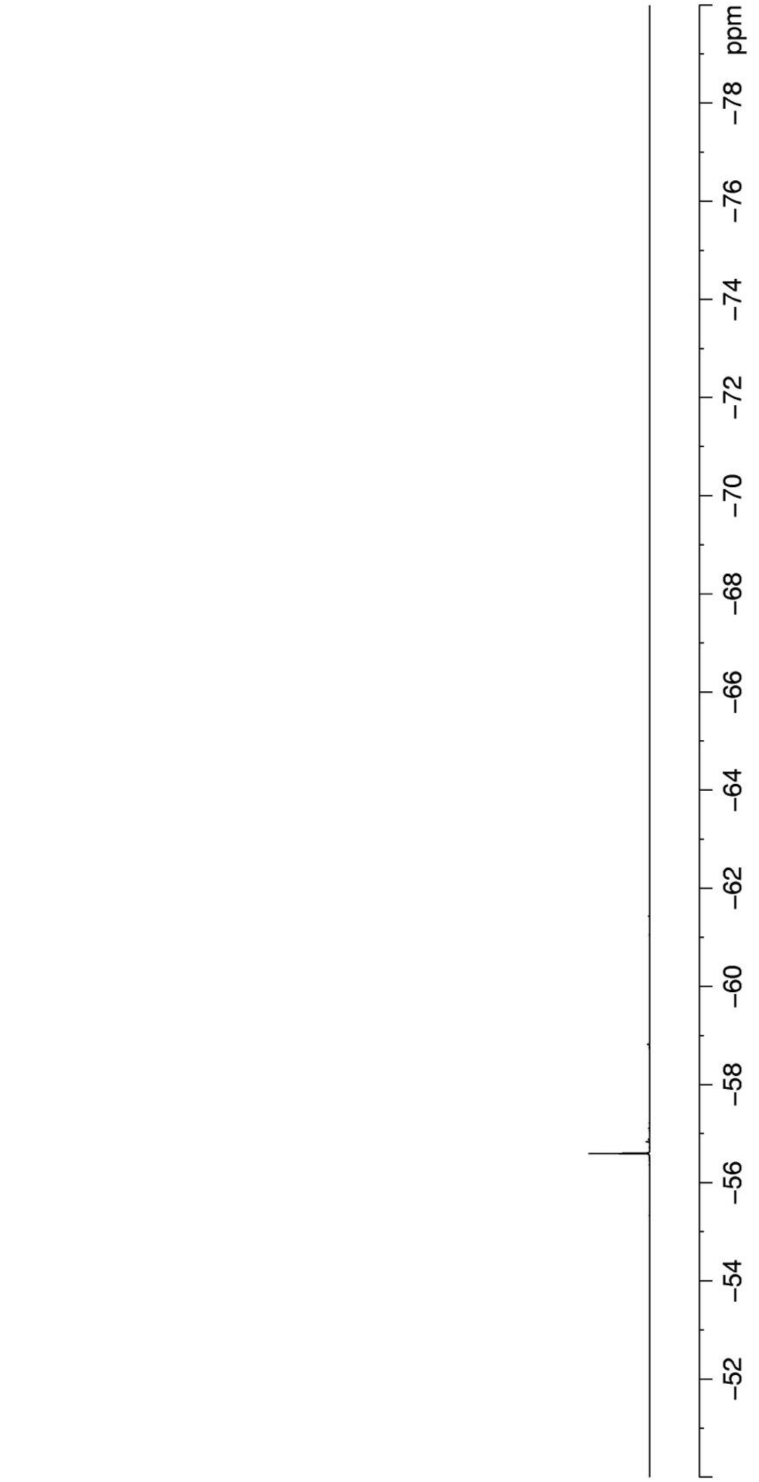

^{1}H NMR ($CDCl_3$, 25 ºC) of **4a**

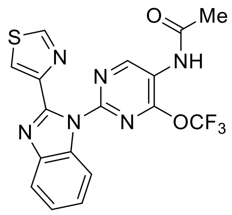

4a

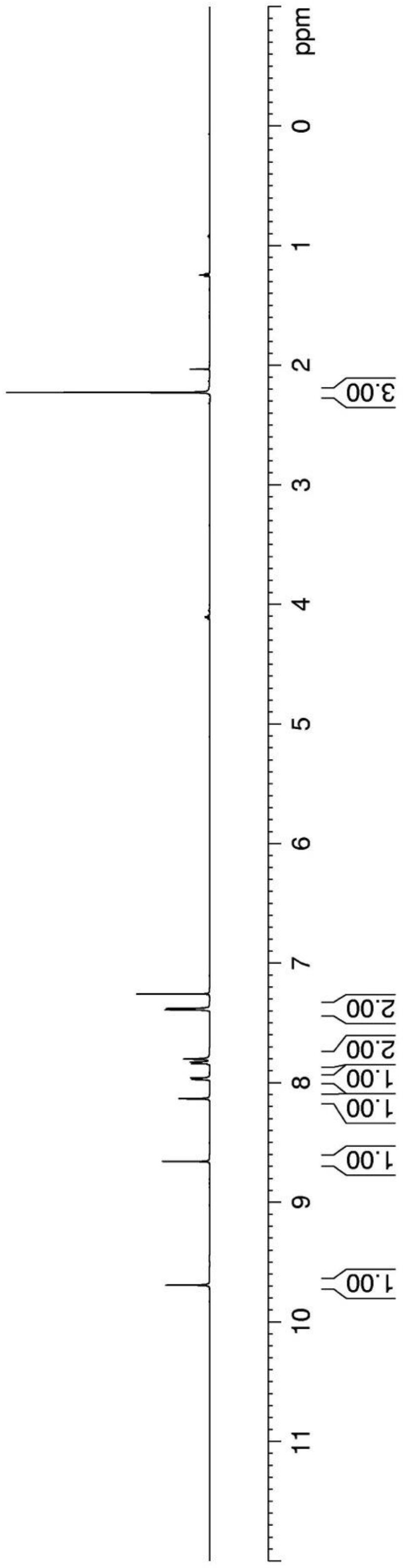

^{13}C NMR ($CDCl_3$, 25 ºC) of **4a**

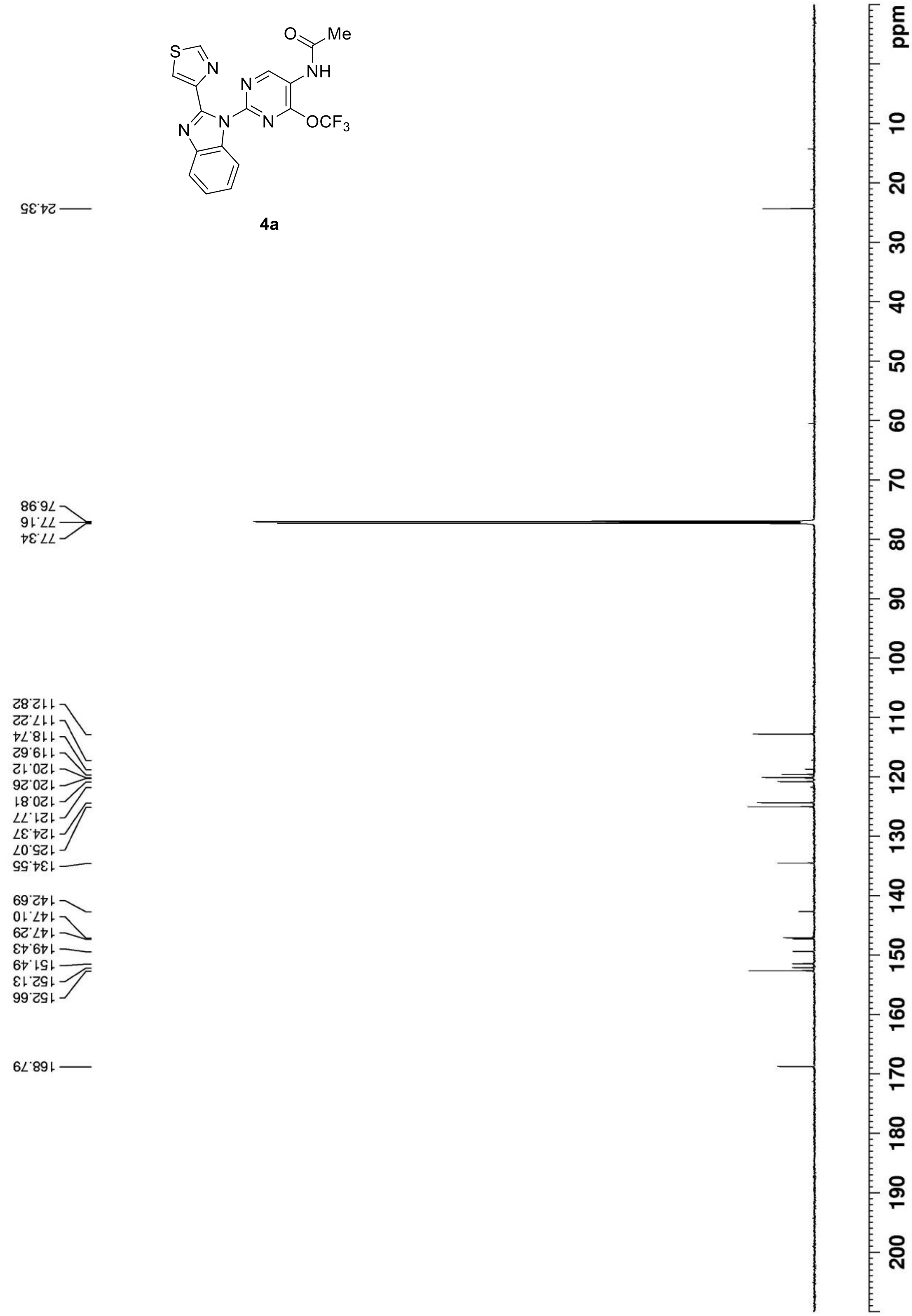

^{19}F NMR ($CDCl_3$, 25 ºC) of **4a**

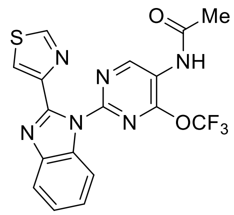

4a

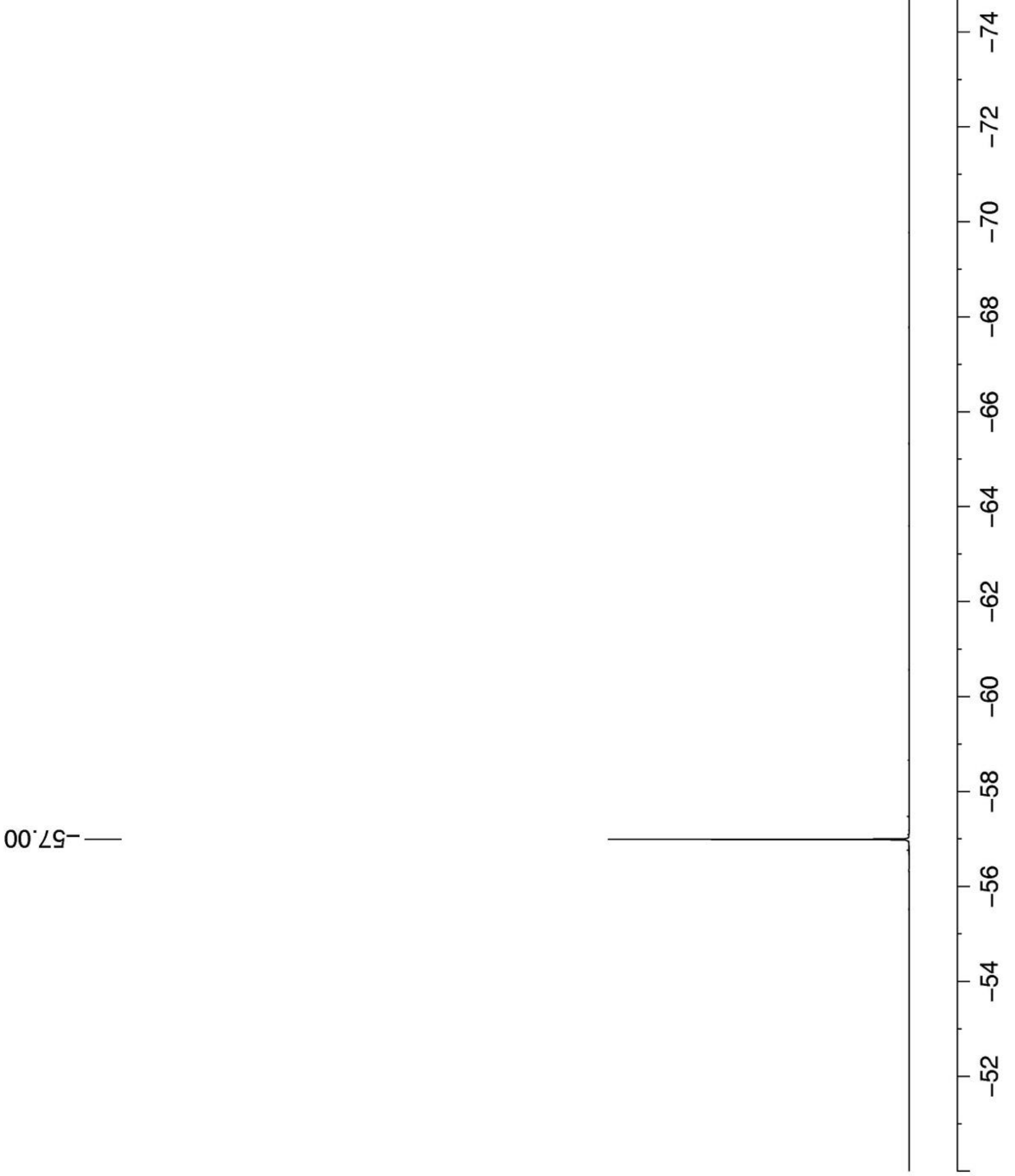

^{1}H NMR ($CDCl_3$, 25 ºC) of **4b**

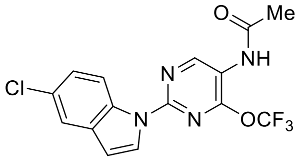

4b

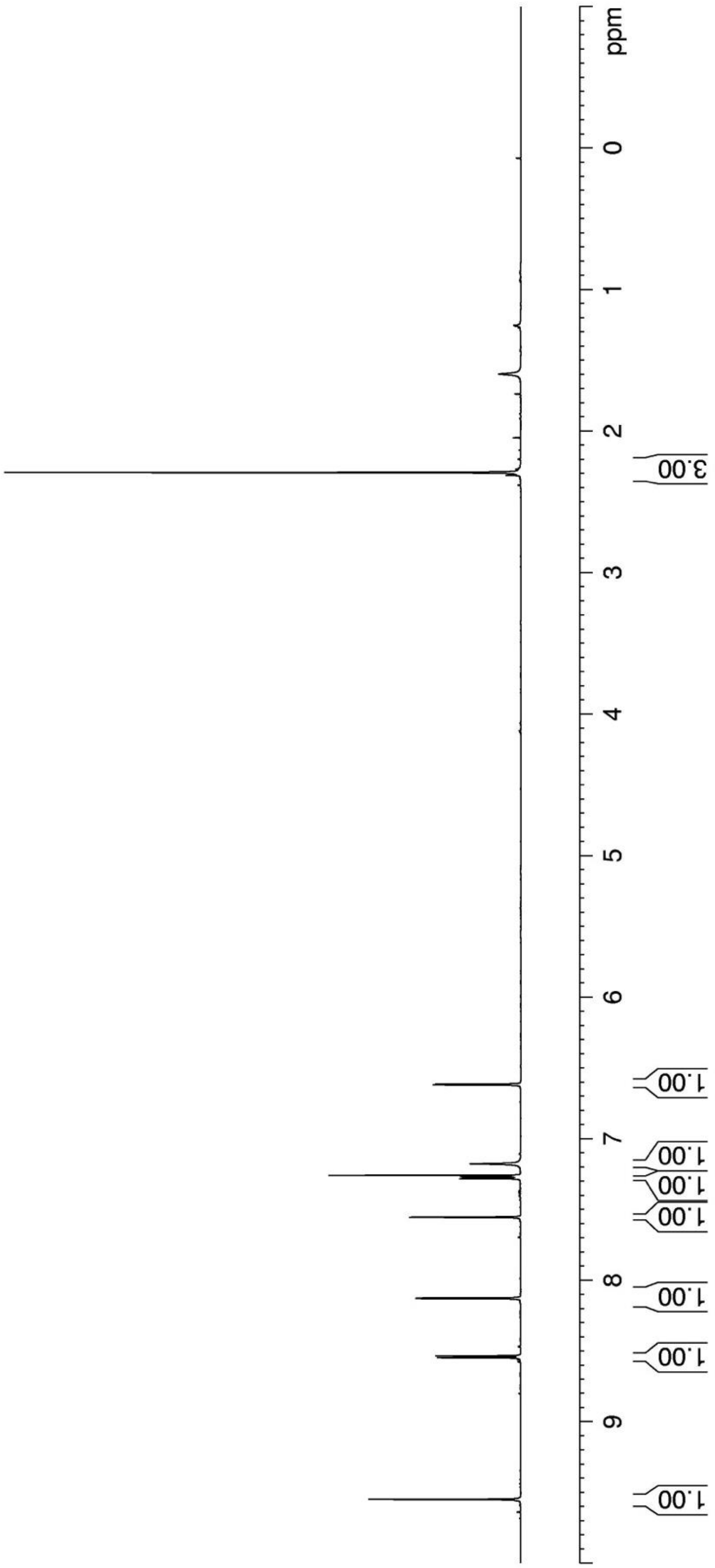

^{13}C NMR ($CDCl_3$, 25 ºC) of **4b**

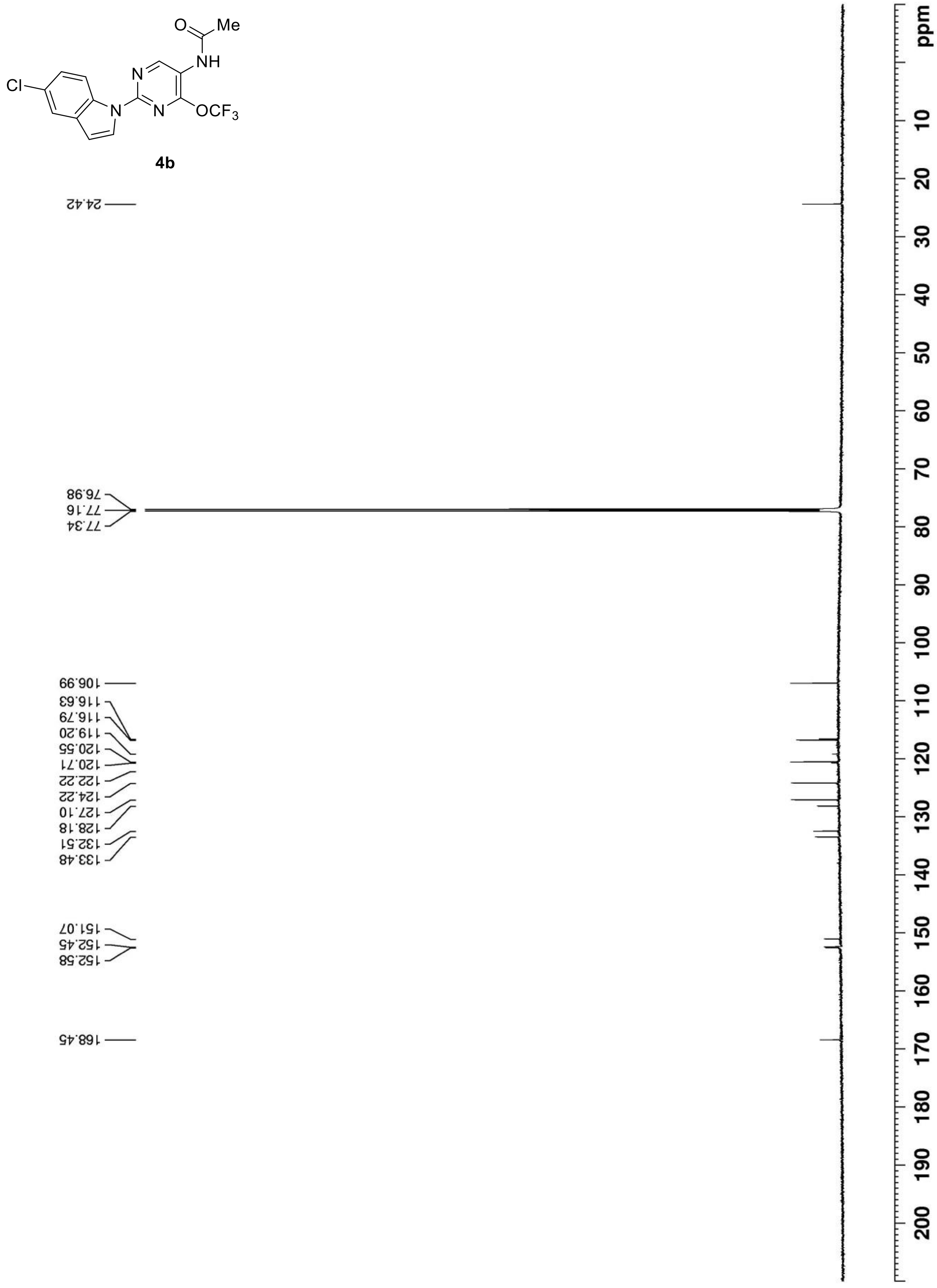

^{19}F NMR ($CDCl_3$, 25 ºC) of **4b**

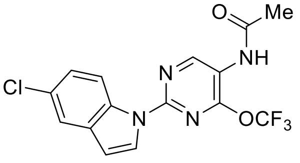

4b

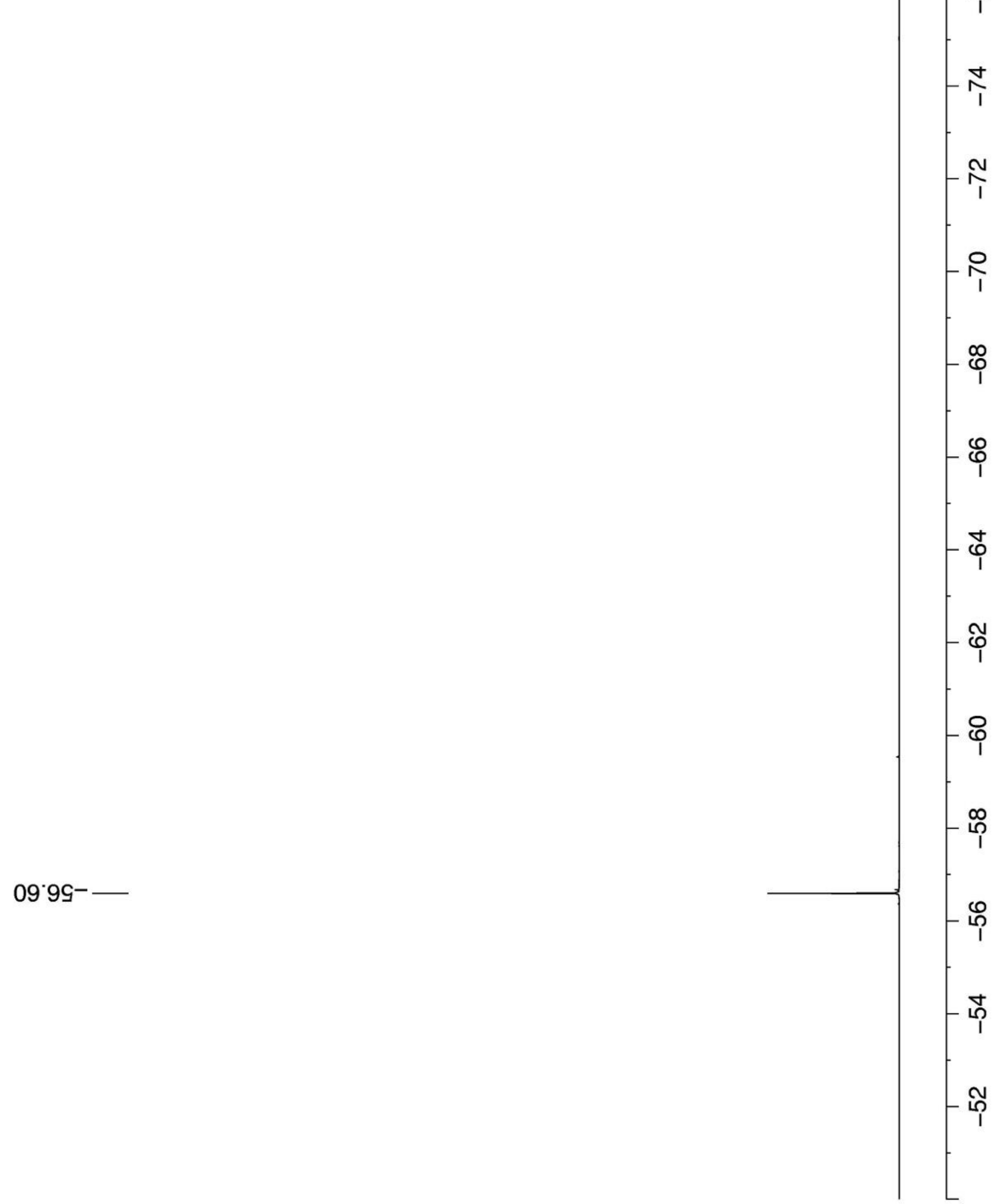

^{1}H NMR ($CDCl_3$, 25 ºC) of **4c**

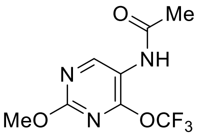

4c

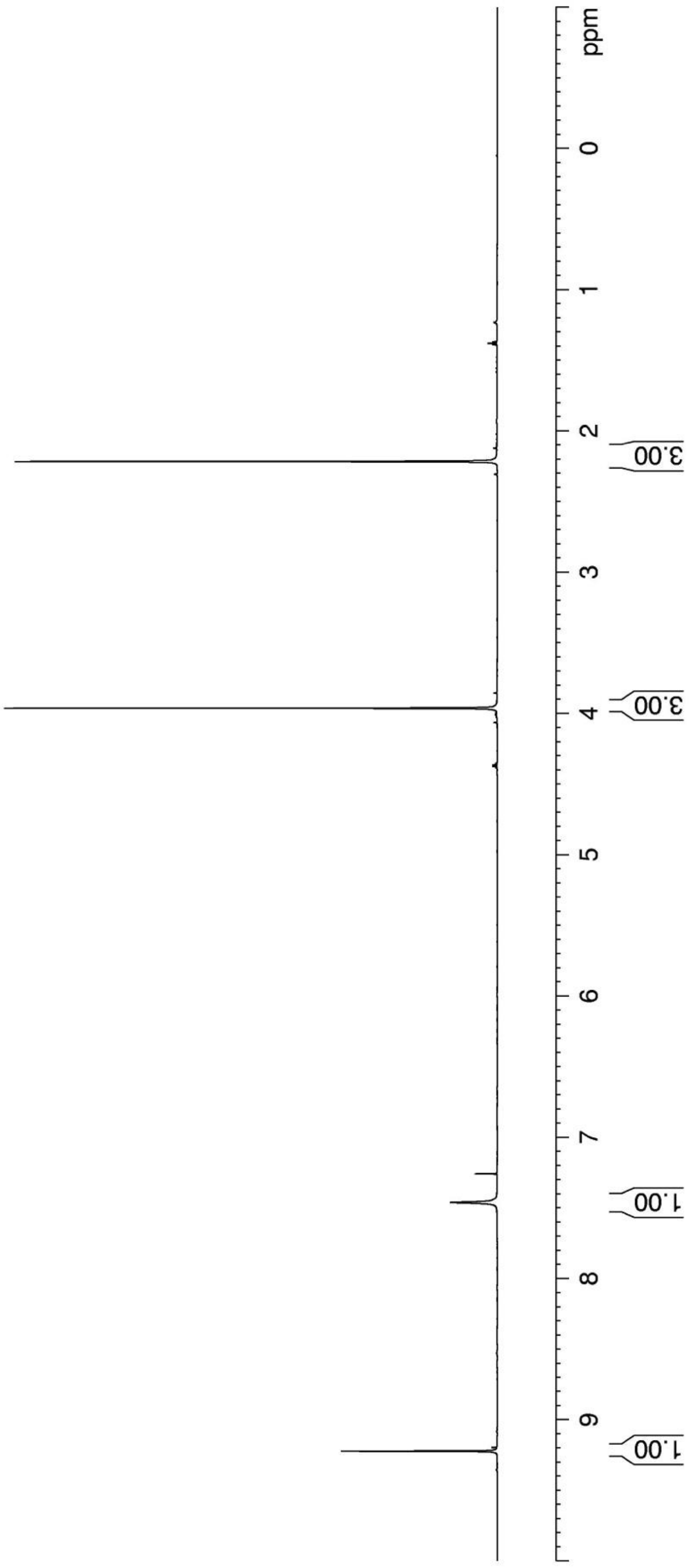

^{13}C NMR ($CDCl_3$, 25 ºC) of **4c**

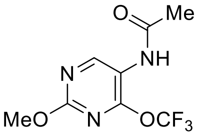

4c

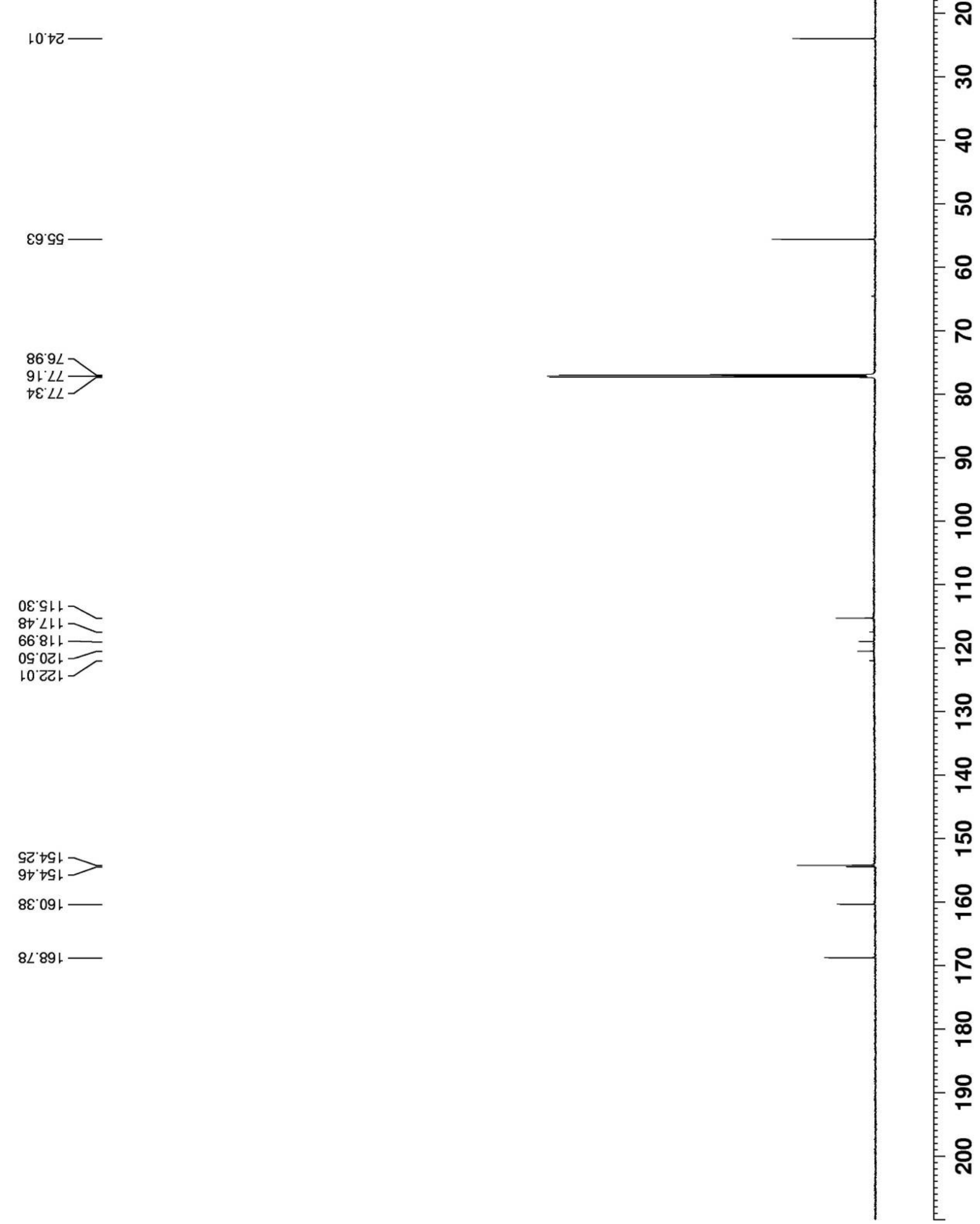

^{19}F NMR ($CDCl_3$, 25 ºC) of **4c**

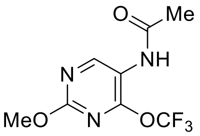

4c

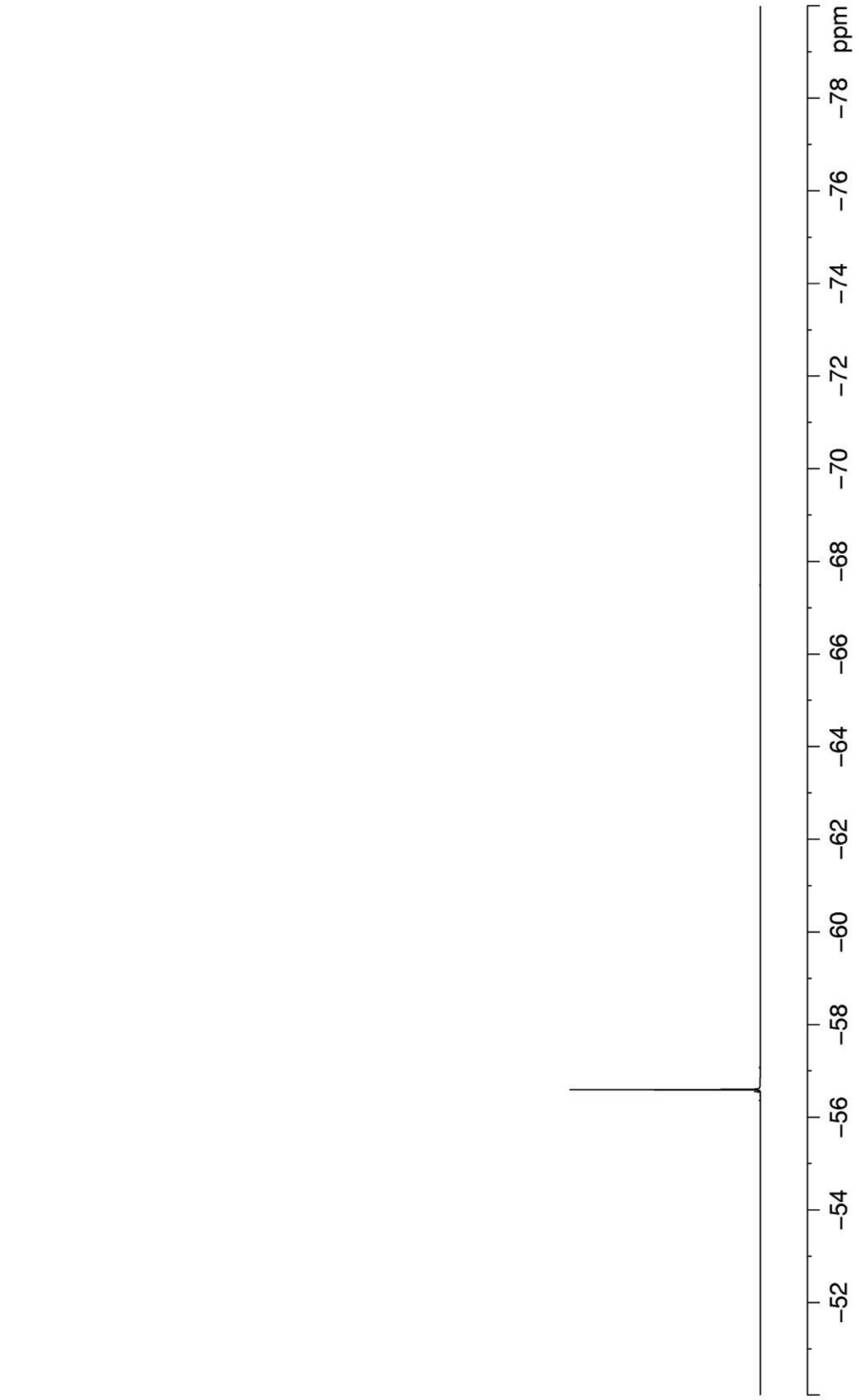

^{1}H NMR ($CDCl_3$, 25 ºC) of **4d**

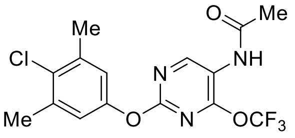

4d

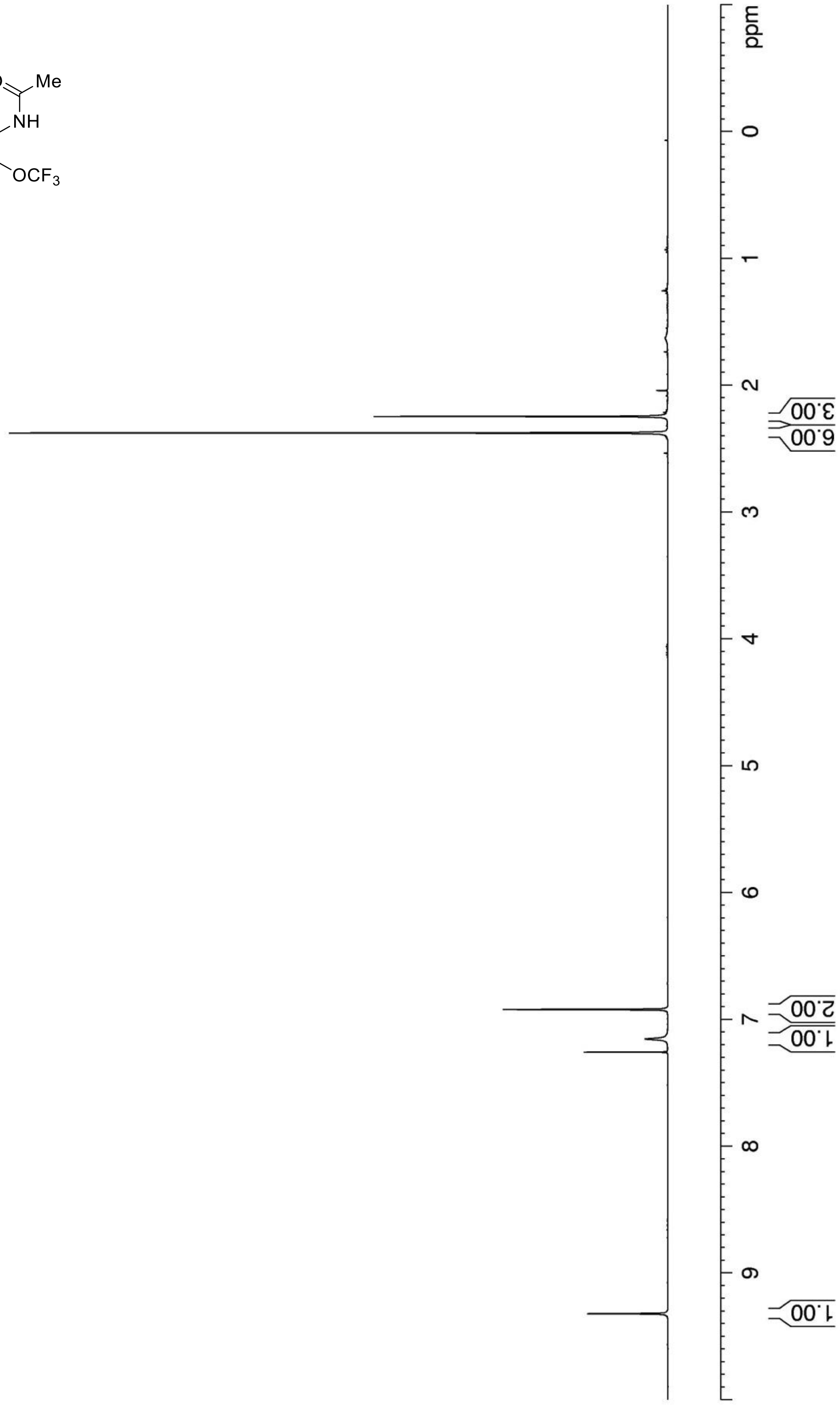

^{13}C NMR ($CDCl_3$, 25 ºC) of **4d**

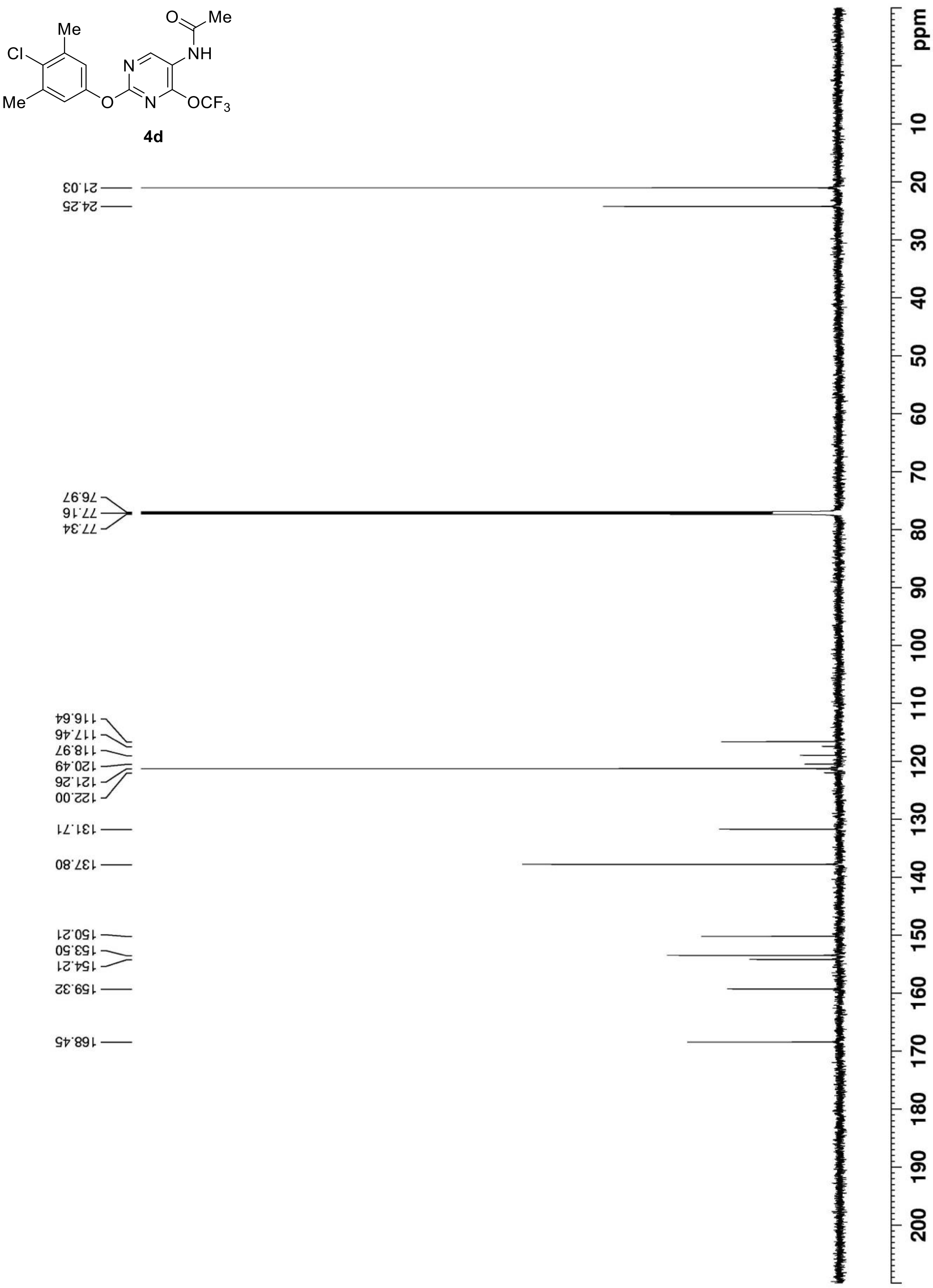

^{19}F NMR ($CDCl_3$, 25 ºC) of **4d**

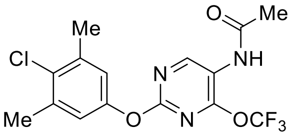

4d

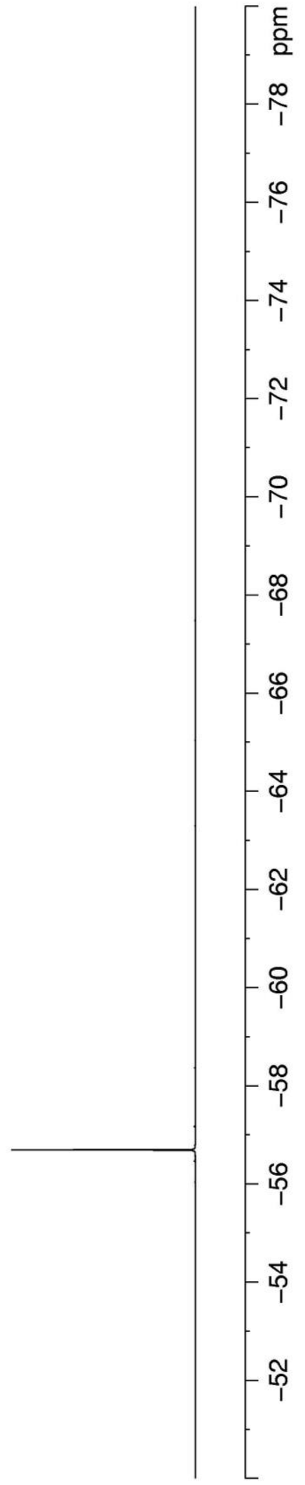

^{1}H NMR ($CDCl_3$, 25 ºC) of **4e**

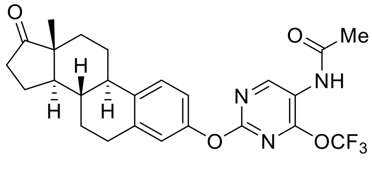

4e

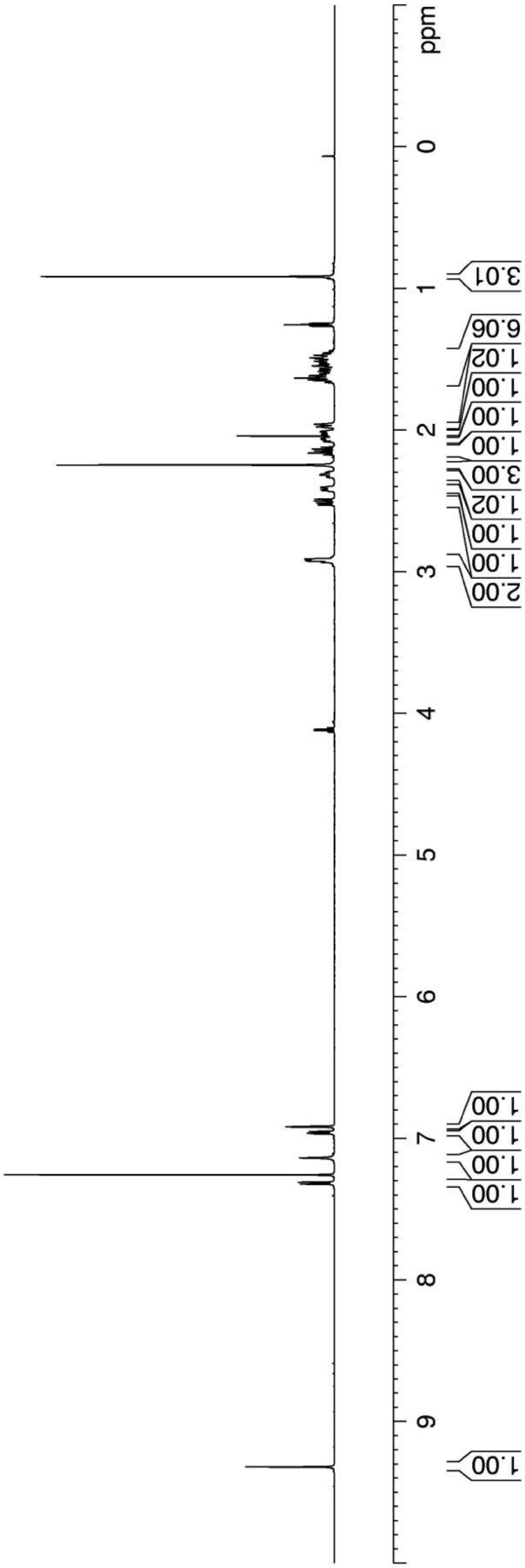

^{13}C NMR ($CDCl_3$, 25 ºC) of **4e**

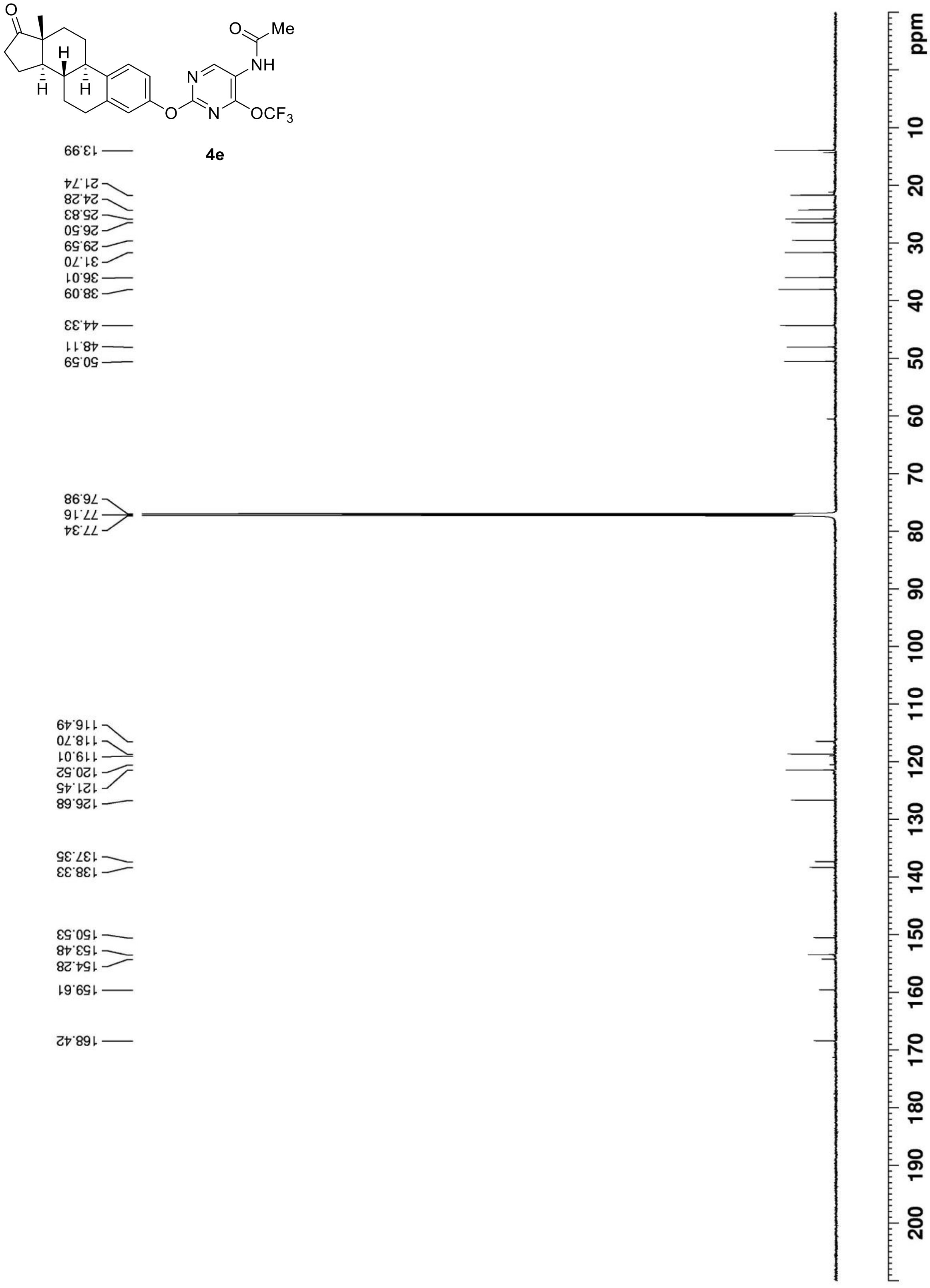

^{19}F NMR ($CDCl_3$, 25 ºC) of **4e**

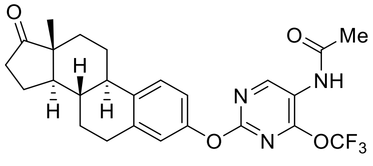

4e

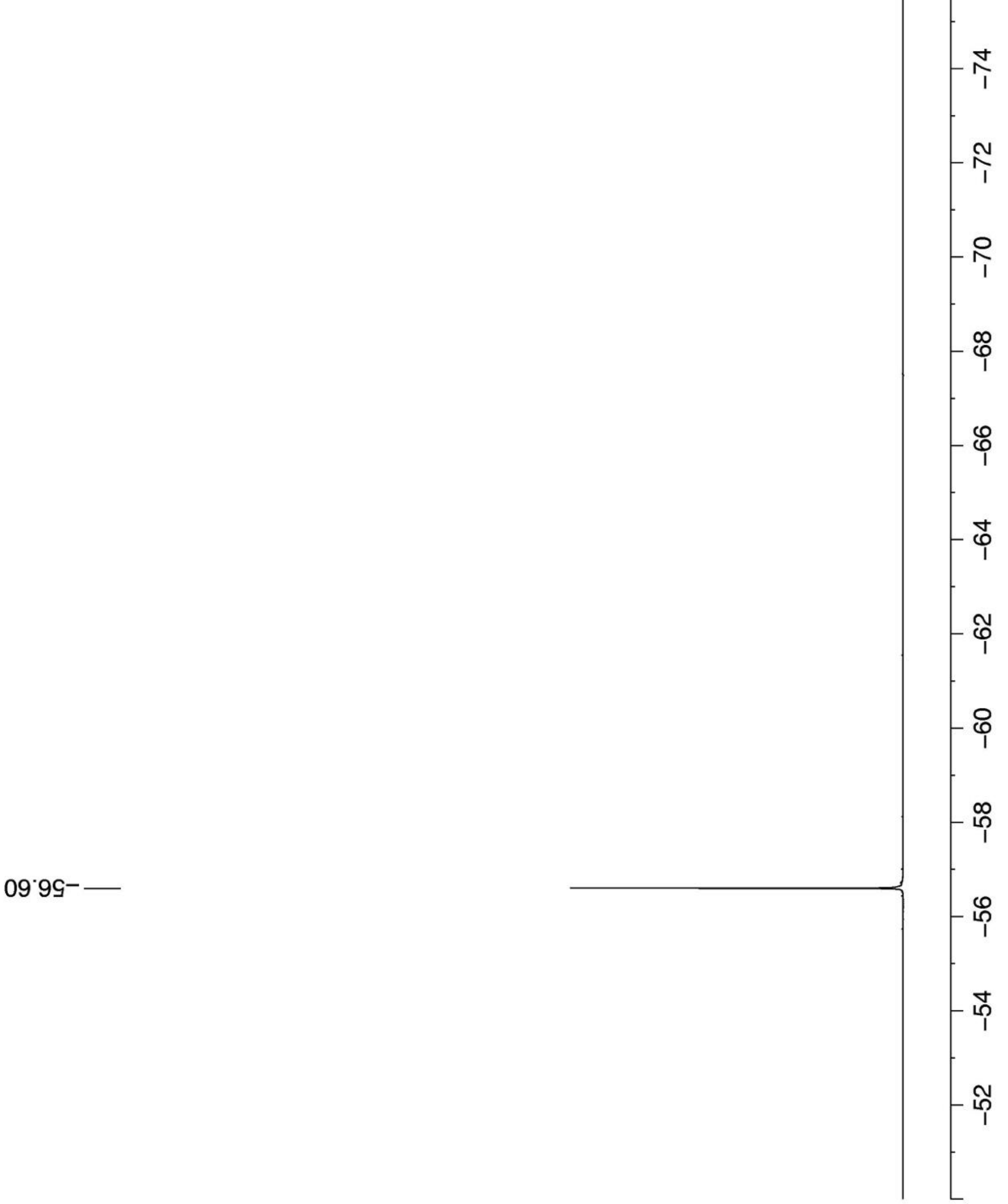

^{1}H NMR ($CDCl_3$, 25 ºC) of **1d’**

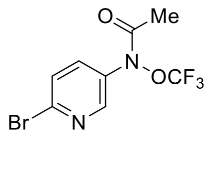

1d'

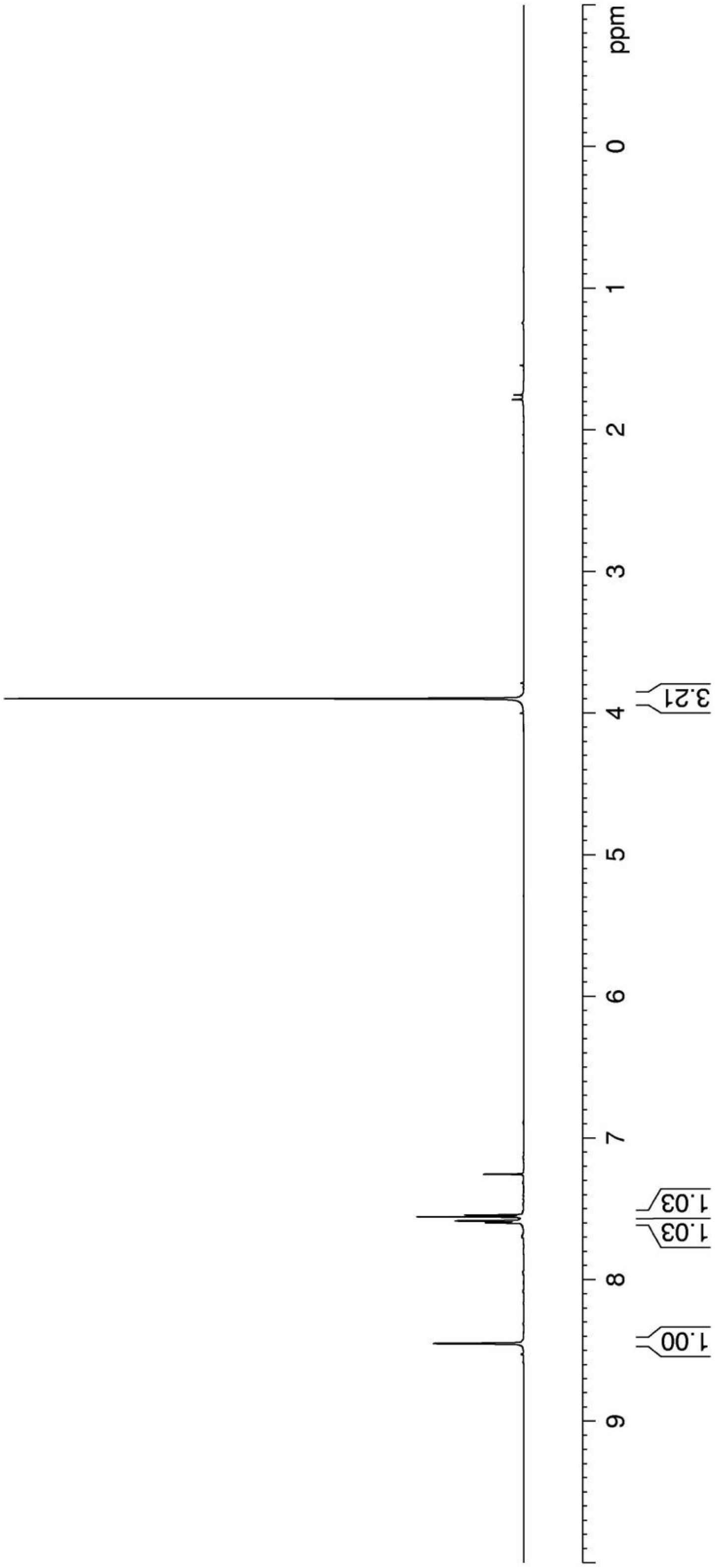

^{13}C NMR ($CDCl_3$, 25 ºC) of **1d’**

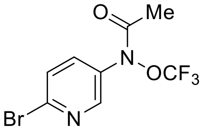

1d'

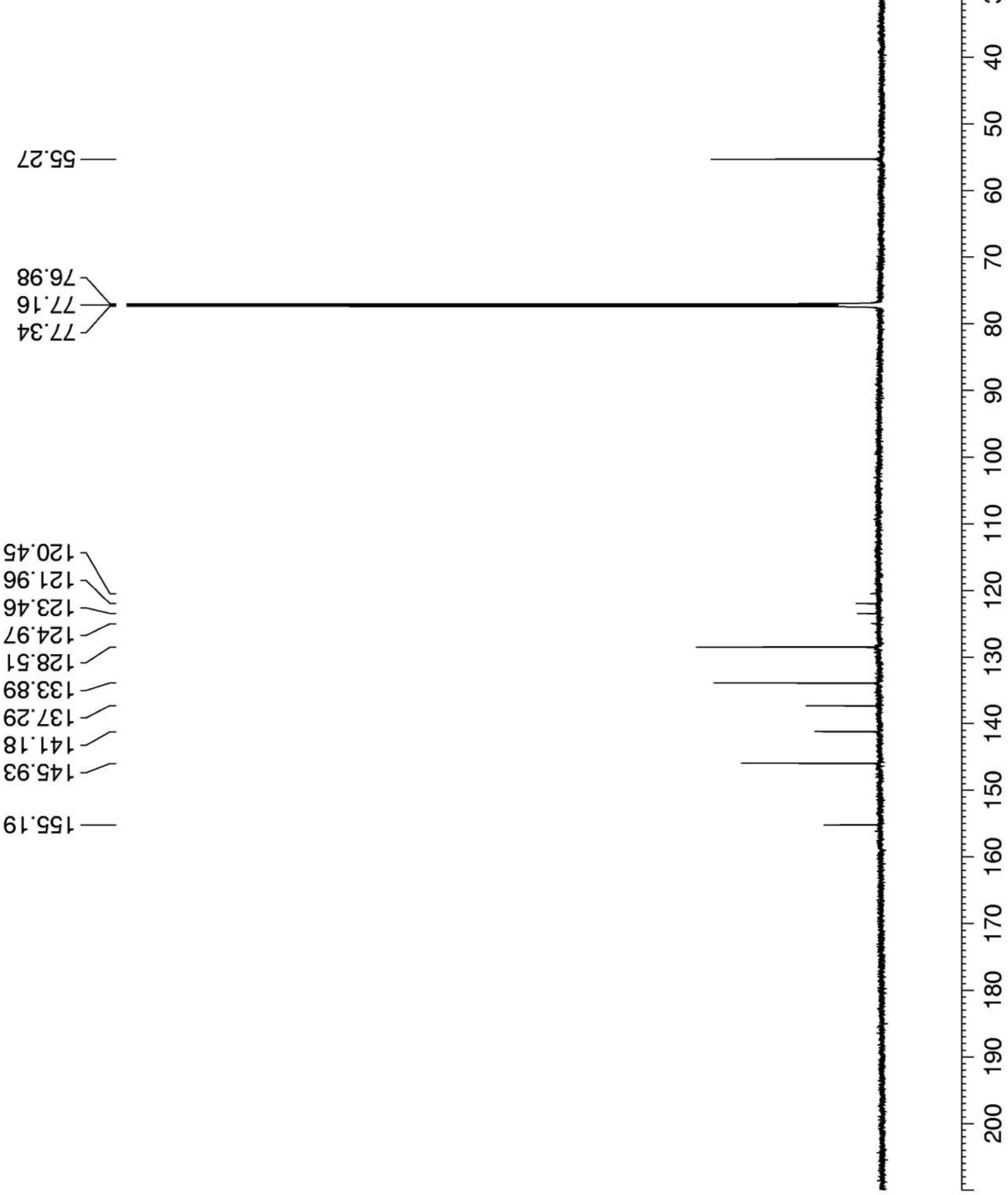

^{19}F NMR ($CDCl_3$, 25 ºC) of **1d’**

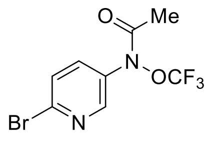

1d'

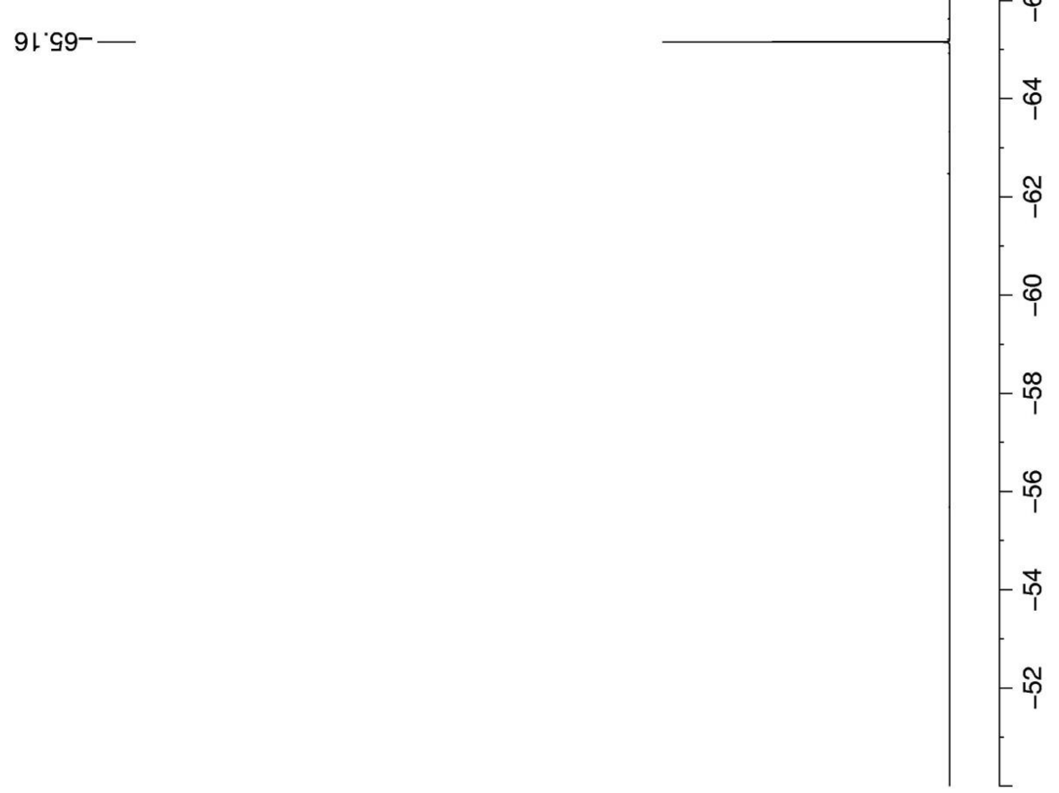

^1H NMR ($CDCl_3$, 25 ºC) of **2a’**

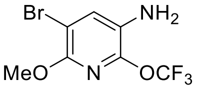

2a'

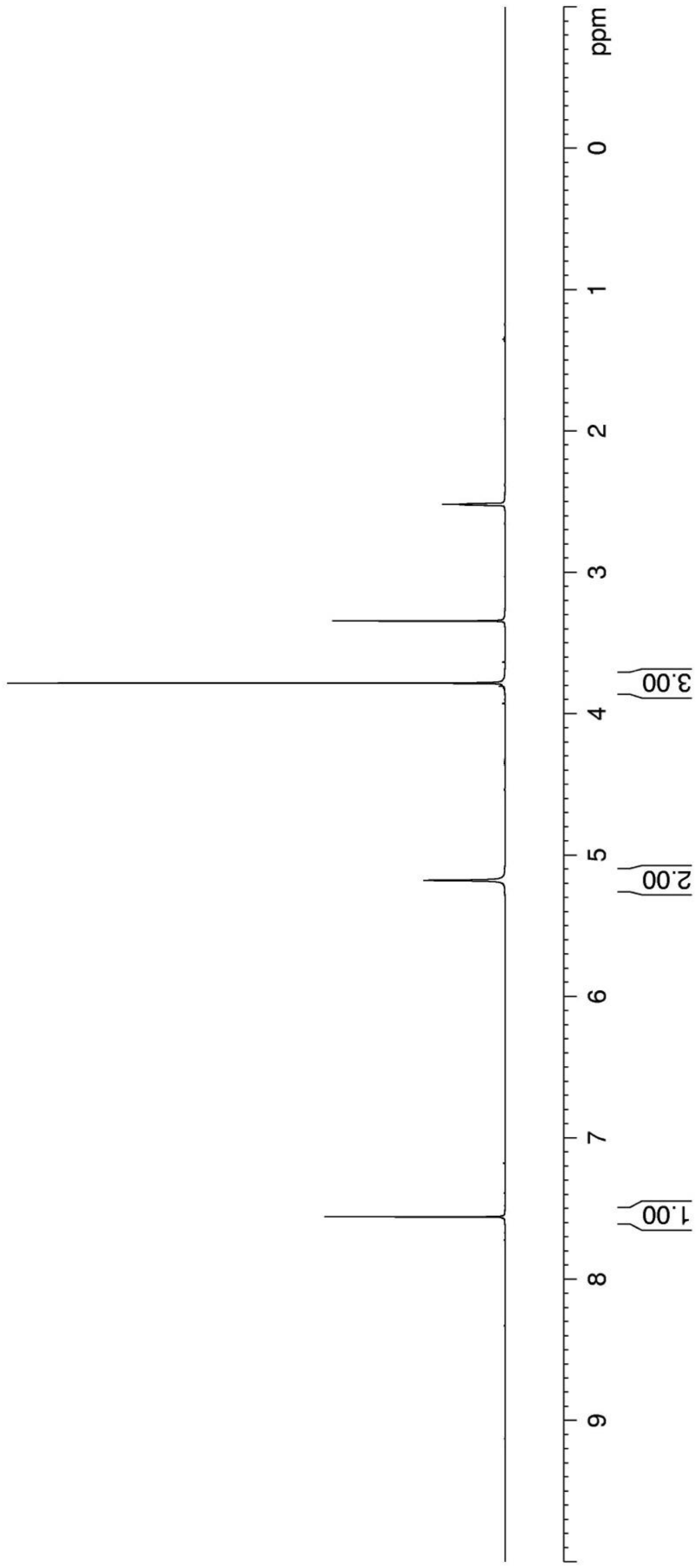

^{13}C NMR ($CDCl_3$, 25 ºC) of **2a'**

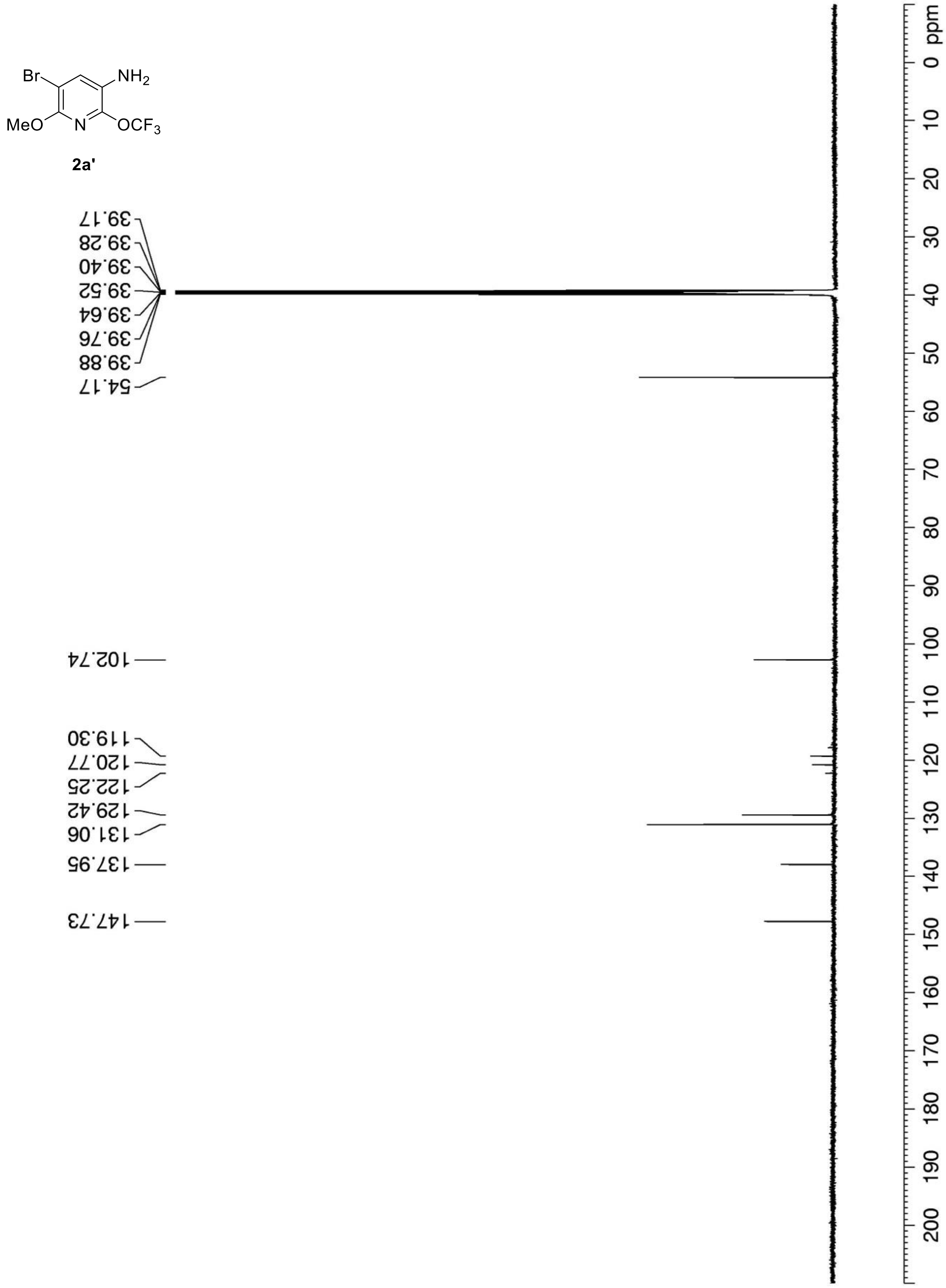

^{19}F NMR ($CDCl_3$, 25 ºC) of **2a’**

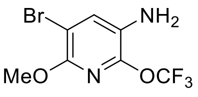

2a'

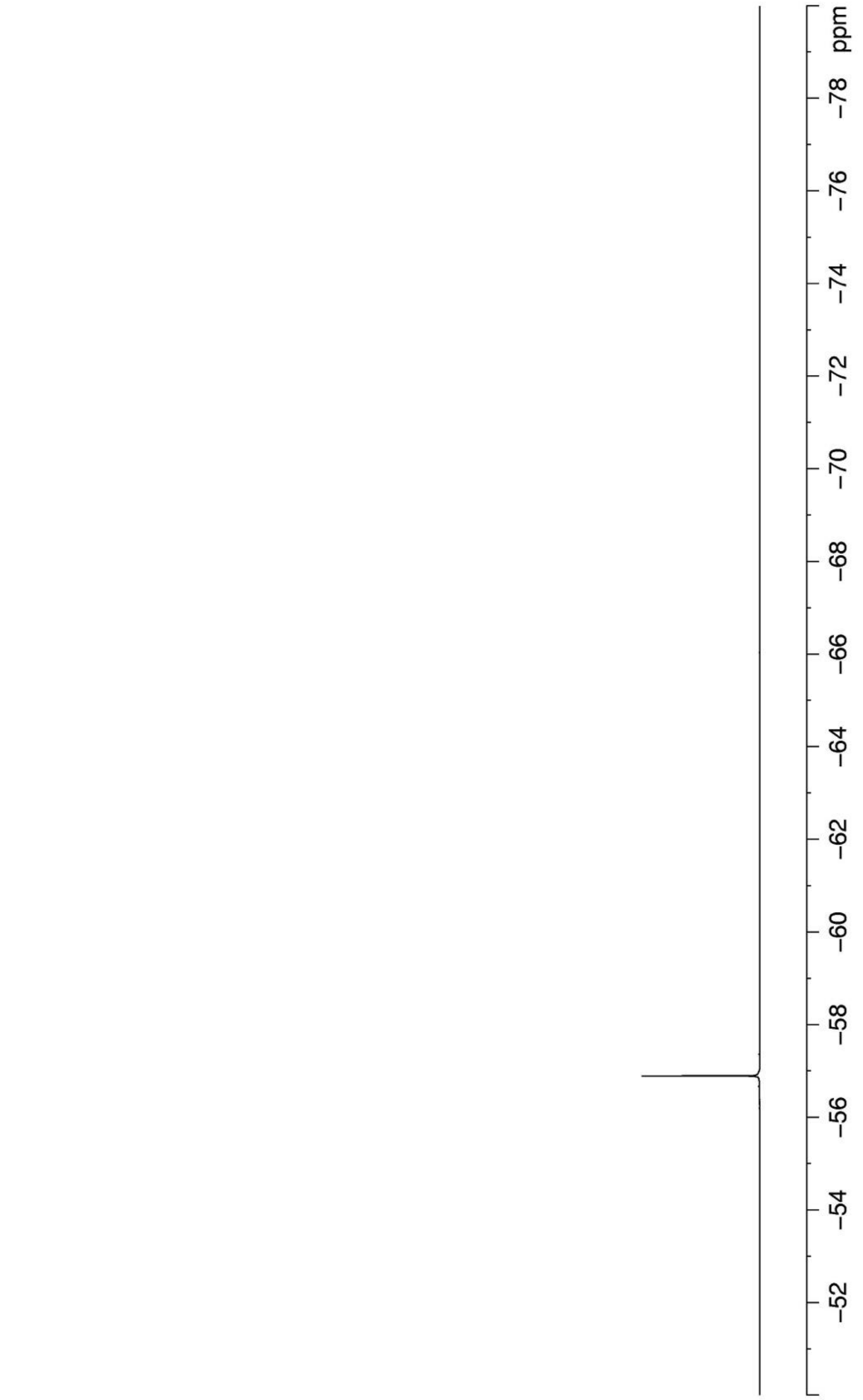

^{1}H NMR ($CDCl_3$, 25 ºC) of **5a**

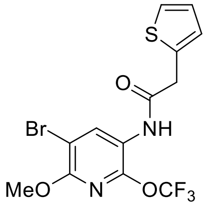

5a

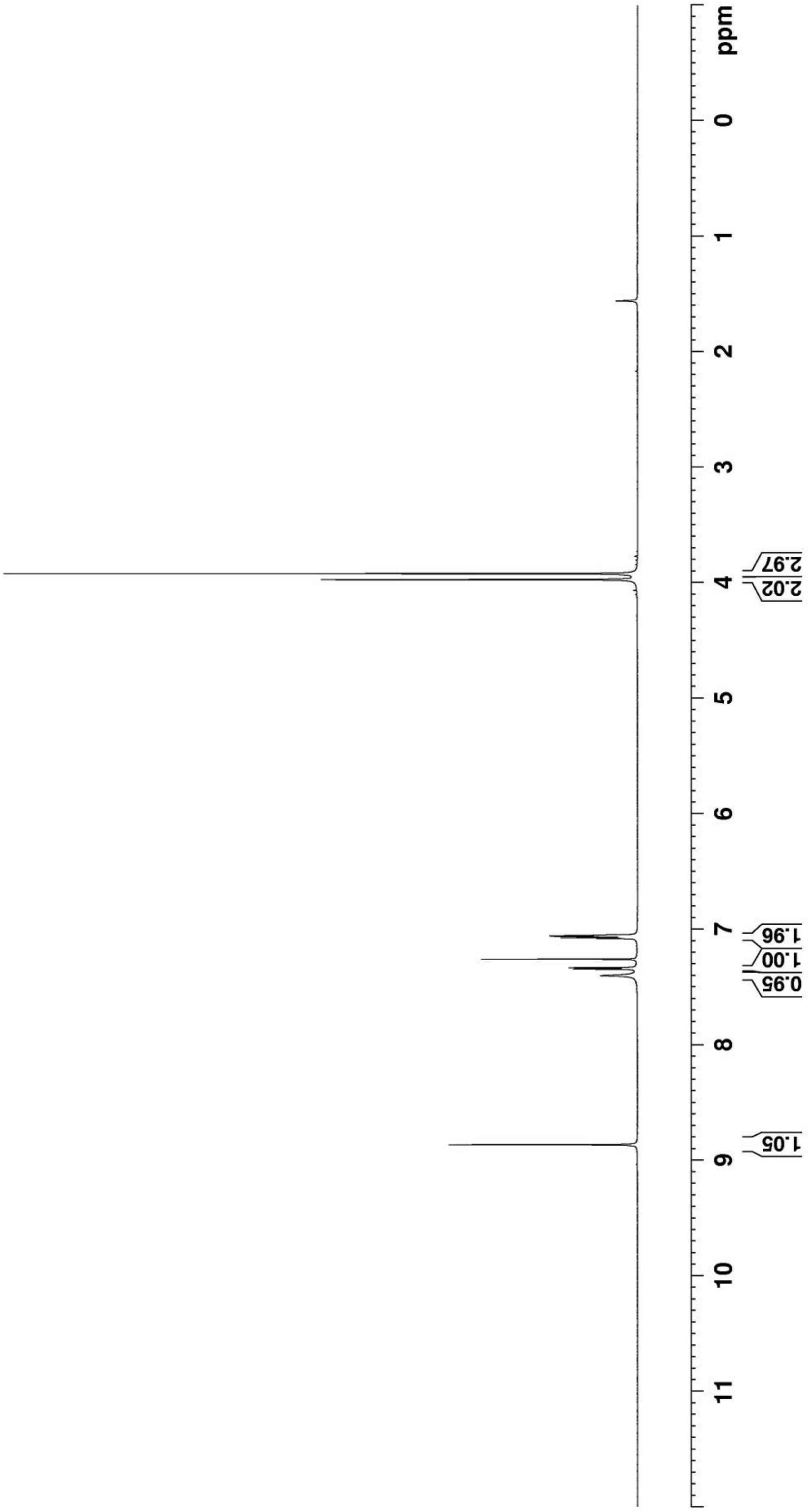

^{13}C NMR ($CDCl_3$, 25 ºC) of **5a**

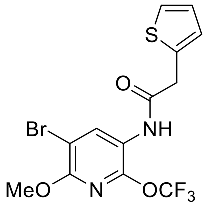

5a

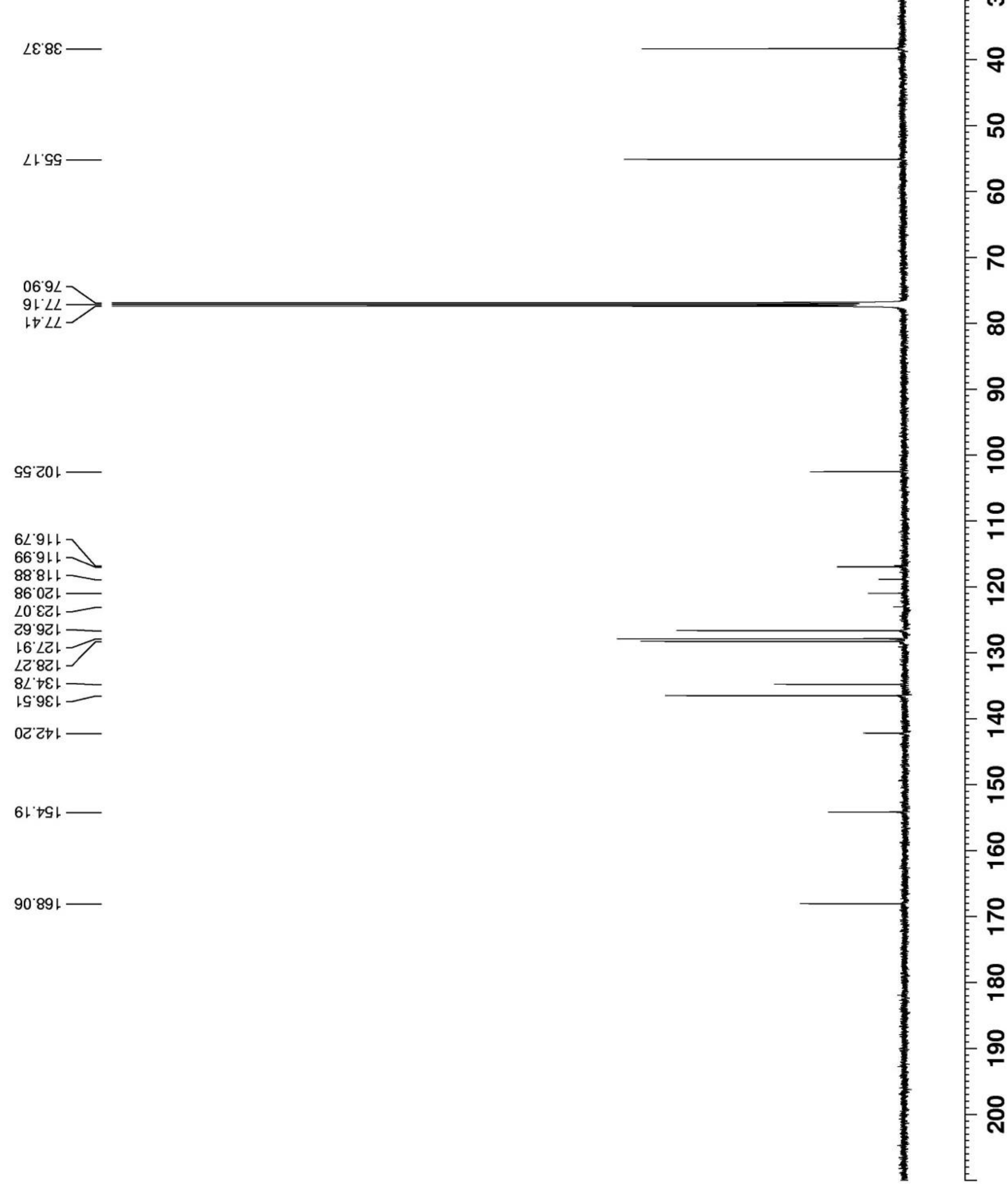

^{19}F NMR ($CDCl_3$, 25 ºC) of **5a**

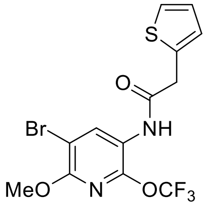

5a

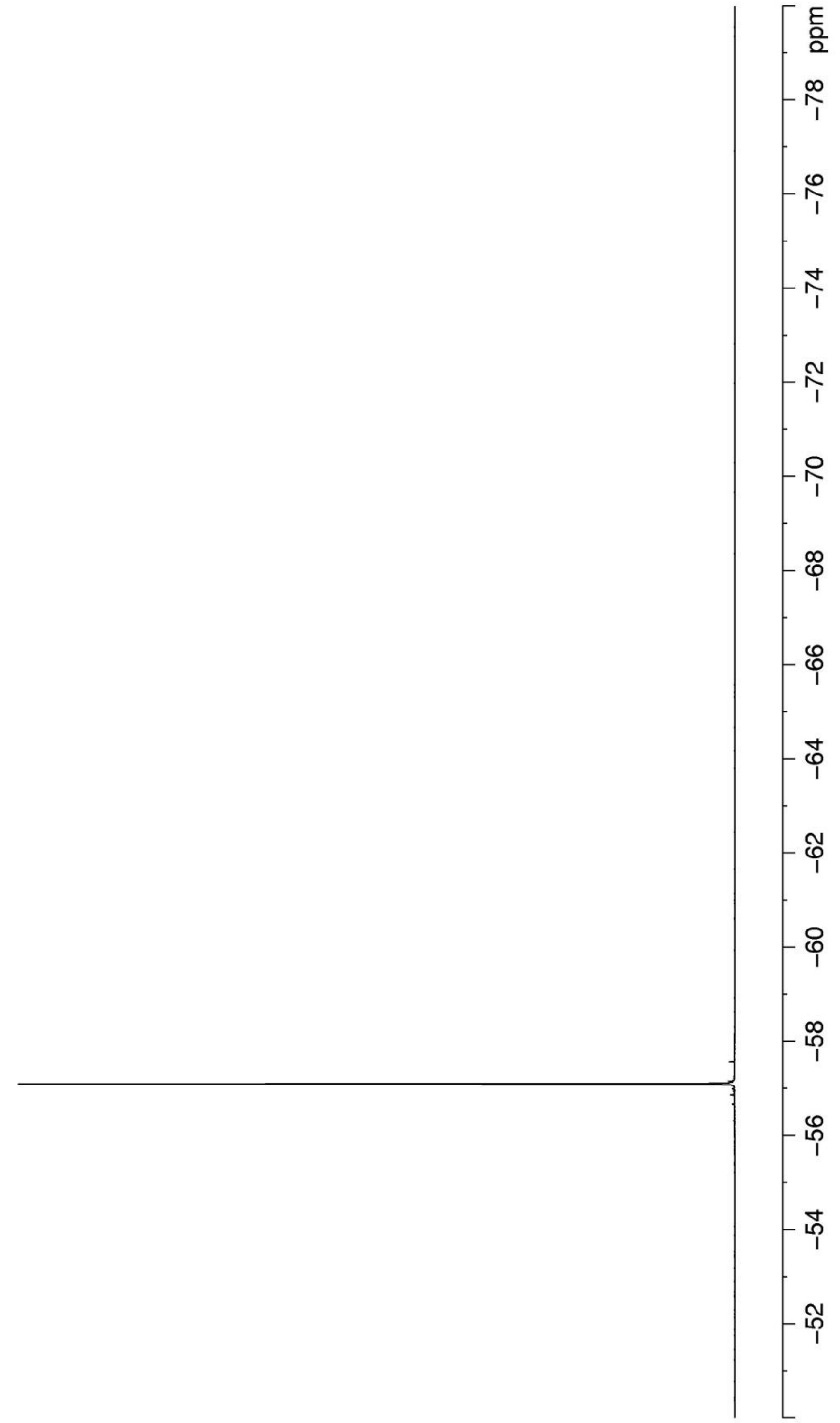

^{1}H NMR ($CDCl_3$, 25 ºC) of **6a**

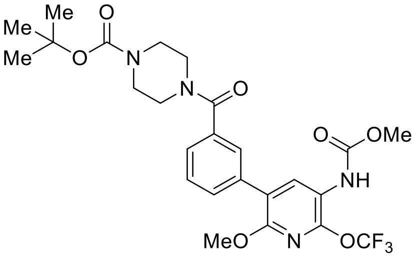

6a

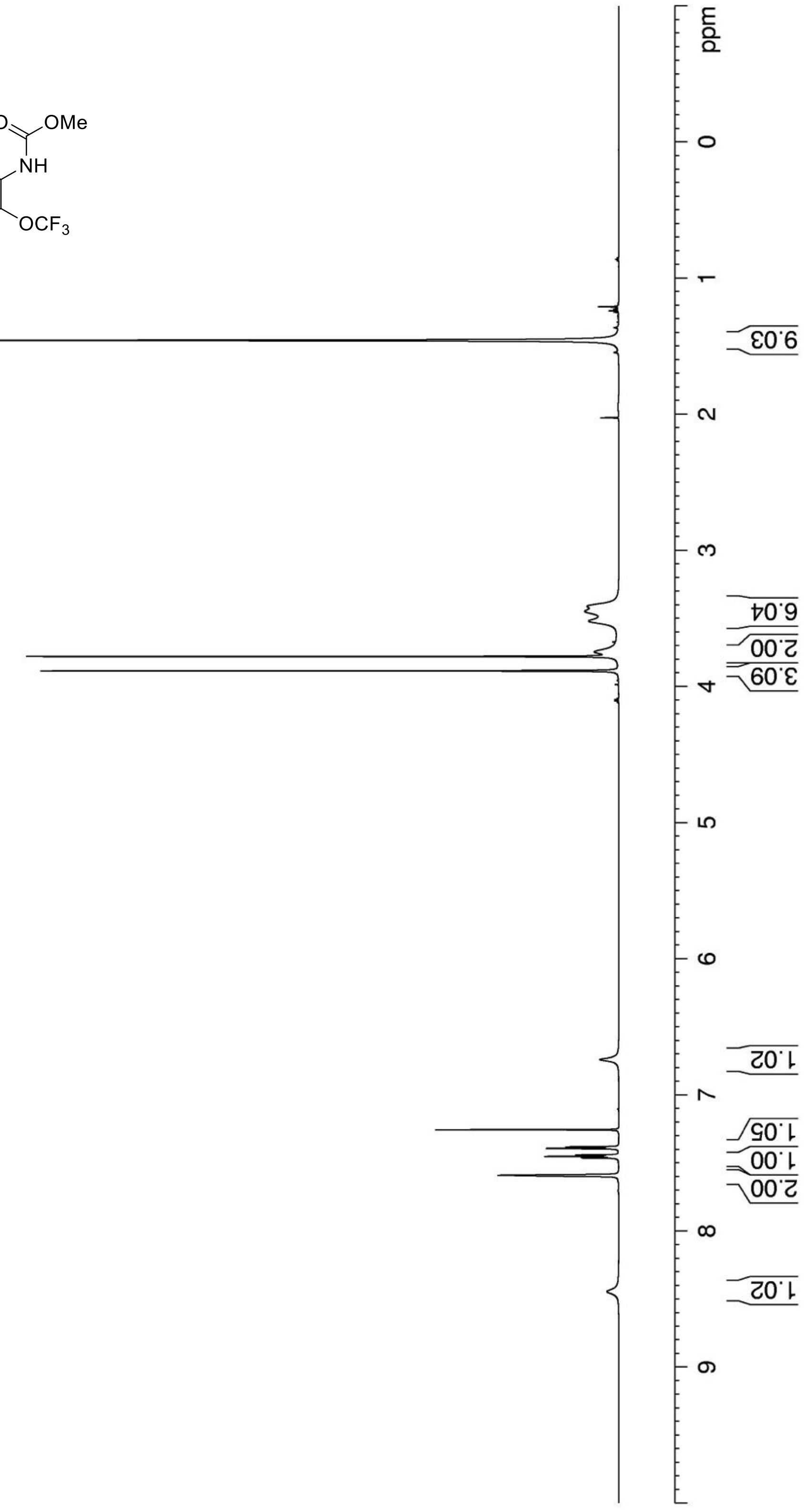

^{13}C NMR ($CDCl_3$, 25 ºC) of **6a**

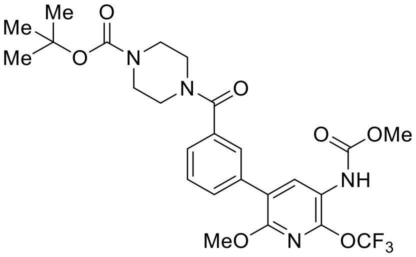

6a

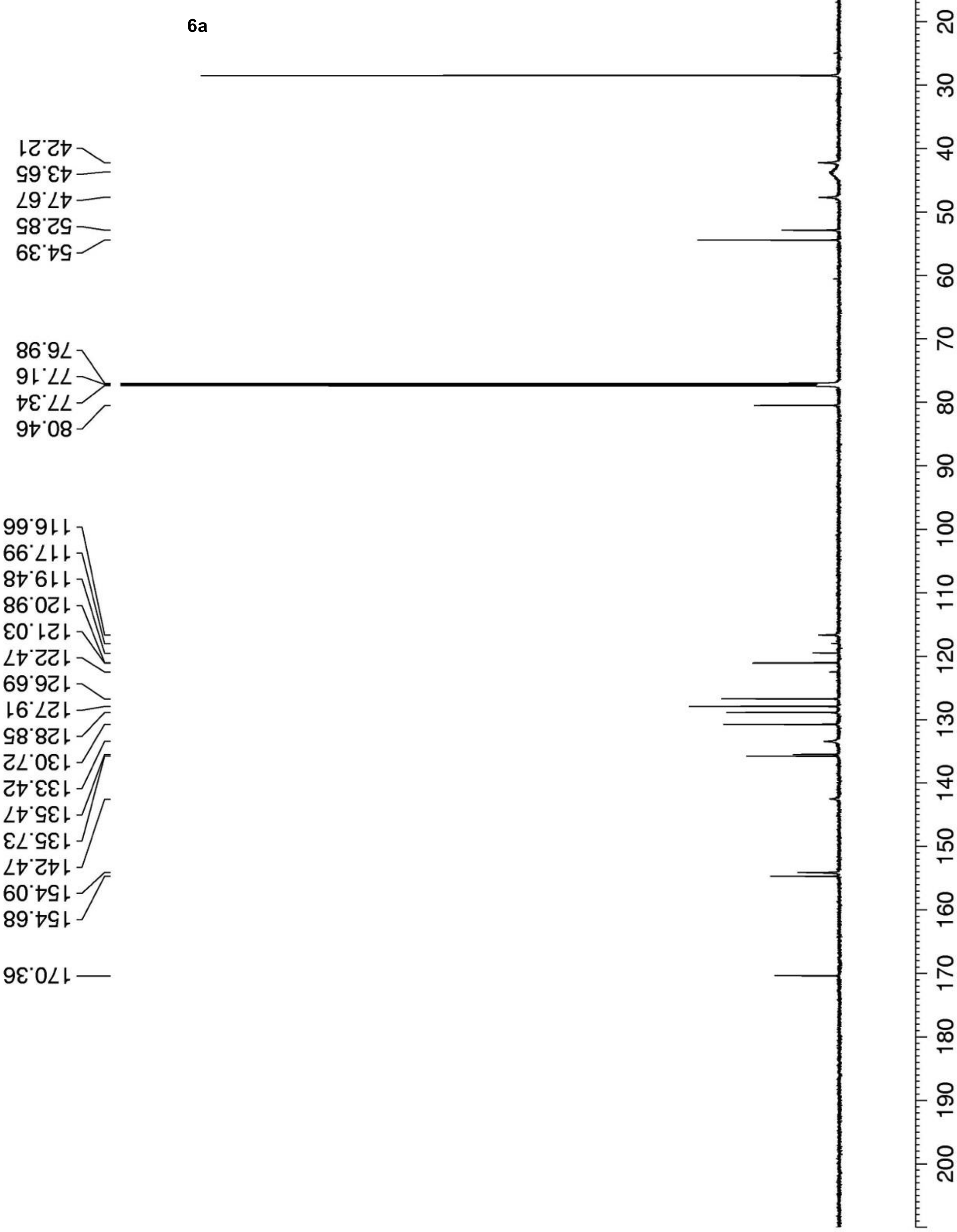

^{19}F NMR ($CDCl_3$, 25 ºC) of **6a**

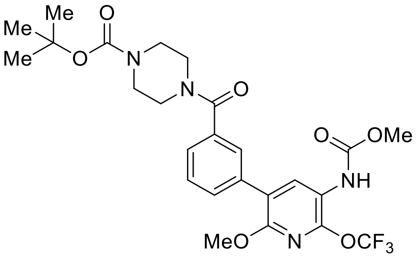

6a

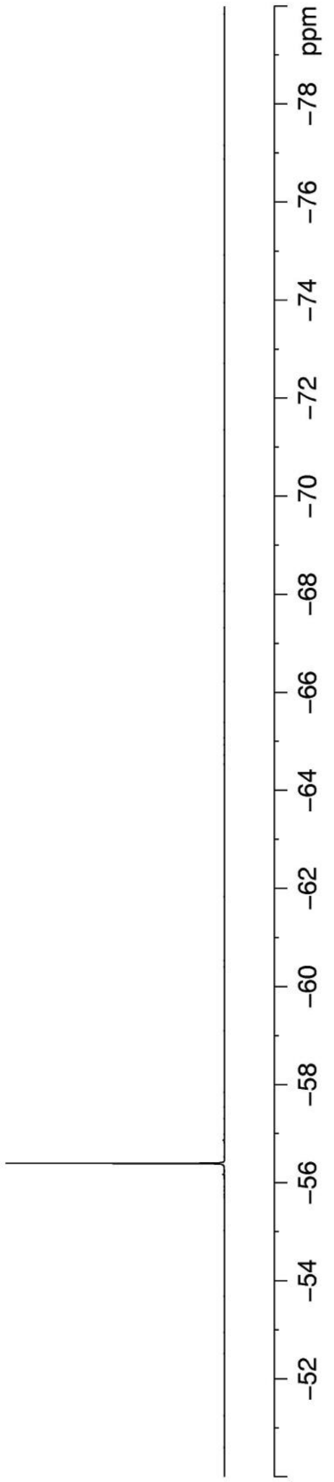

^{1}H NMR ($CDCl_3$, 25 ºC) of **7a**

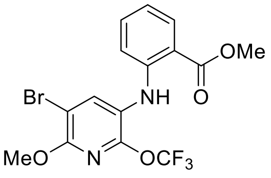

7a

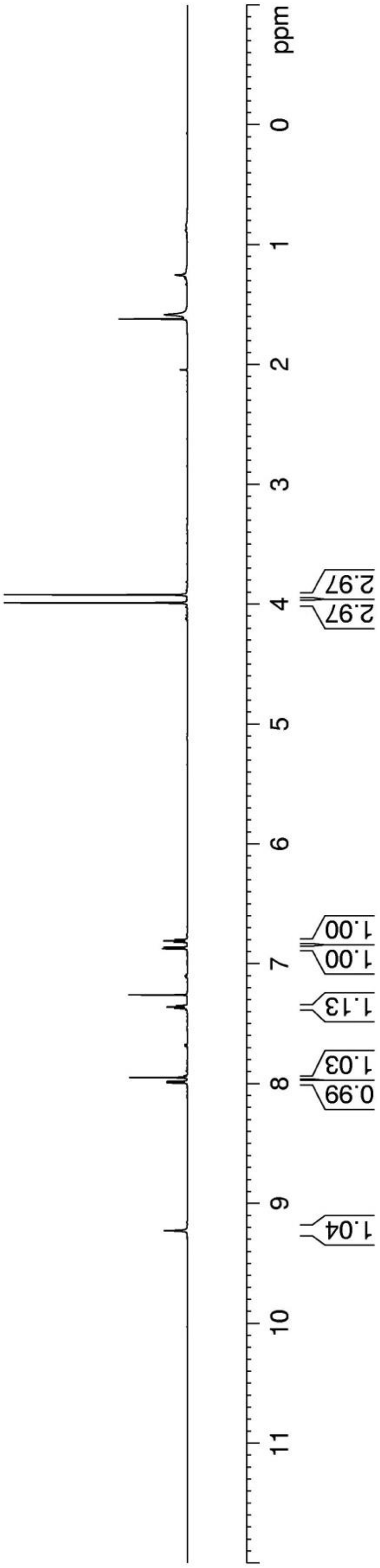

^{13}C NMR ($CDCl_3$, 25 ºC) of **7a**

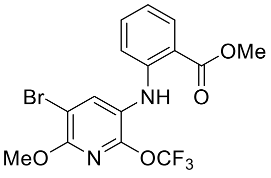

7a

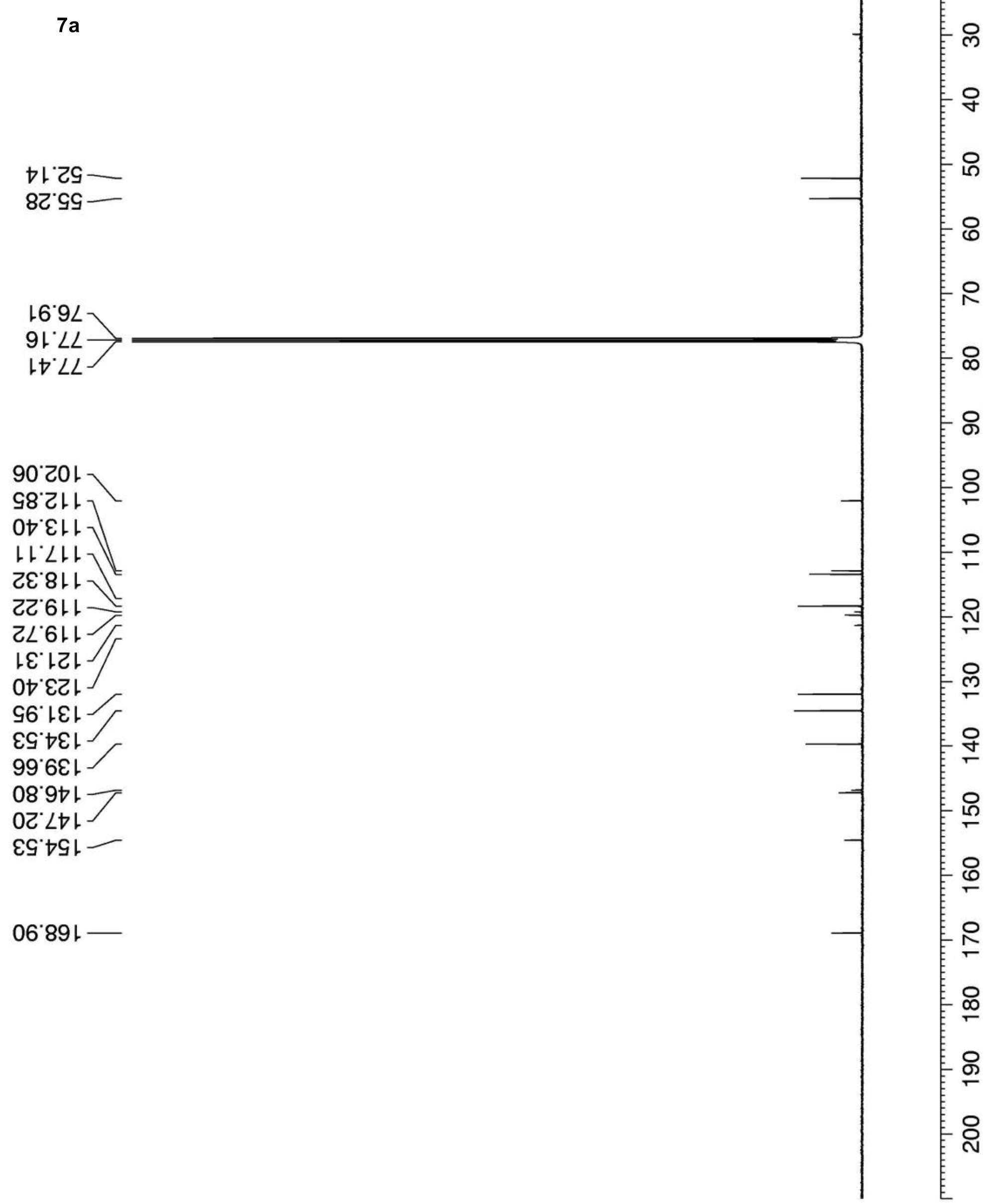

^{19}F NMR ($CDCl_3$, 25 ºC) of **7a**

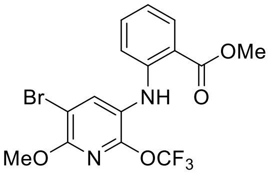

7a

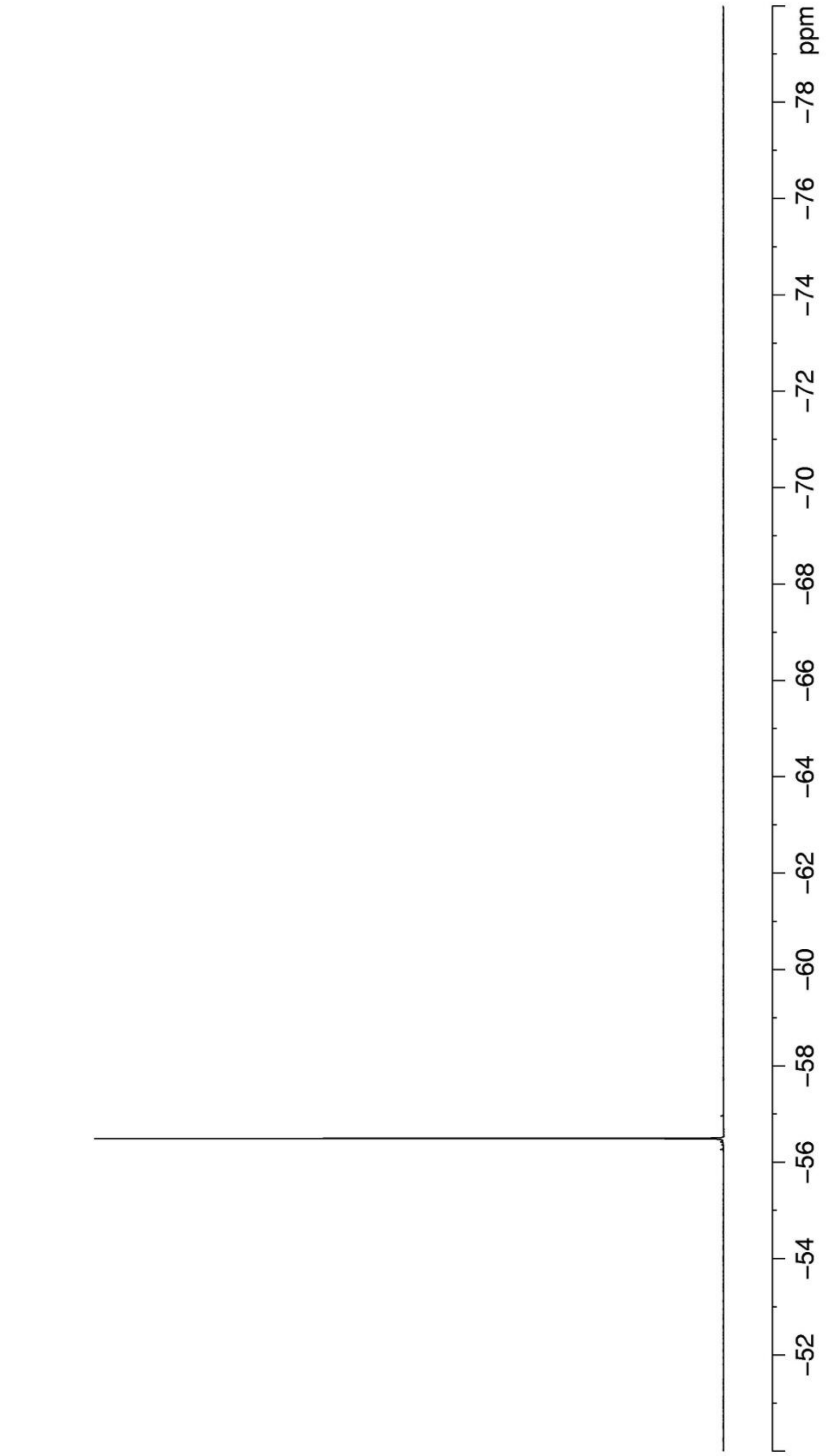

^{1}H NMR ($CDCl_3$, 25 ºC) of **8a**

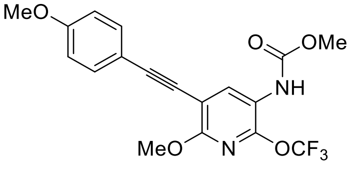

8a

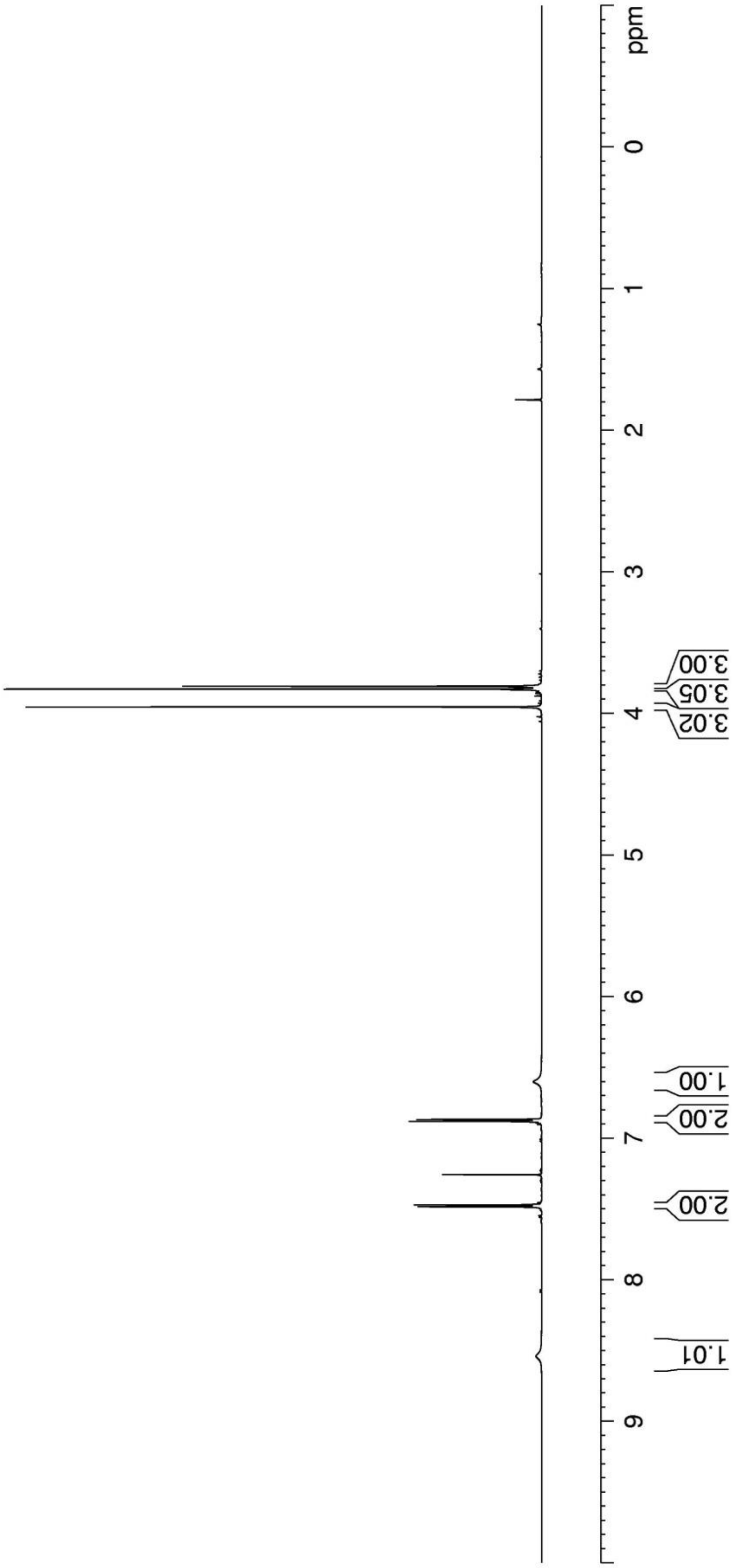

^{13}C NMR ($CDCl_3$, 25 ºC) of **8a**

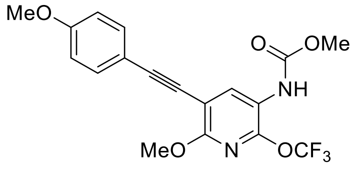

8a

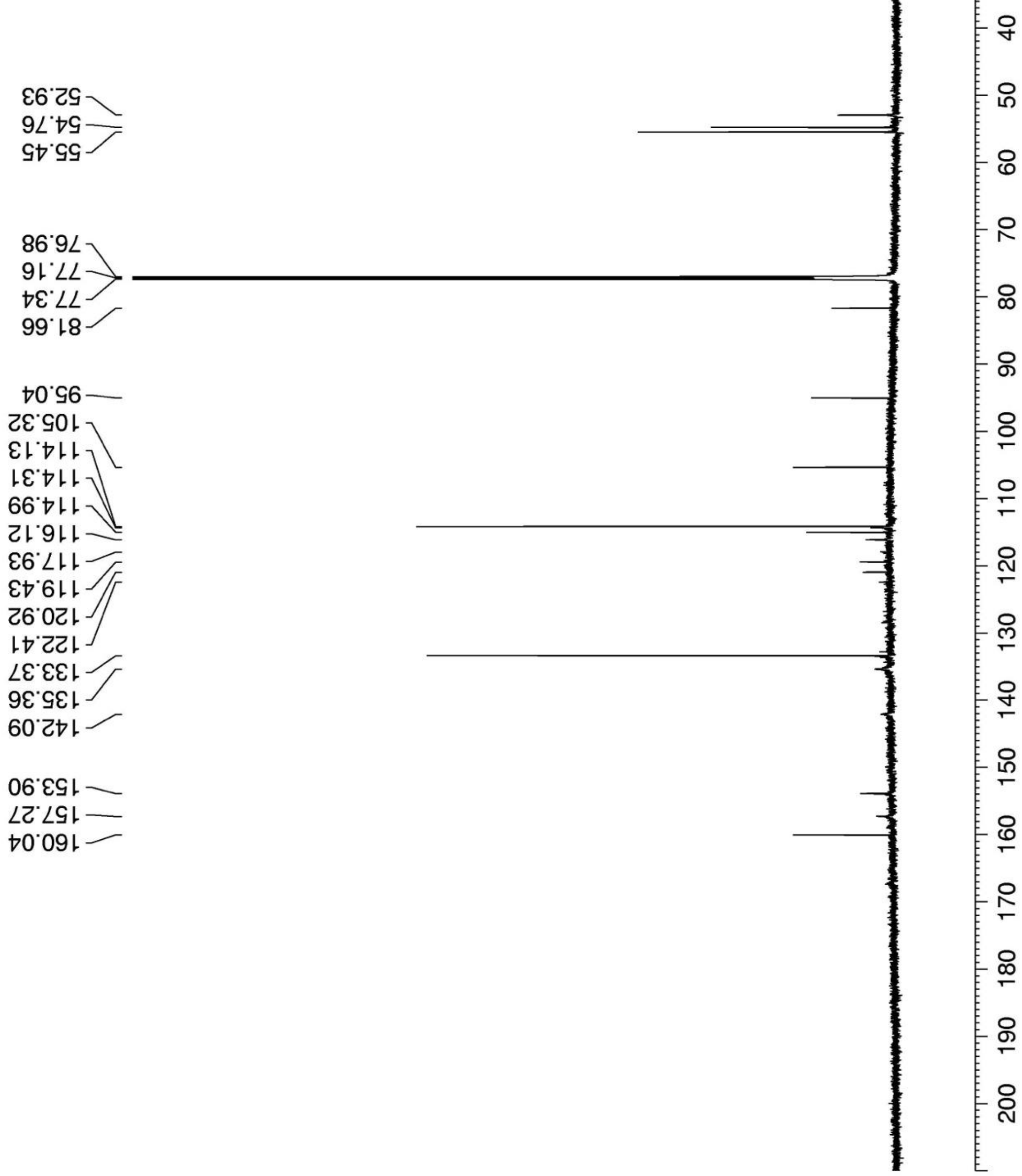

^{19}F NMR ($CDCl_3$, 25 ºC) of **8a**

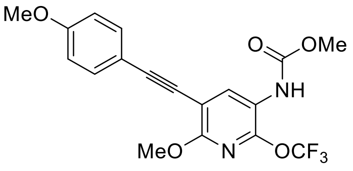

8a

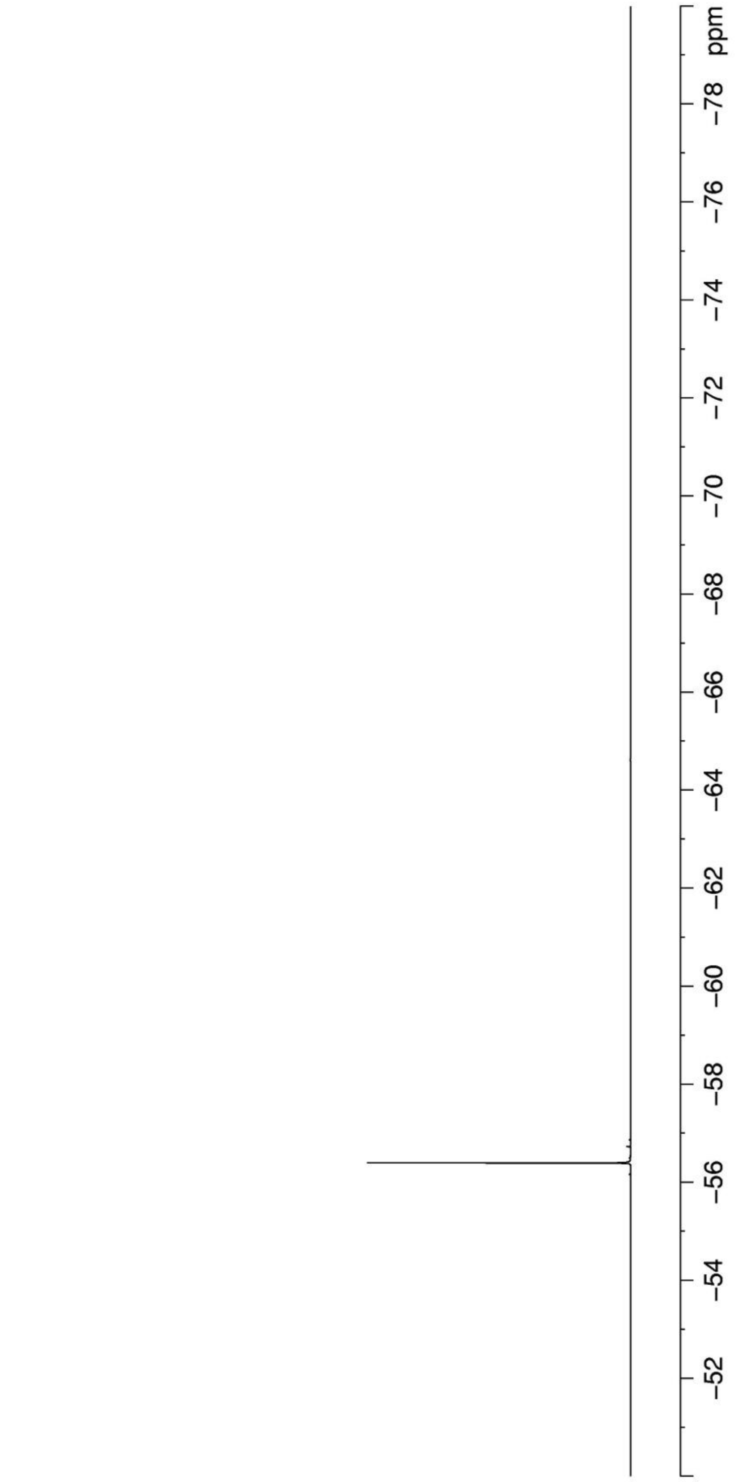

Supplement: Supplementary file 1 [file SC-007-C5SC02983J-s001.pdf]
